# Povorcitinib for hidradenitis suppurativa: the randomized, double-blind, placebo-controlled STOP-HS1 and STOP-HS2 phase 3 trials

**DOI:** 10.1038/s41591-026-04534-z

**Published:** 2026-07-23

**Authors:** Martina L. Porter, Antonio Martorell, Christopher J. Sayed, Falk G. Bechara, Hadar Lev-Tov, Jennifer L. Hsiao, John W. Frew, Ziad Reguiaï, Melinda J. Gooderham, Noah Goldfarb, Hessel H. van der Zee, Veronique del Marmol, Angelo V. Marzano, Benjamin D. Ehst, Lindsay S. Ackerman, Koremasa Hayama, Chenwei Tian, Jennifer Kelley, Huiling Zhen, Joslyn S. Kirby, Kurt Brown, Leandro L. Santos, Christos C. Zouboulis

**Affiliations:** 1 Department of Dermatology, Harvard Medical School, Boston, MA USA; 2 Beth Israel Deaconess Medical Center, Boston, MA USA; 3 Inflammatory Diseases Unit, Department of Dermatology, Venereology and Surgery, Hospital de Manises, Valencia, Spain; 4 Department of Dermatology, University of North Carolina School of Medicine, Chapel Hill, NC USA; 5 ICH – International Center for Hidradenitis Suppurativa/Acne Inversa, Department of Dermatology, Venereology and Allergology, Ruhr-University Bochum, Bochum, Germany; 6 Dr. Philip Frost Department of Dermatology and Cutaneous Surgery, University of Miami, Miami, FL USA; 7 Department of Dermatology, University of Southern California, Los Angeles, CA USA; 8 Laboratory of Translational Cutaneous Medicine, Ingham Institute for Applied Medical Research, Liverpool, New South Wales Australia; 9 Department of Dermatology, Liverpool Hospital, Liverpool, New South Wales Australia; 10 School of Clinical Medicine, University of New South Wales Sydney, Kensington, New South Wales Australia; 11 Department of Dermatology, Polyclinique Courlancy-Bezannes, Reims-Bezannes, France; 12 Probity Medical Research, Waterloo, Ontario Canada; 13 SKiN Centre for Dermatology, Peterborough, Ontario Canada; 14 Queen’s University, Kingston, Ontario Canada; 15 Departments of Dermatology and Medicine, University of Minnesota, Minneapolis, MN USA; 16 Department of Dermatology, University of Minnesota Physicians, Minneapolis, MN USA; 17 Department of Dermatology, Erasmus Medical Center, Rotterdam, Netherlands; 18 Department of Dermatology, Hôpital Erasme, Université Libre de Bruxelles, Brussels, Belgium; 19 Dermatology Unit, Fondazione IRCCS Ca’ Granda Ospedale Maggiore Policlinico, Milan, Italy; 20 Department of Pathophysiology and Transplantation, Università degli Studi di Milano, Milan, Italy; 21 Oregon Medical Research Center, Portland, OR USA; 22 USA Medical Dermatology Specialists, US Dermatology Partners, Phoenix, AZ USA; 23 Division of Cutaneous Science, Department of Dermatology, Nihon University School of Medicine, Itabashi City, Tokyo, Japan; 24 Incyte Corporation, Wilmington, DE USA; 25 Department of Dermatology, Venereology, and Immunology, Universitaetsklinikum Ruppin-Brandenburg, Brandenburg Medical School Theodor Fontane and Faculty of Health Sciences Brandenburg, Neuruppin, Germany

**Keywords:** Phase III trials, Inflammatory diseases

## Abstract

Although therapeutic options for hidradenitis suppurativa (HS) continue to expand, unmet needs remain substantial. We evaluated the efficacy and safety of povorcitinib (an oral, highly selective Janus kinase 1 (JAK1) inhibitor) in patients with moderate to severe HS in two randomized trials. STOP-HS1 and STOP-HS2 were identically designed, randomized, double-blind, placebo-controlled phase 3 trials. Adults with moderate to severe HS were randomized 2:2:1:1 to once-daily treatment through week 54: povorcitinib 45 mg; povorcitinib 75 mg; placebo with crossover at week 12 to povorcitinib 45 mg; or placebo with crossover at week 12 to povorcitinib 75 mg. The primary endpoint was HiSCR50 (≥50% decrease in total abscess and inflammatory nodule count, with no increase from baseline in abscess or draining tunnel count) at week 12. STOP-HS1/STOP-HS2 enrolled 608/619 patients, respectively. In both studies, both povorcitinib doses met the primary endpoint; in STOP-HS1, 82/204 (40%) patients in the povorcitinib 45-mg group and 82/202 (41%) in the 75-mg group versus 60/202 (30%) in the placebo group achieved HiSCR50 (OR (95% CI) 45 mg: 1.6 (1.1−2.5), *P* = 0.0240; 75 mg: 1.6 (1.1−2.4), *P* = 0.0214); corresponding values for STOP-HS2 were 88/208 (42%) and 88/208 (42%) versus 58/203 (29%; OR (95% CI) 45 mg: 1.8 (1.2−2.8), *P* = 0.0035; 75 mg: 1.9 (1.2−2.8); *P* = 0.0033). Through week 12, across STOP-HS1/STOP-HS2, serious treatment-emergent adverse events (AEs) occurred in 1−2% of patients across povorcitinib doses and in 2−3% with placebo; through week 54, serious AEs occurred in 5%/6% of patients receiving 45-mg/75-mg povorcitinib, respectively. The most frequent AEs among patients treated with 45-mg/75-mg povorcitinib were acne (17%/20%), nasopharyngitis (10%/12%) and upper respiratory tract infection (11%/10%). One death of undetermined etiology was reported and deemed unrelated to treatment. No new or unexpected safety findings were identified. Oral povorcitinib demonstrated significant improvements in HiSCR50 versus placebo and a favorable safety profile in patients with moderate to severe HS. ClinicalTrials.gov registration: NCT05620823 and NCT05620836.

## Abstract (web summary)

In a pair of pivotal phase 3, placebo-controlled, randomized clinical trials, the oral JAK1 inhibitor povorcitinib significantly reduced abscesses and inflammatory nodules in patients with moderate to severe hidradenitis suppurativa.

## Main

HS is a chronic inflammatory condition characterized by painful skin nodules, abscesses, draining tunnels and scarring that often affects intertriginous areas of the body^1,2^. Accurate estimates of disease prevalence have been challenging due to diagnostic variability and underrecognition^3^, but recent data suggest that HS affects approximately 0.7−1.5% of the global population, with substantial variation across geographic regions^4^. HS course is unpredictable and marked by flares^1^; common symptoms such as pain, itch, drainage, odor and fatigue lead to a profoundly negative impact on patients’ quality of life (QoL), including psychological distress, social isolation, functional impairment and reduced work productivity^1,5,6^. In addition, HS is associated with a substantial comorbidity burden, including increased rates of infections and cardiovascular complications such as major adverse cardiovascular events (MACE) and thromboembolic disease^7–9^.

Although therapeutic options for HS continue to expand^10^, unmet needs remain substantial^11^. Currently approved treatments for moderate to severe disease are limited to subcutaneously administered biologics: adalimumab (tumor necrosis factor (TNF) inhibitor), secukinumab (interleukin (IL)-17A inhibitor) and bimekizumab (IL-17A/IL-17F inhibitor)^12–14^. However, inadequate response or diminishing efficacy over time is common among treated patients^11,15^. In addition, patient preference studies indicate a strong interest in oral therapies, with aversion to injections (including needle phobia), potentially limiting uptake of injectable biologics^16,17^.

HS pathogenesis is multifactorial and involves immune dysregulation across multiple cell types and proinflammatory cytokines, including interferon-gamma (IFNγ), TNF, IL-6 and IL-17, many of which converge on the JAK−STAT pathway^1,18^. Targeting individual cytokines may, therefore, be insufficient in many patients^15^. By contrast, inhibition of JAK−STAT signaling, particularly JAK1, offers the potential to diminish signaling through multiple inflammatory pathways, which may be important in managing heterogeneous inflammatory diseases, including HS^1,18,19^.

Povorcitinib is an oral, highly selective JAK1 inhibitor in clinical development for several inflammatory conditions. In in vitro enzymatic and human cell-based assays, povorcitinib has demonstrated potent inhibition of key inflammatory cytokine signaling, including IL-2, IL-6 and IFNs, while sparing JAK2-dependent pathways^20^. In a dose-ranging phase 2 study of patients with HS, povorcitinib improved inflammatory lesion counts and HS symptoms and was generally well tolerated across doses^21^. Based on these findings, two phase 3 trials—Selective Treatment of Oral Povorcitinib in Hidradenitis Suppurativa studies 1 and 2 (STOP-HS1 and STOP-HS2)—were conducted to evaluate the efficacy and safety of povorcitinib over 54 weeks in adults with moderate to severe HS. Similar to previously reported phase 3 studies in HS, side-by-side results from each of these independent confirmatory clinical trials are presented here^13,14^.

## Results

### Study overview

**Extended Data Fig. 1:**
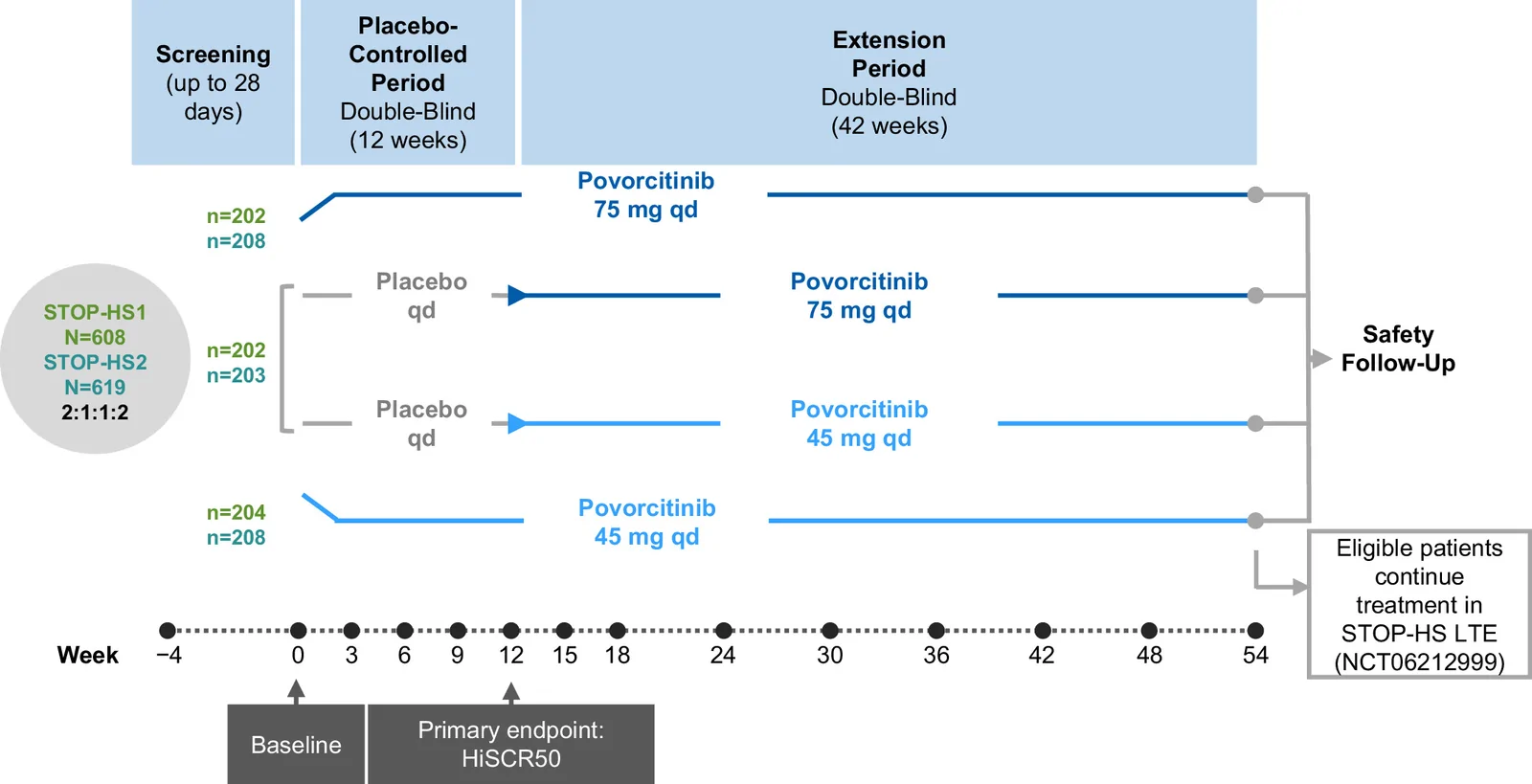
Study Design. At baseline, patients with moderate to severe HS were randomly assigned 2:2:1:1 to once-daily povorcitinib 45 mg; once-daily povorcitinib 75 mg; placebo with crossover to povorcitinib 45 mg at Week 12; or placebo with crossover to povorcitinib 75 mg at Week 12; the overall treatment period was 54 weeks. Patients who prematurely discontinued treatment during placebo-controlled period or extension period, or patients who did not continue treatment in the STOP-HS LTE study, entered the safety follow-up period (30 days). HiSCR=Hidradenitis Suppurativa Clinical Response; HiSCR50=at least 50% reduction from baseline in the total abscess and inflammatory nodule count, with no increase from baseline in abscess or draining tunnel count; HS=hidradenitis suppurativa; LTE=long-term extension; qd=once daily.

STOP-HS1 and STOP-HS2 were identically designed, global, multicenter, randomized, parallel-group, double-blind, placebo-controlled phase 3 trials of povorcitinib in HS conducted at 103 sites (STOP-HS1) and 99 sites (STOP-HS2) across Australia, Canada, Europe, Japan and the United States. Eligible patients were at least 18 years of age with a diagnosis of moderate to severe HS (at least one anatomical area assessed as Hurley stage II or III) for at least 3 months before screening; had a total abscess and inflammatory nodule count of at least five; had HS lesions present in at least two distinct anatomical areas; and had an inadequate response (after at least a 3-month treatment period) to a prior HS systemic therapy (oral antibiotic or biologic drug) or intolerance or contraindication to such therapy. Patients were randomized 2:2:1:1 to once-daily povorcitinib 45 mg; povorcitinib 75 mg; placebo with crossover to povorcitinib 45 mg at week 12; or placebo with crossover to povorcitinib 75 mg at week 12. The study had two periods, both double-blinded: a 12-week placebo-controlled period and a 42-week extension period, for a total of 54 weeks (Extended Data Fig. 1).

In both trials, the primary endpoint was HiSCR50 (reduction of at least 50% from baseline in total abscess and inflammatory nodule count, with no increase from baseline in abscess or draining tunnel count) at week 12. Key secondary endpoints were HiSCR75 (reduction of ≥75% from baseline in total abscess and inflammatory nodule count, with no increase from baseline in abscess or draining tunnel count) at week 12; skin pain response among patients with baseline Skin Pain Numerical Rating Scale (NRS) score of at least 3 (response defined either as 3-point decrease from baseline or as NRS30, defined as at least 30% decrease and 1-unit decrease from baseline) at week 12; and occurrence of flares (defined as ≥25% and ≥2-unit increase in abscess and inflammatory nodule count relative to baseline) by week 12. Secondary endpoints included the percentage of patients with baseline Functional Assessment of Chronic Illness Therapy–Fatigue (FACIT-F) score ≤48 with ≥4-point increase (improvement) at week 12 and the percentage achieving HiSCR50 and HiSCR75 at week 12 and maintaining it at each visit during the extension period. Percentages of patients achieving HiSCR50, HiSCR75 or skin pain response or experiencing a flare were assessed at week 24 and week 54. Change from baseline in Dermatology Life Quality Index (DLQI) and change/percentage change from baseline in lesion counts (abscess, inflammatory nodule and draining tunnel) were evaluated at each visit through week 54. Prespecified exploratory outcomes evaluating the long-term efficacy of povorcitinib included HiSCR50 and HiSCR75 as well as HiSCR90 and HiSCR100 (reduction of ≥90% or 100% from baseline in total abscess and inflammatory nodule count, with no increase from baseline in abscess or draining tunnel count).

### Patients

**Fig. 1:**
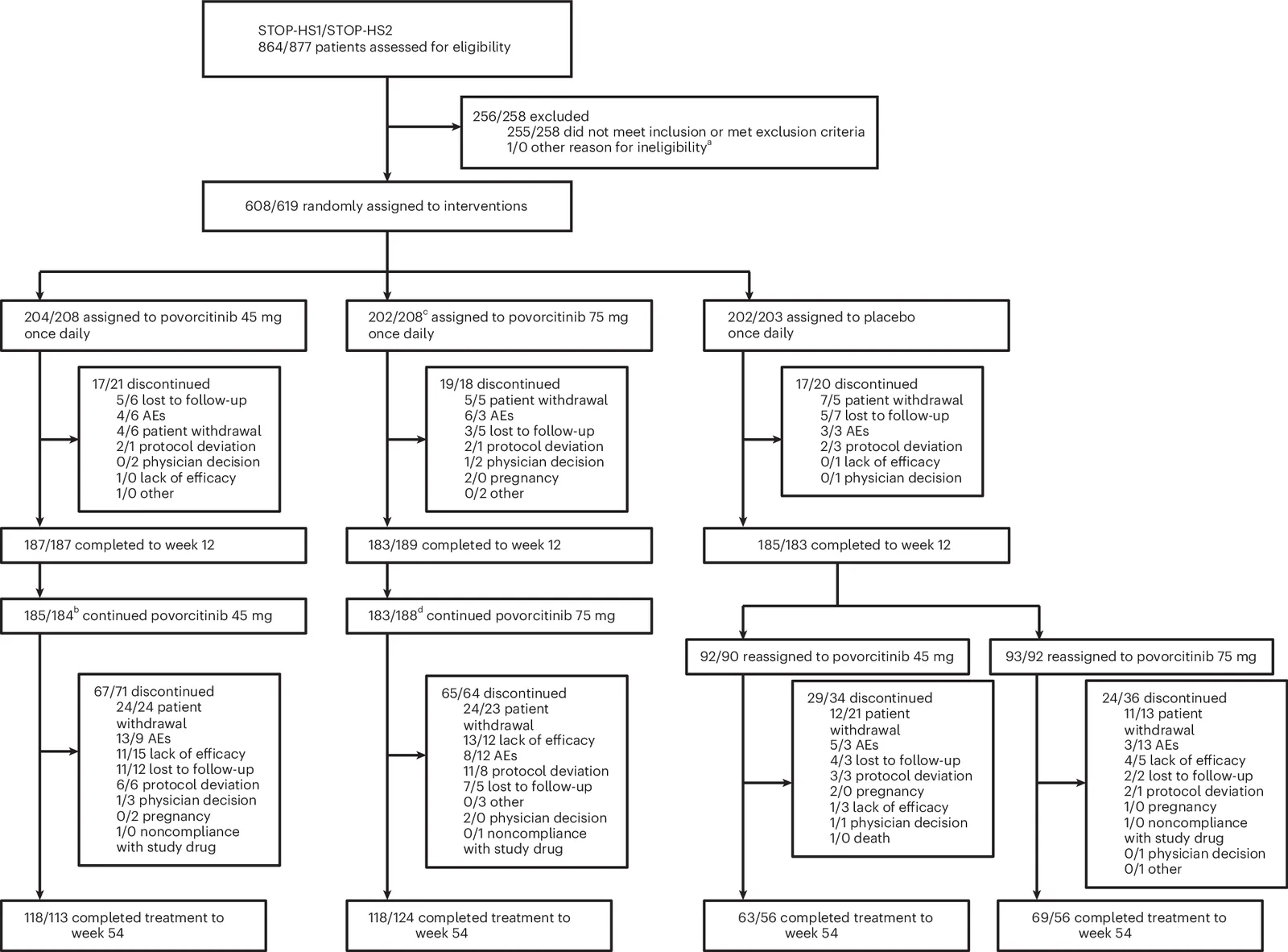
Trial profiles. STOP-HS1 and STOP-HS2 trial profiles to week 54. ^a^ Study drug was not available for dispensing to this patient within 28 days of the screening visit. ^b^ In STOP-HS1, two patients discontinued after completing treatment through week 12: one was lost to follow-up, and one withdrew. In STOP-HS2, three patients discontinued after completing treatment through week 12: two discontinued due to physician decision, and one withdrew. ^c^ In STOP-HS2, one patient was randomized but discontinued the study before receiving treatment. ^d^ One patient withdrew after completing treatment through week 12.

STOP-HS1 was conducted from 19 December 2022 (first patient dosed) to 23 December 2025 (last patient with last study visit completed), and STOP-HS2 was conducted from 22 February 2023 (first patient dosed) to 21 November 2025 (last patient with last study visit completed). Of the 864 patients screened in STOP-HS1, 608 were randomized to povorcitinib 45 mg (*n* = 204), to povorcitinib 75 mg (*n* = 202) or to placebo (*n* = 202). Of the 877 patients screened in STOP-HS2, 619 were randomized to povorcitinib 45 mg (*n* = 208), to povorcitinib 75 mg (*n* = 208) or to placebo (*n* = 203). A total of 553 patients in STOP-HS1 and 554 patients in STOP-HS2 entered the extension period, of which 368 (67%) and 349 (63%), respectively, completed through week 54 (Fig. 1).

Baseline demographics were consistent with patients with moderate to severe HS. Across STOP-HS1 and STOP-HS2, most patients were female and White, with a substantial proportion of Black patients (33% in the United States; Supplementary Table 1). Nearly half were current smokers, and mean (s.d.) body mass index (BMI) was 33.3 (8.2) to 35.2 (8.8) kg m^−^^2^, with a high proportion of severe obesity (18−27% with BMI ≥ 40 kg m^−^^2^). Baseline disease characteristics were indicative of recalcitrant, longstanding HS (mean (range) disease duration, 9.7 (0.4−49.1)−10.8 (0.4–57.7) years), with a high proportion of prior biologic use for HS (35−39%) and prior HS local excision surgery (18−24%) and mean (s.d.) abscess and inflammatory nodule counts of 11.1 (7.1) to 13.0 (11.2) and draining tunnel counts of 2.5 (3.0) to 3.0 (3.5); 74−76% of patients had at least one draining tunnel at baseline. Symptom and QoL burdens at baseline were substantial, with mean (s.d.) Skin Pain NRS and hidradenitis suppurativa quality of life (HiSQoL) total score of 4.9 (2.5) to 5.2 (2.5) and 30.9 (15.6) to 32.5 (15.0), respectively (Table 1).

**Table 1:** Baseline demographic and disease characteristics in STOP-HS1 and STOP-HS2

Characteristic	STOP-HS1 (*N* = 608)			STOP-HS2 (*N* = 619)		
	Povorcitinib 45 mg (*n* = 204)	Povorcitinib 75 mg (*n* = 202)	Placebo (*n* = 202)	Povorcitinib 45 mg (*n* = 208)	Povorcitinib 75 mg (*n* = 208)	Placebo (*n* = 203)
Age, years	37.5 (18–76)	38.4 (18–77)	37.0 (18–71)	37.4 (18–70)	35.6 (18–68)	37.8 (18–68)
Age group, years						
18 to <35	86 (42%)	80 (40%)	87 (43%)	82 (39%)	102 (49%)	83 (41%)
35 to <50	91 (45%)	87 (43%)	87 (43%)	98 (47%)	83 (40%)	84 (41%)
≥50	27 (13%)	35 (17%)	28 (14%)	28 (13%)	23 (11%)	36 (18%)
Sex						
Female	131 (64%)	134 (66%)	138 (68%)	134 (64%)	123 (59%)	110 (54%)
Male	73 (36%)	68 (34%)	64 (32%)	74 (36%)	85 (41%)	93 (46%)
Race						
White	139 (68%)	147 (73%)	152 (75%)	156 (75%)	159 (76%)	162 (80%)
Black	43 (21%)	26 (13%)	29 (14%)	30 (14%)	25 (12%)	21 (10%)
Asian	12 (6%)	14 (7%)	8 (4%)	3 (1%)	0	7 (3%)
Body weight, kg	101.1 (25.4)	97.7 (26.0)	100.2 (26.5)	97.1 (26.1)	99.1 (26.3)	97.9 (25.4)
BMI, kg m^−^^2^	35.2 (8.8)	33.7 (8.6)	34.9 (9.3)	33.4 (8.3)	33.5 (8.6)	33.3 (8.2)
BMI category, kg m^−^^2^						
<30	58 (28%)	78 (39%)	67 (33%)	84 (40%)	80 (38%)	81 (40%)
30 to <40	91 (45%)	85 (42%)	81 (40%)	87 (42%)	86 (41%)	80 (39%)
≥40	55 (27%)	39 (19%)	54 (27%)	37 (18%)	42 (20%)	42 (21%)
Smoking status						
Current	101 (50%)	100 (50%)	96 (48%)	98 (47%)	91 (44%)	94 (46%)
Former	32 (16%)	42 (21%)	34 (17%)	34 (16%)	32 (15%)	34 (17%)
Medical cannabis use						
Current	32 (16%)	34 (17%)	26 (13%)	29 (14%)	29 (14%)	25 (12%)
Former	8 (4%)	12 (6%)	14 (7%)	14 (7%)	18 (9%)	10 (5%)
Previous use of biologic therapy						
Any biologic	74 (36%)	71 (35%)	73 (36%)	82 (39%)	80 (38%)	77 (38%)
≥2 biologics	20 (10%)	26 (13%)	18 (9%)	18 (9%)	22 (11%)	17 (8%)
TNF inhibitor	60 (29%)	56 (28%)	51 (25%)	65 (31%)	67 (32%)	65 (32%)
IL-17 inhibitor	30 (15%)	32 (16%)	32 (16%)	27 (13%)	27 (13%)	27 (13%)
Other biologic	7 (3%)	13 (6%)	13 (6%)	9 (4%)	12 (6%)	4 (2%)
Prior HS surgery						
Any HS surgery	91 (45%)	87 (43%)	81 (40%)	94 (45%)	102 (49%)	101 (50%)
Incision and drainage	55 (27%)	49 (24%)	46 (23%)	46 (22%)	49 (24%)	52 (26%)
Local excision	41 (20%)	43 (21%)	37 (18%)	42 (20%)	44 (21%)	49 (24%)
Deroofing	9 (4%)	15 (7%)	6 (3%)	16 (8%)	16 (8%)	10 (5%)
Disease duration, years	10.6 (0.4–62.6)	10.2 (0.3–51.1)	10.8 (0.3–48.7)	10.8 (0.4–57.7)	9.7 (0.4–49.1)	9.7 (0.4–53.3)
HS family history	42 (21%)	46 (23%)	53 (26%)	54 (26%)	52 (25%)	38 (19%)
Abscess and inflammatory nodule count	13.0 (11.2)	12.1 (8.3)	12.3 (8.2)	11.6 (8.4)	12.0 (9.0)	11.1 (7.1)
Abscess count	2.4 (3.9)	2.2 (3.9)	2.2 (3.7)	2.1 (2.9)	1.9 (3.5)	2.5 (3.8)
Inflammatory nodule count	10.7 (10.2)	9.9 (7.8)	10.1 (7.0)	9.5 (7.8)	10.2 (7.7)	8.5 (5.3)
Draining tunnel count	3.0 (3.5)	2.8 (3.5)	2.9 (3.4)	2.7 (3.2)	2.5 (3.0)	2.9 (3.4)
≥1 draining tunnel	156 (76%)	150 (74%)	152 (75%)	158 (76%)	155 (75%)	155 (76%)
Abscess, inflammatory nodule and draining tunnel count, category						
<11	88 (43%)	85 (42%)	87 (43%)	96 (46%)	90 (43%)	89 (44%)
≥11	116 (57%)	117 (58%)	115 (57%)	112 (54%)	118 (57%)	114 (56%)
Hurley stage						
II	128 (63%)	127 (63%)	118 (58%)	135 (65%)	144 (69%)	144 (71%)
III	76 (37%)	75 (37%)	84 (42%)	73 (35%)	64 (31%)	59 (29%)
IHS4 score	27.2 (23.2)	25.6 (20.8)	26.0 (20.4)	24.5 (16.5)	24.0 (17.6)	25.4 (19.5)
Skin Pain NRS	5.2 (2.5)	5.2 (2.3)	5.0 (2.7)	5.0 (2.5)	5.0 (2.5)	4.9 (2.5)
Skin Pain NRS score ≥3	157 (77%)	158 (78%)	149 (74%)	165 (79%)	159 (76%)	151 (74%)
Itch NRS	4.6 (2.5)	4.6 (2.4)	4.5 (2.6)	4.5 (2.6)	4.4 (2.6)	4.1 (2.6)
Itch NRS score ≥3	138 (68%)	135 (67%)	138 (68%)	146 (70%)	140 (67%)	127 (63%)
HiSQoL total score	30.9 (15.6)	31.6 (14.2)	31.6 (15.2)	32.5 (15.0)	31.9 (13.5)	31.4 (14.5)
Severe or very severe category (total score 22−68)	138 (67%)	147 (72%)	142 (70%)	155 (75%)	162 (78%)	139 (69%)
DLQI total score	12.7 (7.4)	12.8 (7.2)	12.8 (7.6)	13.9 (7.5)	12.9 (6.9)	12.5 (7.1)
Very large or extremely large effect category (total score 11−30)	114 (56%)	114 (57%)	111 (55%)	135 (65%)	125 (60%)	118 (58%)
FACIT-F score	30.7 (12.0)	31.1 (12.2)	32.3 (12.3)	30.1 (12.6)	31.0 (11.8)	32.4 (11.5)
hsCRP, mg l^−1^	17.2 (26.5)	15.7 (25.1)	16.0 (23.6)	13.8 (21.7)	14.6 (22.7)	16.4 (26.1)
Concomitant GLP-1 inhibitor	7 (3%)	13 (6%)	11 (5%)	8 (4%)	12 (6%)	11 (5%)
Concomitant oral contraceptives^a^	20 (15%)	20 (15%)	22 (16%)	22 (16%)	30 (24%)	15 (14%)
Select medical history^b^						
COVID-19	83 (41%)	79 (39%)	77 (38%)	98 (47%)	94 (45%)	100 (49%)
Varicella	77 (38%)	77 (38%)	75 (37%)	88 (42%)	101 (49%)	102 (50%)
Acne	62 (30%)	80 (40%)	82 (41%)	86 (41%)	97 (47%)	84 (41%)
Obesity	60 (29%)	60 (30%)	79 (39%)	56 (27%)	60 (29%)	60 (30%)
Hypertension	46 (23%)	37 (18%)	50 (25%)	44 (21%)	35 (17%)	52 (26%)
Depression	36 (18%)	44 (22%)	41 (20%)	45 (22%)	38 (18%)	31 (15%)
Anxiety	33 (16%)	32 (16%)	51 (25%)	43 (21%)	38 (18%)	28 (14%)
Asthma	30 (15%)	19 (9%)	29 (14%)	23 (11%)	17 (8%)	21 (10%)
Seasonal/environmental allergy	21 (10%)	22 (11%)	33 (16%)	25 (12%)	27 (13%)	21 (10%)
Gastroesophageal reflux disease	24 (12%)	25 (12%)	22 (11%)	22 (11%)	20 (10%)	15 (7%)
Diabetes mellitus	18 (9%)	18 (9%)	30 (15%)	16 (8%)	12 (6%)	31 (15%)
Hyperlipidemia	15 (7%)	20 (10%)	27 (13%)	22 (11%)	14 (7%)	22 (11%)
Polycystic ovarian syndrome	16 (8%)	15 (7%)	17 (8%)	19 (9%)	18 (9%)	9 (4%)
Anemia	18 (9%)	11 (5%)	18 (9%)	20 (10%)	9 (4%)	13 (6%)
Psoriasis	22 (11%)	7 (3%)	8 (4%)	9 (4%)	8 (4%)	9 (4%)
Pilonidal disease	9 (4%)	11 (5%)	4 (2%)	12 (6%)	14 (7%)	9 (4%)
Herpes zoster	7 (3%)	11 (5%)	8 (4%)	2 (1%)	6 (3%)	12 (6%)
Arthritis	7 (3%)	5 (2%)	7 (3%)	6 (3%)	7 (3%)	7 (3%)
Crohn’s disease	0	0	2 (1%)	2 (1%)	3 (1%)	0
Ulcerative colitis	1 (<1%)	0	0	1 (<1%)	0	2 (1%)

Data are mean (range), mean (s.d.) or *n* (%). Percentages might not add up to 100% due to rounding.

^a^ Percentages calculated relative to the count of female patients.

^b^ Includes events grouped using a custom preferred-term search based on MedDRA version 27.1.

GLP-1, glucagon-like peptide-1.

### Primary endpoint

**Fig. 2:**
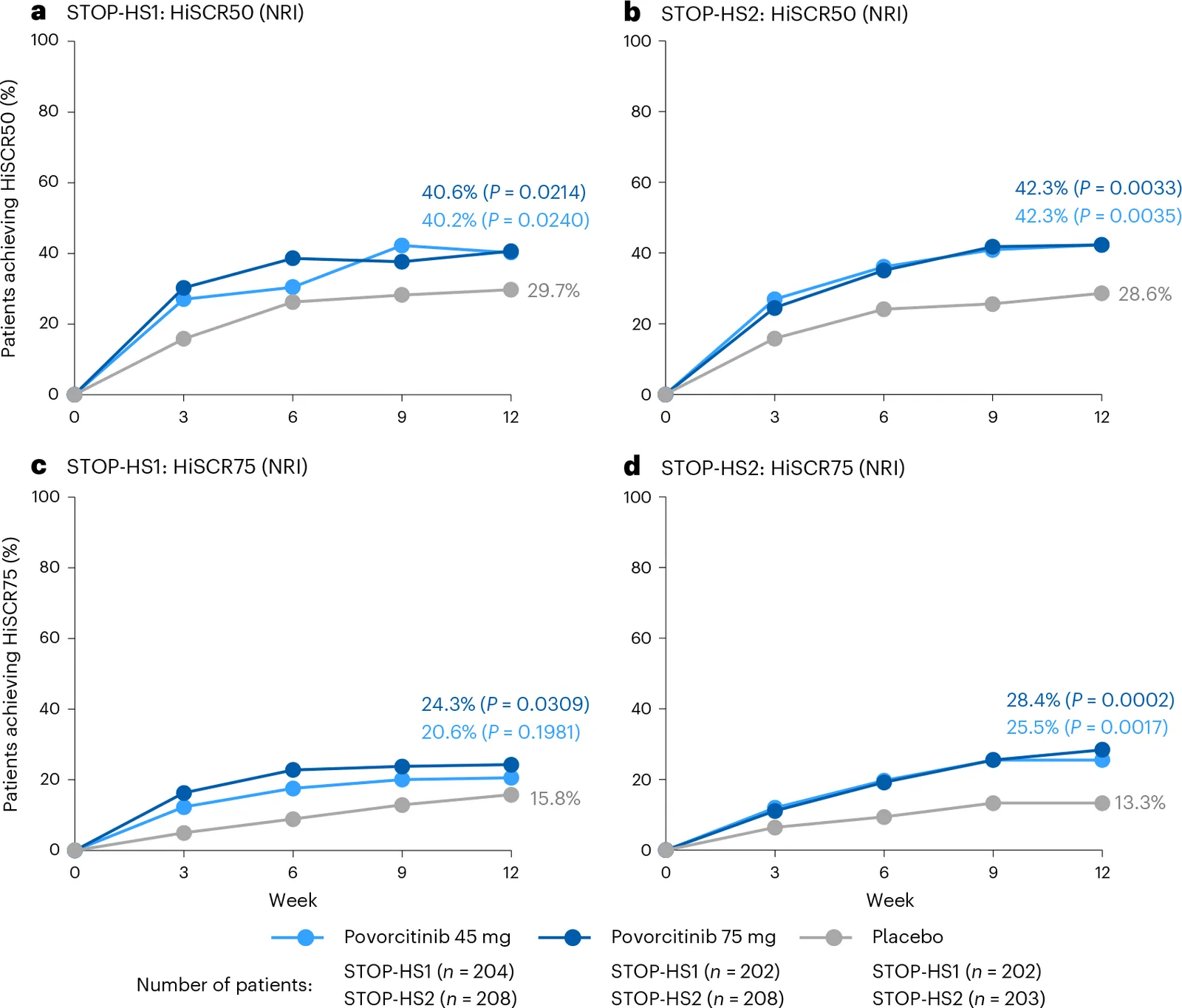
HiSCR responses from randomization to week 12. Rates of HiSCR50 (**a**,**b**) and HiSCR75 (**c**,**d**) from randomization to week 12 in STOP-HS1 and STOP-HS2. Superiority of each povorcitinib dose (45 mg and 75 mg) versus placebo was assessed separately, at week 12, using a Cochran−Mantel−Haenszel test stratified by randomization factors, with *P* values representing comparisons versus placebo. The overall familywise type I error was controlled at a two-sided *α* level of 0.05. A graphical approach was applied to adjust for multiplicity. Patients with missing post-baseline values or who received other systemic treatment with potential effect on HS outcomes were imputed as nonresponders.

**Fig. 3:**
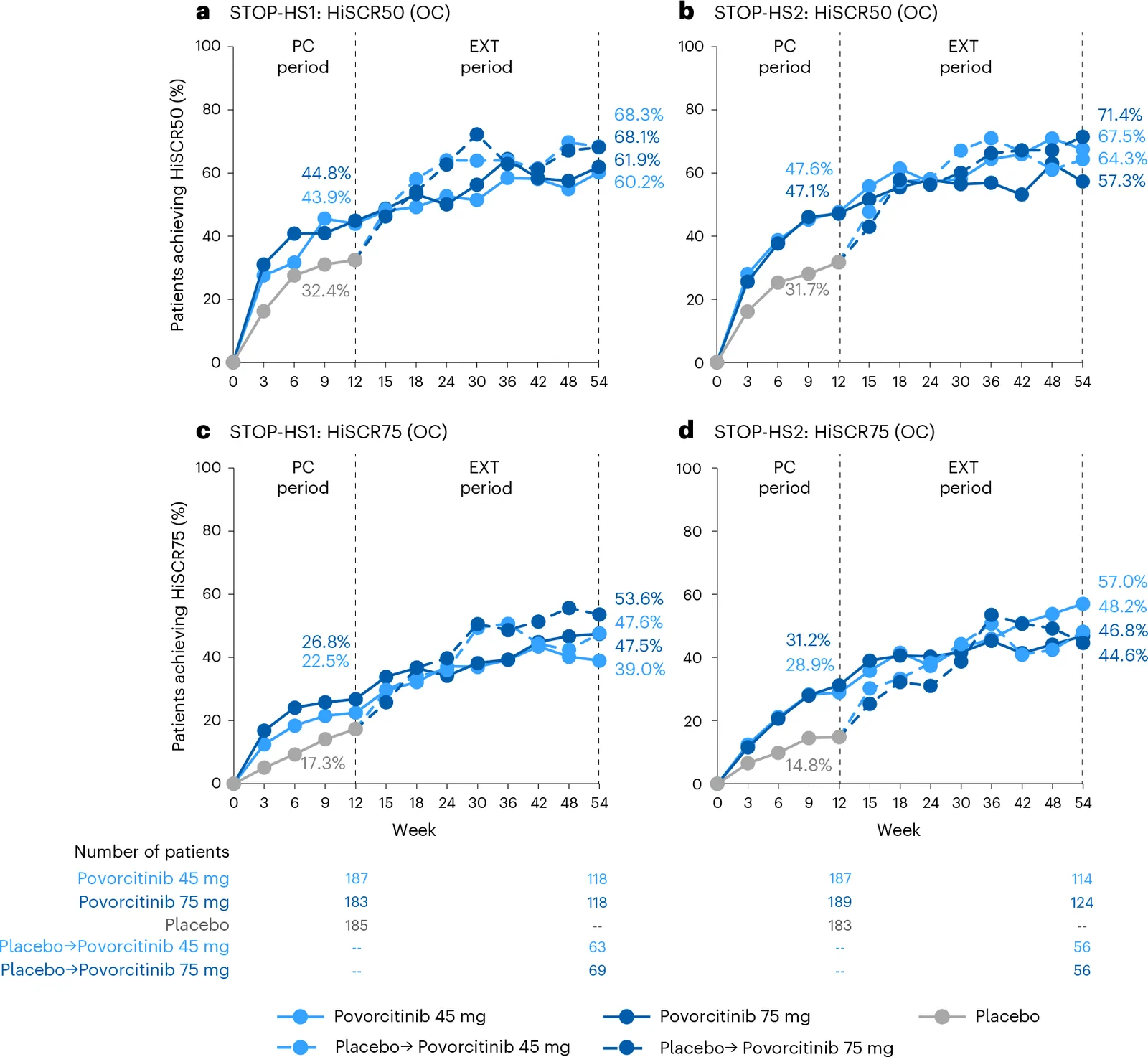
HiSCR responses from randomization to week 54. Rates of HiSCR50 (**a**,**b**) and HiSCR75 (**c**,**d**) from randomization to week 54 in STOP-HS1 and STOP-HS2. Data are shown for observed cases and summarized descriptively. EXT, extension; PC, placebo-controlled; OC, observed case.

**Extended Data Fig. 2:**
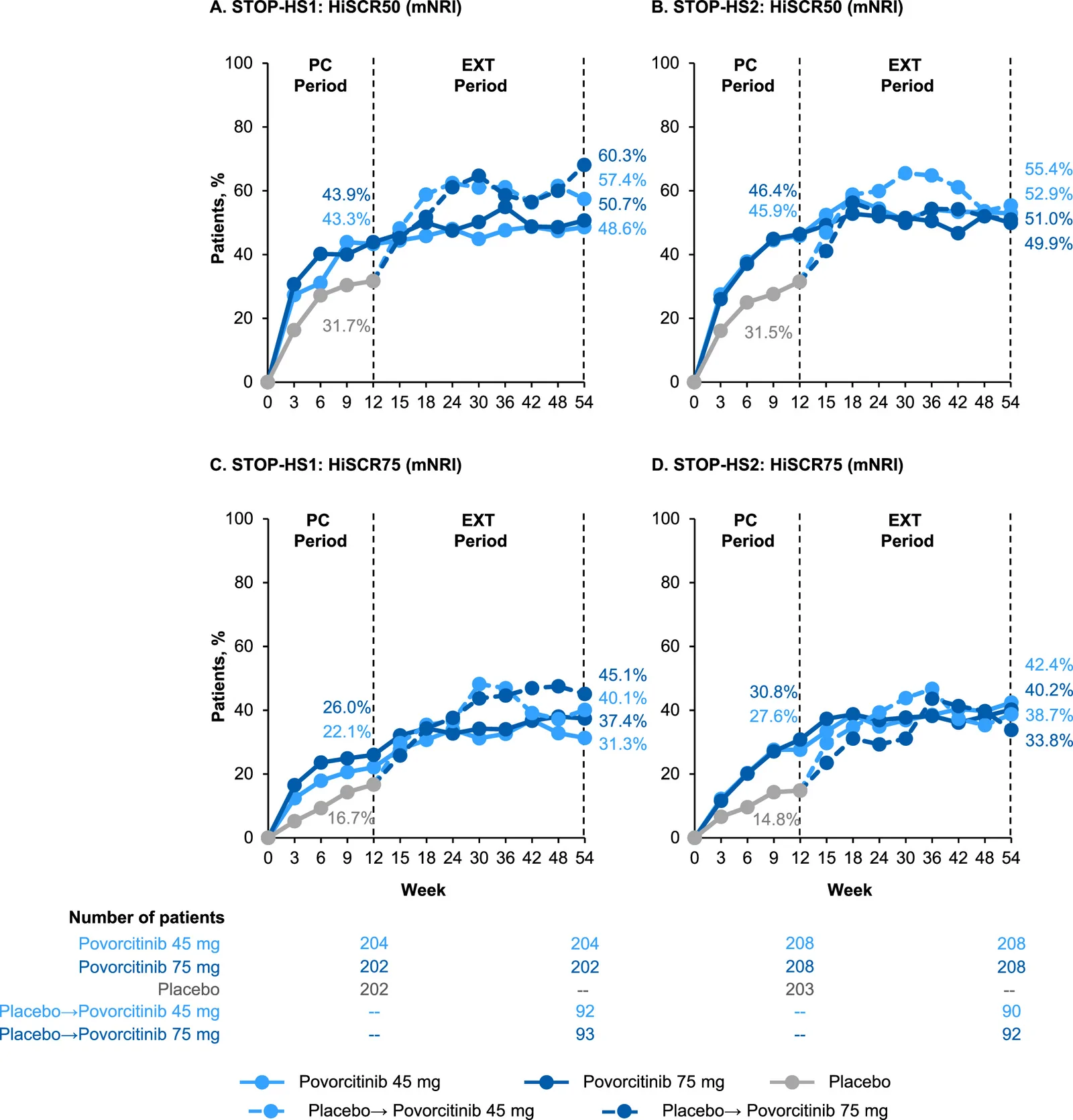
HiSCR50 and HiSCR75 Responses From Randomization Through Week 54 (mNRI). Panels show HiSCR50 (**a**,**b**) and HiSCR75 (**c**,**d**) responses from STOP-HS1 (**a**,**c**) and STOP-HS2 (**b**,**d**). Data were summarized descriptively and the estimation was combined using Rubin’s rule. EXT=extension; HiSCR=Hidradenitis Suppurativa Clinical Response; HiSCR50/75 = ≥ 50%/ ≥ 75% reduction from baseline in the total abscess and inflammatory nodule count, with no increase from baseline in abscess or draining tunnel count; HS=hidradenitis suppurativa; mNRI=modified nonresponder imputation; PC=placebo-controlled.

The primary endpoint of HiSCR50 at week 12 was met in both trials and for both doses of povorcitinib (Table 2). In STOP-HS1, 82 (40%) of 204 patients in the povorcitinib 45-mg group and 82 (41%) of 202 patients in the povorcitinib 75-mg group versus 60 (30%) of 202 patients in the placebo group met HiSCR50 (odds ratio (95% confidence interval) 45 mg: 1.6 (1.1−2.5), *P* = 0.0240; 75 mg: 1.6 (1.1−2.4), *P* = 0.0214). In STOP-HS2, 88 (42%) of 208 patients in the povorcitinib 45-mg group and 88 (42%) of 208 patients in the povorcitinib 75-mg group versus 58 (29%) of 203 patients in the placebo group met HiSCR50 (odds ratio (95% confidence interval) 45 mg: 1.8 (1.2−2.8), *P* = 0.0035; 75 mg: 1.9 (1.2−2.8); *P* = 0.0033). In both trials, response onset with povorcitinib treatment was rapid (by week 3, the first post-baseline visit; Fig. 2a,b) and was sustained through week 54, with a response rate of 57.3−71.4% (as observed; Fig. 3a,b), which was sustained even under a more stringent modified nonresponder imputation (mNRI) analysis (48.6−60.3%; Supplementary Table 2 and Extended Data Fig. 2). Additionally, maintenance of response (week 12 HiSCR50 responders who maintained or improved their response rate at each visit) during the extension period (pooled data; mNRI analyses) for povorcitinib 45 mg and 75 mg was, at week 24, 74.4% and 74.2%, respectively, with similar results at week 54 (66.3% and 69.3%) (Supplementary Table 3).

**Table 2:** Primary and key secondary efficacy outcomes in STOP-HS1 and STOP-HS2 placebo-controlled period

	STOP-HS1			STOP-HS2		
	Povorcitinib 45 mg (*n* = 204)	Povorcitinib 75 mg (*n* = 202)	Placebo (*n* = 202)	Povorcitinib 45 mg (*n* = 208)	Povorcitinib 75 mg (*n* = 208)	Placebo (*n* = 203)
**Primary efficacy endpoint**						
Evaluable, *n*	204	202	202	208	208	203
HiSCR50^a^ at week 12	82 (40%)	82 (41%)	60 (30%)	88 (42%)	88 (42%)	58 (29%)
Povorcitinib 45 mg versus placebo	1.6 (1.1−2.5); *P* = 0.0240^e^	—	—	1.8 (1.2−2.8); *P* = 0.0035^e^	—	—
Povorcitinib 75 mg versus placebo	—	1.6 (1.1−2.4); *P* = 0.0214^e^	—	—	1.9 (1.2−2.8); *P* = 0.0033^e^	—
**Key secondary endpoints**						
**HiSCR75 at week 12**						
Evaluable, *n*	204	202	202	208	208	203
HiSCR75^a^	42 (21%)	49 (24%)	32 (16%)	53 (25%)	59 (28%)	27 (13%)
Povorcitinib 45 mg versus placebo	1.4 (0.8−2.4); *P* = 0.1981^f^	—	—	2.2 (1.3−3.8); *P* = 0.0017^e^	—	—
Povorcitinib 75 mg versus placebo	—	1.7 (1.0−2.9); *P* = 0.0309^f^	—	—	2.6 (1.6−4.4); *P* = 0.0002^e^	—
**Skin pain response at week 12**						
Evaluable, *n*	157	158	149	165	159	151
≥3-point decrease in Skin Pain NRS^a,b,c^	27 (17%)	35 (22%)	17 (11%)	48 (29%)	35 (22%)	14 (9%)
Povorcitinib 45 mg versus placebo	1.6 (0.9−3.2); *P* = 0.1375^f^	—	—	4.1 (2.1−7.8); *P* < 0.0001^e^	—	—
Povorcitinib 75 mg versus placebo	—	2.1 (1.1−4.0); *P* = 0.0172^f^	—	—	2.9 (1.5−5.7); *P* = 0.0015^e^	—
Skin Pain NRS30^a,b,c^	50 (32%)	55 (35%)	33 (22%)	67 (41%)	60 (38%)	27 (18%)
Povorcitinib 45 mg versus placebo	1.7 (1.0−2.8); *P* = 0.0512^f^	—	—	3.1 (1.8−5.2); *P* < 0.0001^e^	—	—
Povorcitinib 75 mg versus placebo	—	1.9 (1.1−3.2); *P* = 0.0138^f^	—	—	2.7 (1.6−4.6); *P* = 0.0001^e^	—
**Flares through week 12**						
Evaluable, *n*	204	202	202	208	208	203
Flare^a,d^	51 (25%)	53 (26%)	68 (34%)	45 (22%)	43 (21%)	68 (33%)
Povorcitinib 45 mg versus placebo	0.7 (0.4−1.0); *P* = 0.0557^f^	—	—	0.5 (0.4−0.8); *P* = 0.0067^e^	—	—
Povorcitinib 75 mg versus placebo	—	0.7 (0.5−1.1); *P* = 0.1053^f^	—	—	0.5 (0.3−0.8); *P* = 0.0032^e^	—

Data are *n* (%) or odds ratio (95% confidence interval); *P* value, unless otherwise specified. Odds ratios are presented for binary variables.

^a^Patients with missing post-baseline values or who received other systemic treatment with potential effect on HS outcomes were imputed as nonresponders. Response rates and 95% confidence intervals for treatment comparisons were stratum adjusted using Mantel−Haenszel weights, and 95% confidence intervals for individual response rates were calculated using the Clopper−Pearson exact method. The Cochran−Mantel−Haenszel test was stratified by baseline abscess, inflammatory nodule and draining tunnel count (<11 versus ≥11) and prior treatment of HS with a biological drug (yes or no).

^b^Determined among patients with a baseline Skin Pain NRS score of 3 or higher.

^c^Decrease of 3 points or more in Skin Pain NRS score was evaluated for FDA-aligned countries; Skin Pain NRS30 was evaluated for EMA-aligned countries.

^d^Flare by week 12 was defined as at least one occurrence of flare between baseline and up to week 12, in which flare was defined as at least a 25% increase in the total abscess and inflammatory nodule count with an increase of at least two abscess and inflammatory nodules relative to baseline.

^e^Statistically significant per the statistical hierarchy.

^f^*P* value was calculated based on statistical testing methodology, had the given povorcitinib dose succeeded at hierarchical testing.

### Other efficacy endpoints

In both trials, povorcitinib demonstrated higher response rates than placebo across all key secondary endpoints; in STOP-HS2, statistical significance over placebo was met for all prespecified key secondary endpoints and for both doses of povorcitinib (Table 2).

In STOP-HS1, week 12 HiSCR75 was observed in 42 (21%) and 49 (24%) patients treated with povorcitinib 45 mg and 75 mg, respectively, versus 32 (16%) treated with placebo (not statistically significant). In STOP-HS2, week 12 HiSCR75 was met for both doses of povorcitinib: responders were 53 (25%; *P* = 0.0017) and 59 (28%; *P* = 0.0002) in the 45-mg and 75-mg groups, respectively, versus 27 (13%) treated with placebo. Similar to HiSCR50, response onset for HiSCR75 was rapid (Fig. 2c,d) and sustained during the study, with week 54 response rates of 39.0−57.0% (as observed; Fig. 3c,d) or 31.3−45.1% (mNRI; Supplementary Table 2 and Extended Data Fig. 2).

**Extended Data Fig. 3:**
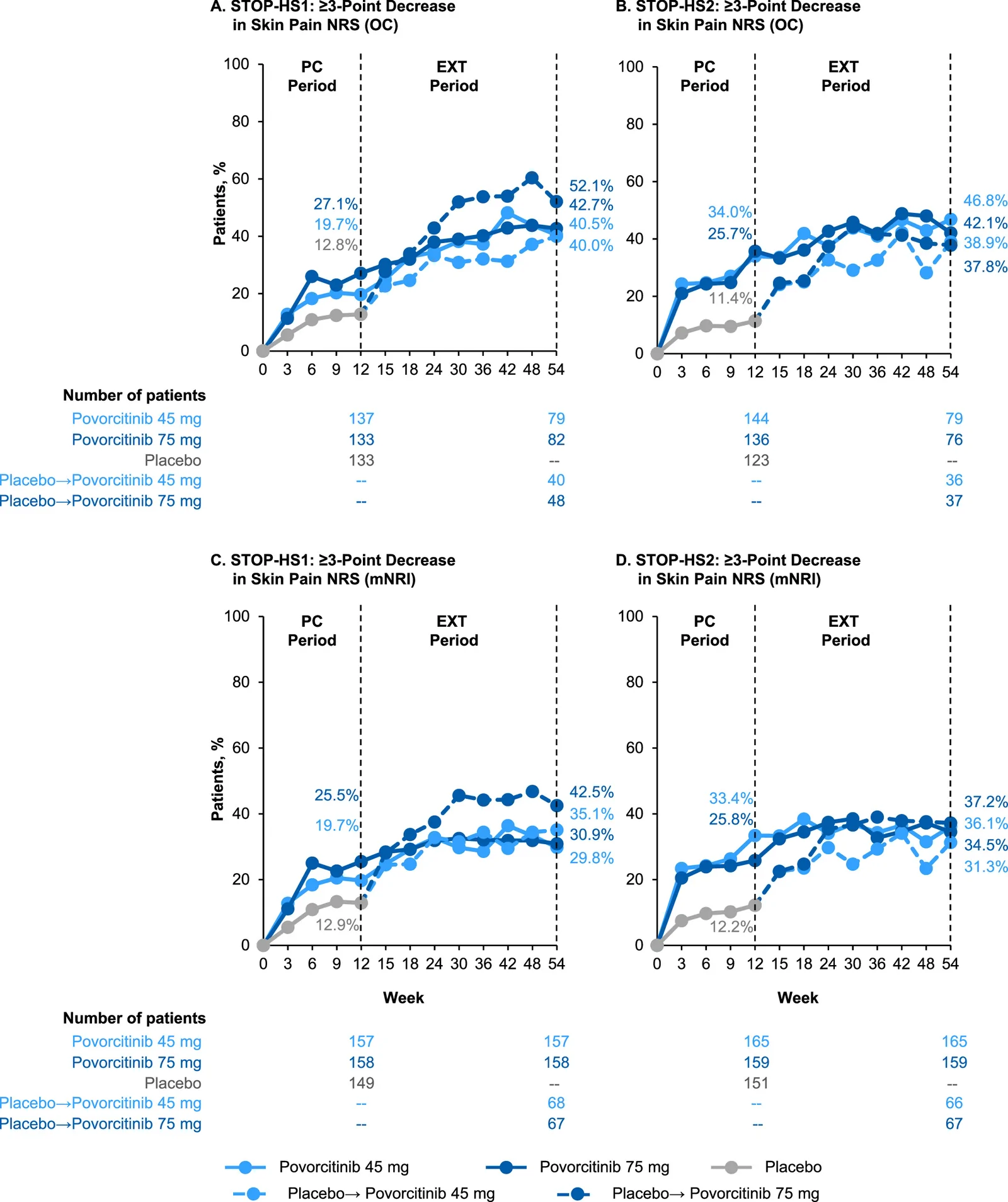
Skin Pain ≥3-Point Decrease Over Time Through Week 54 (Among Patients With Skin Pain NRS ≥3 at Baseline; OC and mNRI). Panels show Skin Pain NRS response from STOP-HS1 (**a**,**c**) and STOP-HS2 (**b**,**d**) using OC (**a**,**b**) and mNRI (**c**,**d**) methodology. Data were summarized descriptively and the estimation was combined using Rubin’s rule for mNRI. EXT=extension; HS=hidradenitis suppurativa; mNRI=modified nonresponder imputation; NRS=numerical rating scale; OC=observed cases; PC=placebo-controlled.

More patients treated with povorcitinib versus placebo had improvements in skin pain at week 12 in both studies, reaching statistical significance in STOP-HS2 (Table 2). Reductions in skin pain (≥3-point improvement and NRS30) were observed as early as week 3 and were sustained through week 54 (Supplementary Table 4, Extended Data Fig. 3 and Supplementary Fig. 1).

Flare control during the 12-week placebo-controlled period was statistically significant for both povorcitinib doses in STOP-HS2 (Table 2). Sustained flare control was observed with povorcitinib-randomized patients over 54 weeks of treatment (Extended Data Table 1).

**Extended Data Fig. 4:**
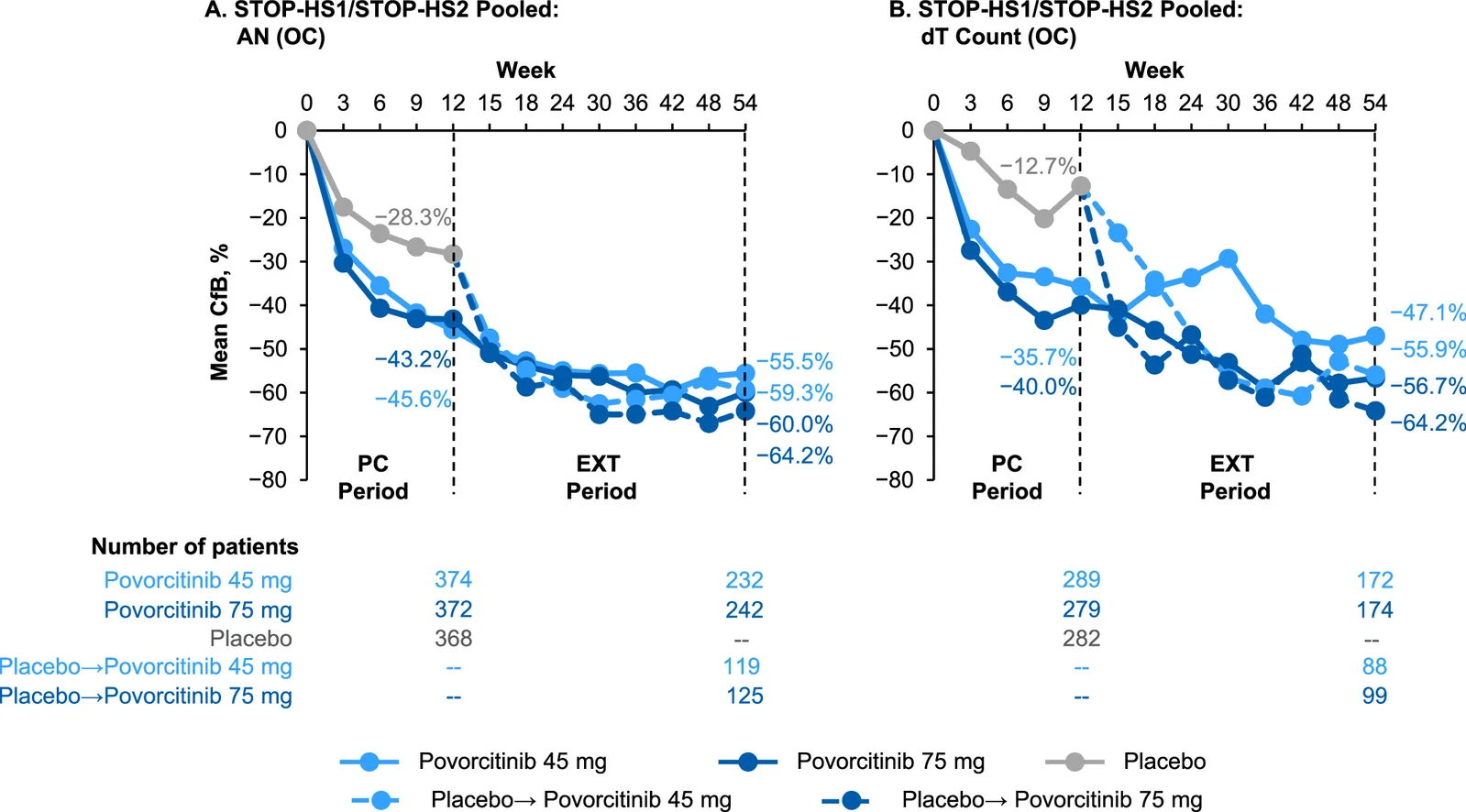
STOP-HS1 and STOP-HS2 Pooled Mean Percentage Change From Baseline Through Week 54 in (a) AN Count and (b) dT Count (OC). Data from STOP-HS1 and STOP-HS2 were pooled and summarized descriptively. AN=abscess and inflammatory nodule; CfB=change from baseline; dT=draining tunnel; EXT=extension; OC=observed cases; PC=placebo-controlled.

In both studies, reductions from baseline in abscess, inflammatory nodule and draining tunnel counts were observed by week 3 across povorcitinib doses, with lower counts in povorcitinib-treated patients than in those receiving placebo (Supplementary Table 5 and Extended Data Fig. 4). Continued reductions in lesion counts were observed during the extension period (Supplementary Tables 5 and 6 and Extended Data Fig. 4). At week 54, pooled STOP-HS1/STOP-HS2 mean percentage reductions from baseline in total abscess and inflammatory nodule and draining tunnel counts ranged from 55.5% to 64.2% and from 47.1% to 64.2%, respectively (Extended Data Fig. 4). Of relevance, patients treated with povorcitinib 75 mg generally experienced a greater improvement in draining tunnel counts than those in the 45-mg group (Supplementary Table 6 and Extended Data Fig. 4).

**Extended Data Fig. 5:**
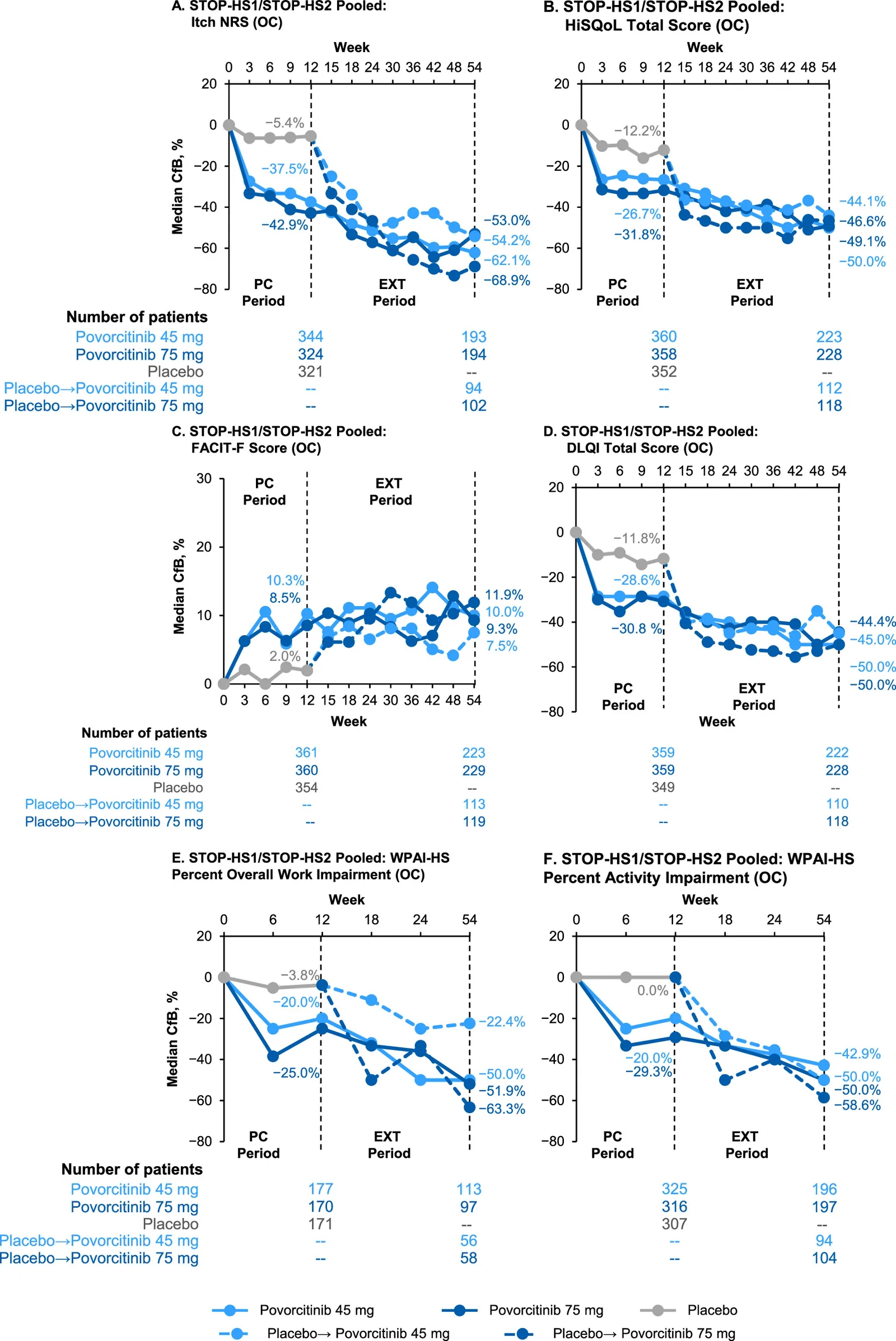
STOP-HS1 and STOP-HS2 Pooled Median Percentage Change From Baseline Through Week 54 in (a) Itch NRS, (b) HiSQoL, (c) FACIT-F,^a^ (d) DLQI, (e) WPAI-HS Work Impairment, and (f) WPAI-HS Activity Impairment (OC). Data from STOP-HS1 and STOP-HS2 were pooled and summarized descriptively. ^a^An increase in FACIT-F score (ie, increase in the median %CfB) indicates improvement in fatigue (ie, less severe fatigue). CfB=change from baseline; DLQI=Dermatology Life Quality Index; EXT=extension; FACIT-F=Functional Assessment of Chronic Illness Therapy–Fatigue; HiSQoL=Hidradenitis Suppurativa Quality of Life; NRS=numerical rating scale; OC=observed cases; PC=placebo-controlled; WPAI-HS=Work Productivity and Activity Impairment in Hidradenitis Suppurativa.

Improvements in other HS symptoms, besides skin pain, and in health-related QoL, assessed via Itch NRS, HiSQoL, DLQI, FACIT-F and Work Productivity and Activity Impairment–Hidradenitis Suppurativa (WPAI-HS), were greater with povorcitinib than with placebo and were observed as early as week 3, with sustained effect throughout the study (Extended Data Fig. 5).

### Safety outcomes

In both trials, povorcitinib was generally well tolerated at both doses. During the placebo-controlled period, the frequency of treatment-emergent adverse events (TEAEs) was generally similar between povorcitinib doses; serious AEs and AEs leading to discontinuation were infrequent and generally similiar across treatment groups (Table 3). The safety profile of povorcitinib remained consistent, with no new or unexpected safety events through week 54 (Table 3, Extended Data Table 2 and Supplementary Tables 7 and 8); during weeks 0−54, rates of serious AEs and TEAEs leading to discontinuation were low and occurred at similar rates between povorcitinib treatment groups. Across both trials and over 54 weeks, a single death of undetermined etiology was reported. The event occurred in the placebo to povorcitinib 45-mg crossover group (day 228 of povorcitinib treatment) in a patient with significant comorbidities, including morbid obesity (BMI of 67.2 kg m^−^^2^), type 2 diabetes, hypertension and a family history of sudden cardiac death. The event followed hospitalization for fall-related leg fractures, without routine thromboprophylaxis; the investigator determined that the death was unrelated to povorcitinib. Safety narratives for select events are presented in Supplementary Table 9. The most frequently reported TEAEs through week 54 were acne, nasopharyngitis, upper respiratory tract infection and headache.

**Table 3:** AEs to week 12 and week 54 in STOP-HS1 and STOP-HS2

Event	Placebo-controlled period (weeks 0−12)						Weeks 0–54	
	STOP-HS1			STOP-HS2			STOP-HS1/STOP-HS2 (pooled)	
	Povorcitinib 45 mg (*n* = 204) 100 PY = 0.46	Povorcitinib 75 mg (*n* = 202) 100 PY = 0.45	Placebo (*n* = 202) 100 PY = 0.46	Povorcitinib 45 mg (*n* = 208) 100 PY = 0.46	Povorcitinib 75 mg (*n* = 207) 100 PY = 0.47	Placebo (*n* = 203) 100 PY = 0.46	Povorcitinib 45 mg (*n* = 594)^a^ 100 PY = 4.48	Povorcitinib 75 mg (*n* = 594)^b^ 100 PY = 4.55
Any TEAE	122 (60%)	134 (66%)	106 (52%)	130 (63%)	131 (63%)	99 (49%)	459 (77%)	488 (82%)
Serious TEAE	3 (1%)	3 (1%)	6 (3%)	4 (2%)	4 (2%)	5 (2%)	30 (5%)	38 (6%)
Discontinuation due to TEAE	5 (2%)	7 (3%)	3 (1%)	12 (6%)	7 (3%)	4 (2%)	49 (8%)	46 (8%)
Treatment-related TEAE	43 (21%)	60 (30%)	33 (16%)	61 (29%)	79 (38%)	40 (20%)	226 (38%)	285 (48%)
Deaths	0	0	0	0	0	0	1 (<1%)	0
Most common TEAEs								
Acne	17 (8%)	30 (15%)	7 (3%)	18 (9%)	24 (12%)	11 (5%)	99 (17%)	119 (20%)
Headache	13 (6%)	16 (8%)	12 (6%)	14 (7%)	16 (8%)	7 (3%)	45 (8%)	50 (8%)
Nasopharyngitis	11 (5%)	14 (7%)	18 (9%)	10 (5%)	16 (8%)	9 (4%)	61 (10%)	72 (12%)
Upper respiratory tract infection	8 (4%)	11 (5%)	6 (3%)	12 (6%)	12 (6%)	6 (3%)	63 (11%)	61 (10%)
Select TEAEs								
Anemia^c^	4 (2%)	0	1 (<1%)	2 (1%)	1 (<1%)	1 (<1%)	23 (4%)	27 (5%)
Neutropenia	0	1 (<1%)	0	0	0	0	1 (<1%)	1 (<1%)
Lymphopenia	0	0	0	0	0	0	0	2 (<1%)
Thrombocytopenia	0	1 (<1%)	0	0	3 (1%)	0	0	7 (1%)
Hyperlipidemia^c^	6 (3%)	5 (2%)	0	7 (3%)	5 (2%)	11 (5%)	28 (5%)	24 (4%)
TEAEs of interest								
MACE^c^	0	0	0	0	0	0	1 (<1%)	0
Venous embolic/thrombotic events^c^	0	0	0	0	0	0	1 (<1%)	2 (<1%)
Serious infections^c^	1 (<1%)	2 (1%)	4 (2%)	0	0	2 (1%)	6 (1%)	12 (2%)
Cutaneous^c^	1 (<1%)	1 (<1%)	2 (1%)	0	0	1 (<1%)	4 (1%)	10 (2%)
Extracutaneous^c^	0	1 (<1%)	2 (1%)	0	0	1 (<1%)	2 (<1%)	2 (<1%)
Opportunistic infections^c^	0	0	0	1 (<1%)	0	0	5 (1%)	1 (<1%)
Tuberculosis^c^	0	0	0	0	0	0	0	0
Herpes zoster^c^	0	1 (<1%)	0	2 (1%)	2 (1%)	0	11 (2%)	12 (2%)
Malignancies^c^	0	0	0	0	0	2 (1%)	4 (1%)	1 (<1%)
NMSC^c^	0	0	0	0	0	1 (<1%)	4 (1%)	1 (<1%)
Excluding NMSC^c^	0	0	0	0	0	1 (<1%)	0	0
Gastrointestinal perforations^c^	0	0	0	0	0	0	0	0
HS exacerbations	0	1 (<1%)	1 (<1%)	2 (1%)	5 (2%)	4 (2%)	29 (5%)	30 (5%)
Renal dysfunction events^c^	1 (<1%)	3 (1%)	0	0	1 (<1%)	0	3 (1%)	3 (1%)
Hepatic events	3 (1%)	2 (1%)	3 (1%)	3 (1%)	2 (1%)	1 (<1%)	23 (4%)	31 (5%)
Increased CPK events^c^	7 (3%)	7 (3%)	2 (1%)	9 (4%)	7 (3%)	2 (1%)	30 (5%)	35 (6%)
Rhabdomyolysis	0	0	0	0	0	0	0	0
Myositis	0	0	0	0	0	0	0	0
Acne^c^	17 (8%)	31 (15%)	7 (3%)	18 (9%)	27 (13%)	12 (6%)	104 (18%)	132 (22%)

Data are *n* (%). AEs are presented as per MedDRA version 27.1. Data from STOP-HS1 and STOP-HS2 were pooled and summarized descriptively.

^a^Includes pooled STOP-HS1 and STOP-HS2 patients receiving povorcitinib 45 mg from day 1 and those who crossed over from placebo to 45 mg at week 12.

^b^Includes pooled STOP-HS1 and STOP-HS2 patients receiving povorcitinib 75 mg from day 1 and those who crossed over from placebo to 75 mg at week 12.

^c^Each term represents grouped TEAEs based on standard MedDRA query or custom preferred-term search.

NMSC, nonmelanoma skin cancer; PY, patient-year.

Acne was reported in 47 of 406 (12%) patients receiving povorcitinib in STOP-HS1 and in 42 of 415 (10%) patients in STOP-HS2 in the placebo-controlled period; over 54 weeks and across both studies, acne was reported in 99 of 594 (17%) patients receiving povorcitinib 45 mg and in 119 of 594 (20%) patients receiving 75 mg (Table 3 and Supplementary Table 10). All events of acne were grade 1 or 2, except one grade 3; discontinuations were rare (*n* = 7). During the placebo-controlled period, a total of five herpes zoster cases (0.6%), all grade 2, were reported; none led to discontinuation (Table 3). By week 54, 11 herpes zoster events occurred in the 45-mg group (all grade 2) and 12 in the 75-mg group (grade 1 or 2), with one discontinuation in each group. Most events of herpes zoster involved a single dermatome (83%) and were nonserious (100%). In STOP-HS1, 41.1% of patients had a history of varicella infection, 16.0% had received varicella vaccination, 4.1% had a history of dermatomal herpes zoster and 2.5% had received a herpes zoster vaccine; corresponding values in STOP-HS2 were 48.5%, 17.0%, 3.1% and 4.0%, respectively.

Increases in blood creatine phosphokinase (CPK) were observed infrequently through week 54 in either study; no cases of rhabdomyolysis or myositis were reported. Rates of TEAEs associated with laboratory parameters, such as anemia, neutropenia, lymphopenia, thrombocytopenia and hyperlipidemia, were 5% or lower during the overall 54 weeks of treatment, with no clear differentiation between the povorcitinib doses (Table 3, Extended Data Table 2 and Supplementary Table 11).

### Exploratory efficacy outcomes

**Extended Data Fig. 6:**
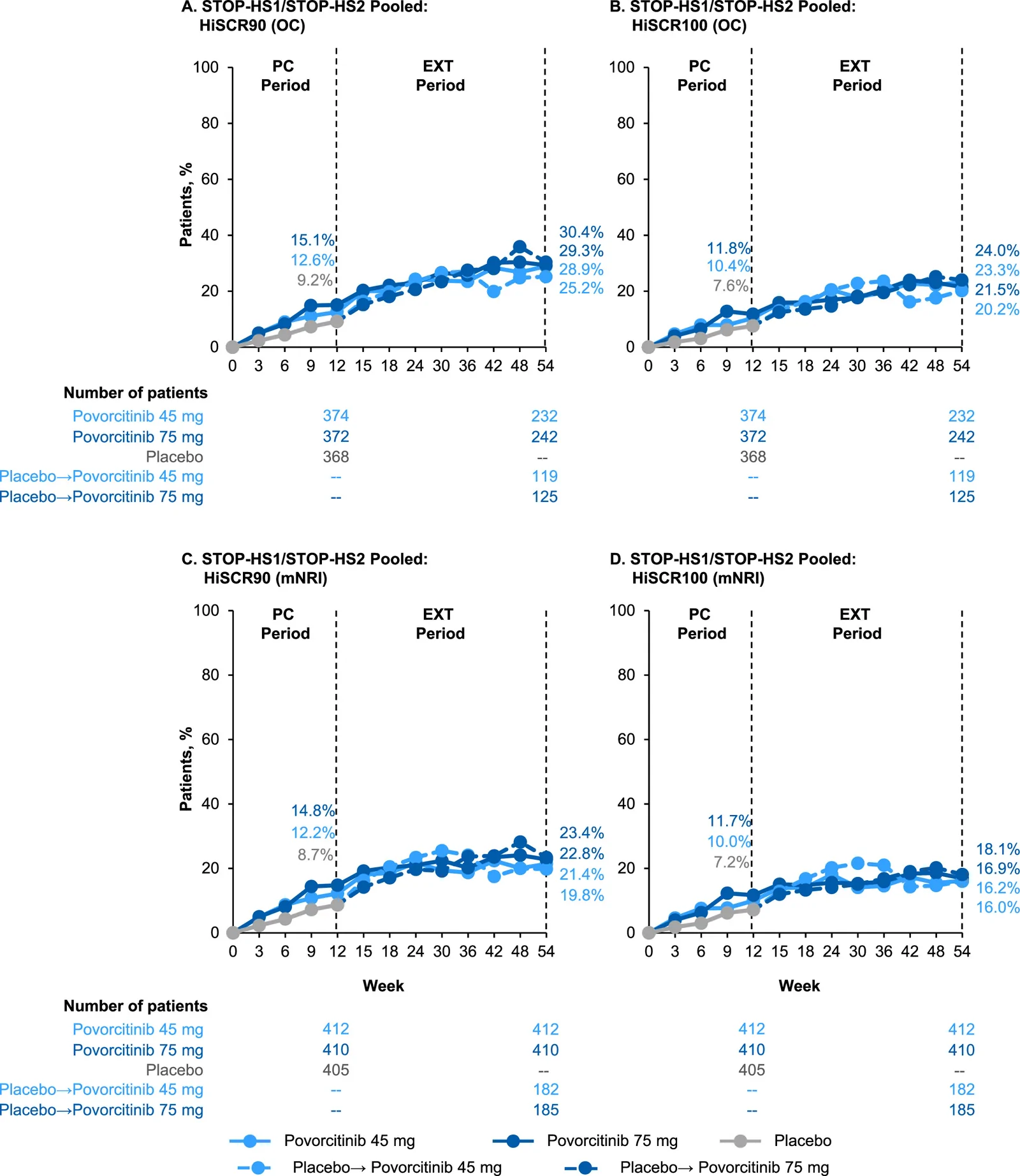
STOP-HS1 and STOP-HS2 Pooled HiSCR90 and HiSCR100 Responses From Randomization Through Week 54 (OC and mNRI). Panels show HiSCR90 (**a**,**c**) and HiSCR100 (**b**,**d**) responses using OC (**a**,**b**) and mNRI (**c**,**d**) methodology. Data from STOP-HS1 and STOP-HS2 were pooled and summarized descriptively, and the estimation was combined using Rubin’s rule for mNRI. EXT=extension; HiSCR=Hidradenitis Suppurativa Clinical Response; HiSCR90/100 = ≥ 90%/100% reduction from baseline in the total abscess and inflammatory nodule count, with no increase from baseline in abscess or draining tunnel count; HS=hidradenitis suppurativa; mNRI=modified nonresponder imputation; OC=observed cases; PC=placebo-controlled.

High-threshold HiSCR and International Hidradenitis Suppurativa Severity Score System (IHS4) endpoints were consistent with what was observed with HiSCR50/HiSCR75, with improvements seen early and sustained throughout the study. At week 54, HiSCR90 and HiSCR100 were observed in 23.2−36.2% and 17.9−29.0% of patients, respectively (Supplementary Table 2, Extended Data Fig. 6 and Supplementary Fig. 2).

Improvements in high-sensitivity C-reactive protein (hsCRP) were rapid and dose dependent, with marked reduction compared to placebo, and were maintained with povorcitinib treatment (Supplementary Fig. 3).

**Extended Data Fig. 7:**
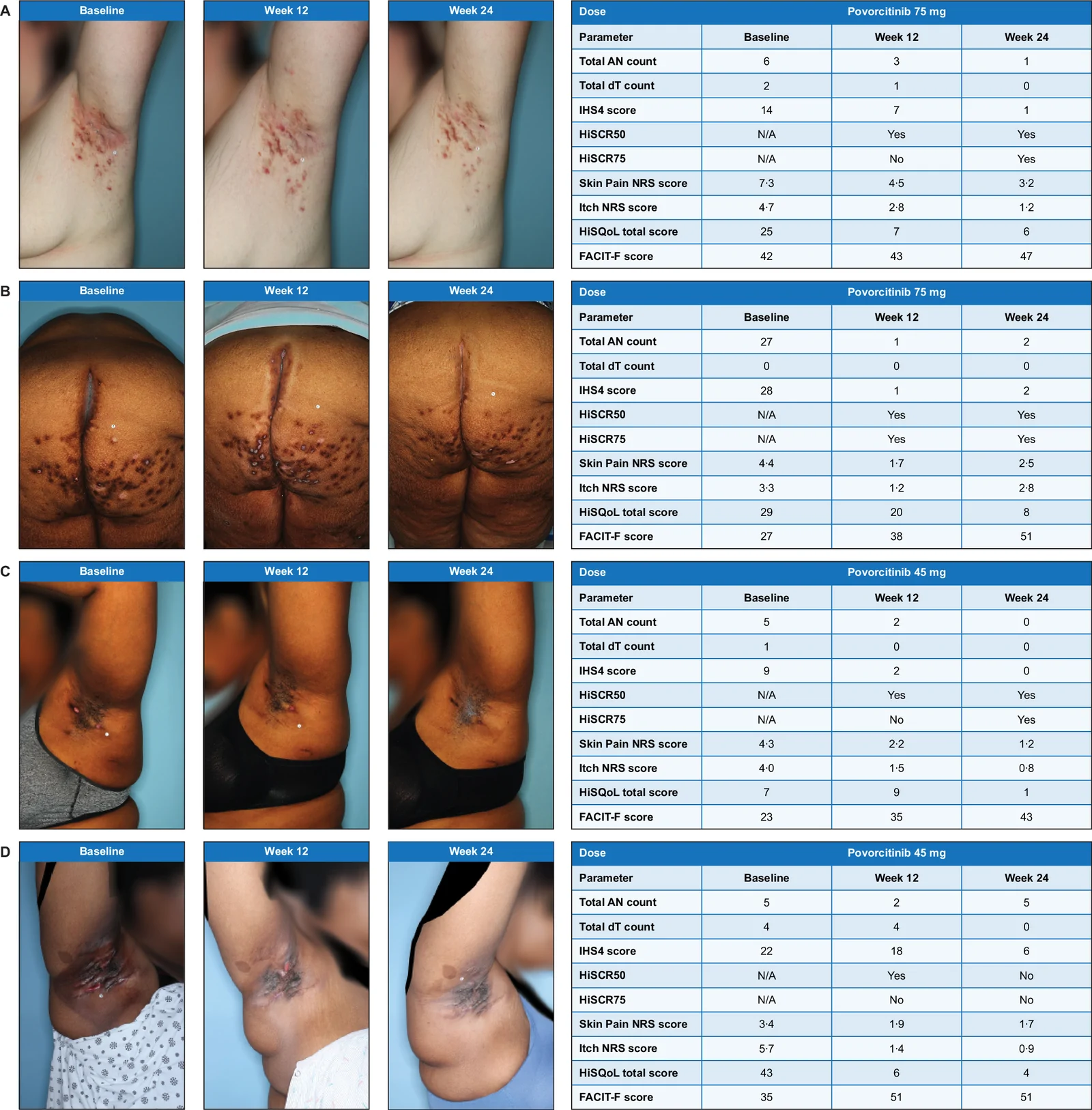
Representative Clinical Images Showing Reductions in HS Disease Burden With Povorcitinib From Baseline to Week 24. AN=abscess and inflammatory nodule; BMI=body mass index; dT=draining tunnel; FACIT-F=Functional Assessment of Chronic Illness Therapy–Fatigue; HiSCR50/75=at least 50%/75% reduction from baseline in the total abscess and inflammatory nodule count, with no increase from baseline in abscess or draining tunnel count; HiSQoL=Hidradenitis Suppurativa Quality of Life; HS=hidradenitis suppurativa; IHS4=International Hidradenitis Suppurativa Severity Score System; N/A=not applicable; NRS=numerical rating scale. (**a**) A 29-year-old White/Caucasian female, non-smoker, with Hurley Stage II HS, a disease duration of 13.4 years, and a BMI of 39.4 kg m^−2^, had no family history of HS. She had previously failed multiple biologics, including adalimumab and infliximab due to inadequate response (medication stopped working after a period of time), and secukinumab due to intolerance (adverse events). Over the 24-week study interval, there was a consistent decline in whole-body AN and dT counts. Clinical efficacy was evidenced by attainment of both HiSCR50 and HiSCR75, accompanied by a > 3-point reduction in skin pain and in itch, and a 76% improvement in HiSQoL score relative to baseline. Localized evaluation of the left axilla revealed marked resolution of inflammatory nodules, papules, and perilesional erythema, transitioning to residual post-inflammatory erythema and scarring. (**b**) A 67-year-old Black/African American female, former smoker, with Hurley Stage II HS, a disease duration of 20.9 years, and a BMI of 44.9 kg m^−2^, had a reported family history of HS. She had previously received systemic antibiotics, including clindamycin and doxycycline, both of which were discontinued due to inadequate response and adverse events. Over the 24-week study interval, the patient exhibited a rapid decline in whole-body AN counts, with no new dTs over this period, successfully achieving both HiSCR50 and HiSCR75. This clinical response was accompanied by a reduction in skin pain and itch, a 72% improvement in HiSQoL score from baseline, and a clinically meaningful improvement in fatigue (almost achieving a score of 52, which represents no fatigue). Targeted evaluation of the buttocks showed resolution of inflammation in the gluteal cleft, a typically recalcitrant area, along with scattered inflammatory nodules and papules on the buttocks. By week 24, near-complete resolution of active inflammation was observed, with residual scarring and reduced post-inflammatory hyperpigmentation compared with baseline. (**c**) A 38-year-old Black/African American female, non-smoker, with Hurley Stage II HS, a disease duration of 13.9 years, and a BMI of 42.3 kg m^−2^, had a reported family history of HS. She had previously received systemic therapy with doxycycline and adalimumab, both of which were discontinued due to inadequate response (lack of efficacy). Treatment resulted in achievement of HiSCR50 by week 12 and full clearance of inflammatory lesions by week 24, marked reductions in skin pain and itch scores, and an 86% improvement in HiSQoL score and a 20-point improvement in FACIT-F relative to baseline. Longitudinal photography of the left axilla documented involution of a dT. At baseline, the lesion presented as a domed, elongated tract between two inflammatory openings and showed decreased activity by week 12. By week 24, the dT was considered resolved, leaving only residual scarring and post-inflammatory hyperpigmentation, without evidence of active induration. (**d**) A 19-year-old Black/African American female, non-smoker, with Hurley Stage III HS, 6.8 years’ disease duration, and a BMI 41.6 kg m^−2^, had no family history of HS. She had previously completed systemic therapy with minocycline, which provided an inadequate response. Clinical assessment demonstrated achievement of HiSCR50 at week 12. At week 24, the AN count did not meet HiSCR50 criteria; however, there was total clearance of dTs. Patient-reported outcomes showed profound improvement, with a 91% decrease in HiSQoL score and a concurrent reduction in skin pain, itch, and fatigue (almost achieving a score of 52, which represents no fatigue). Targeted evaluation of the right axilla showed multiple inflamed longitudinal plaques consistent with dTs, as well as significant surrounding induration. The ulcerated tunnel orifices present at baseline had reduced in size and erythema by week 12 and subsequently transitioned into quiescent, linear fibrotic scars by week 24, with no evidence of active inflammation in the surrounding parenchyma by week 24.

Representative clinical images of patients achieving meaningful reduction in disease activity with povorcitinib treatment are shown in Extended Data Fig. 7.

## Discussion

Across both STOP-HS1 and STOP-HS2 phase 3 trials, povorcitinib demonstrated statistically significant improvements for both doses in the primary endpoint of HiSCR50 at week 12 versus placebo. Notably, the trials evaluated once-daily povorcitinib monotherapy in patients with moderate to severe HS and with baseline disease characteristics suggestive of a treatment-refractory population. The clinical responses were rapid and were sustained or further enhanced through week 54. Responses were meaningful, as supported by achievement of stringent, high-threshold outcomes, including HiSCR90 and HiSCR100, and by reductions in draining tunnels, a feature of advanced HS that remains particularly difficult to treat and is closely linked to long-term morbidity^22^. Beyond clinician-assessed outcomes, povorcitinib was associated with clinically meaningful improvements in patient-reported measures of skin pain, itch, fatigue and QoL (DLQI and HiSQoL). Of note, improvements in skin pain, the most common and disabling symptom of HS, were rapid and sustained during treatment with povorcitinib^23^.

Notably, too, clinical outcomes continued to improve after the 12-week placebo-controlled period. This pattern is consistent with other phase 3 HS studies and expected for a chronic inflammatory disease such as HS, which often requires treatment beyond the conventional 12- to 16-week primary endpoint to yield additional clinical benefit^12–14^. The reduction in overall lesion counts, symptom improvement and long-term flare control further suggest that povorcitinib may help stabilize disease activity and lessen the episodic burden of HS.

Currently approved treatments for HS include adalimumab (TNF inhibitor), secukinumab (IL-17A inhibitor) and bimekizumab (IL-17A/IL-17F inhibitor)^12–14^. Despite these options, the proportion of patients who are primary nonresponders or who have diminished responses over time is still considerable, likely owing to the heterogenous HS pathophysiology, in which biomarker validation is still an evolving process. Consequently, there is still a substantial unmet need for additional treatments involving other mechanisms of action^11,15^. By modulating JAK1 intracellular signaling downstream of multiple cytokines implicated in HS, such as IFNγ, TNF, IL-6 and IL-17 (refs. ^1,18,24^), povorcitinib provides a broader, pathway-level immunomodulatory effect compared to biologics that typically target single or dual cytokines^15^. This mechanistic breadth may underlie the rapid onset, depth and durability of clinical responses observed through week 54. Although no single biomarker has yet been adequately validated for routine clinical use in HS^25^, decreases in hsCRP (a nonspecific marker of inflammation) have been associated with JAK inhibition^26^ and were observed as early as week 3 (first post-baseline study visit).

Both regimens demonstrated robust efficacy; however, the 75-mg treatment group generally demonstrated greater reductions in draining tunnel counts as early as week 3 and sustained through the extension period. Given that HS is a heterogeneous condition, the evaluation of two distinct povorcitinib doses provides additional information to clinicians and is valuable in the context of therapeutic flexibility.

Povorcitinib was generally well tolerated at both doses. Acne, a frequent comorbidity of HS^27^ and a common AE associated with JAK inhibitor treatment^28^, was predominantly grade 1 or 2 and rarely led to discontinuation. Other safety observations, including asymptomatic elevations in CPK, were manageable and consistent with the known safety profile of JAK1 inhibitors^29,30^. Additionally, during the initial 12-week period, the incidence of anemia, neutropenia, lymphopenia, thrombocytopenia and hyperlipidemia remained low and similar to placebo across both dose groups; through 54 weeks, these events also remained uncommon. MACE were rare, with a single event reported in the extension period. Similarly, venous thromboembolic events were infrequent; in all cases, the affected patients had concurrent risk factors for these events, such as prolonged travel and family history of clotting. Serious infections, opportunistic infections and malignancies were also infrequent; no cases of tuberculosis were observed. The overall safety profile, including the low frequency of AEs associated with laboratory abnormalities, remained stable through week 54, supporting long-term use. The observed tolerability reflects the JAK1 selectivity of povorcitinib, which limits off-target inhibition of JAK2-mediated and JAK3-mediated pathways that are closely linked to hematopoiesis and maintenance of broader immune homeostasis^31^.

Limitations of the STOP-HS1 and STOP-HS2 studies include the relatively short 12-week placebo-controlled period, which may affect interpretation of longer-term efficacy outcomes. No formal statistical testing was performed to compare efficacy or safety outcomes between povorcitinib doses. In addition, heterogeneity in underlying disease severity and the multifactorial nature of HS may contribute to variability in treatment response^32^. Finally, the use of NRI, a conservative approach that classifies discontinuation for any reason, and administration of systemic antibiotic rescue for HS and prohibited therapies as nonresponse, may further underestimate treatment effects.

In conclusion, oral povorcitinib 45 mg and 75 mg were well tolerated in patients with moderate to severe HS and provided rapid, deep, sustained and clinically meaningful improvements in clinician-assessed and patient-reported outcome measures through 54 weeks. The safety profile was favorable, with no new or unexpected safety concerns identified, and broadly consistent across both doses. These data support the potential of oral povorcitinib as a treatment option for moderate to severe HS.

## Methods

### Study design and participants

STOP-HS1 and STOP-HS2 (ClinicalTrials.gov identifiers NCT05620823 and NCT05620836; date of registration: 10 November 2022 for both) were identically designed, global, multicenter, randomized, parallel-group, double-blind, placebo-controlled phase 3 trials of povorcitinib in HS conducted at 103 sites (STOP-HS1) and 99 sites (STOP-HS2) across Australia, Canada, Europe, Japan and the United States. The study protocol and all amendments (Supplementary Information) were reviewed and approved by the independent ethics committee or institutional review board at each study center. These trials were conducted in compliance with the Declaration of Helsinki, the study protocol, Good Clinical Practices and all applicable laws and regulations. All participants provided written informed consent before any study procedures were undertaken. Participation in these studies was voluntary and associated with monetary compensation.

Patients eligible for inclusion in either study were required to meet all of the following criteria:

1. Ability to comprehend and willingness to sign a written informed consent form for the study
2. Age ≥18 years at the time of signing the informed consent form
3. Diagnosis of moderate to severe HS for at least 3 months prior to the screening visit
4. Total abscess and inflammatory nodule count of at least five at both the screening and baseline visits
5. HS lesions in at least two distinct anatomic areas, one of which must be at least Hurley stage II or III, at both the screening and baseline visits
6. Documented history of inadequate response, intolerance or contraindication to at least a 3-month course of at least one conventional systemic therapy (oral antibiotic or biologic drug) for HS
7. Agreement not to use topical or systemic antibiotics for the treatment of HS during the placebo-controlled period, unless the criterion for systemic antibiotic rescue therapy was met
8. Agreement not to use diluted bleach baths or topical antiseptic washes containing chlorhexidine gluconate or benzoyl peroxide on areas affected by HS lesions during the placebo-controlled period. Over-the-counter soap and water were permitted
9. Agreement to use contraception, as follows:
  - Male and female patients of childbearing potential who agree to take appropriate precautions to avoid pregnancy from screening through 90 days after the last dose of study drug
  - Male and female patients who agree to refrain from donating sperm and oocytes, respectively, from screening through 90 days after the last dose of study drug
  - Permitted methods that are highly effective in preventing pregnancy should be communicated to the participants and their understanding confirmed
10. Willingness and ability to comply with the study protocol and procedures

Patients were not eligible for inclusion in either trial if they met any of the following criteria:

1. Presence of more than 20 draining tunnels (fistulas) at either the screening or baseline visit
2. Women who are pregnant (or who are considering pregnancy) or breastfeeding
3. Concurrent conditions or history of other diseases, as follows:

- Thrombocytopenia, coagulopathy or platelet dysfunction
- Venous and arterial thrombosis, deep vein thrombosis, pulmonary embolism, stroke, moderate to severe heart failure (New York Heart Association class III or IV), cerebrovascular accident, myocardial infarction, coronary stenting or coronary arterial revascularization with bypass graft surgery
- Diagnosis of other significant cardiovascular diseases, including, but not limited to, angina, peripheral arterial disease or uncontrolled arrhythmias such as atrial fibrillation, supraventricular tachycardia, ventricular tachycardia and forms of carditis
- Uncontrolled hypertension, as defined by a confirmed systolic blood pressure >160 mmHg or diastolic blood pressure >100 mmHg
- Patients who are permanently bedridden or wheelchair assisted
- Recipient of an organ transplant that requires continued immunosuppression
- Any malignancies or history of malignancies with the exception of adequately treated or excised nonmetastatic basal cell or squamous cell cancer of the skin or cervical carcinoma in situ
- Conditions that could interfere with drug absorption, including, but not limited, to short bowel syndrome
- Chronic or recurrent infectious disease, including, but not limited to, chronic renal infection, chronic chest infection (for example, bronchiectasis), recurrent urinary tract infection (recurrent pyelonephritis or chronic nonremitting cystitis), fungal infection, prior prosthetic joint infection at any time or open, draining or infected skin wounds or ulcers
- Current or history of disseminated herpes zoster or recurrent (more than one episode of) dermatomal herpes zoster
- Current or history of disseminated herpes simplex
- Active systemic infection or any active infection that, based on the investigatorʼs clinical assessment, makes the participant an unsuitable candidate for the study
- Any other active skin disease or condition (for example, bacterial, fungal or viral infection) that may interfere with the course, severity or assessments of HS
- Any clinically significant medical condition (other than HS) or any other reason that the investigator determines would interfere with the participantʼs ability to take part in the study, render them unsuitable for the study drug or place them at risk
- Any clinically significant medical condition other than HS, as determined by the investigator, that is not adequately controlled with appropriate treatment or may interfere with the course, severity or assessments of HS
- Albinism

4. A screening 12-lead electrocardiogram showing clinically significant abnormalities that require treatment (for example, acute myocardial infarction, serious tachyarrhythmias and bradyarrhythmias), findings indicative of serious underlying heart disease (for example, cardiomyopathy, major congenital heart disease, low voltage in all leads and Wolff−Parkinson−White syndrome) or evidence of abnormal Q wave interval (QT) or Fridericia-corrected Q wave interval (QTcF)
5. Patients who have undergone significant trauma or major surgery (per investigator’s assessment) within 30 days preceding the screening visit
6. Patients with a history of clinically significant (per investigator’s judgment) drug or alcohol abuse within 6 months preceding the screening visit
7. Patients with a history of treatment failure with any systemic or topical JAK inhibitor for HS or any other inflammatory condition
8. Patients who have received any of the following treatments within the minimum specified timeframes:
  - Use of any investigational or experimental treatments within 12 weeks or 5 half-lives (whichever is longer) prior to baseline visit
  - Use of immunomodulating biologic drugs within 12 weeks or 5 half-lives (whichever is longer) prior to baseline visit
  - Use of live vaccine within 8 weeks prior to baseline visit through 8 weeks after the last dose of study drug
  - Use of any topical or systemic JAK inhibitor within 4 weeks prior to baseline visit
  - Use of systemic immunosuppressive or immunomodulating small-molecule drugs (for example, oral or intravenous corticosteroids, avacopan, IRAK4 inhibitors, methotrexate, cyclosporine, dapsone and azathioprine) within 4 weeks prior to baseline visit
    Note 1: Use of corticosteroid inhalers and intranasal sprays was allowed.
    Note 2: Use of oral corticosteroids for nondermatologic conditions (for example, asthma exacerbation and bronchitis) was allowed for no longer than 7 days if deemed acceptable by the investigator and the sponsor (or designee).
  - Use of surgical, laser or any phototherapy intervention in areas with HS lesions within 4 weeks prior to baseline visit
  - Use of other systemic therapies for HS (for example, retinoids, antihypertensives, antihyperglycemics and antiandrogens such as acitretin, isotretinoin, metformin, spironolactone and finasteride) with potential therapeutic impact within 2 weeks prior to baseline visit
    Note that, at the discretion of the investigator (or designee), these drugs were permitted during the study if not prescribed for HS treatment, provided the dose was stable and not expected to change over the course of the study.
  - Use of systemic anti-infectives for HS treatment (for example, antibiotics, antivirals and antifungals) within 2 weeks prior to baseline visit
  - Use of any oral or topical phosphodiesterase-4 (PDE4) inhibitor (for example, apremilast and crisaborole); topical anti-infectives for HS lesions (for example, antibiotics, antivirals and antifungals); topical products containing chlorhexidine gluconate or benzoyl peroxide, diluted bleach baths and any other topical drug applied to HS lesions; or topical or intralesional corticosteroids within 1 week prior to baseline visit
    Note 1: Over-the-counter soap and water were not restricted.
    Note 2: Topical corticosteroids for dermatologic conditions other than HS (for example, atopic dermatitis and psoriasis) were allowed on areas not affected by HS, provided the extent of body surface area involvement did not interfere with participant safety or HS assessments.
  - Use of strong systemic cytochrome P450 3A4 (CYP3A4) inhibitors and strong and moderate systemic CYP3A4 inducers (for example, rifampicin/rifampin, ketoconazole, itraconazole, carbamazepine, ritonavir, St. John’s wort, grapefruit/grapefruit juice and Seville oranges) within 1 week or 5 half-lives (whichever is longer) prior to baseline visit
9. Patients with any of the following laboratory abnormalities at screening, defined as follows:
  - Platelets <100 × 10^9^ per liter
  - Hemoglobin <9 g dl^−1^
  - Absolute neutrophil count <1.5 × 10^9^ per liter
  - Total white blood cell count (leucocyte count) <3.0 × 10^9^ per liter
  - Absolute lymphocyte count <0.8 × 10^9^ per liter
  - Alanine aminotransferase >2× upper limit of normal (ULN)
  - Aspartate aminotransferase >2× ULN
  - Total bilirubin >1.5× ULN; participants with a clinical diagnosis of Gilbert syndrome were eligible provided the direct bilirubin was <ULN
  - Estimated glomerular filtration rate <45 ml min^−1^ 1.73 m^−^^2^, calculated using the simplified four-variable Modification of Diet in Renal Disease formula

10. Patients with *Mycobacterium tuberculosis* infection
11. Patients with active HIV or AIDS; active HIV is defined as confirmed positive anti-HIV antibody test
12. Patients with evidence of hepatitis B virus or hepatitis C virus infection or risk of viral reactivation
13. Patients with known hypersensitivity or severe reaction to povorcitinib or excipients of povorcitinib and/or other products in the same class
14. Patients with any condition, laboratory abnormality or screening assessment result that, in the judgment of the investigator and sponsor (or designee), could interfere with full participation in the study (including administration of the study drug or attendance at required study visits), pose a significant risk to the participant or compromise the interpretation of study data
15. France-specific exclusion: Vulnerable populations as defined in Article L.1121-6 of the French Public Health Code, as well as adults under legal protection or those unable to provide consent in accordance with Article L.1121-8 of the French Public Health Code, were excluded

Patients were prohibited from receiving concomitant systemic antibiotics for HS during the placebo-controlled period, except for protocol-defined rescue. Concomitant use of analgesics (non-opioid and opioid) was permitted during the study (full protocols are provided in the Supplementary Information).

Sex was recorded as a biological variable based on clinical records at screening; included options were female and male. Demographic information about the number of females and males is provided in Table 1 and Supplementary Table 1; no further analyses were conducted. The protocols for both studies were updated with two amendments each in response to regulatory requests from European Union national health authorities. These amendments provided clarity on the studies and patient management but did not alter study endpoints, patient eligibility or study conduct in general. Specific details on the amendments are available in the Supplementary Information.

### Randomization and masking

An interactive response technology system was used to assign patient identification numbers, track visits, randomize patients per prespecified parameters and mask treatment group assignments. Patients were stratified by the total abscess, inflammatory nodule and draining tunnel count (<11 or ≥11) and previous biologic use for HS (yes or no) and were randomly assigned 2:2:1:1 to once-daily povorcitinib 45 mg, povorcitinib 75 mg, placebo with crossover to povorcitinib 45 mg at week 12 or placebo with crossover to povorcitinib 75 mg at week 12; the overall treatment period was 54 weeks. Patients and investigators remained blinded to treatment assignments during the placebo-controlled and extension periods of the study. The study sponsor remained blinded through completion of the placebo-controlled period and was unblinded after primary endpoint database lock.

### Procedures

The study consisted of two treatment periods (placebo-controlled: 12 weeks; extension: 42 weeks), both double-blinded (Supplementary Information and Extended Data Fig. 1). Patients randomized to povorcitinib were treated with the same dose (once-daily 45 mg or 75 mg) through week 54; patients initially randomized to placebo started, after week 12, treatment with once-daily povorcitinib (45 mg or 75 mg, as previously described) and continued it through week 54.

Lesion count assessments and patient-reported outcomes, including HiSQoL (a 17-item HS-specific health-related QoL instrument that assesses HS symptoms and the impact of HS on QoL), DLQI (a 10-item questionnaire that assesses general skin-related QoL) and FACIT-F (a 13-item questionnaire that assesses fatigue), were conducted at baseline and at weeks 3, 6, 9, 12, 15, 18, 24, 30, 36, 42, 48 and 54 (or end of treatment due to early termination). WPAI-HS was assessed at baseline and at weeks 6, 12, 18, 24 and 54 (or end of treatment due to early termination). Skin Pain NRS and Itch NRS were used to electronically collect daily data from screening through week 54 using a 24-hour recall period. The NRS instruments were based on an 11-point NRS, from 0 (no skin pain; no itch) to 10 (skin pain as bad as you can imagine; worst itch imaginable). Safety was assessed at each study visit. Safety follow-up visits were scheduled 30 days after the last dose of study drug for patients who did not enter the subsequent STOP-HS long-term extension study (NCT06212999) or who prematurely discontinued treatment. Rescue treatments for HS—including systemic antibiotics, intralesional corticosteroid injections and incision and drainage procedures—were allowed based on protocol-defined criteria. For a subset of consenting patients, photographs of the affected areas were taken at baseline and on weeks 12 and 24; patients also consented to use of these photographs in publications. Further details on study design are described in the protocols (Supplementary Information).

### Outcomes

The primary endpoint in each trial was HiSCR50 at week 12, defined as a reduction of at least 50% from baseline in total abscess and inflammatory nodule count, with no increase from baseline in abscess or draining tunnel count. Key secondary endpoints were HiSCR75 (reduction of ≥75% from baseline in total abscess and inflammatory nodule count, with no increase from baseline in abscess or draining tunnel count) at week 12; skin pain response (among patients with baseline Skin Pain NRS score of at least 3) at week 12, assessed either as the percentage of patients who achieved a reduction of at least 3 points from baseline (for US Food and Drug Administration (FDA) and aligned countries) or as Skin Pain NRS30 (defined as a reduction of ≥30% and at least 1 unit from baseline (for European Medicines Agency (EMA) and aligned countries)); and flare occurrence (defined as a ≥25% and ≥2-unit increase in abscess and inflammatory nodule count relative to baseline) by week 12.

Data are reported for all prespecified secondary endpoints, including abscess, inflammatory nodule and draining tunnel counts at each visit; the percentage of patients with baseline FACIT-F score no more than 48 achieving at least a 4-point increase (improvement) at week 12; and the percentage achieving HiSCR50 and HiSCR75 at week 12 and maintaining it at each visit during the extension period. At weeks 24 and 54, the percentages of patients achieving HiSCR50, HiSCR75 or skin pain response or experiencing a flare were assessed. Additionally, change from baseline in DLQI and change/percentage change from baseline in lesion counts (abscess, inflammatory nodule and draining tunnel) were evaluated at each visit through week 54.

Additional prespecified exploratory outcomes evaluated the long-term efficacy of povorcitinib measured by HiSCR50, HiSCR75, HiSCR90 and HiSCR100 (reduction of ≥50%, ≥75%, ≥90% or 100% from baseline in total abscess and inflammatory nodule count, with no increase from baseline in abscess or draining tunnel count). Full list of exploratory outcomes is presented in the Supplementary Information.

Safety and tolerability were assessed by monitoring TEAEs and laboratory parameters through week 54. Events were graded according to Common Terminology Criteria for Adverse Events and coded using the Medical Dictionary for Regulatory Activities (MedDRA) version 27.1. TEAEs were reported for all treatment groups from weeks 0 to 12; in addition, a pooled analysis of both studies evaluated TEAEs from baseline to week 54 in patients receiving at least one dose of povorcitinib 45 mg or 75 mg. Prespecified AEs of interest included infection (serious, opportunistic, herpes zoster and tuberculosis), malignancy, MACE, other thromboembolic events, renal dysfunction, hepatic events, increased CPK and acne. An external independent data monitoring committee periodically assessed safety data and provided recommendations on study conduct and safety analyses; in addition, potential cardiovascular or thromboembolic TEAEs were adjudicated by an external independent committee.

### Statistical analysis

Statistical analyses for both trials were governed by a shared statistical analysis plan. The primary objective was to evaluate the efficacy of each dose of povorcitinib compared to placebo in patients with moderate to severe HS by assessing HiSCR50 response at week 12. Based on HiSCR50 response rates observed in a phase 2, randomized, dose-ranging study of povorcitinib, the assumed HiSCR50 response rates at week 12 were 45% for povorcitinib 45 mg, 48% for povorcitinib 75 mg and 30% for placebo. Under these assumptions, a sample size of 200 participants per group (600 total) provided 93% power to demonstrate statistical superiority of povorcitinib 75 mg over placebo and 81% power to demonstrate superiority of povorcitinib 45 mg over placebo, at a two-sided significance level of 0.025 using a *χ*^2^ test without continuity correction. Both trials were independently powered to test primary and key secondary outcomes.

All randomized patients were included in the intent-to-treat (ITT) population used for the summary of demographics, baseline characteristics, patient disposition and efficacy analyses during the placebo-controlled period. The safety population included all patients who received at least one dose of povorcitinib or placebo during the placebo-controlled period. Superiority of each povorcitinib dose (45 mg and 75 mg) versus placebo in the primary analysis was assessed separately using a Cochran−Mantel−Haenszel test stratified by randomization factors at a two-sided *α* = 0.025. Stratum-adjusted HiSCR50 rate differences with 95% confidence intervals were estimated using Mantel−Haenszel weights, and common odds ratios with 95% confidence intervals were reported. Participants who discontinued treatment for any reason, or received systemic antibiotics for HS or prohibited medications, were considered nonresponders for all subsequent visits (NRI); those with other missing post-baseline data were imputed as nonresponders at that visit only. Key secondary endpoints (HiSCR75, skin pain response and flare) were analyzed similarly in the ITT population using the stratified Cochran−Mantel−Haenszel test and NRI. The overall familywise type I error was controlled at a two-sided *α* level of 0.05. A graphical approach was applied to the primary and key secondary endpoints to adjust for multiplicity (Supplementary Fig. 4). All other secondary and exploratory endpoints were analyzed in the ITT population in the placebo-controlled period without control for type I error. Categorical endpoints were analyzed using stratified Cochran−Mantel−Haenszel tests with NRI. Continuous endpoints were assessed using analysis of covariance, with treatment group, randomization stratification factors and baseline measurement included as covariates. Long-term efficacy across the placebo-controlled and extension periods was summarized descriptively in the extension-evaluable population. Post hoc mNRI analyses were conducted for some endpoints. In short, patients with treatment discontinuation due to lack of efficacy or an AE were considered nonresponders for all subsequent visits on or after study treatment discontinuation. Patients who started a systemic antibiotic for HS had last observation prior to rescue treatment initiation carried forward to all subsequent visits from the rescue treatment start date onward; all other data were imputed based on multiple imputation. Some endpoints were presented as observed cases. In these analyses, missing data were not imputed. Only participants with available data who had not discontinued study treatment at the given timepoint were included. Participants with missing data or who prematurely discontinued study treatment were treated as missing. Data from select non-key secondary and exploratory endpoints, as well as safety data, were pooled to facilitate evaluation of the results. When applicable, given the identical study designs and overall similarity across the demographics and disease characteristics of the study populations, data from both studies were pooled and summarized descriptively (no statistical model was used for data pooling); data pooling was not prespecified in the protocol or the statistical analysis plan and was performed in post hoc analyses. All analyses were done with SAS software version 9.4 (SAS Institute; additional details are in the Supplementary Information). Both STOP-HS1 (NCT05620823) and STOP-HS2 (NCT05620836) trials are registered with ClinicalTrials.gov and are completed.

### Reporting summary

Further information on research design is available in the Nature Portfolio Reporting Summary linked to this article.

## Online content

Any methods, additional references, Nature Portfolio reporting summaries, source data, extended data, supplementary information, acknowledgements, peer review information; details of author contributions and competing interests; and statements of data and code availability are available at https://doi.org/10.1038/s41591-026-04534-z.

## Supplementary information

Supplementary Information Figs. 1−4, Tables 1−11, STOP-HS1 protocol, STOP-HS2 protocol and combined STOP-HS1/STOP-HS2 statistical analysis plan.

Reporting Summary

## Data Availability

Incyte Corporation is committed to data sharing that advances science and medicine while protecting patient privacy. Qualified external scientific researchers may request anonymized datasets owned by Incyte for the purpose of conducting legitimate scientific research. Researchers may request anonymized datasets from any interventional study (except phase 1 studies) for which the product and indication have been approved on or after 1 January 2020 in at least one major market (for example, United States, European Union and Japan). Data will be available for request after the primary publication or 2 years after the study has ended. Information on Incyte’s clinical trial data-sharing policy and instructions for submitting clinical trial data requests are available at https://www.incyte.com/Portals/0/Assets/Compliance%20and%20Transparency/clinical-trial-data-sharing.pdf?ver=2020-05-21-132838-960. The redacted study protocol is available in the Supplementary Information.

## Extended data

**Extended Data Table 1:** STOP-HS1 and STOP-HS2 Pooled Percentage of Patients With ≥1 Flare for Select Study Intervals

Time frame, patients with ≥1 flare, % (n/N)	Povorcitinib 45 mg	Povorcitinib 75 mg	Placebo→45 mg	Placebo→75 mg
Baseline through Week 12	23.3 (96/412)	23.4 (96/410)	33.6 (136/405)	
Baseline through Week 24	19.8 (80/404)	20.8 (84/404)	—	—
Baseline through Week 54	24.3 (98/404)	24.3 (98/404)	—	—
Crossover (Week 12) through Week 24	—	—	9.9 (18/181)	7.0 (13/185)
Crossover (Week 12) through Week 54	—	—	18.2 (33/181)	11.4 (21/185)

The ‘baseline through Week 12’ interval utilised intercurrent events to account for flares; consequently, patients who discontinued for any reason or who started systemic antibiotic rescue therapy or prohibited medication with potential effect on HS are counted as having a flare. The ‘baseline through Week 24’ and ‘baseline through Week 54’ intervals counted as having a flare only those patients who met the protocol-defined criteria (≥25% and ≥2-unit increase in abscess and inflammatory nodule count relative to baseline) considering the specified treatment period (Day 1 through Week 24, or Day 1 through Week 54, respectively). For placebo crossovers, the evaluated study period for presence of a flare was determined from the first dose of povorcitinib (ie, after Week 12) onward. Data from STOP-HS1 and STOP-HS2 were pooled and summarized descriptively. HS=hidradenitis suppurativa.

**Extended Data Table 2:** STOP-HS1 and STOP-HS2 Pooled Incidence and Exposure-Adjusted Rates of TEAEs (≥5%), AESIs, and ECIs During the Placebo-Controlled Period by Treatment Regimen

Event, n (%) [EAIR per 100 PY]	Povorcitinib 45 mg (n=412) PY=92.14	Povorcitinib 75 mg (n=409) PY=91.92	Placebo (n=405) PY=91.33
TEAEs occurring in ≥5% in any treatment group			
Acne	35 (8%) [38.0]	54 (13%) [58.7]	18 (4%) [19.7]
Headache	27 (7%) [29.3]	32 (8%) [34.8]	19 (5%) [20.8]
Nasopharyngitis	21 (5%) [22.8]	30 (7%) [32.6]	27 (7%) [29.6]
Upper respiratory tract infection	20 (5%) [21.7]	23 (6%) [25.0]	12 (3%) [13.1]
TEAEs of special interest			
MACE^a^	0	0	0
Venous embolic/ thrombotic events^a^	0	0	0
Serious infections^a^	1 (<1%) [1.1]	2 (<1%) [2.2]	6 (1%) [6.6]
Opportunistic infections^a^	1 (<1%) [1.1]	0	0
Tuberculosis^a^	0	0	0
Herpes zoster^a^	2 (<1%) [2.2]	3 (1%) [3.3]	0
Malignancies^a^	0	0	2 (<1%) [2.2]
NMSC^a^	0	0	1 (<1%) [1.1]
Excluding NMSC^a^	0	0	1 (<1%) [1.1]
Gastrointestinal perforations^a^	0	0	0
HS exacerbations	2 (<1%) [2.2]	6 (1%) [6.5]	5 (1%) [5.5]
TEAEs of clinical interest			
Renal dysfunction events^a^	1 (<1%) [1.1]	4 (1%) [4.4]	0
Hepatic events^a^	6 (1%) [6.5]	4 (1%) [4.4]	4 (1%) [4.4]
Increased CPK^a^	16 (4%) [17.4]	14 (3%) [15.2]	4 (1%) [4.4]
Acne^a^	35 (8%) [38.0]	58 (14%) [63.1]	19 (5%) [20.8]

Data from STOP-HS1 and STOP-HS2 were pooled and summarized descriptively.

^a^ Each term represents grouped TEAEs based on Standard MedDRA Query or custom preferred-term search. AESI=adverse events of special interest; CPK=creatine phosphokinase; EAIR=exposure-adjusted incidence rates; ECI=events of clinical interest; HS=hidradenitis suppurativa; MACE=major adverse cardiovascular event; NMSC=nonmelanoma skin cancer; PC=placebo-controlled; PY=patient-year; TEAE=treatment-emergent adverse event.
