# Author Correction: Povorcitinib for hidradenitis suppurativa: the randomized, double-blind, placebo-controlled STOP-HS1 and STOP-HS2 phase 3 trials

**DOI:** 10.1038/s41591-026-04747-2

**Published:** 2026-10-21

**Authors:** Martina L. Porter, Antonio Martorell, Christopher J. Sayed, Falk G. Bechara, Hadar Lev-Tov, Jennifer L. Hsiao, John W. Frew, Ziad Reguiaï, Melinda J. Gooderham, Noah Goldfarb, Hessel H. van der Zee, Veronique del Marmol, Angelo V. Marzano, Benjamin D. Ehst, Lindsay S. Ackerman, Koremasa Hayama, Chenwei Tian, Jennifer Kelley, Huiling Zhen, Joslyn S. Kirby, Kurt Brown, Leandro L. Santos, Christos C. Zouboulis

**Affiliations:** 1 Department of Dermatology, Harvard Medical School, Boston, MA USA; 2 Beth Israel Deaconess Medical Center, Boston, MA USA; 3 Inflammatory Diseases Unit, Department of Dermatology, Venereology and Surgery, Hospital de Manises, Valencia, Spain; 4 Department of Dermatology, University of North Carolina School of Medicine, Chapel Hill, NC USA; 5 ICH – International Center for Hidradenitis Suppurativa/Acne Inversa, Department of Dermatology, Venereology and Allergology, Ruhr-University Bochum, Bochum, Germany; 6 Dr. Philip Frost Department of Dermatology and Cutaneous Surgery, University of Miami, Miami, FL USA; 7 Department of Dermatology, University of Southern California, Los Angeles, CA USA; 8 Laboratory of Translational Cutaneous Medicine, Ingham Institute for Applied Medical Research, Liverpool, New South Wales Australia; 9 Department of Dermatology, Liverpool Hospital, Liverpool, New South Wales Australia; 10 School of Clinical Medicine, University of New South Wales Sydney, Kensington, New South Wales Australia; 11 Department of Dermatology, Polyclinique Courlancy-Bezannes, Reims-Bezannes, France; 12 Probity Medical Research, Waterloo, Ontario Canada; 13 SKiN Centre for Dermatology, Peterborough, Ontario Canada; 14 Queen’s University, Kingston, Ontario Canada; 15 Departments of Dermatology and Medicine, University of Minnesota, Minneapolis, MN USA; 16 Department of Dermatology, University of Minnesota Physicians, Minneapolis, MN USA; 17 Department of Dermatology, Erasmus Medical Center, Rotterdam, Netherlands; 18 Department of Dermatology, Hôpital Erasme, Université Libre de Bruxelles, Brussels, Belgium; 19 Dermatology Unit, Fondazione IRCCS Ca’ Granda Ospedale Maggiore Policlinico, Milan, Italy; 20 Department of Pathophysiology and Transplantation, Università degli Studi di Milano, Milan, Italy; 21 Oregon Medical Research Center, Portland, OR USA; 22 USA Medical Dermatology Specialists, US Dermatology Partners, Phoenix, AZ USA; 23 Division of Cutaneous Science, Department of Dermatology, Nihon University School of Medicine, Itabashi City, Tokyo, Japan; 24 Incyte Corporation, Wilmington, DE USA; 25 Department of Dermatology, Venereology, and Immunology, Universitaetsklinikum Ruppin-Brandenburg, Brandenburg Medical School Theodor Fontane and Faculty of Health Sciences Brandenburg, Neuruppin, Germany

**Keywords:** Phase III trials, Inflammatory diseases

Correction to: *Nature Medicine* https://doi.org/10.1038/s41591-026-04534-z, published online 23 July 2026.

In the version of the article initially published, in the second paragraph of the “Patients” section of the Results, “Baseline disease characteristics were indicative of recalcitrant, longstanding HS (mean (range) disease duration, 9.7 (0.4−49.1)−10.8 (0.3−48.7) years)” should have read “Baseline disease characteristics were indicative of recalcitrant, longstanding HS (mean (range) disease duration, 9.7 (0.4−49.1)−10.8 (0.4−57.7) years)”. In the Fig. 1 legend, in footnote ^b^, “In STOP-HS2, two patients discontinued after completing treatment through week 12: one discontinued due to physician decision, and one withdrew” should have read “In STOP-HS2, three patients discontinued after completing treatment through week 12: two discontinued due to physician decision, and one withdrew”; and footnote ^d^ was “Two patients discontinued after completing treatment through week 12; both withdrew” but should have been “One patient withdrew after completing treatment through week 12”. In Table 2, the superscript for the *P* values in STOP-HS1 for the 75 mg dose in the rows for ≥3-point decrease in Skin Pain NRS and Skin Pain NRS30 should be “^f^” rather than “^e^”. In Extended Data Fig. 5e, f, the grey dotted line between weeks 18 and 24 should have been blue and the values next to the week 6 dots should have been next to the week 12 dots. Additionally, in Supplementary Table 6A, the “Week 12” row of the “A75, Baseline A count, 3−5” section originally read “37.2 (16/43), 36.8 (25/68), 35.3 (18/51)” but should have read “36.8 (25/68), 35.3 (18/51), 37.2 (16/43)”. These corrections have been made to the HTML and PDF versions of the article.

