# Supplementary material for: Povorcitinib for hidradenitis suppurativa: the randomized, double-blind, placebo-controlled STOP-HS1 and STOP-HS2 phase 3 trials
Source: Nat Med. 2026 Jul 23;32(8):3071–81. doi: 10.1038/s41591-026-04534-z (PMC13473019; doi:10.1038/s41591-026-04534-z)
Supplement: Supplementary file 1 [file 41591_2026_4534_MOESM1_ESM.pdf]

nature medicine

Supplementary information https://doi.org/10.1038/s41591-026-04534-z

Povorcitinib for hidradenitis suppurativa: the randomized, double-blind, placebo-controlled STOP-HS1 and STOP-HS2 phase 3 trials

In the format provided by the authors and unedited

Table of Contents

STOP-HS1 Study Group: List of Investigators, Sub-Investigators, and Study Sites.................... 2

STOP-HS2 Study Group: List of Investigators, Sub-Investigators, and Study Sites...................15

STOP-HS1 Institutional Review Boards and/or Ethics Committees..27

STOP-HS2 Institutional Review Boards and/or Ethics Committees..28

Table S1. STOP-HS1 and STOP-HS2 Pooled Baseline Demographics and Disease Characteristics by Country or Geographic Region..30

Table S2. HiSCR50, HiSCR75, HiSCR90 and HiSCR100 Responses (OC and mNRI) at Weeks 12, 24, and 54 32

Table S3. STOP-HS1 and STOP-HS2 Pooled Maintenance of HiSCR50 and HiSCR75 During Extension Period in Patients Randomised to Povorcitinib (mNRI)...34

Table S4. STOP-HS1 and STOP-HS2 Pooled Skin Pain NRS: Proportion of Patients in Each Categorical Score at Baseline and Weeks 12, 24, and 54 (mNRI)...35

Table S5. AN and dT Counts at Baseline and Weeks 3, 12, 24, and 54 (OC) 37

Table S6. STOP-HS1 and STOP-HS2 Pooled Lesion-Specific Response Rates at Weeks 12, 24, and 54 (OC) 38

Table S7. STOP-HS1 and STOP-HS2 Pooled TEAEs by Severity Grade in the Full Study Period 43

Table S8. STOP-HS1 and STOP-HS2 Pooled Incidence and Exposure-Adjusted Rates of TEAEs (≥5%), AESIs, and ECIs Through Week 54 by Treatment Regimen 44

Table S9. Select Safety Narratives..45

Table S10. STOP-HS1 and STOP-HS2 Pooled, Additional Information on TEAEs of Acne Through Week 54 48

Table S11. STOP-HS1 and STOP-HS2 Pooled Worst Laboratory Grade Shifts From Baseline During Study Periods 49

Figure S1. Skin Pain NRS30 Response Over Time Through Week 54 (Among Patients With Skin Pain NRS ≥3 at Baseline; OC and mNRI)..55

Figure S2. STOP-HS1 and STOP-HS2 Pooled IHS4 Responses From Randomisation Through Week 54 (OC)...56

Figure S3. STOP-HS1 and STOP-HS2 Pooled Median Percentage Change in hsCRP From Baseline Through Week 54 (OC) 57

Figure S4. Graphic Approach for Multiplicity Adjustment for Testing Primary and Key Secondary Endpoints..58

STOP-HS1 Protocol……………………………………………………………………………………59

STOP-HS2 Protocol…………………………………………………………………………………...190

STOP-HS1/STOP-HS2 Statistical Analysis Plan………………………………………………..321

STOP-HS1 Study Group: List of Investigators, Sub-Investigators, and Study Sites

Country	Investigator/Sub-Investigator	Position/Role	Study Site
Austria	Christian Posch	Principal Investigator	Krankenhaus Hietzing M. Neurolog. Zentrum Rosenhugel, Wien, Austria, 1130
	Katharina Cavada	Sub-Investigator	
	Katrin Mihalics	Sub-Investigator	
	Sophie Burner	Sub-Investigator	
	Paul Sator	Sub-Investigator	
	Johannes Bisschoff	Principal Investigator	Medizin am Hauptbahnhof, Wien, Austria, 1100
	Stephanie Geisler	Sub-Investigator	
	Berthold Rzany	Sub-Investigator	
	Barbara Ebner	Principal Investigator	Ordensklinikum Linz GmbH Elisabethinen, Linz, Austria, 4020
	Astrid Badescu	Sub-Investigator	
	Elisabeth Fabian	Sub-Investigator	
	Susanne Kallab	Sub-Investigator	
	Doris Koch-Thalhammer	Sub-Investigator	
	Konrad Lehner	Sub-Investigator	
	Rebecca Ram	Sub-Investigator	
	Norbert Sepp	Previous Investigator	
	Peter Wolf	Principal Investigator	Landeskrankenhaus Universitatsklinikum Graz, Graz, Austria, 8036
	Romana Kupsa*	Previous Principal Investigator/Sub-Investigator	
	Katja Grossschaedl	Sub-Investigator	
	Elisabeth Jungwirth	Sub-Investigator	
	Paul Gressenberger	Sub-Investigator	
	Aline Blanke	Sub-Investigator	
	Constanze Jonak	Principal Investigator	Allgemeines Krankenhaus der Stadt Wien, Vienna, Austria, 1090
	Johannes Griss	Sub-Investigator	
	Carina Borst	Sub-Investigator	
	Matthias Schmuth	Principal Investigator	Medizinische Universität Innsbruck, Innsbruck, Austria, 6020
	Stefan Blunder	Sub-Investigator	
	Christine Brunner	Sub-Investigator	
	Lara Durakbasa	Sub-Investigator	
	Robert Gruber	Sub-Investigator	
	Angelika Le Beau	Sub-Investigator	
	Fiona Andre	Sub-Investigator	
	Eliane Steinicke	Sub-Investigator	
	Thomas Trafoier	Sub-Investigator	
	Luca Tuertscher	Sub-Investigator	
Belgium	Veronique del Marmol	Principal Investigator	ULB Hospital Erasme, Bruxelles, Belgium, 1070
	Farida Benhadou	Sub-Investigator	
	Anne Sophie Sarkis	Sub-Investigator	
	Katharina Wunderlich	Sub-Investigator	
	Jo Lambert	Principal Investigator	

	Katia Ongenae	Sub-Investigator	Universitair Ziekenhuis Gent (UZ Gent), Gent, Belgium, 9000
	Reinhart Speeckaert	Sub-Investigator	
	Arjen Nikkels	Principal Investigator	Centre Hospitalier Universitaire de Liege - Sart Tilman, Liege, Belgium, 4000
	Thibaut Delahaye	Sub-Investigator	
	Constance Groszmann	Sub-Investigator	
	Gaelle Jouret	Sub-Investigator	
	Dalal Lahrichi	Sub-Investigator	
	Florence Libon	Sub-Investigator	
	Mathilde Longree	Sub-Investigator	
	Sophie Bailleux	Sub-Investigator	
	Yseult Senterre	Sub-Investigator	
	Amandine Bouillenne	Sub-Investigator	
	Olivier Vanhooteghem	Principal Investigator	CHU UCL Namur De Saint Elisabeth, Namur, Belgium, 5000
	Rita Makhoul	Sub-Investigator	
	Pierre-Dominique Ghislain	Principal Investigator	Cliniques Universitaires UCL Saint-Luc, Brussels, Belgium, 1200
	Laura Nobile	Sub-Investigator	
	An Van Laethem	Principal Investigator	Universitaire Ziekenhuis Leuven - Gasthuisberg, Leuven, Belgium, 3000
	Tom Hillary	Sub-Investigator	
	Esther Lissens	Principal Investigator	AZ Sint-Lucas, Gent, Belgium, 9000
	Bernard Kint	Sub-Investigator	
Canada	Marni Wiseman	Principal Investigator	Wiseman Dermatology Research Inc, Winnipeg, Canada, R3M 3Z4
	Danielle Roy	Sub-Investigator	
	Andrea Mirmulstein	Sub-Investigator	
	Melinda Gooderham	Principal Investigator	Skin Centre For Dermatology, Peterborough, Canada, K9J 5K2
	William Swales	Sub-Investigator	
	Ashley O'Toole	Sub-Investigator	
	Wei Loo	Principal Investigator	DermEffects, London, Canada, N6H 5L5
	Denise Wexler	Sub-Investigator	
	Keng Yeow Tay	Sub-Investigator	
	Faria Rubab	Sub-Investigator	
	Maryam Shayesteh Alam	Principal Investigator	SimcoMed Health Ltd, Barrie, Canada, L4M 1G7
	Mahnaz Mahmoodi	Sub-Investigator	
	Panteha Eshtiaghi	Sub-Investigator	
	Danielle Brassard	Principal Investigator	Clinique D, Laval, Canada, H7N 6L2
	Angelique Gagne-Henley	Sub-Investigator	
	Camille Loranger	Sub-Investigator	
	Robert Bissonnette	Principal Investigator	Innovaderm Research Inc., Montreal, Canada, H2X 2V1
	Catherine Maari	Sub-Investigator	
	Etienne Saint-Cyr	Sub-Investigator	
	Jose Hermenio Cavalcante Lima Filho	Principal Investigator	Leader Research, Hamilton, Canada, L8L 3C3
	Rosilene Lanzini	Sub-Investigator	

	Helene Veillette	Principal Investigator	Diex Recherche Quebec Inc., Quebec, Canada, G1V 4T3
	Helene Doucet Beaupre	Sub-Investigator	
Czech Republic	Alena Machovcova	Principal Investigator	Fakultni Nemocnice V Motole, Praha 5, Czech Republic, 150 06
	Gabriela Janouskova	Sub-Investigator	
	Katerina Svarcova	Sub-Investigator	
	Yvetta Vantuchova	Principal Investigator	Fakultni Nemocnice Ostrava, Ostrava - Poruba, Czech Republic, 708 52
	Jana Novakova	Sub-Investigator	
Germany	Florian Schenck	Principal Investigator	Hautarzte Zentrum-Hannover, Hannover, Germany, 30159
	Markus Kaspari	Sub-Investigator	
	Andreas Pinter	Principal Investigator	Universitatsklinikum Frankfurt, Frankfurt, Germany, 60590
	Lea Daniello	Sub-Investigator	
	Tobias Dietlein	Sub-Investigator	
	Julia Eichhorn	Sub-Investigator	
	Ufuk Erdogdu	Sub-Investigator	
	Franziska Graen	Sub-Investigator	
	Marilena Hess	Sub-Investigator	
	Nora Kauffmann	Sub-Investigator	
	Emilie Laurent	Sub-Investigator	
	Guelcin Mengi	Sub-Investigator	
	Nadia Ninosu	Sub-Investigator	
	Martina Sarah Poeschl	Sub-Investigator	
	Georgios Tassopoulos	Sub-Investigator	
	Mariam Urban	Sub-Investigator	
	Roland Aschoff	Principal Investigator	Universitaetsklinikum Carl Gustav Carus Tu Dresden, Dresden, Germany, 01307
	Helena Hasler	Sub-Investigator	
	Stefanie Heyne	Sub-Investigator	
	Paulka Karitzky	Sub-Investigator	
	Susann Meisterfeld	Sub-Investigator	
	Ulrike Proske	Sub-Investigator	
	Andrea Bauer	Sub-Investigator	
	Matthias Augustin	Principal Investigator	Universitatsklinikum Hamburg Eppendorf, Hamburg, Germany, 20246
	Hatice Birkner	Sub-Investigator	
	Slatina Christov	Sub-Investigator	
	Nomun Ganjuur	Sub-Investigator	
	Franziska Gensel	Sub-Investigator	
	Caroline Gewiss	Sub-Investigator	
	Carolin Grote	Sub-Investigator	
	Christine Lee Seifert	Sub-Investigator	
	Brigitte Stephan	Sub-Investigator	
	Jan Wagner	Sub-Investigator	
	Charlotte Willers	Sub-Investigator	
	Nesrine Ben-Anaya	Sub-Investigator	
	Beate Schwarz	Principal Investigator	

	Susanne Arendt-Puke	Sub-Investigator	Praxis Dr. Beate Schwarz, Langenau, Germany, 89129
	Simone Fuenkele	Sub-Investigator	
	Mirsada Mulavdic	Sub-Investigator	
	Mana Zur Brügge (Née Sarlak)*	Principal Investigator/Sub-Investigator	Klinikum Darmstadt Gmbh, Darmstadt, Germany, 64283
	Maurizio Podda	Previous Principal Investigator	
	Laura Gotta	Sub-Investigator	
	Maximilian Kovacs	Sub-Investigator	
	Norman-Philipp Hoff	Principal Investigator	University of Dusseldorf, Duesseldorf, Germany, 40225
	Geraldine Anna Bihoi	Sub-Investigator	
	Sarah Janssen	Sub-Investigator	
	Harm Henning Lindhof	Sub-Investigator	
	Barbora Reznakova	Sub-Investigator	
	Bernhard Homey	Previous Investigator	
	Robin Springer	Sub-Investigator	
	Nicolai Treiber*	Principal Investigator/Sub-Investigator	Beldio Research GmbH, Memmingen, Germany, 87700
	Andreas Schwinn	Previous Principal Investigator	
	Leys Anita Luya Estrada	Sub-Investigator	
Spain	Alejandro Molina Leyva	Principal Investigator	Hospital Universitario Virgen De Las Nieves, Granada, Spain, 18014
	Carlos Cuenca Barrales	Sub-Investigator	
	Sofia M Haselgruber de Francisco	Sub-Investigator	
	Trinidad Montero-Vilchez	Sub-Investigator	
	Julio Bassas-Vila	Principal Investigator	Ceim Hospital Universitari Germans Trias i Pujol, Badalona, Spain, 08916
	Veronica Mora Fernandez	Sub-Investigator	
	Gemma Martin Ezquerra	Principal Investigator	Hospital Del Mar, Barcelona, Spain, 08003
	Marta Ferran Farres	Sub-Investigator	
	Ramon Maria Pujol Vallverdu	Sub-Investigator	
	Pedro Pinto	Previous Investigator	
	Maria Isabel Rodriguez Blanco	Principal Investigator	Complejo Hospitalario Universitario De Santiago, Santiago De Compostela, Spain, 15706
	Hugo Alberto Vazquez Veiga*	Previous Principal Investigator/Sub-Investigator	
	Manuel Javier Ginarte Val	Sub-Investigator	
	Daniel Gonzalez Vilas	Sub-Investigator	
	Noelia Moreiras Arias	Sub-Investigator	
	Raquel Rivera Diaz	Principal Investigator	Hospital Universitario 12 De Octubre, Madrid, Spain, 28041
	Jorge Arroyo Andres	Sub-Investigator	
	Daniel Falkenhain Lopez	Sub-Investigator	

	Concepcion Postigo Llorente	Sub-Investigator	
France	Herve Bachelez	Principal Investigator	Hospital Saint Louis, Cedex 10, France, 75010
	Charles Cassius	Sub-Investigator	
	Estelle Charvet	Sub-Investigator	
	Alia Galadari	Sub-Investigator	
	Thibault Mahevas	Sub-Investigator	
	Jean Luc Perrot	Principal Investigator	University Hospital of Saint Etienne, Saint Priest En Jarez, France, 42270
	Chloé David Ferreira	Sub-Investigator	
	Sarah Kourdjee	Sub-Investigator	
	Estelle Ravni	Sub-Investigator	
	Celine Bourgea	Sub-Investigator	
	Gaelle Quereux	Principal Investigator	Centre Hospitalier Universitaire de Nantes (CHU de Nantes) - Hotel-Dieu, Nantes, France, 44093
	Sarah Le Naour	Sub-Investigator	
	Marie Le Moigne	Sub-Investigator	
	Laurent Misery	Principal Investigator	Hospital Morvan, Brest Cedex 2, France, 29609
	Emilie Brenaut	Sub-Investigator	
	Claire Abasq-Thomas	Sub-Investigator	
	Nadia Salinas	Sub-Investigator	
	Marie-Aleth Richard	Principal Investigator	CHU Hopital De La Timone, Marseille, France, 13385
	Jessica Fareau	Sub-Investigator	
	Laurie Gosse	Sub-Investigator	
	Nausicaa Malissen	Sub-Investigator	
	Stephanie Mallet	Sub-Investigator	
	Sandrine Monestier-Gasmi	Sub-Investigator	
	Jilliana Monnier	Sub-Investigator	
	Chirine Rezzag Mahcene	Sub-Investigator	
	Berangere Demarez	Sub-Investigator	
	Caroline Gaudy	Sub-Investigator	
	Claire Sarles	Sub-Investigator	
	Olivier Cogrel	Principal Investigator	Bordeaux CHU Hopital Saint - Andre, Bordeaux, France, 33000
	Paul Cirotteau	Sub-Investigator	
	Celine Eldani	Sub-Investigator	
	Clementine Toussant	Sub-Investigator	
	Serge Boulinguez	Principal Investigator	CHU De Toulouse Hopital Larrey Centre De Reference Des Maladies Rares De La Peau Service De Dermatol, Toulouse Cedex 9, France, 31059
	Amel Bouznad	Sub-Investigator	
	Aurore Brun	Sub-Investigator	
	Cristina Livideanu	Sub-Investigator	
	Carle Paul	Previous Principal Investigator/Sub-Investigator	
	Nathalie Beneton Benhard	Principal Investigator	Centre Hospitalier Du Mans, Le Mans Cedex 9, France, 72037
	Corina Bara	Sub-Investigator	
	Mathilde Letzelter	Sub-Investigator	
	Herve Maillard	Sub-Investigator	

	Romain Samaran	Sub-Investigator	
	Arthur Vrignaud	Sub-Investigator	
Greece	Evangelos Giamarellos-Bourboulis	Principal Investigator	University Hospital of West Attica - Attikon, Athens, Greece, 12462
	Christina Bolanou	Sub-Investigator	
	Christina Damoulari	Sub-Investigator	
	Nikolaos Antonakos	Sub-Investigator	
	Theodora Kanni	Sub-Investigator	
	Antigone Kotsaki	Sub-Investigator	
	Evdoxia Kyriazopoulou	Sub-Investigator	
	Konstantinos Leventogiannis	Sub-Investigator	
	Christos Psarrakis	Sub-Investigator	
	Asimina Safarika	Sub-Investigator	
	Dimitra Stergianou	Sub-Investigator	
	Styliani Theochari	Sub-Investigator	
	Eleni Sotiriou*	Principal Investigator/Sub-Investigator	Skin And Venereal Diseases' Hospital, Thessaloniki, Greece, 54643
	Aggeliki Panagopoulou	Sub-Investigator	
	Ilias Papadimitriou	Sub-Investigator	
	Dimitrios Ioannides	Previous Principal Investigator	
	Efstratios Vakirlis	Sub-Investigator	
	Elisavet Lazaridou	Principal Investigator	General Hospital Papageorgiou, Thessaloniki, Greece, 56403
	Zoe Apalla	Sub-Investigator	
	Elisavet Miggiani	Sub-Investigator	
	Aikaterini Patsatsi	Sub-Investigator	
	Stefanos Pipeles	Sub-Investigator	
	Myrto Georgia Trakatelli	Sub-Investigator	
	Anastasia Trigoni	Sub-Investigator	
	Fragkiski Tsatsou	Sub-Investigator	
	Alexandros Stratigos	Principal Investigator	'Andreas Syggros' Hospital, Athens, Greece, 16121
	Aikaterini Liakou	Sub-Investigator	
	Eleni Chatzidimitriou	Sub-Investigator	
Japan	Ayano Maruyama*	Principal Investigator/Sub-Investigator	University Hospital Kyoto Prefectural University of Medicine, Kyoto-Shi, Japan, 602-8566
	Takahiro Arita	Sub-Investigator	
	Natsue Ioka	Sub-Investigator	
	Hiroyuki Komori	Sub-Investigator	
	Jun Asai	Previous Principal Investigator	
	Makoto Nakae	Sub-Investigator	
	Masanari Takamizo	Sub-Investigator	
	Manaka Ushida	Sub-Investigator	
	Yuki Yokoi	Sub-Investigator	

	Teruki Yanagi	Principal Investigator	University of the Ryukyus Hospital, Ginowan-Shi, Japan, 901-2725
	Takuya Omine	Previous Principal Investigator	
	Kiriko Ishikawa	Sub-Investigator	
	Takuya Miyagi	Sub-Investigator	
	Aoi Ohira	Sub-Investigator	
	Sayaka Yamaguchi	Sub-Investigator	
	Ryoko Omori	Principal Investigator	Tohoku University Hospital, Sendai-Shi, Japan, 980-8574
	Tetsuya Ikawa	Sub-Investigator	
	Airi Kobayashi	Sub-Investigator	
	Toshiki Okazaki	Sub-Investigator	
	Mana Sekine	Sub-Investigator	
	Toshiya Takahashi	Sub-Investigator	
	Hitoshi Terui	Sub-Investigator	
	Emi Yamazaki	Sub-Investigator	
	Takehiro Takahashi	Sub-Investigator	
	Yuki Saito*	Principal Investigator/Sub-Investigator	Niigata University Medical & Dental Hospital, Niigata-Shi, Japan, 951-8520
	Natsumi Hama	Sub-Investigator	
	Akito Hasegawa	Sub-Investigator	
	Ryota Hayashi	Sub-Investigator	
	Akihiko Yuki	Previous Principal Investigator	
	Riichiro Abe	Sub-Investigator	
	Risa Hagiwara	Sub-Investigator	
	Tatsuya Katsumi	Sub-Investigator	
	Nusrat Akhtercruickshank	Previous Principal Investigator	
	Koremasa Hayama	Principal Investigator	Nihon University Itabashi Hospital, Itabashi-Ku, Japan, 173-8610
	Hideki Fujita	Sub-Investigator	
	Natsumi Ikumi	Sub-Investigator	
	Satoshi Izaki	Sub-Investigator	
	Kumiko Kimura	Sub-Investigator	
	Nobuyuki Nishimori	Sub-Investigator	
	Yusuke Niwa	Sub-Investigator	
	Maho Tagui	Sub-Investigator	
	Hiroshi Koga*	Principal Investigator/Sub-Investigator	Kurume University Hospital, Kurume-Shi, Japan, 830-0011
	Norito Ishii	Sub-Investigator	
	Eri Katayama	Sub-Investigator	
	Hiroyuki Kodama	Sub-Investigator	
	Takekuni Nakama	Previous Principal Investigator	
	Hiroyuki Morita	Sub-Investigator	

	Keiko Hashikawa	Sub-Investigator	
	Yohei Natsuaki	Sub-Investigator	
	Hiroshi Oyama	Sub-Investigator	
	Ayaka Ozono	Sub-Investigator	
	Toshihiro Shirahama	Sub-Investigator	
	Mari Soeda	Sub-Investigator	
	Akihiro Koga	Previous Investigator	
	Akihiro Takagi	Previous Investigator	
	Toshifumi Nomura	Principal Investigator	University of Tsukuba Hospital, Tsukuba-Shi, Japan, 305-8576
	Yoshiyuki Ishii	Sub-Investigator	
	Shoichiro Ishizuki	Sub-Investigator	
	Noriko Kubota	Sub-Investigator	
	Keisuke Anjyu	Sub-Investigator	
	Ichiro Kurokawa	Principal Investigator	Meiwa Hospital, Nishinomiya-Shi, Japan, 663-8186
	Tomonori Ishihara	Sub-Investigator	
	Kanako Kita	Sub-Investigator	
	Mika Watanabe*	Principal Investigator/Sub-Investigator	Hokkaido University Hospital, Sapporo-Shi, Japan, 060-8648
	Shiori Hikichi	Sub-Investigator	
	Hideyuki Kosumi	Sub-Investigator	
	Takuya Maeda	Sub-Investigator	
	Shoko Mai	Sub-Investigator	
	Yosuke Mai	Sub-Investigator	
	Keiko Mitsuda	Sub-Investigator	
	Satsuki Naruse	Sub-Investigator	
	Ken Natsuga	Sub-Investigator	
	Takuma Nohara	Sub-Investigator	
	Chihiro Shiiya	Sub-Investigator	
	Mayuna Shimano	Sub-Investigator	
	Arisa Tanaka	Sub-Investigator	
	Keiko Tokuchi	Sub-Investigator	
	Hideyuki Ujiie	Sub-Investigator	
	Meri Watanabe	Sub-Investigator	
	Takuya Fujimoto	Sub-Investigator	
	Teruki Yanagi	Previous Principal Investigator	
	Noriyasu Sakai	Principal Investigator	Tokyo Medical University Hospital, Shinjuku-Ku, Japan, 160-0023
	Ryokichi Irisawa	Sub-Investigator	
	Tomonobu Ito	Sub-Investigator	
	Yukari Okubo	Sub-Investigator	
	Hiroko Wakimoto	Sub-Investigator	
Netherlands	Hessel van der Zee	Principal Investigator	Erasmus Medisch Centrum, Rotterdam, Netherlands, 3015 GD
	Errol Prens*	Previous Principal Investigator	
	Pim Aarts	Sub-Investigator	

	Jessie Aitken	Sub-Investigator	
	Daan De Kort	Sub-Investigator	
	Colin Spence	Sub-Investigator	
	Johanna Catharina Van Huijstee	Sub-Investigator	
	Sebastiaan Prens	Principal Investigator	Ampia Zienkenhuis - Locatie Molengracht Breda, Breda, Netherlands, 4818 CK
	Mick Asselman	Sub-Investigator	
	Melissa de Meza	Sub-Investigator	
	Ralph Heijmans	Sub-Investigator	
	Jacobus Nuiten	Sub-Investigator	
	Seray Oz	Sub-Investigator	
	Barbara Horvath	Principal Investigator	Universitair Medisch Centrum Groningen, Groningen, Netherlands, 9713 GZ
	Nicole Koerts	Sub-Investigator	
	Romy Toxopeus	Sub-Investigator	
	Despoina Tsampazi	Sub-Investigator	
	Marjolein Albert Julia Hiel	Sub-Investigator	
Poland	Jacek Szepietowski	Principal Investigator	Cityclinic Przychodnia Lekarsko-Psychologiczna Matusiak Spolka Partnerska, Wroclaw, Poland, 50-566
	Beata Bien	Sub-Investigator	
	Edyta Lelonek	Sub-Investigator	
	Lukasz Matusiak	Sub-Investigator	
	Ewa Wojcik	Sub-Investigator	
	Jolanta Weglowska	Principal Investigator	Dermmedica Sp. Z O.O., Wrocław, Poland, 51-503
	Anna Czajkowska	Sub-Investigator	
	Piotr Jarlinski	Sub-Investigator	
	Wojciech Przywara	Sub-Investigator	
	Waldemar Stepien	Sub-Investigator	
	Anna Domagala	Sub-Investigator	
	Zygmunt Adamski	Principal Investigator	Solumed Centrum Medyczne, Poznan, Poland, 60-529
	Kinga Adamska	Sub-Investigator	
	Adrianna Liberska-Nobis	Sub-Investigator	
	Piotr Parcheta	Principal Investigator	Dermedic Dr. Zdybski, Ostrowiec Swietokrzyski, Poland, 27-400
	Joanna Dlugosz-Bukowska	Sub-Investigator	
	Klaudia Tutka Prystupa	Sub-Investigator	
	Artur Wrona	Sub-Investigator	
	Jacek Zdybski	Previous Investigator/Sub-Investigator	
USA	Soyeon Paek	Principal Investigator	Texas Dermatology Research Center, Dallas, USA, 75246
	Claire Jung	Sub-Investigator	
	Benjamin Ehst	Principal Investigator	Oregon Medical Research Center, Portland, USA, 97201
	Andrew Blauvelt	Previous Principal Investigator/Sub-Investigator	
	Timothy Carrasco	Sub-Investigator	
	Robert Matheson	Sub-Investigator	

	Jason Hawkes	Sub-Investigator	
	Milan Anadkat	Principal Investigator	Washington University School of Medicine Dermatology, St. Louis, USA, 63108
	Amy Musiek	Sub-Investigator	
	Lindsay Ackerman	Principal Investigator	Medical Dermatology Specialists Phoenix, Phoenix, USA, 85006
	Christina Harview	Sub-Investigator	
	Margaret Kessler	Sub-Investigator	
	Adam Luber	Sub-Investigator	
	Randi Rubenzik	Sub-Investigator	
	Alexandra Kimball	Principal Investigator	Beth Israel Deaconess Medical Center, Boston, USA, 02215
	Julia Gao	Sub-Investigator	
	Ruby Gibson	Sub-Investigator	
	Tracey Otto	Sub-Investigator	
	Martina Porter	Sub-Investigator	
	Sally Tan Renehan	Sub-Investigator	
	Maneli Tehrani	Sub-Investigator	
	Bruna Wafae	Sub-Investigator	
	Ira Thorla	Principal Investigator	Delricht Research, Baton Rouge, USA, 70809
	Sarah McGinty	Sub-Investigator	
	Eduardo Weiss	Principal Investigator	Skin Care Research, LLC SCR Hollywood, Hollywood, USA, 33021
	Nancy Muskat	Sub-Investigator	
	Sandra Johnson	Principal Investigator	Johnson Dermatology, Fort Smith, USA, 72916
	Scarlett Bone	Sub-Investigator	
	Katherine Reed	Principal Investigator	Dermatology Specialists of Spokane, Spokane, USA, 99202
	Regan Brown	Sub-Investigator	
	Ashley Lundgren	Sub-Investigator	
	Matthew Zook	Principal Investigator	Olympian Clinical Research, Tampa, USA, 33615
	Patricia Delgado	Sub-Investigator	
	Laura Gulick	Sub-Investigator	
	April Silverberg	Sub-Investigator	
	Panayiotis Vasiloudes	Sub-Investigator	
	Kelley Yokum	Sub-Investigator	
	Giselle Torres-Bonilla	Previous Investigator	
	Mark Amster	Principal Investigator	Metro Boston Clinical Partners, Brighton, USA, 02135
	Richard Black	Sub-Investigator	
	Mark Knautz	Principal Investigator	Marietta Dermatology The Skin Cancer Center Marietta, Marietta, USA, 30060
	George Dobo	Sub-Investigator	
	Jamie Groh	Sub-Investigator	
	Neil Korman	Principal Investigator	University Hospitals Cleveland Medical Center, Cleveland, USA, 44106
	Amy Johnson	Sub-Investigator	
	Jordan Kahle	Sub-Investigator	
	Jack Fite	Sub-Investigator	

	Emma Larson	Sub-Investigator	
	Maleck Saleh	Sub-Investigator	
	David Pariser	Principal Investigator	Virginia Clinical Research Inc, Norfolk, USA, 23502
	Robert Pariser	Sub-Investigator	
	Tina Watkins	Sub-Investigator	
	Alan Fleischer*	Principal Investigator/Sub-Investigator	University Of Cincinnati, Cincinnati, USA, 45219
	Debra Breneman	Previous Principal Investigator	
	Katherine Baquerizo Nole	Sub-Investigator	
	Hassan Hassan	Sub-Investigator	
	Tanya Evans	Principal Investigator	Avance Clinical Trials, Laguna Niguel, USA, 92677
	Tessa Buzzetti	Sub-Investigator	
	David Thornsberry	Sub-Investigator	
	Eva-Maria Heurich	Principal Investigator	ARS - Maitland Clinical Research Unit, Maitland, USA, 32751
	Debra Grayman*	Previous Principal Investigator/Sub-Investigator	
	Sedigheh Ebadi	Sub-Investigator	
	Szheckera Fearon	Sub-Investigator	
	Andrea Marraffino	Sub-Investigator	
	Roy Richardson	Sub-Investigator	
	Leomaris Trujillo	Sub-Investigator	
	Veronica Walker	Sub-Investigator	
	Frank Don	Principal Investigator	Direct Helpers Research Center, Hialeah, USA, 33012-3618
	Manuel Sanchez	Sub-Investigator	
	Abel Jarell	Principal Investigator	ActivMed Practices & Research, LLC Beverly, Portsmouth, USA, 03801
	Bertrand Cole	Sub-Investigator	
	Polly Jarell	Sub-Investigator	
	Christopher Mancuso	Sub-Investigator	
	Sarah O Connor	Sub-Investigator	
	Evelyn Stein	Sub-Investigator	
	Stephanie Mehlis	Principal Investigator	Northshore Medical Group Dermatology Skokie, Skokie, USA, 60077
	Shannon Ewing	Sub-Investigator	
	Joel Joyce	Sub-Investigator	
	Ann Reed	Principal Investigator	Skin Care Research, LLC SCR Hollywood, Boca Raton, USA, 33486
	Chere Lucas Anthony	Sub-Investigator	
	Lisa Tack	Sub-Investigator	
	Tory Sullivan	Principal Investigator	Sullivan Dermatology North Miami Beach Office, North Miami Beach, USA, 33162
	Kristyn Cullen	Sub-Investigator	
	Randi Loomis	Sub-Investigator	
	Paradi Mirmirani	Principal Investigator	Kaiser Permanente, San Francisco, USA, 94118
	Christine Jacobson	Sub-Investigator	
	Christine C Jacobson	Sub-Investigator	

	Kenneth Katz	Sub-Investigator	
	Bhavnit Bhatia	Sub-Investigator	
	Natalie Viakhireva	Sub-Investigator	
	Jeffrey Weinberg	Principal Investigator	Forest Hills Dermatology Group, Kew Gardens, USA, 11415
	Vicky Luo	Sub-Investigator	
	David Petrosian	Sub-Investigator	
	Chesahna Kindred Weaver	Principal Investigator	Care Access Research LLC, Marriottsville, USA, 21104-8901
	Courtney Green	Sub-Investigator	
	Beverly Johnson	Sub-Investigator	
	Howard Sofen	Principal Investigator	Dermatology Research Associates, Los Angeles, USA, 90045
	Rita Price	Sub-Investigator	
	Julia Mhlaba	Principal Investigator	Northwestern University Feinberg School of Medicine Department of Dermatology, Chicago, USA, 60611
	Kelsey Flood	Sub-Investigator	
	Itzel Lara Landa	Sub-Investigator	
	Patrick Shannon*	Principal Investigator	Optima Dermatology Optima Research, Boardman, USA, 44512
	Sarah Marlinski Merlo	Sub-Investigator	
	George Shanno	Previous Investigator	
	John Durkin	Principal Investigator	University of New Mexico Hospital, Albuquerque, USA, 87102
	Therese Holguin	Sub-Investigator	
	Robert Koppel	Principal Investigator	Clinical Trials Management LLC, Metairie, USA, 70006
	Rebecca Lee Delahoussaye Soine	Sub-Investigator	
	Douglas Koppel	Sub-Investigator	
	Cheryl Hull	Principal Investigator	Northwest Arkansas Clinical Trials Center, Rogers, USA, 72758
	Seth Forman	Principal Investigator	ForCare Clinical Research, Tampa, USA, 33613
	Casey Viera	Sub-Investigator	
	Deborah White	Sub-Investigator	
	Tanya Bogle	Sub-Investigator	
	Maria Hicks	Sub-Investigator	
	Kenneth Dawes	Principal Investigator	Dawes Fretzin Clinical Research Group, Indianapolis, USA, 46250
	Jennifer Conner	Sub-Investigator	
	Scott Fretzin	Sub-Investigator	
	Elizabeth Golden	Sub-Investigator	
	Michael Isaacs	Sub-Investigator	
	Courtney Keasey	Sub-Investigator	
	Presley Pearman	Sub-Investigator	
	Christina Race	Sub-Investigator	
	Brad Glick	Principal Investigator	GSI Clinical Research, Margate, USA, 33063
	Allan Snyder	Sub-Investigator	
	Matthew Zirwas	Principal Investigator	

	Sherri Howard	Sub-Investigator	Bexley Dermatology, Bexley, USA, 43209
	Craig Teller	Principal Investigator	Bellaire Dermatology Associates BDA, Bellaire, USA, 77401
	Shelby Bonn	Sub-Investigator	
	Courtney Brown	Sub-Investigator	
	Casey Wang	Sub-Investigator	
	James Allred	Principal Investigator	Equity Medical, LLC, Bowling Green, USA, 42104
	Kyle Herald	Sub-Investigator	

* Investigators with more than one role due to changes in study responsibilities at the site.

STOP-HS2 Study Group: List of Investigators, Sub-Investigators, and Study Sites

Country	Investigator/Sub-Investigator	Position/Role	Study Site
Australia	John Frew	Principal Investigator	Liverpool Hospital, Liverpool, Australia, 2170
	Samuel Der Sarkissian	Sub-Investigator	
	James Pham	Sub-Investigator	
	Peter Foley	Principal Investigator	Skin Health Institute Inc., Carlton, Australia, 3053
	Nagalaxmi Iyengar	Sub-Investigator	
	Conor Larney	Sub-Investigator	
	Jacqueline Nguyen	Sub-Investigator	
	Nisal Punchihewa	Sub-Investigator	
	Michael Freeman	Principal Investigator	The Skin Centre, Benowa, Australia, 4217
	Andrew Freeman	Sub-Investigator	
	Juliana Junger	Principal Investigator	Novatrials, Charlestown, Australia, 2290
	Anna Creelman	Sub-Investigator	
	Oscar Cumming	Sub-Investigator	
	Renata Fuljahn	Sub-Investigator	
	Sheree Joyce	Sub-Investigator	
	Benjamin Cowley	Sub-Investigator	
	Toni Mccallum Pardey	Sub-Investigator	
	Eliza Milliken	Sub-Investigator	
	Adam Daunton	Principal Investigator	Dr Rodney Sinclair Pty Ltd, Melbourne, Australia, 3002
	Ragini Ghiya	Sub-Investigator	
	Meghana Paranjape	Sub-Investigator	
	Ritva Vyas	Previous Principal Investigator	
	Valerie Bai Jia Yii	Sub-Investigator	
	Nicole Yoong	Sub-Investigator	
	Deirdre Murrell	Principal Investigator	Premier Specialists Pty Ltd, Kogarah, Australia, 2217
	Ben Koszegi	Sub-Investigator	
	Sera Sarsam	Sub-Investigator	
	Brice Serquina	Sub-Investigator	
	Madeleine Stark	Sub-Investigator	
	Corey Stone	Sub-Investigator	
	Jade Tangqi Ng	Sub-Investigator	
	Liana Victory	Sub-Investigator	
	Lynda Spelman	Principal Investigator	Veracity Clinical Research, Woolloongabba, Australia, 4102
	Jonathan Peek	Sub-Investigator	
	Khine Kyaw	Sub-Investigator	
	Aimal Fatima	Sub-Investigator	
	Cody Frear	Sub-Investigator	
	Erin Mcmeniman	Principal Investigator	Princess Alexandra Hospital Australia, Woolloongabba, Australia, 4102
	Kiarash Khosrotehrani*	Previous Principal Investigator/Sub-Investigator	
	Timothy Liu	Sub-Investigator	

	Jonathan Peek	Sub-Investigator	
	Peter Soyer	Sub-Investigator	
	Deborah Toledo Flores	Sub-Investigator	
Bulgaria	Maria Ratkova	Principal Investigator	Medical Center Hera EOOD, Sofia, Bulgaria, 1510
	Dzhemile Dervish	Sub-Investigator	
	Desislava Dimitrova	Sub-Investigator	
	Hristo Mangarov	Sub-Investigator	
	Maya Stoyanova-Kurtisheva	Sub-Investigator	
	Evgeniya Hristakieva	Principal Investigator	Umhat Prof. Dr. Stoyan Kirkovich AD, Stara Zagora, Bulgaria, 6000
	Radostina Deliyska	Sub-Investigator	
	Tanya Gancheva	Sub-Investigator	
	Desislava Gancheva Panayotova	Sub-Investigator	
	Rositsa Lavcheva	Sub-Investigator	
	Plamen Minkov	Sub-Investigator	
	Gavrail Poterov	Sub-Investigator	
	Zhenya Troeva-Hristova	Sub-Investigator	
	Ivelina Andreeva	Sub-Investigator	
	Grisha Mateev	Principal Investigator	DCC Focus 5 - MEOH OOD, Sofia, Bulgaria, 1463
	Valeria Mateeva	Sub-Investigator	
	Radoslav Petrov	Sub-Investigator	
	Ivan Botev	Principal Investigator	Ambulatory For Specialized Medical Help - Skin And Venereal Diseases, Sofia, Bulgaria, 1407
	Iveta Dencheva	Sub-Investigator	
	Vessel Kantardjiev	Principal Investigator	Military Medical Academy - MHAT - Sofia, Sofia, Bulgaria, 1606
	Stanko Klisarski	Sub-Investigator	
	Tsvetomila Vuteva	Sub-Investigator	
Canada	Lorne Albrecht	Principal Investigator	Enverus Medical Research, Surrey, Canada, V3V 0C6
	Bez Toosi	Sub-Investigator	
	Soodabeh Zandi	Sub-Investigator	
	Susan Poelman	Principal Investigator	Beacon Dermatology, Calgary, Canada, T3E 0B2
	Andrei Metelitsa	Sub-Investigator	
	Kamal Ohson	Principal Investigator	Karma Clinical Trials Inc., St. John's, Canada, A1A 4Y3
	Joshua Mercer	Sub-Investigator	
	Tim Strand	Sub-Investigator	
	Jaggi Rao	Principal Investigator	Alberta DermaSurgery Centre, Edmonton, Canada, T6G 1C3
	Tiffany Kwok	Sub-Investigator	
	Irina Turchin	Principal Investigator	Brunswick Dermatology Center, Fredericton, Canada, E3B 1G9
	Irene Vasiliu	Sub-Investigator	
	Angelique Gagne-Henley	Principal Investigator	Dre Angelique Gagne-Henley M.D. Inc., St-Jérôme, Canada, J7Z 7E2
	Roula El Rassi	Sub-Investigator	
	Anne Sophie Smilga	Sub-Investigator	
	Raed Alhusayen	Principal Investigator	

	Faisal Al-Mohhamedi	Sub-Investigator	DermCare Clinic, Mississauga, Canada, L4W 0C2
	Michael Cecchini	Principal Investigator	York Dermatology Center, Richmond Hill, Canada, L4B 1L1
	Patrick Fleming	Sub-Investigator	
Germany	Sascha Gerdes	Principal Investigator	Universitatsklinikum Schleswig Holstein, Kiel, Germany, 24105
	Juliane Reckert	Sub-Investigator	
	Max Rogatsch	Sub-Investigator	
	Elisabeth Schuldt	Sub-Investigator	
	Dora Stoelzl	Sub-Investigator	
	Julia Suttorp	Sub-Investigator	
	Stephan Weidinger	Sub-Investigator	
	Elena Luisa Bauer	Sub-Investigator	
	Christos Zouboulis*	Principal Investigator/Sub-Investigator	Staedtisches Klinikum Dessau Brandenburg Medical School Theodor Fontane, Dessau-Rosslau, Germany, 06847
	Georgios Nikolakis*	Previous Principal Investigator/Sub-Investigator	
	Daifallah Almansouri	Sub-Investigator	
	Abdulaziz Alzahrani	Sub-Investigator	
	Katja Kreibich	Sub-Investigator	
	Karolina Reiprich	Sub-Investigator	
	Georgia Vradeli	Sub-Investigator	
	Sonja Molin*	Principal Investigator/Sub-Investigator	Charite Universitaetsmedizin Berlin - Campus Charite Mitte, Berlin, Germany, 10117
	Georgios Kokolakis	Previous Principal Investigator	
	Kamran Ghoreschi	Sub-Investigator	
	Tereza Jakovljevicova	Sub-Investigator	
	Torben Krause	Sub-Investigator	
	Marie Luise Lrmer	Sub-Investigator	
	Michael Zolotas	Sub-Investigator	
	Falk G. Bechara	Principal Investigator	St. Josef-Hospital Universitaetsklinikum, Bochum, Germany, 44791
	Yannik Haven	Sub-Investigator	
	Maria Iordanou	Sub-Investigator	
	Christina Scheel	Sub-Investigator	
	Caroline Marie Seifert	Sub-Investigator	
	Eggert Stockfleth	Sub-Investigator	
	Diamant Thaci	Principal Investigator	Universitatsklinik Schleswig-Holstein Campus Lubeck, Luebeck, Germany, 23538
	Julia Guenther	Sub-Investigator	
	Tomasz Hawro	Sub-Investigator	
	Ralf Ludwig	Sub-Investigator	
	Lena Lueth	Sub-Investigator	

	Cristian Papara	Sub-Investigator	
	Elisabeth Spallek	Sub-Investigator	
	Sascha Staender	Sub-Investigator	
	David Aldo De Luca Laredo	Sub-Investigator	
	Knut Schaekel	Principal Investigator	Universitaetsklinikum Heidelberg, Heidelberg, Germany, 69120
	Silvia Mihalceanu	Sub-Investigator	
	Niklas Negele	Sub-Investigator	
	Patryk Porebski	Sub-Investigator	
	Martin Salzmann	Sub-Investigator	
	Franziska Keidel	Sub-Investigator	
	Katharina Seel	Sub-Investigator	
	Sebastian Zimmer	Principal Investigator	Hautmedizin Saar, Merzig, Germany, 66663
	Sophia Zimmer	Sub-Investigator	
	Michael Sticherling	Principal Investigator	Universitatsklinikum Erlangen Hautklinik, Erlangen, Germany, 91054
	Franz Heppt	Sub-Investigator	
	Jennifer Lermer (Née Groetsch)	Sub-Investigator	
	Laura Rechtien	Sub-Investigator	
	Moritz Ronicke	Sub-Investigator	
	Ioannis Sagonas	Sub-Investigator	
	Lukas Sollfrank	Sub-Investigator	
	Dorothee Busch	Sub-Investigator	
	Martin Zeschick	Sub-Investigator	
	Christian Kromer	Principal Investigator	Universitatsmedizin Goettingen, Goettingen, Germany, 37075
	Sara Isik	Sub-Investigator	
	Anike Lockmann	Sub-Investigator	
	Patryk Porebski	Sub-Investigator	
	Marie Charlotte Schuppe	Sub-Investigator	
	Hedwig Stanisz Bogeski	Sub-Investigator	
Denmark	Mads Rasmussen	Principal Investigator	Aarhus University Hospital, Århus N, Denmark, 8200
	Trine Bertelsen	Sub-Investigator	
	Line Kibsgaard	Sub-Investigator	
	Hans Christian Ring*	Principal Investigator/Sub-Investigator	Sjaellands Universitetshospital, Roskilde, Denmark, 4000
	Elisabeth Rygvold Haugsten	Sub-Investigator	
	Ditte Saunte	Sub-Investigator	
	Peter Theut Riis	Sub-Investigator	
	Gregor Jemec	Previous Principal Investigator	
Spain	Antonio Martorell	Principal Investigator	Hospital De Manis, Manises, Spain, 46940
	Luis Hueso Gabriel	Sub-Investigator	
	Maria Luisa Garcia Melgares Linares	Sub-Investigator	
	Gemma Ochando Ibernon	Sub-Investigator	
	Jose Manuel Rueda Carnero	Sub-Investigator	

	Alberto Alfaro Rubio	Sub-Investigator	
	Maria Cristina Ciudad Blanco	Principal Investigator	Hospital General Universitario Gregorio Maranon, Madrid, Spain, 28009
	Ofelia Baniandres Rodriguez	Sub-Investigator	
	Jorge Martin Nieto Gonzalez	Sub-Investigator	
	Belen Rodriguez Sanchez	Sub-Investigator	
	Elena Castro Gonzalez	Principal Investigator	Hospital Universitario De Gran Canaria Doctor Negrin, Las Palmas De Gran Carania, Spain, 35010
	Irene Castano Gonzalez	Sub-Investigator	
	Natalia Naranjo Guerrero	Sub-Investigator	
	Gregorio Carretero Hernandez	Previous Investigator	
	Jose Carlos Pascual Ramirez	Principal Investigator	Hospital General Unviersitario De Alicante, Alicante, Spain, 03010
	Jose Carrasco Munoz	Sub-Investigator	
	Iris Gonzalez Villanueva	Sub-Investigator	
	Yolanda Delgado Jimenez	Principal Investigator	Hospital Universitario De La Princesa, Madrid, Spain, 28005
	Mireia Segui Olmedilla	Sub-Investigator	
	Daniella Paz Cullen Aravena	Principal Investigator	Fundacion Jimenez Diaz University Hospital, Madrid, Spain, 28003
	Maria Del Carmen Farina Sabaris	Sub-Investigator	
	Irene Hoyas Rodriguez	Sub-Investigator	
	David Revilla Nebreda	Sub-Investigator	
	Elisa Rios Vinuela	Sub-Investigator	
France	Pierre-Andre Becherel	Principal Investigator	Hospital Prive d'Antony, Antony, France, 92160
	Marina Thomas	Sub-Investigator	
	Thierry Boye	Principal Investigator	Hopital Sainte Anne (H.I.A), Toulon, France, 83000
	Safia Abed	Sub-Investigator	
	Aude Valois	Sub-Investigator	
	Anne-Benedicte Duval-Modeste	Principal Investigator	Hopital Charles Nicolle Chu Rouen Hospital De Bois-Guillaume, Rouen Cedex, France, 76031
	Claire Mignard	Sub-Investigator	
	Bilal Tedbirt	Sub-Investigator	
	Raphael Janela Lapert	Sub-Investigator	
	Thierry Passeron	Principal Investigator	CHU De Nice - Hospital L Archet, Nice Cedex 3, France, 06200
	Stephany Marie Luce	Sub-Investigator	
	Pierre-Michel Dugourd	Sub-Investigator	
	Axel Villani	Principal Investigator	Hopital Edouard Herrio, Lyon Cedex 03, France, 69437
	Ines Chircop	Sub-Investigator	
	Marie Danset	Sub-Investigator	
	Denis Jullien	Previous Investigator	
	Claire Thibaud	Sub-Investigator	
	Marine Chastagner	Sub-Investigator	
	Christophe Bedane	Principal Investigator	CHU Dijon-Bourgogne, Dijon, France, 21000
	Eleonore Goubeau	Sub-Investigator	
	Geraldine Jeudy	Sub-Investigator	
	Cécile Juzot	Sub-Investigator	
	Camille Leleu	Sub-Investigator	

	Ziad Reguiai	Principal Investigator	Polyclinique De Courlancy, Reims, France, 51100
	Fabienne Leonard	Sub-Investigator	
United Kingdom	Evmorfia Ladoyanni	Principal Investigator	Russells Hall Hospital, Dudley, United Kingdom, DY1 2HQ
	Kandace Baggan	Sub-Investigator	
	Dinushani Bandara	Sub-Investigator	
	Farishta Khan	Sub-Investigator	
	Maria Ahmad	Sub-Investigator	
	Elizabeth Peterknecht	Sub-Investigator	
	Kave Shams	Principal Investigator	Leeds General Infirmary, Leeds, United Kingdom, LS7 4SA
	Laura Savage	Sub-Investigator	
	Ser Ling Chua*	Principal Investigator/Sub-Investigator	Queen Elizabeth Hospital, Birmingham, United Kingdom, B15 2TH
	Richard Barlow	Sub-Investigator	
	Ser Ling Chua	Sub-Investigator	
	Fatima Dalal	Sub-Investigator	
	Agustin Martin-Clavijo	Previous Principal Investigator	
	Lauren Passby	Sub-Investigator	
	Naser Qasem	Sub-Investigator	
	Neenu Sebastian	Sub-Investigator	
	Emma McMullen	Principal Investigator	Salford Care Organisation, Salford, United Kingdom, M6 8HD
	Omar Chircop	Sub-Investigator	
	Neenu Sebastian	Sub-Investigator	
	Elham Rashidghamat	Previous Investigator	St John's Institute Of Dermatology, London, United Kingdom, SE1 9RT
	Mia Steyn*	Previous Principal Investigator/Sub-Investigator	
	Manpreet Lakhan	Sub-Investigator	
Italy	Oriana Simonetti*	Principal Investigator/Sub-Investigator	Azienda Ospedaliero Universitaria Delle Marche - Ancona, Ancona, Italy, 60126
	Elisa Molinelli*	Previous Principal Investigator/Sub-Investigator	
	Anna Maria Offidani	Previous Principal Investigator	
	Andrea Sebastianelli	Sub-Investigator	
	Cecilia Busignani	Sub-Investigator	
	Edoardo De Simoni	Sub-Investigator	
	Davide Febi	Sub-Investigator	
	Helena Gioacchini	Sub-Investigator	
	Antonio Costanzo	Principal Investigator	IRCCS Istituto Clinico Humanitas, Rozzano, Italy, 20089
	Ruggero Cascio Ingurgio	Sub-Investigator	
	Luigi Gargiulo	Sub-Investigator	

	Alessandra Narcisi	Sub-Investigator	
	Mario Valenti	Sub-Investigator	
	Angelo Valerio Marzano	Principal Investigator	Fondazione IRCCS Ca Granda Ospedale Maggiore, Milano, Italy, 20122
	Giovanni Genovese	Sub-Investigator	
	Carlo Maronese	Sub-Investigator	
	Giulia Marruzzo	Sub-Investigator	
	Federica Derlino	Sub-Investigator	
	Luca Valtellini	Sub-Investigator	
	Massimiliano Scalvenzi	Principal Investigator	Azienda Ospedaliera Universitaria Federico II, Naples, Italy, 80131
	Gabriella Fabbrocini	Previous Principal Investigator	
	Luigi Guerriero	Sub-Investigator	
	Fabrizio Martora	Sub-Investigator	
	Megna Matteo	Sub-Investigator	
	Luca Potestio	Sub-Investigator	
	Flavia Raia	Sub-Investigator	
	Valeria Esposito	Sub-Investigator	
	Piergiacomo Calzavara Pinton	Principal Investigator	Azienda Socio Sanitaria Territoriale Degli Spedali Civili Di Brescia (Presidio Spedali Civili), Brescia, Italy, 25124
	Carola Romano	Sub-Investigator	
	Sara Rovaris	Sub-Investigator	
	Marina Venturini	Sub-Investigator	
	Simone Viola	Sub-Investigator	
	Ketty Peris	Principal Investigator	Fondazione Policlinico Universitario Agostino Gemelli IRCCS, Roma, Italy, 00136
	Maria Vittoria Cannizzaro	Sub-Investigator	
	Andrea Chiricozzi	Sub-Investigator	
	Dalma Malvaso	Sub-Investigator	
	Flaminia Antonelli	Sub-Investigator	
	Marco Romanelli	Principal Investigator	Azienda Ospedaliero Universitaria Pisana - Ospedale S. Chiara, Pisa, Italy, 56126
	Ferdinando De Negri	Sub-Investigator	
	Valentina Dini	Sub-Investigator	
	Elisabetta Fini	Sub-Investigator	
	Giovanni Gori	Sub-Investigator	
	Giammarco Granieri	Sub-Investigator	
	Flavia Margiotta	Sub-Investigator	
	Alessandra Michelucci	Sub-Investigator	
	Alice Cangialosi	Sub-Investigator	
	Giuseppe Micali	Principal Investigator	Azienda Ospedaliero Universitaria Policlinico G.Rodolico San Marco, Catania, Italy, 95123
	Federica Finazzo	Sub-Investigator	
	Giuliana Caruso	Sub-Investigator	
	Maria Letizia Musumeci	Sub-Investigator	
	Sabrina Zappulla	Sub-Investigator	
Poland	Adam Reich	Principal Investigator	Uniwersytecki Szpital Kliniczny im. Fryderyka Chopina W Rzeszowie, Rzeszow, Poland, 35-055
	Justyna Szczech	Sub-Investigator	
	Gabriela Zuk	Sub-Investigator	
	Dominik Samotij	Sub-Investigator	

	Irena Walecka Herniczek	Principal Investigator	Panstwowy Instytut Medyczny Ministerstwa Spraw Wewnetrznych Administracji, Warszawa, Poland, 02-507
	Anna Czaplicka	Sub-Investigator	
	Ewa Czaplicka	Sub-Investigator	
	Elzbieta Szymanska	Sub-Investigator	
	Piotr Ciechanowicz	Sub-Investigator	
	Jan Wolak	Sub-Investigator	
	Jerzy Wolak	Sub-Investigator	
	Natalia Zajac	Sub-Investigator	
	Witold Owczarek	Principal Investigator	Royalderm, Warszawa, Poland, 02-962
	Anna Kozera	Sub-Investigator	
	Agnieszka Nawrocka	Sub-Investigator	
	Elwira Paluchowska	Sub-Investigator	
	Iwona Czarnecka	Sub-Investigator	
	Dorota Krasowska	Principal Investigator	Luxderm Specjalistyczny Gabinet Dermatologiczny, Lublin, Poland, 20573
	Michal Adamczyk	Sub-Investigator	
	Danuta Krasowska	Sub-Investigator	
United States	Ashley Cauthen	Principal Investigator	Renstar Medical Research Ocala, Ocala, USA, 34470
	Jessin Blossom	Sub-Investigator	
	Sidney Clevinger	Sub-Investigator	
	Jordan Griffis	Sub-Investigator	
	Natasha Maguire	Sub-Investigator	
	Sierra Mahoney	Sub-Investigator	
	Sierra Rebchook	Sub-Investigator	
	Seth Forman	Principal Investigator	Forcare Clinical Research, Tampa, USA, 33613
	Casey Viera	Sub-Investigator	
	Deborah White	Sub-Investigator	
	Tanya Bogle	Sub-Investigator	
	Maria Hicks	Sub-Investigator	
	Saakshi Khattri	Principal Investigator	Mount Sinai Doctors, New York, USA, 10028
	Emma Guttman-Yassky	Previous Principal Investigator	
	Hassan Hamade	Sub-Investigator	
	Madeleine Degrange	Sub-Investigator	
	Anusha Pasumarthi	Sub-Investigator	
	Hailey Pfeifer	Sub-Investigator	
	Jessica Kaffenberger	Principal Investigator	Ohio State University Wexner Medical Center, Columbus, USA, 43210
	Bianka Bubic	Sub-Investigator	
	Rachel Kirven	Sub-Investigator	
	Eleonora Marcacci	Sub-Investigator	
	Morgan Rousseau	Sub-Investigator	
	Hadar Lev-Tov	Principal Investigator	University of Miami Miller School of Medicine, Miami, USA, 33125
	Jorge Carrillo	Sub-Investigator	
	Gabriela Beraja	Sub-Investigator	
	Katrina Munoz	Sub-Investigator	
	Olumide Ojoola	Sub-Investigator	
	Marita Yaghi	Sub-Investigator	

	Christopher Sayed	Principal Investigator	UNC Dermatology And Skin Cancer Center At Southern Village, Chapel Hill, USA, 27516
	Aida Lugo	Sub-Investigator	
	Matthew Lipner	Sub-Investigator	
	Kenneth Dawes	Principal Investigator	Dawes Fretzin Clinical Research Group LLC, Indianapolis, USA, 46250
	Jennifer Conner	Sub-Investigator	
	Scott Fretzin	Sub-Investigator	
	Elizabeth Golden	Sub-Investigator	
	Michael Isaacs	Sub-Investigator	
	Courtney Keasey	Sub-Investigator	
	Presley Pearman	Sub-Investigator	
	Christina Race	Sub-Investigator	
	Robert Koppel	Principal Investigator	Clinical Trials Management LLC, Metairie, USA, 70006
	Douglas Koppel	Sub-Investigator	
	Rebecca Lee Delahoussaye Soine	Sub-Investigator	
	David Greenstein	Principal Investigator	Allcutis Research Inc Beverly, Beverly, USA, 01915
	Nicole Branum	Sub-Investigator	
	Debra Litke	Sub-Investigator	
	Michael McCartney	Sub-Investigator	
	Cindy Owen	Principal Investigator	Dermatologist Specialist, Louisville, USA, 40241
	Jyoti Burruss	Sub-Investigator	
	Anna De Benedetto	Principal Investigator	UR Dermatology At College Town, Rochester, USA, 14620
	Lauren Baughman	Sub-Investigator	
	Kristin Calley	Sub-Investigator	
	Cameron Clements	Sub-Investigator	
	Andrea Alexander	Sub-Investigator	
	David Field	Sub-Investigator	
	Leah Laageide	Sub-Investigator	
	Alison Moy	Sub-Investigator	
	Stacey Swinehart	Sub-Investigator	
	Kathryn Wisnowski	Sub-Investigator	
	Renato Dominguez	Principal Investigator	Nova Research Institute LLC, Miami, USA, 33125
	Amado Viera	Previous Investigator	
	Noah Goldfarb	Principal Investigator	University of Minnesota, Minneapolis, USA, 55455
	David Pearson	Sub-Investigator	
	Fasahat Hamzavi	Principal Investigator	Hamzavi Dermatology, Fort Gratiot, USA, 48059
	Iltefat Hamzavi*	Previous Principal Investigator/Sub-Investigator	
	Bryan Sofen	Sub-Investigator	
	Cheryl Hull	Principal Investigator	Northwest Arkansas Clinical Trials Center, Rogers, USA, 72758
	Hanna Cox	Sub-Investigator	
	Matthew Zirwas	Principal Investigator	

	Sherri Howard	Sub-Investigator	Bexley Dermatology Research, Bexley, USA, 43209
	Cory Rubin	Principal Investigator	Michigan Dermatology Institute, Waterford, USA, 48328
	Julie Pfeifle	Sub-Investigator	
	Aimee Adams	Sub-Investigator	
	Kailey Temple	Sub-Investigator	
	Craig Teller	Principal Investigator	Bellaire Dermatology Associates BDA, Bellaire, USA, 77401
	Shelby Bonn	Sub-Investigator	
	Courtney Brown	Sub-Investigator	
	Casey Wang	Sub-Investigator	
	Paul Getz	Principal Investigator	Dundee Dermatology, West Dundee, USA, 60118
	Bonnie Barsky	Sub-Investigator	
	Victoria Kuohung	Principal Investigator	Beacon Clinical Research BCR, LLC, Quincy, USA, 02169
	Candace Broussard	Sub-Investigator	
	Zachary Gross	Sub-Investigator	
	Paradi Mirmirani	Principal Investigator	Kaiser Permanente, Oakland, USA, 94612
	Barbara Garcia	Sub-Investigator	
	Christine C Jacobson	Sub-Investigator	
	Katz Kenneth	Sub-Investigator	
	Bhavnit Bhatia	Sub-Investigator	
	Natalie Viakhireva	Sub-Investigator	
	Akhil Wadhera	Sub-Investigator	
	Natalie E. Wong	Sub-Investigator	
	Jennifer Hsiao	Principal Investigator	USC Keck School of Medicine, Los Angeles, USA, 90033
	Ashley Crew	Sub-Investigator	
	Melissa Lee	Sub-Investigator	
	Luis Garza	Principal Investigator	John Hopkins University-School of Medicine, Baltimore, USA, 21287
	Leigh Curvin Aquilla	Sub-Investigator	
	Ginette Okoye	Previous Investigator	
	Dinah Price	Sub-Investigator	
	Stacey Swinehart	Sub-Investigator	
	Alexandra Charrow	Principal Investigator	Brigham And Women's Hospital, Boston, USA, 02115
	Stephanie Cohen	Sub-Investigator	
	Joseph Merola	Sub-Investigator	
	Arash Mostaghimi	Sub-Investigator	
	Michelle Pelle	Principal Investigator	Medderm Associates, Inc, San Diego, USA, 92103
	Michael Lee	Sub-Investigator	
	Brad Glick	Principal Investigator	GSI Clinical Research, Margate, USA, 33063
	Allan Snyder	Sub-Investigator	
	Stephen Lockwood*	Principal Investigator/Sub-Investigator	TrueBlue Clinical Research Moore Clinical Research, Inc MCR Tampa Clinic Location, Tampa, USA, 33609
	Michael Tharp*	Previous Principal Investigator	
	Rosemarie Acuna	Sub-Investigator	

	Sergio Aguilar	Sub-Investigator	
	Lisabeth Bustos	Sub-Investigator	
	Alexander Hervey	Sub-Investigator	
	Latia Jackson	Sub-Investigator	
	Elizabeth Juneja	Sub-Investigator	
	Theresa Lopez Delfi	Sub-Investigator	
	Julian Melamed	Sub-Investigator	
	Scott Miller	Sub-Investigator	
	George Monlux Jr	Sub-Investigator	
	Michelle Orzechowski	Sub-Investigator	
	Xairyl Ruiz-Rivera	Sub-Investigator	
	Temitope Soares	Sub-Investigator	
	Sarah Jackson	Principal Investigator	Delricht Research, New Orleans, USA, 70115
	Deirdre Hooper	Sub-Investigator	
	Patrick Dennis	Sub-Investigator	
	Jerry Bagel	Principal Investigator	Windsor Dermatology, East Windsor, USA, 08520
	Brianna Butler	Sub-Investigator	
	Alexa Hetzel	Sub-Investigator	
	David Nieves	Sub-Investigator	
	Kristen Teixeira	Sub-Investigator	
	Christina Feser	Principal Investigator	International Clinical Research Tennessee LLC, Murfreesboro, USA, 37130
	David Feser	Sub-Investigator	
	Matthew Middler	Sub-Investigator	
	Frances Miller	Sub-Investigator	
	Ronald Nelson	Sub-Investigator	
	Minity Shukla	Sub-Investigator	
	Mark Lee	Principal Investigator	Progressive Clinical Research, San Antonio, USA, 78213
	Theresia Lee	Sub-Investigator	
	Angela Moore	Principal Investigator	Arlington Research Center, Arlington, USA, 76011
	Todd Moore	Sub-Investigator	
	Giovanna Ciocca	Principal Investigator	Skin Research of South Florida, LLC, Miami, USA, 33173
	Marilyn Fleites	Sub-Investigator	
	Aaron Mangold	Principal Investigator	Mayo Clinic-Scottsdale, Phoenix, USA, 85054
	Xiang Chen	Sub-Investigator	
	Samantha Zunich	Sub-Investigator	
	Edward Lain	Principal Investigator	Austin Institute For Clinical Research AICR Pflugerville, Pflugerville, USA, 78660
	Jennifer Vickers	Sub-Investigator	
	Joel Schlessinger	Principal Investigator	Skin Specialists PC The Advanced Skin Research Center, Omaha, USA, 68144
	Jacqueline Gaffney	Sub-Investigator	
	Shea Perillo	Sub-Investigator	
	Daniel Schlessinger	Sub-Investigator	
	Karan Lal	Principal Investigator	Affiliated Dermatology, Scottsdale, USA, 85255
	Christine Johnson	Sub-Investigator	

	Megan Maley	Sub-Investigator	
	Rebekah Baltz	Principal Investigator	Premier Dermatology Clinical Trials Institute At Northwest Arkansas, Fayetteville, USA, 72703
	Cheri Cunningham	Sub-Investigator	
	Mimi Hill	Sub-Investigator	
	Teresa Mckinney	Sub-Investigator	
	Kara Richardson	Sub-Investigator	

* Investigators with more than one role due to changes in study responsibilities at the site.

STOP-HS1 Institutional Review Boards and/or Ethics Committees

Country	IRB or Ethics Committee
United States	Advarra IRB<br>Columbia, MD 21044 USA
	NorthShore University Health Systems IRB<br>Evanston, IL 60201 USA
Japan	University Hospital, Kyoto Prefectural<br>University of Medicine IRB<br>Kyoto-Fu 602-8566 Japan
	University of the Ryukyus Hospital IRB<br>Okinawa-Ken 901-2725 Japan
	Tohoku University Hospital IRB<br>Miyagi-Ken 980-8574 Japan
	Niigata University Medical & Dental Hospital IRB<br>Niigata-Ken 951-8520 Japan
	Nihon University Hospitals' Joint IRB<br>Tokyo-To 173-8610 Japan
	Yokohama Minoru Clinic IRB<br>Kanagawa-Ken 232-0064 Japan
	Hokkaido University Hospital IRB<br>Hokkaido 060-8648 Japan
	Tokyo Medical University Hospital IRB<br>Tokyo-To 160-0023 Japan
France	CPP Sud-Est IV<br>Centre Léon Bérard<br>69373 Lyon CEDEX 08 France
Germany	Ethik-Kommission der Bayerischen Landesärztekammer<br>D-81677 München, Germany
Canada	Committee for Harmonized Health<br>Impact, Privacy, and Ethics Review<br>(CHIPER) - Biomedical Ethics Board<br>Winnipeg, MB R3E 0T6 Canada
	Advarra Canada<br>Aurora, ON L4G 0A5 Canada
Spain	CEIm Hospital General Universitario<br>Gregorio Marañón<br>28007 Madrid, Spain
Belgium	Not disclosed
Poland	Naczelna Komisja Bioetyczna<br>00-801 Warszawa, Poland
Austria	Johannes Kepler University Linz<br>MED Ethics Commission<br>4020 Linz, Austria
Greece	National Ethics Committee<br>115 62 Holargos, Greece
Netherlands	MREC UMC Groningen

	9700 RB Groningen, Netherlands
Czech Republic	Eticka komise SUKL<br>100 41 Praha 10, Czech Republic

STOP-HS2 Institutional Review Boards and/or Ethics Committees

**Country**	**IRB or Ethics Committee**
United States	Advarra IRB<br>Columbia, MD 21044 USA
	University of Miami Human Subject<br>Research Office IRB<br>Coral Gables, FL 33146 USA
	WCG<br>Puyallup, WA 98374 USA
	The Johns Hopkins Medical School IRB<br>Baltimore, MD 21205-1911 USA
	Mayo Clinic Institutional Review Board<br>Rochester, MN 55905 USA
Germany	Ethik-Kommission der Bayerischen Landesärztekammer<br>D-81677 München, Germany
Canada	Advarra Canada<br>Aurora, ON L4G 0A5 Canada
	Health Research Ethics Board of Alberta (HREBA-CTC)<br>Edmonton, AB T5J 0H8 Canada
	Health Research Ethics Authority<br>St John's, NL A1L 2X5 Canada
	Horizon Health Network Research Ethics Board<br>Saint John, NB E2L 4L2 Canada
Italy	CET Lazio Area 5<br>00144 Roma, Italy
Australia	South Western Sydney Local Health District (RGO)<br>Liverpool NSW 1871, Australia
	South Western Sydney Local Health District HREC<br>Warwick Farm NSW 2170, Australia
	Bellberry Human Research Ethics Committees<br>Dulwich SA 5065, Australia
	Sinclair Dermatology RGO<br>East Melbourne VIC 3002, Australia
	Metro South Health Service District<br>Human Research Ethics Committee (RGO)<br>Centres for Health Research<br>Princess Alexandra Hospital, Ipswich Road<br>Woolloongabba QLD 4102, Australia
France	CPP Sud-Est IV<br>Centre Léon Bérard<br>69373 Lyon CEDEX 08, France

Bulgaria	Ethics Committee for Clinical Trials<br>1303 Sofia, Bulgaria
Spain	CEIm Hospital General Universitario<br>Gregorio Marañón<br>28007 Madrid, Spain
United Kingdom	Dudley Group NHS Foundation Trust<br>Dudley DY1 2HQ, United Kingdom
	Wales REC5<br>Cardiff CF11 9AB, United Kingdom
	Leeds Teaching Hospitals NHS Trust<br>Leeds LS2 9LN, United Kingdom
	University Hospitals Birmingham NHS Foundation Trust<br>Birmingham B15 2TT, United Kingdom
	Northern Care Alliance NHS Foundation Trust<br>Salford M55AP, United Kingdom
	Guy's and St Thomas' NHS Foundation Trust<br>London SE1 9RT, United Kingdom
Poland	Naczelna Komisja Bioetyczna<br>00-801 Warszawa, Poland
Denmark	The Danish National Committee on Health Research Ethics (NVK)<br>and Medical Research Ethics Committees (MREC)<br>2300 København S, Denmark

.....

Table S1. STOP-HS1 and STOP-HS2 Pooled Baseline Demographics and Disease Characteristics by Country or Geographic Region

Characteristic	USA (n=501)	Canada (n=137)	Europe (n=533)	Australia (n=34)	Japan (n=22)
Age, years	36.9 (11.0)	38.9 (11.9)	37.5 (11.7)	32.4 (10.8)	38.1 (13.5)
Sex					
Female	374 (74.7)	97 (70.8)	269 (50.5)	22 (64.7)	8 (36.4)
Male	127 (25.3)	40 (29.2)	264 (49.5)	12 (35.3)	14 (64.6)
Body weight, kg	105.3 (27.9)	104.8 (25.9)	92.0 (21.4)	99.8 (32.2)	78.2 (19.2)
BMI, kg/m^2	36.9 (9.1)	37.0 (8.9)	30.7 (6.5)	34.5 (10.4)	28.1 (7.3)
Smoking status					
Current	160 (32%)	69 (50%)	327 (61%)	12 (35%)	12 (55%)
Former	86 (17%)	28 (20%)	85 (16%)	6 (18%)	3 (14%)
Race					
White	303 (61%)	113 (83%)	475 (89%)	24 (71%)	0
Black	164 (33%)	4 (3%)	5 (1%)	1 (3%)	0
Asian	8 (2%)	6 (4%)	6 (1%)	2 (6%)	22 (100%)
Previous use of biologic therapy					
Any biologic	196 (39%)	41 (30%)	208 (39%)	9 (27%)	3 (14%)
TNF-α inhibitor	171 (34%)	32 (23%)	151 (28%)	8 (24%)	2 (9%)
IL-17 inhibitor	54 (11%)	9 (7%)	109 (21%)	2 (6%)	1 (5%)
Other biologic	20 (4%)	5 (4%)	31 (6%)	2 (6%)	0
Prior HS surgery					
Any HS surgery	199 (40%)	69 (50%)	264 (50%)	16 (47%)	8 (36%)
Incision and drainage	119 (24%)	42 (31%)	123 (23%)	7 (21%)	6 (27%)
Local excision	73 (15%)	30 (22%)	144 (27%)	6 (18%)	3 (14%)
Deroofing	18 (4%)	12 (9%)	38 (7%)	4 (12%)	0
Disease duration, years	11.2 (0.5–53.3)	11.2 (0.3–62.6)	9.6 (0.4–57.7)	8.2 (0.5–49.1)	1.8 (0.3–20.8)

HS family history	412 (82%)	117 (85%)	271 (51%)	23 (68%)	17 (77%)
Abscess and inflammatory nodule count	13.2 (9.7)	11.5 (6.5)	10.8 (7.8)	14.6 (14.5)	14.6 (7.4)
Abscess count	2.5 (4.2)	2.5 (3.2)	1.8 (3.1)	1.0 (1.5)	5.0 (4.3)
Inflammatory nodule count	10.7 (8.2)	9.0 (5.9)	9.0 (7.1)	13.6 (14.5)	9.5 (5.9)
Draining tunnel count	2.3 (3.0)	3.2 (3.3)	3.2 (3.4)	1.6 (3.1)	4.9 (5.2)
≥1 draining tunnel	334 (67%)	118 (86%)	442 (83%)	16 (47%)	16 (73%)
Hurley Stage					
II	339 (68%)	79 (58%)	341 (64%)	26 (77%)	11 (50%)
III	162 (32%)	58 (42%)	192 (36%)	8 (24%)	11 (50%)
IHS4 score	24.9 (20.6)	26.7 (17.7)	25.3 (18.9)	21.9 (20.2)	39.1 (26.5)
hsCRP, mg/L	17.9 (25.9)	12.7 (16.1)	14.4 (24.8)	9.8 (14.4)	18.6 (26.1)
Skin Pain NRS	5.3 (2.5)	4.8 (2.2)	4.9 (2.6)	4.8 (2.4)	4.6 (2.5)
Skin Pain NRS score ≥3	396 (79%)	107 (78%)	396 (74%)	24 (71%)	16 (73%)
Itch NRS	4.8 (2.6)	4.3 (2.2)	4.2 (2.6)	4.3 (2.7)	3.6 (2.2)
HiSQoL total score	33.0 (14.5)	32.3 (14.7)	30.6 (14.9)	32.1 (12.5)	22.3 (11.3)
DLQI total score	13.9 (7.2)	13.0 (7.2)	12.3 (7.4)	13.0 (6.7)	6.2 (3.5)
FACIT-F score	30.7 (12.2)	29.8 (11.9)	31.9 (12.0)	27.5 (11.3)	42.5 (6.8)

Data are mean (range), mean (SD), or n (%). Data from STOP-HS1 and STOP-HS2 were pooled and summarized descriptively. Percentages might not add up to 100% due to rounding. Europe includes the countries that had active study sites: Austria, Belgium, Bulgaria, Czechia, Denmark, France, Germany, Greece, Italy, Netherlands, Poland, Spain, and United Kingdom. BMI=body mass index; DLQI=Dermatology Life Quality Index; FACIT-F=Functional Assessment of Chronic Illness Therapy–Fatigue; HiSQoL=Hidradenitis Suppurativa Quality of Life; HS=hidradenitis suppurativa; hsCRP=high-sensitivity C-reactive protein; IHS4=International Hidradenitis Suppurativa Severity Score System; IL=interleukin; NRS=numerical rating scale; TNF-α=tumour necrosis factor alpha.

Table S2. HiSCR50, HiSCR75, HiSCR90 and HiSCR100 Responses (OC and mNRI) at Weeks 12, 24, and 54

		STOP-HS1				STOP-HS2			
**Endpoint** (OC: % [n/N]; mNRI: % [SE])		**Povorcitinib 45 mg**	**Povorcitinib 75 mg**	**Placebo→ 45 mg**	**Placebo→ 75 mg**	**Povorcitinib 45 mg**	**Povorcitinib 75 mg**	**Placebo→ 45 mg**	**Placebo→ 75 mg**
**HiSCR50**									
Week 12	OC	43.9 (82/187)	44.8 (82/183)	32.4 (60/185)		47.6 (89/187)	47.1 (89/189)	31.7 (58/183)	
	mNRI	43.3 (3.6)	43.9 (3.6)	31.7 (3.4)		45.9 (3.5)	46.4 (3.6)	31.5 (3.4)	
Week 24	OC	52.6 (82/156)	50.0 (79/158)	64.0 (55/86)	62.7 (52/83)	57.1 (92/161)	57.9 (92/159)	58.0 (47/81)	56.3 (49/87)
	mNRI	48.0 (3.8)	47.5 (3.7)	62.4 (5.2)	61.1 (5.2)	54.4 (3.6)	52.0 (3.7)	59.9 (5.3)	53.4 (5.2)
Week 54	OC	60.2 (71/118)	61.9 (73/118)	68.3 (43/63)	68.1 (47/69)	67.5 (77/114)	57.3 (71/124)	64.3 (36/56)	71.4 (40/56)
	mNRI	48.6 (3.8)	50.7 (3.8)	57.4 (5.6)	60.3 (5.5)	52.9 (3.9)	49.9 (3.8)	55.4 (5.7)	51.0 (5.7)
**HiSCR75**									
Week 12	OC	22.5 (42/187)	26.8 (49/183)	17.3 (32/185)		28.9 (54/187)	31.2 (59/189)	14.8 (27/183)	
	mNRI	22.1 (3.0)	26.0 (3.2)	16.7 (2.7)		27.6 (3.2)	30.8 (3.3)	14.8 (2.6)	
Week 24	OC	37.2 (58/156)	34.2 (54/158)	36.0 (31/86)	39.8 (33/83)	37.3 (60/161)	40.3 (64/159)	38.3 (31/81)	31.0 (27/87)
	mNRI	33.9 (3.5)	32.7 (3.5)	36.6 (5.2)	37.6 (5.2)	34.9 (3.5)	36.9 (3.6)	39.3 (5.3)	29.3 (4.8)
Week 54	OC	39.0 (46/118)	47.5 (56/118)	47.6 (30/63)	53.6 (37/69)	57.0 (65/114)	46.8 (58/124)	48.2 (27/56)	44.6 (25/56)
	mNRI	31.3 (3.6)	37.4 (3.7)	40.1 (5.6)	45.1 (5.5)	42.4 (3.9)	40.2 (3.8)	38.7 (5.7)	33.8 (5.4)
**HiSCR90**									
Week 12	OC	8.6 (16/187)	15.3 (28/183)	9.2 (17/185)		16.6 (31/187)	14.8 (28/189)	9.3 (17/183)	
	mNRI	8.4 (2.0)	14.7 (2.6)	8.8 (2.0)		15.9 (2.6)	14.9 (2.6)	8.7 (2.0)	

Week 24	OC	23.7<br>(37/156)	20.3<br>(32/158)	24.4<br>(21/86)	27.7<br>(23/83)	24.8<br>(40/161)	25.8<br>(41/159)	22.2<br>(18/81)	13.8<br>(12/87)
	mNRI	21.2<br>(3.1)	18.6<br>(2.9)	24.5<br>(4.6)	25.9<br>(4.7)	22.4<br>(3.1)	23.2<br>(3.1)	22.3<br>(4.6)	13.4<br>(3.6)
Week 54	OC	23.7<br>(28/118)	28.8<br>(34/118)	23.8<br>(15/63)	36.2<br>(25/69)	34.2<br>(39/114)	29.8<br>(37/124)	26.8<br>(15/56)	23.2<br>(13/56)
	mNRI	18.7<br>(3.1)	21.6<br>(3.2)	20.7<br>(4.7)	30.2<br>(5.1)	24.1<br>(3.4)	23.9<br>(3.3)	18.8<br>(4.5)	16.5<br>(4.4)
**HiSCR100**									
Week 12	OC	7.5<br>(14/187)	10.4<br>(19/183)	6.5<br>(12/185)		13.4<br>(25/187)	13.2<br>(25/189)	8.7<br>(16/183)	
	mNRI	7.3<br>(1.9)	10.0<br>(2.2)	6.2<br>(1.8)		12.7<br>(2.4)	13.4<br>(2.5)	8.2<br>(2.0)	
Week 24	OC	21.2<br>(33/156)	14.6<br>(23/158)	19.8<br>(17/86)	20.5<br>(17/83)	19.9<br>(32/161)	19.5<br>(31/159)	21.0<br>(17/81)	9.2<br>(8/87)
	mNRI	18.4<br>(2.9)	13.1<br>(2.5)	20.1<br>(4.3)	19.2<br>(4.2)	17.8<br>(2.8)	18.0<br>(2.9)	20.3<br>(4.4)	9.0<br>(3.0)
Week 54	OC	19.5<br>(23/118)	22.0<br>(26/118)	19.0<br>(12/63)	29.0<br>(20/69)	27.2<br>(31/114)	21.0<br>(26/124)	21.4<br>(12/56)	17.9<br>(10/56)
	mNRI	14.6<br>(2.8)	16.3<br>(2.9)	16.5<br>(4.3)	23.4<br>(4.6)	17.8<br>(3.0)	17.6<br>(3.0)	15.5<br>(4.2)	12.8<br>(4.0)

Data are summarized descriptively, and the results of estimation and standard error were combined using Rubin's rule for mNRI. HiSCR=Hidradenitis Suppurativa Clinical Response; HiSCR50/75/90/100=≥50%/≥75%/≥90%/100% reduction from baseline in the total abscess and inflammatory nodule count, with no increase from baseline in abscess or draining tunnel count; mNRI=modified nonresponder imputation; OC=observed cases.

Table S3. STOP-HS1 and STOP-HS2 Pooled Maintenance of HiSCR50 and HiSCR75 During Extension Period in Patients Randomised to Povorcitinib (mNRI)

	Maintenance of HiSCR50, % (SE)		Maintenance of HiSCR75, % (SE)	
	Povorcitinib 45 mg (n=171)	Povorcitinib 75 mg (n=171)	Povorcitinib 45 mg (n=96)	Povorcitinib 75 mg (n=108)
Week 15	81.4 (3.1)	78.3 (3.2)	79.1 (4.3)	78.6 (4.0)
Week 18	79.3 (3.1)	79.3 (3.1)	75.9 (4.5)	75.4 (4.3)
Week 24	74.4 (3.5)	74.2 (3.4)	70.8 (5.0)	65.5 (4.7)
Week 30	66.9 (3.7)	74.8 (3.4)	61.3 (5.2)	60.1 (4.9)
Week 36	71.0 (3.7)	73.2 (3.5)	66.6 (5.0)	59.5 (4.9)
Week 42	67.7 (3.9)	69.8 (3.7)	68.4 (5.1)	58.7 (5.0)
Week 48	67.5 (4.0)	69.9 (3.5)	59.3 (5.6)	59.5 (4.8)
Week 54	66.3 (3.9)	69.3 (3.6)	58.5 (5.4)	61.3 (4.8)

Maintenance of response (HiSCR50/HiSCR75) was defined as patients who achieved HiSCR50 or HiSCR75 at Week 12 and maintained or achieved a greater response at each visit during the extension period; "n" represents the number of patients who achieved response at Week 12, out of a total of 369 and 371 for povorcitinib 45 mg and 75 mg, respectively. Data from STOP-HS1 and STOP-HS2 were pooled and summarized descriptively. The results of estimation and standard error were combined using Rubin's rule. HiSCR=Hidradenitis Suppurativa Clinical Response; HiSCR50/75=≥50%/≥75% reduction from baseline in the total abscess and inflammatory nodule count, with no increase from baseline in abscess or draining tunnel count; mNRI=modified nonresponder imputation.

Table S4. STOP-HS1 and STOP-HS2 Pooled Skin Pain NRS: Proportion of Patients in Each Categorical Score at Baseline and Weeks 12, 24, and 54 (mNRI)

Skin Pain NRS Category, % (SE)	Povorcitinib 45 mg*	Povorcitinib 75 mg*	Placebo→45 mg*	Placebo→75 mg*
**0 (no pain)**				
Baseline	2.5 (0.8)	1.3 (0.6)	3.0 (0.9)	
Week 12	6.9 (1.3)	7.6 (1.4)	5.3 (1.2)	
Week 24	11.3 (1.7)	11.8 (1.7)	10.8 (2.5)	11.9 (2.5)
Week 54	10.5 (1.7)	10.9 (1.7)	9.4 (2.3)	12.2 (2.8)
**>0–≤4 (mild)**				
Baseline	33.1 (2.3)	34.2 (2.4)	36.2 (2.4)	
Week 12	49.5 (2.6)	49.2 (2.6)	39.7 (2.5)	
Week 24	49.6 (2.7)	52.5 (2.7)	54.6 (3.9)	52.6 (3.8)
Week 54	52.5 (2.8)	53.0 (2.9)	57.4 (4.2)	55.6 (4.0)
**>4–≤7 (moderate)**				
Baseline	39.0 (2.4)	40.9 (2.5)	36.4 (2.4)	
Week 12	29.7 (2.4)	26.9 (2.3)	34.0 (2.5)	
Week 24	25.5 (2.4)	20.7 (2.3)	25.7 (3.4)	24.2 (3.3)
Week 54	21.9 (2.3)	19.7 (2.2)	19.5 (3.3)	20.8 (3.3)
**>7 (severe)**				
Baseline	25.4 (2.2)	23.7 (2.1)	24.3 (2.2)	
Week 12	13.9 (1.8)	16.3 (1.9)	21.0 (2.1)	
Week 24	13.7 (1.8)	15.0 (1.9)	9.0 (2.2)	11.3 (2.5)
Week 54	15.0 (2.0)	16.4 (2.1)	13.7 (3.0)	11.4 (2.5)

Data from STOP-HS1 and STOP-HS2 were pooled and summarized descriptively. The results of estimation and standard error were combined using Rubin's rule.
*Povorcitinib 45 mg, n=412; povorcitinib 75 mg, n=410; placebo (placebo-controlled period), n=405; placebo→45 mg, n=134; placebo→75 mg, n=134.
mNRI=modified nonresponder imputation; NRS=numerical rating scale.

Table S5. AN and dT Counts at Baseline and Weeks 3, 12, 24, and 54 (OC)

	STOP-HS1				STOP-HS2			
**Endpoint**	**Povorcitinib 45 mg**	**Povorcitinib 75 mg**	**Placebo→ 45 mg**	**Placebo→ 75 mg**	**Povorcitinib 45 mg**	**Povorcitinib 75 mg**	**Placebo→ 45 mg**	**Placebo→ 75 mg**
**AN count, mean (SD)**								
Baseline	13.0 (11.2)	12.1 (8.3)	12.3 (8.2)		11.6 (8.4)	12.1 (9.0)	11.1 (7.1)	
Week 3	10.0 (9.7)	8.8 (8.5)	10.3 (8.8)		8.2 (7.5)	8.6 (8.5)	9.3 (7.4)	
Week 12	7.3 (8.0)	7.9 (11.4)	9.0 (10.3)		6.0 (7.8)	6.9 (8.7)	8.3 (8.4)	
Week 24	6.2 (7.9)	5.0 (6.5)	5.8 (8.4)	5.2 (6.7)	4.6 (6.0)	4.7 (6.0)	4.2 (5.4)	4.9 (5.3)
Week 54	5.7 (7.9)	4.5 (6.9)	4.3 (5.5)	4.3 (7.0)	4.2 (6.6)	4.4 (6.0)	4.9 (9.8)	4.3 (6.3)
**dT count, mean (SD)**								
Baseline	3.0 (3.5)	2.8 (3.5)	2.9 (3.4)		2.7 (3.2)	2.5 (3.0)	3.0 (3.3)	
Week 3	2.4 (3.2)	2.0 (2.9)	2.7 (3.7)		2.2 (3.0)	1.8 (2.3)	3.0 (3.9)	
Week 12	2.1 (3.0)	1.8 (3.0)	2.6 (4.6)		1.7 (2.7)	1.5 (2.4)	2.6 (3.8)	
Week 24	2.0 (3.5)	1.4 (2.8)	1.6 (3.0)	1.4 (2.5)	1.5 (2.5)	1.2 (2.1)	1.5 (2.7)	2.2 (3.5)
Week 54	1.2 (2.2)	1.1 (2.2)	1.1 (2.1)	0.7 (1.3)	1.0 (2.1)	0.9 (1.8)	1.8 (3.6)	1.4 (2.6)

Data are summarized descriptively. AN=abscess and inflammatory nodule; dT=draining tunnel; OC=observed cases.

Table S6. STOP-HS1 and STOP-HS2 Pooled Lesion-Specific Response Rates at Weeks 12, 24, and 54 (OC)

A. Abscesses (A)

Endpoint, % (n/N)	Povorcitinib 45 mg	Povorcitinib 75 mg	Placebo→45 mg	Placebo→75 mg
**A75**				
**Baseline A count, 1–2**				
Week 12	62.6 (72/115)	56.1 (60/107)	51.9 (69/133)	
Week 24	58.5 (55/94)	57.6 (53/92)	70.0 (42/60)	57.6 (38/66)
Week 54	68.1 (47/69)	64.8 (46/71)	73.9 (34/46)	66.0 (31/47)
**Baseline A count, 3–5**				
Week 12	36.8 (25/68)	35.3 (18/51)	37.2 (16/43)	
Week 24	48.3 (28/58)	53.7 (22/41)	50.0 (11/22)	76.5 (13/17)
Week 54	50.0 (21/42)	58.1 (18/31)	64.7 (11/17)	75.0 (9/12)
**Baseline A count, ≥6**				
Week 12	43.6 (17/39)	43.6 (17/39)	25.0 (12/48)	
Week 24	66.7 (22/33)	66.7 (22/33)	59.1 (13/22)	45.5 (10/22)
Week 54	78.3 (18/23)	64.0 (16/25)	66.7 (10/15)	66.7 (10/15)
**A100**				
**Baseline A count, 1–2**				
Week 12	62.6 (72/115)	56.1 (60/107)	51.9 (69/133)	
Week 24	58.5 (55/94)	57.6 (53/92)	70.0 (42/60)	57.6 (38/66)
Week 54	68.1 (47/69)	64.8 (46/71)	73.9 (34/46)	66.0 (31/47)
**Baseline A count, 3–5**				
Week 12	26.5 (18/68)	25.5 (13/51)	23.3 (10/43)	
Week 24	41.4 (24/58)	48.8 (20/41)	45.5 (10/22)	47.1 (8/17)
Week 54	45.2 (19/42)	51.6 (16/31)	52.9 (9/17)	58.3 (7/12)
**Baseline A count, ≥6**				
Week 12	17.9 (7/39)	25.6 (10/39)	10.4 (5/48)	
Week 24	45.5 (15/33)	36.4 (12/33)	36.4 (8/22)	31.8 (7/22)
Week 54	47.8 (11/23)	40.0 (10/25)	33.3 (5/15)	46.7 (7/15)

B. Inflammatory Nodules (N)

Endpoint, % (n/N)	Povorcitinib 45 mg	Povorcitinib 75 mg	Placebo→45 mg	Placebo→75 mg
**N75**				
**Baseline N count, 1–5**				
Week 12	31.6 (31/98)	40.7 (35/86)	27.6 (24/87)	
Week 24	49.4 (41/83)	50.7 (38/75)	47.2 (17/36)	43.2 (19/44)
Week 54	60.3 (35/58)	51.6 (32/62)	41.4 (12/29)	64.3 (18/28)
**Baseline N count, 6–8**				
Week 12	35.0 (41/117)	39.7 (46/116)	25.8 (32/124)	
Week 24	55.6 (55/99)	57.0 (57/100)	43.8 (28/64)	46.9 (23/49)
Week 54	56.3 (45/80)	63.3 (50/79)	58.3 (28/48)	56.1 (23/41)
**Baseline N count, ≥9**				

Week 12	30.5 (47/154)	29.8 (50/168)	14.1 (22/156)	
Week 24	43.1 (56/130)	47.9 (67/140)	38.8 (26/67)	38.2 (29/76)
Week 54	52.2 (47/90)	51.5 (51/99)	54.8 (23/42)	47.3 (26/55)
**N100**				
**Baseline N count, 1–5**				
Week 12	19.4 (19/98)	26.7 (23/86)	21.8 (19/87)	
Week 24	39.8 (33/83)	33.3 (25/75)	38.9 (14/36)	31.8 (14/44)
Week 54	37.9 (22/58)	40.3 (25/62)	31.0 (9/29)	42.9 (12/28)
**Baseline N count, 6–8**				
Week 12	18.8 (22/117)	22.4 (26/116)	12.1 (15/124)	
Week 24	34.3 (34/99)	28.0 (28/100)	29.7 (19/64)	22.4 (11/49)
Week 54	32.5 (26/80)	36.7 (29/79)	29.2 (14/48)	26.8 (11/41)
**Baseline N count, ≥9**				
Week 12	8.4 (13/154)	7.7 (13/168)	3.2 (5/156)	
Week 24	16.2 (21/130)	16.4 (23/140)	13.4 (9/67)	14.5 (11/76)
Week 54	18.9 (17/90)	17.2 (17/99)	26.2 (11/42)	21.8 (12/55)

C. Abscesses and Inflammatory Nodules (AN)

Endpoint, % (n/N)	Povorcitinib 45 mg	Povorcitinib 75 mg	Placebo→45 mg	Placebo→75 mg
**AN75**				
**Baseline AN count, 5–7**				
Week 12	27.3 (36/132)	40.3 (50/124)	26.4 (34/129)	
Week 24	48.2 (54/112)	44.1 (49/111)	46.6 (27/58)	48.3 (29/60)
Week 54	56.2 (50/89)	58.1 (54/93)	45.2 (19/42)	61.4 (27/44)
**Baseline AN count, 8–14**				
Week 12	32.3 (51/158)	34.2 (55/161)	15.5 (24/155)	
Week 24	42.6 (58/136)	44.2 (61/138)	42.0 (29/69)	38.4 (28/73)
Week 54	51.6 (47/91)	51.0 (53/104)	62.7 (32/51)	50.0 (28/56)
**Baseline AN count, ≥15**				
Week 12	22.6 (19/84)	20.7 (18/87)	9.5 (8/84)	
Week 24	42.0 (29/69)	42.6 (29/68)	32.5 (13/40)	37.8 (14/37)
Week 54	46.2 (24/52)	48.9 (22/45)	46.2 (12/26)	44.0 (11/25)
**AN100**				
**Baseline AN count, 5–7**				
Week 12	16.7 (22/132)	22.6 (28/124)	14.7 (19/129)	
Week 24	31.3 (35/112)	22.5 (25/111)	34.5 (20/58)	25.0 (15/60)
Week 54	34.8 (31/89)	32.3 (30/93)	26.2 (11/42)	36.4 (16/44)
**Baseline AN count, 8–14**				
Week 12	11.4 (18/158)	11.2 (18/161)	5.2 (8/155)	
Week 24	22.1 (30/136)	21.0 (29/138)	17.4 (12/69)	13.7 (10/73)
Week 54	22.0 (20/91)	21.2 (22/104)	21.6 (11/51)	21.4 (12/56)
**Baseline AN count, ≥15**				
Week 12	1.2 (1/84)	2.3 (2/87)	1.2 (1/84)	
Week 24	7.2 (5/69)	7.4 (5/68)	7.5 (3/40)	8.1 (3/37)
Week 54	9.6 (5/52)	4.4 (2/45)	11.5 (3/26)	12.0 (3/25)

D. Draining Tunnels (dT)

Endpoint, % (n/N)	Povorcitinib 45 mg	Povorcitinib 75 mg	Placebo→45 mg	Placebo→75 mg
**dT75**				
**Baseline dT count, 1–2**				
Week 12	46.7 (70/150)	54.6 (77/141)	37.3 (50/134)	
Week 24	50.8 (67/132)	64.0 (80/125)	67.8 (40/59)	59.0 (36/61)
Week 54	59.4 (57/96)	69.9 (65/93)	67.5 (27/40)	77.6 (38/49)
**Baseline dT count, 3–5**				
Week 12	41.8 (33/79)	40.4 (36/89)	25.0 (20/80)	
Week 24	55.2 (32/58)	52.2 (36/69)	48.5 (16/33)	50.0 (20/40)
Week 54	59.5 (25/42)	55.6 (30/54)	48.1 (13/27)	57.7 (15/26)
**Baseline dT count, ≥6**				
Week 12	16.7 (10/60)	24.5 (12/49)	13.2 (9/68)	
Week 24	26.9 (14/52)	36.6 (15/41)	27.6 (8/29)	36.1 (13/36)
Week 54	41.2 (14/34)	51.9 (14/27)	33.3 (7/21)	62.5 (15/24)
**dT100**				
**Baseline dT count, 1–2**				
Week 12	46.7 (70/150)	54.6 (77/141)	37.3 (50/134)	
Week 24	50.8 (67/132)	64.0 (80/125)	67.8 (40/59)	59.0 (36/61)
Week 54	59.4 (57/96)	69.9 (65/93)	67.5 (27/40)	77.6 (38/49)
**Baseline dT count, 3–5**				
Week 12	30.4 (24/79)	36.0 (32/89)	22.5 (18/80)	
Week 24	39.7 (23/58)	44.9 (31/69)	42.4 (14/33)	45.0 (18/40)
Week 54	47.6 (20/42)	44.4 (24/54)	44.4 (12/27)	46.2 (12/26)
**Baseline dT count, ≥6**				
Week 12	10.0 (6/60)	14.3 (7/49)	5.9 (4/68)	
Week 24	7.7 (4/52)	19.5 (8/41)	3.4 (1/29)	13.9 (5/36)
Week 54	23.5 (8/34)	22.2 (6/27)	14.3 (3/21)	20.8 (5/24)

E. Abscesses, Inflammatory Nodules, and Draining Tunnels (ANdT)

Endpoint, % (n/N)	Povorcitinib 45 mg	Povorcitinib 75 mg	Placebo→45 mg	Placebo→75 mg
**ANdT75**				
**Baseline ANdT count, 5–9**				
Week 12	30.1 (40/133)	41.7 (50/120)	20.0 (27/135)	
Week 24	48.7 (57/117)	48.6 (52/107)	46.2 (30/65)	31.6 (18/57)
Week 54	56.5 (52/92	57.6 (53/92)	58.3 (28/48)	50.0 (23/46)
**Baseline ANdT count, 10–15**				
Week 12	26.7 (31/116)	32.8 (45/137)	19.3 (21/109)	
Week 24	34.4 (33/96)	42.1 (51/121)	29.5 (13/44)	42.1 (24/57)
Week 54	45.6 (31/68)	47.8 (43/90)	45.2 (14/31)	52.5 (21/40)
**Baseline ANdT count, ≥16**				
Week 12	22.4 (28/125)	20.0 (23/115)	9.7 (12/124)	
Week 24	35.7 (37/104)	33.7 (30/89)	22.4 (13/58)	30.4 (17/56)
Week 54	44.4 (32/72)	46.7 (28/60)	47.5 (19/40)	48.7 (19/39)

**ANdT100**				
**Baseline ANdT count, 5–9**				
Week 12	17.3 (23/133)	19.2 (23/120)	10.4 (14/135)	
Week 24	27.4 (32/117)	20.6 (22/107)	30.8 (20/65)	8.8 (5/57)
Week 54	26.1 (24/92)	25.0 (23/92)	27.1 (13/48)	23.9 (11/46)
**Baseline ANdT count, 10–15**				
Week 12	6.0 (7/116)	8.0 (11/137)	7.3 (8/109)	
Week 24	15.6 (15/96)	14.0 (17/121)	13.6 (6/44)	15.8 (9/57)
Week 54	20.6 (14/68)	14.4 (13/90)	9.7 (3/31)	17.5 (7/40)
**Baseline ANdT count, ≥16**				
Week 12	1.6 (2/125)	1.7 (2/115)	1.6 (2/124)	
Week 24	5.8 (6/104)	5.6 (5/89)	5.2 (3/58)	10.7 (6/56)
Week 54	6.9 (5/72)	10.0 (6/60)	7.5 (3/40)	12.8 (5/39)

F. Abscesses and Draining Tunnels (AdT)

Endpoint, % (n/N)	Povorcitinib 45 mg	Povorcitinib 75 mg	Placebo→45 mg	Placebo→75 mg
**AdT75**				
**Baseline AdT count, 1–2**				
Week 12	41.8 (41/98)	53.4 (55/103)	38.7 (43/111)	
Week 24	41.4 (36/87)	50.6 (45/89)	72.5 (37/51)	49.0 (25/51)
Week 54	44.4 (28/63)	60.9 (42/69)	71.8 (28/39)	68.3 (28/41)
**Baseline AdT count, 3–5**				
Week 12	32.7 (37/113)	40.0 (42/105)	24.2 (22/91)	
Week 24	42.4 (39/92)	44.7 (42/94)	35.0 (14/40)	42.9 (18/42)
Week 54	52.2 (36/69)	59.2 (42/71)	65.5 (19/29)	53.3 (16/30)
**Baseline AdT count, ≥6**				
Week 12	33.1 (40/121)	22.6 (24/106)	20.3 (24/118)	
Week 24	39.0 (39/100)	48.8 (41/84)	40.4 (21/52)	42.4 (25/59)
Week 54	44.1 (30/68)	48.4 (30/62)	47.4 (18/38)	64.1 (25/39)
**AdT100**				
**Baseline AdT count, 1–2**				
Week 12	41.8 (41/98)	53.4 (55/103)	38.7 (43/111)	
Week 24	41.4 (36/87)	50.6 (45/89)	72.5 (37/51)	49.0 (25/51)
Week 54	44.4 (28/63)	60.9 (42/69)	71.8 (28/39)	68.3 (28/41)
**Baseline AdT count, 3–5**				
Week 12	22.1 (25/113)	31.4 (33/105)	16.5 (15/91)	
Week 24	32.6 (30/92)	31.9 (30/94)	30.0 (12/40)	38.1 (16/42)
Week 54	46.4 (32/69)	38.0 (27/71)	41.4 (12/29)	46.7 (14/30)
**Baseline AdT count, ≥6**				
Week 12	15.7 (19/121)	13.2 (14/106)	6.8 (8/118)	
Week 24	19.0 (19/100)	25.0 (21/84)	17.3 (9/52)	16.9 (10/59)
Week 54	23.5 (16/68)	24.2 (15/62)	13.2 (5/38)	25.6 (10/39)

Data from STOP-HS1 and STOP-HS2 were pooled and summarized descriptively. A75/100=≥75%/100% decrease from baseline in abscess count; AdT75/100=≥75%/100% decrease from baseline in abscess and draining tunnel count; AN75/100=≥75%/100% decrease from baseline in abscess and inflammatory nodule count; ANdT75/100=≥75%/100% decrease from baseline in abscess, inflammatory nodule, and draining tunnel count;

dT75/100=≥75%/100% decrease from baseline in draining tunnel count; N75/100=≥75%/100% decrease from baseline in inflammatory nodule count.

Table S7. STOP-HS1 and STOP-HS2 Pooled TEAEs by Severity Grade in the Full Study Period

Event, n (%)	Povorcitinib 45 mg (n=594)	Povorcitinib 75 mg (n=594)	Placebo (n=405)
Any TEAE	459 (77.3)	488 (82.2)	205 (50.6)
Mild	112 (18.9)	118 (19.9)	83 (20.5)
Moderate	308 (51.9)	323 (54.4)	110 (27.2)
Severe	39 (6.6)	46 (7.7)	12 (3.0)

Patients receiving placebo were evaluated from baseline through Week 12; povorcitinib 45 mg and 75 mg groups represent patients who received at least one dose of povorcitinib and were treated up to Week 54. Data from STOP-HS1 and STOP-HS2 were pooled and summarized descriptively. TEAE=treatment-emergent adverse event.

Table S8. STOP-HS1 and STOP-HS2 Pooled Incidence and Exposure-Adjusted Rates of TEAEs (≥5%), AESIs, and ECIs Through Week 54 by Treatment Regimen

Event, n (%) [EAIR per 100 PY]	Overall Study Period (Weeks 0–54)		
	**Povorcitinib 45 mg (n=594)**	**Povorcitinib 75 mg (n=594)**	**Povorcitinib Total (N=1188)**
Person-years of exposure	447.89	454.72	902.61
TEAE occurring in ≥5% in any povorcitinib group			
Acne	99 (17%) [22.1]	119 (20%) [26.2]	218 (18%) [24.2]
Nasopharyngitis	61 (10%) [13.6]	72 (12%) [15.8]	133 (11%) [14.7]
Upper respiratory tract infection	63 (11%) [14.1]	61 (10%) [13.4]	124 (10%) [13.7]
Headache	45 (8%) [10.0]	50 (8%) [11.0]	95 (8%) [10.5]
COVID-19	32 (5%) [7.1]	51 (9%) [11.2]	83 (7%) [9.2]
Urinary tract infection	35 (6%) [7.8]	32 (5%) [7.0]	67 (6%) [7.4]
Influenza	27 (5%) [6.0]	33 (6%) [7.3]	60 (5%) [6.6]
Hidradenitis	29 (5%) [6.5]	30 (5%) [6.6]	59 (5%) [6.5]
Any TEAE of special interest, n (%)	57 (10%) [12.7]	58 (10%) [12.8]	115 (10%) [12.7]
MACE*	1 (<1%) [0.2]	0	1 (<1%) [0.1]
Venous embolic/thrombotic event*	1 (<1%) [0.2]	2 (<1%) [0.4]	3 (<1%) [0.3]
Serious infection*	6 (1%) [1.3]	12 (2%) [2.6]	18 (2%) [2.0]
Opportunistic infection*	5 (1%) [1.1]	1 (<1%) [0.2]	6 (1%) [0.7]
Tuberculosis*	0	0	0
Herpes zoster*	11 (2%) [2.5]	12 (2%) [2.6]	23 (2%) [2.5]
Malignancies*	4 (1%) [0.9]	1 (<1%) [0.2]	5 (<1%) [0.6]
NMSC*	4 (1%) [0.9]	1 (<1%) [0.2]	5 (<1%) [0.6]
Excluding NMSC*	0	0	0
Gastrointestinal perforations*	0	0	0
HS exacerbation	29 (5%) [6.5]	30 (5%) [6.6]	59 (5%) [6.5]
Events of clinical interest, n (%)	143 (24%) [31.9]	179 (30%) [39.4]	322 (27%) [35.7]
Renal dysfunction events*	3 (1%) [0.7]	7 (1%) [1.5]	10 (1%) [1.1]
Hepatic events*	23 (4%) [5.1]	31 (5%) [6.8]	54 (5%) [6.0]
Increased CPK*	30 (5%) [6.7]	35 (6%) [7.7]	65 (5%) [7.2]
Rhabdomyolysis	0	0	0
Myositis	0	0	0
Acne*	104 (18%) [23.2]	132 (22%) [29.0]	236 (20%) [26.1]

Data from STOP-HS1 and STOP-HS2 were pooled and summarized descriptively. *Each term represents grouped TEAEs based on Standard MedDRA Query or custom preferred-term search. AESI=adverse events of special interest; CPK=creatine phosphokinase; EAIR, exposure-adjusted incidence rate; ECI=events of clinical interest; HS=hidradenitis suppurativa; MACE=major adverse cardiovascular event; NMSC=nonmelanoma skin cancer; TEAE=treatment-emergent adverse event.

Table S9. Select Safety Narratives

Event/Patient Details	Safety Narrative
**Death**<br>**(Grade 5)**<br>31-year-old female, White, Hurley Stage III, baseline AN count 30, dT count 5, HS duration 7.4 years, current smoker, BMI 67.2 kg/m^2	**Relevant medical history:** hypertension, severe obesity, sleep apnoea syndrome, type 2 diabetes mellitus, neuropathy peripheral, spinal pain, anxiety, asthma, gastrooesophageal reflux disease, irritable bowel syndrome, polycystic ovarian syndrome, bipolar disorder, migraine, and impaired gastric emptying. The participant's history also included multiple prior surgeries for HS and a family history of sudden cardiac death. Concomitant medications at the time of the event included insulin, opioids, antihypertensives, antiepileptics, and psychotropic agents.<br>**Treatment group:** participant was randomised to placebo (Day 1–91); during the extension period, she crossed over to povorcitinib 45 mg qd on Day 92; event happened on Day 313.<br>**Narrative:** in the months prior to the event, the participant underwent surgery for a right foot fracture with cast placement, followed by a period of immobilisation. Soon afterward, while still wearing a cast on her right leg, the participant had a second fall and sustained fractures of the left fibula and tibia. The participant was hospitalised for these fractures but did not receive standard antithrombotic therapy despite prolonged immobilisation. The participant was discharged 2 weeks later and was found deceased at home later that day. The cause of death was undetermined, and no autopsy was performed.
**Ischaemic stroke**<br>**(Grade 4)**<br>34-year-old female, White, Hurley Stage II, baseline AN count 9, dT count 1, HS duration 4.4 years, current smoker, BMI 41.4 kg/m^2	**Relevant medical history:** obesity and post-traumatic stress disorder. Concomitant medications at the time of the event included cetirizine, acyclovir, and ibuprofen/paracetamol.<br>**Treatment group:** participant was randomised to povorcitinib 45 mg qd (Day 1–85) and continued treatment in the extension period (Day 86 onward) with the same dose; drug was interrupted on Day 377; event happened on Day 386.<br>**Narrative:** in the week prior to the event, the participant reported persistent headaches. She was hospitalised and diagnosed with ophthalmic migraine. Despite unremarkable imaging (CT, CT angiography, and MRI) findings, which showed no acute intracranial process or vessel occlusion, she received treatment with

	tenecteplase, acetylsalicylic acid, atorvastatin, and antihypertensives. The study drug was discontinued, and the participant was discharged 2 days later.
**Bilateral pulmonary emboli (Grade 3)**<br>32-year-old male, White, Hurley Stage II, baseline AN count 15, dT count 0, HS duration 5.4 years, non-smoker, BMI 33.9 kg/m^2	**Relevant medical history:** The participant had a family history of deep vein thrombosis on the paternal side, including his father and grandfather. The participant had no relevant concomitant medications.<br>**Treatment group:** participant was randomised to povorcitinib 75 mg qd (Day 1–87) and continued treatment in the extension period (Day 88 onward) with the same dose; drug was discontinued on Day 201, the same day event was reported.<br>**Narrative:** in the months prior to the event, the participant experienced periods of relative immobilisation, including two flights and two long-distance drives. He developed shortness of breath, dry cough, and chest pain and was hospitalised for bilateral pulmonary embolism, confirmed by CT pulmonary angiogram. Treatment included dalteparin, followed by a discharge regimen of apixaban.
**Deep vein thrombosis (Grade 2)**<br>40-year-old female, White, Hurley Stage II, baseline AN count 5, dT count 0, HS duration 10.2 years, current smoker, BMI 42.6 kg/m^2	**Relevant medical history:** hypercholesterolaemia, metabolic dysfunction–associated liver disease, type 2 diabetes mellitus, osteoarthritis, and COVID-19. The participant also had a family history of sudden cardiac death (grandparent), and a history of factor V Leiden, protein C or S deficiency, prothrombin gene mutation, or thromboembolic disease.<br>**Treatment group:** participant was randomised to placebo (Day 1–84); during the extension period, crossed over to povorcitinib 75 mg qd on Day 85; event happened on Day 89.<br>**Narrative:** participant experienced a deep vein thrombosis following a recent long-haul flight. No relevant laboratory or diagnostic tests were performed to confirm the event. Treatment was initiated with a single dose of dalteparin sodium 18,000 IU, followed by rivaroxaban 15 mg twice daily.
**Breast cancer (Grade 3)**<br>53-year-old female, White, Hurley Stage II, baseline AN count 9, dT count 0, HS	**Relevant medical history:** obesity, hypertension, and hypothyroidism. Concomitant medications at the time of the event included dienogest/estradiol valerate, ramipril, and levothyroxine.<br>**Treatment group:** participant was randomised to placebo (Day 1 onward); event was reported on Day 53, while participant was still receiving placebo.<br>**Narrative:** in the 3 weeks prior to the event, the participant noticed a tumour in her right breast with visible tissue retraction. She was referred to radiology following a gynaecologic consultation. MRI revealed a mass

duration 6.7 years, current smoker, BMI 36.1 kg/m^2	with typical malignant morphology, and a subsequent biopsy confirmed invasive breast carcinoma. The participant withdrew from the study, and treatment was not reported at that time.
**Unstable angina (Grade 4)**<br>48-year-old male, White, Hurley Stage II, baseline AN count 10, dT count 1, HS duration 10.9 years, former smoker, BMI 38.9 kg/m^2	**Relevant medical history:** coronary artery disease, obesity, hypertension, hepatic steatosis, and hypercholesterolaemia. Concomitant medications at the time of the event included lisinopril, carvedilol, acetylsalicylic acid, glyceryl trinitrate, vitamins, and semaglutide.<br>**Treatment group:** participant was randomised to placebo (Day 1–84); during the extension period, crossed over to povorcitinib 75 mg qd on Day 85. However, the first dose of povorcitinib was received on Day 104 and continued through study discontinuation on Day 225; event was reported during the safety follow-up period, on Day 239.<br>**Narrative:** during the safety follow-up period, the participant discussed worsening chest pain with a cardiologist. He was hospitalised, and ECG, chest x-ray, and CT angiography findings were unremarkable. The participant underwent planned coronary balloon angiography and stent placement. The procedure was completed without sequelae, and his blood pressure normalised during the stay. He was discharged with a medication regimen including clopidogrel and rosuvastatin.

AN=abscess and inflammatory nodule; BMI=body mass index; CT=computed tomography; dT=draining tunnel; ECG=electrocardiogram; HS=hidradenitis suppurativa; IU=international units; MRI=magnetic resonance imaging; qd=once daily.....................................

Table S10. STOP-HS1 and STOP-HS2 Pooled, Additional Information on TEAEs of Acne Through Week 54

Event	Placebo-Controlled Period Only (Weeks 0–12)				Extension Period Only (After Week 12)			Povorcitinib-Randomised (Weeks 0–54)		
	Povorcitinib 45 mg (n=412)	Povorcitinib 75 mg (n=409)	Placebo (n=405)	Povorcitinib total (N=821)	Placebo→ Povorcitinib 45 mg (n=182)	Placebo→ Povorcitinib 75 mg (n=185)	Total (N=367)	Povorcitinib 45 mg (n=412)	Povorcitinib 75 mg (n=409)	Total (N=821)
Patients with available additional information on acne TEAE, n (%)	35 (8.5)	58 (14.2)	19 (4.7)	93 (11.3)	32 (17.6)	29 (15.7)	61 (16.6)	72 (17.5)	103 (25.2)	175 (21.3)
Predominant form/type of acne (grouped), n (%)										
Nodules/cysts	3 (8.3)	5 (8.5)	1 (5.3)	8 (8.4)	5 (14.3)	1 (3.2)	6 (9.1)	8 (10.4)	8 (6.8)	16 (8.2)
Papules/pustules	24 (66.7)	47 (79.7)	16 (84.2)	71 (74.7)	20 (57.1)	20 (64.5)	40 (60.6)	49 (63.6)	90 (76.3)	139 (71.3)
Whiteheads/blackheads	9 (25.0)	4 (6.8)	2 (10.5)	13 (13.7)	9 (25.7)	10 (32.3)	19 (28.8)	19 (24.7)	16 (13.6)	35 (17.9)
Anatomic location of acne lesions, n (%)										
Face/scalp	31 (86.1)	51 (86.4)	17 (89.5)	82 (86.3)	30 (85.7)	28 (90.3)	58 (87.9)	68 (88.3)	103 (87.3)	171 (87.7)
Neck	10 (27.8)	10 (16.9)	3 (15.8)	20 (21.1)	5 (14.3)	4 (12.9)	9 (13.6)	17 (22.1)	17 (14.4)	34 (17.4)
Chest	4 (11.1)	13 (22.0)	4 (21.1)	17 (17.9)	5 (14.3)	6 (19.4)	11 (16.7)	7 (9.1)	18 (15.3)	25 (12.8)
Torso	5 (13.9)	8 (13.6)	3 (15.8)	13 (13.7)	3 (8.6)	4 (12.9)	7 (10.6)	10 (13.0)	13 (11.0)	23 (11.8)
Buttock/groin	2 (5.6)	1 (1.7)	0	3 (3.2)	1 (2.9)	2 (6.5)	3 (4.5)	3 (3.9)	2 (1.7)	5 (2.6)
Thigh/leg	1 (2.8)	0	0	1 (1.1)	0	2 (6.5)	2 (3.0)	2 (2.6)	0	2 (1.0)
Other	4 (11.1)	7 (11.9)	1 (5.3)	11 (11.6)	6 (17.1)	3 (9.7)	9 (13.6)	6 (7.8)	11 (9.3)	17 (8.7)
Number of anatomic locations, n (%)										
1–2 locations	31 (86.1)	45 (76.3)	17 (89.5)	76 (80.0)	30 (85.7)	27 (87.1)	57 (86.4)	68 (88.3)	99 (83.9)	167 (85.6)
≥3 locations	5 (13.9)	13 (22.0)	2 (10.5)	18 (18.9)	4 (11.4)	4 (12.9)	8 (12.1)	8 (10.4)	16 (13.6)	24 (12.3)
Episodes associated with new medication, n (%)	17 (47.2)	34 (57.6)	8 (42.1)	51 (53.7)	15 (42.9)	17 (54.8)	32 (48.5)	37 (48.1)	69 (58.5)	106 (54.4)

Data from STOP-HS1 and STOP-HS2 were pooled and summarized descriptively. BL=baseline; TEAE=treatment-emergent adverse event.

Table S11. STOP-HS1 and STOP-HS2 Pooled Worst Laboratory Grade Shifts From Baseline During Study Periods

A. Haemoglobin (g/L)

Study period, worst CTCAE grade change and mean CfB	Baseline	Grade 0 (≥LLN)			Grade 1 (<LLN to 100 g/L)			Grade 2 (<100 to 80 g/L)		
		Placebo	45 mg	75 mg	Placebo	45 mg	75 mg	Placebo	45 mg	75 mg
	N	345	354	365	53	47	37	7	11	7
	Mean (SD)	139.0 (12.24)	138.0 (12.46)	138.1 (11.40)	113.5 (7.70)	113.7 (7.75)	113.9 (8.73)	94.1 (3.63)	93.7 (4.08)	94.0 (5.20)
12 Weeks (PC Period)	Median CfB	−1.50	−1.12	−2.00	−1.54	0.88	0.42	0.58	4.50	2.25
	n (%) Gr2	1 (0.3)	2 (0.6)	1 (0.3)	13 (24.5)	4 (8.5)	6 (16.2)	5 (71.4)	10 (90.9)	5 (71.4)
	n (%) Gr3	0 (0.0)	0 (0.0)	0 (0.0)	0 (0.0)	0 (0.0)	0 (0.0)	0 (0.0)	0 (0.0)	1 (14.3)
54 Weeks (Povorcitinib Randomised)	Median CfB	—	−2.00	−3.44	—	−0.08	0.59	—	5.00	2.25
	n (%) Gr2	—	12 (3.4)	11 (3.0)	—	15 (31.9)	10 (27.0)	—	9 (81.8)	6 (85.7)
	n (%) Gr3	—	1 (0.3)	0 (0.0)	—	0 (0.0)	1 (2.7)	—	1 (9.1)	1 (14.3)

LLN values were as follows: females aged 12–65 years, 116 g/L; females aged 66–133 years, 110 g/L; males, 130 g/L.

B. Lymphocytes (counts, 10^9/L)

Study period, worst CTCAE grade change and mean CfB	Baseline	Grade 0 (≥LLN)			Grade 1 (<LLN to 0.8×10^9/L)			Grade 2 (<0.8 to <0.5×10^9/L)		
		Placebo	45 mg	75 mg	Placebo	45 mg	75 mg	Placebo	45 mg	75 mg
	N	402	408	406	2	4	3	1	0	0
	Mean (SD)	2.32 (0.71)	2.34 (0.86)	2.33 (0.68)	0.91 (0.01)	0.92 (0.05)	0.92 (0.08)	0.66 (–)	–	–
12 Weeks (PC Period)	Median CfB	0.02	0.20	0.12	0.77	0.67	0.31	0.24	–	–
	n (%) Gr2	4 (1.0)	1 (0.2)	2 (0.5)	0	0	1 (33.3)	1 (100.0)	0	0
	n (%) Gr3	0	0	0	0	0	0	0	0	0
54 Weeks (Povorcitinib Randomised)	Median CfB	—	0.088	0.04	—	0.44	0.30	—	–	–
	n (%) Gr2	—	2 (0.5)	10 (2.5)	—	2 (50.0)	1 (33.3)	—	0	0
	n (%) Gr3	—	1 (0.2)	1 (0.2)	—	0	0	—	0	0

LLN was 1.02×10^9/L.

C. Neutrophils (counts, 10^9/L)

Study period, worst CTCAE grade change and mean CfB	Baseline	Grade 0 (≥LLN)			Grade 1 (<LLN to 1.5×10^9/L)			Grade 2 (<1.5 to 1.0×10^9/L)		
		Placebo	45 mg	75 mg	Placebo	45 mg	75 mg	Placebo	45 mg	75 mg
	N	403	407	404	2	4	5	0	1	0
	Mean (SD)	6.11 (2.43)	6.04 (2.49)	6.26 (2.40)	1.66 (0.13)	1.74 (0.05)	1.77 (0.16)	–	1.21 (–)	–
12 Weeks (PC Period)	Median CfB	0.14	−0.22	−0.75	0.52	0.02	1.27	–	1.38	–
	n (%) Gr2	1 (0.2)	4 (1.0)	6 (1.5)	1 (50.0)	3 (75.0)	1 (20.0)	0	0	0
	n (%) Gr3	1 (0.2)	0	0	0	0	1 (20.0)	0	0	0
54 Weeks (Povorcitinib Randomised)	Median CfB	—	−0.03	−0.43	—	0.55	1.45	—	1.38	–
	n (%) Gr2	—	5 (1.2)	7 (1.7)	—	3 (75.0)	1 (20.0)	—	0	0
	n (%) Gr3	—	1 (0.2)	0	—	0	1 (20.0)	—	0	0

LLN was 2.03×10^9/L.

D. Platelets (counts, 10^9/L)

Study period, worst CTCAE grade change and mean CfB	Baseline	Grade 0 (≥LLN)			Grade 1 (<LLN to 75×10^9/L)			Grade 2 (<75 to <50×10^9/L)		
		Placebo	45 mg	75 mg	Placebo	45 mg	75 mg	Placebo	45 mg	75 mg
	N	404	410	408	1	2	1	0	0	0
	Mean (SD)	327.1 (97.24)	325.0 (94.76)	317.1 (85.73)	131.0 (–)	126.0 (16.97)	134.0 (–)	–	–	–
12 Weeks (PC Period)	Median CfB	−1.63	−48.00	−72.83	39.50	−39.33	3.25	–	–	–
	n (%) Gr2	0	0	2 (0.5)	0	1 (50.0)	0	0	0	0
	n (%) Gr3	0	0	0	0	0	0	0	0	0
54 Weeks (Povorcitinib Randomised)	Median CfB	—	−40.42	−57.06	—	−22.06	−2.80	—	–	–
	n (%) Gr2	—	0	2 (0.5)	—	1 (50.0)	0	—	0	0
	n (%) Gr3	—	0	0	—	0	0	—	0	0

LLN was 140×10^9/L.

E. Alanine Aminotransferase (U/L)

Study period, worst CTCAE grade change and mean CfB	Baseline	Grade 0 (≤ULN)		
		Placebo	45 mg	75 mg
	N	405	412	409
	Mean (SD)	21.0 (12.29)	21.3 (12.99)	21.0 (13.64)
12 Weeks (PC Period)	Median CfB	0	1.75	1.50
	n (%) Gr2	0	0	0
	n (%) Gr3	0	1 (0.2)	0
54 Weeks (Povorcitinib Randomised)	Median CfB	—	2.18	2.08
	n (%) Gr2	—	4 (1.0)	5 (1.2)
	n (%) Gr3	—	1 (0.2)	0

ULN values were as follows: females, 33 U/L; males, 41 U/L. Grade 2: >3.0 to 5.0×ULN; grade 3: >5.0 to 20.0×ULN.

F. Aspartate Aminotransferase (U/L)

Study period, worst CTCAE grade change and mean CfB	Baseline	Grade 0 (≤ULN)		
		Placebo	45 mg	75 mg
	N	405	412	409
	Mean (SD)	19.6 (9.85)	19.4 (8.30)	20.6 (24.81)
12 Weeks (PC Period)	Median CfB	0	2.50	3.00
	n (%) Gr2	0	0	1 (0.2)
	n (%) Gr3	0	2 (0.5)	0
54 Weeks (Povorcitinib Randomised)	Median CfB	—	2.60	3.64
	n (%) Gr2	—	4 (1.0)	4 (1.0)
	n (%) Gr3	—	2 (0.5)	1 (0.2)

ULN values were as follows: females, 32 U/L; males, 40 U/L. Grade 2: >3.0 to 5.0×ULN; grade 3: >5.0 to 20.0×ULN

G. Creatine Kinase (U/L)

Study period, worst CTCAE grade change and mean CfB	Baseline	Grade 0 (≤ULN)			Grade 1 (>ULN to 2.5×ULN)			Grade 2 (>2.5×ULN to 5×ULN)			Grade 3 (>5×ULN to 10×ULN)			Grade 4 (>10×ULN)		
		Placebo	45 mg	75 mg	Placebo	45 mg	75 mg	Placebo	45 mg	75 mg	Placebo	45 mg	75 mg	Placebo	45 mg	75 mg
	N	379	375	374	24	29	23	0	6	7	0	1	2	2	1	3
	Mean (SD)	92.3 (48.66)	93.9 (50.07)	90.1 (50.43)	315.2 (80.65)	337.3 (117.35)	371.3 (130.40)	–	871.7 (163.31)	740.3 (190.59)	–	1552.0 (–)	1633.0 (902.27)	5118.5 (2512.35)	4224.0 (–)	10268.3 (10598.16)
**12 Weeks (PC Period)**	Median CfB	2.00	43.75	62.71	−53.13	45.63	44.00	–	−364.50	−243.50	–	−1198.25	−1418.62	−4941.12	−3945.00	−4761.25
	n (%) Gr2	0	11 (2.9)	8 (2.1)	4 (16.7)	8 (27.6)	5 (21.7)	0	3 (50.0)	2 (28.6)	0	0	0	0	0	1 (33.3)
	n (%) Gr3	5 (1.3)	3 (0.8)	8 (2.1)	0	1 (3.4)	4 (17.4)	0	1 (16.7)	1 (14.3)	0	0	0	0	0	0
**54 Weeks (Povorcitinib Randomised)**	Median CfB	—	51.10	68.54	—	61.42	44.00	—	−354.58	−289.67	—	−1231.41	−1437.99	—	−3711.66	−4609.66
	n (%) Gr2	—	24 (6.4)	28 (7.5)	—	14 (48.3)	5 (21.7)	—	3 (50.0)	1 (14.3)	—	0	0	—	0	0
	n (%) Gr3	—	7 (1.9)	13 (3.5)	—	1 (3.4)	6 (26.1)	—	1 (16.7)	2 (28.6)	—	0	0	—	1 (100.0)	1 (33.3)

ULN values were as follows: females, 192 U/L; males, 308 U/L.

H. Creatinine (μmol/L)

Study period, worst CTCAE grade change and mean CfB	Baseline	Grade 0 (≤ULN)			Grade 1 (>ULN to 1.5×ULN)			Grade 2 (>1.5 to 3.0×ULN)		
		Placebo	45 mg	75 mg	Placebo	45 mg	75 mg	Placebo	45 mg	75 mg
	N	388	388	386	17	24	22	0	0	1
	Mean (SD)	70.3 (12.59)	69.3 (12.00)	70.1 (13.13)	95.8 (9.25)	101.7 (11.59)	103.2 (15.72)	–	–	132.0 (–)
**12 Weeks (PC Period)**	Median CfB	0	3.50	4.50	−8.00	−3.25	−0.88	–	–	−13.67
	n (%) Gr2	1 (0.3)	2 (0.5)	1 (0.3)	0	1 (4.2)	1 (4.5)	0	0	1 (100.0)
	n (%) Gr3	0	0	0	0	0	0	0	0	0
**54 Weeks (Povorcitinib Randomised)**	Median CfB	—	3.33	4.04	—	−2.00	1.38	—	0	−13.67
	n (%) Gr2	—	7 (1.8)	7 (1.8)	—	2 (8.3)	3 (13.6)	—	0	1 (100.0)
	n (%) Gr3	—	0	0	—	0	0	—	0	0

ULN values were as follows: females, 84 μmol/L (0.95 mg/dL); males, 103 μmol/L (1.17 mg/dL).

I. Total Cholesterol (mmol/L)

Study period, worst CTCAE grade change and mean CfB	Baseline	Grade 0 (≤ULN)			Grade 1 (>ULN to 7.75 mmol/L)			Grade 2 (>7.75 to 10.34 mmol/L)		
		Placebo	45 mg	75 mg	Placebo	45 mg	75 mg	Placebo	45 mg	75 mg
	N	285	280	282	111	124	115	2	3	1
	Mean (SD)	4.22 (0.59)	4.23 (0.57)	4.22 (0.63)	5.92 (0.59)	5.87 (0.56)	5.94 (0.59)	8.16 (0.30)	8.70 (0.26)	7.77 (–)
12 Weeks (PC Period)	Median CfB	0	0.32	0.43	−0.30	0.13	0.38	−1.89	−0.75	1.50
	n (%) Gr2	0	1 (0.4)	1 (0.4)	2 (1.8)	8 (6.5)	17 (14.8)	0	2 (66.7)	1 (100.0)
	n (%) Gr3	0	0	0	0	1 (0.8)	0	0	0	0
54 Weeks (Povorcitinib Randomised)	Median CfB	—	0.32	0.44	—	0.10	0.33	—	−0.78	1.38
	n (%) Gr2	—	2 (0.7)	2 (0.7)	—	14 (11.3)	19 (16.5)	—	3 (100.0)	1 (100.0)
	n (%) Gr3	—	0	0	—	1 (0.8)	2 (1.7)	—	0	0

ULN was ≥5.16 mmol/L (200 mg/dL).

J. Triglycerides (mmol/L)

Study period, worst CTCAE grade change and mean CfB	Baseline	Grade 0 (≤1.71 mmol/L)			Grade 1 (1.71 to 3.42 mmol/L)			Grade 2 (>3.42 to 5.7 mmol/L)			Grade 3(>5.7 to 11.4 mmol/L)			Grade 4 (>11.4 mmol/L)		
		Placebo	45 mg	75 mg	Placebo	45 mg	75 mg	Placebo	45 mg	75 mg	Placebo	45 mg	75 mg	Placebo	45 mg	75 mg
	N	282	293	304	100	94	88	17	15	9	1	4	2	1	1	0
	Mean (SD)	1.04 (0.31)	1.06 (0.33)	1.04 (0.33)	2.31 (0.45)	2.27 (0.46)	2.27 (0.46)	3.94 (0.48)	3.89 (0.34)	4.24 (0.79)	7.09 (–)	7.90 (1.60)	7.06 (1.79)	33.26 (–)	16.06 (–)	–
12 Weeks (PC Period)	Median CfB	0.03	0.05	0.03	−0.14	−0.12	−0.27	−0.59	−1.15	−1.32	−5.26	−4.70	−1.09	–	−13.17	–
	n (%) Gr2	0	0	5 (1.6)	10 (10.0)	16 (17.0)	10 (11.4)	8 (47.1)	3 (20.0)	4 (44.4)	0	1 (25.0)	1 (50.0)	0	0	0
	n (%) Gr3	0	0	1 (0.3)	1 (1.0)	2 (2.1)	2 (2.3)	2 (11.8)	0	0	0	1 (25.0)	0	1 (100.0)	0	0
54 Weeks (Povorcitinib Randomised)	Median CfB	—	0.04	0.04	—	−0.11	−0.27	—	−1.20	−1.16	—	−4.31	−2.67	—	−13.32	0
	n (%) Gr2	—	5 (1.7)	6 (2.0)	—	18 (19.1)	15 (17.0)	—	6 (40.0)	5 (55.6)	—	2 (50.0)	1 (50.0)	—	0	0
	n (%) Gr3	—	0	2 (0.7)	—	6 (6.4)	3 (3.4)	—	1 (6.7)	0	—	1 (25.0)	0	—	0	0

ULN was 1.71 mmol/L (≈150 mg/dL).
Table shows the baseline CTCAE grade for each treatment group and the respective mean (SD) for each group, followed by the percentage of participants in each CTCAE grade (worst grade for study period, 12 or 54 weeks), and the respective median CfB. Grade levels not shown in a table indicate no patients had a

laboratory abnormality at that grade or above. Data from STOP-HS1 and STOP-HS2 were pooled and summarized descriptively. CfB=change from baseline; CTCAE=Common Terminology Criteria for Adverse Events; LLN=lower limit of normal; PC=placebo-controlled; ULN=upper limit of normal.

Figure S1. Skin Pain NRS30 Response Over Time Through Week 54 (Among Patients With Skin Pain NRS ≥3 at Baseline; OC and mNRI)

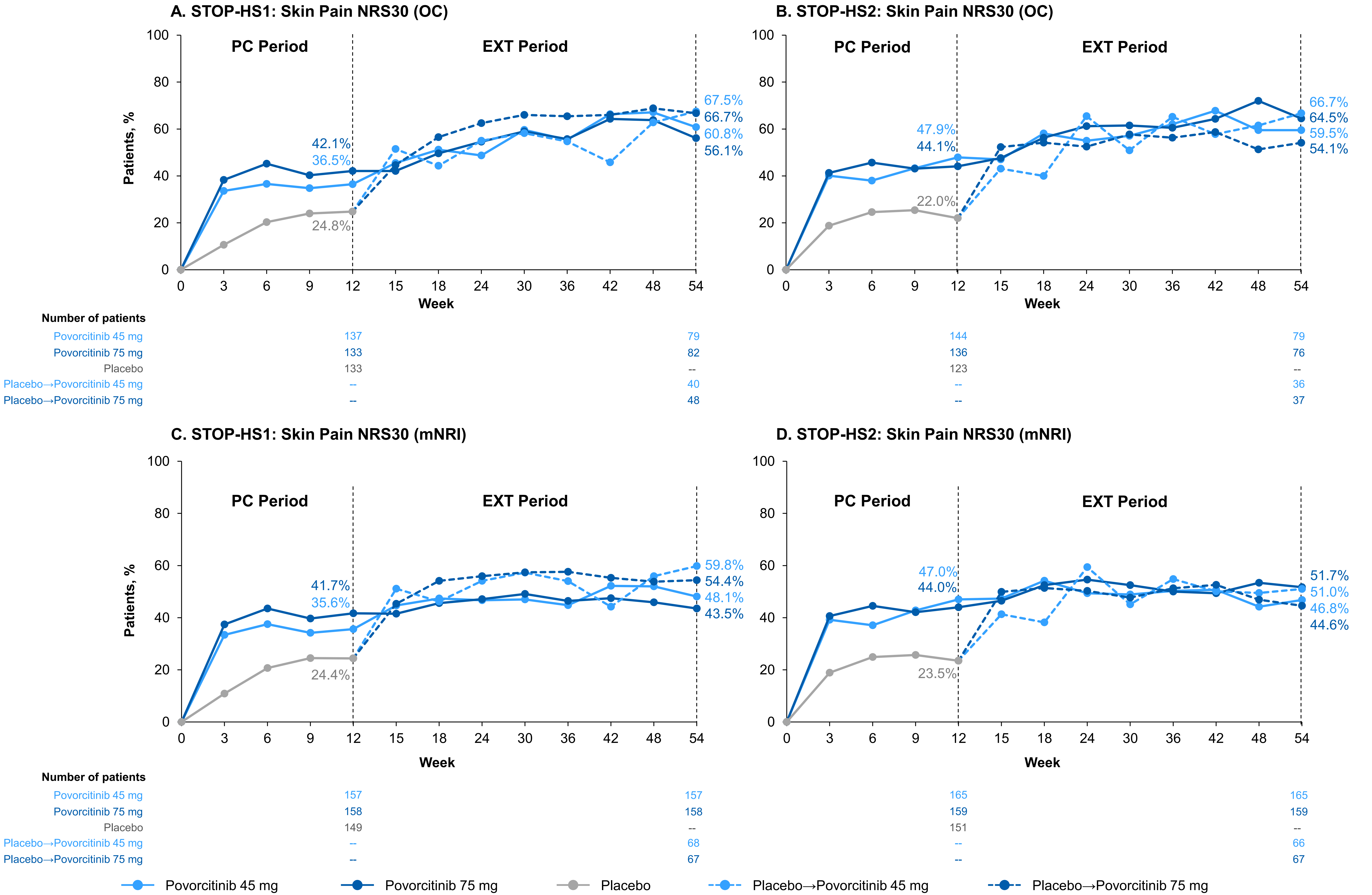

Data were summarized descriptively and the estimation was combined using Rubin's rule for mNRI. EXT=extension; HS=hidradenitis suppurativa; mNRI=modified nonresponder imputation; NRS=numerical rating scale; NRS30=at least 30% reduction and at least 1-unit reduction from baseline in Skin Pain; OC=observed cases; PC=placebo-controlled.

Figure S2. STOP-HS1 and STOP-HS2 Pooled IHS4 Responses From Randomisation Through Week 54 (OC)

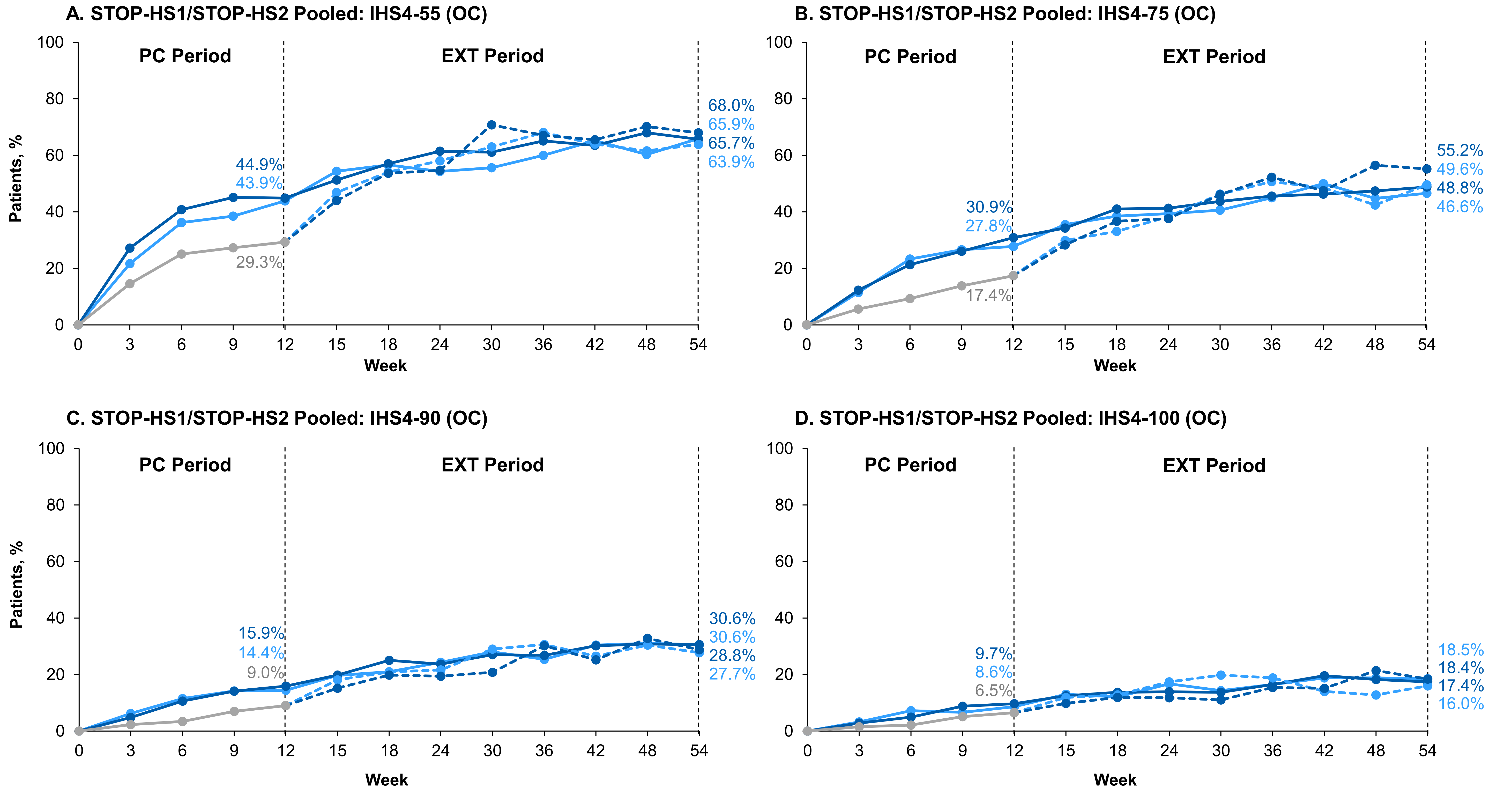

Number of patients

	C: Week 12	C: Week 54	D: Week 12	D: Week 54
Povorcitinib 45 mg	374	232	374	232
Povorcitinib 75 mg	372	242	372	242
Placebo	368	--	368	--
Placebo→Povorcitinib 45 mg	--	119	--	119
Placebo→Povorcitinib 75 mg	--	125	--	125

Povorcitinib 45 mg — Povorcitinib 75 mg — Placebo — Placebo→Povorcitinib 45 mg — Placebo→Povorcitinib 75 mg

Data from STOP-HS1 and STOP-HS2 were pooled and summarized descriptively. EXT=extension; IHS4=International Hidradenitis Suppurativa Severity Score System; IHS4-55/75/90/100=≥55%/≥75%/≥90%/100% reduction from baseline in IHS4 score; OC=observed cases; PC=placebo-controlled

Figure S3. STOP-HS1 and STOP-HS2 Pooled Median Percentage Change in hsCRP From Baseline Through Week 54 (OC)

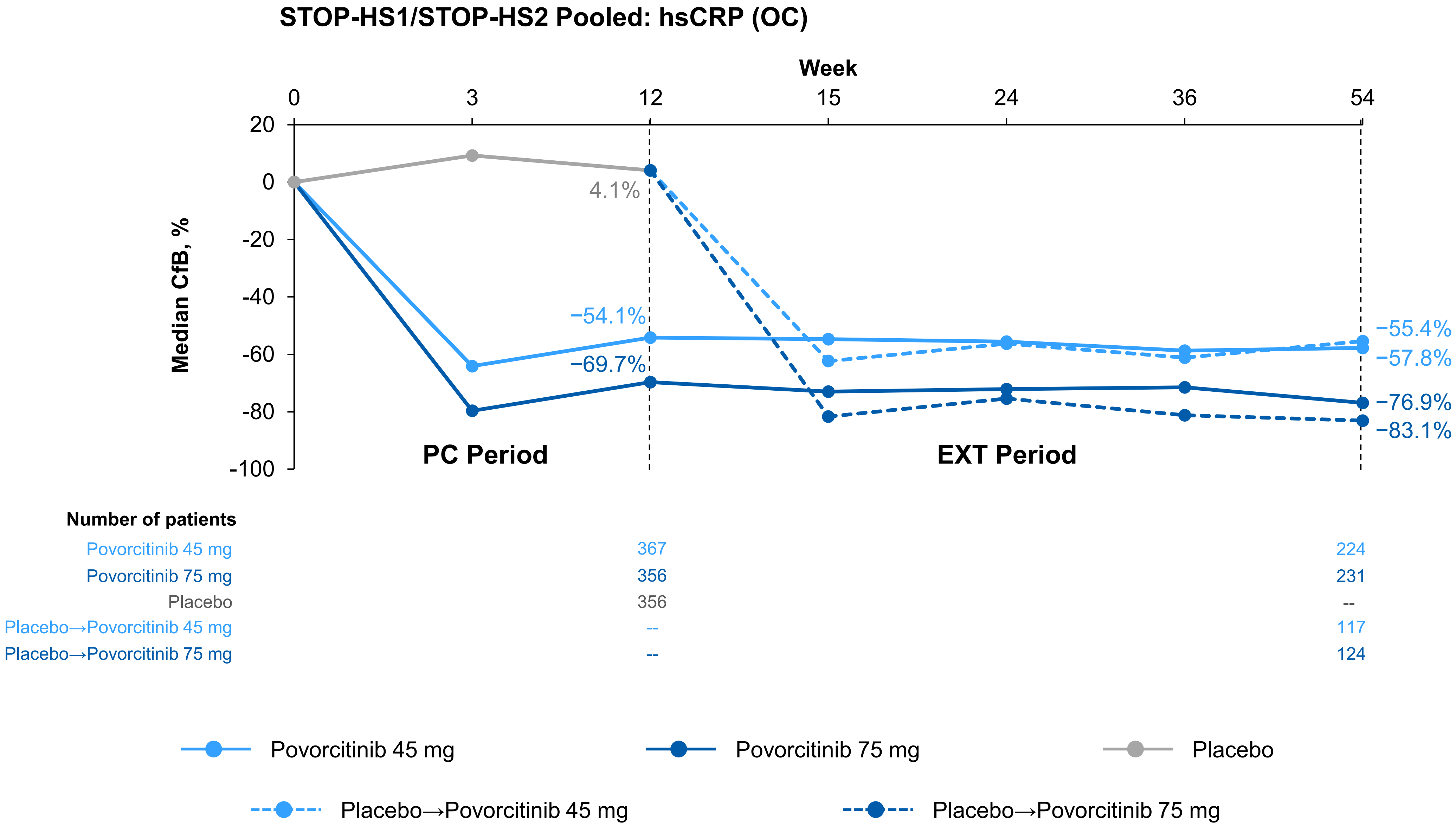

Data from STOP-HS1 and STOP-HS2 were pooled summarized descriptively. CfB=change from baseline; EXT=extension; hsCRP=high-sensitivity C-reactive protein; OC=observed cases; PC=placebo-controlled.

Figure S4. Graphic Approach for Multiplicity Adjustment for Testing Primary and Key Secondary Endpoints

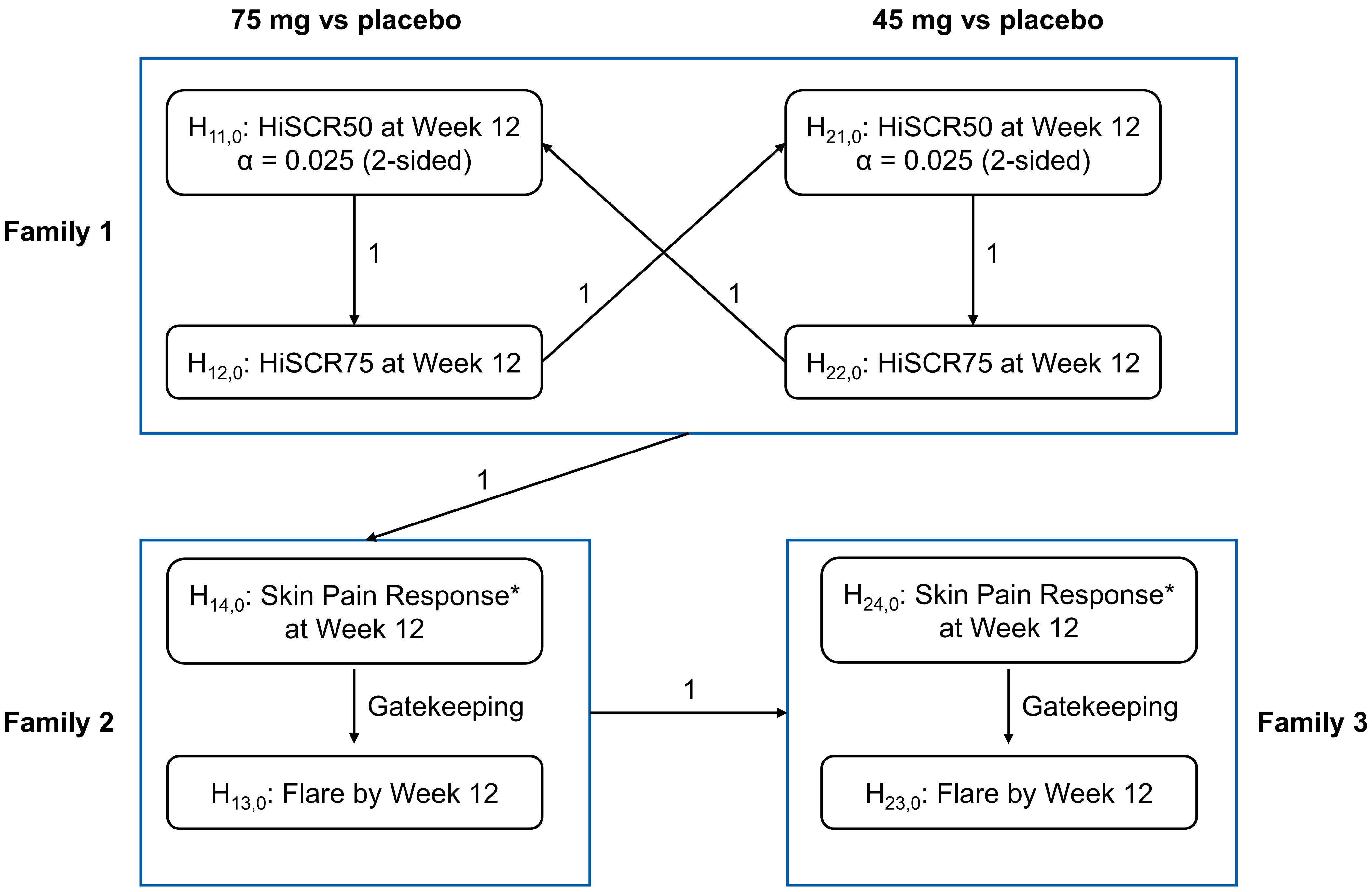

*Skin Pain Response was defined as a decrease of 3 points or more from baseline in Skin Pain NRS score for regulatory purposes in the United States and other FDA-aligned countries or Skin Pain NRS30 for regulatory purposes in the European Union and other EMA-aligned countries. EMA=European Medicines Agency; FDA=US Food and Drug Administration; HiSCR=Hidradenitis Suppurativa Clinical Response; HiSCR50/75=≥50%/≥75% reduction from baseline in the total abscess and inflammatory nodule count, with no increase from baseline in abscess or draining tunnel count; NRS=numerical rating scale; NRS30=at least 30% reduction and at least 1-unit reduction from baseline in Skin Pain NRS.

Clinical Study Protocol

INCB 54707-301

Selective **T**reatment of **O**ral **P**ovorcitinib in **H**idradenitis **S**uppurativa Study **1** (**STOP-HS1**)

A Phase 3, Double-Blind, Randomized, Placebo-Controlled, Efficacy and Safety Study of Povorcitinib (INCB054707) in Participants With Moderate to Severe Hidradenitis Suppurativa

**Product:**	**Povorcitinib**
**IND Number:**	CCI [redacted]
**EU CT Number:**	**2022-501752-29-00**
**Phase of Study:**	**3**
**Sponsor:**	**Incyte Corporation**<br>**1801 Augustine Cut-Off**<br>**Wilmington, DE 19803 USA**
**Original Protocol:**	**24 AUG 2022**
**Amendment 1:**	**10 APR 2023**
**Amendment 2:**	**01 MAY 2023**

This study will be performed in accordance with ethical principles that have their origin in the Declaration of Helsinki (Brazil 2013) and conducted in adherence to the study Protocol, applicable Good Clinical Practices, and applicable laws and country-specific regulations, including WMO (Medical Research Involving Human Participants Act) and Clinical Trials Regulation (EU) No. 536/2014, in which the study is being conducted.

The information in this document is confidential. No part of this information may be duplicated, referenced, or transmitted in any form or by any means (electronic, mechanical, photocopy, recording, or otherwise) without prior written consent.

INVESTIGATOR'S AGREEMENT

I have read the INCB 54707-301 Protocol Amendment 2 (dated 01 MAY 2023) and agree to conduct the study as outlined. I agree to maintain the confidentiality of all information received or developed in connection with this Protocol.

____

(Printed Name of Investigator)

____________________________________ ____________________________________

(Signature of Investigator) (Date)

TABLE OF CONTENTS

TITLE PAGE 1
INVESTIGATOR'S AGREEMENT 2
TABLE OF CONTENTS 3
LIST OF ABBREVIATIONS 9
1. PROTOCOL SUMMARY 13
2. INTRODUCTION 19
2.1. Background 19
2.2. Povorcitinib 20
2.2.1. Nonclinical Safety 20
2.2.2. Clinical Experience 21
2.2.2.1. Clinical Pharmacokinetics in Phase 1 Studies 21
2.2.2.2. Hidradenitis Suppurativa 22
2.3. Study Rationale 27
2.3.1. Scientific Rationale for Study Design 27
2.3.2. Justification for Doses 29
2.3.3. Justification for Placebo 30
2.4. Benefit/Risk Assessment 30
3. OBJECTIVES AND ENDPOINTS 33
4. STUDY DESIGN 38
4.1. Overall Design 38
4.2. Overall Study Duration 39
4.3. Study Termination 39
5. STUDY POPULATION 40
5.1. Inclusion Criteria 40
5.2. Exclusion Criteria 41
5.3. Lifestyle Considerations 45
5.3.1. Contraception 45
5.3.1.1. Female Participants 45
5.3.1.2. Male Participants 45
5.3.2. Meals and Dietary Restrictions 45
5.3.3. Prohibited Therapies During the Placebo-Controlled Period 46

5.4. Screen Failures 46
5.5. Replacement of Participants 46
5.6. Data Safety Monitoring Board 46
5.7. Cardiovascular Adjudication Committee 47
6. STUDY TREATMENT 47
6.1. Study Treatment Administered 47
6.2. Preparation, Handling, and Accountability 48
6.3. Measures to Minimize Bias: Randomization and Blinding 48
6.4. Study Treatment Compliance 49
[redacted]
6.5.2. Criteria for Permanent Discontinuation of Study Drug 54
6.6. Concomitant Medications and Procedures 54
6.6.1. Prior HS Therapy 54
[redacted]
6.6.6. Permitted Medications and Procedures 58
[redacted]
6.6.6.2. Wound Care Dressings 59
6.6.6.3. COVID-19 Vaccination 59
[redacted]
6.6.8. Prohibited Medications and Procedures 60
6.6.9. Lesion Rescue 61

6.6.10. Systemic Antibiotic Rescue Therapy......62
6.7. Treatment After the End of the Study......63
7. DISCONTINUATION OF STUDY TREATMENT AND PARTICIPANT WITHDRAWAL......63
7.1. Discontinuation of Study Treatment......63
7.1.1. Reasons for Discontinuation......63
7.1.2. Discontinuation Procedures......64
7.2. Participant Withdrawal From the Study......64
7.3. Lost to Follow-Up......65
8. STUDY ASSESSMENTS AND PROCEDURES......65
8.1. Administrative and General Procedures......65
8.1.1. Informed Consent Process......65
8.1.2. Screening Procedures......66
8.1.3. Interactive Response Technology Procedure......66
8.1.4. Distribution of Participant Card......67
8.1.5. Demography and Medical History......67
8.1.5.1. Demographics and Medical History......67
8.1.5.2. Disease Characteristics and Treatment History......68
CCI [redacted]
8.2. Efficacy Assessments......69
8.2.1. HS Lesion Assessment and Count......69
8.2.1.1. Hidradenitis Suppurativa Clinical Response......72
8.2.1.2. Change in HS Lesion Counts......72
8.2.1.3. International Hidradenitis Suppurativa Severity Score System......72
8.2.1.4. Flare Incidence......73
8.2.1.5. Lesion Rescue......73
8.2.2. Hurley Stage......73
8.2.3. Photography......74
8.2.4. Patient-Reported Outcomes......74
8.2.4.1. Daily Diary Patient-Reported Outcomes......74
8.2.4.2. Patient-Reported Outcomes Completed at Study Visits......75
CCI [redacted]
8.3. Safety Assessments......77

8.3.1. Adverse Events 77
8.3.2. Physical Examinations 78
8.3.3. Vital Signs 78
8.3.4. Electrocardiograms 79
8.3.5. Laboratory Assessments 79
8.3.5.1. Pregnancy Testing 81
8.3.5.2. Tuberculosis Assessment 82
8.3.5.3. HIV Testing 83
8.3.5.4. Hepatitis Testing 84
8.4. Pharmacokinetic Assessments 84
CCI [redacted]
8.6. Unscheduled Visits 85
8.7. End of Treatment and Early Termination 85
8.8. Follow-Up 86
8.8.1. Safety Follow-Up 86
9. ADVERSE EVENTS: DEFINITIONS AND PROCEDURES FOR RECORDING, EVALUATING, FOLLOW-UP, AND REPORTING 87
9.1. Definition of Adverse Event 87
9.2. Definition of Serious Adverse Event 88
9.3. Recording and Follow-Up of Adverse Events and/or Serious Adverse Events 89
9.4. Reporting of Serious Adverse Events 91
9.5. Potential Drug-Induced Liver Injury 93
CCI [redacted]
9.7. Emergency Unblinding of Treatment Assignment 93
9.8. Pregnancy 94
9.9. Warnings and Precautions 94
9.10. Product Complaints 95
9.11. Treatment of Overdose 95
10. STATISTICS 96
10.1. Sample Size Determination 96
10.2. Populations for Analysis 97
10.3. Level of Significance 97

10.4. Statistical Analyses 99
10.4.1. Primary Analysis 100
CCI [redacted]
10.4.1.2. Sensitivity Analysis 101
10.4.1.3. Subgroup Analyses 101
10.4.2. Secondary Analyses 101
CCI [redacted]
10.4.2.2. Other Secondary and Exploratory Analyses 104
10.4.3. Safety Analyses 104
10.4.4. Exploratory Analysis 105
10.4.4.1. Pharmacokinetic Analyses 105
10.4.4.2. Pharmacokinetic/Pharmacodynamic Analyses 105
10.5. Interim Analysis 105
11. SUPPORTING DOCUMENTATION AND OPERATIONAL CONSIDERATIONS 106
11.1. Investigator Responsibilities 106
11.1.1. Identification of the Coordinating Principal Investigator 107
11.2. Data Management 107
11.3. Data Quality Assurance 110
11.4. Data Privacy and Confidentiality of Study Records 110
11.5. Financial Disclosure 111
11.6. Publication Policy 111
11.7. Study and Site Closure 111
12. REFERENCES 113
APPENDIX A. INFORMATION REGARDING EFFECTIVENESS OF CONTRACEPTIVE METHODS AND DEFINITIONS 117
APPENDIX B. LIST OF STRONG SYSTEMIC CYP3A4 INHIBITORS AND STRONG/MODERATE SYSTEMIC CYP3A4 INDUCERS 119
CCI [redacted]

APPENDIX F. PROTOCOL AMENDMENT SUMMARY OF CHANGES 128

LIST OF TABLES

Table 1: Primary and Key Secondary Objectives and Endpoints 13
Table 2: Key Study Design Elements 14
Table 3: Schedule of Activities 16
Table 4: [redacted] 25
Table 5: INCB 54707-204 Summary of TEAEs (Individual TEAEs With a Cutoff ≥ 2.0% Based on Total Povorcitinib Values) 26
Table 6: Summary of Serious Treatment-Emergent Adverse Events During the Placebo-Controlled Period (ITT Population)..27
[redacted]
Table 8: Objectives and Endpoints 33
Table 9: Exclusionary Laboratory Values 44
Table 10: Study Treatment Information 47
[redacted]
Table 13: HS Lesion Definition..70
Table 14: Hurley Stages of Hidradenitis Suppurativa 74
[redacted]
Table 16: Interpretation and Management of HBV Serologic Test Results 84
Table 17: [redacted] 96
Table 18: Populations for Analysis..97
Table 19: [redacted] 100
Table 20: [redacted] 102

LIST OF FIGURES

Figure 1: Study Design Schema 15
Figure 2: Proportion of HiSCR Responders During the Placebo-Controlled Period.................24
Figure 3: Mean (SE) Change From Baseline in Skin Pain NRS During the Placebo-Controlled Period..24
[redacted]

LIST OF ABBREVIATIONS

Abbreviations and Special Terms	Definition
AE	adverse event
AESI	adverse event of special interest
ALP	alkaline phosphatase
ALT	alanine aminotransferase
AN	abscess and inflammatory nodule
ANC	absolute neutrophil count
ANdT	abscess, inflammatory nodule, and draining tunnel (formerly known as draining fistula)
aPTT	activated partial thromboplastin time
AST	aspartate aminotransferase
BCG	Bacille Calmette-Guérin
CABG	coronary artery bypass graft
CAC	cardiovascular adjudication committee
$C_{avg,ss}$	average concentration of study drug at steady state
CI	confidence interval
C_{max}	maximum observed plasma or serum concentration
$C_{max,ss}$	maximum concentration of study drug at steady state
CMH	Cochran-Mantel-Haenszel
COVID-19	coronavirus disease 2019
CPK	creatine phosphokinase
CRO	contract research organization
CSR	Clinical Study Report
CT	computed tomography
$C_{tau,ss}$	trough concentration of study drug at steady state
CTCAE	Common Terminology Criteria for Adverse Events
CXR	chest x-ray
CYP3A4	cytochrome P450 3A4 enzyme
DILI	drug-induced liver injury
DLQI	Dermatology Life Quality Index
DNA	deoxyribonucleic acid
DSMB	Data Safety Monitoring Board
ECG	electrocardiogram
ECI	event of clinical interest
eCOA	electronic clinical outcome assessment

Abbreviations and Special Terms	Definition
eCRF	electronic case report form
EDC	electronic data capture
eDiary	electronic diary
EMA	European Medicines Agency
EOS	end of study
EOT	end of treatment
EOT1	end of treatment during the placebo-controlled period
EOT2	end of treatment during the open-label extension period
E-R	exposure-response
ET	early termination
CCI [redacted]	[redacted]
[redacted]	[redacted]
ET1	early termination during the placebo-controlled period
ET2	early termination during the open-label extension period
EXT	extension
FACIT-F	Functional Assessment of Chronic Illness Therapy – Fatigue
FDA	Food and Drug Administration
FSH	follicle-stimulating hormone
GCP	Good Clinical Practice
GDPR	General Data Protection Regulation
GGT	gamma-glutamyl transferase
GM-CSF	granulocyte-macrophage colony-stimulating factor
HBcAb	hepatitis B core antibody
HBsAb	hepatitis B surface antibody
HBsAg	hepatitis B surface antigen
HBV	hepatitis B virus
HCV	hepatitis C virus
HCVAb	hepatitis C virus antibody
HDL	high-density lipoprotein
HIPAA	Health Insurance Portability and Accountability Act
HiSCR	Hidradenitis Suppurativa Clinical Response
CCI [redacted]	[redacted]
HIV	human immunodeficiency virus
HRT	hormone replacement therapy
HS	hidradenitis suppurativa

Abbreviations and Special Terms	Definition
hsCRP	high-sensitivity C-reactive protein
IB	Investigator's Brochure
IC_{50}	concentration that results in 50% inhibition
ICF	informed consent form
ID	identification
IEC	independent ethics committee
IGRA	interferon gamma release assay
IHS4	International Hidradenitis Suppurativa Severity Score System
IL	interleukin
INR	international normalized ratio
IRAK	interleukin-1 receptor–associated kinase
IRB	institutional review board
IRT	interactive response technology
ITT	intent-to-treat
JAK	Janus kinase
LDL	low-density lipoprotein
LTE	long-term extension
MACE	major adverse cardiovascular event
MCP-1	monocyte chemoattractant protein 1
MDRD	Modification of Diet in Renal Disease
MedDRA	Medical Dictionary for Regulatory Activities
MMRM	mixed model for repeated measures
MRI	magnetic resonance imaging
NIH	National Institutes of Health
NRI	nonresponder imputation
NRS	Numeric Rating Scale
NSAID	nonsteroidal anti-inflammatory drug
NYHA	New York Heart Association
OLE	open-label extension
PC	placebo-controlled
PCR	polymerase chain reaction
PD	pharmacodynamic(s)
PDE	phosphodiesterase
PK	pharmacokinetic(s)
PMDA	Pharmaceuticals and Medical Devices Agency

Abbreviations and Special Terms	Definition
PP	per protocol
PPD	purified protein derivative
PPI	proton pump inhibitor
PRO	patient-reported outcome
PT	prothrombin time
QALY	quality-adjusted life year
QD	once daily
QoL	quality of life
RNA	ribonucleic acid
RSI	Reference Safety Information
SAE	serious adverse event
SAP	Statistical Analysis Plan
SARS-CoV-2	severe acute respiratory syndrome coronavirus 2
SE	standard error
SoA	schedule of activities
SOP	standard operating procedure
SpO_2	oxygen saturation measured by pulse oximetry
STAT	signal transducer and activator of transcription
STD	standard deviation
$t_{½}$	half-life of study drug
TB	tuberculosis
TEAE	treatment-emergent adverse event
Th	T-helper
t_{max}	time to maximum concentration
$t_{max,ss}$	time to achieve maximum concentration of study drug at steady state
TNF	tumor necrosis factor
TYK2	tyrosine kinase 2
ULN	upper limit of normal
VAS	visual analog scale
WBC	white blood cell
WOCBP	women of childbearing potential
WONCBP	women of nonchildbearing potential
CCI [redacted]	[redacted]

1. PROTOCOL SUMMARY

Protocol Title:

Selective **T**reatment of **O**ral **P**ovorcitinib in **H**idradenitis **S**uppurativa Study **1** (**STOP-HS1**)

A Phase 3, Double-Blind, Randomized, Placebo-Controlled, Efficacy and Safety Study of Povorcitinib (INCB054707) in Participants With Moderate to Severe Hidradenitis Suppurativa

Protocol Number: INCB 54707-301

Objectives and Endpoints:

Table 1 presents the primary and key secondary objectives and endpoints.

Table 1: Primary and Key Secondary Objectives and Endpoints

Objectives	Endpoints
**Primary**	
To evaluate the efficacy of povorcitinib.	• Proportion of participants who achieve Hidradenitis Suppurativa Clinical Response (HiSCR) at Week 12.<br>− HiSCR is defined as at least a 50% reduction from baseline in the total abscess and inflammatory nodule count, with no increase from baseline in abscess or draining tunnel count.
**Key Secondary**	
To further evaluate the efficacy of povorcitinib.	• Proportion of participants who achieve Hidradenitis Suppurativa Clinical Response 75 (HiSCR75) at Week 12.<br>− HiSCR75 is defined as at least a 75% reduction from baseline in the total abscess and inflammatory nodule count, with no increase from baseline in abscess or draining tunnel count.
To evaluate the effect of povorcitinib in reducing the incidence of flares.	• Proportion of participants with flare by Week 12.<br>− Participants who experience at least 1 flare over 12 weeks; flare is defined as at least a 25% increase in the total abscess and inflammatory nodule count with a minimum increase of 2 relative to baseline.
To evaluate the effect of povorcitinib in decreasing skin pain.	• Key secondary endpoint for regulatory purposes in US/FDA/aligned countries:<br>Proportion of participants with a ≥ 3-point decrease in Skin Pain Numeric Rating Scale (NRS) score at Week 12 among participants with baseline Skin Pain NRS score ≥ 3.<br>− Participants with a Skin Pain score of at least 3 at baseline and who experience at least a 3-point decrease in Skin Pain score at Week 12, relative to baseline. Skin Pain is an 11-point NRS, ranging from 0 (no skin pain) to 10 (worst skin pain).<br>• Key secondary endpoint for regulatory purposes in EU/EMA/aligned countries:<br>Proportion of participants who achieve Skin Pain NRS30 at Week 12 among participants with baseline Skin Pain NRS score ≥ 3.<br>− Participants with a Skin Pain score of at least 3 at baseline and who achieve at Week 12 Skin Pain NRS30, defined as at least a 30% reduction and at least 1-unit reduction from baseline in the Skin Pain NRS.

Overall Design:

Table 2 presents the key study design elements. Further study details are presented after the table.

Table 2: Key Study Design Elements

**Study Phase**	Phase 3
**Clinical Indication**	Treatment of patients 18 years and older with moderate to severe HS who have a history of inadequate response, intolerance, or contraindication to an adequate course of conventional systemic therapy for HS.
**Population**	Men or women ≥ 18 years of age who have had a diagnosis of moderate to severe HS (Hurley Stage II or III) for at least 3 months and who have a documented history of inadequate response to at least a 3-month course of at least 1 conventional systemic therapy (oral antibiotic or biologic drug) for HS (or demonstrated intolerance to, or have a contraindication to, a conventional systemic therapy for treatment of their HS), with HS lesions in at least 2 distinct anatomical areas and total abscess and inflammatory nodule count of at least 5.
**Number of Participants**	Approximately 600 participants will be randomized 1:1:1 to povorcitinib ([redacted] or [redacted] mg) or placebo QD.
**Study Design**	This is a Phase 3, multicenter, randomized, parallel-group, PC, double-blind study to evaluate the efficacy and safety of povorcitinib ([redacted] or [redacted] mg QD) over a 12-week period, followed by a 42-week double-blind EXT period (povorcitinib [redacted] or [redacted] mg QD).<br>[redacted]
**Estimated Duration of Study Participation**	Screening: up to 28 days<br>PC period (double-blind): 12 weeks<br>EXT period (double-blind): 42 weeks<br>Safety follow-up: 30 days after last dose of study drug<br>Total: up to approximately 62 weeks (≈14 months)
**DSMB**	Yes (external)
**Cardiovascular Adjudication Committee**	Yes (external)
**Coordinating Principal Investigator**	Joslyn Sciacca Kirby, MD, MS, MEd

Treatment Groups and Duration:

Approximately 600 adult participants with moderate to severe HS (Hurley Stage II or III) for at least 3 months, HS lesions in ≥ 2 distinct anatomical areas, total abscess and inflammatory nodule count ≥ 5, and a history of inadequate response to at least a 3-month course of at least 1 conventional systemic therapy (oral antibiotic or biologic drug) for HS (or demonstrated intolerance to, or have a contraindication to, a conventional systemic therapy for treatment of their HS) will be randomized 1:1:1 to povorcitinib ([redacted] mg or [redacted] mg) or placebo (see Figure 1). During the double-blind, PC period, participants will be treated with orally administered povorcitinib ([redacted] or [redacted] mg) or placebo QD for 12 weeks. At Week 12, participants will enter the double-blind EXT period, which will last for 42 weeks. During the EXT period, participants randomized to povorcitinib at baseline will remain at their respective dose level ([redacted] or [redacted] mg QD), while participants randomized to placebo will receive povorcitinib [redacted] or [redacted] mg QD (1:1 ratio). Further information on the study design is presented in Section 4.1.

Table 3 presents the SoA, including the PC and EXT periods. Adherence to the study design requirements, including those specified in the SoA, is essential and required for study conduct.

The COVID-19 global pandemic may present challenges to the normal conduct of this study (including AE and laboratory assessments), requiring the outline of potential mitigation strategies described in Appendix E.

Participants who complete the 54-week treatment in this study may be eligible to participate in a separate extension study to further evaluate the safety and efficacy of the investigational product.

Figure 1: Study Design Schema

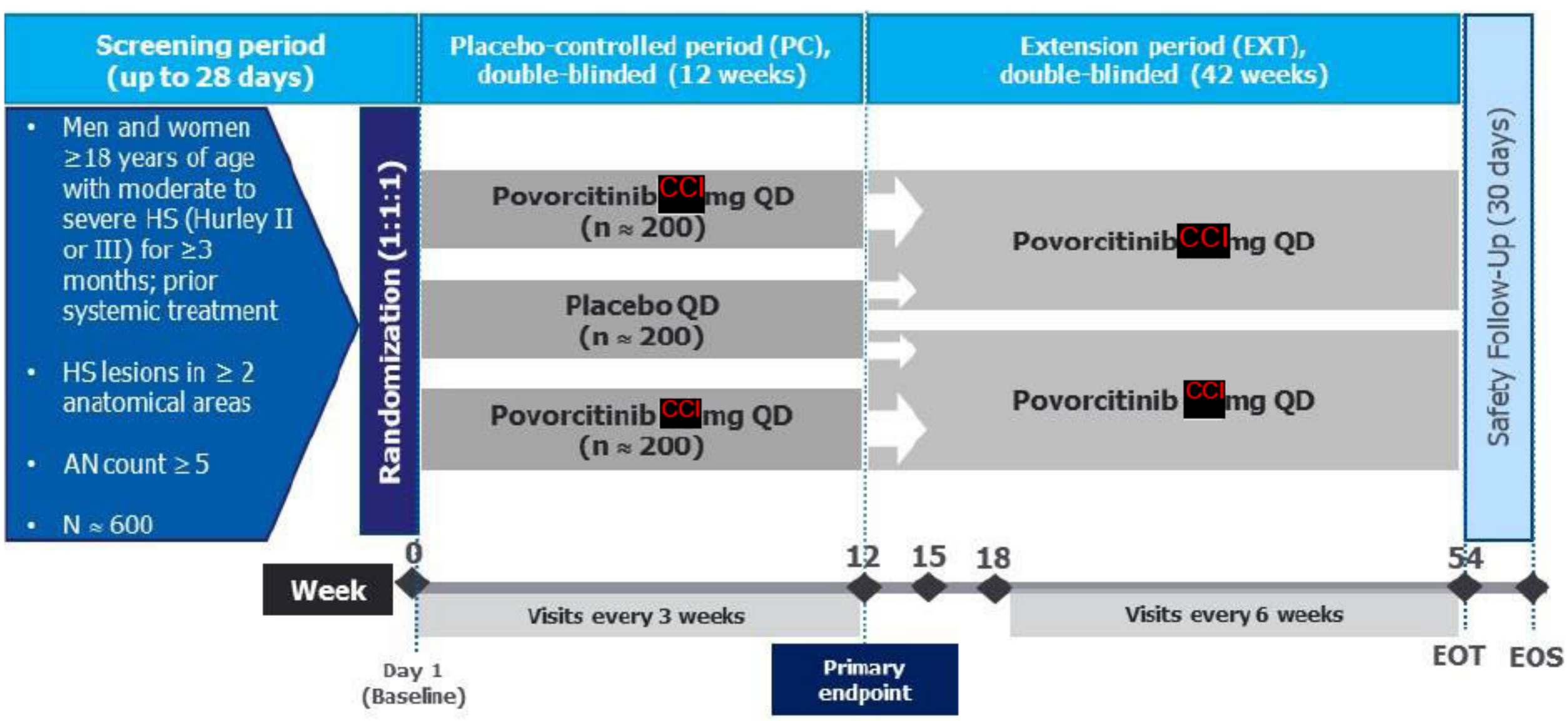

Table 3: Schedule of Activities

	Screening	PC Period (12 Weeks)					EXT Period (42 Weeks)						Safety Follow-Up	
**Visit**	**Days −28 to −1**	**Baseline (Day 1)**	**Wk 3 (± 3 d)**	**Wk 6 (± 3 d)**	**Wk 9 (± 3 d)**	**Wk 12 (± 3 d) EOT1 (or ET1)**	**Wk 15 (± 3 d)**	**Wk 18 (± 3 d)**	**Wk 24 (± 5 d)**	**Wk 30, Wk 36 (± 5 d)**	**Wk 42, Wk 48 (± 5 d)**	**Wk 54 (± 5 d) EOT2 (or ET2)**	**30 d (+ 7 d) After EOT or ET (EOS)**	**Section**
**Administrative procedures**														
Informed consent	X													8.1.1
Inclusion/exclusion criteria	X	X												5.1/5.2
Demographic data, medical history, and HS medical and treatment history	X													8.1.5
Prior/concomitant medications	X	X	X	X	X	X	X	X	X	X	X	X	X	6.6
Contact IRT	X	X	X	X	X	X	X	X	X	X	X	X		8.1.3
Randomization		X												8.1.2
Dispense study drug		X	X	X	X	X	X	X	X	X	X			6.1
Distribute trial participant card	X													8.1.4
Collect study drug and review drug compliance			X	X	X	X	X	X	X	X	X	X		6.4
Dispense eDiary (handheld device) and instruct participant on use	X													8.2.4
Collect eDiary (handheld device) from participant												X		8.2.4
Follow-up phone call for daily diary compliance (1 week before baseline visit and 1 week before Week 12 visit)	X					X								8.2.4.1.1.1
Study drug administration diary		Study drug administration diary to be completed daily by participant (baseline through Week 54)												6.1
Review diary compliance		X	X	X	X	X	X	X	X	X	X	X		8.2.4.1.1.2
[redacted]						X						X		[redacted]
[redacted]		X	X	X	X	X	X	X	X	X	X	X	X	[redacted]

Table 3: Schedule of Activities (Continued)

	Screening	PC Period (12 Weeks)					EXT Period (42 Weeks)						Safety Follow-Up	
**Visit**	**Days −28 to −1**	**Baseline (Day 1)**	**Wk 3 (± 3 d)**	**Wk 6 (± 3 d)**	**Wk 9 (± 3 d)**	**Wk 12 (± 3 d) EOT1 (or ET1)**	**Wk 15 (± 3 d)**	**Wk 18 (± 3 d)**	**Wk 24 (± 5 d)**	**Wk 30, Wk 36 (± 5 d)**	**Wk 42, Wk 48 (± 5 d)**	**Wk 54 (± 5 d) EOT2 (or ET2)**	**30 d (+ 7 d) After EOT or ET (EOS)**	**Section**
**Safety assessments**														
AE assessments (If an AE is noted, relevant body systems should be physically examined)	X	X	X	X	X	X	X	X	X	X	X	X	X	8.3.1
Contraception check	X	X	X	X	X	X	X	X	X	X	X	X	X	5.3.1
Comprehensive physical examination	X	X				X						X		8.3.2
Height	X													8.3.2
Weight	X	X				X						X		8.3.2
Vital signs	X	X	X	X	X	X	X	X	X	X	X	X	X	8.3.3
12-lead ECG	X					X						X		8.3.4
**Efficacy assessments**														
Lesion assessment and count	X	X	X	X	X	X	X	X	X	X	X	X	X	8.2.1
Hurley stage assessment	X	X				X			X			X		8.2.2
Photography (subset of sites/participants)		X				X			X					8.2.3
**QoL assessments – daily diaries**														
Skin Pain NRS	PRO daily diary (screening through Week 54)													8.2.4.1.2
CCI [redacted]	PRO daily diary (screening through Week 54)													CCI [redacted]
[redacted]	PRO daily diary (screening through Week 54)													[redacted]
**QoL assessments and health economics (conducted at study site before any other assessments)**														
CCI [redacted]														
DLQI		X	X	X	X	X	X	X	X	X	X	X	X	8.2.4.2.2
FACIT-F		X	X	X	X	X	X	X	X	X	X	X	X	8.2.4.2.3
CCI [redacted]														

Table 3: Schedule of Activities (Continued)

	Screening	PC Period (12 Weeks)					EXT Period (42 Weeks)						Safety Follow-Up	
**Visit**	**Days −28 to −1**	**Baseline (Day 1)**	**Wk 3 (± 3 d)**	**Wk 6 (± 3 d)**	**Wk 9 (± 3 d)**	**Wk 12 (± 3 d) EOT1 (or ET1)**	**Wk 15 (± 3 d)**	**Wk 18 (± 3 d)**	**Wk 24 (± 5 d)**	**Wk 30, Wk 36 (± 5 d)**	**Wk 42, Wk 48 (± 5 d)**	**Wk 54 (± 5 d) EOT2 (or ET2)**	**30 d (+ 7 d) After EOT or ET (EOS)**	**Section**
**Laboratory assessments**														
Serum FSH (WONCBP only)	X													8.3.5.1.1
Serum pregnancy test (WOCBP only)	X												X	8.3.5.1.2
Urine pregnancy test (WOCBP only; conducted at study site)		X	X	X	X	X	X	X	X	X	X	X		8.3.5.1.2
TB assessment	X													8.3.5.2
HIV testing	X													8.3.5.3
Hepatitis testing	X													8.3.5.4
HBV DNA (for required participants)	X					X			X	X (Wk 36)	X (Wk 48)	X		8.3.5.4.1
Chemistry and hematology	X	X	X	X	X	X	X	X	X	X	X	X	X	8.3.5
Coagulation panel	X	X				X						X	X	8.3.5
Urinalysis	X	X				X			X			X	X	8.3.5
Nicotine metabolite screen (urine)		X										X		8.3.5
Lipid panel (fasting required)		X		X		X		X	X			X	X	8.3.5
hsCRP		X	X	X	X	X	X	X	X	X	X	X	X	8.3.5
[redacted]														
[redacted]			X			X	X							8.4
[redacted]			X											8.4
[redacted]														
[redacted]		X	X			X								8.5

2. INTRODUCTION

Povorcitinib (also known as INCB054707) is a novel, potent, oral small-molecule JAK1 inhibitor under evaluation in HS, vitiligo, and prurigo nodularis clinical trials. Janus kinase signaling regulates several proinflammatory pathways and has been well recognized as a key driver for numerous skin diseases. JAK inhibitors have been approved in various inflammatory disorders such as rheumatoid arthritis, atopic dermatitis, and alopecia areata. JAK-mediated proinflammatory cytokines persist in skin lesions of patients with HS, and results from a recent Phase 2b study with povorcitinib, demonstrating a dose-dependent efficacy in HS, support further investigation of this molecule in Phase 3 studies.

2.1. Background

Hidradenitis suppurativa, also designated as acne inversa, is a chronic inflammatory skin condition characterized by painful nodules and abscesses in early stages and draining tunnels (fistulas) and irreversible tissue damage and scarring in more severe disease (van Straalen et al 2022b). Cutaneous changes seem to start around hair follicles and involve activation of cells of the innate and adaptive immune systems, with pivotal roles for proinflammatory cytokines such as TNF, IL-1β, and IL-17 (Frew et al 2019, Zouboulis et al 2022). A genetic predisposition, smoking, obesity, and hormonal factors are established etiologic factors for HS (Zouboulis et al 2020a).

Typically starting in early adulthood and with a median delay of diagnosis of approximately 7 years, HS affects the intertriginous skin (eg, axillary, inguinal, gluteal and perianal body sites) and is associated with numerous systemic comorbidities, such as Crohn disease, metabolic syndrome, and type 2 diabetes mellitus (Sabat et al 2020). Hidradenitis suppurativa has profound negative effects on patients' QoL, which often culminate in social withdrawal, unemployment, depression, and suicidal thoughts (Matusiak 2020). The estimated prevalence of HS varies substantially but most likely ranges between 0.7% and 1.2% in the European and US population (Nguyen et al 2021); the female-to-male ratio in Western populations is approximately 3:1, but the reverse is found in other parts of the world, such as in Japan and South Korea (Chu et al 2021, Ingram 2020). The role of race in the pathogenesis of HS is not well understood; in the US, those of African American and biracial ancestry appear to have a higher prevalence of HS, and some data also suggest that African American patients may have earlier disease onset than the remainder of the population (Shao et al 2022).

Adalimumab, a monoclonal antibody targeting the anti-inflammatory cytokine TNF-α, is the only therapy approved for HS treatment and is recommended for the treatment of moderate to severe HS (Hurley Stages II and III). Other biologics, including another TNF-α inhibitor, infliximab, may also be used for the treatment of HS, based on North America and EU guidelines (Hendricks et al 2021). Studies have reported significant dropout rates in patients being treated with adalimumab, primarily due to lack of efficacy (ranging from 27% to 50%; Frew et al 2020). A recent study also demonstrated that the median time to discontinuation from adalimumab was 36 weeks, which was comparable to other biologics used off-label for HS; this relatively short drug survival rate may be a result of the suboptimal efficacy of biologics and the higher inflammatory load in HS compared to other autoimmune diseases (Ring et al 2022).

Additionally, the antagonism of a single proinflammatory cytokine may not be sufficient in many patients with HS.

Other treatments, although not approved, are also used in HS and discussed in the treatment guidelines. Systemic antibiotics are the most commonly recommended therapy for treatment of moderate to severe HS, typically for at least 12 weeks (or as long-term maintenance or adjunct therapy). Other systemic oral drugs are also used as second- or third-line therapies, such as metformin, spironolactone, and retinoids (Hendricks et al 2021). There is not a strong level of scientific evidence on the use of antibiotics and the other systemic therapies in HS and data from PC studies are limited (Frew et al 2019), thus demonstrating a substantial unmet need for more efficacious therapies, evaluated in well-controlled clinical trials, and with potential to target diverse immunomodulatory mechanisms.

2.2. Povorcitinib

Povorcitinib is a novel, potent, and selective oral small-molecule inhibitor of the JAK class, with marked selectivity for JAK1. Povorcitinib potently inhibits JAK1 (IC_{50} [redacted] nM), with approximately [redacted] to [redacted]-fold selectivity over the other JAK family members (JAK2, JAK3, and TYK2). Povorcitinib also demonstrated high potency in JAK1-dependent cell-based assays (IC_{50} [redacted] nM) and in other cell-based assays relevant to the pathogenesis of Th1/Th17 lymphocyte-mediated diseases by inhibiting the production of proinflammatory factors (eg, IL-17, MCP-1) induced by other cytokines, such as IL-23 and IL-6, with IC_{50} values of [redacted] nM. In contrast, povorcitinib showed significantly less inhibition (IC_{50} [redacted] nM) in cell-based assays dependent on JAK2 (eg, thrombopoietin- or GM-CSF–stimulated STAT phosphorylation), suggesting JAK1 selectivity in both biochemical and cellular assays.

Povorcitinib oral bioavailability is ≈100%, with a median time to maximum concentration of ≈2 to 4 hours, and a mean terminal half-life of ≈27 to 35 hours. The primary clearance pathway is hepatic, via metabolism through CYP3A4. Renal excretion is minimal, which is consistent with its high plasma protein binding.

Additional information is presented in the IB.

2.2.1. Nonclinical Safety

[redacted]

[redacted]

2.2.2. Clinical Experience

2.2.2.1. Clinical Pharmacokinetics in Phase 1 Studies

Single doses of povorcitinib up to 405 mg and multiple doses of povorcitinib up to 120 mg QD for up to 10 days have been evaluated in 2 Phase 1 studies in healthy participants. The most common TEAE was mild to moderate headache in [redacted]% of participants who received povorcitinib in the multiple-dose study; the events resolved spontaneously following discontinuation of povorcitinib. Furthermore, decreases in platelet counts were observed among participants treated with povorcitinib at doses [redacted] mg QD. The magnitude of the decrease was larger for participants treated with [redacted] mg QD compared with those who received [redacted] mg QD, suggesting a dose-related trend. Of note, the participants with a treatment-emergent decrease in platelet counts still had this laboratory parameter within the normal range.

A population PK model demonstrated that the central volume of distribution was allometrically scaled with body weight. The typical value of drug clearance appears to be [redacted]% lower in females in comparison to males and [redacted]% higher and [redacted]% lower in Black and Asian participants, respectively, in comparison to the reference group of White participants and participants of other races. Overall, the gender and racial differences in PK were not considered significant.

A drug-drug interaction study in healthy volunteers was evaluated in 3 separate cohorts. Following coadministration with itraconazole (a strong CYP3A4 inhibitor), the total exposure was [redacted]% greater than when povorcitinib was administered alone; C_{max} and t_{max} remained similar, while $t_{½}$ increased from [redacted] to [redacted] hours. Following coadministration with rifampin (a strong CYP3A4 inducer), the total exposure was [redacted]% lower than when povorcitinib was administered alone, C_{max} was [redacted]% lower, t_{max} remained unchanged, and $t_{½}$ decreased from [redacted] to [redacted] hours. Following coadministration with esomeprazole (a PPI), the total exposure was comparable to povorcitinib administered alone; C_{max} was [redacted]% lower, and t_{max} and $t_{½}$ remained

unchanged. These results indicate that coadministration of povorcitinib with strong CYP3A4 inhibitors or strong/moderate inducers should be avoided, while PPIs or other pH-modifying agents do not have a substantial effect.

A study to evaluate the safety, tolerability, and PK of povorcitinib ([redacted] or [redacted] mg QD) in healthy male Japanese participants was conducted. Povorcitinib was generally safe and well tolerated, with [redacted] SAE ([redacted]; not drug related) and [redacted] Grade 3 TEAEs ([redacted]). Although the estimated PK exposures in Japanese participants for [redacted] mg QD will be higher by up to [redacted]% (in terms of peak concentration) and [redacted]% in terms of area under the plasma concentration-time curve than that of the non-Japanese participants receiving the same dose, the distribution range of exposure in Japanese participants is within the range observed in non-Japanese participants; therefore, this PK difference is not clinically meaningful.

2.2.2.2. Hidradenitis Suppurativa

2.2.2.2.1. INCB 54707-202

INCB 54707-202 was an open-label study that evaluated the safety and efficacy of povorcitinib 15 mg QD administered over 8 weeks in 10 adult participants with moderate to severe HS. There were no deaths or serious TEAEs in this study. One participant (10.0%) discontinued treatment with povorcitinib due to TEAEs. At least 1 TEAE was reported for 7 participants (70.0%). The most frequently reported TEAE in this study was upper respiratory tract infection. All TEAEs reported in this study were mild or moderate in severity.

Three participants (43%) achieved HiSCR at Week 8. The mean (STD) change from baseline in IHS4 was −2.1 (7.5) at Week 8. At Week 8, the mean (STD) change from baseline in [redacted] was −15.3 (40.9). At Week 8, the mean (STD) change from baseline in worst skin pain score was −2.0 (1.8). Further results of this study have been reported by Alavi et al (2022).

2.2.2.2.2. INCB 54707-203

INCB 54707-203 was a randomized, PC study that evaluated the safety and efficacy of povorcitinib (30, 60, or 90 mg QD) administered over 8 weeks in 35 adult participants with moderate to severe HS. There were no deaths, serious TEAEs, or TEAEs leading to discontinuation of treatment. At least 1 TEAE was reported for 25 participants (71.4%). The most frequently reported TEAE in this study was fatigue.

In this study, 17 participants (65%) receiving povorcitinib (5 [56%] on 30 mg QD, 5 [56%] on 60 mg QD, 7 [88%] on 90 mg QD) versus 4 participants (57%) receiving placebo achieved HiSCR at Week 8. For IHS4, the mean (STD) changes from baseline at Week 8 were −9.4 (6.8), −21.4 (21.4), and −16.1 (22.9) for participants treated with 30 mg, 60 mg, and 90 mg, respectively, versus −10.7 (21.0) for the placebo group. The mean (STD) changes from baseline in [redacted] were −34.3 (41.7), −39.0 (30.8), and −28.0 (21.4) for participants treated with 30, 60, or 90 mg, respectively, at Week 8 versus 3.4 (16.1) for placebo. At Week 8, the mean (STD) changes from baseline in worst skin pain score were −2.2 (2.2), −1.4 (1.4), and −3.1 (3.3) for participants treated with 30, 60 and 90 mg versus 0.3 (2.8) for placebo. Further results of this study have been reported by Alavi et al (2022).

2.2.2.2.3. INCB 54707-204

INCB 54707-204 is a randomized, PC, dose-range (povorcitinib 15, 45, or 75 mg) finding study in 209 adult participants with HS (Hurley Stage I, II, or III) with a total abscess and inflammatory nodule count ≥ 5. In this study, QD doses of study drug were administered for up to 16 weeks, followed by a 36-week OLE period and a 48-week LTE period. The OLE/LTE periods of this study were still ongoing at the time of the latest data cutoff date (15 DEC 2021).

2.2.2.2.3.1. Efficacy Summary

Povorcitinib demonstrated a dose-dependent improvement in HS lesions and on participants' QoL. Overall:

- HiSCR was achieved in [redacted]% and [redacted]% of participants in the povorcitinib [redacted] and [redacted] mg groups, respectively, as compared with [redacted]% of participants in the placebo group at Week 12 and in [redacted]% and [redacted]% of participants in the povorcitinib [redacted] and [redacted] mg groups, respectively, as compared to [redacted]% of participants in the placebo group at Week 16 (see Figure 2).
- Improvement in QoL was observed as early as Week 2 for the povorcitinib [redacted] and [redacted] mg groups as demonstrated via Skin Pain NRS (see Figure 3), as well as for [redacted] Itch NRS, DLQI, and FACIT-F (data not shown).
- Although comparable across the majority of efficacy and QoL endpoints, participants in the povorcitinib [redacted] mg group appeared to have a faster and trending better response than those in the povorcitinib [redacted] mg group, as shown for the achievement of clinical response (HiSCR) for participants with more severe disease (ANdT ≥ 11), improvement in skin pain, decrease in analgesic use, decrease in draining tunnel count, decrease in Itch NRS, and improvement in FACIT-F scores, as summarized in Table 4.

Figure 2: Proportion of HiSCR Responders During the Placebo-Controlled Period

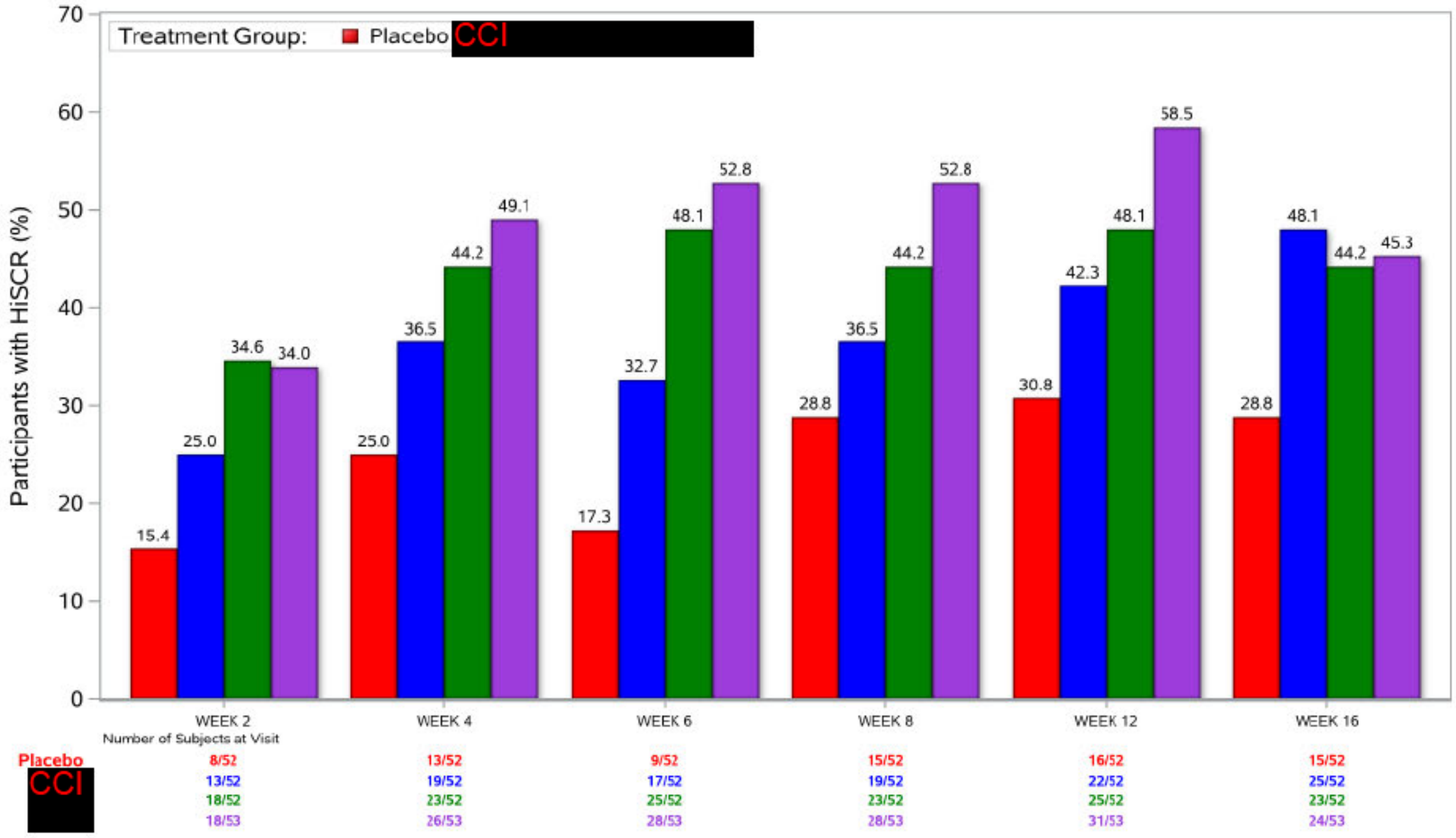

Figure 3: Mean (SE) Change From Baseline in Skin Pain NRS During the Placebo-Controlled Period

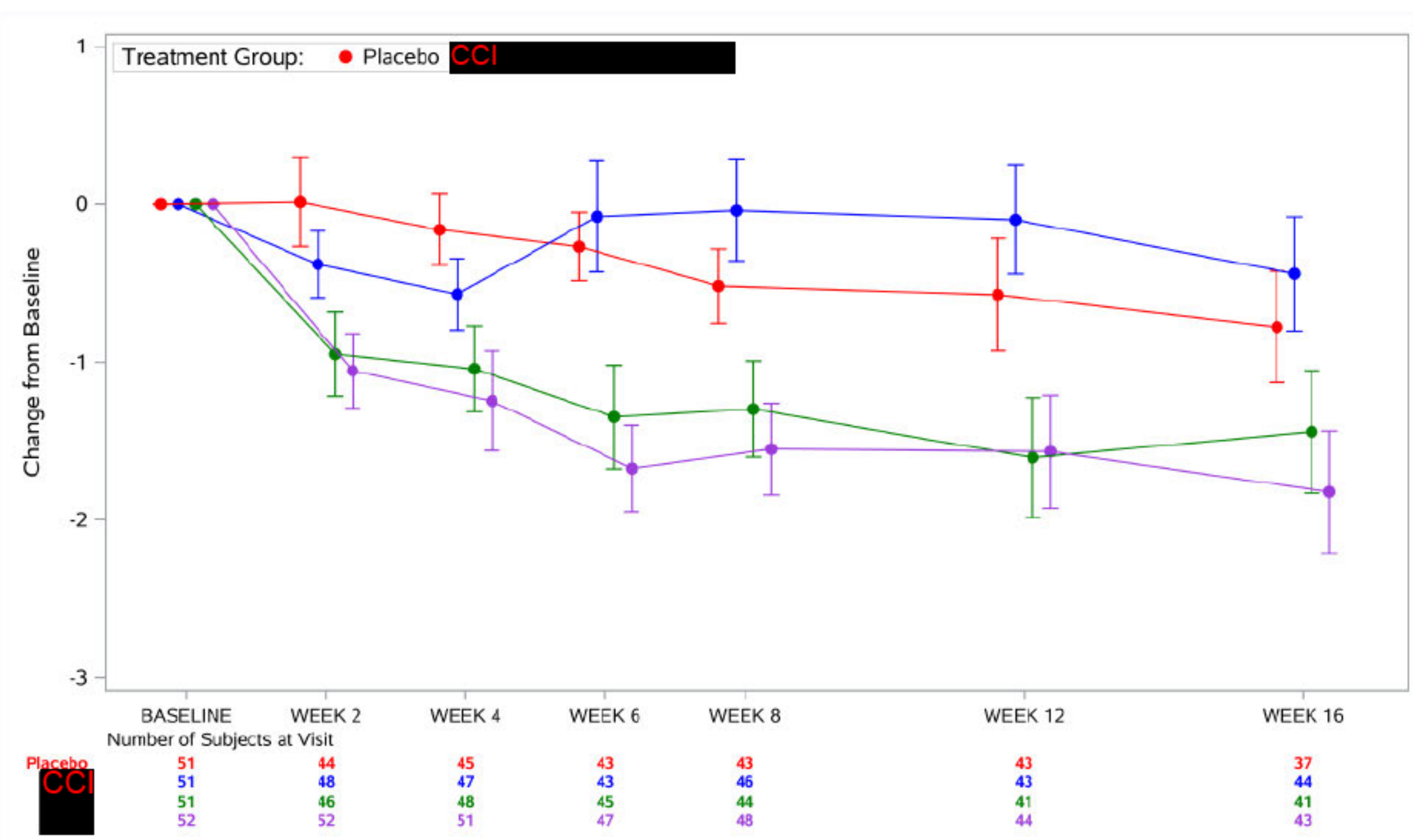

Table 4: [redacted]

Parameter	Visit	Placebo	[redacted]	[redacted]	[redacted]
Proportion of participants with ANdT count ≥ 11 at baseline achieving HiSCR	Week 4<br>Week 12<br>Week 16	[redacted]			
Proportion of participants achieving Skin Pain NRS30	Week 4<br>Week 12<br>Week 16				
Proportion of participants with analgesic use	Baseline<br>Week 4<br>Week 12<br>Week 16				
Mean draining tunnel count for participants with ≥ 1 draining tunnel at baseline	Baseline<br>Week 4<br>Week 12<br>Week 16				
Proportion of participants achieving ≥ 4-point reduction in Itch NRS score from baseline	Week 4<br>Week 12<br>Week 16				
Mean change in FACIT-F score from baseline	Week 4<br>Week 8<br>Week 16				

2.2.2.2.3.2. Safety Summary

At the time of the primary completion data cutoff date (15 DEC 2021), all participants had either completed ([redacted]%]) or discontinued from treatment ([redacted]%]) in the 16-week PC period. Participants who completed treatment in the PC period ([redacted]%]) entered the 36-week OLE period; of these, [redacted] participants ([redacted]%) completed treatment in the OLE period and started the 48-week LTE period, and [redacted] participants ([redacted]%) discontinued treatment in the OLE period.

Overall, povorcitinib was safe and well tolerated in adult participants with HS, with a low discontinuation rate due to TEAEs and [redacted] serious or Grade 3 or higher TEAEs. [redacted]

[redacted]. In the ongoing OLE period, the [redacted] Nonetheless and despite the longer treatment duration, [redacted].

The percentage of participants who experienced a TEAE in the placebo and povorcitinib [redacted] and [redacted] mg groups was [redacted]%, [redacted]%, [redacted]%, and [redacted]%, respectively; in the same groups, the percentage of participants who had an SAE was [redacted]%, [redacted]%, [redacted]%, and [redacted]%, respectively. The TEAEs (with a cutoff ≥ 2.0% based on total povorcitinib values) and SAEs are respectively shown in Table 5 and Table 6. Overall, no clear dose-dependent safety trend was observed across the 3 doses of povorcitinib.

Table 5: INCB 54707-204 Summary of TEAEs (Individual TEAEs With a Cutoff ≥ 2.0% Based on Total Povorcitinib Values)

		Povorcitinib			
**Parameter**	**Placebo (n = [redacted])**	[redacted]			**Total (n = [redacted])**
Number (%) of participants with TEAEs	[redacted]%)	[redacted]			[redacted]%)
TEAE grade					
Grade 1	[redacted]				
Grade 2					
Grade 3					
Grade 4					
MedDRA preferred term					
Fatigue					
Headache					
Diarrhea					
Nausea					
Acne					
Nasopharyngitis					
Vomiting					
COVID-19					
Constipation					
Decrease appetite					
Upper respiratory tract infection					
Back pain					
Folliculitis					
Fungal infection					
Urinary tract infection					

Table 6: Summary of Serious Treatment-Emergent Adverse Events During the Placebo-Controlled Period (ITT Population)

Parameter	Placebo (N = 52)	Povorcitinib Treatment Group	
		[redacted]	Total (N = 155)
Number (%) of participants who had a serious TEAE	[redacted]		
Fall			
Intervertebral disc protrusion			
Pneumonia			
Pulmonary embolism			
Rib fracture			
Abscess limb			
Atrial fibrillation			
Bacterial infection			
Cholecystitis			

2.3. Study Rationale

2.3.1. Scientific Rationale for Study Design

[redacted] Povorcitinib, an oral JAK1 inhibitor, was evaluated in 2 Phase 2 trials (15, 30, 60, and 90 mg QD) in 45 participants and demonstrated proof of concept for JAK1 inhibition in HS by showing lesion count decrease, improvement in disease-specific PROs, and changes in disease-associated serum biomarkers (Alavi et al 2022). A third Phase 2 study with povorcitinib (15, 45, and 75 mg QD) in 209 participants further demonstrated a dose-dependent clinical response across lesion count decrease, HiSCR, and improvement in PROs, such as in Skin Pain NRS, Itch NRS, [redacted] DLQI, and FACIT-F (see Section 2.2.2.2.3). The collective efficacy and safety results suggest that povorcitinib represents a promising therapeutic agent in HS and supports further investigation of this molecule in Phase 3 studies.

The objective of this study is to confirm the safety and efficacy profile of povorcitinib in a larger population of participants with HS as part of Phase 3 studies. The population to be studied comprises participants 18 years of age and older who have had a diagnosis of moderate to severe HS (Hurley Stage II or III) for at least 3 months and who have a documented history of inadequate response to at least a 3-month course of at least 1 conventional systemic therapy (oral antibiotic or biologic drug) for HS (or demonstrated intolerance to, or have a contraindication to, a conventional systemic therapy for treatment of their HS), with HS lesions in at least 2 distinct anatomical areas and total abscess and inflammatory nodule count of at least 5. [redacted]

CCI

[redacted]

CCI [redacted]

2.3.2. Justification for Doses

CCI [redacted]

CCI [redacted]

2.3.3. Justification for Placebo

CCI [redacted]

2.4. Benefit/Risk Assessment

The potential risks to participants, based on the available nonclinical and clinical data, are summarized in CCI [redacted] Additionally, monitoring of AEs and applicable actions will be performed as indicated in Section 6.5.

Based on the expected mode of action of povorcitinib, participants randomly assigned to the active treatment group may experience clinically meaningful improvements in their HS lesions

during the PC period (12 weeks), and all participants will be offered povorcitinib during the EXT period (42 weeks). Participants will also contribute to the process of developing a novel anti-inflammatory agent for HS, a disease with high unmet need that is severely debilitating to patients' well-being and daily functioning.

Considering the cumulative safety information on povorcitinib, the potential risks identified are justifiable and appropriately balanced by the anticipated efficacy benefits expected to be afforded to participants. More detailed information about the known and expected benefits and risks and reasonably expected AEs of povorcitinib may be found in the IB.

CCI

[redacted]

CCI

[redacted]

3. OBJECTIVES AND ENDPOINTS

Table 8 presents the objectives and endpoints.

Table 8: Objectives and Endpoints

Objectives	Endpoints
**Primary**	
To evaluate the efficacy of povorcitinib.	• Proportion of participants who achieve Hidradenitis Suppurativa Clinical Response (HiSCR) at Week 12.<br>− HiSCR is defined as at least a 50% reduction from baseline in the total abscess and inflammatory nodule count, with no increase from baseline in abscess or draining tunnel count.
**Key Secondary**	
To further evaluate the efficacy of povorcitinib.	• Proportion of participants who achieve Hidradenitis Suppurativa Clinical Response 75 (HiSCR75) at Week 12.<br>− HiSCR75 is defined as at least a 75% reduction from baseline in the total abscess and inflammatory nodule count, with no increase from baseline in abscess or draining tunnel count.
To evaluate the effect of povorcitinib in reducing the incidence of flares.	• Proportion of participants with flare by Week 12.<br>− Participants who experience at least 1 flare over 12 weeks; flare is defined as at least a 25% increase in the total abscess and inflammatory nodule count with a minimum increase of 2 relative to baseline.
To evaluate the effect of povorcitinib in decreasing skin pain.	• Key secondary endpoint for regulatory purposes in US/FDA/aligned countries:<br>Proportion of participants with a ≥ 3-point decrease in Skin Pain Numeric Rating Scale (NRS) score at Week 12 among participants with baseline Skin Pain NRS score ≥ 3.<br>− Participants with a Skin Pain score of at least 3 at baseline and who experience at least a 3-point decrease in Skin Pain score at Week 12, relative to baseline. Skin Pain is an 11-point NRS, ranging from 0 (no skin pain) to 10 (worst skin pain).<br>• Key secondary endpoint for regulatory purposes in EU/EMA/aligned countries:<br>Proportion of participants who achieve Skin Pain NRS30 at Week 12 among participants with baseline Skin Pain NRS score ≥ 3.<br>− Participants with a Skin Pain score of at least 3 at baseline and who achieve at Week 12 Skin Pain NRS30, defined as at least a 30% reduction and at least 1-unit reduction from baseline in the Skin Pain NRS.

Table 8: Objectives and Endpoints (Continued)

Objectives	Endpoints
**Secondary**	
To further evaluate the effect of povorcitinib in decreasing skin pain.	• Secondary endpoint for regulatory purposes in US/FDA/aligned countries:<br>Proportion of participants who achieve Skin Pain NRS30 at Week 12 among participants with baseline Skin Pain NRS score ≥ 3.<br>− Participants with a Skin Pain score of at least 3 at baseline and who achieve at Week 12 Skin Pain NRS30, defined as at least a 30% reduction and at least 1-unit reduction from baseline in the Skin Pain NRS.<br>• Secondary endpoint for regulatory purposes in EU/EMA/aligned countries:<br>Proportion of participants with a ≥ 3-point decrease in Skin Pain NRS score at Week 12 among participants with baseline Skin Pain NRS score ≥ 3.<br>− Participants with a Skin Pain score of at least 3 at baseline and who experience at least a 3-point decrease in Skin Pain score at Week 12, relative to baseline. Skin Pain is an 11-point NRS, ranging from 0 (no skin pain) to 10 (worst skin pain).
To evaluate the effect of povorcitinib in improving fatigue.	• Proportion of participants with at least a 4-point increase from baseline in Functional Assessment of Chronic Illness Therapy – Fatigue (FACIT-F) score at Week 12.<br>− Participants with a baseline FACIT-F score ≤ 48 and who experience at least a 4-point increase in FACIT-F score at Week 12, relative to baseline. The FACIT-F scale is a 13-item questionnaire that assesses self-reported fatigue and its impact upon daily activities and function over the past 7 days, with scores ranging from 0 (worst fatigue) to 52 (no fatigue).
To evaluate the effect of povorcitinib on QoL during the PC and EXT periods.	• Mean change from baseline in Dermatology Life Quality Index (DLQI) score at each visit.
To further evaluate the efficacy of povorcitinib.	• Mean change from baseline in abscess count at each visit.<br>• Percentage change from baseline in abscess count at each visit.<br>• Mean change from baseline in inflammatory nodule count at each visit.<br>• Percentage change from baseline in inflammatory nodule count at each visit.<br>• Mean change from baseline in draining tunnel count at each visit.<br>• Percentage change from baseline in draining tunnel count at each visit.

Table 8: Objectives and Endpoints (Continued)

Objectives	Endpoints
**Secondary (continued)**	
To evaluate the efficacy of povorcitinib during the EXT period.	• Proportion of participants who achieve HiSCR at Week 24.<br>• Proportion of participants who achieve HiSCR75 at Week 24.<br>• Proportion of participants with flare by Week 24.<br>• Proportion of participants who achieved Skin Pain NRS30 at Week 24 among participants with baseline Skin Pain NRS score ≥ 3.<br>• Proportion of participants who achieve HiSCR at Week 54.<br>• Proportion of participants who achieve HiSCR75 at Week 54.<br>• Proportion of participants with flare by Week 54.<br>• Proportion of participants who achieved Skin Pain NRS30 at Week 54 among participants with baseline Skin Pain NRS score ≥ 3.
To evaluate the maintenance of response during the EXT period.	• Proportion of participants who achieve maintenance of HiSCR or greater response at each visit during the EXT period.<br>− Maintenance of response defined as participants who achieve HiSCR at Week 12 and maintain it or achieve greater response at each visit during the EXT period.<br>• Proportion of participants who achieve maintenance of HiSCR75 or greater response at each visit during the EXT period.<br>− Maintenance of response defined as participants who achieve HiSCR75 at Week 12 and maintain it or achieve greater response at each visit during the EXT period.
**Safety**	
To evaluate the safety and tolerability of povorcitinib.	• Frequency and severity of AEs, including changes in clinical laboratory results, vital signs, ECGs, and the results of physical examinations.

CCI
[redacted]

Table 8: Objectives and Endpoints (Continued)

Objectives	Endpoints
CCI [redacted]	[redacted]

Table 8: Objectives and Endpoints (Continued)

Objectives	Endpoints
CCI [redacted]	

Table 8: Objectives and Endpoints (Continued)

Objectives	Endpoints
CCI [redacted]	

4. STUDY DESIGN

4.1. Overall Design

This is a Phase 3, multicenter, randomized, parallel-group, PC, double-blind study in adult participants (age ≥ 18 years) with moderate to severe HS (Hurley Stage II or III), HS lesions in ≥ 2 distinct anatomical areas, and total abscess and inflammatory nodule count ≥ 5 at screening and baseline and who have a history of failure of, intolerance to, or contraindication to an adequate course of conventional systemic therapy for HS. Approximately 600 participants will be randomized 1:1:1 to povorcitinib (CCI [redacted] or CCI [redacted] mg QD) or placebo QD for 12 weeks; participants who complete the PC period may continue to a 42-week double-blind EXT period with povorcitinib CCI [redacted] mg or CCI [redacted] mg QD (see Figure 1). CCI [redacted]

Participants randomized to povorcitinib at baseline will remain at their respective dose level (CCI [redacted] or CCI [redacted] mg QD) throughout the PC and EXT periods, while participants randomized to placebo will receive povorcitinib CCI [redacted] or CCI [redacted] mg QD (CCI [redacted]) at the Week 12 visit onward, after completion of the PC period. Treatment assignment during the PC and EXT periods will be blinded to investigators and participants.

Participants will be screened for up to 28 days before the first dose of study drug; participants who are randomized in the study will receive the first dose of study drug on Day 1, and the last dose within the PC period will occur at the Week 12 visit. The first dose of study drug within the 42-week EXT period will occur on the next day after the Week 12 visit; the last dose within the EXT period will occur on the day of the Week 54 visit (or last study drug administration known to the investigator or designee as reported in the EDC for patients who prematurely discontinue the study or are lost to follow-up). Participants who complete the 54-week treatment in this study may be eligible to participate in a separate extension study to further evaluate the safety and efficacy of the investigational product. For participants who do not participate in the separate extension study, a safety follow-up visit (EOS) should be conducted approximately 30 (+ 7) days after EOT, or last administration of study drug (in case of premature discontinuation/early termination), for all participants.

Lesion count assessments will be performed to determine clinical efficacy endpoints; participants will answer daily diary QoL questionnaires on skin pain, [redacted] and complete the study drug daily diary during the PC and EXT periods. Additional PRO questionnaires will be answered by participants at the study visits. Participants will be assessed for safety and tolerability throughout the study by monitoring the type, frequency, and severity of AEs and measuring vital signs and clinical laboratory parameters. Details of the assessments are presented in Table 3.

4.2. Overall Study Duration

The study begins when the first participant signs the study ICF. The end of the study is defined as the date of the last visit of the last participant in the study globally. A participant is considered to have completed the study if they have completed all study treatment visits; for participants who do not participate in the separate extension study, study completion includes the safety follow-up visit (see Table 3).

It is estimated that an individual will participate for approximately 14 months, including up to 28 days for screening, continuous treatment for 54 weeks (including the PC and EXT periods, as long as participants are receiving benefit and have not met any criteria for study ET/withdrawal), and 30 (+ 7) days for follow-up after the last dose of study drug.

4.3. Study Termination

The investigator retains the right to terminate study participation at any time, according to the terms specified in the study contract. The investigator/head of the study site (Japan) is to notify the IRB/IEC in writing of the study's completion or early termination, send a copy of the notification to the sponsor or sponsor's designee, and retain 1 copy for the site study regulatory file.

The sponsor may terminate the study electively, if, for example, required by regulatory decision or upon advice of the DSMB (see Section 5.6). If the study is terminated prematurely, the sponsor will notify the investigators/head of the study site (Japan), the IRBs and IECs, and regulatory bodies of the decision and reason for termination of the study. For Japan, the decision from the sponsor will be via the head of the study site(s), who will notify the investigators and the IRBs of the decision and reason for termination of the study.

5. STUDY POPULATION

Deviations from eligibility criteria are not allowed because they can potentially jeopardize the scientific integrity of the study, regulatory acceptability, and/or participant safety. Therefore, adherence to the criteria as specified in this Protocol is essential. Prospective approval of Protocol deviations to recruitment and enrollment criteria, also known as Protocol waivers or exemptions, are not permitted.

5.1. Inclusion Criteria

Participants are eligible to be included in the study only if all of the following criteria apply:

1. Ability to comprehend and willingness to sign a written ICF for the study.
2. Age ≥ 18 years at the time of signing the ICF.
3. Diagnosis of moderate to severe HS for at least 3 months prior to the screening visit.
4. Total abscess and inflammatory nodule count of at least 5 at both the screening and baseline visits (see Section 8.2.1).
5. HS lesions in at least 2 distinct anatomical areas (examples include but are not limited to left and right axilla or left and right inguinocrural fold), 1 of which must be at least Hurley Stage II or Hurley Stage III, at both the screening and baseline visits (see Section 8.2.2).
6. Documented history of inadequate response to at least a 3-month course of at least 1 conventional systemic therapy (oral antibiotic or biologic drug) for HS (or demonstrated intolerance to, or have a contraindication to, a conventional systemic therapy for treatment of their HS; see Section 6.6.1.1).
7. CCI [redacted]
8. CCI [redacted]
9. Agreement to use contraception (see Appendix A), as follows:
   a. Female participants of childbearing potential (WOCBP) must agree to take appropriate precautions to avoid pregnancy with a highly effective method (ie, at least 99% certainty) from screening through 90 days after the last dose of study drug. Permitted methods that are at least 99% effective in preventing pregnancy should be communicated to the participants and their understanding confirmed. Note: This criterion does not apply to women of nonchildbearing potential (WONCBP), as defined in Appendix A.
   b. All women must agree to not donate eggs (ova, oocytes) for the purposes of assisted reproduction during the study and for at least 90 days after receiving the last dose of study drug.

c. A male participant who is sexually active with a woman of childbearing potential (WOCBP) must agree to use a highly effective barrier method of birth control (as described in Appendix A) during the study and for at least 90 days after receiving the last dose of study drug.

d. All men must agree to not donate sperm during the study and for at least 90 days after receiving the last dose of study drug.

10. Willing and able to comply with the study protocol and procedures.

5.2. Exclusion Criteria

Participants are excluded from the study if any of the following criteria apply:

1. Presence of > 20 draining tunnels (fistulas) at either the screening or baseline visit.

CCI [redacted]

[redacted]

4. A screening 12-lead ECG that demonstrates clinically significant abnormalities requiring treatment (eg, acute myocardial infarction, serious tachyarrhythmias or bradyarrhythmias) or that is indicative of serious underlying heart disease (eg, cardiomyopathy, major congenital heart disease, low voltage in all leads, Wolff-Parkinson-White syndrome) or criteria associated with Q wave interval (QT)/Fridericia-corrected Q wave interval (QTcF) abnormalities.
5. Have undergone significant trauma or major surgery (per investigator's assessment) within 30 days preceding the screening visit.
6. History of clinically significant (per investigator's judgment) drug or alcohol abuse within 6 months preceding the screening visit.

[redacted]

CCI

[redacted]

9. At the screening visit, any of the laboratory abnormalities defined in Table 9.

Table 9: Exclusionary Laboratory Values

Laboratory Parameter	Exclusion Criterion
CCI [redacted]	

10. Evidence of infection with *Mycobacterium tuberculosis* (ie, TB) as defined in Section 8.3.5.2.
11. Active HIV or acquired immunodeficiency syndrome. Active HIV is defined as confirmed positive anti-HIV antibody test (see Section 8.3.5.3).
12. Evidence of HBV or HCV infection or risk of reactivation (see Section 8.3.5.4).
13. Known hypersensitivity or severe reaction to povorcitinib or excipients of povorcitinib (refer to the IB) and/or other products in the same class.
14. Any condition, laboratory result, or result of screening assessments that would, in the investigator's and sponsor's (or designee's) judgment, interfere with full participation in the study, including administration of study drug and attending required study visits, pose a significant risk to the participant, or interfere with interpretation of study data.
15. The following participants are excluded in France: vulnerable populations according to article L.1121-6 of the French Public Health Code and adults under legal protection or who are unable to express their consent per article L.1121-8 of the French Public Health Code.

5.3. Lifestyle Considerations

5.3.1. Contraception

5.3.1.1. Female Participants

All female participants who are of childbearing potential must agree to use a highly effective method of contraception (ie, at least 99% certainty) consistently and correctly for the duration of the study and for at least 90 days after the last dose of investigational product.

The investigator or designee, in consultation with the participant, will confirm that the participant has selected an appropriate method of contraception for the individual participant from the permitted list of contraception methods (see Appendix A) and will confirm that the participant has been instructed in its consistent and correct use.

At timepoints indicated in the SoA (see Table 3), the investigator or designee will inform the participant of the need to use highly effective contraception consistently and correctly. Participants need to affirm their consistent and correct use of at least 1 of the selected methods of contraception (see Appendix A). In addition, the investigator or designee will instruct the participant to call immediately if the selected contraception method is discontinued or if pregnancy is known or suspected.

5.3.1.2. Male Participants

All male participants must be willing to take appropriate precautions to avoid fathering children (with at least 99% certainty) consistently and correctly (as outlined in Appendix A) for the duration of the study and for at least 90 days after the last dose of investigational product. If the male participant has a partner who is of childbearing potential, the partner should also use contraception as outlined in Appendix A.

5.3.2. Meals and Dietary Restrictions

- Study drug will be taken orally, preferably in the morning and at about the same time each day, with a full glass of water. The study drug can be taken with or without food. [redacted] [Table 3]). Further instructions on study drug handling are outlined in Appendix C.
- On any of the study visit days, participants should not smoke or ingest caffeine during the 30 minutes prior to blood pressure and heart rate measurements.
- On study visit days when a lipid panel blood sample will be collected (Baseline/Day 1; Weeks 6, 12, 18, 24, and 54; and EOS/Safety Follow-Up; see Table 3), participants should comply with the fasting requirement for at least 8 hours prior to the visit. Water and permitted nonstudy medications are allowed (see Section 6.6.6).
- Participants must abstain from consumption of grapefruit or grapefruit juice (pomelos, exotic citrus fruits, grapefruit hybrids) and of Seville oranges from 1 week before the start of study drug until after the final dose of study drug.

- Participants must abstain from consumption of dietary supplements containing St John's wort (*Hypericum perforatum*) from 1 week before the start of study drug until after the final dose of study drug.

5.3.3. Prohibited Therapies During the Placebo-Controlled Period

Some commonly used therapies may have an impact on HS and are specifically prohibited in this study as described in Section 6.6.

5.4. Screen Failures

Screen failures are defined as participants who consent to participate in the clinical study but are not subsequently randomly assigned to study drug.

Tests with results that fail eligibility requirements may be repeated once during screening if the investigator believes the result to be in error. Additionally, a participant who fails screening may repeat the screening process 1 time if the investigator believes that there has been a change in eligibility status. Participants who rescreen must reconsent and be assigned a new participant number.

All screening procedures with the possible exceptions noted below will be repeated during rescreening. The participant must meet all the eligibility criteria at the time of rescreening in order to qualify for the study. There is no minimum period of time a participant must wait to rescreen for the study. If the participant had a complete initial screening evaluation including the following assessments, these tests will not be required to be repeated for rescreening, provided the conditions noted in Section 5.1 and Section 5.2 are met, there are no changes in the participant's medical history that would warrant retesting, and no more than 90 days have passed:

- HBV, HCV, and HIV serology
- IGRA (QuantiFERON-TB Gold test [or IGRA equivalent such as T-SPOT® test] and/or local PPD skin test, if required)
- ECG

5.5. Replacement of Participants

No participants will be replaced at any time during this study. However, as noted in COVID-19–related guidance (see Appendix E), due to the evolving situation of the COVID-19 pandemic, the sponsor may decide to recruit additional participants in the study beyond the expected number (eg, if a substantial number of participants withdraw early from the study).

5.6. Data Safety Monitoring Board

This study will use an external DSMB to monitor safety. The voting members of the committee are external to the sponsor and will not be involved with the study in any other way (eg, they cannot be study investigators) and will have no competing interests that could affect their roles with respect to the study.

The DSMB will make recommendations to the sponsor regarding steps to ensure both participant safety and the continued ethical integrity of the study. Specific details regarding composition,

responsibilities, and governance, including the roles and responsibilities of the various members of the clinical study team, and requirements for the proper documentation of DSMB reports, minutes, and recommendations will be described in a separate DSMB charter, which will be reviewed and approved by all DSMB members.

5.7. Cardiovascular Adjudication Committee

An independent committee of physician experts in cardiovascular adjudication will be utilized to assess potential cardiovascular and thromboembolic AEs. Details will be described in a separate CAC charter.

6. STUDY TREATMENT

6.1. Study Treatment Administered

Information regarding study drug and administration is provided in Table 10. Participants will record study drug administration in a daily diary. Further information regarding study drug administration is provided in Appendix C.

The COVID-19 global pandemic may present challenges to the normal conduct of this study, requiring the outline of potential mitigation strategies described in Appendix E.

Table 10: Study Treatment Information

	**Study Treatment**	
**Study treatment name**	Povorcitinib	Matching placebo
**Mechanism of action**	JAK1 inhibitor	Not applicable
**Dose formulation**	Tablet, oral	Tablet, oral
**Unit dose strength(s) /dose level(s)**	[redacted] mg: 1 tablet<br>[redacted] mg: 1 tablet	Placebo: 1 tablet
**Administration instructions**	Povorcitinib or matching placebo will be taken orally QD, preferably in the morning and at about the same time each day, with a full glass of water, with or without food (see Appendix C for further information).<br>[redacted]	
**Packaging and labeling**	Povorcitinib will be provided as [redacted]-mg or [redacted]-mg tablets; both strengths match each other in smell, taste, and appearance. Placebo tablets will match the povorcitinib tablets in smell, taste, and appearance. Tablets will be packaged in bottles.<br>Each container will be labeled as required per country requirement.	
**Storage**	Ambient (15°C-30°C/59°F-86°F)	
**Status of treatment in participating countries**	Investigational	

6.2. Preparation, Handling, and Accountability

Note: For Japan, the term "investigator" below also represents the investigational drug storage manager.

The investigator or designee must confirm appropriate temperature conditions have been maintained during transit for all study treatments received and any discrepancies are reported and resolved before use of the study treatment.

Only participants enrolled in the study may receive study treatment, and only authorized site staff may supply or administer study treatment. All study treatment must be stored in a secure, environmentally controlled, and monitored (manual or automated) area in accordance with the labeled storage conditions with access limited to the investigator and authorized site staff.

The investigator or designee is responsible for study treatment accountability, reconciliation, and record maintenance (ie, receipt, reconciliation, and final disposition records). Inventory and accountability records must be maintained and readily available for inspection by the study monitor and are open to inspection at any time by any applicable regulatory authorities. The investigator or designee must maintain records that document the following:

- Delivery of study drug to the study site.
- Inventory of study drug at the site.
- Participant use of the study drug, including tablet counts from each supply dispensed.
- Return of study drug to the investigator or designee by participants.

The investigational product must be used only in accordance with the Protocol (see Appendix C). The investigator or designee will also maintain records adequately documenting that the participants were provided the specified study drug. These records should include dates, quantities, and any available batch or serial numbers or unique code numbers assigned to the investigational product and study participants.

Completed accountability records will be archived by the site. The investigator or designee will be expected to collect and retain all used, unused, and partially used containers of study drug until verified by the study monitor (unless otherwise agreed to by the sponsor). At the conclusion of the study, the investigator or designee will oversee the destruction of any remaining study drug according to institutional SOPs. If, however, local procedures do not allow on-site destruction, shipment of the study drug back to the sponsor is allowed. In this case, the site should (where local procedures allow) maintain the investigational supply until the study monitor inspects the accountability records in order to evaluate compliance and accuracy of accountability by the investigative site. At sites where the study drug is destroyed before monitor inspection, the monitors rely on documentation of destruction per the site SOP.

Further guidance and information for the final disposition of unused study treatments are provided in the study materials provided to sites.

6.3. Measures to Minimize Bias: Randomization and Blinding

All participants will be centrally assigned to study treatment using an IRT system (see Section 8.1.3). The system will assign in a 1:1:1 ratio (povorcitinib [redacted] mg QD, povorcitinib

[redacted] mg QD, placebo QD), [redacted] Before the study is initiated, the telephone number and call-in directions for the IRT and/or the log-in information and directions for the IRT will be provided to each site. Full details will be provided in the IRT Study Reference Manual.

[redacted]

Emergency unblinding will occur if an AE requires the investigator to be made aware of the participant's treatment assignment (see emergency unblinding procedures in Section 9.7 and refer to the IRT Study Reference Manual).

6.4. Study Treatment Compliance

Compliance with all study-related treatments should be emphasized to the participant by the site personnel, and appropriate steps should be taken to optimize compliance during the study. Compliance with study drug will be calculated by the sponsor based on the drug accountability documented by the site staff and monitored by the sponsor/designee. Participants will be instructed to bring all unused study drugs with them to the study visits in order for site personnel to conduct tablet counts to assess study drug accountability.

Participants' compliance must be within [redacted]% to [redacted]%, assessed at each study visit by the investigator (or designee). If outside of this range, it will be considered a protocol deviation. Participants consistently noncompliant with the study drug may be withdrawn from the study. The decision on withdrawal will be made by the investigator after consultation with the sponsor, and relevant correspondence will be archived in the site study file.

Participants who are randomized in the study will receive the first dose of study drug on Day 1. For the purposes of compliance calculation, the last dose of study drug within each compliance period will be taken on the day of the respective study visit. The last dose within the PC period will occur at the Week 12 visit. The first dose of the study drug within the 42-week EXT period will occur on the next day after the Week 12 visit; the last dose within the EXT period will occur on the day of the Week 54 visit or last study drug administration known to the investigator (or designee) as reported in the EDC (for participants who prematurely discontinue the study or are lost to follow-up).

[redacted]

CCI

[redacted]

CCI

[redacted]

CCI

[redacted]

CCI

[redacted]

[redacted]

6.5.2. Criteria for Permanent Discontinuation of Study Drug

The occurrence of unacceptable toxicity not caused by the underlying disease will require study drug to be permanently discontinued. Unacceptable toxicity is defined as follows:

- The occurrence of an AE that is related to study drug that, in the judgment of the investigator or the sponsor's medical monitor, compromises the participant's ability to continue study-specific procedures or is considered to not be in the participant's best interest.
- A persistent AE requiring an interruption of study drug for more than 28 days unless a greater delay has been approved by the sponsor (or designee).
- Recurrence of the initial AE(s) that previously led to drug interruption after restarting the study drug.
- Specific AEs requiring permanent discontinuation of study drug as described in Section 6.5.1.

See Section 7.1.2 for discontinuation procedures.

6.6. Concomitant Medications and Procedures

6.6.1. Prior HS Therapy

Any topical and systemic treatments for HS since initial diagnosis and any prescribed treatments for HS prior to study entry will be recorded in the eCRF. Information associated with response to prior HS therapy and reason for discontinuation will also be recorded in the eCRF. Examples of commonly used therapies for HS include, but are not limited to, the following:

- Topical antibiotics (eg, clindamycin)
- Other topical treatments (eg, azelaic acid, benzoyl peroxide, bleach baths/sodium hypochlorite, chlorhexidine, resorcinol, retinoids [isotretinoin, acitretin, alitretinoin], steroids, zinc pyrithione)
- Systemic antibiotics (eg, ciprofloxacin, clindamycin, dapsone, doxycycline, ertapenem, erythromycin, linezolid, metronidazole, minocycline, moxifloxacin, rifampin/rifampicin, tetracycline)
- Systemic hormonal therapies (eg, antiandrogen contraceptives [ethinyl estradiol, cyproterone acetate], finasteride, metformin, spironolactone)
- Systemic or topical JAK inhibitors (see examples in Section 6.6.3)

- Other systemic therapies (eg, apremilast, avacopan, azathioprine, colchicine, cyclosporine, methotrexate, retinoids [isotretinoin, acitretin, alitretinoin], steroids)
- Biologics, adalimumab (Humira®) or biosimilars
- Biologics, other anti–TNF-α (infliximab, etanercept)
- Biologics, anti-IL17A/anti-IL17A/F (secukinumab [Cosentyx®], bimekizumab [Bimzelx®], ixekizumab [Taltz®])
- Other biologics (see examples in Section 6.6.4)

CCI

[redacted]

CCI

[redacted]

CCI

[redacted]

CCI

[redacted]

6.6.6. Permitted Medications and Procedures

CCI

[redacted]

6.6.6.2. Wound Care Dressings

Concomitant use of wound care dressings for HS is allowed; however, options are limited to alginates, hydrocolloids, and hydrogels.

6.6.6.3. COVID-19 Vaccination

Participants who elect to receive non–live attenuated types of COVID-19 vaccines during the study may continue study drug administration.

CCI [redacted]

CCI

[redacted]

[redacted]

6.6.9. Lesion Rescue

In the event that a particularly painful HS lesion occurs that necessitates an acute intervention, the investigator (or designee) will have the option to perform Protocol-allowed interventions. Two types of interventions are allowed:

- [redacted]
  - [redacted]
  - [redacted]
- [redacted]
  - [redacted]
  - [redacted]

[redacted]

Each event of lesion rescue will also be captured as part of the HS lesion assessment and count (see Section 8.2.1).

The maximum number of rescue lesions per each period of the study is outlined below.

- PC period:

  [redacted]

- EXT period:

  [redacted]

[redacted]

CCI [redacted]

6.7. Treatment After the End of the Study

Participants who complete the 54-week treatment in this study may be eligible to participate in a separate extension study to further evaluate the safety and efficacy of the investigational product.

7. DISCONTINUATION OF STUDY TREATMENT AND PARTICIPANT WITHDRAWAL

7.1. Discontinuation of Study Treatment

7.1.1. Reasons for Discontinuation

Participants **must** be discontinued from study treatment for the following reasons:

- The participant becomes pregnant.
- Consent is withdrawn.

  Note: Consent withdrawn means that the participant has explicitly indicated that they do not want to be followed any longer; in this case no further data, except data in the public domain, may be solicited from or collected on the participant. Consent may also be partial. Participants may choose to discontinue study drug and remain in the study to complete the safety follow-up visit.
- Further participation would be injurious to the participant's health or well-being, in the investigator's medical judgment.
- Any AE of unacceptable severity as noted in Section 6.5.
- Participant meets discontinuation criteria as noted in Section 6.5.
- Participant has received a prohibited medication or procedure as described in Section 6.6.8.
- Participant has received a prohibited systemic rescue therapy/regimen other than those described in Section 6.6.10.
- The study is terminated by the sponsor.
- The study is terminated by the local health authority, IRB, or IEC.

A participant **may** be discontinued from study treatment as follows:

- If, during the course of the study, a participant is found not to have met eligibility criteria, the medical monitor, in collaboration with the investigator, will determine whether the participant should be withdrawn from study treatment.
- If a participant is noncompliant with study procedures or study drug administration in the investigator's opinion, the sponsor should be consulted for instruction on handling the participant.

7.1.2. Discontinuation Procedures

In the event that the decision is made to permanently discontinue the study treatment, the EOT/ET visit should be conducted (see Section 8.7). Reasonable efforts should be made to have the participant return for a safety follow-up visit. These visits are described in Table 3. The last date of the last dose of study drug and the reason for discontinuation of study drug will be recorded in the eCRF.

If a participant is discontinued from study treatment:

- The reason(s) for discontinuation must be documented in the participant's medical record and the primary reason for discontinuation must be included in the eCRF.
- The EOT/ET visit should be performed and date recorded.
- The status of the participant should be updated to EOT in the IRT.
- Participants must be followed for safety until the time of the follow-up visit or until study drug–related toxicities resolve, return to baseline, or are deemed irreversible, whichever is longest.

If the participant discontinues study treatment and actively withdraws consent for collection of follow-up data (safety follow-up or disease assessment), then no additional data collection should occur; however, participants will have the option of withdrawing consent for study treatment but continuing in the safety follow-up period of the study for safety/efficacy assessments.

7.2. Participant Withdrawal From the Study

A participant may withdraw from the study at any time at their own request or may be withdrawn at any time at the discretion of the investigator for safety, behavioral, compliance, or administrative reasons.

If a participant withdraws from the study, they may request destruction of any samples taken and not tested, and the investigator must document this in the site study records. If the participant withdraws consent for disclosure of future information, the sponsor may retain and continue to use any data collected before such a withdrawal of consent. See Table 3 for data to be collected at the time of study withdrawal and follow-up and for any further evaluations that need to be completed.

7.3. Lost to Follow-Up

A participant will be considered lost to follow-up if they repeatedly fail to return for scheduled visits and are unable to be contacted by the study site. The following actions must be taken if a participant fails to return to the clinic for a required study visit:

- The site must attempt to contact the participant and reschedule the missed visit as soon as possible and counsel the participant on the importance of maintaining the assigned visit schedule and ascertain whether or not the participant wishes to and/or should continue in the study.
- Before a participant is deemed lost to follow-up, the investigator or designee must make every effort to regain contact with the participant (where possible, 3 telephone calls and, if necessary, a certified letter to the participant's last known mailing address or local equivalent methods). These contact attempts should be documented in the participant's medical record.
- Should the participant continue to be unreachable, they will be considered to have withdrawn from the study.

8. STUDY ASSESSMENTS AND PROCEDURES

See Appendix E for COVID-19–related guidance.

8.1. Administrative and General Procedures

8.1.1. Informed Consent Process

- The investigator or their representative will explain the nature of the study to the participant or their legally authorized representative and answer all questions regarding the study.
  - Informed consent must be obtained before any study-related procedures are conducted, unless otherwise specified by the Protocol.
  - Informed consent must be obtained using the IRB/IEC-approved version in a language that is native and understandable to the participant. An ICF template will be provided by the sponsor or its designee. The sponsor or its designee must review and acknowledge the site-specific changes to the ICF template. The ICF must include a statement that the sponsor or its designee and regulatory authorities have direct access to participant records.
  - The ICF must contain all required elements including optional samples/procedures (eg, optional biopsy) and describe the nature, scope, and possible consequences of the study in a form understandable to the study participant.
- Participants must be informed that their participation is voluntary. Participants or their legally authorized representative will be required to sign a statement of informed consent that meets the applicable requirements and regulations for the countries in which the study is being conducted as well as the IRB/IEC or study center.

- The participant must be informed that their personal data collected for the study will be used by the sponsor and/or their designee(s) in accordance with local data protection laws. The level of disclosure must also be explained to the participant.
- The participant must be informed that their medical records may be examined by Clinical Quality Assurance auditors or other authorized personnel appointed by the sponsor, by appropriate IRB/IEC members, and by inspectors from regulatory authorities.
- The medical record must include a statement that written informed consent was obtained before the participant was enrolled in the study and the date the written consent was obtained. The authorized person obtaining the informed consent must also sign the ICF.
- Participants who are current or past long-time smokers must be informed of the potential risks associated with participation in this study and treatment with the investigational product.
- Participants must provide consent to the most current version of the ICF during their participation in the study.
- A copy of the ICF(s) must be provided to the participant.
- Participants who are rescreened are required to sign a new ICF. A new participant number will be assigned.

8.1.2. Screening Procedures

Screening is the interval between signing the ICF and the day the participant is randomized in the study (Day 1/Baseline). Screening may not exceed 28 days. Assessments that are required to demonstrate eligibility may be performed over the course of 1 or more days during the screening process. A signed ICF must be obtained as the first step and before IRT screening registration or any other study-specific assessments. The screening of a participant should be registered in the IRT, which will assign the study-specific ID. Each participant who has signed the ICF should be registered in the IRT contemporaneously and without undue delays.

Central laboratory results will be used to determine eligibility unless otherwise stated (see Sections 5.1, 5.2, 8.3.4, and 8.3.5).

Results from the screening visit evaluations will be reviewed to confirm eligibility before the randomization transaction is registered in the IRT and before the administration of study drug. Tests with results that fail eligibility requirements may be repeated once during screening if the investigator believes the results to be in error. For screening assessments that are repeated, the most recent available result before randomization will be used to determine eligibility.

See Sections 5.4 and 5.5 for information regarding screen failures and replacement of participants, respectively.

8.1.3. Interactive Response Technology Procedure

Each participant will be identified in the study by a participant ID number, which is a combination of the 2-letter alpha country International Standards Organization (ISO) code and

then a 3-digit site number, followed by a 3 digit participant number. Site staff should contact the IRT to obtain the participant ID number during screening.

Upon determining that the participant is eligible for randomization, the IRT will be contacted to obtain the treatment assignment. Additionally, the IRT will be contacted at the study visits indicated in Table 3 to update the study drug supply. Additional details will be provided in the IRT Manual.

8.1.4. Distribution of Participant Card

Participants will be provided with a trial participant card at screening. The trial participant card will provide general information on study drug administration, including on study visits when they should not self-administer study drug; remind the participant regarding fasting procedures for predetermined visits (see Section 5.3.2); and contain a telephone number to contact in case of emergency.

8.1.5. Demography and Medical History

8.1.5.1. Demographics and Medical History

Demographic data and general medical history will be collected at screening by the investigator or qualified designee and will include year of birth/age; race; ethnicity; medical and surgical history; family history of cardiovascular disease; family history of clotting disorders; risk factors associated with cardiovascular disease; history of nicotine, alcohol, and medical cannabis (marijuana) use; and current illnesses. Medical history will include relevant medical and surgical treatments that are considered to be clinically significant by the investigator.

History of clinical herpes zoster, herpes zoster vaccination, chickenpox vaccination, and hepatitis B vaccination status will be recorded as part of the medical history.

History of COVID-19 vaccination and COVID-19 infection (and/or hospitalization) will be recorded as part of the medical history.

A detailed medical history with respect to TB risk factors will be documented in the study source documentation. This information will include BCG vaccination, cohabitation with individuals who have had TB, and travel to, residence in, or work in TB endemic locations.

8.1.5.1.1. Justification for Collection of Race and Ethnicity

Recent studies have reported that patients of African or Hispanic descent experience more severe (ie, higher Hurley stage) HS, are more likely to have been previously treated with more effective treatments, have an average higher number of comorbid conditions, and are more likely to have recalcitrant HS (Lee et al 2017, Shao et al 2022). Although the reasons behind these discrepancies are unclear, further studies are required to better understand the link between skin of color and HS severity (Kilgour et al 2021).

With the above considerations in perspective, demographic data, including race and ethnicity, will be collected during the screening period for this study and considered as part of assessments for treatment response heterogeneity.

8.1.5.2. Disease Characteristics and Treatment History

Complete HS medical and treatment history, including date of diagnosis, relevant disease characteristics, and prior treatments, including systemic treatments and surgical procedures, will be collected at screening. A medical or surgical history of other conditions related to HS or relevant to the conduct of this clinical trial will also be collected at screening

Additional information on the procedure for collection of prior HS therapy and surgery information is presented in Section 6.6.1 and Section 6.6.2. Prior use of JAK inhibitor(s) and prior use of immunomodulatory biologic drug(s) for any indication will also be collected, as respectively outlined in Section 6.6.3 and Section 6.6.4.

CCI [redacted]

CCI [redacted]

8.2. Efficacy Assessments

Clinician-reported outcomes and PROs are described in this section.

Clinical evaluations of HS (lesion assessment/count and Hurley staging) will be performed by an experienced and qualified evaluator. The evaluator must receive and document Protocol-specific HS lesion assessment/count and Hurley staging training prior to performing any evaluation in any participant in this study.

To assure consistency and reduce variability, the same evaluator must perform all HS clinical evaluations for any individual participant throughout the study. A backup experienced and qualified, Protocol-trained evaluator will only be allowed and documented in case of emergency or special situations when the designated evaluator is unable to perform the HS clinical evaluations.

The HS lesion assessment/count, Hurley staging, information on lesion rescue, and any other information related to efficacy assessments will be captured in a paper-based study-specific form and entered in the corresponding eCRF. The calculations used for the endpoints described in Section 8.2.1.1 through Section 8.2.1.5 will be performed by the sponsor (or designee).

8.2.1. HS Lesion Assessment and Count

The lesion count is defined as a broad assessment of all the various skin "appearances" that are termed "lesions" in participants with HS. The lesion count must address all relevant anatomical regions in each participant and will be captured at all visits in a paper-based study-specific form and entered in the corresponding eCRF (see Table 3). The lesion count will be used for calculation (by the sponsor or designee) of various efficacy parameters, such as HiSCR, IHS4, and flare incidence.

The nomenclature for HS lesions adapts the glossary proposed by Frew et al (2021) and is outlined in Table 13.

Table 13: HS Lesion Definition

HS Lesion	Definition	Schematic
Abscess	A tender, fluctuant (compressible), palpable lesion, with erythema	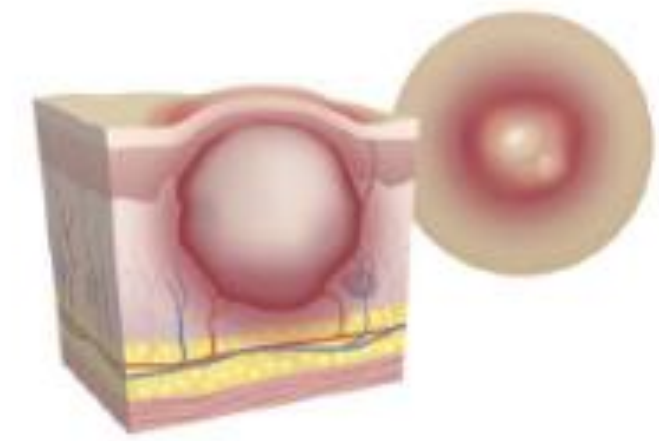
Inflammatory nodule	A solid, spherical, palpable lesion >1 cm; tender and/or erythematous	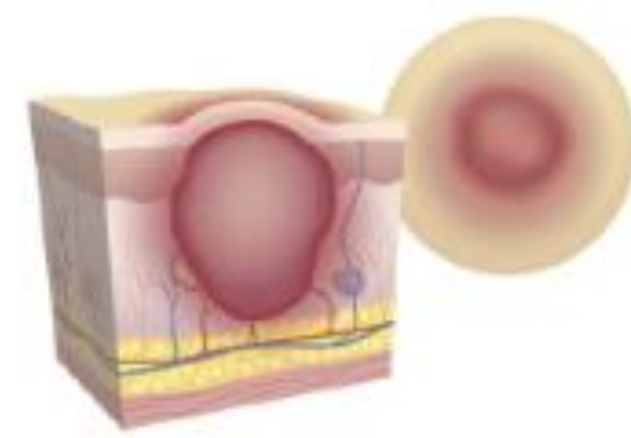
Noninflammatory nodule	Nontender, nonerythematous nodule	
Eroded nodule	A friable nonepithelialized nodule	
Draining tunnel (fistula)	A linear tract that may open onto the skin surface, with drainage expressed at rest or with compression of surrounding structures	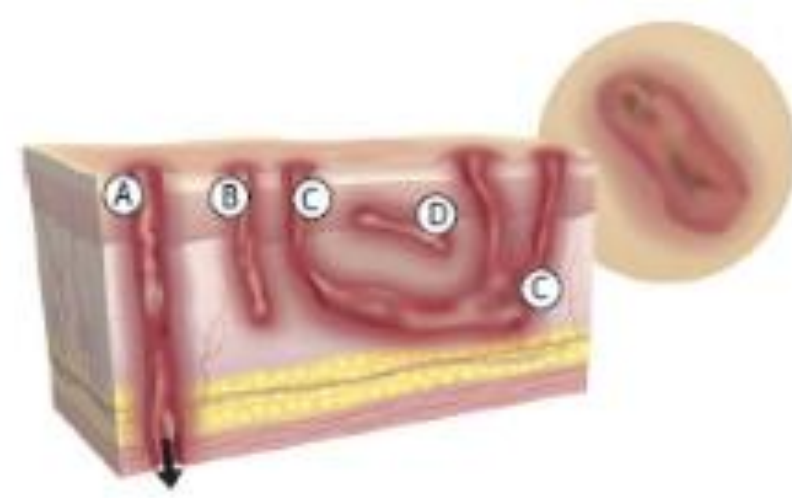
Nondraining tunnel (fistula)	A linear tract that may open onto the skin surface; compression of surrounding structures does not elicit drainage of contents	(A) connection to a hollow organ (eg, bladder, intestine); (B) blind ending; (C) 2 skin-to-skin openings; (D) no opening to the surface
Papule	A solid, palpable lesion ≤1 cm<br>Inflammatory papule: tender and/or erythematous<br>Noninflammatory papule: nontender, nonerythematous	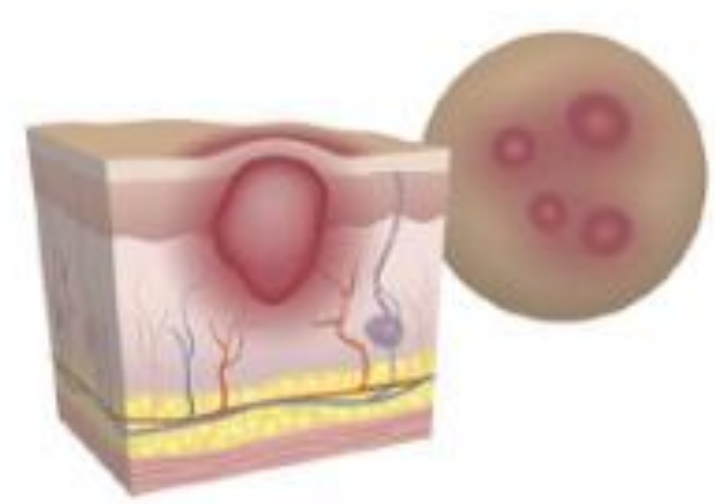
Pustule	A superficial circumscribed lesion that contains opaque fluid (pus) ≤ 1 cm	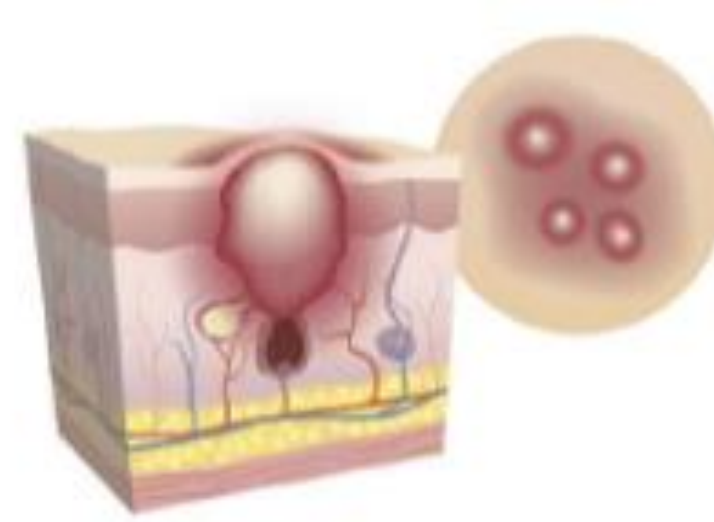

Table 13: HS Lesion Definition (Continued)

HS Lesion	Definition	Schematic
Plaque	A variably shaped, nonspherical, palpable lesion > 1 cm<br>Inflammatory plaque: tender and/or erythematous<br>Noninflammatory plaque: nontender, nonerythematous, may be a type of scar	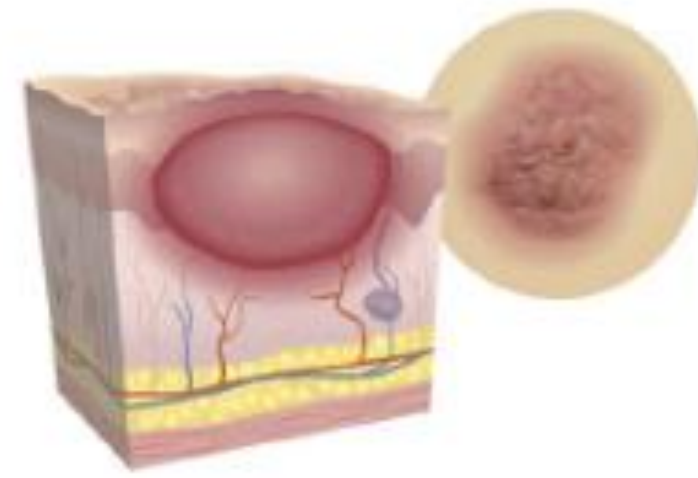
Ulcer	Full-thickness loss of epidermis and at least a portion of the dermis	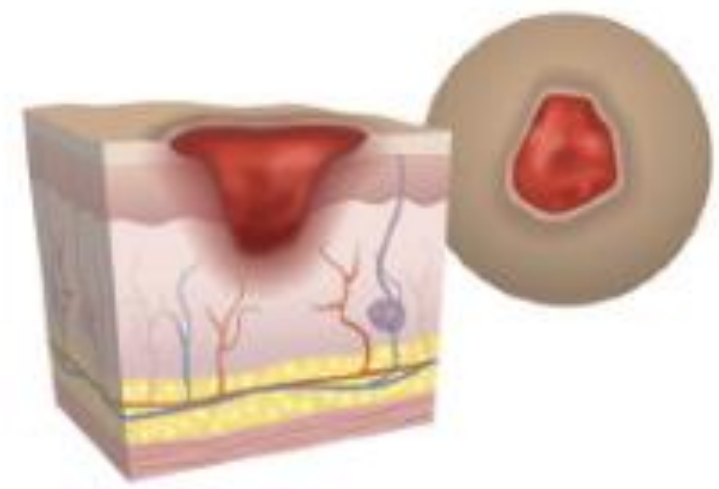
Comedo	Dilated skin opening with keratinous debris<br>Double comedo: interconnected comedones	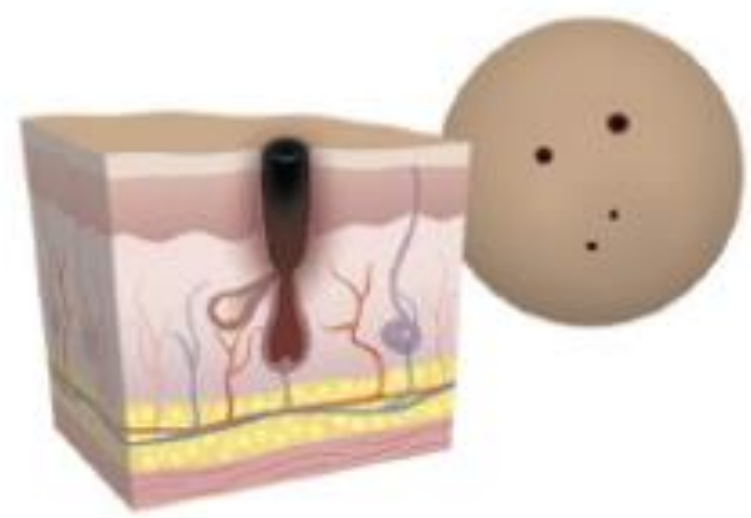

The HS lesions that will be counted in this study are outlined below:

CCI [redacted]

The distinct anatomical areas (with associated alternate nomenclature, if applicable) that will be considered for lesion assessment and count are outlined below. If lesions are present in an anatomical area(s) not outlined below, they will be recorded under "Other area(s)."

CCI [redacted]

CCI

[redacted]

8.2.1.1. Hidradenitis Suppurativa Clinical Response

The HiSCR is a validated endpoint that shows responsiveness to improvement in disease activity, simplifies the scoring process, and increases the sensitivity to detect certain HS-specific lesions. HiSCR calculations will be performed by the sponsor (or designee). The HiSCR is defined by the status of 3 types of lesions (Kimball et al 2016c): abscess, inflammatory nodules, and draining tunnels.

The definition of response requires the 3 criteria below to be met, relative to baseline:

- At least 50% decrease in abscess plus inflammatory nodule count; and
- No increase in abscess count; and
- No increase in draining tunnel count

HiSCR75 and HiSCR90 will also be calculated by the sponsor (or designee) as endpoints in this study (see Table 8); their only difference compared to HiSCR is the minimum percentage decrease in total abscess and inflammatory nodule count required to achieve response of 75% and 90%, respectively.

8.2.1.2. Change in HS Lesion Counts

Absolute and relative changes in the counts of the different types of HS lesions will be calculated by the sponsor (or designee) as endpoints in this study (see Table 8).

8.2.1.3. International Hidradenitis Suppurativa Severity Score System

The IHS4 is a composite, dynamic score and validated tool used to determine HS severity. It employs a weighted scale that sums the number of inflammatory nodules (multiplied by 1), the number of abscesses (multiplied by 2), and the number of draining tunnels (multiplied by 4; Zouboulis et al 2017).

Although not used as an inclusion criteria for HS severity in this study (see Hurley stage in Section 8.2.2), the total IHS4 score outlines the following disease categories:

- Mild disease: ≤ 3 points
- Moderate disease: 4 to 10 points
- Severe disease: ≥ 11 points

The use of the IHS4 as a dichotomous endpoint has been proposed recently (IHS4-55), suggesting a performance similar to HiSCR. The definition of response requires at least a 55% decrease from baseline in the total IHS4 score (van Straalen et al 2022a).

IHS4 and related measurements will be calculated by the sponsor (or designee) as endpoints in this study (see Table 8).

8.2.1.4. Flare Incidence

In this study, the definition of flare is outlined below, as proposed by Zouboulis et al (2019):

- At least 25% increase in the total abscess and inflammatory nodule count, relative to baseline; and
- An increase of at least 2 in the total abscess and inflammatory nodule count, relative to baseline

Flare and related flare patterns will be calculated by the sponsor (or designee) as endpoints in this study (see Table 8).

8.2.1.5. Lesion Rescue

In the event of acutely painful lesion(s) that may require intervention, the investigator or designee may perform Protocol-allowed rescue. The frequency and number of lesion rescue treatments will be assessed as endpoints (see Table 8). Rescued lesions will be counted as permanently present (ie, nonresponder lesions), even if they subside over the course of the study.

Specific information on the type and frequency of Protocol-allowed lesion rescue intervention is described in Section 6.6.9.

8.2.2. Hurley Stage

The presence of draining or nondraining tunnels (also known as fistulas/tracts) is the key aspect that sets apart Hurley Stage I from Hurley Stage II/III; tunnels are associated with irreversible tissue damage, which can only be treated with surgery. Therefore, once an anatomical region is identified as Stage II or III, it can never fall below Stage II (Zouboulis et al 2021).

Each anatomical area (as outlined in forms specifically designed for this study) will have their specific Hurley stage captured at visits determined in the SoA (see Table 3). If more than 1 stage is present in the same region, the worst stage in that region should be documented. The participant will be assigned an overall Hurley stage classification corresponding to the stage of the worst involved anatomical region.

The definition of each Hurley stage is outlined in Table 14.

Table 14: Hurley Stages of Hidradenitis Suppurativa

Stage I	Stage II	Stage III
Abscess formation, single or multiple, without tunnels and cicatrization (scarring)	One or more widely separated recurrent abscesses with tunnel (draining or nondraining) formation and cicatrization (scarring)	Multiple interconnected tunnels (draining or nondraining) and abscesses across the entire area, with diffuse or near-diffuse involvement

Source: Jemec 2012.

CCI [redacted]

[redacted]

[redacted]

8.2.4. Patient-Reported Outcomes

All PROs will be collected via eCOA. Sites will be provided with training materials and instructions on the use of eCOA and will be responsible to train participants on eDiary (handheld device) and eCOA (tablet device) use. The participant handheld device will be dispensed at the screening visit. Upon completion of participation in the study, the participant should return the device to the site; the device must be checked for any pending data transmissions and deactivated. Further details on use of the devices will be provided in respective eCOA study documentation.

For PROs scheduled to be completed at study visits (see Table 3), questionnaires will be completed by the participant before site personnel perform any assessments to avoid biasing the participant's responses.

8.2.4.1. Daily Diary Patient-Reported Outcomes

8.2.4.1.1. Administrative Procedures

8.2.4.1.1.1. Follow-Up Phone Call

Study sites should contact participants 1 week prior to the baseline visit and 1 week prior to the Week 12 visit to confirm compliance with the daily diary assessments.

8.2.4.1.1.2. Compliance With Daily Diary Patient-Reported Outcomes

Participants' compliance with daily diary PRO assessments (Skin Pain NRS, Analgesic Use, and Itch NRS) should be monitored by the investigator (or designee) throughout study conduct and required follow-up performed with the participant in case of noted noncompliance. Compliance will be evaluated as outlined below:

- Screening period:
  - CCI [redacted]
- PC and EXT periods:
  - Participants' compliance with the daily diary assessments should be assessed at each study visit during the PC and EXT periods.
  - Participants who are consistently noncompliant with the daily diary assessments may be withdrawn from the study.

8.2.4.1.2. Skin Pain NRS

Participants will answer the Skin Pain NRS question daily via eDiary from screening through Week 54 based on a 24-hour recall period (see Table 3).

The Skin Pain NRS ranges from 0 (no skin pain) to 10 (skin pain as bad as you can imagine) and evaluates the worst level of skin pain due to the participant's HS.

CCI [redacted]

8.2.4.2. Patient-Reported Outcomes Completed at Study Visits

For PROs scheduled to be completed at study visits per the SoA (see Table 3), questionnaires will be completed by the participant before site personnel perform any clinic assessments to avoid biasing the participant's response.

CCI [redacted]

8.2.4.2.2. Dermatology Life Quality Index

The DLQI is a simple, 10-question validated questionnaire to measure how much the skin problem has affected the participant over the previous 7 days, across symptoms and feelings, daily activities, leisure, work and school, personal relations, and treatment (Finlay and Khan 1994). Participants will complete the DLQI at the study visits designated in Table 3.

8.2.4.2.3. Functional Assessment of Chronic Illness Therapy – Fatigue Scale

Participants will complete the FACIT-F scale at the study visits designated in Table 3. The FACIT-F scale is a 13-item questionnaire originally designed to assess fatigue, over a 7-day recall, in patients with chronic disease. The total FACIT-F score ranges from 0 (maximum fatigue) to 52 (no fatigue). Lower scores are indicative of a greater impact on the participants' QoL (Cella et al 2019).

CCI [redacted]

CCI [redacted]

CCI [redacted]

8.3. Safety Assessments

Planned timepoints for all safety assessments are provided in the SoA (see Table 3).

See Section 6.5 for guidelines regarding the management of relevant laboratory or other safety assessment abnormalities.

The COVID-19 global pandemic may present challenges to the normal conduct of this study, requiring the outline of potential mitigation strategies described in Appendix E.

8.3.1. Adverse Events

Adverse events will be monitored from the time the participant signs the ICF until at least 30 days after the last dose of study drug. Adverse events for enrolled participants that begin or worsen after informed consent should be recorded on the Adverse Events Form in the eCRF regardless of the assumption of a causal relationship with the study drug. Conditions that were already present at the time of informed consent should be recorded on the Medical History Form in the eCRF. Adverse events (including laboratory abnormalities that constitute AEs) should be described using a diagnosis whenever possible rather than by individual underlying signs and symptoms.

Adverse events will be reported by the participant (or, when appropriate, by a caregiver, surrogate, or the participant's legally authorized representative). The investigator and any qualified designees are responsible for detecting, documenting, and recording events that meet the definition of an AE or SAE and remain responsible for following up on AEs that are serious, that are considered related to the study drug/procedures, or that caused the participant to discontinue the study drug. Care will be taken not to introduce bias when detecting AEs and/or SAEs. Open-ended and nonleading verbal questioning of the participant, such as "How are you feeling?", is the preferred method to inquire about AE occurrences. Adverse events may also be

detected when they are volunteered by the participant during the screening process or between visits or through physical examinations, laboratory tests, or other assessments. The definition, reporting, and recording requirements for AEs are described in Section 9.

If an AE is noted, a targeted physical examination should be conducted as indicated by the symptoms reported by the participant or other findings (eg, laboratory abnormalities). Abnormalities that are considered clinically significant in the judgment of the investigator (or designee) are to be reported as AEs.

All SAEs will be reported to the sponsor or designee within 24 hours, as outlined in Section 9.4. The investigator will submit any updated SAE data to the sponsor within 24 hours of it being available.

After the initial AE/SAE report, the investigator is required to proactively follow each participant at subsequent visits/contacts. All SAEs, and nonserious AEs of special interest (as defined in Section 9.6.1), will be followed until resolution, stabilization, the event is otherwise explained, or the participant is lost to follow-up (as defined in Section 7.3).

8.3.2. Physical Examinations

Physical examinations will be performed as indicated in the SoA (see Table 3).

Height and body weight will be assessed as determined in the SoA (see Table 3).

A comprehensive physical examination, at visits specified in Table 3, will assess the following organ or body systems: skin; head, eyes, ears, nose, and throat; thyroid; lungs; cardiovascular system; abdomen (liver, spleen); extremities; and lymph nodes. A brief neurologic examination will also be performed.

As noted in Section 8.3.1, a targeted physical examination should be conducted as indicated by symptoms reported by the participant, AEs, or other findings. Abnormalities that are considered clinically significant in the judgment of the investigator are to be reported as AEs.

Physical examinations must be performed by a medically qualified individual such as a licensed physician, a physician assistant, or an advanced registered nurse practitioner, as local law permits.

8.3.3. Vital Signs

Vital signs measurements include blood pressure, pulse, respiratory rate, and body temperature and will be obtained as outlined in the SoA (see Table 3). Participant should not smoke or ingest caffeine during the 30 minutes prior to blood pressure and heart rate measurements (see Section 5.3.2 for details).

Vital signs should be collected before blood collection procedures. Alternatively, a minimum interval of 30 minutes between the completion of the blood collection procedures and the beginning of the vital signs collection is recommended. Blood pressure and pulse will be taken with the participant in the recumbent, semirecumbent, or sitting position after 5 minutes of rest.

Abnormal vital sign results identified after the first dose of study drug/treatment constitute an AE if they are considered clinically meaningful, induce clinical signs or symptoms, require concomitant therapy, or require changes in study drug/treatment.

8.3.4. Electrocardiograms

Single 12-lead ECGs will be obtained as outlined in the SoA (see Table 3) using an ECG machine and the following parameters will be recorded in the eCRF: PR, QRS, QT, and QTc intervals. All 12-lead ECGs will be performed with the participant in a recumbent or semirecumbent position after 5 minutes of rest.

The 12-lead ECGs will be interpreted by the investigator or qualified designee for adequate participant management. Additional 12-lead ECGs may be performed as clinically indicated to manage participant safety. Clinically notable abnormalities that are considered clinically significant in the judgment of the investigator are to be reported as AEs.

8.3.5. Laboratory Assessments

See Table 15 for the list of clinical laboratory tests to be performed and Table 3 for the laboratory assessment visit schedule. A central laboratory will perform all clinical laboratory assessments for safety (ie, blood chemistries, hematology assessments, coagulation tests, endocrine function, and urinalysis). The sponsor or designee will perform all PK and translational sample analyses. Additional testing may be required by the investigator or sponsor based on emerging safety data. All Protocol-required laboratory assessments must be conducted in accordance with the Laboratory Manual and Table 3.

Information regarding collection, processing, and shipping of samples for laboratory assessment is provided in the Laboratory Manual.

Unless specified otherwise for selected tests and under the conditions outlined in Section 5.4, if screening laboratory assessments are performed more than 28 days before Day 1, then the tests must be repeated and eligibility confirmed before study drug administration on Day 1.

Clinically significant abnormal laboratory findings are those that are not associated with the underlying disease, unless judged by the investigator to be more severe than expected for the participant's condition. All laboratory tests with values considered clinically significantly abnormal during participation in the study or within 30 (+ 7) days after the last dose of study drug should be repeated until the values return to normal or baseline or are no longer considered clinically significant by the investigator or medical monitor.

Laboratory sample collection on Day 1 must be performed before study drug administration.

See Section 9.1 for information regarding laboratory abnormalities that should be recorded as an AE in the eCRF.

CCI

[redacted]

8.3.5.1. Pregnancy Testing

Based on assessment at the screening visit (see guidance in Appendix A), female participants will be determined as WONCBP or WOCBP, and specific testing will be conducted as outlined respectively in Sections 8.3.5.1.1 and 8.3.5.1.2.

8.3.5.1.1. Women of Nonchildbearing Potential

A female who is permanently surgically sterile or postmenopausal (no menses for 12 or more months without an alternative medical cause) is considered to be a WONCBP and is not required to follow contraception recommendations. Further information on the determination of WONCBP status is presented in Appendix A. Confirmation of WONCBP status by serum FSH testing will be conducted at screening for all female participants considered to be permanently surgically sterile or postmenopausal.

- If FSH is not tested, it is assumed that the participant is of childbearing potential and Protocol-specified contraception requirements must be followed.
- If the FSH is tested and the result is consistent with postmenopausal status, contraception requirements are not necessary.
- If the FSH is tested and the result is consistent with premenopausal status, contraception requirements and pregnancy testing requirements for WOCBP must be followed (see Section 8.3.5.1.2).

8.3.5.1.2. Women of Childbearing Potential

A female who does not meet the definition of WONCBP is considered of childbearing potential and is required to practice at least 1 highly effective method of birth control (see Appendix A) from screening through at least 90 days after the last dose of study drug.

If during the course of the study a female participant becomes surgically sterile or postmenopausal, and complete documentation is available, contraceptive measures are no longer required.

A serum pregnancy test will be required for all WOCBP during screening (before the first dose of study drug) and at the safety follow-up visit as indicated in Table 3. Urine pregnancy tests will be performed locally as outlined in Table 3, as medically indicated (eg, in case of loss of menstrual cycle, when pregnancy is suspected), or per country-specific requirement (note that country-required urine pregnancy testing will be outlined and communicated to investigational sites under separate cover). If a urine pregnancy test is positive, the results should be confirmed with a serum pregnancy test.

If the serum pregnancy test is negative after a urine test was positive, the investigator will assess the potential benefit/risk to the participant and determine whether it is in the participant's best interest to resume study drug and continue participation in the study.

If a pregnancy is confirmed by a serum pregnancy test, the participant should be immediately discontinued (see Section 7.1). Additionally, Section 9.8 should be followed for reporting requirements.

8.3.5.2. Tuberculosis Assessment

During the screening period, all participants will be screened for TB infection via central laboratory assessment (see Section 8.3.5.2.1). Confirmed negative status must be obtained before the participant is randomized in the study and receives study drug. There are different outcomes that may be considered:

- If QuantiFERON Gold (or IGRA equivalent) test (and PPD skin test, if applicable) is negative, the participant may be randomized.
- If QuantiFERON Gold (or IGRA equivalent) test (or PPD skin test, if applicable) is positive, the participant must be further evaluated via CXR (or alternative imaging method, such as MRI or CT) for confirmation of TB status (active or latent).
  - Participants with evidence of active TB must not be randomized.
  - Participants with evidence of latent TB must not be randomized, unless a course of at least 2 months of appropriate therapy has been completed prior to randomization.

During the clinical trial, if a participant is experiencing signs or symptoms suspicious for TB, an ad hoc TB test (including CXR or alternative imaging method, such as MRI or CT) may be performed ahead of the next scheduled assessment. The sponsor's medical monitor should be informed of such cases. Confirmed TB infection, after the participant has started the study, should be reported as an AE.

8.3.5.2.1. Tuberculosis Test

- QuantiFERON-TB Gold test (or IGRA equivalent, such as T-SPOT TB test) is a default central laboratory assessment for TB. When required as per local standards, a PPD skin test (also known as tuberculin skin test or Mantoux) can be performed. The same TB testing method should be used throughout the study.

  Note: The PPD skin test should be read by a licensed health care professional between 48 and 72 hours after administration. A participant who does not return within 72 hours will need to be rescheduled for another skin test. The reaction will be measured in millimeters of induration, and induration ≥ 5 mm is considered a positive reaction. The absence of induration will be recorded as "0 mm," not "negative." Participants who have had an ulcerating reaction to the PPD in the past should not be re-exposed and the PPD should be considered positive.
- For regions that require both PPD and QuantiFERON-TB Gold testing (or IGRA equivalent, such as T-SPOT TB test), both will be performed. If either PPD or QuantiFERON-TB Gold (or IGRA equivalent, such as T-SPOT TB test) is positive, the TB test is considered positive.

- If the first QuantiFERON-TB Gold (or IGRA equivalent) test is indeterminate, then the investigator should perform a second QuantiFERON-TB Gold (or IGRA equivalent) test to rule out a positive test result. The second QuantiFERON-TB Gold (or IGRA equivalent) test should be performed centrally; if not possible to be performed centrally, a local laboratory result may be acceptable. If testing remains indeterminate after the second QuantiFERON-TB Gold (or IGRA equivalent) test, then a PPD skin test will be performed.

8.3.5.2.2. Chest X-Ray

Participants can have a CXR (or alternative imaging method, such as MRI or CT) at any time during the study as warranted based on the opinion of the investigator (or designee). The CXR requires posterior-anterior and lateral views. A radiologist or pulmonologist must perform and document an assessment of the CXR (or alternative imaging method). The investigator (or designee) will indicate the clinical significance of any findings and will sign and date the report. In the assessment of the CXR (or alternative imaging method), the investigator (or designee) must indicate the presence or absence of (1) calcified granulomas, (2) pleural scarring/thickening, and (3) signs of active TB. If the CXR (or alternative imaging method) demonstrates changes suggestive of previous TB (eg, calcified nodule, fibrotic scar, apical or basilar pleural thickening) or other findings that are clinically significant, the investigator (or designee) should contact the sponsor's medical monitor (or designee) before randomizing the participant. Participants who have already been randomized in the study but have a CXR (or alternative imaging method) after randomization that is clinically significant for TB must be discontinued.

8.3.5.2.3. Tuberculosis Prophylaxis

At screening, if the participant has evidence of latent TB infection, prophylactic treatment must be initiated at least 2 months prior to administration of the first dose of study drug (or per local guidelines, whichever is longer). Prophylaxis needs to be completed per local guidelines; however, the full course of prophylaxis does not need to be completed prior to the first dose of study drug.

Note: Rifampicin or rifapentine are not allowed for TB prophylaxis. See Section 6.6.8 for a list of other prohibited medications.

Participants with a prior history of latent TB who have documented completion of a full course of anti-TB therapy may be allowed to be randomized in the study, provided nothing has changed in the participant's medical history to warrant repeat treatment. For participants with completion of a full course of anti-TB therapy but insufficient documentation, the investigator should consult with the sponsor's medical monitor (or designee) before randomizing the participant.

During the study, participants with new evidence of TB must be discontinued.

8.3.5.3. HIV Testing

All participants will be tested for the presence of HIV at screening. Participants will be screen failed if they test positive for HIV infection.

8.3.5.4. Hepatitis Testing

All participants will be tested for the presence of HBV and HCV at screening.

8.3.5.4.1. Hepatitis B Virus

Participants must undergo screening for HBV at the study visits indicated in Table 3. At a minimum, this includes testing for HBsAg, HBcAb, and HBsAb.

A diagram is shown in Table 16, with further explanation below.

Table 16: Interpretation and Management of HBV Serologic Test Results

Scenario	Surface Antigen (HBsAg)	Core Antibody (HBcAb)	Surface Antibody (HBsAb)	Status
A	Positive	Negative or positive	Negative or positive	Not eligible
B	Negative	Negative	Negative	Eligible
C	Negative	Negative	Positive	**HBV DNA PCR required**<br>Positive results: not eligible<br>Negative results: eligible
D	Negative	Positive	Positive	
E	Negative	Positive	Negative	

For participants in Scenarios C, D, and E:

- If the HBV DNA test is negative, the participant is eligible and may be randomized.
  - For participants who test HBV DNA negative and are randomized in the study: an HBV DNA PCR test should be repeated approximately every 12 weeks (as outlined in Table 3). HBV DNA PCR testing approximately every 12 weeks after randomization is not necessary for participants in Scenario C with a documented history of HBV vaccination.
- If the HBV DNA test is positive, the participant is NOT eligible and will be screen failed.
- In the event the HBV DNA test cannot be performed, the participant is NOT eligible and will be screen failed.

8.3.5.4.2. Hepatitis C Virus

Blood samples for HCV serology will be obtained at the screening visit and tested. A positive HCVAb will trigger an HCV RNA test (reflex test). A participant will not be eligible and will be screen failed if test results indicate active hepatitis C (HCV RNA detectable in any participant with anti-HCVAb). Participants who test positive for HCV but are successfully treated for HCV infection and have their screening visit at least 12 weeks after the last dose of HCV therapy may be eligible if HCV RNA is undetectable.

CCI [redacted]

CCI

[redacted]

8.6. Unscheduled Visits

Unscheduled visits may occur at any time medically warranted. Any assessments performed at those visits should be recorded in the eCRF.

8.7. End of Treatment and Early Termination

The EOT1 coincides with the Week 12 visit, and the EOT2 (EXT period) coincides with the Week 54 visit. A participant who completes the Week 12/EOT1 visit will have completed the PC period and may continue into the EXT period. A participant who completes the Week 54/EOT2 visit will have completed the EXT period.

If a decision is made that the participant will permanently discontinue study drug prior to the Week 54/EOT2 visit, then the ET visit should be conducted and will be determined as ET1 (if during the PC period) or ET2 (if during the EXT period). If the ET visit coincides with a regular study visit, then the ET evaluations will supersede those of that scheduled visit, and the data should be entered in the ET folder in the eCRF. If this decision does not coincide with a regular visit, reasonable efforts should be made to have the participant return to the site to complete the ET procedures.

8.8. Follow-Up

8.8.1. Safety Follow-Up

For participants who do not participate in the separate extension study, the safety follow-up period is the interval between the EOT visit and the scheduled follow-up visit, which should occur 30 (+ 7) days after the EOT visit (or after the last dose of study drug if the EOT visit was not performed). Adverse events and SAEs must be reported up until 1) at least 30 days after the last dose of study drug or 2) until toxicities resolve, return to baseline, or are deemed irreversible, whichever is longer. Reasonable efforts should be made to have the participant return for the follow-up visit and report any AEs that may occur during this period. In exceptional cases when the participant cannot return to the site for the safety follow-up visit, the participant should be contacted by telephone for assessment of AEs and SAEs and the site should properly document the contact.

9. ADVERSE EVENTS: DEFINITIONS AND PROCEDURES FOR RECORDING, EVALUATING, FOLLOW-UP, AND REPORTING

9.1. Definition of Adverse Event

**Adverse Event Definition**
• An AE is any untoward medical occurrence associated with the use of a drug in humans, whether or not it is considered drug-related.<br>• An AE can therefore be any unfavorable or unintended sign (including an abnormal laboratory finding), symptom, or disease (new or exacerbated) temporally associated with the use of study drug.
**Additional Guidance for Events Meeting the Adverse Event Definition**
• Any safety assessments (eg, ECG, vital signs measurements), including those that worsen from baseline, considered clinically significant in the medical and scientific judgment of the investigator (ie, not related to worsening of underlying disease) are to be reported as an AE.<br>• Abnormal laboratory test results are to be reported as an AE if they are considered clinically meaningful, induce clinical signs or symptoms, require concomitant therapy, or require changes in study drug. Whenever possible, a diagnosis (eg, anemia, thrombocytopenia) should be recorded in the eCRF rather than the abnormal laboratory test result (eg, low hemoglobin, platelet count decreased).<br>• Exacerbation of a chronic or intermittent pre-existing condition/disease, including either an increase in the frequency and/or intensity of the condition, is to be reported as an AE.<br>• New conditions detected or diagnosed after the start of study drug administration are to be reported as an AE.<br>• Signs, symptoms, or the clinical sequelae of a suspected drug-drug interaction are to be reported as an AE.<br>• Signs and/or symptoms from dose administration errors of a study drug (eg, overdose) or a concomitant medication are to be reported as an AE.<br>• "Lack of efficacy," "disease worsening," or "failure of expected pharmacological action" will not be reported as an AE or SAE. Such instances will be captured in the efficacy assessments.<br>• A condition that leads to a medical or surgical procedure (eg, endoscopy, appendectomy) will be reported as an AE if it occurs after obtaining informed consent. If the condition is present before entering the study, then it should be captured as medical history.<br>• Pre-existing diseases or conditions with expected fluctuations in signs or symptoms should be reported as an AE only if the investigator judges the fluctuation to have worsened more than expected during study participation.

9.2. Definition of Serious Adverse Event

If an event is not an AE per the definition above, then it cannot be an SAE even if serious conditions are met (eg, hospitalization for signs/symptoms of the disease under study, death due to progression of disease).

**A serious adverse event is defined as any untoward medical occurrence that, at any dose:**
**a. Results in death**
**b. Is life-threatening**<br>The term "life-threatening" in the definition of "serious" refers to an adverse drug experience that places the participant, in the opinion of the initial reporter, at immediate risk of death from the adverse experience as it occurs. This does not include an adverse drug experience that, had it occurred in a more severe form, might have caused death.
**c. Requires inpatient hospitalization or prolongation of existing hospitalization**<br>In general, hospitalization signifies that the participant has been detained (involving at least an overnight stay) at the hospital or emergency department for observation and/or treatment that would not have been appropriate in the physician's office or outpatient setting. Complications that occur during hospitalization are AEs. If a complication prolongs hospitalization or fulfills any other serious criteria, the event is serious. When in doubt as to whether hospitalization occurred or was necessary, the AE should be considered serious.<br>Hospitalization for elective treatment or planned surgery (eg, stent replacement, hip surgery) is not considered an SAE.<br>Hospitalization for medical interventions in which no unfavorable medical occurrence occurred (ie, elective procedures or routine medical visits) is not considered an SAE.
**d. Results in persistent or significant disability/incapacity**<br>The term "disability" means a substantial disruption of a person's ability to conduct normal life functions. This definition is not intended to include experiences of relatively minor medical significance, such as uncomplicated headache, nausea, vomiting, diarrhea, influenza, and accidental trauma (eg, sprained ankle), that may interfere with or prevent everyday life functions but do not constitute a substantial disruption.
**e. Is a congenital anomaly/birth defect**
**f. Is an important medical event**<br>An important medical event is an event that may not result in death, be immediately life-threatening, or require hospitalization but may be considered serious when, based on appropriate medical judgment, the event may jeopardize the participant and may require medical or surgical intervention to prevent one of the outcomes listed in the above definition. Examples of such events include new invasive or malignant cancers; intensive treatment in an emergency department or at home for allergic bronchospasm, blood dyscrasias, or convulsions that do not result in hospitalization; development of drug dependency or drug abuse; or suspected transmission of an infectious agent via a medicinal product. Secondary malignancies should always be considered SAEs**.**<br>**For Japan:** An event that may lead to disability is also considered an important medical event. It includes a case that is exposed to a risk of dysfunction to an extent that interferes with daily life when the adverse drug reaction occurs. It does not include an adverse drug reaction that, had the reaction been more severe, may have caused disability.

9.3. Recording and Follow-Up of Adverse Events and/or Serious Adverse Events

**Adverse Event and Serious Adverse Event Recording**
• An AE/SAE that begins or worsens after informed consent is signed should be recorded on the Adverse Events Form in the eCRF. All AEs/SAEs should be reported for enrolled participants, but only SAEs need to be reported for screen failure participants. For enrolled participants, conditions that were present at the time informed consent was given should be recorded on the Medical History Form in the eCRF. For detailed information, refer to the eCRF guidelines.<br>• When an AE/SAE occurs, it is the responsibility of the investigator to review all documentation (eg, hospital progress notes, laboratory reports, and diagnostic reports) related to the event.<br>• The investigator (or designee) will then record all relevant AE/SAE information in the eCRF.<br>• It is **not** acceptable for the investigator to send photocopies of the participant's medical records in lieu of completing the Adverse Events Form in the eCRF.<br>• There may be rare instances when copies of medical records for certain cases are requested. In this case, all participant identifiers, with the exception of the participant number, will be redacted by the site staff on the copies of the medical records before submission. These records can be submitted to Incyte Pharmacovigilance by email/fax per the contact information listed in the Study Binder as per SAE completing guidelines.<br>• The investigator will attempt to establish a diagnosis of the event based on signs, symptoms, and/or other clinical information. Whenever possible, the diagnosis (not the individual signs/symptoms) will be documented as the AE/SAE. When a clear diagnosis cannot be identified, each sign or symptom should be reported as a separate AE/SAE.<br>To the extent possible, each AE/SAE should be evaluated to determine the following:<br>• The severity grade (CTCAE v5.0 Grade 1 to 5). See below for further instructions on the assessment of intensity.<br>• Whether there is at least a reasonable possibility that the AE is related to the study drug: suspected (yes) or not suspected (no). See below for further instructions on the assessment of causality.<br>• The start and end dates, unless unresolved at the final safety follow-up visit.<br>• The action taken with regard to study drug as a result of the AE/SAE(s).<br>• The event outcome (eg, not recovered/not resolved, recovered/resolved, recovering/resolving, recovered/resolved with sequelae, fatal, unknown).<br>• The seriousness, as per the SAE definition provided in Section 9.2.<br>• The action taken with regard to the event. Note: If an AE is treated with a concomitant medication or nondrug therapy, this action should be recorded on the Adverse Events Form and the treatment should be specified on the appropriate eCRF (eg, Prior/Concomitant Medications, Procedures and Non-Drug Therapy).

Assessment of Intensity
The severity of AEs will be assessed using CTCAE v5.0 Grades 1 through 5. If an event is not classified by CTCAE, the severity of the AE will be graded according to the scale below to estimate the grade of severity.<br>The investigator will make an assessment of intensity for each AE and SAE reported during the study and assign it to 1 of the following categories:<br>• **Grade 1:** Mild; asymptomatic or mild symptoms; clinical or diagnostic observations only; treatment not indicated.<br>• **Grade 2:** Moderate; minimal, local, or noninvasive treatment indicated; limiting age-appropriate activities of daily living.<br>• **Grade 3:** Severe or medically significant but not immediately life-threatening; hospitalization or prolongation of hospitalization indicated; disabling; limiting self-care activities of daily living.<br>• **Grade 4:** Life-threatening consequences; urgent treatment indicated.<br>• **Grade 5:** Fatal.
**Assessment of Causality**
• The investigator is obligated to assess the relationship between study drug and each occurrence of each AE/SAE.<br>• A "reasonable possibility" of a relationship conveys that there are medical facts, evidence, and/or arguments to suggest a causal relationship, rather than that a relationship cannot be ruled out.<br>• The investigator will use clinical judgment to determine the possibility of a relationship.<br>• The investigator will also consult the RSI in the IB or Product Information for study drug or marketed products, respectively, in making their assessment.<br>• Alternative causes, such as underlying or concurrent disease(s), concomitant therapy, and other risk factors, as well as the temporal relationship of the event to study drug administration, will be considered and investigated.<br>• For each AE/SAE, the investigator **must** document in the medical notes that they have reviewed the AE/SAE and have provided an assessment of causality.<br>• With regard to assessing causality of SAEs:<br>– There may be situations in which an SAE has occurred and the investigator has minimal information to include in the initial report. However, the causality assessment is one of the criteria used when determining regulatory reporting requirements. **Therefore, it is very important that the investigator always make an assessment of causality based on the available information for every event before the initial transmission of the SAE.**<br>– The investigator may change their opinion of causality in light of follow-up information and submit the updated causality assessment.

**Follow-Up of Adverse Events and Serious Adverse Events**
• The investigator is obligated to perform or arrange for the conduct of supplemental measurements and/or evaluations as medically indicated or as requested by the sponsor to elucidate the nature and/or causality of the AE or SAE as fully as possible. This may include additional laboratory tests or investigations, histopathological examinations, or consultation with other health care professionals.
• Once an AE is detected, it should be followed in the Adverse Events Form in the eCRF until it has resolved or until it is judged to be permanent; assessment should be made at each visit (or more frequently if necessary) of any changes in severity, the suspected relationship to the study drug, the interventions required to treat the event, and the outcome.
• When the severity of an AE changes over time for a reporting period (eg, between visits), each change in severity will be reported as a separate AE.
• If a participant dies during participation in the study or during a recognized follow-up period, the investigator will provide the sponsor with a copy of any postmortem findings, including histopathology.
• Updated SAE information will be recorded in the originally completed eCRF and reported to Incyte Pharmacovigilance or its designee as directed in the Study Binder until resolution, stabilization, the event is otherwise explained, or the participant is lost to follow-up.
• Any updated SAE data (including SAEs being downgraded to nonserious) will be submitted to the sponsor (or designee) within 24 hours of receipt of the information.

9.4. Reporting of Serious Adverse Events

Regardless of suspected causality (eg, relationship to study drug or study procedure[s]), all SAEs occurring after the participant has signed the ICF through 30 days after the last dose of study drug must be reported to the sponsor (or designee) immediately, without undue delay but not later than within **24 hours** of obtaining knowledge of its occurrence unless otherwise specified by the Protocol. The investigator will submit any updated SAE data to the sponsor (or designee) immediately, without undue delay but not later than within 24 hours of it being available. **For Japan:** This information must also be reported immediately to the head of the study site.

Investigators are not obligated to actively seek SAE information after the safety follow-up visit or more than 30 days after the last dose of study drug. If the investigator learns of any SAE, including death, at any time during this period, and they consider the event to be reasonably related to the study drug or study participation, then the investigator must notify the sponsor (or designee) within 24 hours of becoming aware of the event.

After the initial AE/SAE report, the investigator is required to proactively follow each participant at subsequent visits/contacts. All SAEs and AESIs (as defined in Section 9.6.1) will be followed until resolution, stabilization, the event is otherwise explained, or the participant is lost to follow-up (as defined in Section 7.3).

Prompt notification by the investigator to the sponsor regarding an SAE is essential so that legal obligations and ethical responsibilities for the safety of participants and the safety of a study drug under clinical investigation are met.

If the SAE is not documented in the RSI of the IB for the study drug (new occurrence) and is thought to be related to the study drug, the sponsor or its designee may urgently require further

information from the investigator for expedited reporting to health authorities. The sponsor or its designee may need to issue an Investigator Notification to inform all investigators involved in any study with the same drug that this SAE has been reported. Suspected unexpected serious adverse reactions will be collected and reported to the competent authorities and relevant ethics committees following the sponsor's SOPs and in accordance with Clinical Trials Regulation (EU) No 536/2014, Clinical Trials Information System guidelines, or as per national regulatory requirements in participating countries.

For Japan: The sponsor will report suspected expected deaths and life-threatening events to the PMDA as per local regulatory requirements.

The sponsor has a legal responsibility to notify both the local regulatory authority and other regulatory agencies about the safety of a study drug under clinical investigation. The sponsor will comply with country-specific regulatory requirements relating to safety reporting to the regulatory authority, IRB/IEC, and investigators.

Investigator safety reports must be prepared for suspected unexpected serious adverse reactions according to local regulatory requirements and sponsor policy and forwarded to investigators as necessary.

An investigator who receives an investigator safety report describing an SAE or other specific safety information (eg, summary or listing of SAEs) from the sponsor will review and then file it along with the IB and will notify the IRB/IEC, if appropriate, according to local requirements.

**Serious Adverse Event Reporting**
• Information about all SAEs is collected and recorded on the Adverse Events Form in the eCRF.<br>• The investigator must report within 24 hours of learning of its occurrence any SAE via the EDC system (primary method) or by completing the Serious Adverse Event Report Form in English (only if the EDC system is not available). The contact information for Incyte Pharmacovigilance by email/fax is listed in the Incyte Reference Guide for Completing the Serious Adverse Event Report Form.<br>• In circumstances where the EDC system is not accessible for reporting SAE information (initial and/or follow-up SAE information) to the sponsor within 24 hours, refer to the Incyte Reference Guide for Completing the Serious Adverse Event Report Form. Once the EDC system is functional, the SAE report should be retrospectively added to the EDC system and follow-up should be completed through the EDC. The original copy of the Serious Adverse Event Report Form and the email or facsimile confirmation sheet must be kept at the study site (refer to the Incyte Reference Guide for Completing the Serious Adverse Event Report Form for details and for the email address or fax number).<br>• Follow-up information is also recorded in the eCRF and transmitted to Incyte Pharmacovigilance via the EDC system. The follow-up report should include information that was not provided previously, such as the outcome of the event, treatment provided, action taken with study drug because of the SAE (eg, dose reduced, interrupted, or discontinued), or participant disposition (eg, continued or withdrew from study participation). Each recurrence, complication, or progression of the original event should be reported as follow-up to that event, regardless of when it occurs.<br>• Contacts for SAE reporting can be found in the Study Binder.

9.5. Potential Drug-Induced Liver Injury

Details are presented in Appendix D.

Follow the SAE and follow-up reporting requirements per Section 9.2 as potential DILI AEs may be potential SAEs classified in the category of important medical event.

CCI [redacted]

9.7. Emergency Unblinding of Treatment Assignment

In case of a medical emergency, for a participant's safety management, the procedure for emergency unblinding is provided to the investigator in the IRT Manual. The decision to break the treatment code in an emergency situation resides at the sole discretion of the investigator.

If a participant's treatment assignment is unblinded, the sponsor or its designee should be notified immediately, without disclosure of the actual treatment assignment, by telephone for awareness.

If an investigator, the site personnel performing assessments, or a participant is unblinded, then the participant must discontinue study drug unless there are ethical reasons to have the participant remain on the study treatment. In these cases, the investigator must obtain specific approval from the sponsor's (or its designee's) medical monitor for the participant to continue in the study.

9.8. Pregnancy

Pregnancy, in and of itself, is not regarded as an AE unless there is suspicion that the study drug may have interfered with the effectiveness of a contraceptive medication or method. When a pregnancy has been confirmed in a participant during maternal or paternal exposure to study drug, the following procedures should be followed in order to ensure safety:

- The study drug must be discontinued immediately (female participants only).
- The investigator must complete and submit the Incyte Clinical Trial Pregnancy Form to the sponsor or its designee within **24 hours** of learning of the pregnancy.

Data on fetal outcome are collected for regulatory reporting and drug safety evaluations. Follow-up should be conducted for each pregnancy to determine outcome, including spontaneous or voluntary termination, details of the birth, and the presence or absence of any birth defects, congenital abnormalities, or maternal and/or newborn complications, by following until the first well-baby visit. Pregnancy should be recorded on a Clinical Trial Pregnancy Form and reported by the investigator to the sponsor or its designee. Pregnancy follow-up information should be recorded on the same form and should include an assessment of the possible causal relationship to the sponsor's study drug to any pregnancy outcome, as well as follow-up to the first well-baby visit or the duration specified in local regulations, whichever is later. Refer to the Incyte Reference Guide for Completing the Clinical Trial Pregnancy Form in the Study Binder.

Any SAE occurring during the pregnancy of a study participant must be recorded and reported as described in Section 9.4.

Abnormal pregnancy outcomes (eg, spontaneous abortion, fetal death, stillbirth, congenital anomalies, or ectopic pregnancy) are considered SAEs (if occurring in the study participant) and must be reported as described in Section 9.4. If an abnormal pregnancy outcome is reported in a study participant's partner, the event should be reported to the sponsor on the Clinical Trial Pregnancy Form.

9.9. Warnings and Precautions

Special warnings or precautions for the study drug, derived from safety information collected by the sponsor or its designee, are presented in the IB. Additional safety information collected between IB updates will be communicated in the form of Investigator Notifications. Any important new safety information should be discussed with the participant during the study as necessary. If new significant risks are identified, they will be added to the ICF.

9.10. Product Complaints

The sponsor collects product complaints on study drugs and drug delivery systems used in clinical studies in order to ensure the safety of study participants, monitor quality, and facilitate process and product improvements.

All product complaints associated with material packaged, labeled, and released by the sponsor or its designee will be reported to the sponsor. All product complaints associated with other study material will be reported directly to the respective manufacturer.

The investigator or their designee is responsible for reporting a complete description of the product complaint via email or other written communication to the sponsor contact or respective manufacturer as noted in the packaging information. Any AE associated with a product complaint should be recorded as described in Section 9.3.

If the investigator is asked to return the product for investigation, they will return a copy of the product complaint communication with the product.

For Japan: Complaints associated with unapproved medical devices will be reported to the sponsor with a Medical Device Defect Report Form, and the sponsor will report medical device defects to the PMDA as per local regulatory requirements.

9.11. Treatment of Overdose

There has been no clinical experience with overdose of povorcitinib. Treatment of overdose should consist of general supportive measures.

10. STATISTICS

10.1. Sample Size Determination

The primary endpoint of this study is the proportion of participants who achieve HiSCR at Week 12. Participants will be randomized in a 1:1:1 ratio to 1 of 2 doses of povorcitinib ([redacted] or [redacted] mg QD) or placebo [redacted]

Based on the observed HiSCR rates at Weeks 8 and 12 reported in a Phase 2, randomized, dose-ranging study of povorcitinib (INCB 54707-204), it is assumed that the HiSCR rate at Week 12 would be [redacted]% for the povorcitinib [redacted] mg group, [redacted]% for the povorcitinib [redacted] mg group, and 30% for the placebo group. Using the chi-square test, without continuity correction, at a 2-sided $\alpha = 0.025$, a sample size of 200 participants per group (with a total of 600 participants) will achieve approximately [redacted]% power to detect a statistically significant difference between the povorcitinib [redacted] mg group and the placebo group and approximately [redacted]% power to detect a statistically significant difference between the povorcitinib [redacted] mg and the placebo group.

The powers for primary and key secondary endpoints are provided in Table 17.

[redacted]

10.2. Populations for Analysis

The populations for analysis are provided in Table 18.

Table 18: Populations for Analysis

Population	Description
ITT	The ITT population includes all randomized participants. Treatment groups for this population will be defined according to the treatment assignment at randomization.<br>The ITT population will be used for the summary of demographics, baseline characteristics, participant disposition, and analyses of all efficacy data in the PC period.
PP	The PP population includes randomized participants who are considered to be sufficiently compliant with the Protocol. In general, the following are considered important protocol deviations that may significantly affect the primary analysis:<br>• Missing data for the primary endpoint<br>• Overall compliance less than 50% during the PC period<br>Participants with 1 or more such deviations will be excluded from the PP population. In addition, important protocol deviations related to inclusion/exclusion criteria, discontinuation criteria, and use of prohibited concomitant medications will be reviewed. Decisions as to whether any of these deviations warrant exclusion from the PP population will be made prior to database lock and randomization unblinding.<br>The PP population will be used in the supportive sensitivity analysis for the primary estimand.
Safety	The safety population includes all participants who received at least 1 dose of study drug during the PC period. Treatment groups for this population will be determined according to the actual treatment the participant received on Day 1.
Extension evaluable	The extension evaluable population includes all participants who received at least 1 dose of povorcitinib during the extension period.

CCI [redacted]

10.3. Level of Significance

For the primary endpoint and key secondary endpoints, the overall familywise Type I error rate is 2-sided $\alpha = 0.05$.

The following is a list of primary and key secondary null hypotheses to be tested, denoted by $H_{ij,0}$, where i indicates the povorcitinib dose group (i =1 for [redacted] mg, 2 for [redacted] mg) and j is the numerical identifier of the endpoint for the dose group (j = 1 for HiSCR at Week 12, j = 2 for HiSCR75 at Week 12, j = 3 for flare by Week 12, j = 4 for Skin Pain Response at Week 12).

Primary null hypotheses:

- $H_{11,0}$: Proportion of participants achieving HiSCR at Week 12 in the povorcitinib [redacted] mg group is equal to the placebo group
- $H_{21,0}$: Proportion of participants achieving HiSCR at Week 12 in the povorcitinib [redacted] mg group is equal to the placebo group

Key secondary null hypotheses:

- $H_{12,0}$: Proportion of participants achieving HiSCR75 at Week 12 in the povorcitinib [redacted] mg group is equal to the placebo group
- $H_{22,0}$: Proportion of participants achieving HiSCR75 at Week 12 in the povorcitinib [redacted] mg group is equal to the placebo group
- $H_{13,0}$: Proportion of participants with flare by Week 12 in the povorcitinib [redacted] mg group is equal to the placebo group
- $H_{23,0}$: Proportion of participants with flare by Week 12 in the povorcitinib [redacted] mg group is equal to the placebo group
- $H_{14,0}$: Proportion of participants achieving Skin Pain Response at Week 12 in the povorcitinib [redacted] mg group is equal to the placebo group
- $H_{24,0}$: Proportion of participants achieving Skin Pain Response at Week 12 in the povorcitinib [redacted] mg group is equal to the placebo group

where Skin Pain Response is defined as ≥ 3-point reduction from baseline in Skin Pain NRS for regulatory purposes in US/FDA/aligned countries or Skin Pain NRS30 for regulatory purposes in EU/EMA/aligned countries.

To adjust for multiplicity and provide strong control of overall familywise error rate, the graphic approach (Bretz et al 2011) will be used in the primary endpoint and the key secondary endpoints outlined in Figure 4.

In Step 1, within Family 1, the algorithm begins by testing the null hypotheses for each of the 2 povorcitinib dose groups versus placebo on the primary endpoint ($H_{11,0}$ and $H_{21,0}$) at its assigned significance level of 2-sided $\alpha = 0.025$. If any hypothesis is rejected (ie, deemed significant), then its significance level will be transferred to the subsequent test following the arrow. The numbers along the arrows denote the weights for transferring significance levels. Specifically, a weight of 1 indicates 100% transfer of the significance level. It should be noted it is possible that a hypothesis for a particular endpoint fails to be rejected the first time it is tested but subsequently receives enough significance level from successfully rejected hypotheses and is rejected the second time it is tested. This iterative process of updating the graph and redistribution of the significance level is continued until no further hypotheses can be tested within Family 1.

In Step 2, if all 4 null hypotheses within Family 1 are rejected, the 2 null hypotheses $H_{13,0}$ and $H_{14,0}$ for povorcitinib [redacted] mg versus placebo within Family 2 will be tested using Hochberg procedure at a significance level of 2-sided $\alpha = 0.05$.

In Step 3, if both null hypotheses $H_{13,0}$ and $H_{14,0}$ within Family 2 are rejected, the 2 null hypotheses $H_{23,0}$ and $H_{24,0}$ for povorcitinib [redacted] mg versus placebo within Family 3 will be tested using Hochberg procedure at a significance level of 2-sided $\alpha = 0.05$.

[redacted]

10.4. Statistical Analyses

Given the design, a 12-week analysis will be performed to access efficacy and safety during the PC, double-blind treatment period. This will occur after all participants have completed the study activities up to Week 12 and all data pertaining to the PC, double-blind period are cleaned.

The final analysis to summarize additional efficacy and safety (without statistical comparison to placebo) will be conducted once the last participant completes the last visit in the study.

The statistical analysis will be described in detail and fully documented in the SAP. The SAP will be finalized prior to the database lock for the 12-week analysis. This section is a summary of the planned statistical analyses of the primary and secondary endpoints.

10.4.1. Primary Analysis

10.4.1.1. Primary Estimand

The primary estimand with the 5 attributes and the strategies for addressing the defined intercurrent events is provided in Table 19. The primary analysis will be based on the ITT population. The primary alternative hypothesis (superiority of each povorcitinib dose group [[redacted] mg, [redacted] mg] compared with the placebo group) will be tested at a 2-sided α = 0.025 level using the CMH test stratified by randomization stratification factors. Stratum-adjusted HiSCR rate difference between each of the povorcitinib dose groups ([redacted] mg, [redacted] mg) versus placebo and the associated 95% CI will be computed using Mantel-Haenszel weights (Mantel and Haenszel 1959). The stratified CMH p-value and common odds ratio with 95% CI will be provided. The number of participants achieving HiSCR at Week 12 and the respective rates as well as the 2-sided 95% exact CIs based on the Clopper-Pearson method will also be reported by treatment group.

Table 19: Primary Estimand

Estimand Attribute	Definition
**Treatment**	Each of the povorcitinib dose groups ([redacted] mg, [redacted] mg) compared with placebo
**Population**	Participants with HS (as defined by trial inclusion/exclusion criteria)
**Variable**	Achievement of HiSCR at Week 12, defined as at least a 50% reduction from baseline in the total abscess and inflammatory nodule count with no increase from baseline in abscess or draining tunnel count at Week 12
**Intercurrent Events**	**Strategies for Addressing Intercurrent Events**
[redacted]	
**Population-level summary**	Proportion of participants achieving HiSCR at Week 12 in each treatment group, the difference in HiSCR rate between each of the povorcitinib dose groups ([redacted] mg, [redacted] mg) vs placebo.

Based on the composite strategy for handling intercurrent events of treatment discontinuation and initiation of systemic antibiotic rescue therapy or prohibited medication, participants who have any of the intercurrent events are considered as nonresponders for all subsequent visits after the occurrence date of the intercurrent event. As well, participants who miss a postbaseline value at a visit (ie, true missing values due to data not being collected) are imputed as nonresponders on that visit only.

10.4.1.2. Sensitivity Analysis

A sensitivity analysis for the primary estimand will be conducted using the treatment strategy to address the intercurrent events with all data included regardless of occurrence of any intercurrent event, followed by multiple imputation of missing values. A fully conditional specification method (van Buuren 2007) that assumes the existence of a joint distribution of all variables will be used to impute missing postbaseline values for HiSCR components, including abscess count, inflammatory nodule count, and draining tunnel count. For each of the 3 HiSCR components, a regression model including treatment group, randomization stratification factors, and baseline and scheduled postbaseline values up to Week 12 will be specified for the fully conditional specification method. The imputation will be repeated a number of times to generate corresponding complete datasets in order to reflect the uncertainty around the true values. After the missing HiSCR component values are imputed, the binary variables will be derived based on the definition. The stratified CMH test will be applied to each full dataset for the timepoint of interest, and then the results will be combined for the inference using Rubin's rule.

Another sensitivity analysis will be conducted using treatment strategy for intercurrent events following tipping point analysis to examine the potential effects of missing data. The missing HiSCR at Week 12 in each treatment group will be replaced by a range of values from the most conservative scenario to the least conservative scenario. For each of the povorcitinib dose groups ([redacted] mg, [redacted] mg), the most conservative scenario is that all the missing cases from participants in the povorcitinib dose group are nonresponders and all the missing cases from participants in the placebo group are responders, while the least conservative scenario is the other way around. For each scenario, comparisons between each of the povorcitinib dose groups ([redacted] mg, [redacted] mg) versus placebo will be performed using the Fisher exact test.

In addition, the primary estimand will be performed using the same stratified CMH test in the PP population as a supportive analysis.

10.4.1.3. Subgroup Analyses

Subgroup analysis for the primary efficacy endpoint will be performed based on the demographics and baseline characteristics, including but not limited to age, geographic region, Hurley stage, and baseline randomization stratification factors ([redacted]).

10.4.2. Secondary Analyses

10.4.2.1. Key Secondary Estimands

For the key secondary estimands, the 5 attributes and the strategies for addressing the defined intercurrent events are provided in Table 20.

Table 20: Key Secondary Estimands

Estimand Attribute	Definition
**Key secondary estimand for HiSCR75 at Week 12**	
**Treatment**	Same as for primary estimand
**Population**	Same as for primary estimand
**Variable**	Achievement of HiSCR75 at Week 12, defined as at least a 75% reduction from baseline in the total abscess and inflammatory nodule count with no increase from baseline in abscess or draining tunnel count
**Intercurrent Events**	**Strategies for Addressing Intercurrent Events**
[redacted]	
**Population-level summary**	Proportion of participants achieving HiSCR75 at Week 12 in each treatment group, the difference in HiSCR75 rate between each of the povorcitinib dose groups ([redacted] mg, [redacted] mg) vs placebo
**Key secondary estimand for flare by Week 12**	
**Treatment**	Same as for primary estimand
**Population**	Same as for primary estimand
**Variable**	Having flare at Week 12, defined as at least a 25% increase in the total abscess and inflammatory nodule count with a minimum increase of 2 relative to baseline
**Intercurrent Events**	**Strategies for Addressing Intercurrent Events**
[redacted]	

Table 20: Key Secondary Estimands (Continued)

Estimand Attribute	Definition
[redacted]	
**Population-level summary**	Proportion of participants with flare by Week 12 in each treatment group, the difference between each of the povorcitinib dose groups ([redacted] mg, [redacted] mg) vs placebo
**Key secondary estimand for Skin Pain Response at Week 12**	
**Treatment**	Same as for primary estimand
**Population**	Same as for primary estimand
**Variable**	Achievement of Skin Pain Response at Week 12, defined as ≥ 3-point reduction from baseline in Skin Pain NRS at Week 12 for regulatory purposes in US/FDA/aligned countries or Skin Pain NRS30 at Week 12 for regulatory purposes in EU/EMA/aligned countries
**Intercurrent Events**	**Strategies for Addressing Intercurrent Events**
[redacted]	
**Population-level summary**	Proportion of participants achieving Skin Pain Response at Week 12 in each treatment group, the difference in response rate between each of the povorcitinib dose groups ([redacted] mg, [redacted] mg) vs placebo

The key secondary analyses will be conducted based on the ITT population for HiSCR75 at Week 12 and flare by Week 12, the ITT population with baseline Skin Pain NRS score ≥ 3 for ≥ 3-point reduction in Skin Pain NRS at Week 12, and the ITT population with baseline Skin Pain NRS score ≥ 1 for Skin Pain NRS30 at Week 12. All key secondary efficacy endpoints will be analyzed using the stratified CMH method similarly as specified in the primary analysis. Similarly as for the primary analysis, the NRI approach will be used for addressing intercurrent events as well as true missing values due to data not collected.

10.4.2.2. Other Secondary and Exploratory Analyses

In the PC period, all other secondary and exploratory endpoints will be analyzed based on the ITT population.

For categorical endpoints, a similar stratified CMH test as specified in the primary analysis will be used, if applicable. Composite strategy will be used to handle the interrcurrent events followed by the NRI method to impute missing values.

For continuous efficacy endpoints at Week 12, treatment strategy will be used to handle the intercurrent events followed by multiple imputation of the missing values (details described in Section 10.4.1.2). The analysis of covariance with treatment group (povorcitinib [redacted] mg, povorcitinib [redacted] mg, and placebo), randomization stratification factors, and baseline measurement will be used to analyze each full dataset, and then the results will be combined for the inference using Rubin's rule. From this analysis, the estimated treatment difference between each of the povorcitinib dose groups ([redacted] mg, [redacted] mg) and the placebo group and the associated 95% CIs and the p-value will be presented.

As a supportive analysis, the continuous efficacy endpoints may be analyzed using the MMRM to include all available data at postbaseline visits up to Week 12. Participants who have a baseline value and at least 1 postbaseline value in the PC period will be included in the analysis. The MMRM will include the fixed effects of treatment group, randomization stratification factors, visit, treatment by visit interaction, and covariates of baseline measurements and baseline measurements by visit interaction. The variance-covariance matrix of the within-participant errors in the MMRM will be modeled as unstructured. The Kenward-Roger method will be used to estimate the degrees of freedom. Missing values will not be otherwise imputed using the MMRM approach. The least squares means along with 95% CIs and the p-value for the difference in mean change from baseline at each visit between each of the povorcitinib dose groups ([redacted] mg, [redacted] mg) and the placebo group from the MMRM will be provided.

Long-term efficacy endpoints (across the PC period and the EXT period) will be summarized using descriptive statistics based on the extension evaluable population. For categorical measurements, summary statistics will include sample size, frequency, and percentage. For continuous measurements, summary statistics will include sample size, mean, median, standard deviation, standard error of the mean, minimum, and maximum. Summary statistics for continuous measures will be provided for baseline, the actual measurements at each visit, and the change and percentage change from baseline at each visit, if applicable.

10.4.3. Safety Analyses

Safety analyses will be conducted for the safety population in the PC period, the extension evaluable population in the EXT period, and across both periods.

Adverse events will be coded using MedDRA. A TEAE is defined as any AE either reported for the first time or worsening of a pre-existing event after first dose of study drug until the end of the safety follow-up.

Severity of AEs will be based on the National Cancer Institute CTCAE v5.0 using Grades 1 through 5.

The subset of AEs considered by the investigator to have a causal relationship to study drug will be considered treatment-related AEs. If the investigator does not specify the relationship of the AE to study drug, then the AE will be considered treatment-related.

The number and percentages of participants experiencing TEAEs will be tabulated using the MedDRA system organ class and preferred term for all TEAEs, drug-related TEAEs, serious TEAEs, Grade 3 or higher TEAEs, TEAEs leading to discontinuation of study drug, and AESIs,

Long-term AE rates for each of the povorcitinib dose groups ([redacted] mg, [redacted] mg) will be analyzed using event rates adjusted by cumulative exposure. This include TEAEs under each povorcitinib dose exposure from participants starting on the povorcitinib dose and participants switching from placebo to the povorcitinib dose.

For clinical laboratory and vital signs, measured values, change from baseline, and percentage change from baseline will be summarized using descriptive statistics. Laboratory test values outside the normal range will be assessed for severity based on the normal ranges for the clinical reference laboratory. The incidence of abnormal laboratory values and shift tables relative to baseline will be tabulated.

10.4.4. Exploratory Analysis

[redacted]

10.5. Interim Analysis

No formal interim analysis is planned in this study. However, preplanned analyses of safety will be provided to an external DSMB (see Section 5.6) as specified in the DSMB charter. The process by which the DSMB will review data and make recommendations and decisions will be documented in the DSMB charter.

11. SUPPORTING DOCUMENTATION AND OPERATIONAL CONSIDERATIONS

11.1. Investigator Responsibilities

- The Protocol, Protocol Amendments, ICF, IB, and other relevant documents (eg, advertisements) must be submitted to an IRB/IEC by the investigator and reviewed and approved by the IRB/IEC and health authorities before the study is initiated.
- The investigator is responsible for ensuring that the safety reports provided by the sponsor are reviewed and processed in accordance with regulatory requirements, the policies and procedures established by the IRB/IEC, and institutional requirements.
- Any amendments to the Protocol will require approval from both health authorities and the IRB/IEC before implementation of changes made to the study design, except for changes necessary to eliminate an immediate hazard to study participants.
- The investigator will be responsible for the following:
  - Providing written summaries of the status of the study to the IRB/IEC annually or more frequently in accordance with the requirements, policies, and procedures established by the IRB/IEC.
  - Notifying the IRB/IEC of SAEs or other significant safety findings as required by IRB/IEC procedures.
  - Providing oversight of the conduct of the study at the site and adherence to GCP, IRB/IEC requirements, institutional requirements, and applicable laws and country-specific regulations.
- Adhering to the Protocol as described in this document and agreeing that changes to the Protocol procedures, with the exception of medical emergencies, must be discussed and approved, first, by the sponsor or its designee and, second, by the IRB/IEC. Each investigator is responsible for enrolling participants who have met the specified eligibility criteria.
- Retaining records in accordance with all local, national, and regulatory laws but for a minimum period of at least 2 years after the last marketing application approval in an ICH region and until there are no pending or contemplated marketing applications in an ICH region, or if not approved, 2 years after the termination of the test article for investigation to ensure the availability of study documentation should it become necessary for the sponsor or a regulatory authority to review.
  - The investigator must not destroy any records associated with the study during the retention period without receiving approval from the sponsor. The investigator must notify the sponsor or its designee in the event of accidental loss or destruction of any study records. If the investigator leaves the institution where the study was conducted, the sponsor or its designee must be contacted to arrange alternative record storage options.

  - All eCRF data entered by the site (including audit trail), as well as computer hardware and software (for accessing the data), will be maintained or made available at the site in compliance with applicable record retention regulations. The sponsor will retain the original eCRF data and audit trail.
- **For Japan:** The record retainer (delegated by the head of the study site) will retain the J-GCP–defined essential documentation at this site until the regulatory approval of the study drug or at least 3 years after the discontinuation or completion of the study conduct, whichever is later. If the sponsor requires retention of these documents for a longer period of time, the duration and method of retention will be decided upon through discussion between the sponsor and the study site. It is the responsibility of the sponsor to inform the head of the study site as to when the documents no longer need to be retained.

11.1.1. Identification of the Coordinating Principal Investigator

A coordinating principal investigator has been appointed by the sponsor. As part of her responsibilities, the coordinating principal investigator will review the final CSR. Agreement with the final CSR will be documented by the dated signature of the coordinating principal investigator.

11.2. Data Management

Data management will be performed in a validated EDC system. The investigator will be provided with access to an EDC system so that an eCRF can be completed for each participant.

The site will be provided with eCRF completion guidelines for instructions on data entry in the eCRF. The study monitor will reference the Monitoring Plan in order to ensure that each issue identified is appropriately documented, reported, and resolved in a timely manner in accordance with the plan's requirements. Other data outside the EDC system required in the study conduct of the Protocol, such as documents or results transmitted to the sponsor via a central laboratory or specialized technical vendors and as designated by the sponsor, will have their own data flow management plans, study charters, or biomarker plans, as applicable.

The sponsor (or designee) will be responsible for the following:

- Managing the integrity of the data and the quality of the conduct of the study, such as ensuring that study monitors perform ongoing source data verification to confirm that data entered into the eCRF by authorized site personnel are accurate, complete, and verifiable from source documents; that the safety and rights of participants are being protected; and that the study is being conducted in accordance with the currently approved Protocol and any other study agreements, ICH GCP, and all applicable regulatory requirements.
- Managing and reconciling the data generated and/or collected, including documents and results such as laboratory or imaging data analyzed centrally by a designated vendor of the sponsor.

The investigator will be responsible for the following:

- Recording, or ensuring the recording of, all relevant data relating to the study in the eCRF.
- Delivering, or ensuring the delivery of, all other results, documents, data, know-how, or formulas relating to the study to the sponsor or designee electronically and/or centrally (eg, laboratory data, imaging data, biomarker data, photographs, diary data) or as otherwise specified in the Protocol.
- Maintaining adequate and accurate source documents and trial records that include all pertinent observations on each of the site's trial participants. Source data should be attributable, legible, contemporaneous, original, accurate, and complete. Changes to source data should be traceable, should not obscure the original entry, and should be explained if necessary (eg, via an audit trail). Source data are, in general, all information in original records and certified copies of original records of clinical findings, observations, or other activities in a clinical trial necessary for the reconstruction and evaluation of the trial. Source data are contained in source documents (original records or certified copies).
- Verifying that data entries are accurate and correct by physically or electronically signing the eCRF.
- Maintaining accurate documentation (source data) that supports the information entered in the eCRF, sent to a central vendor designated by the sponsor, or as described in other study and data flow manuals.
  - Source documents provide evidence for the existence of the participant and substantiate the integrity of the data collected. Source documents are filed and available at the investigator's site. Examples of source documents are original documents, data, and records (eg, hospital records; electronic hospital records; clinical and office charts; laboratory notes; memoranda; participants' diaries or evaluation checklists; pharmacy dispensing records; recorded data from automated instruments; copies or transcriptions certified after verification as being accurate copies; microfiches; photographic negatives; microfilm or magnetic media; x-rays; participants' files; and e-records/records kept at the pharmacy, at the laboratories, and at medico-technical departments involved in the clinical trial).
  - Data entered in the eCRF that are transcribed from source documents must be consistent with the source documents or the discrepancies must be explained. The investigator may need to request previous medical records or transfer records, depending on the study. Current applicable medical records must be available.

- Sending participants' data, either as unique samples, copies, or photographs, to be evaluated centrally or analyzed centrally, or both, by a qualified vendor designated by the sponsor.
  - As required by privacy and data protection regulations and Incyte's privacy policies, if any photographs of participants are to be taken, the photographs must be limited to the area of the face or the body that is strictly necessary and the photographs should be masked (ie, identifying features such as eyes, mouth, scars, tattoos, or unique markings or features should be either obscured with a black bar or digitally pixelated so as to not permit the reidentification of the participants and preserve their confidentiality) by a specially designated photography vendor prior to sending the photographs to Incyte or any other third-party vendors for analysis or further processing.
  - **For France:** In accordance with French regulations, sites in France must perform the masking before the photographs are transferred, including to any specially designated photography vendor, Incyte, or any other third-party vendors for analysis or further processing. In addition, the participant's specific consent for photographs shall be collected.
- Permitting study-related monitoring, sponsor audits, IRB/IEC review, and regulatory inspections by providing direct access to source data and other relevant clinical study documents.
  - Monitoring: Qualified representatives of the sponsor or its designee, study monitors, will monitor the study according to a predetermined plan. The investigator must allow the study monitors to review any study materials and participant records at each monitoring visit.
  - Auditing: Qualified representatives of the sponsor or its designee may audit the clinical study site and study data to evaluate compliance with the Protocol, applicable local clinical study regulations, and overall study conduct. The investigator must allow the auditors to review original source records and study documentation for all participants.
  - Regulatory inspection: Regulatory authorities may conduct an inspection of the study and the site at any time during the development of an investigational product. The investigator and staff are expected to cooperate with the inspectors and allow access to all source documents supporting the eCRFs and other study-related documents. The investigator must immediately notify the sponsor when contacted by any regulatory authority for the purposes of conducting an inspection.

11.3. Data Quality Assurance

The sponsor assumes accountability for actions delegated to other individuals (eg, contract research organizations). The sponsor or designee is responsible for the data management of this study, including quality checking of the data. Further, monitoring details describing strategy, including definition of study-critical data items and processes (eg, risk-based initiatives in operations and quality such as risk management and mitigation strategies and analytical risk-based monitoring), methods, responsibilities, and requirements, including handling of noncompliance issues, Protocol deviations, and monitoring techniques (eg, central, remote, or on-site monitoring) are provided in the relevant study plans.

Quality tolerance limits will be predefined in the Risk Assessment Mitigation Plan and Integrated Project Management Plan as per CRO SOPs to identify systematic issues that can impact participants' safety, efficacy results and analysis, and/or reliability of study results. These predefined parameters will be monitored during the study and can be adjusted during the study upon data review. Important deviations from the quality tolerance limits and remedial actions taken, including reporting to IRBs/IECs and health authorities if applicable, will be summarized in the CSR.

11.4. Data Privacy and Confidentiality of Study Records

The investigator and the sponsor or its designee must adhere to applicable data protection laws and regulations. The investigator and the sponsor or its designee are responsible for ensuring that personal information is handled in accordance with local data protection laws (including but not limited to HIPAA and GDPR) as applicable, and the sponsor operates comprehensive data privacy and data security programs that are applicable to this study. Appropriate notice, or notice and consent (as may be required by each applicable jurisdiction), for collection, use, disclosure, and/or transfer (if applicable) of personal information must be obtained in accordance with local data protection laws. Appropriate data protection terms that comply with applicable laws will be included in relevant study agreements.

To ensure confidentiality of records and protect personal data, participant names will not be supplied to the sponsor or its designee. Only the participant number will be recorded in the eCRF; if the participant's name appears on any other document (eg, laboratory report), it must be obliterated on the copy of the document to be supplied to the sponsor or its designee. Study findings stored on a computer will be stored in accordance with appropriate technical and organizational measures as required by local data protection laws.

In the event of a data breach involving participant data, the sponsor or its designee will follow the sponsor's incident response procedures. The precise definition of a data breach varies in accordance with applicable law but may generally be understood as a breach of security leading to the accidental or unlawful destruction, loss, alteration, unauthorized disclosure of, or access to, personal data. In accordance with its incident response procedures, the sponsor will assess the breach to consider its notification and remediation obligations under applicable law.

11.5. Financial Disclosure

Before study initiation, all clinical investigators participating in clinical studies subject to FDA Regulation Title 21 CFR Part 54 – Financial Disclosure by Clinical Investigators (ie, "covered studies") are required to submit a completed Clinical Investigator Financial Disclosure Form that sufficiently details any financial interests and arrangements that apply. For the purpose of this regulation, "clinical investigator" is defined as any investigator or subinvestigator who is directly involved in the treatment or evaluation of research participants, including the spouse and each dependent child of the clinical investigator or subinvestigator. These requirements apply to both US and foreign clinical investigators conducting covered clinical studies.

Any new clinical investigators added to the covered clinical study during its conduct must also submit a completed Clinical Investigator Financial Disclosure Form. During a covered clinical study, any changes to the financial information previously reported by a clinical investigator must be reported to the sponsor or its designee. At the conclusion of the covered clinical study, the clinical investigators will be reminded of their obligations. In the event that the clinical investigator is not reminded, they nevertheless will remain obligated to report to the sponsor or its designee any changes to the financial information previously reported, as well as any changes in their financial information for a period of 1 year after completion of the covered clinical study.

11.6. Publication Policy

By signing the study Protocol, the investigator and their institution agree that the results of the study may be used by the sponsor, Incyte Corporation, for the purposes of national and international registration, publication, and information for medical and pharmaceutical professionals. Study results will be published in accordance with applicable local and national regulations. If necessary, the authorities will be notified of the investigator's name, address, qualifications, and extent of involvement. The terms regarding the publication of study results are contained in the agreement signed with the sponsor or its designee. A signed agreement will be retained by the sponsor or its designee.

The results of this study may be published or presented at scientific meetings. If this is foreseen, the investigator agrees to submit all manuscripts or abstracts to the sponsor before submission. This allows the sponsor to protect proprietary information and to provide comments.

The sponsor will comply with the requirements for publication of study results. In accordance with standard editorial and ethical practice, the sponsor will generally support publication of multicenter studies only in their entirety and not as individual site data. In this case, a coordinating investigator will be designated by mutual agreement.

Authorship will be determined in line with International Committee of Medical Journal Editors authorship requirements.

11.7. Study and Site Closure

The sponsor or designee reserves the right to close the study site or terminate the study at any time for any reason at the sole discretion of the sponsor. Study sites will be closed upon study completion. A study site is considered closed when all required documents and study supplies have been collected and a study-site closure visit has been performed.

For Japan: When the trial is completed, the investigator should inform the head of the study site of the completion in writing and submit a written summary of the trial's outcome, and then the head of the study site should promptly inform the IRB and sponsor or designee of the completion in writing.

The investigator may initiate study-site closure at any time, provided there is reasonable cause and sufficient notice is given in advance of the intended termination.

Reasons for the early closure of a study site by the sponsor or investigator may include but are not limited to:

- Failure of the investigator to comply with the Protocol, the requirements of the IRB/IEC or local health authorities, the sponsor's procedures, or GCP guidelines.
- Inadequate recruitment of participants by the investigator.
- Discontinuation of further study treatment development.

12. REFERENCES

Alavi A, Hamzavi I, Brown K, et al. Janus kinase 1 inhibitor INCB054707 for patients with moderate-to-severe hidradenitis suppurativa: results from two phase II studies. Br J Dermatol 2022;186:803-813.

Amir Ali A, Seng EK, Alavi A, Lowes MA. Exploring changes in placebo treatment arms in hidradenitis suppurativa randomized clinical trials: a systematic review. J Am Acad Dermatol 2020;82:45-53.

Bató A, Brodszky V, Gergely LH, et al. The measurement performance of the EQ-5D-5L versus EQ-5D-3L in patients with hidradenitis suppurativa. Qual Life Res 2021;30:1477-1490.

Bretz F, Posch M, Glimm E, Klinglmueller F, Maurer W, Rohmeyer K. Graphical approaches for multiple comparison procedures using weighted Bonferroni, Simes, or parametric tests. Biom J 2011;53:894-913.

Cella D, Wilson H, Shalhoub H, et al. Content validity and psychometric evaluation of Functional Assessment of Chronic Illness Therapy-Fatigue in patients with psoriatic arthritis. J Patient Rep Outcomes 2019;3:30.

Chen SX, Greif C, Gibson RS, Porter ML, Kimball AB. Advances in biologic and small molecule therapies for hidradenitis suppurativa. Exp Opin Pharmacother 2022;23:959-978.

Chu C-B, Yang C-C, Tsai S-J. Global data analysis supports smoking as the fundamental element associated with geographical sex disparities in hidradenitis suppurativa. Br J Dermatol 2021;185:1054-1056.

Clinical Trials Facilitation and Coordination Group. Recommendations related to contraception and pregnancy testing in clinical trials. 2020.

Fernandez JM, Rizvi OH, Marr KD, et al. Itch and pain by lesion morphology in hidradenitis suppurativa patients. Int J Dermatol 2021;60:56-59.

Food and Drug Administration. FDA Drug Safety Communication: FDA requires warnings about increased risk of serious heart-related events, cancer, blood clots, and death for JAK inhibitors that treat certain chronic inflammatory conditions. 2021.

Finlay AY, Khan GK. Dermatology Life Quality Index (DLQI) — a simple practical measure for routine clinical use. Clin Exp Dermatol 1994;19:210-216.

Frew JW, Hawkes JE, Krueger JG, Topical, systemic and biologic therapies in hidradenitis suppurativa: pathogenic insights by examining therapeutic mechanisms. Ther Adv Chronic Dis 2019;10:1-24.

Frew JW, Jiang CS, Singh N, et al. Clinical response rates, placebo response rates, and significantly associated covariates are dependent on choice of outcome measure in hidradenitis suppurativa: a post hoc analysis of PIONEER 1 and 2 individual patient data. J Am Acad Dermatol 2020;82:1150-1157.

Frew JW, Lowes MA, Goldfarb N, et al. Global harmonization of morphological definitions in hidradenitis suppurativa for a proposed glossary. JAMA Dermatol 2021;157:449-455.

Hendricks AJ, Hsiao JL, Lowes MA, Shi VY. A comparison of international management guidelines for hidradenitis suppurativa. Dermatology 2021;237:81-96.

Herdman M, Gudex C, Lloyd A, et al. Development and preliminary testing of the new five-level version of EQ-5D (EQ-5D-5L). Qual Life Res 2011;20:1727-1736.

INCB054707 Investigator's Brochure. Wilmington, DE: Incyte Corporation.

Ingram JR. The epidemiology of hidradenitis suppurativa. Br J Dermatol 2020;183:990-998.

Jemec GB. Hidradenitis suppurativa. N Engl J Med 2012;366:158-164.

Kilgour JM, Li S, Sarin KY. Hidradenitis suppurativa in patients of color is associated with increased disease severity and healthcare utilization: A retrospective analysis of 2 U.S. cohorts. JAAD Int 2021;3:42-52.

Kimball AB, Naegeli AN, Edson-Heredia E, et al. Psychometric properties of the Itch Numeric Rating Scale in patients with moderate-to-severe plaque psoriasis. Br J Dermatol 2016a;175:157-162.

Kimball AB, Okun MM, Williams DA, et al. Two phase 3 trials of adalimumab for hidradenitis suppurativa. N Engl J Med 2016b;375:422-434.

Kimball AB, Sobell JM, Zouboulis CC, et al. HiSCR (Hidradenitis Suppurativa Clinical Response): a novel clinical endpoint to evaluate therapeutic outcomes in patients with hidradenitis suppurativa from the placebo-controlled portion of a phase 2 adalimumab study. J Eur Acad Dermatol Venereol 2016c;30:989-994.

Kirby JS, Thorlacius L, Villumsen B, et al. The Hidradenitis Suppurativa Quality of Life (HiSQOL) score: development and validation of a measure for clinical trials. Br J Dermatol 2020;183:340-348.

Lee DE, Clark AK, Shi VY. Hidradenitis suppurativa: disease burden and etiology in skin of color. Dermatology 2017;233:1-6.

Mantel N, Haenszel W. Statistical aspects of analysis of data from retrospective studies of disease. J Natl Cancer Inst 1959;22:719-748.

Matusiak L, Bieniek A, Szepietowski JC. Psychophysical aspects of hidradenitis suppurativa. Acta Derm Venereol 2010;90:264-268.

Matusiak Ł. Profound consequences of hidradenitis suppurativa: a review. Br J Dermatol 2020;183;e171-e177.

Millum J, Grady C. The ethics of placebo-controlled trials: methodological justifications. Contemp Clin Trials 2013;36:510-514.

National Institutes of Health. COVID-19 Treatment Guidelines. Coronavirus Disease 2019 (COVID-19) Treatment Guidelines. Accessed April 7, 2023. https://www.covid19treatmentguidelines.nih.gov

Nguyen TV, Damiani G, Orenstein LAV, Hamzavi I, Jemec GB. Hidradenitis suppurativa: an update on epidemiology, phenotypes, diagnosis, pathogenesis, comorbidities and quality of life. J Eur Acad Dermatol Venereol 2021;35:50-61.

Reilly MC, Zbrozek AS, Dukes EM. The validity and reproducibility of a work productivity and activity impairment instrument. Pharmacoeconomics 1993;4:353-365.

Ring HC, Maul JT, Yao Y, et al. Drug survival of biologics in patients with hidradenitis suppurativa. JAMA Dermatol 2022;158:184-188.

Rumberger BE, Boarder EL, Owens SL, Howell MD. Transcriptomic analysis of hidradenitis suppurativa skin suggests roles for multiple inflammatory pathways in disease pathogenesis. Inflamm Res 2020;69:967-973.

Sabat R, Jemec GBE, Matusiak Ł, Kimball AB, Prens E, Wolk K. Hidradenitis suppurativa. Nat Rev Dis Primers 2020;6:18.

Savage KT, Singh V, Patel ZS, et al. Pain management in hidradenitis suppurativa and a proposed treatment algorithm. J Am Acad Dermatol 2021;85:187-199.

Shao K, Hooper J, Feng H. Racial and ethnic health disparities in dermatology in the United States. Part 2: Disease-specific epidemiology, characteristics, management, and outcomes. J Am Acad Dermatol 2022;87:733-744.

Torres PA, Helmstetter JA, Kaye AM, Kaye AD. Rhabdomyolysis: pathogenesis, diagnosis, and treatment. Ochsner J 2015;15:58-69.

Van Buuren S. Multiple imputation of discrete and continuous data by fully conditional specification. Stat Methods Med Res 2007;16:219-242.

Van Straalen KR, Ingram JR, Augustin M, Zouboulis CC. New treatments and new assessment instruments for hidradenitis suppurativa. Exp Dermatol 2022a;31(suppl 1):33-39.

Van Straalen KR, Prens EP, Gudjonsson JE. Insights into hidradenitis suppurativa. J Allergy Clin Immunol 2022b;149:1150-1161.

Vossen ARJV, Schoenmakers A, van Straalen KR, Prens EP, van der Zee HH. Assessing pruritus in hidradenitis suppurativa: a cross-sectional study. Am J Clin Dermatol 2017;18:687-695.

Yao Y, Jørgensen AR, Thomsen SF. Work productivity and activity impairment in patients with hidradenitis suppurativa: a cross-sectional study. Int J Dermatol 2020;59:333-340.

Zouboulis CC, Benhadou F, Byrd AS, et al. What causes hidradenitis suppurativa? — 15 years after. Exp Dermatol 2020a;29:1154-1170.

Zouboulis CC, Frew JW, Giamarellos-Bourboulis EJ, et al. Target molecules for future hidradenitis suppurativa treatment. Exp Dermatol 2021;30(suppl 1):8-17.

Zouboulis CC, Gulliver W, Ingram J, et al. Endpoints of clinical trials for hidradenitis suppurativa: proceedings of a round-table session. Exp Dermatol 2020b;29(suppl 1):67-72.

Zouboulis CC, Okun MM, Jemec GBE, et al. Long-term adalimumab efficacy in patients with moderate-to-severe hidradenitis suppurativa/acne inversa: 3-year results of a phase 3 open-label extension study. J Am Acad Dermatol 2019;80:60-69.

Zouboulis CC, Tzellos T, Kyrgidis A, et al. Development and validation of the International Hidradenitis Suppurativa Severity Score System (IHS4), a novel dynamic scoring system to assess HS severity. Br J Dermatol 2017;177:1401-1409.

Zouboulis VA, Zouboulis KC, Zouboulis CC. Hidradenitis suppurativa and comorbid disorder biomarkers, druggable genes, new drugs and drug repurposing — a molecular meta-analysis. Pharmaceutics 2022;14:44.

APPENDIX A. INFORMATION REGARDING EFFECTIVENESS OF CONTRACEPTIVE METHODS AND DEFINITIONS

**Definitions**
WOCBP: A woman who is considered fertile following menarche and until becoming postmenopausal unless permanently sterile (see below).<br>Women in the following categories are not considered WOCBP:<br>• Premenarchal<br>• Premenopausal with 1 of the following:[a]<br>– Documented hysterectomy<br>– Documented bilateral salpingectomy<br>– Documented bilateral oophorectomy<br>• Postmenopausal is defined as:<br>– Participants with no menses for 12 or more months without an alternative medical cause AND a serum FSH level > 40 IU/L.<br>– Female participants on HRT and whose menopausal status is in doubt will be required to use 1 of the nonhormonal, highly effective contraception methods if they wish to continue their HRT during the study. Otherwise, they must discontinue HRT to allow confirmation of postmenopausal status before study enrollment.
**For male participants of reproductive potential**[b]
The following methods during the Protocol-defined timeframe in Section 5.1 are highly effective:<br>• Use of a male condom plus partner use of an additional contraceptive method when having penile-vaginal intercourse with a WOCBP who is not currently pregnant<br>• Vasectomy with medical assessment of the surgical success (verified by site personnel's review of the participant's medical records)<br>• Permanently sterile (verified by site personnel's review of the participant's medical records)<br>• Sexual abstinence[c]<br>– Be abstinent from penile-vaginal intercourse as their usual and preferred lifestyle (abstinent on a long-term and persistent basis) and agree to remain abstinent (sexual abstinence is not approved in Japan).<br>The following are **not** acceptable methods of contraception:<br>• Periodic abstinence (calendar, symptothermal, postovulation methods), withdrawal (coitus interruptus), spermicides only, and lactational amenorrhea method<br>• Male condom with cap, diaphragm, or sponge with spermicide<br>• Male and female condom used together<br>Note: Men with a pregnant or breastfeeding partner must agree to remain abstinent from penile-vaginal intercourse or use a male condom during each episode of penile penetration (sexual abstinence is not approved in Japan).

For female participants who are WOCBP
The following methods during the Protocol-defined timeframe in Section 5.1 that can achieve a failure rate of less than 1% per year when used consistently and correctly are considered as highly effective birth control methods:<br>• Combined (estrogen and progestogen containing) hormonal contraception associated with inhibition of ovulation[d]<br>– oral<br>– intravaginal (administration route is not approved in Japan)<br>– transdermal (administration route is not approved in Japan)<br>• Progestogen-only hormonal contraception associated with inhibition of ovulation[d] (progesterone-only hormonal contraception is not approved in Japan, so this bullet and its sub-bullets will not apply for Japan)<br>– oral<br>– injectable<br>– implantable[e]<br>• Intrauterine device[e]<br>• Intrauterine hormone-releasing system[e]<br>• Bilateral tubal occlusion[e]<br>• Vasectomized or permanently sterile partner[e,f]<br>• Sexual abstinence[c] (sexual abstinence is not approved in Japan)

[a] Documentation can come from the site personnel's review of the participant's medical records, medical examination, or medical history interview.
[b] If the male participant has a partner of childbearing potential, the partner should also use contraceptives.
[c] In the context of this guidance, sexual abstinence is considered a highly effective method only if defined as refraining from heterosexual intercourse during the entire period of risk associated with the study treatment. The reliability of sexual abstinence needs to be evaluated in relation to the duration of the clinical study and the preferred and usual lifestyle of the participant.
[d] It is unknown if hormonal contraception may be susceptible to interaction with the investigational medicinal product, which may reduce the efficacy of the contraception method. Therefore, 2 methods of contraception should be used.
[e] Contraception methods that in the context of this guidance are considered to have low user dependency.
[f] Vasectomized or permanently sterile partner is a highly effective method of avoiding pregnancy provided that partner is the sole sexual partner of the WOCBP study participant and that the partner has received medical assessment of sterility.

Source: Clinical Trials Facilitation and Coordination Group 2020.

APPENDIX B. LIST OF STRONG SYSTEMIC CYP3A4 INHIBITORS AND STRONG/MODERATE SYSTEMIC CYP3A4 INDUCERS

Strong Systemic CYP3A4 Inhibitors	Strong and Moderate Systemic CYP3A4 Inducers
• Ceritinib<br>• Clarithromycin<br>• Cobicistat<br>• Danoprevir<br>• Dasabuvir<br>• Elvitegravir<br>• Idelalisib<br>• Indinavir<br>• Itraconazole<br>• Ketoconazole<br>• Lopinavir<br>• Nefazodone<br>• Nelfinavir<br>• Ombitasvir<br>• Paritaprevir<br>• Posaconazole<br>• Ritonavir[a]<br>• Saquinavir<br>• Telithromycin<br>• Tipranavir<br>• Troleandomycin<br>• Voriconazole<br><br>• Grapefruit/grapefruit juice<br>• Seville oranges	• Apalutamide<br>• Bosentan<br>• Carbamazepine<br>• Cenobamate<br>• Dabrafenib<br>• Efavirenz<br>• Enzalutamide<br>• Etravirine<br>• Ivosidenib<br>• Lorlatinib<br>• Lumacaftor<br>• Mitotane<br>• Pexidartinib<br>• Phenobarbital<br>• Phenytoin<br>• Primidone<br>• Rifampicin/rifampin<br>• Rifapentine<br>• Sotorasib<br><br>• St John's wort

Note: Updated lists of CYP3A4 inhibitors/inducers can be found at Food and Drug Administration. Drug Development and Drug Interactions: Table of Substrates, Inhibitors and Inducers. Available at: https://www fda.gov/drugs/drug-interactions-labeling/drug-development-and-drug-interactions-table-substrates-inhibitors-and-inducers.

[a] Paxlovid™ is a coadministration of nirmatrelvir and ritonavir.

CCI

[redacted]

[redacted] C

CCI

[redacted]

CCI

[redacted]

CCI

[redacted]

CCI

[redacted]

CCI

[redacted]

CCI

[redacted]

CCI

[redacted]

APPENDIX F. PROTOCOL AMENDMENT SUMMARY OF CHANGES

Document	Date
Amendment 1	10 APR 2023
Amendment 2	01 MAY 2023

Amendment 2 (01 MAY 2023)

Overall Rationale for the Amendment:

The primary purpose of the amendment is to address regulatory requests provided after review by the EU national health authorities. This amendment is applicable to the following countries: Austria, Belgium, Czechia, France, Germany, Greece, Netherlands, Poland, and Spain.

1. **Section 1, Protocol Summary; Section 4.1, Overall Design; Section 4.2, Overall Study Duration; Section 6.7, Treatment After the End of the Study; Section 8.8.1, Safety Follow-up**

   Description of change: Clarified aspects related to poststudy access to study drug.

   Rationale for change: Regulatory request.

2. **Section 2.4, Benefit/Risk Assessment (Table 7: Risk-Mitigation Assessment for Povorcitinib)**

   Description of change: Included statement regarding the benefits and risks of participating in this study for potential participants who are current or past long-time smokers.

   Rationale for change: Regulatory request.

3. **Section 6.5.1, Criteria and Procedures for Dose Interruption and Discontinuation of Study Drug (Table 11: Guidelines for Interruption, Restarting, and Discontinuation of Study Drug)**

   Description of change: Revised guidance to describe management of CPK elevations.

   Rationale for change: Regulatory request.

4. **Section 8.1.1, Informed Consent Process**

   Description of change: Included statement explaining that the ICF will describe the potential risks associated with participation in this study and treatment with the investigational product for participants who are current or past long-time smokers.

   Rationale for change: Regulatory request.

5. **Incorporation of administrative changes.** Other administrative changes have been incorporated throughout the Protocol and are noted in the redline version of the amendment.

Amendment 1 (10 APR 2023)

Overall Rationale for the Amendment:

The primary purpose of the amendment is to address regulatory requests provided after review by the EU national health authorities. This amendment is applicable to the following countries: Austria, Belgium, Czechia, France, Germany, Greece, Netherlands, Poland, and Spain.

1. **Title Page**

   Description of change: Revised the binding statement that the clinical trial will be conducted in compliance with Clinical Trials Regulation (EU) No. 536/2014.

   Rationale for change: Regulatory request.

2. **Section 2.4, Benefit/Risk Assessment (Table 7: Risk-Mitigation Assessment for Povorcitinib); Section 5.2, Exclusion Criteria (Exclusion Criterion 3g)**

   Description of change: Removed "or cervical carcinoma in situ."

   Rationale for change: Regulatory request.

3. **Section 6.5.1, Criteria and Procedures for Dose Interruption and Discontinuation of Study Drug (Table 11: Guidelines for Interruption, Restarting, and Discontinuation of Study Drug)**

   Description of change: Revised guidance to describe management of ALT/AST elevations.

   Rationale for change: Regulatory request.

4. **Section 6.5.1.3, Management of COVID-19**

   Description of change: Revised guidance regarding COVID-19 management.

   Rationale for change: Regulatory request.

5. **Section 6.5.1.5, Management of Malignancy**

   Description of change: Revised to include any malignancy.

   Rationale for change: Regulatory request.

6. **Section 6.6.10, Systemic Antibiotic Rescue Therapy**

   Description of change: Clarified rationale on why rifampin/rifampicin is prohibited for systemic antibiotic rescue therapy.

   Rationale for change: Regulatory request.

7. **Section 6.7, Treatment After the End of the Study**

   Description of change: Clarified aspects related to poststudy access to study drug.

   Rationale for change: Regulatory request.

8. **Section 9.4, Reporting of Serious Adverse Events**

   Description of change: Updated directive reference.

   Rationale for change: Regulatory request.

9. **Incorporation of administrative changes.** Other administrative changes have been incorporated throughout the Protocol and are noted in the redline version of the amendment.

Approval Task	PPD<br>Document Preparer<br>PPD<br>01-May-2023 19:45:25 GMT+0000

Approval Task	PPD<br>Approver<br>PPD<br>01-May-2023 19:47:24 GMT+0000

Approval Task	PPD<br><br>PPD<br>01-May-2023 19:52:47 GMT+0000

Approval Task	PPD<br>Approver<br>PPD<br>01-May-2023 20:08:36 GMT+0000

Approval Task	PPD<br>Approver<br>PPD<br>01-May-2023 20:14:38 GMT+0000

Clinical Study Protocol

INCB 54707-302

Selective **T**reatment of **O**ral **P**ovorcitinib in **H**idradenitis **S**uppurativa Study **2** (**STOP-HS2**)

A Phase 3, Double-Blind, Randomized, Placebo-Controlled, Efficacy and Safety Study of Povorcitinib (INCB054707) in Participants With Moderate to Severe Hidradenitis Suppurativa

**Product:**	**Povorcitinib**
**IND Number:**	[redacted]
**EU CT Number:**	**2022-501753-36-00**
**Phase of Study:**	**3**
**Sponsor:**	**Incyte Corporation**<br>**1801 Augustine Cut-Off**<br>**Wilmington, DE 19803 USA**
**Original Protocol:**	**24 AUG 2022**
**Amendment 1:**	**10 APR 2023**
**Amendment 2:**	**01 MAY 2023**

This study will be performed in accordance with ethical principles that have their origin in the Declaration of Helsinki (Brazil 2013) and conducted in adherence to the study Protocol, applicable Good Clinical Practices, and applicable laws and country-specific regulations, including WMO (Medical Research Involving Human Participants Act) and Clinical Trials Regulation (EU) No. 536/2014, in which the study is being conducted.

The information in this document is confidential. No part of this information may be duplicated, referenced, or transmitted in any form or by any means (electronic, mechanical, photocopy, recording, or otherwise) without prior written consent.

INVESTIGATOR'S AGREEMENT

I have read the INCB 54707-302 Protocol Amendment 2 (dated 01 MAY 2023) and agree to conduct the study as outlined. I agree to maintain the confidentiality of all information received or developed in connection with this Protocol.

____

(Printed Name of Investigator)

____________________________________ ____________________________________

(Signature of Investigator) (Date)

TABLE OF CONTENTS

TITLE PAGE 1
INVESTIGATOR'S AGREEMENT 2
TABLE OF CONTENTS 3
LIST OF ABBREVIATIONS 9
1. PROTOCOL SUMMARY 13
2. INTRODUCTION 19
2.1. Background 19
2.2. Povorcitinib 20
[redacted]
2.2.2. Clinical Experience 21
2.2.2.1. Clinical Pharmacokinetics in Phase 1 Studies 21
2.2.2.2. Hidradenitis Suppurativa 22
2.3. Study Rationale 27
2.3.1. Scientific Rationale for Study Design 27
[redacted]
2.4. Benefit/Risk Assessment 30
3. OBJECTIVES AND ENDPOINTS 33
4. STUDY DESIGN 38
4.1. Overall Design 38
4.2. Overall Study Duration 39
4.3. Study Termination 39
5. STUDY POPULATION 40
5.1. Inclusion Criteria 40
5.2. Exclusion Criteria 41
5.3. Lifestyle Considerations 45
5.3.1. Contraception 45
5.3.1.1. Female Participants 45
5.3.1.2. Male Participants 45
5.3.2. Meals and Dietary Restrictions 45
5.3.3. Prohibited Therapies During the Placebo-Controlled Period 46

5.4. Screen Failures 46
5.5. Replacement of Participants 46
5.6. Data Safety Monitoring Board 46
5.7. Cardiovascular Adjudication Committee 47
6. STUDY TREATMENT 47
6.1. Study Treatment Administered 47
6.2. Preparation, Handling, and Accountability 48
6.3. Measures to Minimize Bias: Randomization and Blinding 48
6.4. Study Treatment Compliance 49
[redacted]
6.5.1. Criteria and Procedures for Dose Interruption and Discontinuation of Study Drug 50
[redacted]
6.6. Concomitant Medications and Procedures 54
6.6.1. Prior HS Therapy 54
[redacted]
6.6.6. Permitted Medications and Procedures 58
[redacted]
6.6.6.2. Wound Care Dressings 58
6.6.6.3. COVID-19 Vaccination 59
[redacted]
6.6.9. Lesion Rescue 61

6.6.10. Systemic Antibiotic Rescue Therapy....62
6.7. Treatment After the End of the Study....63
7. DISCONTINUATION OF STUDY TREATMENT AND PARTICIPANT WITHDRAWAL....63
7.1. Discontinuation of Study Treatment....63
7.1.1. Reasons for Discontinuation....63
7.1.2. Discontinuation Procedures....64
7.2. Participant Withdrawal From the Study....64
7.3. Lost to Follow-Up....65
8. STUDY ASSESSMENTS AND PROCEDURES....65
8.1. Administrative and General Procedures....65
8.1.1. Informed Consent Process....65
8.1.2. Screening Procedures....66
8.1.3. Interactive Response Technology Procedure....66
8.1.4. Distribution of Participant Card....67
8.1.5. Demography and Medical History....67
8.1.5.1. Demographics and Medical History....67
8.1.5.2. Disease Characteristics and Treatment History....68
[redacted]
8.2. Efficacy Assessments....69
8.2.1. HS Lesion Assessment and Count....69
8.2.1.1. Hidradenitis Suppurativa Clinical Response....72
8.2.1.2. Change in HS Lesion Counts....72
8.2.1.3. International Hidradenitis Suppurativa Severity Score System....72
8.2.1.4. Flare Incidence....73
8.2.1.5. Lesion Rescue....73
8.2.2. Hurley Stage....73
8.2.3. Photography....74
8.2.4. Patient-Reported Outcomes....74
8.2.4.1. Daily Diary Patient-Reported Outcomes....74
8.2.4.2. Patient-Reported Outcomes Completed at Study Visits....75
[redacted]
8.3. Safety Assessments....77

8.3.1. Adverse Events 77
8.3.2. Physical Examinations 78
8.3.3. Vital Signs 78
8.3.4. Electrocardiograms 79
8.3.5. Laboratory Assessments 79
8.3.5.1. Pregnancy Testing 81
8.3.5.2. Tuberculosis Assessment 82
8.3.5.3. HIV Testing 83
8.3.5.4. Hepatitis Testing 84
[redacted]
8.6. Unscheduled Visits 85
8.7. End of Treatment and Early Termination 85
8.8. Follow-Up 86
8.8.1. Safety Follow-Up 86
9. ADVERSE EVENTS: DEFINITIONS AND PROCEDURES FOR RECORDING, EVALUATING, FOLLOW-UP, AND REPORTING 87
9.1. Definition of Adverse Event 87
9.2. Definition of Serious Adverse Event 88
9.3. Recording and Follow-Up of Adverse Events and/or Serious Adverse Events 89
9.4. Reporting of Serious Adverse Events 91
9.5. Potential Drug-Induced Liver Injury 93
[redacted]
9.7. Emergency Unblinding of Treatment Assignment 93
9.8. Pregnancy 94
9.9. Warnings and Precautions 94
9.10. Product Complaints 95
9.11. Treatment of Overdose 95
10. STATISTICS 96
10.1. Sample Size Determination 96
10.2. Populations for Analysis 97
10.3. Level of Significance 97

10.4. Statistical Analyses 99
10.4.1. Primary Analysis 100
10.4.1.1. Primary Estimand 100
10.4.1.2. Sensitivity Analysis 101
10.4.1.3. Subgroup Analyses 101
10.4.2. Secondary Analyses 102
10.4.2.1. Key Secondary Estimands 102
10.4.2.2. Other Secondary and Exploratory Analyses 104
10.4.3. Safety Analyses 105
[redacted]
10.5. Interim Analysis 106
11. SUPPORTING DOCUMENTATION AND OPERATIONAL CONSIDERATIONS 106
11.1. Investigator Responsibilities 106
11.1.1. Identification of the Coordinating Principal Investigator 107
11.2. Data Management 107
11.3. Data Quality Assurance 110
11.4. Data Privacy and Confidentiality of Study Records 110
11.5. Financial Disclosure 111
11.6. Publication Policy 111
11.7. Study and Site Closure 112
12. REFERENCES 113
APPENDIX A. INFORMATION REGARDING EFFECTIVENESS OF CONTRACEPTIVE METHODS AND DEFINITIONS 117
APPENDIX B. LIST OF STRONG SYSTEMIC CYP3A4 INHIBITORS AND STRONG/MODERATE SYSTEMIC CYP3A4 INDUCERS 119
[redacted]

APPENDIX F. PROTOCOL AMENDMENT SUMMARY OF CHANGES 128

LIST OF TABLES

Table 1: Primary and Key Secondary Objectives and Endpoints 13
Table 2: Key Study Design Elements 14
Table 3: Schedule of Activities 16
Table 4: [redacted]
Table 5: INCB 54707-204 Summary of TEAEs (Individual TEAEs With a Cutoff ≥ 2.0% Based on Total Povorcitinib Values) 26
Table 6: Summary of Serious Treatment-Emergent Adverse Events During the Placebo-Controlled Period (ITT Population)...27
Table 7: [redacted]
Table 8: Objectives and Endpoints 33
Table 9: Exclusionary Laboratory Values 44
Table 10: Study Treatment Information 47
Table 11: [redacted]
Table 12: Criteria for Systemic Antibiotic Rescue Therapy..63
Table 13: HS Lesion Definition..70
Table 14: Hurley Stages of Hidradenitis Suppurativa 74
[redacted]
Table 16: Interpretation and Management of HBV Serologic Test Results 84
Table 17: Powers for Primary and Key Secondary Endpoints 96
Table 18: Populations for Analysis...97
Table 19: Primary Estimand 100
Table 20: Key Secondary Estimands 102

LIST OF FIGURES

Figure 1: Study Design Schema 15
Figure 2: Proportion of HiSCR Responders During the Placebo-Controlled Period.................24
Figure 3: Mean (SE) Change From Baseline in Skin Pain NRS During the Placebo-Controlled Period...24
Figure 4: Graphic Approach for Multiplicity Adjustment for Testing Primary and Key Secondary Endpoints 99

LIST OF ABBREVIATIONS

Abbreviations and Special Terms	Definition
AE	adverse event
AESI	adverse event of special interest
ALP	alkaline phosphatase
ALT	alanine aminotransferase
AN	abscess and inflammatory nodule
ANC	absolute neutrophil count
ANdT	abscess, inflammatory nodule, and draining tunnel (formerly known as draining fistula)
aPTT	activated partial thromboplastin time
AST	aspartate aminotransferase
BCG	Bacille Calmette-Guérin
CABG	coronary artery bypass graft
CAC	cardiovascular adjudication committee
$C_{avg,ss}$	average concentration of study drug at steady state
CI	confidence interval
C_{max}	maximum observed plasma or serum concentration
$C_{max,ss}$	maximum concentration of study drug at steady state
CMH	Cochran-Mantel-Haenszel
COVID-19	coronavirus disease 2019
CPK	creatine phosphokinase
CRO	contract research organization
CSR	Clinical Study Report
CT	computed tomography
$C_{tau,ss}$	trough concentration of study drug at steady state
CTCAE	Common Terminology Criteria for Adverse Events
CXR	chest x-ray
CYP3A4	cytochrome P450 3A4 enzyme
DILI	drug-induced liver injury
DLQI	Dermatology Life Quality Index
DNA	deoxyribonucleic acid
DSMB	Data Safety Monitoring Board
ECG	electrocardiogram
ECI	event of clinical interest
eCOA	electronic clinical outcome assessment

Abbreviations and Special Terms	Definition
eCRF	electronic case report form
EDC	electronic data capture
eDiary	electronic diary
EMA	European Medicines Agency
EOS	end of study
EOT	end of treatment
EOT1	end of treatment during the placebo-controlled period
EOT2	end of treatment during the open-label extension period
E-R	exposure-response
ET	early termination
[redacted]	[redacted]
[redacted]	[redacted]
ET1	early termination during the placebo-controlled period
ET2	early termination during the open-label extension period
EXT	extension
FACIT-F	Functional Assessment of Chronic Illness Therapy – Fatigue
FDA	Food and Drug Administration
FSH	follicle-stimulating hormone
GCP	Good Clinical Practice
GDPR	General Data Protection Regulation
GGT	gamma-glutamyl transferase
GM-CSF	granulocyte-macrophage colony-stimulating factor
HBcAb	hepatitis B core antibody
HBsAb	hepatitis B surface antibody
HBsAg	hepatitis B surface antigen
HBV	hepatitis B virus
HCV	hepatitis C virus
HCVAb	hepatitis C virus antibody
HDL	high-density lipoprotein
HIPAA	Health Insurance Portability and Accountability Act
HiSCR	Hidradenitis Suppurativa Clinical Response
HiSQoL	Hidradenitis Suppurativa Quality of Life
HIV	human immunodeficiency virus
HRT	hormone replacement therapy
HS	hidradenitis suppurativa

Abbreviations and Special Terms	Definition
hsCRP	high-sensitivity C-reactive protein
IB	Investigator's Brochure
IC_{50}	concentration that results in 50% inhibition
ICF	informed consent form
ID	identification
IEC	independent ethics committee
IGRA	interferon gamma release assay
IHS4	International Hidradenitis Suppurativa Severity Score System
IL	interleukin
INR	international normalized ratio
IRAK	interleukin-1 receptor–associated kinase
IRB	institutional review board
IRT	interactive response technology
ITT	intent-to-treat
JAK	Janus kinase
LDL	low-density lipoprotein
LTE	long-term extension
MACE	major adverse cardiovascular event
MCP-1	monocyte chemoattractant protein 1
MDRD	Modification of Diet in Renal Disease
MedDRA	Medical Dictionary for Regulatory Activities
MMRM	mixed model for repeated measures
MRI	magnetic resonance imaging
NIH	National Institutes of Health
NRI	nonresponder imputation
NRS	Numeric Rating Scale
NSAID	nonsteroidal anti-inflammatory drug
NYHA	New York Heart Association
OLE	open-label extension
PC	placebo-controlled
PCR	polymerase chain reaction
PD	pharmacodynamic(s)
PDE	phosphodiesterase
PK	pharmacokinetic(s)
PMDA	Pharmaceuticals and Medical Devices Agency

Abbreviations and Special Terms	Definition
PP	per protocol
PPD	purified protein derivative
PPI	proton pump inhibitor
PRO	patient-reported outcome
PT	prothrombin time
QALY	quality-adjusted life year
QD	once daily
QoL	quality of life
RNA	ribonucleic acid
RSI	Reference Safety Information
SAE	serious adverse event
SAP	Statistical Analysis Plan
SARS-CoV-2	severe acute respiratory syndrome coronavirus 2
SE	standard error
SoA	schedule of activities
SOP	standard operating procedure
SpO_2	oxygen saturation measured by pulse oximetry
STAT	signal transducer and activator of transcription
STD	standard deviation
$t_{½}$	half-life of study drug
TB	tuberculosis
TEAE	treatment-emergent adverse event
Th	T-helper
t_{max}	time to maximum concentration
$t_{max,ss}$	time to achieve maximum concentration of study drug at steady state
TNF	tumor necrosis factor
TYK2	tyrosine kinase 2
ULN	upper limit of normal
VAS	visual analog scale
WBC	white blood cell
WOCBP	women of childbearing potential
WONCBP	women of nonchildbearing potential
WPAI	Work Productivity and Activity Impairment

1. PROTOCOL SUMMARY

Protocol Title:

Selective **T**reatment of **O**ral **P**ovorcitinib in **H**idradenitis **S**uppurativa Study **2** (**STOP-HS2**)

A Phase 3, Double-Blind, Randomized, Placebo-Controlled, Efficacy and Safety Study of Povorcitinib (INCB054707) in Participants With Moderate to Severe Hidradenitis Suppurativa

Protocol Number: INCB 54707-302

Objectives and Endpoints:

Table 1 presents the primary and key secondary objectives and endpoints.

Table 1: Primary and Key Secondary Objectives and Endpoints

Objectives	Endpoints
**Primary**	
To evaluate the efficacy of povorcitinib.	• Proportion of participants who achieve Hidradenitis Suppurativa Clinical Response (HiSCR) at Week 12.<br>− HiSCR is defined as at least a 50% reduction from baseline in the total abscess and inflammatory nodule count, with no increase from baseline in abscess or draining tunnel count.
**Key Secondary**	
To further evaluate the efficacy of povorcitinib.	• Proportion of participants who achieve Hidradenitis Suppurativa Clinical Response 75 (HiSCR75) at Week 12.<br>− HiSCR75 is defined as at least a 75% reduction from baseline in the total abscess and inflammatory nodule count, with no increase from baseline in abscess or draining tunnel count.
To evaluate the effect of povorcitinib in reducing the incidence of flares.	• Proportion of participants with flare by Week 12.<br>− Participants who experience at least 1 flare over 12 weeks; flare is defined as at least a 25% increase in the total abscess and inflammatory nodule count with a minimum increase of 2 relative to baseline.
To evaluate the effect of povorcitinib in decreasing skin pain.	• Key secondary endpoint for regulatory purposes in US/FDA/aligned countries:<br>Proportion of participants with a ≥ 3-point decrease in Skin Pain Numeric Rating Scale (NRS) score at Week 12 among participants with baseline Skin Pain NRS score ≥ 3.<br>− Participants with a Skin Pain score of at least 3 at baseline and who experience at least a 3-point decrease in Skin Pain score at Week 12, relative to baseline. Skin Pain is an 11-point NRS, ranging from 0 (no skin pain) to 10 (worst skin pain).<br>• Key secondary endpoint for regulatory purposes in EU/EMA/aligned countries:<br>Proportion of participants who achieve Skin Pain NRS30 at Week 12 among participants with baseline Skin Pain NRS score ≥ 3.<br>− Participants with a Skin Pain score of at least 3 at baseline and who achieve at Week 12 Skin Pain NRS30, defined as at least a 30% reduction and at least 1-unit reduction from baseline in the Skin Pain NRS.

Overall Design:

Table 2 presents the key study design elements. Further study details are presented after the table.

Table 2: Key Study Design Elements

**Study Phase**	Phase 3
**Clinical Indication**	Treatment of patients 18 years and older with moderate to severe HS who have a history of inadequate response, intolerance, or contraindication to an adequate course of conventional systemic therapy for HS.
**Population**	Men or women ≥ 18 years of age who have had a diagnosis of moderate to severe HS (Hurley Stage II or III) for at least 3 months and who have a documented history of inadequate response to at least a 3-month course of at least 1 conventional systemic therapy (oral antibiotic or biologic drug) for HS (or demonstrated intolerance to, or have a contraindication to, a conventional systemic therapy for treatment of their HS), with HS lesions in at least 2 distinct anatomical areas and total abscess and inflammatory nodule count of at least 5.
**Number of Participants**	Approximately 600 participants will be randomized 1:1:1 to povorcitinib (45 or 75 mg) or placebo QD.
**Study Design**	This is a Phase 3, multicenter, randomized, parallel-group, PC, double-blind study to evaluate the efficacy and safety of povorcitinib (45 or 75 mg QD) over a 12-week period, followed by a 42-week double-blind EXT period (povorcitinib 45 or 75 mg QD).<br>Participants will be stratified based on ANdT count (< 11 or ≥ 11) at baseline and previous treatment of HS with a biologic drug.
**Estimated Duration of Study Participation**	Screening: up to 28 days<br>PC period (double-blind): 12 weeks<br>EXT period (double-blind): 42 weeks<br>Safety follow-up: 30 days after last dose of study drug<br>Total: up to approximately 62 weeks (≈14 months)
**DSMB**	Yes (external)
**Cardiovascular Adjudication Committee**	Yes (external)
**Coordinating Principal Investigator**	Joslyn Sciacca Kirby, MD, MS, MEd

Treatment Groups and Duration:

Approximately 600 adult participants with moderate to severe HS (Hurley Stage II or III) for at least 3 months, HS lesions in ≥ 2 distinct anatomical areas, total abscess and inflammatory nodule count ≥ 5, and a history of inadequate response to at least a 3-month course of at least 1 conventional systemic therapy (oral antibiotic or biologic drug) for HS (or demonstrated intolerance to, or have a contraindication to, a conventional systemic therapy for treatment of their HS) will be randomized 1:1:1 to povorcitinib (45 mg or 75 mg) or placebo (see Figure 1). During the double-blind, PC period, participants will be treated with orally administered povorcitinib (45 or 75 mg) or placebo QD for 12 weeks. At Week 12, participants will enter the double-blind EXT period, which will last for 42 weeks. During the EXT period, participants randomized to povorcitinib at baseline will remain at their respective dose level (45 or 75 mg QD), while participants randomized to placebo will receive povorcitinib 45 or 75 mg QD (1:1 ratio). Further information on the study design is presented in Section 4.1.

Table 3 presents the SoA, including the PC and EXT periods. Adherence to the study design requirements, including those specified in the SoA, is essential and required for study conduct.

The COVID-19 global pandemic may present challenges to the normal conduct of this study (including AE and laboratory assessments), requiring the outline of potential mitigation strategies described in Appendix E.

Participants who complete the 54-week treatment in this study may be eligible to participate in a separate extension study to further evaluate the safety and efficacy of the investigational product.

Figure 1: Study Design Schema

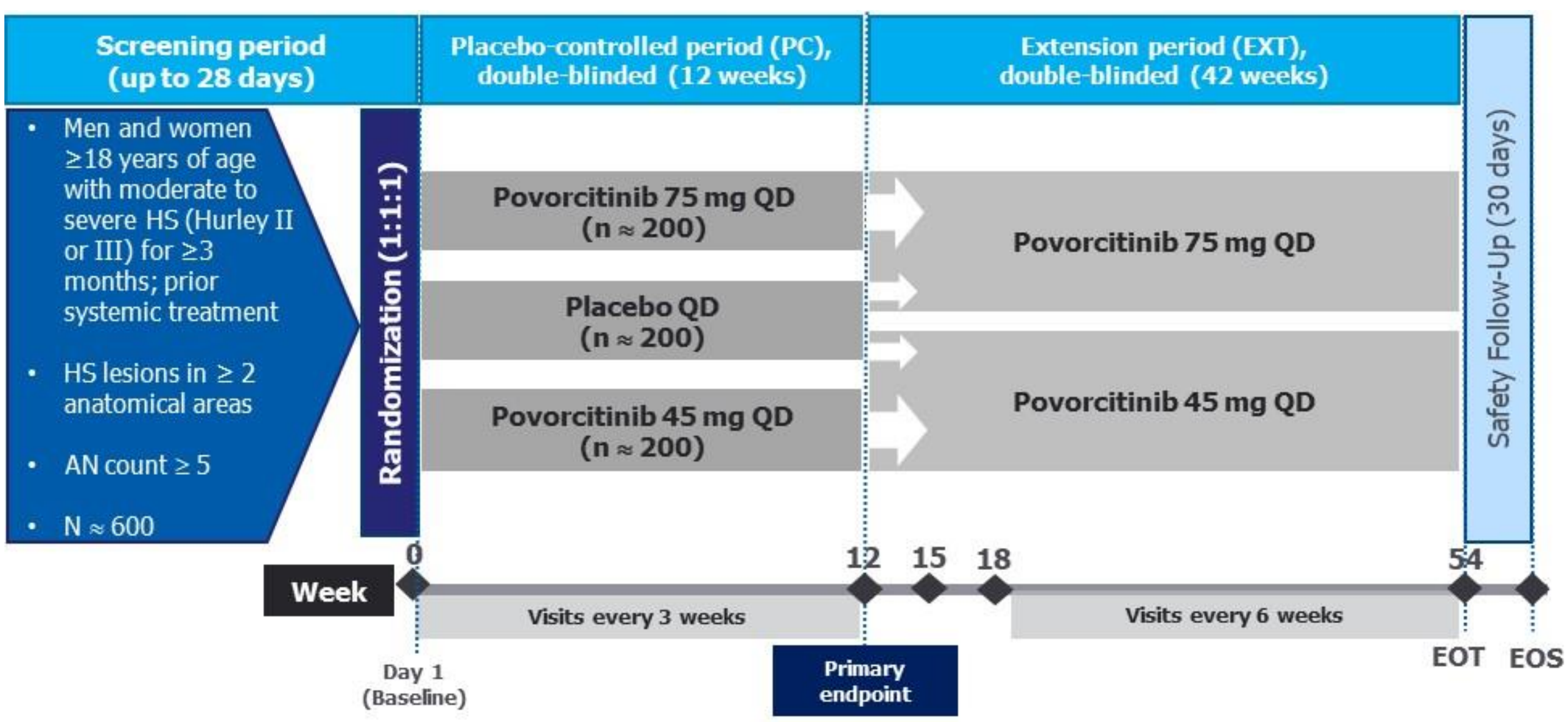

Table 3: Schedule of Activities

	Screening	PC Period (12 Weeks)					EXT Period (42 Weeks)						Safety Follow-Up	
**Visit**	**Days −28 to −1**	**Baseline (Day 1)**	**Wk 3 (± 3 d)**	**Wk 6 (± 3 d)**	**Wk 9 (± 3 d)**	**Wk 12 (± 3 d) EOT1 (or ET1)**	**Wk 15 (± 3 d)**	**Wk 18 (± 3 d)**	**Wk 24 (± 5 d)**	**Wk 30, Wk 36 (± 5 d)**	**Wk 42, Wk 48 (± 5 d)**	**Wk 54 (± 5 d) EOT2 (or ET2)**	**30 d (+ 7 d) After EOT or ET (EOS)**	**Section**
**Administrative procedures**														
Informed consent	X													8.1.1
Inclusion/exclusion criteria	X	X												5.1/5.2
Demographic data, medical history, and HS medical and treatment history	X													8.1.5
Prior/concomitant medications	X	X	X	X	X	X	X	X	X	X	X	X	X	6.6
Contact IRT	X	X	X	X	X	X	X	X	X	X	X	X		8.1.3
Randomization		X												8.1.2
Dispense study drug		X	X	X	X	X	X	X	X	X	X			6.1
Distribute trial participant card	X													8.1.4
Collect study drug and review drug compliance			X	X	X	X	X	X	X	X	X	X		6.4
Dispense eDiary (handheld device) and instruct participant on use	X													8.2.4
Collect eDiary (handheld device) from participant												X		8.2.4
Follow-up phone call for daily diary compliance (1 week before baseline visit and 1 week before Week 12 visit)	X					X								8.2.4.1.1.1
Study drug administration diary		Study drug administration diary to be completed daily by participant (baseline through Week 54)												6.1
Review diary compliance		X	X	X	X	X	X	X	X	X	X	X		8.2.4.1.1.2
[redacted]														

Table 3: Schedule of Activities (Continued)

Visit	Screening<br>Days −28 to −1	PC Period (12 Weeks)<br>Baseline (Day 1)	Wk 3 (± 3 d)	Wk 6 (± 3 d)	Wk 9 (± 3 d)	Wk 12 (± 3 d) EOT1 (or ET1)	EXT Period (42 Weeks)<br>Wk 15 (± 3 d)	Wk 18 (± 3 d)	Wk 24 (± 5 d)	Wk 30, Wk 36 (± 5 d)	Wk 42, Wk 48 (± 5 d)	Wk 54 (± 5 d) EOT2 (or ET2)	Safety Follow-Up<br>30 d (+ 7 d) After EOT or ET (EOS)	Section
**Safety assessments**														
AE assessments<br>(If an AE is noted, relevant body systems should be physically examined)	X	X	X	X	X	X	X	X	X	X	X	X	X	8.3.1
Contraception check	X	X	X	X	X	X	X	X	X	X	X	X	X	5.3.1
Comprehensive physical examination	X	X				X						X		8.3.2
Height	X													8.3.2
Weight	X	X				X						X		8.3.2
Vital signs	X	X	X	X	X	X	X	X	X	X	X	X	X	8.3.3
12-lead ECG	X					X						X		8.3.4
**Efficacy assessments**														
Lesion assessment and count	X	X	X	X	X	X	X	X	X	X	X	X	X	8.2.1
Hurley stage assessment	X	X				X			X			X		8.2.2
Photography (subset of sites/participants)		X				X			X					8.2.3
**QoL assessments – daily diaries**														
Skin Pain NRS	PRO daily diary (screening through Week 54)													8.2.4.1.2
Analgesic use	PRO daily diary (screening through Week 54)													8.2.4.1.3
Itch NRS	PRO daily diary (screening through Week 54)													8.2.4.1.4
**QoL assessments and health economics (conducted at study site before any other assessments)**														
HiSQoL		X	X	X	X	X	X	X	X	X	X	X	X	8.2.4.2.1
DLQI		X	X	X	X	X	X	X	X	X	X	X	X	8.2.4.2.2
FACIT-F		X	X	X	X	X	X	X	X	X	X	X	X	8.2.4.2.3
WPAI-HS		X		X		X		X	X			X	X	8.2.4.2.4

[redacted]

Table 3: Schedule of Activities (Continued)

	Screening	PC Period (12 Weeks)					EXT Period (42 Weeks)						Safety Follow-Up	
**Visit**	**Days −28 to −1**	**Baseline (Day 1)**	**Wk 3 (± 3 d)**	**Wk 6 (± 3 d)**	**Wk 9 (± 3 d)**	**Wk 12 (± 3 d) EOT1 (or ET1)**	**Wk 15 (± 3 d)**	**Wk 18 (± 3 d)**	**Wk 24 (± 5 d)**	**Wk 30, Wk 36 (± 5 d)**	**Wk 42, Wk 48 (± 5 d)**	**Wk 54 (± 5 d) EOT2 (or ET2)**	**30 d (+ 7 d) After EOT or ET (EOS)**	**Section**
**Laboratory assessments**														
Serum FSH (WONCBP only)	X													8.3.5.1.1
Serum pregnancy test (WOCBP only)	X												X	8.3.5.1.2
Urine pregnancy test (WOCBP only; conducted at study site)		X	X	X	X	X	X	X	X	X	X	X		8.3.5.1.2
TB assessment	X													8.3.5.2
HIV testing	X													8.3.5.3
Hepatitis testing	X													8.3.5.4
HBV DNA (for required participants)	X					X			X	X (Wk 36)	X (Wk 48)	X		8.3.5.4.1
Chemistry and hematology	X	X	X	X	X	X	X	X	X	X	X	X	X	8.3.5
Coagulation panel	X	X				X						X	X	8.3.5
Urinalysis	X	X				X			X			X	X	8.3.5
Nicotine metabolite screen (urine)		X										X		8.3.5
Lipid panel (fasting required)		X		X		X		X	X			X	X	8.3.5
hsCRP		X	X	X	X	X	X	X	X	X	X	X	X	8.3.5

[redacted]

2. INTRODUCTION

Povorcitinib (also known as INCB054707) is a novel, potent, oral small-molecule JAK1 inhibitor under evaluation in HS, vitiligo, and prurigo nodularis clinical trials. Janus kinase signaling regulates several proinflammatory pathways and has been well recognized as a key driver for numerous skin diseases. JAK inhibitors have been approved in various inflammatory disorders such as rheumatoid arthritis, atopic dermatitis, and alopecia areata. JAK-mediated proinflammatory cytokines persist in skin lesions of patients with HS, and results from a recent Phase 2b study with povorcitinib, demonstrating a dose-dependent efficacy in HS, support further investigation of this molecule in Phase 3 studies.

2.1. Background

Hidradenitis suppurativa, also designated as acne inversa, is a chronic inflammatory skin condition characterized by painful nodules and abscesses in early stages and draining tunnels (fistulas) and irreversible tissue damage and scarring in more severe disease (van Straalen et al 2022b). Cutaneous changes seem to start around hair follicles and involve activation of cells of the innate and adaptive immune systems, with pivotal roles for proinflammatory cytokines such as TNF, IL-1β, and IL-17 (Frew et al 2019, Zouboulis et al 2022). A genetic predisposition, smoking, obesity, and hormonal factors are established etiologic factors for HS (Zouboulis et al 2020a).

Typically starting in early adulthood and with a median delay of diagnosis of approximately 7 years, HS affects the intertriginous skin (eg, axillary, inguinal, gluteal and perianal body sites) and is associated with numerous systemic comorbidities, such as Crohn disease, metabolic syndrome, and type 2 diabetes mellitus (Sabat et al 2020). Hidradenitis suppurativa has profound negative effects on patients' QoL, which often culminate in social withdrawal, unemployment, depression, and suicidal thoughts (Matusiak 2020). The estimated prevalence of HS varies substantially but most likely ranges between 0.7% and 1.2% in the European and US population (Nguyen et al 2021); the female-to-male ratio in Western populations is approximately 3:1, but the reverse is found in other parts of the world, such as in Japan and South Korea (Chu et al 2021, Ingram 2020). The role of race in the pathogenesis of HS is not well understood; in the US, those of African American and biracial ancestry appear to have a higher prevalence of HS, and some data also suggest that African American patients may have earlier disease onset than the remainder of the population (Shao et al 2022).

Adalimumab, a monoclonal antibody targeting the anti-inflammatory cytokine TNF-α, is the only therapy approved for HS treatment and is recommended for the treatment of moderate to severe HS (Hurley Stages II and III). Other biologics, including another TNF-α inhibitor, infliximab, may also be used for the treatment of HS, based on North America and EU guidelines (Hendricks et al 2021). Studies have reported significant dropout rates in patients being treated with adalimumab, primarily due to lack of efficacy (ranging from 27% to 50%; Frew et al 2020). A recent study also demonstrated that the median time to discontinuation from adalimumab was 36 weeks, which was comparable to other biologics used off-label for HS; this relatively short drug survival rate may be a result of the suboptimal efficacy of biologics and the higher inflammatory load in HS compared to other autoimmune diseases (Ring et al 2022).

Additionally, the antagonism of a single proinflammatory cytokine may not be sufficient in many patients with HS.

Other treatments, although not approved, are also used in HS and discussed in the treatment guidelines. Systemic antibiotics are the most commonly recommended therapy for treatment of moderate to severe HS, typically for at least 12 weeks (or as long-term maintenance or adjunct therapy). Other systemic oral drugs are also used as second- or third-line therapies, such as metformin, spironolactone, and retinoids (Hendricks et al 2021). There is not a strong level of scientific evidence on the use of antibiotics and the other systemic therapies in HS and data from PC studies are limited (Frew et al 2019), thus demonstrating a substantial unmet need for more efficacious therapies, evaluated in well-controlled clinical trials, and with potential to target diverse immunomodulatory mechanisms.

2.2. Povorcitinib

Povorcitinib is a novel, potent, and selective oral small-molecule inhibitor of the JAK class, with marked selectivity for JAK1. Povorcitinib potently inhibits JAK1 (IC_{50} [redacted] nM), with approximately [redacted] fold selectivity over the other JAK family members (JAK2, JAK3, and TYK2). Povorcitinib also demonstrated high potency in JAK1-dependent cell-based assays (IC_{50} [redacted] nM) and in other cell-based assays relevant to the pathogenesis of Th1/Th17 lymphocyte-mediated diseases by inhibiting the production of proinflammatory factors (eg, IL-17, MCP-1) induced by other cytokines, such as IL-23 and IL-6, with IC_{50} values of [redacted] nM. In contrast, povorcitinib showed significantly less inhibition (IC_{50} [redacted] nM) in cell-based assays dependent on JAK2 (eg, thrombopoietin- or GM-CSF–stimulated STAT phosphorylation), suggesting JAK1 selectivity in both biochemical and cellular assays.

Povorcitinib oral bioavailability is ≈100%, with a median time to maximum concentration of ≈2 to 4 hours, and a mean terminal half-life of ≈27 to 35 hours. The primary clearance pathway is hepatic, via metabolism through CYP3A4. Renal excretion is minimal, which is consistent with its high plasma protein binding.

Additional information is presented in the IB.

[redacted]

[redacted]

2.2.2. Clinical Experience

2.2.2.1. Clinical Pharmacokinetics in Phase 1 Studies

Single doses of povorcitinib up to 405 mg and multiple doses of povorcitinib up to 120 mg QD for up to 10 days have been evaluated in 2 Phase 1 studies in healthy participants. The most common TEAE was mild to moderate headache in 31% of participants who received povorcitinib in the multiple-dose study; the events resolved spontaneously following discontinuation of povorcitinib. Furthermore, decreases in platelet counts were observed among participants treated with povorcitinib at doses ≥ 60 mg QD. The magnitude of the decrease was larger for participants treated with 120 mg QD compared with those who received 60 mg QD, suggesting a dose-related trend. Of note, the participants with a treatment-emergent decrease in platelet counts still had this laboratory parameter within the normal range.

A population PK model demonstrated that the central volume of distribution was allometrically scaled with body weight. The typical value of drug clearance appears to be [redacted]% lower in females in comparison to males and [redacted]% higher and [redacted]% lower in Black and Asian participants, respectively, in comparison to the reference group of White participants and participants of other races. Overall, the gender and racial differences in PK were not considered significant.

A drug-drug interaction study in healthy volunteers was evaluated in 3 separate cohorts. Following coadministration with itraconazole (a strong CYP3A4 inhibitor), the total exposure was [redacted]% greater than when povorcitinib was administered alone; C_{max} and t_{max} remained similar, while $t_{½}$ increased from [redacted] to [redacted] hours. Following coadministration with rifampin (a strong CYP3A4 inducer), the total exposure was [redacted]% lower than when povorcitinib was administered alone, C_{max} was [redacted]% lower, t_{max} remained unchanged, and $t_{½}$ decreased from [redacted] to [redacted] hours. Following coadministration with esomeprazole (a PPI), the total exposure was comparable to povorcitinib administered alone; C_{max} was [redacted]% lower, and t_{max} and $t_{½}$ remained

unchanged. These results indicate that coadministration of povorcitinib with strong CYP3A4 inhibitors or strong/moderate inducers should be avoided, while PPIs or other pH-modifying agents do not have a substantial effect.

A study to evaluate the safety, tolerability, and PK of povorcitinib (45 or 90 mg QD) in healthy male Japanese participants was conducted. Povorcitinib was generally safe and well tolerated, with [redacted] SAE [redacted]; not drug related) and [redacted] Grade 3 TEAEs ([redacted]). Although the estimated PK exposures in Japanese participants for [redacted] mg QD will be higher by up to [redacted]% (in terms of peak concentration) and [redacted]% in terms of area under the plasma concentration-time curve than that of the non-Japanese participants receiving the same dose, the distribution range of exposure in Japanese participants is within the range observed in non-Japanese participants; therefore, this PK difference is not clinically meaningful.

2.2.2.2. Hidradenitis Suppurativa

2.2.2.2.1. INCB 54707-202

INCB 54707-202 was an open-label study that evaluated the safety and efficacy of povorcitinib 15 mg QD administered over 8 weeks in 10 adult participants with moderate to severe HS. There were no deaths or serious TEAEs in this study. One participant (10.0%) discontinued treatment with povorcitinib due to TEAEs. At least 1 TEAE was reported for 7 participants (70.0%). The most frequently reported TEAE in this study was upper respiratory tract infection. All TEAEs reported in this study were mild or moderate in severity.

Three participants (43%) achieved HiSCR at Week 8. The mean (STD) change from baseline in IHS4 was −2.1 (7.5) at Week 8. At Week 8, the mean (STD) change from baseline in HiSQoL was −15.3 (40.9). At Week 8, the mean (STD) change from baseline in worst skin pain score was −2.0 (1.8). Further results of this study have been reported by Alavi et al (2022).

2.2.2.2.2. INCB 54707-203

INCB 54707-203 was a randomized, PC study that evaluated the safety and efficacy of povorcitinib (30, 60, or 90 mg QD) administered over 8 weeks in 35 adult participants with moderate to severe HS. There were no deaths, serious TEAEs, or TEAEs leading to discontinuation of treatment. At least 1 TEAE was reported for 25 participants (71.4%). The most frequently reported TEAE in this study was fatigue.

In this study, 17 participants (65%) receiving povorcitinib (5 [56%] on 30 mg QD, 5 [56%] on 60 mg QD, 7 [88%] on 90 mg QD) versus 4 participants (57%) receiving placebo achieved HiSCR at Week 8. For IHS4, the mean (STD) changes from baseline at Week 8 were −9.4 (6.8), −21.4 (21.4), and −16.1 (22.9) for participants treated with 30 mg, 60 mg, and 90 mg, respectively, versus −10.7 (21.0) for the placebo group. The mean (STD) changes from baseline in HiSQoL were −34.3 (41.7), −39.0 (30.8), and −28.0 (21.4) for participants treated with 30, 60, or 90 mg, respectively, at Week 8 versus 3.4 (16.1) for placebo. At Week 8, the mean (STD) changes from baseline in worst skin pain score were −2.2 (2.2), −1.4 (1.4), and −3.1 (3.3) for participants treated with 30, 60 and 90 mg versus 0.3 (2.8) for placebo. Further results of this study have been reported by Alavi et al (2022).

2.2.2.2.3. INCB 54707-204

INCB 54707-204 is a randomized, PC, dose-range (povorcitinib 15, 45, or 75 mg) finding study in 209 adult participants with HS (Hurley Stage I, II, or III) with a total abscess and inflammatory nodule count ≥ 5. In this study, QD doses of study drug were administered for up to 16 weeks, followed by a 36-week OLE period and a 48-week LTE period. The OLE/LTE periods of this study were still ongoing at the time of the latest data cutoff date (15 DEC 2021).

2.2.2.2.3.1. Efficacy Summary

Povorcitinib demonstrated a dose-dependent improvement in HS lesions and on participants' QoL. Overall:

- HiSCR was achieved in 48.1% and 58.5% of participants in the povorcitinib 45 and 75 mg groups, respectively, as compared with 30.8% of participants in the placebo group at Week 12 and in 44.2% and 45.3% of participants in the povorcitinib 45 and 75 mg groups, respectively, as compared to 28.8% of participants in the placebo group at Week 16 (see Figure 2).
- Improvement in QoL was observed as early as Week 2 for the povorcitinib 45 and 75 mg groups as demonstrated via Skin Pain NRS (see Figure 3), as well as for HiSQoL, Itch NRS, DLQI, and FACIT-F (data not shown).
- Although comparable across the majority of efficacy and QoL endpoints, participants in the povorcitinib 75 mg group appeared to have a faster and trending better response than those in the povorcitinib 45 mg group, as shown for the achievement of clinical response (HiSCR) for participants with more severe disease (ANdT ≥ 11), improvement in skin pain, decrease in analgesic use, decrease in draining tunnel count, decrease in Itch NRS, and improvement in FACIT-F scores, as summarized in Table 4.

Figure 2: Proportion of HiSCR Responders During the Placebo-Controlled Period

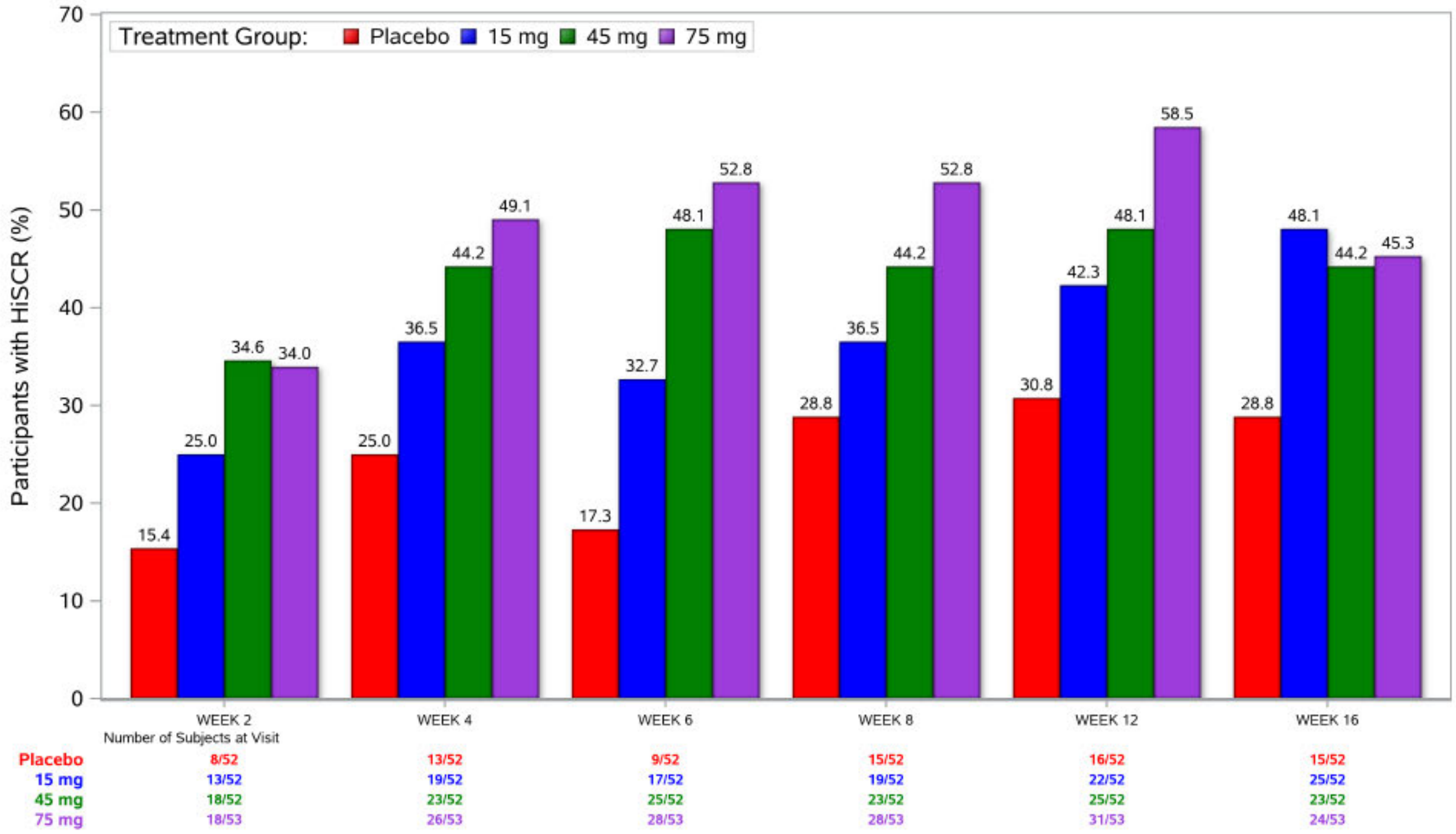

Figure 3: Mean (SE) Change From Baseline in Skin Pain NRS During the Placebo-Controlled Period

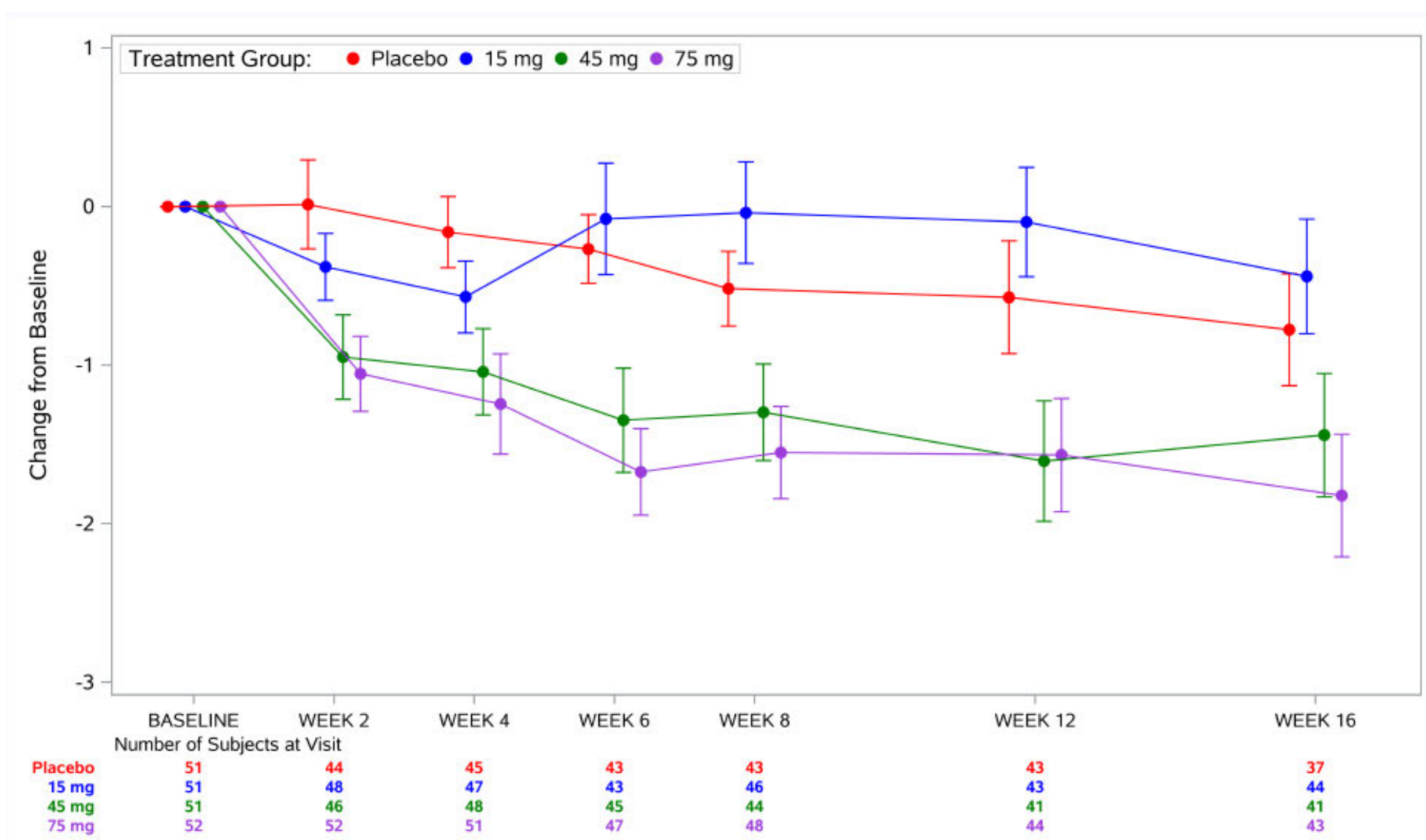

Table 4: [redacted]

Parameter	Visit	[redacted]	[redacted]	[redacted]	[redacted]
Proportion of participants with ANdT count ≥ 11 at baseline achieving HiSCR	[redacted]				
Proportion of participants achieving Skin Pain NRS30					
Proportion of participants with analgesic use					
Mean draining tunnel count for participants with ≥ 1 draining tunnel at baseline					
Proportion of participants achieving ≥ 4-point reduction in Itch NRS score from baseline					
Mean change in FACIT-F score from baseline					

2.2.2.2.3.2. Safety Summary

At the time of the primary completion data cutoff date (15 DEC 2021), all participants had either completed (174 [83.3%]) or discontinued from treatment (35 [16.7%]) in the 16-week PC period. Participants who completed treatment in the PC period (174 [100%]) entered the 36-week OLE period; of these, 28 participants (16.1%) completed treatment in the OLE period and started the 48-week LTE period, and 25 participants (14.4%) discontinued treatment in the OLE period.

Overall, povorcitinib was safe and well tolerated in adult participants with HS, with a low discontinuation rate due to TEAEs and few serious or Grade 3 or higher TEAEs. There were no fatal TEAEs. There was no increase in the incidence of TEAEs or other safety signs with increasing doses of povorcitinib. Further, there were no clinically meaningful changes in chemistry or hematology parameters, and no clinically meaningful changes were noted for vital signs.

During the PC period, the povorcitinib 75 mg group had the lowest incidence of serious TEAEs, Grade 3 or higher TEAEs, and TEAEs leading to the discontinuation of study drug. In the ongoing OLE period, the overall incidence rates of any TEAEs across groups were comparable to those in the PC period, with a slightly higher incidence of Grade 3 or higher TEAEs, as expected with longer duration of treatment. Nonetheless and despite the longer treatment duration, no clear differences in the incidence or type of TEAEs were observed in the 75 mg group (treated continuously with this dose during the PC and OLE periods) relative to the other 3 crossover groups.

The percentage of participants who experienced a TEAE in the placebo and povorcitinib 15, 45, and 75 mg groups was 65.4%, 59.6%, 60.0%, and 60.4%, respectively; in the same groups, the percentage of participants who had an SAE was 5.8%, 3.8%, 2.0%, and 0%, respectively. The TEAEs (with a cutoff ≥ 2.0% based on total povorcitinib values) and SAEs are respectively shown in Table 5 and Table 6. Overall, no clear dose-dependent safety trend was observed across the 3 doses of povorcitinib.

Table 5: INCB 54707-204 Summary of TEAEs (Individual TEAEs With a Cutoff ≥ 2.0% Based on Total Povorcitinib Values)

Parameter	Placebo (n = 52)	Povorcitinib 15 mg (n = 52)	Povorcitinib 45 mg (n = 50)	Povorcitinib 75 mg (n = 53)	Povorcitinib Total (n = 155)
Number (%) of participants with TEAEs	34 (65.4%)	31 (59.6%)	30 (60.0%)	32 (60.4%)	93 (60.0%)
TEAE grade					
Grade 1	21 (40.4%)	17 (32.7%)	15 (30.0%)	12 (22.6%)	44 (28.4%)
Grade 2	10 (19.2%)	12 (23.1%)	13 (26.0%)	19 (35.8%)	44 (28.4%)
Grade 3	3 (5.8%)	2 (3.8%)	1 (2.0%)	1 (1.9%)	4 (2.6%)
Grade 4	0 (0%)	0 (0%)	1 (2.0%)	0 (0%)	1 (0.6%)
MedDRA preferred term					
Fatigue	4 (7.7%)	4 (7.7%)	5 (10.0%)	6 (11.3%)	15 (9.7%)
Headache	5 (9.6%)	4 (7.7%)	2 (4.0%)	4 (7.5%)	10 (6.5%)
Diarrhea	1 (1.9%)	3 (5.8%)	2 (4.0%)	3 (5.7%)	8 (5.2%)
Nausea	5 (9.6%)	3 (5.8%)	2 (4.0%)	3 (5.7%)	8 (5.2%)
Acne	0 (0%)	1 (1.9%)	3 (6.0%)	3 (5.7%)	7 (4.5%)
Nasopharyngitis	0 (0%)	3 (5.8%)	3 (6.0%)	0 (0%)	6 (3.9%)
Vomiting	0 (0%)	3 (5.8%)	2 (4.0%)	1 (1.9%)	6 (3.9%)
COVID-19	1 (1.9%)	2 (3.8%)	2 (4.0%)	1 (1.9%)	5 (3.2%)
Constipation	2 (3.8%)	4 (7.7%)	0 (0%)	1 (1.9%)	5 (3.2%)
Decrease appetite	0 (0%)	1 (1.9%)	2 (4.0%)	2 (3.8%)	5 (3.2%)
Upper respiratory tract infection	2 (3.8%)	0 (0%)	1 (2.0%)	4 (7.5%)	5 (3.2%)
Back pain	1 (1.9%)	3 (5.8%)	1 (2.0%)	0 (0%)	4 (2.6%)
Folliculitis	0 (0%)	1 (1.9%)	1 (2.0%)	2 (3.8%)	4 (2.6%)
Fungal infection	0 (0%)	1 (1.9%)	1 (2.0%)	2 (3.8%)	4 (2.6%)
Urinary tract infection	1 (1.9%)	0 (0%)	1 (2.0%)	3 (5.7%)	4 (2.6%)

Table 6: Summary of Serious Treatment-Emergent Adverse Events During the Placebo-Controlled Period (ITT Population)

Parameter	Placebo (N = 52)	Povorcitinib Treatment Group 15 mg (N = 52)	45 mg (N = 50)	75 mg (N = 53)	Total (N = 155)
Number (%) of participants who had a serious TEAE	3 (5.8)	2 (3.8)	1 (2.0)	0	3 (1.9)
Fall	0	1 (1.9)	0	0	1 (0.6)
Intervertebral disc protrusion	0	0	1 (2.0)	0	1 (0.6)
Pneumonia	0	1 (1.9)	0	0	1 (0.6)
Pulmonary embolism	0	1 (1.9)	0	0	1 (0.6)
Rib fracture	0	1 (1.9)	0	0	1 (0.6)
Abscess limb	1 (1.9)	0	0	0	0
Atrial fibrillation	1 (1.9)	0	0	0	0
Bacterial infection	1 (1.9)	0	0	0	0
Cholecystitis	1 (1.9)	0	0	0	0

2.3. Study Rationale

2.3.1. Scientific Rationale for Study Design

Increased expression of JAK-mediated inflammatory cytokines in HS skin suggests a potential therapeutic strategy for treatment of HS with JAK inhibitors (Rumberger et al 2020, Zouboulis et al 2021). Povorcitinib, an oral JAK1 inhibitor, was evaluated in 2 Phase 2 trials (15, 30, 60, and 90 mg QD) in 45 participants and demonstrated proof of concept for JAK1 inhibition in HS by showing lesion count decrease, improvement in disease-specific PROs, and changes in disease-associated serum biomarkers (Alavi et al 2022). A third Phase 2 study with povorcitinib (15, 45, and 75 mg QD) in 209 participants further demonstrated a dose-dependent clinical response across lesion count decrease, HiSCR, and improvement in PROs, such as in Skin Pain NRS, Itch NRS, HiSQoL, DLQI, and FACIT-F (see Section 2.2.2.2.3). The collective efficacy and safety results suggest that povorcitinib represents a promising therapeutic agent in HS and supports further investigation of this molecule in Phase 3 studies.

The objective of this study is to confirm the safety and efficacy profile of povorcitinib in a larger population of participants with HS as part of Phase 3 studies. The population to be studied comprises participants 18 years of age and older who have had a diagnosis of moderate to severe HS (Hurley Stage II or III) for at least 3 months and who have a documented history of inadequate response to at least a 3-month course of at least 1 conventional systemic therapy (oral antibiotic or biologic drug) for HS (or demonstrated intolerance to, or have a contraindication to, a conventional systemic therapy for treatment of their HS), with HS lesions in at least 2 distinct anatomical areas and total abscess and inflammatory nodule count of at least 5. [redacted]

[redacted]

[redacted]

[redacted]

2.4. Benefit/Risk Assessment

The potential risks to participants, based on the available nonclinical and clinical data, are summarized in [redacted] Additionally, monitoring of AEs and applicable actions will be performed as indicated in Section 6.5.

Based on the expected mode of action of povorcitinib, participants randomly assigned to the active treatment group may experience clinically meaningful improvements in their HS lesions

during the PC period (12 weeks), and all participants will be offered povorcitinib during the EXT period (42 weeks). Participants will also contribute to the process of developing a novel anti-inflammatory agent for HS, a disease with high unmet need that is severely debilitating to patients' well-being and daily functioning.

Considering the cumulative safety information on povorcitinib, the potential risks identified are justifiable and appropriately balanced by the anticipated efficacy benefits expected to be afforded to participants. More detailed information about the known and expected benefits and risks and reasonably expected AEs of povorcitinib may be found in the IB.

[redacted]

[redacted]

3. OBJECTIVES AND ENDPOINTS

Table 8 presents the objectives and endpoints.

Table 8: Objectives and Endpoints

Objectives	Endpoints
**Primary**	
To evaluate the efficacy of povorcitinib.	• Proportion of participants who achieve Hidradenitis Suppurativa Clinical Response (HiSCR) at Week 12.<br>− HiSCR is defined as at least a 50% reduction from baseline in the total abscess and inflammatory nodule count, with no increase from baseline in abscess or draining tunnel count.
**Key Secondary**	
To further evaluate the efficacy of povorcitinib.	• Proportion of participants who achieve Hidradenitis Suppurativa Clinical Response 75 (HiSCR75) at Week 12.<br>− HiSCR75 is defined as at least a 75% reduction from baseline in the total abscess and inflammatory nodule count, with no increase from baseline in abscess or draining tunnel count.
To evaluate the effect of povorcitinib in reducing the incidence of flares.	• Proportion of participants with flare by Week 12.<br>− Participants who experience at least 1 flare over 12 weeks; flare is defined as at least a 25% increase in the total abscess and inflammatory nodule count with a minimum increase of 2 relative to baseline.
To evaluate the effect of povorcitinib in decreasing skin pain.	• Key secondary endpoint for regulatory purposes in US/FDA/aligned countries:<br>Proportion of participants with a ≥ 3-point decrease in Skin Pain Numeric Rating Scale (NRS) score at Week 12 among participants with baseline Skin Pain NRS score ≥ 3.<br>− Participants with a Skin Pain score of at least 3 at baseline and who experience at least a 3-point decrease in Skin Pain score at Week 12, relative to baseline. Skin Pain is an 11-point NRS, ranging from 0 (no skin pain) to 10 (worst skin pain).<br>• Key secondary endpoint for regulatory purposes in EU/EMA/aligned countries:<br>Proportion of participants who achieve Skin Pain NRS30 at Week 12 among participants with baseline Skin Pain NRS score ≥ 3.<br>− Participants with a Skin Pain score of at least 3 at baseline and who achieve at Week 12 Skin Pain NRS30, defined as at least a 30% reduction and at least 1-unit reduction from baseline in the Skin Pain NRS.

Table 8: Objectives and Endpoints (Continued)

Objectives	Endpoints
**Secondary**	
To further evaluate the effect of povorcitinib in decreasing skin pain.	• Secondary endpoint for regulatory purposes in US/FDA/aligned countries:<br>Proportion of participants who achieve Skin Pain NRS30 at Week 12 among participants with baseline Skin Pain NRS score ≥ 3.<br>− Participants with a Skin Pain score of at least 3 at baseline and who achieve at Week 12 Skin Pain NRS30, defined as at least a 30% reduction and at least 1-unit reduction from baseline in the Skin Pain NRS.<br>• Secondary endpoint for regulatory purposes in EU/EMA/aligned countries:<br>Proportion of participants with a ≥ 3-point decrease in Skin Pain NRS score at Week 12 among participants with baseline Skin Pain NRS score ≥ 3.<br>− Participants with a Skin Pain score of at least 3 at baseline and who experience at least a 3-point decrease in Skin Pain score at Week 12, relative to baseline. Skin Pain is an 11-point NRS, ranging from 0 (no skin pain) to 10 (worst skin pain).
To evaluate the effect of povorcitinib in improving fatigue.	• Proportion of participants with at least a 4-point increase from baseline in Functional Assessment of Chronic Illness Therapy – Fatigue (FACIT-F) score at Week 12.<br>− Participants with a baseline FACIT-F score ≤ 48 and who experience at least a 4-point increase in FACIT-F score at Week 12, relative to baseline. The FACIT-F scale is a 13-item questionnaire that assesses self-reported fatigue and its impact upon daily activities and function over the past 7 days, with scores ranging from 0 (worst fatigue) to 52 (no fatigue).
To evaluate the effect of povorcitinib on QoL during the PC and EXT periods.	• Mean change from baseline in Dermatology Life Quality Index (DLQI) score at each visit.
To further evaluate the efficacy of povorcitinib.	• Mean change from baseline in abscess count at each visit.<br>• Percentage change from baseline in abscess count at each visit.<br>• Mean change from baseline in inflammatory nodule count at each visit.<br>• Percentage change from baseline in inflammatory nodule count at each visit.<br>• Mean change from baseline in draining tunnel count at each visit.<br>• Percentage change from baseline in draining tunnel count at each visit.

Table 8: Objectives and Endpoints (Continued)

Objectives	Endpoints
**Secondary (continued)**	
To evaluate the efficacy of povorcitinib during the EXT period.	• Proportion of participants who achieve HiSCR at Week 24.<br>• Proportion of participants who achieve HiSCR75 at Week 24.<br>• Proportion of participants with flare by Week 24.<br>• Proportion of participants who achieved Skin Pain NRS30 at Week 24 among participants with baseline Skin Pain NRS score ≥ 3.<br>• Proportion of participants who achieve HiSCR at Week 54.<br>• Proportion of participants who achieve HiSCR75 at Week 54.<br>• Proportion of participants with flare by Week 54.<br>• Proportion of participants who achieved Skin Pain NRS30 at Week 54 among participants with baseline Skin Pain NRS score ≥ 3.
To evaluate the maintenance of response during the EXT period.	• Proportion of participants who achieve maintenance of HiSCR or greater response at each visit during the EXT period.<br>− Maintenance of response defined as participants who achieve HiSCR at Week 12 and maintain it or achieve greater response at each visit during the EXT period.<br>• Proportion of participants who achieve maintenance of HiSCR75 or greater response at each visit during the EXT period.<br>− Maintenance of response defined as participants who achieve HiSCR75 at Week 12 and maintain it or achieve greater response at each visit during the EXT period.
**Safety**	
To evaluate the safety and tolerability of povorcitinib.	• Frequency and severity of AEs, including changes in clinical laboratory results, vital signs, ECGs, and the results of physical examinations.
**Exploratory**	
To evaluate the efficacy of povorcitinib.	• Proportion of participants who achieve HiSCR at each visit.<br>• Proportion of participants who achieve HiSCR75 at each visit.<br>• Proportion of participants who achieve HiSCR90 at each visit.<br>− HiSCR90 is defined as at least a 90% reduction from baseline in total abscess and inflammatory nodule count, with no increase from baseline in abscess or draining tunnel count.<br>• Proportion of participants with a total abscess and inflammatory nodule count of 0, 1, or 2 at each visit.<br>• Proportion of participants who achieve AN50, AN75, AN90, and AN100 (relative to baseline) at each visit.<br>− Defined as total abscess and inflammatory nodule count change of ≥ 50%, ≥ 75%, ≥ 90%, and ≥ 100% relative to baseline, respectively.<br>• Proportion of participants who achieve ANdT50, ANdT75, ANdT90, and ANdT100 (relative to baseline) at each visit.<br>− Defined as abscess, inflammatory nodule, and draining tunnel count change of ≥ 50%, ≥ 75%, ≥ 90%, and ≥ 100% relative to baseline, respectively.

Table 8: Objectives and Endpoints (Continued)

Objectives	Endpoints
**Exploratory (continued)**	
To evaluate the efficacy of povorcitinib (continued).	• Mean change from baseline in total abscess and inflammatory nodule count at each visit.<br>• Percentage change from baseline in total abscess and inflammatory nodule count at each visit.<br>• Mean change from baseline in International Hidradenitis Suppurativa Severity Score System (IHS4) score at each visit.<br>− The IHS4 assesses the severity of HS, and the resulting score is calculated by the number of inflammatory nodules (multiplied by 1) plus the number of abscesses (multiplied by 2) plus the number of draining tunnels (multiplied by 4).<br>• Percentage change from baseline in IHS4 score at each visit.<br>• Proportion of participants who achieve IHS4-55 at each visit.<br>− IHS4-55 is defined as at least a 55% reduction from baseline in total IHS4 score.<br>• Proportion of participants in each of the IHS4 disease categories at each visit.<br>− IHS4 disease categories are described as follows: a total score of 3 or less signifies mild, 4-10 signifies moderate, and 11 or higher signifies severe disease.
To evaluate the effect of povorcitinib in reducing the incidence of flares.	• Proportion of participants with HS flares at each visit.<br>• Proportion of participants with at least 1 HS flare during the EXT period.<br>• Mean number of flares during the PC period.<br>• Mean number of flares during the EXT period.<br>• Mean time to the first flare during the PC period.<br>• Mean time to the first flare during the EXT period.
To evaluate the effect of povorcitinib on skin pain.	• Proportion of participants achieving Skin Pain NRS30 at each visit among participants with baseline Skin Pain NRS score ≥ 3.<br>• Proportion of participants with a ≥ 3-point decrease from baseline in Skin Pain score at each visit.<br>− Participants with a Skin Pain NRS score of at least 3 at baseline and who experience at least a 3-point decrease in Skin Pain score at each visit, relative to baseline.<br>• Proportion of participants with a ≥ 4-point decrease from baseline in Skin Pain score at each visit.<br>− Participants with a Skin Pain NRS score of at least 4 at baseline and who experience at least a 4-point decrease in Skin Pain score at each visit, relative to baseline.<br>• Mean change from baseline in Skin Pain NRS score at each visit.<br>• Percentage change from baseline in Skin Pain NRS score at each visit.<br>• Proportion of participants with analgesic (nonopioid and opioid) use at each visit.<br>• Proportion of participants with opioid use at each visit.

Table 8: Objectives and Endpoints (Continued)

Objectives	Endpoints
**Exploratory (continued)**	
To evaluate the effect of povorcitinib in decreasing draining tunnel count.	• Proportion of participants with at least a 1-unit decrease from baseline in draining tunnel count at each visit.<br>− Participants with at least 1 draining tunnel at baseline and who experience at least a 1-unit decrease in draining tunnel count at each visit, relative to baseline.<br>• Mean change from baseline in draining tunnel count at each visit.<br>• Percentage change from baseline in draining tunnel count at each visit.
To evaluate the effect of povorcitinib on itch.	• Proportion of participants who achieve Itch NRS30 at each visit for participants with Itch NRS score ≥ 3 at baseline.<br>− Itch NRS30 is defined as at least a 30% reduction and at least 1-unit reduction from baseline in the Itch NRS score (range from 0 [no itch] to 10 [worst itch imaginable]).<br>• Proportion of participants who achieve at least a 4-point reduction in Itch NRS score from baseline at each visit for participants with Itch NRS score ≥ 4 at baseline.<br>• Mean change from baseline in Itch NRS score at each visit.<br>• Percentage change from baseline in Itch NRS score at each visit.
To assess the need for rescue lesional treatment.	• Proportion of participants requiring rescue lesional treatment during the PC period.<br>• Proportion of participants requiring rescue lesional treatment during the EXT period.
To assess the need for systemic antibiotic rescue therapy.	• Proportion of participants requiring systemic antibiotic rescue therapy during the PC period.<br>• Proportion of participants requiring systemic antibiotic rescue therapy during the EXT period.
To evaluate the effect of povorcitinib on QoL.	• Mean change from baseline in Hidradenitis Suppurativa Quality of Life (HiSQoL) score at each visit.<br>• Percentage change from baseline in HiSQoL score at each visit.<br>• Percentage change from baseline in DLQI score at each visit.<br>• Proportion of participants with at least a 4-point decrease in DLQI score from baseline at each visit for participants with DLQI score ≥ 4 at baseline.<br>• Mean change from baseline in FACIT-F score at each visit.<br>• Percentage change from baseline in FACIT-F score at each visit.<br>• Proportion of participants with at least a 4-point increase in FACIT-F score at each visit.<br>• Mean change from baseline in Work Productive and Activity Impairment (WPAI)-HS score at each visit.<br>• [redacted]

Table 8: Objectives and Endpoints (Continued)

Objectives	Endpoints
[redacted]	

4. STUDY DESIGN

4.1. Overall Design

This is a Phase 3, multicenter, randomized, parallel-group, PC, double-blind study in adult participants (age ≥ 18 years) with moderate to severe HS (Hurley Stage II or III), HS lesions in ≥ 2 distinct anatomical areas, and total abscess and inflammatory nodule count ≥ 5 at screening and baseline and who have a history of failure of, intolerance to, or contraindication to an adequate course of conventional systemic therapy for HS. Approximately 600 participants will be randomized 1:1:1 to povorcitinib (45 or 75 mg QD) or placebo QD for 12 weeks; participants who complete the PC period may continue to a 42-week double-blind EXT period with povorcitinib 45 mg or 75 mg QD (see Figure 1). Participants will be stratified based on ANdT count (< 11 or ≥ 11) at baseline and previous treatment of HS with a biologic drug.

Participants randomized to povorcitinib at baseline will remain at their respective dose level (45 or 75 mg QD) throughout the PC and EXT periods, while participants randomized to placebo will receive povorcitinib 45 or 75 mg QD (1:1 ratio) at the Week 12 visit onward, after completion of the PC period. Treatment assignment during the PC and EXT periods will be blinded to investigators and participants.

Participants will be screened for up to 28 days before the first dose of study drug; participants who are randomized in the study will receive the first dose of study drug on Day 1, and the last dose within the PC period will occur at the Week 12 visit. The first dose of study drug within the 42-week EXT period will occur on the next day after the Week 12 visit; the last dose within the EXT period will occur on the day of the Week 54 visit (or last study drug administration known to the investigator or designee as reported in the EDC for patients who prematurely discontinue the study or are lost to follow-up). Participants who complete the 54-week treatment in this study may be eligible to participate in a separate extension study to further evaluate the safety and efficacy of the investigational product. For participants who do not participate in the separate extension study, a safety follow-up visit (EOS) should be conducted approximately 30 (+ 7) days after EOT, or last administration of study drug (in case of premature discontinuation/early termination), for all participants.

Lesion count assessments will be performed to determine clinical efficacy endpoints; participants will answer daily diary QoL questionnaires on skin pain, analgesic use, and itch and complete the study drug daily diary during the PC and EXT periods. Additional PRO questionnaires will be answered by participants at the study visits. Participants will be assessed for safety and tolerability throughout the study by monitoring the type, frequency, and severity of AEs and measuring vital signs and clinical laboratory parameters. Details of the assessments are presented in Table 3.

4.2. Overall Study Duration

The study begins when the first participant signs the study ICF. The end of the study is defined as the date of the last visit of the last participant in the study globally. A participant is considered to have completed the study if they have completed all study treatment visits; for participants who do not participate in the separate extension study, study completion includes the safety follow-up visit (see Table 3).

It is estimated that an individual will participate for approximately 14 months, including up to 28 days for screening, continuous treatment for 54 weeks (including the PC and EXT periods, as long as participants are receiving benefit and have not met any criteria for study ET/withdrawal), and 30 (+ 7) days for follow-up after the last dose of study drug.

4.3. Study Termination

The investigator retains the right to terminate study participation at any time, according to the terms specified in the study contract. The investigator/head of the study site (Japan) is to notify the IRB/IEC in writing of the study's completion or early termination, send a copy of the notification to the sponsor or sponsor's designee, and retain 1 copy for the site study regulatory file.

The sponsor may terminate the study electively, if, for example, required by regulatory decision or upon advice of the DSMB (see Section 5.6). If the study is terminated prematurely, the sponsor will notify the investigators/head of the study site (Japan), the IRBs and IECs, and regulatory bodies of the decision and reason for termination of the study. For Japan, the decision from the sponsor will be via the head of the study site(s), who will notify the investigators and the IRBs of the decision and reason for termination of the study.

5. STUDY POPULATION

Deviations from eligibility criteria are not allowed because they can potentially jeopardize the scientific integrity of the study, regulatory acceptability, and/or participant safety. Therefore, adherence to the criteria as specified in this Protocol is essential. Prospective approval of Protocol deviations to recruitment and enrollment criteria, also known as Protocol waivers or exemptions, are not permitted.

5.1. Inclusion Criteria

Participants are eligible to be included in the study only if all of the following criteria apply:

1. Ability to comprehend and willingness to sign a written ICF for the study.
2. Age ≥ 18 years at the time of signing the ICF.
3. Diagnosis of moderate to severe HS for at least 3 months prior to the screening visit.
4. Total abscess and inflammatory nodule count of at least 5 at both the screening and baseline visits (see Section 8.2.1).
5. HS lesions in at least 2 distinct anatomical areas (examples include but are not limited to left and right axilla or left and right inguinocrural fold), 1 of which must be at least Hurley Stage II or Hurley Stage III, at both the screening and baseline visits (see Section 8.2.2).
6. Documented history of inadequate response to at least a 3-month course of at least 1 conventional systemic therapy (oral antibiotic or biologic drug) for HS (or demonstrated intolerance to, or have a contraindication to, a conventional systemic therapy for treatment of their HS; see Section 6.6.1.1).
7. Agreement to NOT use topical and systemic antibiotics for treatment of HS during the placebo-controlled period (unless the systemic antibiotic rescue criterion is met per Section 6.6.10).
8. Agreement to NOT use a diluted bleach bath or topical antiseptic washes containing chlorhexidine gluconate or benzoyl peroxide on the areas affected by HS lesions during the placebo-controlled period. Note: Over-the-counter soap and water is allowed.
9. Agreement to use contraception (see Appendix A), as follows:
    a. Female participants of childbearing potential (WOCBP) must agree to take appropriate precautions to avoid pregnancy with a highly effective method (ie, at least 99% certainty) from screening through 90 days after the last dose of study drug. Permitted methods that are at least 99% effective in preventing pregnancy should be communicated to the participants and their understanding confirmed. Note: This criterion does not apply to women of nonchildbearing potential (WONCBP), as defined in Appendix A.
    b. All women must agree to not donate eggs (ova, oocytes) for the purposes of assisted reproduction during the study and for at least 90 days after receiving the last dose of study drug.

   c. A male participant who is sexually active with a woman of childbearing potential (WOCBP) must agree to use a highly effective barrier method of birth control (as described in Appendix A) during the study and for at least 90 days after receiving the last dose of study drug.
   d. All men must agree to not donate sperm during the study and for at least 90 days after receiving the last dose of study drug.

10. Willing and able to comply with the study protocol and procedures.

5.2. Exclusion Criteria

Participants are excluded from the study if any of the following criteria apply:

1. Presence of > 20 draining tunnels (fistulas) at either the screening or baseline visit.
2. Women who are pregnant (or who are considering pregnancy) or breastfeeding.
3. Concurrent conditions or history of other diseases, as follows:
   a. Thrombocytopenia, coagulopathy, or platelet dysfunction.
   b. Venous and arterial thrombosis, deep vein thrombosis, pulmonary embolism, stroke, moderate to severe heart failure (NYHA Class III or IV), cerebrovascular accident, myocardial infarction, coronary stenting, or coronary arterial revascularization with bypass graft surgery (CABG).
   c. Diagnosis of other significant cardiovascular diseases, including but not limited to angina, peripheral arterial disease, or uncontrolled arrhythmias such as atrial fibrillation, supraventricular tachycardia, ventricular tachycardia, and forms of carditis, etc.
   d. Uncontrolled hypertension, as defined by a confirmed systolic blood pressure > 160 mm Hg or diastolic blood pressure > 100 mm Hg.
   e. Participants who are permanently bedridden or wheelchair assisted.
   f. Recipient of an organ transplant that requires continued immunosuppression.
   g. Any malignancies or history of malignancies with the exception of adequately treated or excised nonmetastatic basal cell or squamous cell cancer of the skin.
   h. Conditions that could interfere with drug absorption, including but not limited to short-bowel syndrome.
   i. Chronic or recurrent infectious disease, including but not limited to chronic renal infection, chronic chest infection (eg, bronchiectasis), recurrent urinary tract infection (recurrent pyelonephritis or chronic nonremitting cystitis), fungal infection, prior prosthetic joint infection at any time, or open, draining, or infected skin wounds or ulcers.
   j. Current or history of disseminated herpes zoster, or recurrent (more than 1 episode of) dermatomal herpes zoster.
   k. Current or history of disseminated herpes simplex.
   l. Active systemic infection or any active infection that, based on the investigator's clinical assessment, makes the participant an unsuitable candidate for the study.
   m. Any other active skin disease or condition (eg, bacterial, fungal, or viral infection) that may interfere with the course, severity, or assessments of HS.

n. Any clinically significant medical condition (other than HS) or any other reason that the investigator determines would interfere with the participant's participation in this study or would make the participant an unsuitable candidate to receive study drug or would put the participant at risk by participating in the study.
o. Any clinically significant medical condition other than HS, as determined by the investigator, that is not adequately controlled with appropriate treatment or may interfere with the course, severity, or assessments of HS.
p. Albinism.

4. A screening 12-lead ECG that demonstrates clinically significant abnormalities requiring treatment (eg, acute myocardial infarction, serious tachyarrhythmias or bradyarrhythmias) or that is indicative of serious underlying heart disease (eg, cardiomyopathy, major congenital heart disease, low voltage in all leads, Wolff-Parkinson-White syndrome) or criteria associated with Q wave interval (QT)/Fridericia-corrected Q wave interval (QTcF) abnormalities.
5. Have undergone significant trauma or major surgery (per investigator's assessment) within 30 days preceding the screening visit.
6. History of clinically significant (per investigator's judgment) drug or alcohol abuse within 6 months preceding the screening visit.
7. History of treatment failure with any systemic or topical JAK inhibitor for HS or any other inflammatory condition (see Section 6.6.3).
8. Receipt of medical treatment or investigational drugs within the following interval before the baseline visit:

   a. < 12 weeks or 5 half-lives (if known), whichever is longer, for any investigational or experimental treatments.
   b. < 12 weeks or 5 half-lives (if known), whichever is longer, for immunomodulating biologic drugs (see Section 6.6.4).
   c. < 8 weeks for live vaccine, or planning to receive live vaccine during the course of the study or within 8 weeks after the last dose of study drug.
   d. < 4 weeks for any topical or systemic JAK inhibitor (see Section 6.6.3).
   e. < 4 weeks for systemic immunosuppressive or immunomodulating small-molecule drugs (eg, corticosteroids [oral or intravenous], avacopan, IRAK4 inhibitors, methotrexate, cyclosporine, dapsone, azathioprine).

      Note 1: Use of corticosteroid inhalers and intranasal sprays is allowed.
      Note 2: Use of oral corticosteroids for nondermatologic conditions (eg, asthma exacerbation, bronchitis) is allowed for no longer than 7 days if deemed acceptable by the investigator and the sponsor (or designee).

   f. < 4 weeks for surgical, laser, or any phototherapy intervention in areas with HS lesions.

g. < 2 weeks for other systemic therapies for HS (eg, retinoids, antihypertensive, antihyperglycemics, and antiandrogens such as acitretin, isotretinoin, metformin, spironolactone, and finasteride) with potential therapeutic impact.

   Note: Per discretion of the investigator (or designee), these drugs are allowed during the study if not prescribed for HS treatment and if at a stable dose and the dose is not anticipated to change during the course of the study.

h. < 2 weeks for systemic anti-infectives for HS treatment (eg, antibiotics, antivirals, antifungals).
i. < 1 week for any oral or topical PDE4 inhibitor (eg, apremilast, crisaborole).
j. < 1 week for topical anti-infectives for HS lesions (eg, antibiotics, antivirals, antifungals).
k. < 1 week for topical products containing chlorhexidine gluconate, or benzoyl peroxide, and topical diluted bleach bath to treat HS. Note: Over-the-counter soap and water are not restricted.
l. < 1 week for any topical drug applied onto HS lesions.
m. < 1 week for topical or intralesional corticosteroids.

   Note: Use of topical corticosteroids for dermatologic diseases other than HS (eg, atopic dermatitis, psoriasis) is allowed for areas not being treated for HS. The total body surface area involvement for other dermatologic diseases should not interfere with the safety of the participant or the HS assessments.

n. < 1 week or 5 half-lives (if known), whichever is longer, for strong systemic CYP3A4 inhibitors and strong and moderate systemic CYP3A4 inducers (see Appendix B). Examples include but are not limited to the following: rifampicin/rifampin, ketoconazole, itraconazole, carbamazepine, ritonavir, St John's wort, grapefruit/grapefruit juice, and Seville oranges.

9. At the screening visit, any of the laboratory abnormalities defined in Table 9.

Table 9: Exclusionary Laboratory Values

Laboratory Parameter		Exclusion Criterion
**Hematology**		
a	Platelets	< 100 × 10^9/L
b	Hemoglobin	< 9 g/dL
c	Absolute neutrophil count (ANC)	< 1.5 × 10^9/L
d	Total white blood cell (WBC) count (leukocyte count)	< 3.0 × 10^9/L
e	Absolute lymphocyte count	< 0.8 × 10^9/L
**Hepatic**		
f	ALT	> 2 × ULN
g	AST	> 2 × ULN
h	Total bilirubin	> 1.5 × ULN<br>(Note: Participants with clinical diagnosis of Gilbert syndrome may have a direct bilirubin measured and would be eligible provided the direct bilirubin is < ULN)
**Renal**		
i	Estimated glomerular filtration rate	< 45 mL/min per 1.73 m^2<br>Note: Based on the simplified 4-variable Modification of Diet in Renal Disease (MDRD) formula

10. Evidence of infection with *Mycobacterium tuberculosis* (ie, TB) as defined in Section 8.3.5.2.
11. Active HIV or acquired immunodeficiency syndrome. Active HIV is defined as confirmed positive anti-HIV antibody test (see Section 8.3.5.3).
12. Evidence of HBV or HCV infection or risk of reactivation (see Section 8.3.5.4).
13. Known hypersensitivity or severe reaction to povorcitinib or excipients of povorcitinib (refer to the IB) and/or other products in the same class.
14. Any condition, laboratory result, or result of screening assessments that would, in the investigator's and sponsor's (or designee's) judgment, interfere with full participation in the study, including administration of study drug and attending required study visits, pose a significant risk to the participant, or interfere with interpretation of study data.
15. The following participants are excluded in France: vulnerable populations according to article L.1121-6 of the French Public Health Code and adults under legal protection or who are unable to express their consent per article L.1121-8 of the French Public Health Code.

5.3. Lifestyle Considerations

5.3.1. Contraception

5.3.1.1. Female Participants

All female participants who are of childbearing potential must agree to use a highly effective method of contraception (ie, at least 99% certainty) consistently and correctly for the duration of the study and for at least 90 days after the last dose of investigational product.

The investigator or designee, in consultation with the participant, will confirm that the participant has selected an appropriate method of contraception for the individual participant from the permitted list of contraception methods (see Appendix A) and will confirm that the participant has been instructed in its consistent and correct use.

At timepoints indicated in the SoA (see Table 3), the investigator or designee will inform the participant of the need to use highly effective contraception consistently and correctly. Participants need to affirm their consistent and correct use of at least 1 of the selected methods of contraception (see Appendix A). In addition, the investigator or designee will instruct the participant to call immediately if the selected contraception method is discontinued or if pregnancy is known or suspected.

5.3.1.2. Male Participants

All male participants must be willing to take appropriate precautions to avoid fathering children (with at least 99% certainty) consistently and correctly (as outlined in Appendix A) for the duration of the study and for at least 90 days after the last dose of investigational product. If the male participant has a partner who is of childbearing potential, the partner should also use contraception as outlined in Appendix A.

5.3.2. Meals and Dietary Restrictions

- Study drug will be taken orally, preferably in the morning and at about the same time each day, with a full glass of water. The study drug can be taken with or without food. [redacted] [Table 3]). Further instructions on study drug handling are outlined in Appendix C.
- On any of the study visit days, participants should not smoke or ingest caffeine during the 30 minutes prior to blood pressure and heart rate measurements.
- On study visit days when a lipid panel blood sample will be collected (Baseline/Day 1; Weeks 6, 12, 18, 24, and 54; and EOS/Safety Follow-Up; see Table 3), participants should comply with the fasting requirement for at least 8 hours prior to the visit. Water and permitted nonstudy medications are allowed (see Section 6.6.6).
- Participants must abstain from consumption of grapefruit or grapefruit juice (pomelos, exotic citrus fruits, grapefruit hybrids) and of Seville oranges from 1 week before the start of study drug until after the final dose of study drug.

- Participants must abstain from consumption of dietary supplements containing St John's wort (*Hypericum perforatum*) from 1 week before the start of study drug until after the final dose of study drug.

5.3.3. Prohibited Therapies During the Placebo-Controlled Period

Some commonly used therapies may have an impact on HS and are specifically prohibited in this study as described in Section 6.6.

5.4. Screen Failures

Screen failures are defined as participants who consent to participate in the clinical study but are not subsequently randomly assigned to study drug.

Tests with results that fail eligibility requirements may be repeated once during screening if the investigator believes the result to be in error. Additionally, a participant who fails screening may repeat the screening process 1 time if the investigator believes that there has been a change in eligibility status. Participants who rescreen must reconsent and be assigned a new participant number.

All screening procedures with the possible exceptions noted below will be repeated during rescreening. The participant must meet all the eligibility criteria at the time of rescreening in order to qualify for the study. There is no minimum period of time a participant must wait to rescreen for the study. If the participant had a complete initial screening evaluation including the following assessments, these tests will not be required to be repeated for rescreening, provided the conditions noted in Section 5.1 and Section 5.2 are met, there are no changes in the participant's medical history that would warrant retesting, and no more than 90 days have passed:

- HBV, HCV, and HIV serology
- IGRA (QuantiFERON-TB Gold test [or IGRA equivalent such as T-SPOT® test] and/or local PPD skin test, if required)
- ECG

5.5. Replacement of Participants

No participants will be replaced at any time during this study. However, as noted in COVID-19–related guidance (see Appendix E), due to the evolving situation of the COVID-19 pandemic, the sponsor may decide to recruit additional participants in the study beyond the expected number (eg, if a substantial number of participants withdraw early from the study).

5.6. Data Safety Monitoring Board

This study will use an external DSMB to monitor safety. The voting members of the committee are external to the sponsor and will not be involved with the study in any other way (eg, they cannot be study investigators) and will have no competing interests that could affect their roles with respect to the study.

The DSMB will make recommendations to the sponsor regarding steps to ensure both participant safety and the continued ethical integrity of the study. Specific details regarding composition,

responsibilities, and governance, including the roles and responsibilities of the various members of the clinical study team, and requirements for the proper documentation of DSMB reports, minutes, and recommendations will be described in a separate DSMB charter, which will be reviewed and approved by all DSMB members.

5.7. Cardiovascular Adjudication Committee

An independent committee of physician experts in cardiovascular adjudication will be utilized to assess potential cardiovascular and thromboembolic AEs. Details will be described in a separate CAC charter.

6. STUDY TREATMENT

6.1. Study Treatment Administered

Information regarding study drug and administration is provided in Table 10. Participants will record study drug administration in a daily diary. Further information regarding study drug administration is provided in Appendix C.

The COVID-19 global pandemic may present challenges to the normal conduct of this study, requiring the outline of potential mitigation strategies described in Appendix E.

Table 10: Study Treatment Information

	**Study Treatment**	
**Study treatment name**	Povorcitinib	Matching placebo
**Mechanism of action**	JAK1 inhibitor	Not applicable
**Dose formulation**	Tablet, oral	Tablet, oral
**Unit dose strength(s) /dose level(s)**	45 mg: 1 tablet<br>75 mg: 1 tablet	Placebo: 1 tablet
**Administration instructions**	Povorcitinib or matching placebo will be taken orally QD, preferably in the morning and at about the same time each day, with a full glass of water, with or without food (see Appendix C for further information).<br>Note: Dose will be administered at the study site at the Baseline/Day 1, Week 3, Week 12, and Week 15 visits.	
**Packaging and labeling**	Povorcitinib will be provided as 45-mg or 75-mg tablets; both strengths match each other in smell, taste, and appearance. Placebo tablets will match the povorcitinib tablets in smell, taste, and appearance. Tablets will be packaged in bottles.<br>Each container will be labeled as required per country requirement.	
**Storage**	Ambient (15°C-30°C/59°F-86°F)	
**Status of treatment in participating countries**	Investigational	

6.2. Preparation, Handling, and Accountability

Note: For Japan, the term "investigator" below also represents the investigational drug storage manager.

The investigator or designee must confirm appropriate temperature conditions have been maintained during transit for all study treatments received and any discrepancies are reported and resolved before use of the study treatment.

Only participants enrolled in the study may receive study treatment, and only authorized site staff may supply or administer study treatment. All study treatment must be stored in a secure, environmentally controlled, and monitored (manual or automated) area in accordance with the labeled storage conditions with access limited to the investigator and authorized site staff.

The investigator or designee is responsible for study treatment accountability, reconciliation, and record maintenance (ie, receipt, reconciliation, and final disposition records). Inventory and accountability records must be maintained and readily available for inspection by the study monitor and are open to inspection at any time by any applicable regulatory authorities. The investigator or designee must maintain records that document the following:

- Delivery of study drug to the study site.
- Inventory of study drug at the site.
- Participant use of the study drug, including tablet counts from each supply dispensed.
- Return of study drug to the investigator or designee by participants.

The investigational product must be used only in accordance with the Protocol (see Appendix C). The investigator or designee will also maintain records adequately documenting that the participants were provided the specified study drug. These records should include dates, quantities, and any available batch or serial numbers or unique code numbers assigned to the investigational product and study participants.

Completed accountability records will be archived by the site. The investigator or designee will be expected to collect and retain all used, unused, and partially used containers of study drug until verified by the study monitor (unless otherwise agreed to by the sponsor). At the conclusion of the study, the investigator or designee will oversee the destruction of any remaining study drug according to institutional SOPs. If, however, local procedures do not allow on-site destruction, shipment of the study drug back to the sponsor is allowed. In this case, the site should (where local procedures allow) maintain the investigational supply until the study monitor inspects the accountability records in order to evaluate compliance and accuracy of accountability by the investigative site. At sites where the study drug is destroyed before monitor inspection, the monitors rely on documentation of destruction per the site SOP.

Further guidance and information for the final disposition of unused study treatments are provided in the study materials provided to sites.

6.3. Measures to Minimize Bias: Randomization and Blinding

All participants will be centrally assigned to study treatment using an IRT system (see Section 8.1.3). The system will assign in a 1:1:1 ratio (povorcitinib 45 mg QD, povorcitinib

75 mg QD, placebo QD), stratified by ANdT count (< 11 or ≥ 11) and previous treatment of HS with a biologic drug (yes or no). Before the study is initiated, the telephone number and call-in directions for the IRT and/or the log-in information and directions for the IRT will be provided to each site. Full details will be provided in the IRT Study Reference Manual.

After the database lock for the primary analysis (Week 12), the sponsor will be unblinded, but investigators and participants will remain blinded to individual treatment assignments through study completion. This is implemented to minimize bias throughout the PC and EXT periods of the study.

Emergency unblinding will occur if an AE requires the investigator to be made aware of the participant's treatment assignment (see emergency unblinding procedures in Section 9.7 and refer to the IRT Study Reference Manual).

6.4. Study Treatment Compliance

Compliance with all study-related treatments should be emphasized to the participant by the site personnel, and appropriate steps should be taken to optimize compliance during the study. Compliance with study drug will be calculated by the sponsor based on the drug accountability documented by the site staff and monitored by the sponsor/designee. Participants will be instructed to bring all unused study drugs with them to the study visits in order for site personnel to conduct tablet counts to assess study drug accountability.

Participants' compliance must be within [redacted]% to [redacted]%, assessed at each study visit by the investigator (or designee). If outside of this range, it will be considered a protocol deviation. Participants consistently noncompliant with the study drug may be withdrawn from the study. The decision on withdrawal will be made by the investigator after consultation with the sponsor, and relevant correspondence will be archived in the site study file.

Participants who are randomized in the study will receive the first dose of study drug on Day 1. For the purposes of compliance calculation, the last dose of study drug within each compliance period will be taken on the day of the respective study visit. The last dose within the PC period will occur at the Week 12 visit. The first dose of the study drug within the 42-week EXT period will occur on the next day after the Week 12 visit; the last dose within the EXT period will occur on the day of the Week 54 visit or last study drug administration known to the investigator (or designee) as reported in the EDC (for participants who prematurely discontinue the study or are lost to follow-up).

[redacted]

6.5.1. Criteria and Procedures for Dose Interruption and Discontinuation of Study Drug

[redacted]

[redacted]

[redacted]

[redacted]

[redacted]

6.6. Concomitant Medications and Procedures

6.6.1. Prior HS Therapy

Any topical and systemic treatments for HS since initial diagnosis and any prescribed treatments for HS prior to study entry will be recorded in the eCRF. Information associated with response to prior HS therapy and reason for discontinuation will also be recorded in the eCRF. Examples of commonly used therapies for HS include, but are not limited to, the following:

- Topical antibiotics (eg, clindamycin)
- Other topical treatments (eg, azelaic acid, benzoyl peroxide, bleach baths/sodium hypochlorite, chlorhexidine, resorcinol, retinoids [isotretinoin, acitretin, alitretinoin], steroids, zinc pyrithione)
- Systemic antibiotics (eg, ciprofloxacin, clindamycin, dapsone, doxycycline, ertapenem, erythromycin, linezolid, metronidazole, minocycline, moxifloxacin, rifampin/rifampicin, tetracycline)
- Systemic hormonal therapies (eg, antiandrogen contraceptives [ethinyl estradiol, cyproterone acetate], finasteride, metformin, spironolactone)
- Systemic or topical JAK inhibitors (see examples in Section 6.6.3)

- Other systemic therapies (eg, apremilast, avacopan, azathioprine, colchicine, cyclosporine, methotrexate, retinoids [isotretinoin, acitretin, alitretinoin], steroids)
- Biologics, adalimumab (Humira®) or biosimilars
- Biologics, other anti–TNF-α (infliximab, etanercept)
- Biologics, anti-IL17A/anti-IL17A/F (secukinumab [Cosentyx®], bimekizumab [Bimzelx®], ixekizumab [Taltz®])
- Other biologics (see examples in Section 6.6.4)

[redacted]

[redacted]

[redacted]

[redacted]

6.6.6.2. Wound Care Dressings

Concomitant use of wound care dressings for HS is allowed; however, options are limited to alginates, hydrocolloids, and hydrogels.

6.6.6.3. COVID-19 Vaccination

Participants who elect to receive non–live attenuated types of COVID-19 vaccines during the study may continue study drug administration.

[redacted]

[redacted]

[redacted]

6.6.9. Lesion Rescue

In the event that a particularly painful HS lesion occurs that necessitates an acute intervention, the investigator (or designee) will have the option to perform Protocol-allowed interventions. Two types of interventions are allowed:

- Injection with intralesional corticosteroid
  - Per local standard of care. An example includes, but is not limited to, triamcinolone acetonide suspension (at a concentration of up to 5 mg/mL, up to 1 cc).
  - Information on the intralesional corticosteroid must be captured in the eCRF.
- Incision and drainage of the lesion
  - After incision and drainage is performed, the participant is allowed to use over-the-counter soap and water or antiseptic wash locally where rescue was performed.

- Other systemic and topical therapies following incision and drainage (including antibiotics) follow the restrictions outlined in Section 6.6.7 and Section 6.6.8.
- Concomitant use of wound care dressings is allowed; however, options are limited to alginates, hydrocolloids, and hydrogels (see Section 6.6.6.2).
- Concomitant therapies associated with the lesion intervention(s) must be captured in the appropriate eCRF.

Each event of lesion rescue will also be captured as part of the HS lesion assessment and count (see Section 8.2.1).

The maximum number of rescue lesions per each period of the study is outlined below.

- PC period:
  - A total of 2 Protocol-allowed interventions are permissible through Week 12.
  - An intervention can occur maximally on 2 different lesions at the same visit or on the same lesion at 2 different study visits.
  - The same lesion cannot be treated 2 times at the same visit.
  - If a participant requires more than 2 interventions within the first 12 weeks, then they must be discontinued from treatment and undergo the ET visit.
- EXT period:
  - Maximum of 2 interventions every 4 weeks.
  - An intervention can occur on 2 different lesions at the same visit or on the same lesion at 2 different study visits.
  - If a participant requires more than 2 interventions within a 4-week period, then they must be discontinued from treatment and undergo the ET visit.

6.6.10. Systemic Antibiotic Rescue Therapy

At Week 9, if a participant experiences an increase in their AN count such that the total count is ≥ 150% of their baseline AN count, systemic antibiotic rescue medication may be initiated; study drug interruption is not required.

Participants who qualify may initiate treatment following local standard of care guidelines. Examples include, but are not limited to, minocycline or doxycycline up to 100 mg orally twice daily. Rifampin/rifampicin is not allowed due to potential drug-drug interactions with the study drug (see Appendix B). The maximum recommended duration of treatment should not exceed 12 weeks.

The dosing regimen must remain stable throughout study participation. Otherwise, concomitant use of systemic antibiotic therapy for treatment of HS is not allowed. Rescue systemic antibiotic therapy should be captured in the source and the appropriate eCRF.

The calculation will be performed as 1.5 * (baseline AN count), with examples shown in Table 12.

Table 12: Criteria for Systemic Antibiotic Rescue Therapy

Baseline AN Count	Calculation	Minimum AN Count to Be Eligible for Systemic Antibiotic Rescue
9	1.5 * (9) = 13.5	14
20	1.5 * (20) = 30	30

6.7. Treatment After the End of the Study

Participants who complete the 54-week treatment in this study may be eligible to participate in a separate extension study to further evaluate the safety and efficacy of the investigational product.

7. DISCONTINUATION OF STUDY TREATMENT AND PARTICIPANT WITHDRAWAL

7.1. Discontinuation of Study Treatment

7.1.1. Reasons for Discontinuation

Participants **must** be discontinued from study treatment for the following reasons:

- The participant becomes pregnant.
- Consent is withdrawn.

  Note: Consent withdrawn means that the participant has explicitly indicated that they do not want to be followed any longer; in this case no further data, except data in the public domain, may be solicited from or collected on the participant. Consent may also be partial. Participants may choose to discontinue study drug and remain in the study to complete the safety follow-up visit.
- Further participation would be injurious to the participant's health or well-being, in the investigator's medical judgment.
- Any AE of unacceptable severity as noted in Section 6.5.
- Participant meets discontinuation criteria as noted in Section 6.5.
- Participant has received a prohibited medication or procedure as described in Section 6.6.8.
- Participant has received a prohibited systemic rescue therapy/regimen other than those described in Section 6.6.10.
- The study is terminated by the sponsor.
- The study is terminated by the local health authority, IRB, or IEC.

A participant **may** be discontinued from study treatment as follows:

- If, during the course of the study, a participant is found not to have met eligibility criteria, the medical monitor, in collaboration with the investigator, will determine whether the participant should be withdrawn from study treatment.
- If a participant is noncompliant with study procedures or study drug administration in the investigator's opinion, the sponsor should be consulted for instruction on handling the participant.

7.1.2. Discontinuation Procedures

In the event that the decision is made to permanently discontinue the study treatment, the EOT/ET visit should be conducted (see Section 8.7). Reasonable efforts should be made to have the participant return for a safety follow-up visit. These visits are described in Table 3. The last date of the last dose of study drug and the reason for discontinuation of study drug will be recorded in the eCRF.

If a participant is discontinued from study treatment:

- The reason(s) for discontinuation must be documented in the participant's medical record and the primary reason for discontinuation must be included in the eCRF.
- The EOT/ET visit should be performed and date recorded.
- The status of the participant should be updated to EOT in the IRT.
- Participants must be followed for safety until the time of the follow-up visit or until study drug–related toxicities resolve, return to baseline, or are deemed irreversible, whichever is longest.

If the participant discontinues study treatment and actively withdraws consent for collection of follow-up data (safety follow-up or disease assessment), then no additional data collection should occur; however, participants will have the option of withdrawing consent for study treatment but continuing in the safety follow-up period of the study for safety/efficacy assessments.

7.2. Participant Withdrawal From the Study

A participant may withdraw from the study at any time at their own request or may be withdrawn at any time at the discretion of the investigator for safety, behavioral, compliance, or administrative reasons.

If a participant withdraws from the study, they may request destruction of any samples taken and not tested, and the investigator must document this in the site study records. If the participant withdraws consent for disclosure of future information, the sponsor may retain and continue to use any data collected before such a withdrawal of consent. See Table 3 for data to be collected at the time of study withdrawal and follow-up and for any further evaluations that need to be completed.

7.3. Lost to Follow-Up

A participant will be considered lost to follow-up if they repeatedly fail to return for scheduled visits and are unable to be contacted by the study site. The following actions must be taken if a participant fails to return to the clinic for a required study visit:

- The site must attempt to contact the participant and reschedule the missed visit as soon as possible and counsel the participant on the importance of maintaining the assigned visit schedule and ascertain whether or not the participant wishes to and/or should continue in the study.
- Before a participant is deemed lost to follow-up, the investigator or designee must make every effort to regain contact with the participant (where possible, 3 telephone calls and, if necessary, a certified letter to the participant's last known mailing address or local equivalent methods). These contact attempts should be documented in the participant's medical record.
- Should the participant continue to be unreachable, they will be considered to have withdrawn from the study.

8. STUDY ASSESSMENTS AND PROCEDURES

See Appendix E for COVID-19–related guidance.

8.1. Administrative and General Procedures

8.1.1. Informed Consent Process

- The investigator or their representative will explain the nature of the study to the participant or their legally authorized representative and answer all questions regarding the study.
  - Informed consent must be obtained before any study-related procedures are conducted, unless otherwise specified by the Protocol.
  - Informed consent must be obtained using the IRB/IEC-approved version in a language that is native and understandable to the participant. An ICF template will be provided by the sponsor or its designee. The sponsor or its designee must review and acknowledge the site-specific changes to the ICF template. The ICF must include a statement that the sponsor or its designee and regulatory authorities have direct access to participant records.
  - The ICF must contain all required elements including optional samples/procedures (eg, optional biopsy) and describe the nature, scope, and possible consequences of the study in a form understandable to the study participant.
- Participants must be informed that their participation is voluntary. Participants or their legally authorized representative will be required to sign a statement of informed consent that meets the applicable requirements and regulations for the countries in which the study is being conducted as well as the IRB/IEC or study center.

- The participant must be informed that their personal data collected for the study will be used by the sponsor and/or their designee(s) in accordance with local data protection laws. The level of disclosure must also be explained to the participant.
- The participant must be informed that their medical records may be examined by Clinical Quality Assurance auditors or other authorized personnel appointed by the sponsor, by appropriate IRB/IEC members, and by inspectors from regulatory authorities.
- The medical record must include a statement that written informed consent was obtained before the participant was enrolled in the study and the date the written consent was obtained. The authorized person obtaining the informed consent must also sign the ICF.
- Participants who are current or past long-time smokers must be informed of the potential risks associated with participation in this study and treatment with the investigational product.
- Participants must provide consent to the most current version of the ICF during their participation in the study.
- A copy of the ICF(s) must be provided to the participant.
- Participants who are rescreened are required to sign a new ICF. A new participant number will be assigned.

8.1.2. Screening Procedures

Screening is the interval between signing the ICF and the day the participant is randomized in the study (Day 1/Baseline). Screening may not exceed 28 days. Assessments that are required to demonstrate eligibility may be performed over the course of 1 or more days during the screening process. A signed ICF must be obtained as the first step and before IRT screening registration or any other study-specific assessments. The screening of a participant should be registered in the IRT, which will assign the study-specific ID. Each participant who has signed the ICF should be registered in the IRT contemporaneously and without undue delays.

Central laboratory results will be used to determine eligibility unless otherwise stated (see Sections 5.1, 5.2, 8.3.4, and 8.3.5).

Results from the screening visit evaluations will be reviewed to confirm eligibility before the randomization transaction is registered in the IRT and before the administration of study drug. Tests with results that fail eligibility requirements may be repeated once during screening if the investigator believes the results to be in error. For screening assessments that are repeated, the most recent available result before randomization will be used to determine eligibility.

See Sections 5.4 and 5.5 for information regarding screen failures and replacement of participants, respectively.

8.1.3. Interactive Response Technology Procedure

Each participant will be identified in the study by a participant ID number, which is a combination of the 2-letter alpha country International Standards Organization (ISO) code and

then a 3-digit site number, followed by a 3 digit participant number. Site staff should contact the IRT to obtain the participant ID number during screening.

Upon determining that the participant is eligible for randomization, the IRT will be contacted to obtain the treatment assignment. Additionally, the IRT will be contacted at the study visits indicated in Table 3 to update the study drug supply. Additional details will be provided in the IRT Manual.

8.1.4. Distribution of Participant Card

Participants will be provided with a trial participant card at screening. The trial participant card will provide general information on study drug administration, including on study visits when they should not self-administer study drug; remind the participant regarding fasting procedures for predetermined visits (see Section 5.3.2); and contain a telephone number to contact in case of emergency.

8.1.5. Demography and Medical History

8.1.5.1. Demographics and Medical History

Demographic data and general medical history will be collected at screening by the investigator or qualified designee and will include year of birth/age; race; ethnicity; medical and surgical history; family history of cardiovascular disease; family history of clotting disorders; risk factors associated with cardiovascular disease; history of nicotine, alcohol, and medical cannabis (marijuana) use; and current illnesses. Medical history will include relevant medical and surgical treatments that are considered to be clinically significant by the investigator.

History of clinical herpes zoster, herpes zoster vaccination, chickenpox vaccination, and hepatitis B vaccination status will be recorded as part of the medical history.

History of COVID-19 vaccination and COVID-19 infection (and/or hospitalization) will be recorded as part of the medical history.

A detailed medical history with respect to TB risk factors will be documented in the study source documentation. This information will include BCG vaccination, cohabitation with individuals who have had TB, and travel to, residence in, or work in TB endemic locations.

8.1.5.1.1. Justification for Collection of Race and Ethnicity

Recent studies have reported that patients of African or Hispanic descent experience more severe (ie, higher Hurley stage) HS, are more likely to have been previously treated with more effective treatments, have an average higher number of comorbid conditions, and are more likely to have recalcitrant HS (Lee et al 2017, Shao et al 2022). Although the reasons behind these discrepancies are unclear, further studies are required to better understand the link between skin of color and HS severity (Kilgour et al 2021).

With the above considerations in perspective, demographic data, including race and ethnicity, will be collected during the screening period for this study and considered as part of assessments for treatment response heterogeneity.

8.1.5.2. Disease Characteristics and Treatment History

Complete HS medical and treatment history, including date of diagnosis, relevant disease characteristics, and prior treatments, including systemic treatments and surgical procedures, will be collected at screening. A medical or surgical history of other conditions related to HS or relevant to the conduct of this clinical trial will also be collected at screening

Additional information on the procedure for collection of prior HS therapy and surgery information is presented in Section 6.6.1 and Section 6.6.2. Prior use of JAK inhibitor(s) and prior use of immunomodulatory biologic drug(s) for any indication will also be collected, as respectively outlined in Section 6.6.3 and Section 6.6.4.

[redacted]

[redacted]

8.2. Efficacy Assessments

Clinician-reported outcomes and PROs are described in this section.

Clinical evaluations of HS (lesion assessment/count and Hurley staging) will be performed by an experienced and qualified evaluator. The evaluator must receive and document Protocol-specific HS lesion assessment/count and Hurley staging training prior to performing any evaluation in any participant in this study.

To assure consistency and reduce variability, the same evaluator must perform all HS clinical evaluations for any individual participant throughout the study. A backup experienced and qualified, Protocol-trained evaluator will only be allowed and documented in case of emergency or special situations when the designated evaluator is unable to perform the HS clinical evaluations.

The HS lesion assessment/count, Hurley staging, information on lesion rescue, and any other information related to efficacy assessments will be captured in a paper-based study-specific form and entered in the corresponding eCRF. The calculations used for the endpoints described in Section 8.2.1.1 through Section 8.2.1.5 will be performed by the sponsor (or designee).

8.2.1. HS Lesion Assessment and Count

The lesion count is defined as a broad assessment of all the various skin "appearances" that are termed "lesions" in participants with HS. The lesion count must address all relevant anatomical regions in each participant and will be captured at all visits in a paper-based study-specific form and entered in the corresponding eCRF (see Table 3). The lesion count will be used for calculation (by the sponsor or designee) of various efficacy parameters, such as HiSCR, IHS4, and flare incidence.

The nomenclature for HS lesions adapts the glossary proposed by Frew et al (2021) and is outlined in Table 13.

Table 13: HS Lesion Definition

HS Lesion	Definition	Schematic
Abscess	A tender, fluctuant (compressible), palpable lesion, with erythema	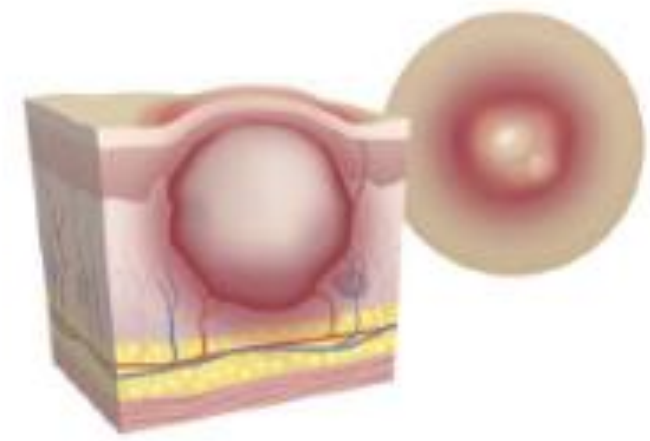
Inflammatory nodule	A solid, spherical, palpable lesion >1 cm; tender and/or erythematous	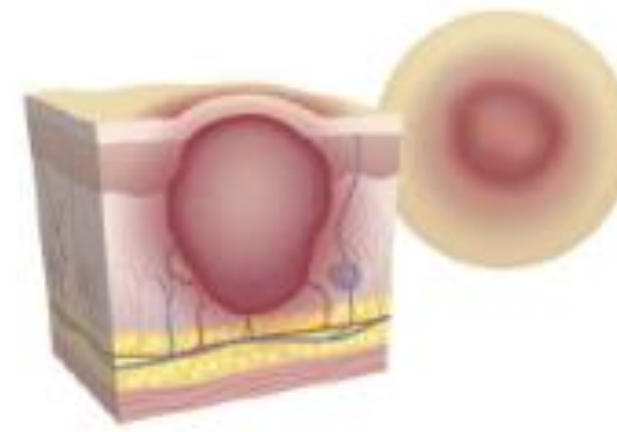
Noninflammatory nodule	Nontender, nonerythematous nodule	
Eroded nodule	A friable nonepithelialized nodule	
Draining tunnel (fistula)	A linear tract that may open onto the skin surface, with drainage expressed at rest or with compression of surrounding structures	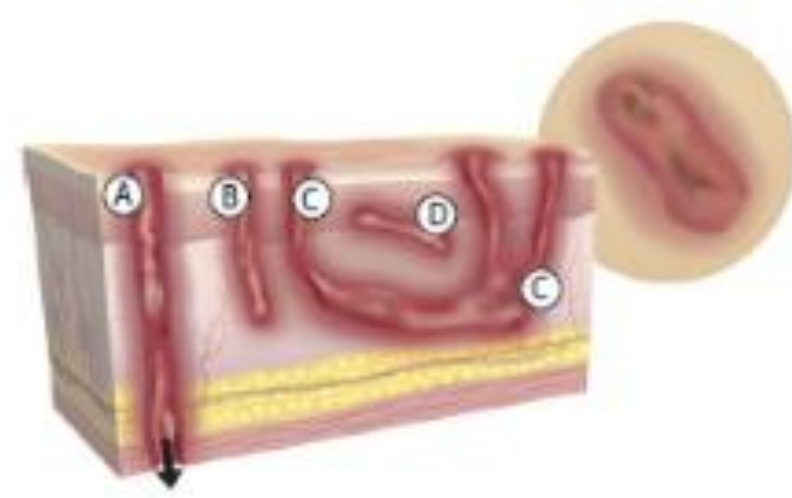
Nondraining tunnel (fistula)	A linear tract that may open onto the skin surface; compression of surrounding structures does not elicit drainage of contents	(A) connection to a hollow organ (eg, bladder, intestine); (B) blind ending; (C) 2 skin-to-skin openings; (D) no opening to the surface
Papule	A solid, palpable lesion ≤1 cm<br>Inflammatory papule: tender and/or erythematous<br>Noninflammatory papule: nontender, nonerythematous	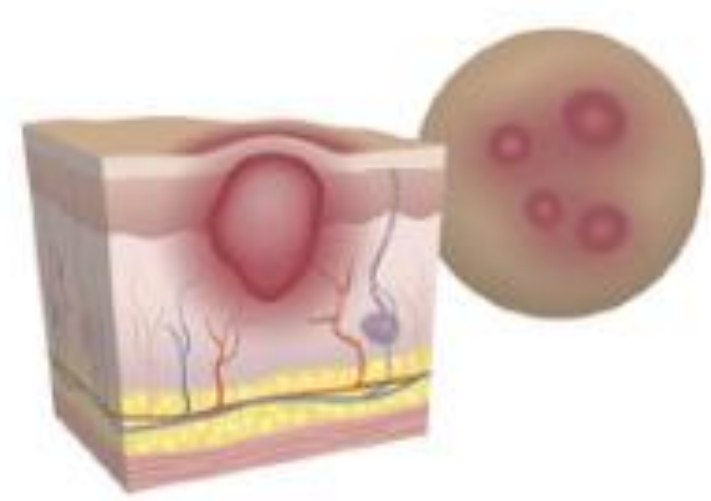
Pustule	A superficial circumscribed lesion that contains opaque fluid (pus) ≤ 1 cm	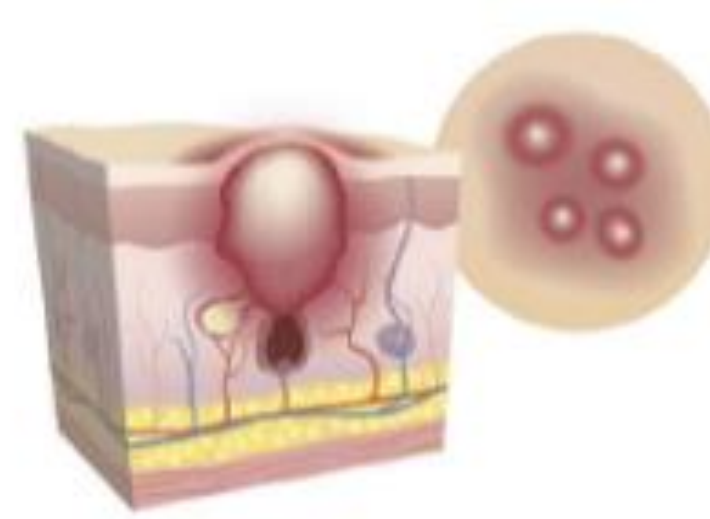

Table 13: HS Lesion Definition (Continued)

HS Lesion	Definition	Schematic
Plaque	A variably shaped, nonspherical, palpable lesion > 1 cm<br>Inflammatory plaque: tender and/or erythematous<br>Noninflammatory plaque: nontender, nonerythematous, may be a type of scar	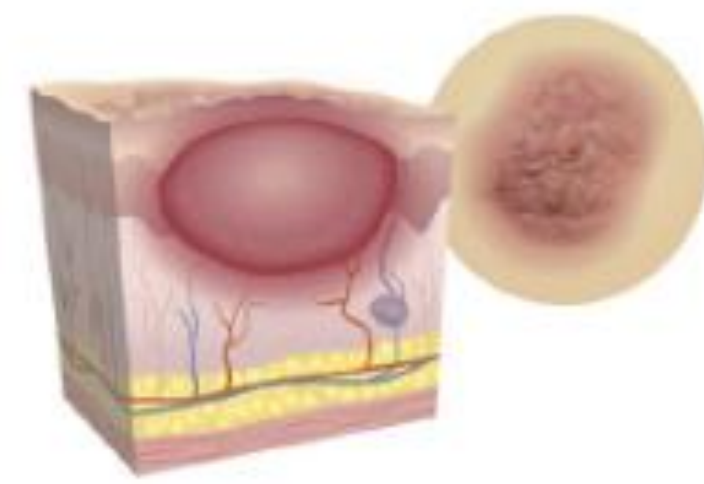
Ulcer	Full-thickness loss of epidermis and at least a portion of the dermis	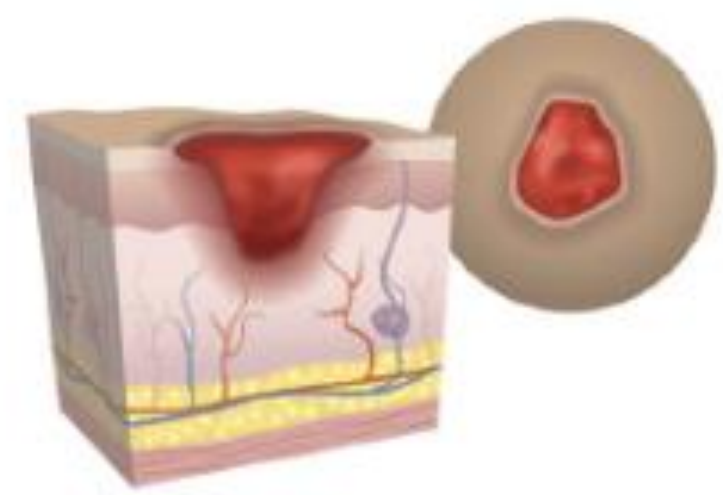
Comedo	Dilated skin opening with keratinous debris<br>Double comedo: interconnected comedones	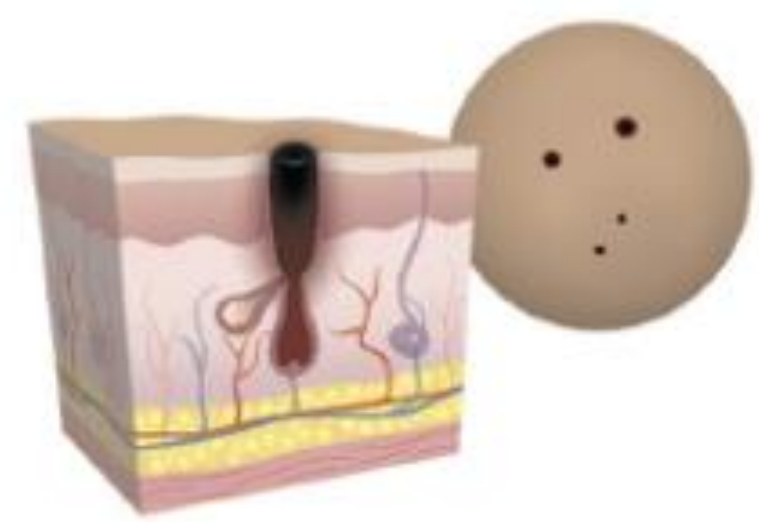

The HS lesions that will be counted in this study are outlined below:

- Abscess
- Inflammatory nodule
- Noninflammatory nodule
- Draining tunnel
- Nondraining tunnel

The distinct anatomical areas (with associated alternate nomenclature, if applicable) that will be considered for lesion assessment and count are outlined below. If lesions are present in an anatomical area(s) not outlined below, they will be recorded under "Other area(s)."

- Face/neck
- Left axilla (armpit)/arm
- Right axilla (armpit)/arm
- Left chest/breast and inframammary area
- Right chest/breast and inframammary area

- Abdomen
- Trunk/torso (back)
- Left buttock (perianal)
- Right buttock (perianal)
- Left inguinocrural fold (groin)/thigh/leg
- Right inguinocrural fold (groin)/thigh/leg
- Genital area
- Other area(s)

8.2.1.1. Hidradenitis Suppurativa Clinical Response

The HiSCR is a validated endpoint that shows responsiveness to improvement in disease activity, simplifies the scoring process, and increases the sensitivity to detect certain HS-specific lesions. HiSCR calculations will be performed by the sponsor (or designee). The HiSCR is defined by the status of 3 types of lesions (Kimball et al 2016c): abscess, inflammatory nodules, and draining tunnels.

The definition of response requires the 3 criteria below to be met, relative to baseline:

- At least 50% decrease in abscess plus inflammatory nodule count; and
- No increase in abscess count; and
- No increase in draining tunnel count

HiSCR75 and HiSCR90 will also be calculated by the sponsor (or designee) as endpoints in this study (see Table 8); their only difference compared to HiSCR is the minimum percentage decrease in total abscess and inflammatory nodule count required to achieve response of 75% and 90%, respectively.

8.2.1.2. Change in HS Lesion Counts

Absolute and relative changes in the counts of the different types of HS lesions will be calculated by the sponsor (or designee) as endpoints in this study (see Table 8).

8.2.1.3. International Hidradenitis Suppurativa Severity Score System

The IHS4 is a composite, dynamic score and validated tool used to determine HS severity. It employs a weighted scale that sums the number of inflammatory nodules (multiplied by 1), the number of abscesses (multiplied by 2), and the number of draining tunnels (multiplied by 4; Zouboulis et al 2017).

Although not used as an inclusion criteria for HS severity in this study (see Hurley stage in Section 8.2.2), the total IHS4 score outlines the following disease categories:

- Mild disease: ≤ 3 points
- Moderate disease: 4 to 10 points
- Severe disease: ≥ 11 points

The use of the IHS4 as a dichotomous endpoint has been proposed recently (IHS4-55), suggesting a performance similar to HiSCR. The definition of response requires at least a 55% decrease from baseline in the total IHS4 score (van Straalen et al 2022a).

IHS4 and related measurements will be calculated by the sponsor (or designee) as endpoints in this study (see Table 8).

8.2.1.4. Flare Incidence

In this study, the definition of flare is outlined below, as proposed by Zouboulis et al (2019):

- At least 25% increase in the total abscess and inflammatory nodule count, relative to baseline; and
- An increase of at least 2 in the total abscess and inflammatory nodule count, relative to baseline

Flare and related flare patterns will be calculated by the sponsor (or designee) as endpoints in this study (see Table 8).

8.2.1.5. Lesion Rescue

In the event of acutely painful lesion(s) that may require intervention, the investigator or designee may perform Protocol-allowed rescue. The frequency and number of lesion rescue treatments will be assessed as endpoints (see Table 8). Rescued lesions will be counted as permanently present (ie, nonresponder lesions), even if they subside over the course of the study.

Specific information on the type and frequency of Protocol-allowed lesion rescue intervention is described in Section 6.6.9.

8.2.2. Hurley Stage

The presence of draining or nondraining tunnels (also known as fistulas/tracts) is the key aspect that sets apart Hurley Stage I from Hurley Stage II/III; tunnels are associated with irreversible tissue damage, which can only be treated with surgery. Therefore, once an anatomical region is identified as Stage II or III, it can never fall below Stage II (Zouboulis et al 2021).

Each anatomical area (as outlined in forms specifically designed for this study) will have their specific Hurley stage captured at visits determined in the SoA (see Table 3). If more than 1 stage is present in the same region, the worst stage in that region should be documented. The participant will be assigned an overall Hurley stage classification corresponding to the stage of the worst involved anatomical region.

The definition of each Hurley stage is outlined in Table 14.

Table 14: Hurley Stages of Hidradenitis Suppurativa

Stage I	Stage II	Stage III
Abscess formation, single or multiple, without tunnels and cicatrization (scarring)	One or more widely separated recurrent abscesses with tunnel (draining or nondraining) formation and cicatrization (scarring)	Multiple interconnected tunnels (draining or nondraining) and abscesses across the entire area, with diffuse or near-diffuse involvement

Source: Jemec 2012.

8.2.3. Photography

Consenting participants at prospectively selected study sites will be asked to have photographs taken of their HS lesions at certain study visits (see Table 3). The cameras for the photographs will be standardized and supplied to the site by a central photography service.

Specific instructions for selection of the anatomical area(s) for photography, as well as for the overall digital image collection, will be detailed via a manual provided by the central photography service and must be followed.

8.2.4. Patient-Reported Outcomes

All PROs will be collected via eCOA. Sites will be provided with training materials and instructions on the use of eCOA and will be responsible to train participants on eDiary (handheld device) and eCOA (tablet device) use. The participant handheld device will be dispensed at the screening visit. Upon completion of participation in the study, the participant should return the device to the site; the device must be checked for any pending data transmissions and deactivated. Further details on use of the devices will be provided in respective eCOA study documentation.

For PROs scheduled to be completed at study visits (see Table 3), questionnaires will be completed by the participant before site personnel perform any assessments to avoid biasing the participant's responses.

8.2.4.1. Daily Diary Patient-Reported Outcomes

8.2.4.1.1. Administrative Procedures

8.2.4.1.1.1. Follow-Up Phone Call

Study sites should contact participants 1 week prior to the baseline visit and 1 week prior to the Week 12 visit to confirm compliance with the daily diary assessments.

8.2.4.1.1.2. Compliance With Daily Diary Patient-Reported Outcomes

Participants' compliance with daily diary PRO assessments (Skin Pain NRS, Analgesic Use, and Itch NRS) should be monitored by the investigator (or designee) throughout study conduct and required follow-up performed with the participant in case of noted noncompliance. Compliance will be evaluated as outlined below:

- Screening period:
  - [redacted]
  - [redacted]
- PC and EXT periods:
  - Participants' compliance with the daily diary assessments should be assessed at each study visit during the PC and EXT periods.
  - Participants who are consistently noncompliant with the daily diary assessments may be withdrawn from the study.

8.2.4.1.2. Skin Pain NRS

Participants will answer the Skin Pain NRS question daily via eDiary from screening through Week 54 based on a 24-hour recall period (see Table 3).

The Skin Pain NRS ranges from 0 (no skin pain) to 10 (skin pain as bad as you can imagine) and evaluates the worst level of skin pain due to the participant's HS.

8.2.4.1.3. Analgesic Use

Participants will answer the analgesic use questionnaire question daily via eDiary from screening through Week 54 based on a 24-hour recall period (see Table 3).

The analgesic use diary will evaluate the use of pain medication (over-the-counter and prescribed) for HS-related pain. The first question will ask if the participant has taken any pain medication (nonopioid or opioid) for HS-related pain in the past 24 hours; if yes, a second question will ask if the medication was an opioid.

Study sites should instruct participants to properly answer the questionnaire based on the generic or commercial (brand) name of analgesics that participants may be familiar with and potentially use during the course of the study. Further information on permitted analgesic therapy is presented in Section 6.6.6.1.

8.2.4.1.4. Itch NRS

Participants will answer the Itch NRS question daily via eDiary from screening through Week 54 based on a 24-hour recall period (see Table 3).

The Itch NRS (Kimball et al 2016a) was developed and validated as a single-item PRO of itch severity that asks patients to rate their itching severity based on their worst level of itching. The scale ranges from 0 (no itch) to 10 (worst itch imaginable).

8.2.4.2. Patient-Reported Outcomes Completed at Study Visits

For PROs scheduled to be completed at study visits per the SoA (see Table 3), questionnaires will be completed by the participant before site personnel perform any clinic assessments to avoid biasing the participant's response.

8.2.4.2.1. Hidradenitis Suppurativa Quality of Life

An assessment of QoL will be performed using the HiSQoL, and participants will complete a questionnaire at the designated study visits listed in Table 3. The HiSQoL is a 17-item HS-specific health-related QoL instrument with a 7-day recall period (Kirby et al 2020), and it will be used to assess HS symptoms and the impact of HS problems on QoL through 3 domains (symptoms, activities and adaptations, and psychosocial).

8.2.4.2.2. Dermatology Life Quality Index

The DLQI is a simple, 10-question validated questionnaire to measure how much the skin problem has affected the participant over the previous 7 days, across symptoms and feelings, daily activities, leisure, work and school, personal relations, and treatment (Finlay and Khan 1994). Participants will complete the DLQI at the study visits designated in Table 3.

8.2.4.2.3. Functional Assessment of Chronic Illness Therapy – Fatigue Scale

Participants will complete the FACIT-F scale at the study visits designated in Table 3. The FACIT-F scale is a 13-item questionnaire originally designed to assess fatigue, over a 7-day recall, in patients with chronic disease. The total FACIT-F score ranges from 0 (maximum fatigue) to 52 (no fatigue). Lower scores are indicative of a greater impact on the participants' QoL (Cella et al 2019).

8.2.4.2.4. Work Productivity and Activity Impairment – Hidradenitis Suppurativa

Participants will complete the WPAI-HS questionnaire at the study visits designated in Table 3. The WPAI-HS questionnaire is an instrument to measure impairments in both paid work and unpaid work. It measures absenteeism and presenteeism as well as impairments in unpaid activity because of HS and other health problems during the past 7 days. The minimum clinically important difference is defined as one-half standard deviation of the total population's baseline score. Absenteeism, presenteeism, and overall work impairment will be assessed only for employed patients (Reilly et al 1993).

[redacted]

[redacted]

8.3. Safety Assessments

Planned timepoints for all safety assessments are provided in the SoA (see Table 3).

See Section 6.5 for guidelines regarding the management of relevant laboratory or other safety assessment abnormalities.

The COVID-19 global pandemic may present challenges to the normal conduct of this study, requiring the outline of potential mitigation strategies described in Appendix E.

8.3.1. Adverse Events

Adverse events will be monitored from the time the participant signs the ICF until at least 30 days after the last dose of study drug. Adverse events for enrolled participants that begin or worsen after informed consent should be recorded on the Adverse Events Form in the eCRF regardless of the assumption of a causal relationship with the study drug. Conditions that were already present at the time of informed consent should be recorded on the Medical History Form in the eCRF. Adverse events (including laboratory abnormalities that constitute AEs) should be described using a diagnosis whenever possible rather than by individual underlying signs and symptoms.

Adverse events will be reported by the participant (or, when appropriate, by a caregiver, surrogate, or the participant's legally authorized representative). The investigator and any qualified designees are responsible for detecting, documenting, and recording events that meet the definition of an AE or SAE and remain responsible for following up on AEs that are serious, that are considered related to the study drug/procedures, or that caused the participant to discontinue the study drug. Care will be taken not to introduce bias when detecting AEs and/or SAEs. Open-ended and nonleading verbal questioning of the participant, such as "How are you feeling?", is the preferred method to inquire about AE occurrences. Adverse events may also be

detected when they are volunteered by the participant during the screening process or between visits or through physical examinations, laboratory tests, or other assessments. The definition, reporting, and recording requirements for AEs are described in Section 9.

If an AE is noted, a targeted physical examination should be conducted as indicated by the symptoms reported by the participant or other findings (eg, laboratory abnormalities). Abnormalities that are considered clinically significant in the judgment of the investigator (or designee) are to be reported as AEs.

All SAEs will be reported to the sponsor or designee within 24 hours, as outlined in Section 9.4. The investigator will submit any updated SAE data to the sponsor within 24 hours of it being available.

After the initial AE/SAE report, the investigator is required to proactively follow each participant at subsequent visits/contacts. All SAEs, and nonserious AEs of special interest (as defined in Section 9.6.1), will be followed until resolution, stabilization, the event is otherwise explained, or the participant is lost to follow-up (as defined in Section 7.3).

8.3.2. Physical Examinations

Physical examinations will be performed as indicated in the SoA (see Table 3).

Height and body weight will be assessed as determined in the SoA (see Table 3).

A comprehensive physical examination, at visits specified in Table 3, will assess the following organ or body systems: skin; head, eyes, ears, nose, and throat; thyroid; lungs; cardiovascular system; abdomen (liver, spleen); extremities; and lymph nodes. A brief neurologic examination will also be performed.

As noted in Section 8.3.1, a targeted physical examination should be conducted as indicated by symptoms reported by the participant, AEs, or other findings. Abnormalities that are considered clinically significant in the judgment of the investigator are to be reported as AEs.

Physical examinations must be performed by a medically qualified individual such as a licensed physician, a physician assistant, or an advanced registered nurse practitioner, as local law permits.

8.3.3. Vital Signs

Vital signs measurements include blood pressure, pulse, respiratory rate, and body temperature and will be obtained as outlined in the SoA (see Table 3). Participant should not smoke or ingest caffeine during the 30 minutes prior to blood pressure and heart rate measurements (see Section 5.3.2 for details).

Vital signs should be collected before blood collection procedures. Alternatively, a minimum interval of 30 minutes between the completion of the blood collection procedures and the beginning of the vital signs collection is recommended. Blood pressure and pulse will be taken with the participant in the recumbent, semirecumbent, or sitting position after 5 minutes of rest.

Abnormal vital sign results identified after the first dose of study drug/treatment constitute an AE if they are considered clinically meaningful, induce clinical signs or symptoms, require concomitant therapy, or require changes in study drug/treatment.

8.3.4. Electrocardiograms

Single 12-lead ECGs will be obtained as outlined in the SoA (see Table 3) using an ECG machine and the following parameters will be recorded in the eCRF: PR, QRS, QT, and QTc intervals. All 12-lead ECGs will be performed with the participant in a recumbent or semirecumbent position after 5 minutes of rest.

The 12-lead ECGs will be interpreted by the investigator or qualified designee for adequate participant management. Additional 12-lead ECGs may be performed as clinically indicated to manage participant safety. Clinically notable abnormalities that are considered clinically significant in the judgment of the investigator are to be reported as AEs.

8.3.5. Laboratory Assessments

See Table 15 for the list of clinical laboratory tests to be performed and Table 3 for the laboratory assessment visit schedule. A central laboratory will perform all clinical laboratory assessments for safety (ie, blood chemistries, hematology assessments, coagulation tests, endocrine function, and urinalysis). The sponsor or designee will perform all PK and translational sample analyses. Additional testing may be required by the investigator or sponsor based on emerging safety data. All Protocol-required laboratory assessments must be conducted in accordance with the Laboratory Manual and Table 3.

Information regarding collection, processing, and shipping of samples for laboratory assessment is provided in the Laboratory Manual.

Unless specified otherwise for selected tests and under the conditions outlined in Section 5.4, if screening laboratory assessments are performed more than 28 days before Day 1, then the tests must be repeated and eligibility confirmed before study drug administration on Day 1.

Clinically significant abnormal laboratory findings are those that are not associated with the underlying disease, unless judged by the investigator to be more severe than expected for the participant's condition. All laboratory tests with values considered clinically significantly abnormal during participation in the study or within 30 (+ 7) days after the last dose of study drug should be repeated until the values return to normal or baseline or are no longer considered clinically significant by the investigator or medical monitor.

Laboratory sample collection on Day 1 must be performed before study drug administration.

See Section 9.1 for information regarding laboratory abnormalities that should be recorded as an AE in the eCRF.

[redacted]

8.3.5.1. Pregnancy Testing

Based on assessment at the screening visit (see guidance in Appendix A), female participants will be determined as WONCBP or WOCBP, and specific testing will be conducted as outlined respectively in Sections 8.3.5.1.1 and 8.3.5.1.2.

8.3.5.1.1. Women of Nonchildbearing Potential

A female who is permanently surgically sterile or postmenopausal (no menses for 12 or more months without an alternative medical cause) is considered to be a WONCBP and is not required to follow contraception recommendations. Further information on the determination of WONCBP status is presented in Appendix A. Confirmation of WONCBP status by serum FSH testing will be conducted at screening for all female participants considered to be permanently surgically sterile or postmenopausal.

- If FSH is not tested, it is assumed that the participant is of childbearing potential and Protocol-specified contraception requirements must be followed.
- If the FSH is tested and the result is consistent with postmenopausal status, contraception requirements are not necessary.
- If the FSH is tested and the result is consistent with premenopausal status, contraception requirements and pregnancy testing requirements for WOCBP must be followed (see Section 8.3.5.1.2).

8.3.5.1.2. Women of Childbearing Potential

A female who does not meet the definition of WONCBP is considered of childbearing potential and is required to practice at least 1 highly effective method of birth control (see Appendix A) from screening through at least 90 days after the last dose of study drug.

If during the course of the study a female participant becomes surgically sterile or postmenopausal, and complete documentation is available, contraceptive measures are no longer required.

A serum pregnancy test will be required for all WOCBP during screening (before the first dose of study drug) and at the safety follow-up visit as indicated in Table 3. Urine pregnancy tests will be performed locally as outlined in Table 3, as medically indicated (eg, in case of loss of menstrual cycle, when pregnancy is suspected), or per country-specific requirement (note that country-required urine pregnancy testing will be outlined and communicated to investigational sites under separate cover). If a urine pregnancy test is positive, the results should be confirmed with a serum pregnancy test.

If the serum pregnancy test is negative after a urine test was positive, the investigator will assess the potential benefit/risk to the participant and determine whether it is in the participant's best interest to resume study drug and continue participation in the study.

If a pregnancy is confirmed by a serum pregnancy test, the participant should be immediately discontinued (see Section 7.1). Additionally, Section 9.8 should be followed for reporting requirements.

8.3.5.2. Tuberculosis Assessment

During the screening period, all participants will be screened for TB infection via central laboratory assessment (see Section 8.3.5.2.1). Confirmed negative status must be obtained before the participant is randomized in the study and receives study drug. There are different outcomes that may be considered:

- If QuantiFERON Gold (or IGRA equivalent) test (and PPD skin test, if applicable) is negative, the participant may be randomized.
- If QuantiFERON Gold (or IGRA equivalent) test (or PPD skin test, if applicable) is positive, the participant must be further evaluated via CXR (or alternative imaging method, such as MRI or CT) for confirmation of TB status (active or latent).
  - Participants with evidence of active TB must not be randomized.
  - Participants with evidence of latent TB must not be randomized, unless a course of at least 2 months of appropriate therapy has been completed prior to randomization.

During the clinical trial, if a participant is experiencing signs or symptoms suspicious for TB, an ad hoc TB test (including CXR or alternative imaging method, such as MRI or CT) may be performed ahead of the next scheduled assessment. The sponsor's medical monitor should be informed of such cases. Confirmed TB infection, after the participant has started the study, should be reported as an AE.

8.3.5.2.1. Tuberculosis Test

- QuantiFERON-TB Gold test (or IGRA equivalent, such as T-SPOT TB test) is a default central laboratory assessment for TB. When required as per local standards, a PPD skin test (also known as tuberculin skin test or Mantoux) can be performed. The same TB testing method should be used throughout the study.

  Note: The PPD skin test should be read by a licensed health care professional between 48 and 72 hours after administration. A participant who does not return within 72 hours will need to be rescheduled for another skin test. The reaction will be measured in millimeters of induration, and induration ≥ 5 mm is considered a positive reaction. The absence of induration will be recorded as "0 mm," not "negative." Participants who have had an ulcerating reaction to the PPD in the past should not be re-exposed and the PPD should be considered positive.
- For regions that require both PPD and QuantiFERON-TB Gold testing (or IGRA equivalent, such as T-SPOT TB test), both will be performed. If either PPD or QuantiFERON-TB Gold (or IGRA equivalent, such as T-SPOT TB test) is positive, the TB test is considered positive.

- If the first QuantiFERON-TB Gold (or IGRA equivalent) test is indeterminate, then the investigator should perform a second QuantiFERON-TB Gold (or IGRA equivalent) test to rule out a positive test result. The second QuantiFERON-TB Gold (or IGRA equivalent) test should be performed centrally; if not possible to be performed centrally, a local laboratory result may be acceptable. If testing remains indeterminate after the second QuantiFERON-TB Gold (or IGRA equivalent) test, then a PPD skin test will be performed.

8.3.5.2.2. Chest X-Ray

Participants can have a CXR (or alternative imaging method, such as MRI or CT) at any time during the study as warranted based on the opinion of the investigator (or designee). The CXR requires posterior-anterior and lateral views. A radiologist or pulmonologist must perform and document an assessment of the CXR (or alternative imaging method). The investigator (or designee) will indicate the clinical significance of any findings and will sign and date the report. In the assessment of the CXR (or alternative imaging method), the investigator (or designee) must indicate the presence or absence of (1) calcified granulomas, (2) pleural scarring/thickening, and (3) signs of active TB. If the CXR (or alternative imaging method) demonstrates changes suggestive of previous TB (eg, calcified nodule, fibrotic scar, apical or basilar pleural thickening) or other findings that are clinically significant, the investigator (or designee) should contact the sponsor's medical monitor (or designee) before randomizing the participant. Participants who have already been randomized in the study but have a CXR (or alternative imaging method) after randomization that is clinically significant for TB must be discontinued.

8.3.5.2.3. Tuberculosis Prophylaxis

At screening, if the participant has evidence of latent TB infection, prophylactic treatment must be initiated at least 2 months prior to administration of the first dose of study drug (or per local guidelines, whichever is longer). Prophylaxis needs to be completed per local guidelines; however, the full course of prophylaxis does not need to be completed prior to the first dose of study drug.

Note: Rifampicin or rifapentine are not allowed for TB prophylaxis. See Section 6.6.8 for a list of other prohibited medications.

Participants with a prior history of latent TB who have documented completion of a full course of anti-TB therapy may be allowed to be randomized in the study, provided nothing has changed in the participant's medical history to warrant repeat treatment. For participants with completion of a full course of anti-TB therapy but insufficient documentation, the investigator should consult with the sponsor's medical monitor (or designee) before randomizing the participant.

During the study, participants with new evidence of TB must be discontinued.

8.3.5.3. HIV Testing

All participants will be tested for the presence of HIV at screening. Participants will be screen failed if they test positive for HIV infection.

8.3.5.4. Hepatitis Testing

All participants will be tested for the presence of HBV and HCV at screening.

8.3.5.4.1. Hepatitis B Virus

Participants must undergo screening for HBV at the study visits indicated in Table 3. At a minimum, this includes testing for HBsAg, HBcAb, and HBsAb.

A diagram is shown in Table 16, with further explanation below.

Table 16: Interpretation and Management of HBV Serologic Test Results

Scenario	Surface Antigen (HBsAg)	Core Antibody (HBcAb)	Surface Antibody (HBsAb)	Status
A	Positive	Negative or positive	Negative or positive	Not eligible
B	Negative	Negative	Negative	Eligible
C	Negative	Negative	Positive	**HBV DNA PCR required**<br>Positive results: not eligible<br>Negative results: eligible
D	Negative	Positive	Positive	
E	Negative	Positive	Negative	

For participants in Scenarios C, D, and E:

- If the HBV DNA test is negative, the participant is eligible and may be randomized.
  - For participants who test HBV DNA negative and are randomized in the study: an HBV DNA PCR test should be repeated approximately every 12 weeks (as outlined in Table 3). HBV DNA PCR testing approximately every 12 weeks after randomization is not necessary for participants in Scenario C with a documented history of HBV vaccination.
- If the HBV DNA test is positive, the participant is NOT eligible and will be screen failed.
- In the event the HBV DNA test cannot be performed, the participant is NOT eligible and will be screen failed.

8.3.5.4.2. Hepatitis C Virus

Blood samples for HCV serology will be obtained at the screening visit and tested. A positive HCVAb will trigger an HCV RNA test (reflex test). A participant will not be eligible and will be screen failed if test results indicate active hepatitis C (HCV RNA detectable in any participant with anti-HCVAb). Participants who test positive for HCV but are successfully treated for HCV infection and have their screening visit at least 12 weeks after the last dose of HCV therapy may be eligible if HCV RNA is undetectable.

[redacted]

8.6. Unscheduled Visits

Unscheduled visits may occur at any time medically warranted. Any assessments performed at those visits should be recorded in the eCRF.

8.7. End of Treatment and Early Termination

The EOT1 coincides with the Week 12 visit, and the EOT2 (EXT period) coincides with the Week 54 visit. A participant who completes the Week 12/EOT1 visit will have completed the PC period and may continue into the EXT period. A participant who completes the Week 54/EOT2 visit will have completed the EXT period.

If a decision is made that the participant will permanently discontinue study drug prior to the Week 54/EOT2 visit, then the ET visit should be conducted and will be determined as ET1 (if during the PC period) or ET2 (if during the EXT period). If the ET visit coincides with a regular study visit, then the ET evaluations will supersede those of that scheduled visit, and the data should be entered in the ET folder in the eCRF. If this decision does not coincide with a regular visit, reasonable efforts should be made to have the participant return to the site to complete the ET procedures.

8.8. Follow-Up

8.8.1. Safety Follow-Up

For participants who do not participate in the separate extension study, the safety follow-up period is the interval between the EOT visit and the scheduled follow-up visit, which should occur 30 (+ 7) days after the EOT visit (or after the last dose of study drug if the EOT visit was not performed). Adverse events and SAEs must be reported up until 1) at least 30 days after the last dose of study drug or 2) until toxicities resolve, return to baseline, or are deemed irreversible, whichever is longer. Reasonable efforts should be made to have the participant return for the follow-up visit and report any AEs that may occur during this period. In exceptional cases when the participant cannot return to the site for the safety follow-up visit, the participant should be contacted by telephone for assessment of AEs and SAEs and the site should properly document the contact.

9. ADVERSE EVENTS: DEFINITIONS AND PROCEDURES FOR RECORDING, EVALUATING, FOLLOW-UP, AND REPORTING

9.1. Definition of Adverse Event

**Adverse Event Definition**
• An AE is any untoward medical occurrence associated with the use of a drug in humans, whether or not it is considered drug-related.<br>• An AE can therefore be any unfavorable or unintended sign (including an abnormal laboratory finding), symptom, or disease (new or exacerbated) temporally associated with the use of study drug.
**Additional Guidance for Events Meeting the Adverse Event Definition**
• Any safety assessments (eg, ECG, vital signs measurements), including those that worsen from baseline, considered clinically significant in the medical and scientific judgment of the investigator (ie, not related to worsening of underlying disease) are to be reported as an AE.<br>• Abnormal laboratory test results are to be reported as an AE if they are considered clinically meaningful, induce clinical signs or symptoms, require concomitant therapy, or require changes in study drug. Whenever possible, a diagnosis (eg, anemia, thrombocytopenia) should be recorded in the eCRF rather than the abnormal laboratory test result (eg, low hemoglobin, platelet count decreased).<br>• Exacerbation of a chronic or intermittent pre-existing condition/disease, including either an increase in the frequency and/or intensity of the condition, is to be reported as an AE.<br>• New conditions detected or diagnosed after the start of study drug administration are to be reported as an AE.<br>• Signs, symptoms, or the clinical sequelae of a suspected drug-drug interaction are to be reported as an AE.<br>• Signs and/or symptoms from dose administration errors of a study drug (eg, overdose) or a concomitant medication are to be reported as an AE.<br>• "Lack of efficacy," "disease worsening," or "failure of expected pharmacological action" will not be reported as an AE or SAE. Such instances will be captured in the efficacy assessments.<br>• A condition that leads to a medical or surgical procedure (eg, endoscopy, appendectomy) will be reported as an AE if it occurs after obtaining informed consent. If the condition is present before entering the study, then it should be captured as medical history.<br>• Pre-existing diseases or conditions with expected fluctuations in signs or symptoms should be reported as an AE only if the investigator judges the fluctuation to have worsened more than expected during study participation.

9.2. Definition of Serious Adverse Event

If an event is not an AE per the definition above, then it cannot be an SAE even if serious conditions are met (eg, hospitalization for signs/symptoms of the disease under study, death due to progression of disease).

**A serious adverse event is defined as any untoward medical occurrence that, at any dose:**
**a. Results in death**
**b. Is life-threatening**<br>The term "life-threatening" in the definition of "serious" refers to an adverse drug experience that places the participant, in the opinion of the initial reporter, at immediate risk of death from the adverse experience as it occurs. This does not include an adverse drug experience that, had it occurred in a more severe form, might have caused death.
**c. Requires inpatient hospitalization or prolongation of existing hospitalization**<br>In general, hospitalization signifies that the participant has been detained (involving at least an overnight stay) at the hospital or emergency department for observation and/or treatment that would not have been appropriate in the physician's office or outpatient setting. Complications that occur during hospitalization are AEs. If a complication prolongs hospitalization or fulfills any other serious criteria, the event is serious. When in doubt as to whether hospitalization occurred or was necessary, the AE should be considered serious.<br>Hospitalization for elective treatment or planned surgery (eg, stent replacement, hip surgery) is not considered an SAE.<br>Hospitalization for medical interventions in which no unfavorable medical occurrence occurred (ie, elective procedures or routine medical visits) is not considered an SAE.
**d. Results in persistent or significant disability/incapacity**<br>The term "disability" means a substantial disruption of a person's ability to conduct normal life functions. This definition is not intended to include experiences of relatively minor medical significance, such as uncomplicated headache, nausea, vomiting, diarrhea, influenza, and accidental trauma (eg, sprained ankle), that may interfere with or prevent everyday life functions but do not constitute a substantial disruption.
**e. Is a congenital anomaly/birth defect**
**f. Is an important medical event**<br>An important medical event is an event that may not result in death, be immediately life-threatening, or require hospitalization but may be considered serious when, based on appropriate medical judgment, the event may jeopardize the participant and may require medical or surgical intervention to prevent one of the outcomes listed in the above definition. Examples of such events include new invasive or malignant cancers; intensive treatment in an emergency department or at home for allergic bronchospasm, blood dyscrasias, or convulsions that do not result in hospitalization; development of drug dependency or drug abuse; or suspected transmission of an infectious agent via a medicinal product. Secondary malignancies should always be considered SAEs.<br>**For Japan:** An event that may lead to disability is also considered an important medical event. It includes a case that is exposed to a risk of dysfunction to an extent that interferes with daily life when the adverse drug reaction occurs. It does not include an adverse drug reaction that, had the reaction been more severe, may have caused disability.

9.3. Recording and Follow-Up of Adverse Events and/or Serious Adverse Events

**Adverse Event and Serious Adverse Event Recording**
• An AE/SAE that begins or worsens after informed consent is signed should be recorded on the Adverse Events Form in the eCRF. All AEs/SAEs should be reported for enrolled participants, but only SAEs need to be reported for screen failure participants. For enrolled participants, conditions that were present at the time informed consent was given should be recorded on the Medical History Form in the eCRF. For detailed information, refer to the eCRF guidelines.<br>• When an AE/SAE occurs, it is the responsibility of the investigator to review all documentation (eg, hospital progress notes, laboratory reports, and diagnostic reports) related to the event.<br>• The investigator (or designee) will then record all relevant AE/SAE information in the eCRF.<br>• It is **not** acceptable for the investigator to send photocopies of the participant's medical records in lieu of completing the Adverse Events Form in the eCRF.<br>• There may be rare instances when copies of medical records for certain cases are requested. In this case, all participant identifiers, with the exception of the participant number, will be redacted by the site staff on the copies of the medical records before submission. These records can be submitted to Incyte Pharmacovigilance by email/fax per the contact information listed in the Study Binder as per SAE completing guidelines.<br>• The investigator will attempt to establish a diagnosis of the event based on signs, symptoms, and/or other clinical information. Whenever possible, the diagnosis (not the individual signs/symptoms) will be documented as the AE/SAE. When a clear diagnosis cannot be identified, each sign or symptom should be reported as a separate AE/SAE.<br>To the extent possible, each AE/SAE should be evaluated to determine the following:<br>• The severity grade (CTCAE v5.0 Grade 1 to 5). See below for further instructions on the assessment of intensity.<br>• Whether there is at least a reasonable possibility that the AE is related to the study drug: suspected (yes) or not suspected (no). See below for further instructions on the assessment of causality.<br>• The start and end dates, unless unresolved at the final safety follow-up visit.<br>• The action taken with regard to study drug as a result of the AE/SAE(s).<br>• The event outcome (eg, not recovered/not resolved, recovered/resolved, recovering/resolving, recovered/resolved with sequelae, fatal, unknown).<br>• The seriousness, as per the SAE definition provided in Section 9.2.<br>• The action taken with regard to the event. Note: If an AE is treated with a concomitant medication or nondrug therapy, this action should be recorded on the Adverse Events Form and the treatment should be specified on the appropriate eCRF (eg, Prior/Concomitant Medications, Procedures and Non-Drug Therapy).

**Assessment of Intensity**
The severity of AEs will be assessed using CTCAE v5.0 Grades 1 through 5. If an event is not classified by CTCAE, the severity of the AE will be graded according to the scale below to estimate the grade of severity.<br>The investigator will make an assessment of intensity for each AE and SAE reported during the study and assign it to 1 of the following categories:<br>• **Grade 1:** Mild; asymptomatic or mild symptoms; clinical or diagnostic observations only; treatment not indicated.<br>• **Grade 2:** Moderate; minimal, local, or noninvasive treatment indicated; limiting age-appropriate activities of daily living.<br>• **Grade 3:** Severe or medically significant but not immediately life-threatening; hospitalization or prolongation of hospitalization indicated; disabling; limiting self-care activities of daily living.<br>• **Grade 4:** Life-threatening consequences; urgent treatment indicated.<br>• **Grade 5:** Fatal.
**Assessment of Causality**
• The investigator is obligated to assess the relationship between study drug and each occurrence of each AE/SAE.<br>• A "reasonable possibility" of a relationship conveys that there are medical facts, evidence, and/or arguments to suggest a causal relationship, rather than that a relationship cannot be ruled out.<br>• The investigator will use clinical judgment to determine the possibility of a relationship.<br>• The investigator will also consult the RSI in the IB or Product Information for study drug or marketed products, respectively, in making their assessment.<br>• Alternative causes, such as underlying or concurrent disease(s), concomitant therapy, and other risk factors, as well as the temporal relationship of the event to study drug administration, will be considered and investigated.<br>• For each AE/SAE, the investigator **must** document in the medical notes that they have reviewed the AE/SAE and have provided an assessment of causality.<br>• With regard to assessing causality of SAEs:<br>– There may be situations in which an SAE has occurred and the investigator has minimal information to include in the initial report. However, the causality assessment is one of the criteria used when determining regulatory reporting requirements. **Therefore, it is very important that the investigator always make an assessment of causality based on the available information for every event before the initial transmission of the SAE.**<br>– The investigator may change their opinion of causality in light of follow-up information and submit the updated causality assessment.

Follow-Up of Adverse Events and Serious Adverse Events
• The investigator is obligated to perform or arrange for the conduct of supplemental measurements and/or evaluations as medically indicated or as requested by the sponsor to elucidate the nature and/or causality of the AE or SAE as fully as possible. This may include additional laboratory tests or investigations, histopathological examinations, or consultation with other health care professionals.
• Once an AE is detected, it should be followed in the Adverse Events Form in the eCRF until it has resolved or until it is judged to be permanent; assessment should be made at each visit (or more frequently if necessary) of any changes in severity, the suspected relationship to the study drug, the interventions required to treat the event, and the outcome.
• When the severity of an AE changes over time for a reporting period (eg, between visits), each change in severity will be reported as a separate AE.
• If a participant dies during participation in the study or during a recognized follow-up period, the investigator will provide the sponsor with a copy of any postmortem findings, including histopathology.
• Updated SAE information will be recorded in the originally completed eCRF and reported to Incyte Pharmacovigilance or its designee as directed in the Study Binder until resolution, stabilization, the event is otherwise explained, or the participant is lost to follow-up.
• Any updated SAE data (including SAEs being downgraded to nonserious) will be submitted to the sponsor (or designee) within 24 hours of receipt of the information.

9.4. Reporting of Serious Adverse Events

Regardless of suspected causality (eg, relationship to study drug or study procedure[s]), all SAEs occurring after the participant has signed the ICF through 30 days after the last dose of study drug must be reported to the sponsor (or designee) immediately, without undue delay but not later than within **24 hours** of obtaining knowledge of its occurrence unless otherwise specified by the Protocol. The investigator will submit any updated SAE data to the sponsor (or designee) immediately, without undue delay but not later than within 24 hours of it being available.
For Japan: This information must also be reported immediately to the head of the study site.

Investigators are not obligated to actively seek SAE information after the safety follow-up visit or more than 30 days after the last dose of study drug. If the investigator learns of any SAE, including death, at any time during this period, and they consider the event to be reasonably related to the study drug or study participation, then the investigator must notify the sponsor (or designee) within 24 hours of becoming aware of the event.

After the initial AE/SAE report, the investigator is required to proactively follow each participant at subsequent visits/contacts. All SAEs and AESIs (as defined in Section 9.6.1) will be followed until resolution, stabilization, the event is otherwise explained, or the participant is lost to follow-up (as defined in Section 7.3).

Prompt notification by the investigator to the sponsor regarding an SAE is essential so that legal obligations and ethical responsibilities for the safety of participants and the safety of a study drug under clinical investigation are met.

If the SAE is not documented in the RSI of the IB for the study drug (new occurrence) and is thought to be related to the study drug, the sponsor or its designee may urgently require further

information from the investigator for expedited reporting to health authorities. The sponsor or its designee may need to issue an Investigator Notification to inform all investigators involved in any study with the same drug that this SAE has been reported. Suspected unexpected serious adverse reactions will be collected and reported to the competent authorities and relevant ethics committees following the sponsor's SOPs and in accordance with Clinical Trials Regulation (EU) No 536/2014, Clinical Trials Information System guidelines, or as per national regulatory requirements in participating countries.

For Japan: The sponsor will report suspected expected deaths and life-threatening events to the PMDA as per local regulatory requirements.

The sponsor has a legal responsibility to notify both the local regulatory authority and other regulatory agencies about the safety of a study drug under clinical investigation. The sponsor will comply with country-specific regulatory requirements relating to safety reporting to the regulatory authority, IRB/IEC, and investigators.

Investigator safety reports must be prepared for suspected unexpected serious adverse reactions according to local regulatory requirements and sponsor policy and forwarded to investigators as necessary.

An investigator who receives an investigator safety report describing an SAE or other specific safety information (eg, summary or listing of SAEs) from the sponsor will review and then file it along with the IB and will notify the IRB/IEC, if appropriate, according to local requirements.

**Serious Adverse Event Reporting**
• Information about all SAEs is collected and recorded on the Adverse Events Form in the eCRF.<br>• The investigator must report within 24 hours of learning of its occurrence any SAE via the EDC system (primary method) or by completing the Serious Adverse Event Report Form in English (only if the EDC system is not available). The contact information for Incyte Pharmacovigilance by email/fax is listed in the Incyte Reference Guide for Completing the Serious Adverse Event Report Form.<br>• In circumstances where the EDC system is not accessible for reporting SAE information (initial and/or follow-up SAE information) to the sponsor within 24 hours, refer to the Incyte Reference Guide for Completing the Serious Adverse Event Report Form. Once the EDC system is functional, the SAE report should be retrospectively added to the EDC system and follow-up should be completed through the EDC. The original copy of the Serious Adverse Event Report Form and the email or facsimile confirmation sheet must be kept at the study site (refer to the Incyte Reference Guide for Completing the Serious Adverse Event Report Form for details and for the email address or fax number).<br>• Follow-up information is also recorded in the eCRF and transmitted to Incyte Pharmacovigilance via the EDC system. The follow-up report should include information that was not provided previously, such as the outcome of the event, treatment provided, action taken with study drug because of the SAE (eg, dose reduced, interrupted, or discontinued), or participant disposition (eg, continued or withdrew from study participation). Each recurrence, complication, or progression of the original event should be reported as follow-up to that event, regardless of when it occurs.<br>• Contacts for SAE reporting can be found in the Study Binder.

9.5. Potential Drug-Induced Liver Injury

[redacted]

Follow the SAE and follow-up reporting requirements per Section 9.2 as potential DILI AEs may be potential SAEs classified in the category of important medical event.

[redacted]

9.7. Emergency Unblinding of Treatment Assignment

In case of a medical emergency, for a participant's safety management, the procedure for emergency unblinding is provided to the investigator in the IRT Manual. The decision to break the treatment code in an emergency situation resides at the sole discretion of the investigator.

If a participant's treatment assignment is unblinded, the sponsor or its designee should be notified immediately, without disclosure of the actual treatment assignment, by telephone for awareness.

If an investigator, the site personnel performing assessments, or a participant is unblinded, then the participant must discontinue study drug unless there are ethical reasons to have the participant remain on the study treatment. In these cases, the investigator must obtain specific approval from the sponsor's (or its designee's) medical monitor for the participant to continue in the study.

9.8. Pregnancy

Pregnancy, in and of itself, is not regarded as an AE unless there is suspicion that the study drug may have interfered with the effectiveness of a contraceptive medication or method. When a pregnancy has been confirmed in a participant during maternal or paternal exposure to study drug, the following procedures should be followed in order to ensure safety:

- The study drug must be discontinued immediately (female participants only).
- The investigator must complete and submit the Incyte Clinical Trial Pregnancy Form to the sponsor or its designee within **24 hours** of learning of the pregnancy.

Data on fetal outcome are collected for regulatory reporting and drug safety evaluations. Follow-up should be conducted for each pregnancy to determine outcome, including spontaneous or voluntary termination, details of the birth, and the presence or absence of any birth defects, congenital abnormalities, or maternal and/or newborn complications, by following until the first well-baby visit. Pregnancy should be recorded on a Clinical Trial Pregnancy Form and reported by the investigator to the sponsor or its designee. Pregnancy follow-up information should be recorded on the same form and should include an assessment of the possible causal relationship to the sponsor's study drug to any pregnancy outcome, as well as follow-up to the first well-baby visit or the duration specified in local regulations, whichever is later. Refer to the Incyte Reference Guide for Completing the Clinical Trial Pregnancy Form in the Study Binder.

Any SAE occurring during the pregnancy of a study participant must be recorded and reported as described in Section 9.4.

Abnormal pregnancy outcomes (eg, spontaneous abortion, fetal death, stillbirth, congenital anomalies, or ectopic pregnancy) are considered SAEs (if occurring in the study participant) and must be reported as described in Section 9.4. If an abnormal pregnancy outcome is reported in a study participant's partner, the event should be reported to the sponsor on the Clinical Trial Pregnancy Form.

9.9. Warnings and Precautions

Special warnings or precautions for the study drug, derived from safety information collected by the sponsor or its designee, are presented in the IB. Additional safety information collected between IB updates will be communicated in the form of Investigator Notifications. Any important new safety information should be discussed with the participant during the study as necessary. If new significant risks are identified, they will be added to the ICF.

9.10. Product Complaints

The sponsor collects product complaints on study drugs and drug delivery systems used in clinical studies in order to ensure the safety of study participants, monitor quality, and facilitate process and product improvements.

All product complaints associated with material packaged, labeled, and released by the sponsor or its designee will be reported to the sponsor. All product complaints associated with other study material will be reported directly to the respective manufacturer.

The investigator or their designee is responsible for reporting a complete description of the product complaint via email or other written communication to the sponsor contact or respective manufacturer as noted in the packaging information. Any AE associated with a product complaint should be recorded as described in Section 9.3.

If the investigator is asked to return the product for investigation, they will return a copy of the product complaint communication with the product.

For Japan: Complaints associated with unapproved medical devices will be reported to the sponsor with a Medical Device Defect Report Form, and the sponsor will report medical device defects to the PMDA as per local regulatory requirements.

9.11. Treatment of Overdose

There has been no clinical experience with overdose of povorcitinib. Treatment of overdose should consist of general supportive measures.

10. STATISTICS

10.1. Sample Size Determination

The primary endpoint of this study is the proportion of participants who achieve HiSCR at Week 12. Participants will be randomized in a 1:1:1 ratio to 1 of 2 doses of povorcitinib (45 or 75 mg QD) or placebo and stratified by ANdT count (< 11 and ≥ 11; approximately ≤ 50% of participants may have an ANdT count < 11) and previous treatment of HS with a biologic drug. Based on the observed HiSCR rates at Weeks 8 and 12 reported in a Phase 2, randomized, dose-ranging study of povorcitinib (INCB 54707-204), it is assumed that the HiSCR rate at Week 12 would be 48% for the povorcitinib 75 mg group, 45% for the povorcitinib 45 mg group, and 30% for the placebo group. Using the chi-square test, without continuity correction, at a 2-sided $\alpha = 0.025$, a sample size of 200 participants per group (with a total of 600 participants) will achieve approximately 93% power to detect a statistically significant difference between the povorcitinib 75 mg group and the placebo group and approximately 80.7% power to detect a statistically significant difference between the povorcitinib 45 mg and the placebo group.

The powers for primary and key secondary endpoints are provided in Table 17.

Table 17: Powers for Primary and Key Secondary Endpoints

Endpoints	Povorcitinib Dose Group	Response Rate for Povorcitinib Dose Group[a]	Response Rate for Placebo Group[a]	Power
Primary endpoint				
Proportion of participants who achieve HiSCR at Week 12	Povorcitinib 75 mg	48%	30%	93%
	Povorcitinib 45 mg	45%	30%	80.7%[b]
Key secondary endpoints				
Proportion of participants who achieve HiSCR75 at Week 12	Povorcitinib 75 mg	38%	20%	96.1%[b]
	Povorcitinib 45 mg	33%	20%	76.2%[b]
Proportion of participants with flare by Week 12	Povorcitinib 75 mg	20%	40%	98.5%[b]
	Povorcitinib 45 mg	25%	40%	83.5%[b]
Proportion of participants achieving ≥ 3-point reduction in Skin Pain NRS at Week 12[c], for regulatory purposes in US/FDA/aligned countries	Povorcitinib 75 mg	22.5%	7.5%	91.3%[d]
	Povorcitinib 45 mg	20%	7.5%	79.6%[d]
Proportion of participants achieving NRS30 at Week 12[e], for regulatory purposes in EU/EMA/aligned countries	Povorcitinib 75 mg	34%	18%	90.3%[b]
	Povorcitinib 45 mg	34%	18%	90.3%[b]

[a] Based on the results from a Phase 2, randomized, dose-ranging study (INCB 54707-204).
[b] Based on the chi-square test at a 2-sided $\alpha = 0.025$.
[c] It is expected that approximately 76% of participants in the ITT population will have Skin Pain NRS score ≥ 3 at baseline based on the results from Study INCB 54707-204.
[d] Based on Fisher exact test at a 2-sided $\alpha = 0.025$.
[e] It is expected that approximately 93% of participants in the ITT population will have Skin Pain NRS score ≥ 1 at baseline based on the results from Study INCB 54707-204.

10.2. Populations for Analysis

The populations for analysis are provided in Table 18.

Table 18: Populations for Analysis

Population	Description
ITT	The ITT population includes all randomized participants. Treatment groups for this population will be defined according to the treatment assignment at randomization.<br>The ITT population will be used for the summary of demographics, baseline characteristics, participant disposition, and analyses of all efficacy data in the PC period.
PP	The PP population includes randomized participants who are considered to be sufficiently compliant with the Protocol. In general, the following are considered important protocol deviations that may significantly affect the primary analysis:<br>• Missing data for the primary endpoint<br>• Overall compliance less than 50% during the PC period<br>Participants with 1 or more such deviations will be excluded from the PP population. In addition, important protocol deviations related to inclusion/exclusion criteria, discontinuation criteria, and use of prohibited concomitant medications will be reviewed. Decisions as to whether any of these deviations warrant exclusion from the PP population will be made prior to database lock and randomization unblinding.<br>The PP population will be used in the supportive sensitivity analysis for the primary estimand.
Safety	The safety population includes all participants who received at least 1 dose of study drug during the PC period. Treatment groups for this population will be determined according to the actual treatment the participant received on Day 1.
Extension evaluable	The extension evaluable population includes all participants who received at least 1 dose of povorcitinib during the extension period.

[redacted]

10.3. Level of Significance

For the primary endpoint and key secondary endpoints, the overall familywise Type I error rate is 2-sided $\alpha = 0.05$.

The following is a list of primary and key secondary null hypotheses to be tested, denoted by $H_{ij,0}$, where i indicates the povorcitinib dose group (i =1 for 75 mg, 2 for 45 mg) and j is the numerical identifier of the endpoint for the dose group (j = 1 for HiSCR at Week 12, j = 2 for HiSCR75 at Week 12, j = 3 for flare by Week 12, j = 4 for Skin Pain Response at Week 12).

Primary null hypotheses:

- $H_{11,0}$: Proportion of participants achieving HiSCR at Week 12 in the povorcitinib 75 mg group is equal to the placebo group
- $H_{21,0}$: Proportion of participants achieving HiSCR at Week 12 in the povorcitinib 45 mg group is equal to the placebo group

Key secondary null hypotheses:

- $H_{12,0}$: Proportion of participants achieving HiSCR75 at Week 12 in the povorcitinib 75 mg group is equal to the placebo group
- $H_{22,0}$: Proportion of participants achieving HiSCR75 at Week 12 in the povorcitinib 45 mg group is equal to the placebo group
- $H_{13,0}$: Proportion of participants with flare by Week 12 in the povorcitinib 75 mg group is equal to the placebo group
- $H_{23,0}$: Proportion of participants with flare by Week 12 in the povorcitinib 45 mg group is equal to the placebo group
- $H_{14,0}$: Proportion of participants achieving Skin Pain Response at Week 12 in the povorcitinib 75 mg group is equal to the placebo group
- $H_{24,0}$: Proportion of participants achieving Skin Pain Response at Week 12 in the povorcitinib 45 mg group is equal to the placebo group

where Skin Pain Response is defined as ≥ 3-point reduction from baseline in Skin Pain NRS for regulatory purposes in US/FDA/aligned countries or Skin Pain NRS30 for regulatory purposes in EU/EMA/aligned countries.

To adjust for multiplicity and provide strong control of overall familywise error rate, the graphic approach (Bretz et al 2011) will be used in the primary endpoint and the key secondary endpoints outlined in Figure 4.

In Step 1, within Family 1, the algorithm begins by testing the null hypotheses for each of the 2 povorcitinib dose groups versus placebo on the primary endpoint ($H_{11,0}$ and $H_{21,0}$) at its assigned significance level of 2-sided $\alpha = 0.025$. If any hypothesis is rejected (ie, deemed significant), then its significance level will be transferred to the subsequent test following the arrow. The numbers along the arrows denote the weights for transferring significance levels. Specifically, a weight of 1 indicates 100% transfer of the significance level. It should be noted it is possible that a hypothesis for a particular endpoint fails to be rejected the first time it is tested but subsequently receives enough significance level from successfully rejected hypotheses and is rejected the second time it is tested. This iterative process of updating the graph and redistribution of the significance level is continued until no further hypotheses can be tested within Family 1.

In Step 2, if all 4 null hypotheses within Family 1 are rejected, the 2 null hypotheses $H_{13,0}$ and $H_{14,0}$ for povorcitinib 75 mg versus placebo within Family 2 will be tested using Hochberg procedure at a significance level of 2-sided $\alpha = 0.05$.

In Step 3, if both null hypotheses $H_{13,0}$ and $H_{14,0}$ within Family 2 are rejected, the 2 null hypotheses $H_{23,0}$ and $H_{24,0}$ for povorcitinib 45 mg versus placebo within Family 3 will be tested using Hochberg procedure at a significance level of 2-sided $\alpha = 0.05$.

Figure 4: Graphic Approach for Multiplicity Adjustment for Testing Primary and Key Secondary Endpoints

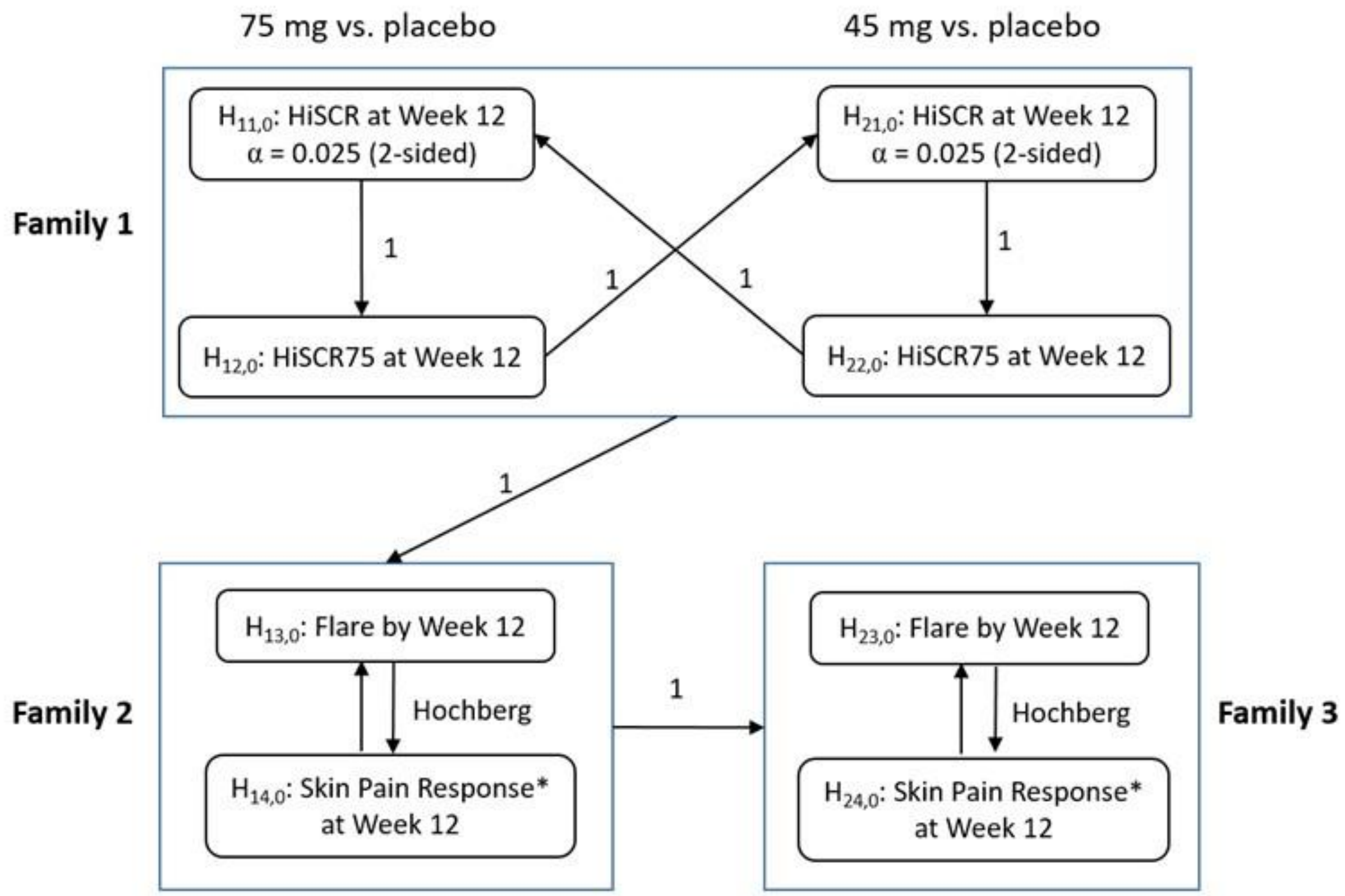

*Skin Pain response defined as ≥ 3-point reduction from baseline in Skin Pain NRS for regulatory purposes in US/FDA/aligned countries, or Skin Pain NRS30 for regulatory purposes in EU/EMA/aligned countries.

Other secondary and exploratory endpoints will be tested using 2-sided $\alpha = 0.05$ but are not considered Type I error–controlled hypotheses.

10.4. Statistical Analyses

Given the design, a 12-week analysis will be performed to access efficacy and safety during the PC, double-blind treatment period. This will occur after all participants have completed the study activities up to Week 12 and all data pertaining to the PC, double-blind period are cleaned.

The final analysis to summarize additional efficacy and safety (without statistical comparison to placebo) will be conducted once the last participant completes the last visit in the study.

The statistical analysis will be described in detail and fully documented in the SAP. The SAP will be finalized prior to the database lock for the 12-week analysis. This section is a summary of the planned statistical analyses of the primary and secondary endpoints.

10.4.1. Primary Analysis

10.4.1.1. Primary Estimand

The primary estimand with the 5 attributes and the strategies for addressing the defined intercurrent events is provided in Table 19. The primary analysis will be based on the ITT population. The primary alternative hypothesis (superiority of each povorcitinib dose group [75 mg, 45 mg] compared with the placebo group) will be tested at a 2-sided $\alpha = 0.025$ level using the CMH test stratified by randomization stratification factors. Stratum-adjusted HiSCR rate difference between each of the povorcitinib dose groups (75 mg, 45 mg) versus placebo and the associated 95% CI will be computed using Mantel-Haenszel weights (Mantel and Haenszel 1959). The stratified CMH p-value and common odds ratio with 95% CI will be provided. The number of participants achieving HiSCR at Week 12 and the respective rates as well as the 2-sided 95% exact CIs based on the Clopper-Pearson method will also be reported by treatment group.

Table 19: Primary Estimand

**Estimand Attribute**	**Definition**
**Treatment**	Each of the povorcitinib dose groups (75 mg, 45 mg) compared with placebo
**Population**	Participants with HS (as defined by trial inclusion/exclusion criteria)
**Variable**	Achievement of HiSCR at Week 12, defined as at least a 50% reduction from baseline in the total abscess and inflammatory nodule count with no increase from baseline in abscess or draining tunnel count at Week 12
**Intercurrent Events**	**Strategies for Addressing Intercurrent Events**
Treatment discontinuation for any reason	Participants who discontinue treatment for any reason at any time before Week 12 are considered to be nonresponders (composite strategy).
Initiation of systemic antibiotic rescue therapy or prohibited medication with effect on HS outcomes (eg, immunomodulatory biologics)	Participants who initiate systemic antibiotic rescue therapy or prohibited medication with effect on HS outcomes at any time before Week 12 are considered to be nonresponders (composite strategy).
Initiation of lesion rescue using Protocol-allowed interventions (injection with intralesional corticosteroid, or incision and drainage of the lesion)	The individual lesions receiving rescue treatment will be counted as permanently present throughout the study (treatment policy).
**Population-level summary**	Proportion of participants achieving HiSCR at Week 12 in each treatment group, the difference in HiSCR rate between each of the povorcitinib dose groups (75 mg, 45 mg) vs placebo.

Based on the composite strategy for handling intercurrent events of treatment discontinuation and initiation of systemic antibiotic rescue therapy or prohibited medication, participants who have any of the intercurrent events are considered as nonresponders for all subsequent visits after the occurrence date of the intercurrent event. As well, participants who miss a postbaseline value at a visit (ie, true missing values due to data not being collected) are imputed as nonresponders on that visit only.

10.4.1.2. Sensitivity Analysis

A sensitivity analysis for the primary estimand will be conducted using the treatment strategy to address the intercurrent events with all data included regardless of occurrence of any intercurrent event, followed by multiple imputation of missing values. A fully conditional specification method (van Buuren 2007) that assumes the existence of a joint distribution of all variables will be used to impute missing postbaseline values for HiSCR components, including abscess count, inflammatory nodule count, and draining tunnel count. For each of the 3 HiSCR components, a regression model including treatment group, randomization stratification factors, and baseline and scheduled postbaseline values up to Week 12 will be specified for the fully conditional specification method. The imputation will be repeated a number of times to generate corresponding complete datasets in order to reflect the uncertainty around the true values. After the missing HiSCR component values are imputed, the binary variables will be derived based on the definition. The stratified CMH test will be applied to each full dataset for the timepoint of interest, and then the results will be combined for the inference using Rubin's rule.

Another sensitivity analysis will be conducted using treatment strategy for intercurrent events following tipping point analysis to examine the potential effects of missing data. The missing HiSCR at Week 12 in each treatment group will be replaced by a range of values from the most conservative scenario to the least conservative scenario. For each of the povorcitinib dose groups (75 mg, 45 mg), the most conservative scenario is that all the missing cases from participants in the povorcitinib dose group are nonresponders and all the missing cases from participants in the placebo group are responders, while the least conservative scenario is the other way around. For each scenario, comparisons between each of the povorcitinib dose groups (75 mg, 45 mg) versus placebo will be performed using the Fisher exact test.

In addition, the primary estimand will be performed using the same stratified CMH test in the PP population as a supportive analysis.

10.4.1.3. Subgroup Analyses

Subgroup analysis for the primary efficacy endpoint will be performed based on the demographics and baseline characteristics, including but not limited to age, geographic region, Hurley stage, and baseline randomization stratification factors (baseline ANdT count; previous treatment of HS with a biologic drug).

10.4.2. Secondary Analyses

10.4.2.1. Key Secondary Estimands

For the key secondary estimands, the 5 attributes and the strategies for addressing the defined intercurrent events are provided in Table 20.

Table 20: Key Secondary Estimands

Estimand Attribute	Definition
**Key secondary estimand for HiSCR75 at Week 12**	
**Treatment**	Same as for primary estimand
**Population**	Same as for primary estimand
**Variable**	Achievement of HiSCR75 at Week 12, defined as at least a 75% reduction from baseline in the total abscess and inflammatory nodule count with no increase from baseline in abscess or draining tunnel count
**Intercurrent Events**	**Strategies for Addressing Intercurrent Events**
Treatment discontinuation for any reason	Same as for primary estimand
Initiation of systemic antibiotic rescue therapy or prohibited medication with effect on HS outcomes (eg, immunomodulatory biologic)	Same as for primary estimand
Initiation of lesion rescue using Protocol-allowed interventions (injection with intralesional corticosteroid, or incision and drainage of the lesion)	Same as for primary estimand
**Population-level summary**	Proportion of participants achieving HiSCR75 at Week 12 in each treatment group, the difference in HiSCR75 rate between each of the povorcitinib dose groups (75 mg, 45 mg) vs placebo
**Key secondary estimand for flare by Week 12**	
**Treatment**	Same as for primary estimand
**Population**	Same as for primary estimand
**Variable**	Having flare at Week 12, defined as at least a 25% increase in the total abscess and inflammatory nodule count with a minimum increase of 2 relative to baseline

Table 20: Key Secondary Estimands (Continued)

Estimand Attribute	Definition
**Intercurrent Events**	**Strategies for Addressing Intercurrent Events**
Treatment discontinuation for any reason	Participants who discontinue treatment for any reason at any time before Week 12 are considered to have a flare (composite strategy).
Initiation of systemic antibiotic rescue therapy or prohibited medication with effect on HS outcomes (eg, immunomodulatory biologic)	Participants who initiate systemic antibiotic rescue therapy or prohibited medication with effect on HS outcomes at any time prior to Week 12 are considered to have a flare (composite strategy)
Initiation of lesion rescue using Protocol-allowed interventions (injection with intralesional corticosteroid, or incision and drainage of the lesion)	Same as for primary estimand
**Population-level summary**	Proportion of participants with flare by Week 12 in each treatment group, the difference between each of the povorcitinib dose groups (75 mg, 45 mg) vs placebo
**Key secondary estimand for Skin Pain Response at Week 12**	
**Treatment**	Same as for primary estimand
**Population**	Same as for primary estimand
**Variable**	Achievement of Skin Pain Response at Week 12, defined as ≥ 3-point reduction from baseline in Skin Pain NRS at Week 12 for regulatory purposes in US/FDA/aligned countries or Skin Pain NRS30 at Week 12 for regulatory purposes in EU/EMA/aligned countries
**Intercurrent Events**	**Strategies for Addressing Intercurrent Events**
Treatment discontinuation for any reason	Same as for primary estimand
Initiation of systemic antibiotic rescue therapy or prohibited medication with effect on HS outcomes (eg, immunomodulatory biologic)	Same as for primary estimand
Initiation of lesion rescue using Protocol-allowed interventions (injection with intralesional corticosteroid, or incision and drainage of the lesion)	Same as for primary estimand
**Population-level summary**	Proportion of participants achieving Skin Pain Response at Week 12 in each treatment group, the difference in response rate between each of the povorcitinib dose groups (75 mg, 45 mg) vs placebo

The key secondary analyses will be conducted based on the ITT population for HiSCR75 at Week 12 and flare by Week 12, the ITT population with baseline Skin Pain NRS score ≥ 3 for ≥ 3-point reduction in Skin Pain NRS at Week 12, and the ITT population with baseline Skin Pain NRS score ≥ 1 for Skin Pain NRS30 at Week 12. All key secondary efficacy endpoints will be analyzed using the stratified CMH method similarly as specified in the primary analysis. Similarly as for the primary analysis, the NRI approach will be used for addressing intercurrent events as well as true missing values due to data not collected.

10.4.2.2. Other Secondary and Exploratory Analyses

In the PC period, all other secondary and exploratory endpoints will be analyzed based on the ITT population.

For categorical endpoints, a similar stratified CMH test as specified in the primary analysis will be used, if applicable. Composite strategy will be used to handle the interrcurrent events followed by the NRI method to impute missing values.

For continuous efficacy endpoints at Week 12, treatment strategy will be used to handle the intercurrent events followed by multiple imputation of the missing values (details described in Section 10.4.1.2). The analysis of covariance with treatment group (povorcitinib 75 mg, povorcitinib 45 mg, and placebo), randomization stratification factors, and baseline measurement will be used to analyze each full dataset, and then the results will be combined for the inference using Rubin's rule. From this analysis, the estimated treatment difference between each of the povorcitinib dose groups (75 mg, 45 mg) and the placebo group and the associated 95% CIs and the p-value will be presented.

As a supportive analysis, the continuous efficacy endpoints may be analyzed using the MMRM to include all available data at postbaseline visits up to Week 12. Participants who have a baseline value and at least 1 postbaseline value in the PC period will be included in the analysis. The MMRM will include the fixed effects of treatment group, randomization stratification factors, visit, treatment by visit interaction, and covariates of baseline measurements and baseline measurements by visit interaction. The variance-covariance matrix of the within-participant errors in the MMRM will be modeled as unstructured. The Kenward-Roger method will be used to estimate the degrees of freedom. Missing values will not be otherwise imputed using the MMRM approach. The least squares means along with 95% CIs and the p-value for the difference in mean change from baseline at each visit between each of the povorcitinib dose groups (75 mg, 45 mg) and the placebo group from the MMRM will be provided.

Long-term efficacy endpoints (across the PC period and the EXT period) will be summarized using descriptive statistics based on the extension evaluable population. For categorical measurements, summary statistics will include sample size, frequency, and percentage. For continuous measurements, summary statistics will include sample size, mean, median, standard deviation, standard error of the mean, minimum, and maximum. Summary statistics for continuous measures will be provided for baseline, the actual measurements at each visit, and the change and percentage change from baseline at each visit, if applicable.

10.4.3. Safety Analyses

Safety analyses will be conducted for the safety population in the PC period, the extension evaluable population in the EXT period, and across both periods.

Adverse events will be coded using MedDRA. A TEAE is defined as any AE either reported for the first time or worsening of a pre-existing event after first dose of study drug until the end of the safety follow-up.

Severity of AEs will be based on the National Cancer Institute CTCAE v5.0 using Grades 1 through 5.

The subset of AEs considered by the investigator to have a causal relationship to study drug will be considered treatment-related AEs. If the investigator does not specify the relationship of the AE to study drug, then the AE will be considered treatment-related.

The number and percentages of participants experiencing TEAEs will be tabulated using the MedDRA system organ class and preferred term for all TEAEs, drug-related TEAEs, serious TEAEs, Grade 3 or higher TEAEs, TEAEs leading to discontinuation of study drug, and AESIs,

Long-term AE rates for each of the povorcitinib dose groups (75 mg, 45 mg) will be analyzed using event rates adjusted by cumulative exposure. This include TEAEs under each povorcitinib dose exposure from participants starting on the povorcitinib dose and participants switching from placebo to the povorcitinib dose.

For clinical laboratory and vital signs, measured values, change from baseline, and percentage change from baseline will be summarized using descriptive statistics. Laboratory test values outside the normal range will be assessed for severity based on the normal ranges for the clinical reference laboratory. The incidence of abnormal laboratory values and shift tables relative to baseline will be tabulated.

[redacted]

10.5. Interim Analysis

No formal interim analysis is planned in this study. However, preplanned analyses of safety will be provided to an external DSMB (see Section 5.6) as specified in the DSMB charter. The process by which the DSMB will review data and make recommendations and decisions will be documented in the DSMB charter.

11. SUPPORTING DOCUMENTATION AND OPERATIONAL CONSIDERATIONS

11.1. Investigator Responsibilities

- The Protocol, Protocol Amendments, ICF, IB, and other relevant documents (eg, advertisements) must be submitted to an IRB/IEC by the investigator and reviewed and approved by the IRB/IEC and health authorities before the study is initiated.
- The investigator is responsible for ensuring that the safety reports provided by the sponsor are reviewed and processed in accordance with regulatory requirements, the policies and procedures established by the IRB/IEC, and institutional requirements.
- Any amendments to the Protocol will require approval from both health authorities and the IRB/IEC before implementation of changes made to the study design, except for changes necessary to eliminate an immediate hazard to study participants.
- The investigator will be responsible for the following:
  - Providing written summaries of the status of the study to the IRB/IEC annually or more frequently in accordance with the requirements, policies, and procedures established by the IRB/IEC.
  - Notifying the IRB/IEC of SAEs or other significant safety findings as required by IRB/IEC procedures.
  - Providing oversight of the conduct of the study at the site and adherence to GCP, IRB/IEC requirements, institutional requirements, and applicable laws and country-specific regulations.
- Adhering to the Protocol as described in this document and agreeing that changes to the Protocol procedures, with the exception of medical emergencies, must be discussed and approved, first, by the sponsor or its designee and, second, by the IRB/IEC. Each investigator is responsible for enrolling participants who have met the specified eligibility criteria.

- Retaining records in accordance with all local, national, and regulatory laws but for a minimum period of at least 2 years after the last marketing application approval in an ICH region and until there are no pending or contemplated marketing applications in an ICH region, or if not approved, 2 years after the termination of the test article for investigation to ensure the availability of study documentation should it become necessary for the sponsor or a regulatory authority to review.
  - The investigator must not destroy any records associated with the study during the retention period without receiving approval from the sponsor. The investigator must notify the sponsor or its designee in the event of accidental loss or destruction of any study records. If the investigator leaves the institution where the study was conducted, the sponsor or its designee must be contacted to arrange alternative record storage options.
  - All eCRF data entered by the site (including audit trail), as well as computer hardware and software (for accessing the data), will be maintained or made available at the site in compliance with applicable record retention regulations. The sponsor will retain the original eCRF data and audit trail.
- **For Japan:** The record retainer (delegated by the head of the study site) will retain the J-GCP–defined essential documentation at this site until the regulatory approval of the study drug or at least 3 years after the discontinuation or completion of the study conduct, whichever is later. If the sponsor requires retention of these documents for a longer period of time, the duration and method of retention will be decided upon through discussion between the sponsor and the study site. It is the responsibility of the sponsor to inform the head of the study site as to when the documents no longer need to be retained.

11.1.1. Identification of the Coordinating Principal Investigator

A coordinating principal investigator has been appointed by the sponsor. As part of her responsibilities, the coordinating principal investigator will review the final CSR. Agreement with the final CSR will be documented by the dated signature of the coordinating principal investigator.

11.2. Data Management

Data management will be performed in a validated EDC system. The investigator will be provided with access to an EDC system so that an eCRF can be completed for each participant.

The site will be provided with eCRF completion guidelines for instructions on data entry in the eCRF. The study monitor will reference the Monitoring Plan in order to ensure that each issue identified is appropriately documented, reported, and resolved in a timely manner in accordance with the plan's requirements. Other data outside the EDC system required in the study conduct of the Protocol, such as documents or results transmitted to the sponsor via a central laboratory or specialized technical vendors and as designated by the sponsor, will have their own data flow management plans, study charters, or biomarker plans, as applicable.

The sponsor (or designee) will be responsible for the following:

- Managing the integrity of the data and the quality of the conduct of the study, such as ensuring that study monitors perform ongoing source data verification to confirm that data entered into the eCRF by authorized site personnel are accurate, complete, and verifiable from source documents; that the safety and rights of participants are being protected; and that the study is being conducted in accordance with the currently approved Protocol and any other study agreements, ICH GCP, and all applicable regulatory requirements.
- Managing and reconciling the data generated and/or collected, including documents and results such as laboratory or imaging data analyzed centrally by a designated vendor of the sponsor.

The investigator will be responsible for the following:

- Recording, or ensuring the recording of, all relevant data relating to the study in the eCRF.
- Delivering, or ensuring the delivery of, all other results, documents, data, know-how, or formulas relating to the study to the sponsor or designee electronically and/or centrally (eg, laboratory data, imaging data, biomarker data, photographs, diary data) or as otherwise specified in the Protocol.
- Maintaining adequate and accurate source documents and trial records that include all pertinent observations on each of the site's trial participants. Source data should be attributable, legible, contemporaneous, original, accurate, and complete. Changes to source data should be traceable, should not obscure the original entry, and should be explained if necessary (eg, via an audit trail). Source data are, in general, all information in original records and certified copies of original records of clinical findings, observations, or other activities in a clinical trial necessary for the reconstruction and evaluation of the trial. Source data are contained in source documents (original records or certified copies).
- Verifying that data entries are accurate and correct by physically or electronically signing the eCRF.
- Maintaining accurate documentation (source data) that supports the information entered in the eCRF, sent to a central vendor designated by the sponsor, or as described in other study and data flow manuals.
  - Source documents provide evidence for the existence of the participant and substantiate the integrity of the data collected. Source documents are filed and available at the investigator's site. Examples of source documents are original documents, data, and records (eg, hospital records; electronic hospital records; clinical and office charts; laboratory notes; memoranda; participants' diaries or evaluation checklists; pharmacy dispensing records; recorded data from automated instruments; copies or transcriptions certified after verification as being accurate copies; microfiches; photographic negatives; microfilm or magnetic media; x-rays; participants' files; and e-records/records kept at the pharmacy, at the laboratories, and at medico-technical departments involved in the clinical trial).

  - Data entered in the eCRF that are transcribed from source documents must be consistent with the source documents or the discrepancies must be explained. The investigator may need to request previous medical records or transfer records, depending on the study. Current applicable medical records must be available.

- Sending participants' data, either as unique samples, copies, or photographs, to be evaluated centrally or analyzed centrally, or both, by a qualified vendor designated by the sponsor.

  - As required by privacy and data protection regulations and Incyte's privacy policies, if any photographs of participants are to be taken, the photographs must be limited to the area of the face or the body that is strictly necessary and the photographs should be masked (ie, identifying features such as eyes, mouth, scars, tattoos, or unique markings or features should be either obscured with a black bar or digitally pixelated so as to not permit the reidentification of the participants and preserve their confidentiality) by a specially designated photography vendor prior to sending the photographs to Incyte or any other third-party vendors for analysis or further processing.

  - **For France:** In accordance with French regulations, sites in France must perform the masking before the photographs are transferred, including to any specially designated photography vendor, Incyte, or any other third-party vendors for analysis or further processing. In addition, the participant's specific consent for photographs shall be collected.

- Permitting study-related monitoring, sponsor audits, IRB/IEC review, and regulatory inspections by providing direct access to source data and other relevant clinical study documents.

  - Monitoring: Qualified representatives of the sponsor or its designee, study monitors, will monitor the study according to a predetermined plan. The investigator must allow the study monitors to review any study materials and participant records at each monitoring visit.

  - Auditing: Qualified representatives of the sponsor or its designee may audit the clinical study site and study data to evaluate compliance with the Protocol, applicable local clinical study regulations, and overall study conduct. The investigator must allow the auditors to review original source records and study documentation for all participants.

  - Regulatory inspection: Regulatory authorities may conduct an inspection of the study and the site at any time during the development of an investigational product. The investigator and staff are expected to cooperate with the inspectors and allow access to all source documents supporting the eCRFs and other study-related documents. The investigator must immediately notify the sponsor when contacted by any regulatory authority for the purposes of conducting an inspection.

11.3. Data Quality Assurance

The sponsor assumes accountability for actions delegated to other individuals (eg, contract research organizations). The sponsor or designee is responsible for the data management of this study, including quality checking of the data. Further, monitoring details describing strategy, including definition of study-critical data items and processes (eg, risk-based initiatives in operations and quality such as risk management and mitigation strategies and analytical risk-based monitoring), methods, responsibilities, and requirements, including handling of noncompliance issues, Protocol deviations, and monitoring techniques (eg, central, remote, or on-site monitoring) are provided in the relevant study plans.

Quality tolerance limits will be predefined in the Risk Assessment Mitigation Plan and Integrated Project Management Plan as per CRO SOPs to identify systematic issues that can impact participants' safety, efficacy results and analysis, and/or reliability of study results. These predefined parameters will be monitored during the study and can be adjusted during the study upon data review. Important deviations from the quality tolerance limits and remedial actions taken, including reporting to IRBs/IECs and health authorities if applicable, will be summarized in the CSR.

11.4. Data Privacy and Confidentiality of Study Records

The investigator and the sponsor or its designee must adhere to applicable data protection laws and regulations. The investigator and the sponsor or its designee are responsible for ensuring that personal information is handled in accordance with local data protection laws (including but not limited to HIPAA and GDPR) as applicable, and the sponsor operates comprehensive data privacy and data security programs that are applicable to this study. Appropriate notice, or notice and consent (as may be required by each applicable jurisdiction), for collection, use, disclosure, and/or transfer (if applicable) of personal information must be obtained in accordance with local data protection laws. Appropriate data protection terms that comply with applicable laws will be included in relevant study agreements.

To ensure confidentiality of records and protect personal data, participant names will not be supplied to the sponsor or its designee. Only the participant number will be recorded in the eCRF; if the participant's name appears on any other document (eg, laboratory report), it must be obliterated on the copy of the document to be supplied to the sponsor or its designee. Study findings stored on a computer will be stored in accordance with appropriate technical and organizational measures as required by local data protection laws.

In the event of a data breach involving participant data, the sponsor or its designee will follow the sponsor's incident response procedures. The precise definition of a data breach varies in accordance with applicable law but may generally be understood as a breach of security leading to the accidental or unlawful destruction, loss, alteration, unauthorized disclosure of, or access to, personal data. In accordance with its incident response procedures, the sponsor will assess the breach to consider its notification and remediation obligations under applicable law.

11.5. Financial Disclosure

Before study initiation, all clinical investigators participating in clinical studies subject to FDA Regulation Title 21 CFR Part 54 – Financial Disclosure by Clinical Investigators (ie, "covered studies") are required to submit a completed Clinical Investigator Financial Disclosure Form that sufficiently details any financial interests and arrangements that apply. For the purpose of this regulation, "clinical investigator" is defined as any investigator or subinvestigator who is directly involved in the treatment or evaluation of research participants, including the spouse and each dependent child of the clinical investigator or subinvestigator. These requirements apply to both US and foreign clinical investigators conducting covered clinical studies.

Any new clinical investigators added to the covered clinical study during its conduct must also submit a completed Clinical Investigator Financial Disclosure Form. During a covered clinical study, any changes to the financial information previously reported by a clinical investigator must be reported to the sponsor or its designee. At the conclusion of the covered clinical study, the clinical investigators will be reminded of their obligations. In the event that the clinical investigator is not reminded, they nevertheless will remain obligated to report to the sponsor or its designee any changes to the financial information previously reported, as well as any changes in their financial information for a period of 1 year after completion of the covered clinical study.

11.6. Publication Policy

By signing the study Protocol, the investigator and their institution agree that the results of the study may be used by the sponsor, Incyte Corporation, for the purposes of national and international registration, publication, and information for medical and pharmaceutical professionals. Study results will be published in accordance with applicable local and national regulations. If necessary, the authorities will be notified of the investigator's name, address, qualifications, and extent of involvement. The terms regarding the publication of study results are contained in the agreement signed with the sponsor or its designee. A signed agreement will be retained by the sponsor or its designee.

The results of this study may be published or presented at scientific meetings. If this is foreseen, the investigator agrees to submit all manuscripts or abstracts to the sponsor before submission. This allows the sponsor to protect proprietary information and to provide comments.

The sponsor will comply with the requirements for publication of study results. In accordance with standard editorial and ethical practice, the sponsor will generally support publication of multicenter studies only in their entirety and not as individual site data. In this case, a coordinating investigator will be designated by mutual agreement.

Authorship will be determined in line with International Committee of Medical Journal Editors authorship requirements.

11.7. Study and Site Closure

The sponsor or designee reserves the right to close the study site or terminate the study at any time for any reason at the sole discretion of the sponsor. Study sites will be closed upon study completion. A study site is considered closed when all required documents and study supplies have been collected and a study-site closure visit has been performed.

For Japan: When the trial is completed, the investigator should inform the head of the study site of the completion in writing and submit a written summary of the trial's outcome, and then the head of the study site should promptly inform the IRB and sponsor or designee of the completion in writing.

The investigator may initiate study-site closure at any time, provided there is reasonable cause and sufficient notice is given in advance of the intended termination.

Reasons for the early closure of a study site by the sponsor or investigator may include but are not limited to:

- Failure of the investigator to comply with the Protocol, the requirements of the IRB/IEC or local health authorities, the sponsor's procedures, or GCP guidelines.
- Inadequate recruitment of participants by the investigator.
- Discontinuation of further study treatment development.

12. REFERENCES

Alavi A, Hamzavi I, Brown K, et al. Janus kinase 1 inhibitor INCB054707 for patients with moderate-to-severe hidradenitis suppurativa: results from two phase II studies. Br J Dermatol 2022;186:803-813.

Amir Ali A, Seng EK, Alavi A, Lowes MA. Exploring changes in placebo treatment arms in hidradenitis suppurativa randomized clinical trials: a systematic review. J Am Acad Dermatol 2020;82:45-53.

Bató A, Brodszky V, Gergely LH, et al. The measurement performance of the EQ-5D-5L versus EQ-5D-3L in patients with hidradenitis suppurativa. Qual Life Res 2021;30:1477-1490.

Bretz F, Posch M, Glimm E, Klinglmueller F, Maurer W, Rohmeyer K. Graphical approaches for multiple comparison procedures using weighted Bonferroni, Simes, or parametric tests. Biom J 2011;53:894-913.

Cella D, Wilson H, Shalhoub H, et al. Content validity and psychometric evaluation of Functional Assessment of Chronic Illness Therapy-Fatigue in patients with psoriatic arthritis. J Patient Rep Outcomes 2019;3:30.

Chen SX, Greif C, Gibson RS, Porter ML, Kimball AB. Advances in biologic and small molecule therapies for hidradenitis suppurativa. Exp Opin Pharmacother 2022;23:959-978.

Chu C-B, Yang C-C, Tsai S-J. Global data analysis supports smoking as the fundamental element associated with geographical sex disparities in hidradenitis suppurativa. Br J Dermatol 2021;185:1054-1056.

Clinical Trials Facilitation and Coordination Group. Recommendations related to contraception and pregnancy testing in clinical trials. 2020.

Fernandez JM, Rizvi OH, Marr KD, et al. Itch and pain by lesion morphology in hidradenitis suppurativa patients. Int J Dermatol 2021;60:56-59.

Food and Drug Administration. FDA Drug Safety Communication: FDA requires warnings about increased risk of serious heart-related events, cancer, blood clots, and death for JAK inhibitors that treat certain chronic inflammatory conditions. 2021.

Finlay AY, Khan GK. Dermatology Life Quality Index (DLQI) — a simple practical measure for routine clinical use. Clin Exp Dermatol 1994;19:210-216.

Frew JW, Hawkes JE, Krueger JG, Topical, systemic and biologic therapies in hidradenitis suppurativa: pathogenic insights by examining therapeutic mechanisms. Ther Adv Chronic Dis 2019;10:1-24.

Frew JW, Jiang CS, Singh N, et al. Clinical response rates, placebo response rates, and significantly associated covariates are dependent on choice of outcome measure in hidradenitis suppurativa: a post hoc analysis of PIONEER 1 and 2 individual patient data. J Am Acad Dermatol 2020;82:1150-1157.

Frew JW, Lowes MA, Goldfarb N, et al. Global harmonization of morphological definitions in hidradenitis suppurativa for a proposed glossary. JAMA Dermatol 2021;157:449-455.

Hendricks AJ, Hsiao JL, Lowes MA, Shi VY. A comparison of international management guidelines for hidradenitis suppurativa. Dermatology 2021;237:81-96.

Herdman M, Gudex C, Lloyd A, et al. Development and preliminary testing of the new five-level version of EQ-5D (EQ-5D-5L). Qual Life Res 2011;20:1727-1736.

INCB054707 Investigator's Brochure. Wilmington, DE: Incyte Corporation.

Ingram JR. The epidemiology of hidradenitis suppurativa. Br J Dermatol 2020;183:990-998.

Jemec GB. Hidradenitis suppurativa. N Engl J Med 2012;366:158-164.

Kilgour JM, Li S, Sarin KY. Hidradenitis suppurativa in patients of color is associated with increased disease severity and healthcare utilization: A retrospective analysis of 2 U.S. cohorts. JAAD Int 2021;3:42-52.

Kimball AB, Naegeli AN, Edson-Heredia E, et al. Psychometric properties of the Itch Numeric Rating Scale in patients with moderate-to-severe plaque psoriasis. Br J Dermatol 2016a;175:157-162.

Kimball AB, Okun MM, Williams DA, et al. Two phase 3 trials of adalimumab for hidradenitis suppurativa. N Engl J Med 2016b;375:422-434.

Kimball AB, Sobell JM, Zouboulis CC, et al. HiSCR (Hidradenitis Suppurativa Clinical Response): a novel clinical endpoint to evaluate therapeutic outcomes in patients with hidradenitis suppurativa from the placebo-controlled portion of a phase 2 adalimumab study. J Eur Acad Dermatol Venereol 2016c;30:989-994.

Kirby JS, Thorlacius L, Villumsen B, et al. The Hidradenitis Suppurativa Quality of Life (HiSQOL) score: development and validation of a measure for clinical trials. Br J Dermatol 2020;183:340-348.

Lee DE, Clark AK, Shi VY. Hidradenitis suppurativa: disease burden and etiology in skin of color. Dermatology 2017;233:1-6.

Mantel N, Haenszel W. Statistical aspects of analysis of data from retrospective studies of disease. J Natl Cancer Inst 1959;22:719-748.

Matusiak L, Bieniek A, Szepietowski JC. Psychophysical aspects of hidradenitis suppurativa. Acta Derm Venereol 2010;90:264-268.

Matusiak Ł. Profound consequences of hidradenitis suppurativa: a review. Br J Dermatol 2020;183;e171-e177.

Millum J, Grady C. The ethics of placebo-controlled trials: methodological justifications. Contemp Clin Trials 2013;36:510-514.

National Institutes of Health. COVID-19 Treatment Guidelines. Coronavirus Disease 2019 (COVID-19) Treatment Guidelines. Accessed April 7, 2023. https://www.covid19treatmentguidelines.nih.gov

Nguyen TV, Damiani G, Orenstein LAV, Hamzavi I, Jemec GB. Hidradenitis suppurativa: an update on epidemiology, phenotypes, diagnosis, pathogenesis, comorbidities and quality of life. J Eur Acad Dermatol Venereol 2021;35:50-61.

Reilly MC, Zbrozek AS, Dukes EM. The validity and reproducibility of a work productivity and activity impairment instrument. Pharmacoeconomics 1993;4:353-365.

Ring HC, Maul JT, Yao Y, et al. Drug survival of biologics in patients with hidradenitis suppurativa. JAMA Dermatol 2022;158:184-188.

Rumberger BE, Boarder EL, Owens SL, Howell MD. Transcriptomic analysis of hidradenitis suppurativa skin suggests roles for multiple inflammatory pathways in disease pathogenesis. Inflamm Res 2020;69:967-973.

Sabat R, Jemec GBE, Matusiak Ł, Kimball AB, Prens E, Wolk K. Hidradenitis suppurativa. Nat Rev Dis Primers 2020;6:18.

Savage KT, Singh V, Patel ZS, et al. Pain management in hidradenitis suppurativa and a proposed treatment algorithm. J Am Acad Dermatol 2021;85:187-199.

Shao K, Hooper J, Feng H. Racial and ethnic health disparities in dermatology in the United States. Part 2: Disease-specific epidemiology, characteristics, management, and outcomes. J Am Acad Dermatol 2022;87:733-744.

Torres PA, Helmstetter JA, Kaye AM, Kaye AD. Rhabdomyolysis: pathogenesis, diagnosis, and treatment. Ochsner J 2015;15:58-69.

Van Buuren S. Multiple imputation of discrete and continuous data by fully conditional specification. Stat Methods Med Res 2007;16:219-242.

Van Straalen KR, Ingram JR, Augustin M, Zouboulis CC. New treatments and new assessment instruments for hidradenitis suppurativa. Exp Dermatol 2022a;31(suppl 1):33-39.

Van Straalen KR, Prens EP, Gudjonsson JE. Insights into hidradenitis suppurativa. J Allergy Clin Immunol 2022b;149:1150-1161.

Vossen ARJV, Schoenmakers A, van Straalen KR, Prens EP, van der Zee HH. Assessing pruritus in hidradenitis suppurativa: a cross-sectional study. Am J Clin Dermatol 2017;18:687-695.

Yao Y, Jørgensen AR, Thomsen SF. Work productivity and activity impairment in patients with hidradenitis suppurativa: a cross-sectional study. Int J Dermatol 2020;59:333-340.

Zouboulis CC, Benhadou F, Byrd AS, et al. What causes hidradenitis suppurativa? — 15 years after. Exp Dermatol 2020a;29:1154-1170.

Zouboulis CC, Frew JW, Giamarellos-Bourboulis EJ, et al. Target molecules for future hidradenitis suppurativa treatment. Exp Dermatol 2021;30(suppl 1):8-17.

Zouboulis CC, Gulliver W, Ingram J, et al. Endpoints of clinical trials for hidradenitis suppurativa: proceedings of a round-table session. Exp Dermatol 2020b;29(suppl 1):67-72.

Zouboulis CC, Okun MM, Jemec GBE, et al. Long-term adalimumab efficacy in patients with moderate-to-severe hidradenitis suppurativa/acne inversa: 3-year results of a phase 3 open-label extension study. J Am Acad Dermatol 2019;80:60-69.

Zouboulis CC, Tzellos T, Kyrgidis A, et al. Development and validation of the International Hidradenitis Suppurativa Severity Score System (IHS4), a novel dynamic scoring system to assess HS severity. Br J Dermatol 2017;177:1401-1409.

Zouboulis VA, Zouboulis KC, Zouboulis CC. Hidradenitis suppurativa and comorbid disorder biomarkers, druggable genes, new drugs and drug repurposing — a molecular meta-analysis. Pharmaceutics 2022;14:44.

APPENDIX A. INFORMATION REGARDING EFFECTIVENESS OF CONTRACEPTIVE METHODS AND DEFINITIONS

Definitions

WOCBP: A woman who is considered fertile following menarche and until becoming postmenopausal unless permanently sterile (see below).

Women in the following categories are not considered WOCBP:

- Premenarchal
- Premenopausal with 1 of the following:[a]
  - Documented hysterectomy
  - Documented bilateral salpingectomy
  - Documented bilateral oophorectomy
- Postmenopausal is defined as:
  - Participants with no menses for 12 or more months without an alternative medical cause AND a serum FSH level > 40 IU/L.
  - Female participants on HRT and whose menopausal status is in doubt will be required to use 1 of the nonhormonal, highly effective contraception methods if they wish to continue their HRT during the study. Otherwise, they must discontinue HRT to allow confirmation of postmenopausal status before study enrollment.

For male participants of reproductive potential[b]

The following methods during the Protocol-defined timeframe in Section 5.1 are highly effective:

- Use of a male condom plus partner use of an additional contraceptive method when having penile-vaginal intercourse with a WOCBP who is not currently pregnant
- Vasectomy with medical assessment of the surgical success (verified by site personnel's review of the participant's medical records)
- Permanently sterile (verified by site personnel's review of the participant's medical records)
- Sexual abstinence[c]
  - Be abstinent from penile-vaginal intercourse as their usual and preferred lifestyle (abstinent on a long-term and persistent basis) and agree to remain abstinent (sexual abstinence is not approved in Japan).

The following are **not** acceptable methods of contraception:

- Periodic abstinence (calendar, symptothermal, postovulation methods), withdrawal (coitus interruptus), spermicides only, and lactational amenorrhea method
- Male condom with cap, diaphragm, or sponge with spermicide
- Male and female condom used together

Note: Men with a pregnant or breastfeeding partner must agree to remain abstinent from penile-vaginal intercourse or use a male condom during each episode of penile penetration (sexual abstinence is not approved in Japan).

For female participants who are WOCBP
The following methods during the Protocol-defined timeframe in Section 5.1 that can achieve a failure rate of less than 1% per year when used consistently and correctly are considered as highly effective birth control methods:<br>• Combined (estrogen and progestogen containing) hormonal contraception associated with inhibition of ovulation[d]<br>– oral<br>– intravaginal (administration route is not approved in Japan)<br>– transdermal (administration route is not approved in Japan)<br>• Progestogen-only hormonal contraception associated with inhibition of ovulation[d] (progesterone-only hormonal contraception is not approved in Japan, so this bullet and its sub-bullets will not apply for Japan)<br>– oral<br>– injectable<br>– implantable[e]<br>• Intrauterine device[e]<br>• Intrauterine hormone-releasing system[e]<br>• Bilateral tubal occlusion[e]<br>• Vasectomized or permanently sterile partner[e,f]<br>• Sexual abstinence[c] (sexual abstinence is not approved in Japan)

[a] Documentation can come from the site personnel's review of the participant's medical records, medical examination, or medical history interview.
[b] If the male participant has a partner of childbearing potential, the partner should also use contraceptives.
[c] In the context of this guidance, sexual abstinence is considered a highly effective method only if defined as refraining from heterosexual intercourse during the entire period of risk associated with the study treatment. The reliability of sexual abstinence needs to be evaluated in relation to the duration of the clinical study and the preferred and usual lifestyle of the participant.
[d] It is unknown if hormonal contraception may be susceptible to interaction with the investigational medicinal product, which may reduce the efficacy of the contraception method. Therefore, 2 methods of contraception should be used.
[e] Contraception methods that in the context of this guidance are considered to have low user dependency.
[f] Vasectomized or permanently sterile partner is a highly effective method of avoiding pregnancy provided that partner is the sole sexual partner of the WOCBP study participant and that the partner has received medical assessment of sterility.
Source: Clinical Trials Facilitation and Coordination Group 2020.

APPENDIX B. LIST OF STRONG SYSTEMIC CYP3A4 INHIBITORS AND STRONG/MODERATE SYSTEMIC CYP3A4 INDUCERS

Strong Systemic CYP3A4 Inhibitors	Strong and Moderate Systemic CYP3A4 Inducers
• Ceritinib<br>• Clarithromycin<br>• Cobicistat<br>• Danoprevir<br>• Dasabuvir<br>• Elvitegravir<br>• Idelalisib<br>• Indinavir<br>• Itraconazole<br>• Ketoconazole<br>• Lopinavir<br>• Nefazodone<br>• Nelfinavir<br>• Ombitasvir<br>• Paritaprevir<br>• Posaconazole<br>• Ritonavir[a]<br>• Saquinavir<br>• Telithromycin<br>• Tipranavir<br>• Troleandomycin<br>• Voriconazole<br><br>• Grapefruit/grapefruit juice<br>• Seville oranges	• Apalutamide<br>• Bosentan<br>• Carbamazepine<br>• Cenobamate<br>• Dabrafenib<br>• Efavirenz<br>• Enzalutamide<br>• Etravirine<br>• Ivosidenib<br>• Lorlatinib<br>• Lumacaftor<br>• Mitotane<br>• Pexidartinib<br>• Phenobarbital<br>• Phenytoin<br>• Primidone<br>• Rifampicin/rifampin<br>• Rifapentine<br>• Sotorasib<br><br>• St John's wort

Note: Updated lists of CYP3A4 inhibitors/inducers can be found at Food and Drug Administration. Drug Development and Drug Interactions: Table of Substrates, Inhibitors and Inducers. Available at: https://www.fda.gov/drugs/drug-interactions-labeling/drug-development-and-drug-interactions-table-substrates-inhibitors-and-inducers.

[a] Paxlovid™ is a coadministration of nirmatrelvir and ritonavir.

[redacted]

[redacted]

[redacted]

[redacted]

[redacted]

[redacted]

[redacted]

[redacted]

APPENDIX F. PROTOCOL AMENDMENT SUMMARY OF CHANGES

Document	Date
Amendment 1	10 APR 2023
Amendment 2	01 MAY 2023

Amendment 2 (01 MAY 2023)

Overall Rationale for the Amendment:

The primary purpose of the amendment is to address regulatory requests provided after review by the EU national health authorities. This amendment is applicable to the following countries: Bulgaria, Denmark, France, Italy, Germany, Poland, and Spain.

1. **Section 1, Protocol Summary; Section 4.1, Overall Design; Section 4.2, Overall Study Duration; Section 6.7, Treatment After the End of the Study; Section 8.8.1, Safety Follow-up**

   Description of change: Clarified aspects related to poststudy access to study drug.

   Rationale for change: Regulatory request.

2. **Section 2.4, Benefit/Risk Assessment (Table 7: Risk-Mitigation Assessment for Povorcitinib)**

   Description of change: Included statement regarding the benefits and risks of participating in this study for potential participants who are current or past long-time smokers.

   Rationale for change: Regulatory request.

3. **Section 6.5.1, Criteria and Procedures for Dose Interruption and Discontinuation of Study Drug (Table 11: Guidelines for Interruption, Restarting, and Discontinuation of Study Drug)**

   Description of change: Revised guidance to describe management of CPK elevations.

   Rationale for change: Regulatory request.

4. **Section 8.1.1, Informed Consent Process**

   Description of change: Included statement explaining that the ICF will describe the potential risks associated with participation in this study and treatment with the investigational product for participants who are current or past long-time smokers.

   Rationale for change: Regulatory request.

5. **Incorporation of administrative changes.** Other administrative changes have been incorporated throughout the Protocol and are noted in the redline version of the amendment.

Amendment 1 (10 APR 2023)

Overall Rationale for the Amendment:

The primary purpose of the amendment is to address regulatory requests provided after review by the EU national health authorities. This amendment is applicable to the following countries: Bulgaria, Denmark, France, Italy, Germany, Poland, and Spain.

1. **Title Page**

   Description of change: Revised the binding statement that the clinical trial will be conducted in compliance with Clinical Trials Regulation (EU) No. 536/2014.

   Rationale for change: Regulatory request.

2. **Section 2.4, Benefit/Risk Assessment (Table 7: Risk-Mitigation Assessment for Povorcitinib); Section 5.2, Exclusion Criteria (Exclusion Criterion 3g)**

   Description of change: Removed "or cervical carcinoma in situ."

   Rationale for change: Regulatory request.

3. **Section 6.5.1, Criteria and Procedures for Dose Interruption and Discontinuation of Study Drug (Table 11: Guidelines for Interruption, Restarting, and Discontinuation of Study Drug)**

   Description of change: Revised guidance to describe management of ALT/AST elevations.

   Rationale for change: Regulatory request.

4. **Section 6.5.1.3, Management of COVID-19**

   Description of change: Revised guidance regarding COVID-19 management.

   Rationale for change: Regulatory request.

5. **Section 6.5.1.5, Management of Malignancy**

   Description of change: Revised to include any malignancy.

   Rationale for change: Regulatory request.

6. **Section 6.6.10, Systemic Antibiotic Rescue Therapy**

   Description of change: Clarified rationale on why rifampin/rifampicin is prohibited for systemic antibiotic rescue therapy.

   Rationale for change: Regulatory request.

7. **Section 6.7, Treatment After the End of the Study**

   Description of change: Clarified aspects related to poststudy access to study drug.

   Rationale for change: Regulatory request.

8. **Section 9.4, Reporting of Serious Adverse Events**

   Description of change: Updated directive reference.

   Rationale for change: Regulatory request.

9. **Incorporation of administrative changes.** Other administrative changes have been incorporated throughout the Protocol and are noted in the redline version of the amendment.

Signature Page for VV-CLIN-021438 v4.0

Approval Task	[redacted]<br>Approver<br>[redacted]<br>01-May-2023 19:43:16 GMT+0000
Approval Task	[redacted]<br>Approver<br>[redacted]<br>01-May-2023 19:45:46 GMT+0000
Approval Task	[redacted]<br>[redacted]<br>01-May-2023 19:46:00 GMT+0000
Approval Task	[redacted]<br>Approver<br>[redacted]<br>01-May-2023 19:53:24 GMT+0000
Approval Task	[redacted]<br>[redacted]<br>01-May-2023 19:59:09 GMT+0000

Signature Page for VV-CLIN-021438 v4.0

Statistical Analysis Plan

INCB 54707-301/302

Selective **T**reatment of **O**ral **P**ovorcitinib in **H**idradenitis **S**uppurativa Study **1/2** (**STOP-HS1/2**)

A Phase 3, Double-Blind, Randomized, Placebo-Controlled, Efficacy and Safety Study of Povorcitinib (INCB054707) in Participants With Moderate to Severe Hidradenitis Suppurativa

**IND Number:**	[redacted]
**EU CT Number:**	**2022-501752-29-00 and 2022-501753-36-00**
**Sponsor:**	**Incyte Corporation**<br>**1801 Augustine Cut-Off**<br>**Wilmington, DE 19803 USA**
**Protocol Version:**	**INCB 54707-301 Protocol Amendment 2 dated 01 MAY 2023**<br>**INCB 54707-302 Protocol Amendment 2 dated 01 MAY 2023**
**CRF Approval Date:**	**INCB 54707-301 and INCB 54707-302: 05 FEB 2024**
**SAP Version:**	**Original**
**SAP Author:**	[redacted]<br>**Executive Director, Biostatistics**
**Date of Plan:**	**17 DEC 2024**

This study is being conducted in compliance with Good Clinical Practice, including the archiving of essential documents.

TABLE OF CONTENTS

TITLE PAGE....1
TABLE OF CONTENTS....2
LIST OF ABBREVIATIONS....6
1. INTRODUCTION....8
2. STUDY INFORMATION, OBJECTIVES, AND ENDPOINTS....9
2.1. Protocol and Case Report Form Version....9
2.2. Study Objectives and Endpoints....9
3. STUDY DESIGN....15
3.1. Randomization....16
3.2. Control of Type I Error....16
3.3. Sample Size Considerations....18
3.4. Schedule of Assessments....19
4. DATA HANDLING DEFINITIONS AND CONVENTIONS....20
4.1. Scheduled Study Evaluations and Study Periods....20
4.1.1. Day 1....20
4.1.2. Study Day....20
4.1.3. Baseline Value....20
4.1.4. Handling of Missing and Incomplete Dates....21
4.2. Variable Definitions....22
4.2.1. Body Mass Index....22
4.2.2. Prior and Concomitant Medication....22
5. STATISTICAL METHODOLOGY....23
5.1. General Methodology....23
5.2. Treatment Groups....23
5.3. Analysis Populations....23
5.3.1. All-Screened Population....23
5.3.2. Intent-to-Treat Population....24
5.3.3. Per Protocol Population....24
5.3.4. Safety Population....24
5.3.5. Extension-Evaluable Population....24
6. BASELINE, EXPOSURE, AND DISPOSITION....25

6.1. Demographics, Baseline Characteristics, and Disease History 25
6.1.1. Demographics and Baseline Characteristics 25
6.1.2. Baseline Disease Characteristics 25
6.1.3. Disease History 26
6.1.4. Medical History 26
6.2. Disposition of Participants 27
6.3. Protocol Deviations 27
6.4. Exposure 27
6.5. Study Drug Compliance 28
6.6. Prior and Concomitant Medication 28
7. EFFICACY 29
7.1. General Considerations 29
7.2. Efficacy Parameters 29
7.2.1. Hidradenitis Suppurativa Lesion Count 29
7.2.1.1. Hidradenitis Suppurativa Clinical Response 30
7.2.1.2. AN and ANdT Counts 30
7.2.1.3. International Hidradenitis Suppurativa Severity Score System 30
7.2.1.4. Flare Incidence 31
7.2.1.5. Lesion Rescue 31
7.2.1.6. Hurley Stage 31
7.2.2. Patient-Reported Outcomes 31
7.2.2.1. Skin Pain NRS 31
7.2.2.2. Analgesic Use 31
7.2.2.3. Itch NRS 32
7.2.2.4. Hidradenitis Suppurativa Quality of Life 32
7.2.2.5. Dermatology Life Quality Index 33
7.2.2.6. Functional Assessment of Chronic Illness Therapy – Fatigue Scale 34
7.2.2.7. Work Productivity and Activity Impairment – Hidradenitis Suppurativa 34
7.2.2.8. EQ-5D-5L Questionnaire 35
7.3. Efficacy Hypotheses 35
7.4. Analysis of the Primary Efficacy Parameter 36
7.4.1. Primary Estimand 36
7.4.2. Sensitivity and Supportive Analyses of Primary Endpoint 37

7.4.3. Subgroup Analyses for Primary Endpoint 37
7.5. Analysis of the Secondary Efficacy Parameters 38
7.5.1. Analysis of the Key Secondary Efficacy Parameters 38
7.5.2. Analysis of Other Secondary Efficacy Parameters 40
7.6. Analysis of Exploratory Efficacy Parameters 41
8. SAFETY AND TOLERABILITY 42
8.1. General Considerations 42
8.2. Adverse Events 42
8.2.1. Adverse Event Definitions 42
8.2.2. Events of Clinical Interest 43
8.2.3. Adverse Events of Special Interest 43
8.2.4. Adverse Events Per 100 Patient-Years 44
8.2.5. Adverse Event Summaries 44
8.3. Clinical Laboratory Tests 46
8.3.1. Laboratory Value Definitions 46
8.3.2. Laboratory Value Summaries 46
8.3.3. Potentially Clinically Important Laboratory Values 46
8.3.4. Potential Hy's Law Events 47
8.4. Vital Signs 48
8.5. Electrocardiograms 48
9. INTERIM ANALYSES 49
10. CHANGES AND MODIFICATIONS TO THE ANALYSIS PLAN 49
10.1. Changes to Protocol-Defined Analyses 49
10.2. Changes to the Statistical Analysis Plan 49
11. REFERENCES 50
APPENDIX A. PLANNED TABLES, FIGURES, AND LISTINGS 51
[redacted]

[redacted]

LIST OF TABLES

Table 1: Objectives and Endpoints 9
Table 2: Powers for Primary and Key Secondary Endpoints 19
Table 3: Definition of Baseline 21
Table 4: Definition of First and Last Dose Dates 28
Table 5: Hidradenitis Suppurativa Quality of Life Scoring 32
Table 6: Hidradenitis Suppurativa Quality of Life Subscales 33
Table 7: Primary Estimand 36
Table 8: Key Secondary Estimands 39
[redacted]
Table 11: Criteria for Potentially Clinically Important Laboratory Values 47
Table 12: Criteria for Clinically Notable Vital Sign Abnormalities 48
Table 13: Statistical Analysis Plan Versions 49

LIST OF FIGURES

Figure 1: Study Design Schema 15
Figure 2: Graphic Approach for Multiplicity Adjustment for Testing Primary and Key Secondary Endpoints 18

LIST OF ABBREVIATIONS

Abbreviation	Definition
AE	adverse event
AESI	adverse event of special interest
ALT	alanine aminotransferase
AN	abscess and inflammatory nodule
ANdT	abscess, inflammatory nodule, and draining tunnel (formerly known as draining fistula)
AST	aspartate aminotransferase
BMI	body mass index
$C_{avg,ss}$	average concentration at steady state
CI	confidence interval
$C_{max,ss}$	maximum concentration at steady state
CMH	Cochran-Mantel-Haenszel
CPK	creatine phosphokinase
CRF	Case Report Form
$C_{tau,ss}$	trough concentration at steady state
CTCAE	Common Terminology Criteria for Adverse Events
DLQI	Dermatology Life Quality Index
ECG	electrocardiogram
ECI	event of clinical interest
eCRF	electronic case report form
EMA	European Medicines Agency
EOS	end of study
EOT	end of treatment
EQ VAS	EuroQol visual analogue scale
EQ-5D-5L	EuroQol 5-dimension 5-level
EU	European Union
EXT	extension
FACIT-F	Functional Assessment of Chronic Illness Therapy - Fatigue
FDA	Food and Drug Administration
GRF	glomerular filtration rate
HDL	high-density lipoprotein
HiSCR	hidradenitis suppurativa clinical response
HiSQoL	hidradenitis suppurativa quality of life
HLT	high-level term
HS	hidradenitis suppurativa
IHS4	International Hidradenitis Suppurativa Severity Score System

Abbreviation	Definition
IL	interleukin
ITT	intent-to-treat
JAK	Janus kinase
LDL	low-density lipoprotein
MACE	major adverse cardiovascular event
MedDRA	Medical Dictionary for Regulatory Activities
MMRM	mixed model for repeated measures
NCI	National Cancer Institute
NRS	numerical rating scale
PC	placebo-controlled
PD	pharmacodynamic(s)
PK	pharmacokinetic(s)
PP	per Protocol
PT	preferred term
PY	patient-years
QD	once daily
QoL	quality of life
QTcF	QT interval corrected using Fridericia's formula
SAP	Statistical Analysis Plan
SMQ	standardized MedDRA query
SOC	system organ class
$t_{½}$	apparent terminal-phase disposition half-life
TEAE	treatment-emergent adverse event
$t_{max,ss}$	time to maximum concentration at steady state
TNF	tumor necrosis factor
ULN	upper limit of normal
UK	United Kingdom
US	United States
VAS	visual analog scale
WHO	World Health Organization
WPAI-HS	Work Productivity and Activity Impairment in Hidradenitis Suppurativa

1. INTRODUCTION

INCB 54707-301 is a Phase 3, multicenter, randomized, parallel-group, PC, double-blind study in adult participants with moderate to severe HS (Hurley Stage II or III), HS lesions in ≥ 2 distinct anatomical areas, a total AN count ≥ 5 at screening and baseline, and a history of failure of, intolerance to, or contraindication to an adequate course of conventional systemic therapy for HS. Approximately 600 participants will be randomized 1:1:1 to povorcitinib (45 or 75 mg QD) or placebo QD for 12 weeks; participants who complete the PC period may continue to a 42-week EXT period with povorcitinib 45 or 75 mg QD. Participants will be stratified based on ANdT count (< 11 or ≥ 11) at baseline and previous treatment of HS with a biologic drug. Section 2 of the Protocol provides a detailed description of the investigational product, target patient population, rationale for doses to be examined, and potential risks and benefits of treatment with povorcitinib.

INCB 54707-302 has a study design identical to INCB 54707-301; this SAP applies to both studies. The purpose of this SAP is to provide details of the statistical analyses that have been outlined in the INCB 54707-301 and INCB 54707-302 Protocols.

Population PK analysis will be conducted by the Incyte pharmacokineticist, and the details of the analysis methodology and results will appear in a separate report.

The details of the analysis methodology of biomarkers and PD and results will appear in a separate report.

2. STUDY INFORMATION, OBJECTIVES, AND ENDPOINTS

2.1. Protocol and Case Report Form Version

This SAP is based on INCB 54707-301 Protocol Amendment 2 and INCB 54707-302 Protocol Amendment 2, both dated 01 MAY 2023, and CRFs for each study approved on 05 FEB 2024. Unless superseded by an amendment, this SAP will be effective for all subsequent Protocol amendments and eCRF versions.

2.2. Study Objectives and Endpoints

Table 1 presents the objectives and endpoints.

Table 1: Objectives and Endpoints

Objectives	Endpoints
**Primary**	
To evaluate the efficacy of povorcitinib.	• Proportion of participants who achieve HiSCR at Week 12.<br>− HiSCR is defined as at least a 50% reduction from baseline in the total abscess and inflammatory nodule count, with no increase from baseline in abscess or draining tunnel count.
**Key Secondary**	
To further evaluate the efficacy of povorcitinib.	• Proportion of participants who achieve HiSCR75 at Week 12.<br>− HiSCR75 is defined as at least a 75% reduction from baseline in the total abscess and inflammatory nodule count, with no increase from baseline in abscess or draining tunnel count.
To evaluate the effect of povorcitinib in reducing the incidence of flares.	• Proportion of participants with flare by Week 12.<br>− Participants who experience at least 1 flare over 12 weeks; flare is defined as at least a 25% increase in the total abscess and inflammatory nodule count with a minimum increase of 2 relative to baseline.
To evaluate the effect of povorcitinib in decreasing skin pain.	• Key secondary endpoint for regulatory purposes in US/FDA/aligned countries:<br>Proportion of participants with a ≥ 3-point decrease in Skin Pain NRS score at Week 12 among participants with baseline Skin Pain NRS score ≥ 3.<br>− Participants with a Skin Pain score of at least 3 at baseline and who experience at least a 3-point decrease in Skin Pain score at Week 12, relative to baseline. Skin Pain is an 11-point NRS, ranging from 0 (no skin pain) to 10 (worst skin pain).<br>• Key secondary endpoint for regulatory purposes in EU/EMA/aligned countries:<br>Proportion of participants who achieve Skin Pain NRS30 at Week 12 among participants with baseline Skin Pain NRS score ≥ 3.<br>− Participants with a Skin Pain score of at least 3 at baseline and who achieve at Week 12 Skin Pain NRS30, defined as at least a 30% reduction and at least 1-unit reduction from baseline in the Skin Pain NRS.

Table 1: Objectives and Endpoints (Continued)

<table>
<tr><th>Objectives</th><th>Endpoints</th></tr>
<tr><td colspan="2">Secondary</td></tr>
<tr><td>To further evaluate the effect of povorcitinib in decreasing skin pain.</td><td>• Secondary endpoint for regulatory purposes in US/FDA/aligned countries:
Proportion of participants who achieve Skin Pain NRS30 at Week 12 among participants with baseline Skin Pain NRS score ≥ 3.
− Participants with a Skin Pain score of at least 3 at baseline and who achieve at Week 12 Skin Pain NRS30, defined as at least a 30% reduction and at least 1-unit reduction from baseline in the Skin Pain NRS.
• Secondary endpoint for regulatory purposes in EU/EMA/aligned countries:
Proportion of participants with a ≥ 3-point decrease in Skin Pain NRS score at Week 12 among participants with baseline Skin Pain NRS score ≥ 3.
− Participants with a Skin Pain score of at least 3 at baseline and who experience at least a 3-point decrease in Skin Pain score at Week 12, relative to baseline. Skin Pain is an 11-point NRS, ranging from 0 (no skin pain) to 10 (worst skin pain).</td></tr>
<tr><td>To evaluate the effect of povorcitinib in improving fatigue.</td><td>• Proportion of participants with at least a 4-point increase from baseline in FACIT-F score at Week 12.
− Participants with a baseline FACIT-F score ≤ 48 and who experience at least a 4-point increase in FACIT-F score at Week 12, relative to baseline. The FACIT-F scale is a 13-item questionnaire that assesses self-reported fatigue and its impact upon daily activities and function over the past 7 days, with scores ranging from 0 (worst fatigue) to 52 (no fatigue).</td></tr>
<tr><td>To evaluate the effect of povorcitinib on QoL during the PC and EXT periods.</td><td>Mean change from baseline in DLQI score at each visit.</td></tr>
<tr><td>To further evaluate the efficacy of povorcitinib.</td><td>• Mean change from baseline in abscess count at each visit.
• Percentage change from baseline in abscess count at each visit.
• Mean change from baseline in inflammatory nodule count at each visit.
• Percentage change from baseline in inflammatory nodule count at each visit.
• Mean change from baseline in draining tunnel count at each visit.
• Percentage change from baseline in draining tunnel count at each visit.</td></tr>
</table>

Table 1: Objectives and Endpoints (Continued)

Objectives	Endpoints
**Secondary (continued)**	
To evaluate the efficacy of povorcitinib during the EXT period.	• Proportion of participants who achieve HiSCR at Week 24.<br>• Proportion of participants who achieve HiSCR75 at Week 24.<br>• Proportion of participants with flare by Week 24.<br>• Proportion of participants who achieved Skin Pain NRS30 at Week 24 among participants with baseline Skin Pain NRS score ≥ 3.<br>• Proportion of participants who achieve HiSCR at Week 54.<br>• Proportion of participants who achieve HiSCR75 at Week 54.<br>• Proportion of participants with flare by Week 54.<br>• Proportion of participants who achieved Skin Pain NRS30 at Week 54 among participants with baseline Skin Pain NRS score ≥ 3.
To evaluate the maintenance of response during the EXT period.	• Proportion of participants who achieve maintenance of HiSCR or greater response at each visit during the EXT period.<br>− Maintenance of response defined as participants who achieve HiSCR at Week 12 and maintain it or achieve greater response at each visit during the EXT period.<br>• Proportion of participants who achieve maintenance of HiSCR75 or greater response at each visit during the EXT period.<br>− Maintenance of response defined as participants who achieve HiSCR75 at Week 12 and maintain it or achieve greater response at each visit during the EXT period.
**Safety**	
To evaluate the safety and tolerability of povorcitinib.	Frequency and severity of AEs, including changes in clinical laboratory results, vital signs, ECGs, and the results of physical examinations.
**Exploratory**	
To evaluate the efficacy of povorcitinib.	• Proportion of participants who achieve HiSCR at each visit.<br>• Proportion of participants who achieve HiSCR75 at each visit.<br>• Proportion of participants who achieve HiSCR90 at each visit.<br>− HiSCR90 is defined as at least a 90% reduction from baseline in total abscess and inflammatory nodule count, with no increase from baseline in abscess or draining tunnel count.<br>• Proportion of participants with a total abscess and inflammatory nodule count of 0, 1, or 2 at each visit.<br>• Proportion of participants who achieve AN50, AN75, AN90, and AN100 (relative to baseline) at each visit.<br>− Defined as total abscess and inflammatory nodule count change of ≥ 50%, ≥ 75%, ≥ 90%, and ≥ 100% relative to baseline, respectively.<br>• Proportion of participants who achieve ANdT50, ANdT75, ANdT90, and ANdT100 (relative to baseline) at each visit.<br>− Defined as abscess, inflammatory nodule, and draining tunnel count change of ≥ 50%, ≥ 75%, ≥ 90%, and ≥ 100% relative to baseline, respectively.

Table 1: Objectives and Endpoints (Continued)

Objectives	Endpoints
**Exploratory (continued)**	
To evaluate the efficacy of povorcitinib (continued).	• Mean change from baseline in total abscess and inflammatory nodule count at each visit.<br>• Percentage change from baseline in total abscess and inflammatory nodule count at each visit.<br>• Mean change from baseline in International Hidradenitis Suppurativa Severity Score System (IHS4) score at each visit.<br>− The IHS4 assesses the severity of HS, and the resulting score is calculated by the number of inflammatory nodules (multiplied by 1) plus the number of abscesses (multiplied by 2) plus the number of draining tunnels (multiplied by 4).<br>• Percentage change from baseline in IHS4 score at each visit.<br>• Proportion of participants who achieve IHS4-55 at each visit.<br>− IHS4-55 is defined as at least a 55% reduction from baseline in total IHS4 score.<br>• Proportion of participants in each of the IHS4 disease categories at each visit.<br>− IHS4 disease categories are described as follows: a total score of 3 or less signifies mild, 4-10 signifies moderate, and 11 or higher signifies severe disease.
To evaluate the effect of povorcitinib in reducing the incidence of flares.	• Proportion of participants with HS flares at each visit.<br>• Proportion of participants with at least 1 HS flare during the EXT period.<br>• Mean number of flares during the PC period.<br>• Mean number of flares during the EXT period.<br>• Mean time to the first flare during the PC period.<br>• Mean time to the first flare during the EXT period.
To evaluate the effect of povorcitinib on skin pain.	• Proportion of participants achieving Skin Pain NRS30 at each visit among participants with baseline Skin Pain NRS score ≥ 3.<br>• Proportion of participants with a ≥ 3-point decrease from baseline in Skin Pain score at each visit.<br>− Participants with a Skin Pain NRS score of at least 3 at baseline and who experience at least a 3-point decrease in Skin Pain score at each visit, relative to baseline.<br>• Proportion of participants with a ≥ 4-point decrease from baseline in Skin Pain score at each visit.<br>− Participants with a Skin Pain NRS score of at least 4 at baseline and who experience at least a 4-point decrease in Skin Pain score at each visit, relative to baseline.<br>• Mean change from baseline in Skin Pain NRS score at each visit.<br>• Percentage change from baseline in Skin Pain NRS score at each visit.<br>• Proportion of participants with analgesic (nonopioid and opioid) use at each visit.<br>• Proportion of participants with opioid use at each visit.

Table 1: Objectives and Endpoints (Continued)

Objectives	Endpoints
**Exploratory (continued)**	
To evaluate the effect of povorcitinib in decreasing draining tunnel count.	• Proportion of participants with at least a 1-unit decrease from baseline in draining tunnel count at each visit.<br>− Participants with at least 1 draining tunnel at baseline and who experience at least a 1-unit decrease in draining tunnel count at each visit, relative to baseline.<br>• Mean change from baseline in draining tunnel count at each visit.<br>• Percentage change from baseline in draining tunnel count at each visit.
To evaluate the effect of povorcitinib on itch.	• Proportion of participants who achieve Itch NRS30 at each visit for participants with Itch NRS score ≥ 3 at baseline.<br>− Itch NRS30 is defined as at least a 30% reduction and at least 1-unit reduction from baseline in the Itch NRS score (range from 0 [no itch] to 10 [worst itch imaginable]).<br>• Proportion of participants who achieve at least a 4-point reduction in Itch NRS score from baseline at each visit for participants with Itch NRS score ≥ 4 at baseline.<br>• Mean change from baseline in Itch NRS score at each visit.<br>• Percentage change from baseline in Itch NRS score at each visit.
To assess the need for rescue lesional treatment.	• Proportion of participants requiring rescue lesional treatment during the PC period.<br>• Proportion of participants requiring rescue lesional treatment during the EXT period.
To assess the need for systemic antibiotic rescue therapy.	• Proportion of participants requiring systemic antibiotic rescue therapy during the PC period.<br>• Proportion of participants requiring systemic antibiotic rescue therapy during the EXT period.
To evaluate the effect of povorcitinib on QoL.	• Mean change from baseline in HiSQoL score at each visit.<br>• Percentage change from baseline in HiSQoL score at each visit.<br>• Percentage change from baseline in DLQI score at each visit.<br>• Proportion of participants with at least a 4-point decrease in DLQI score from baseline at each visit for participants with DLQI score ≥ 4 at baseline.<br>• Mean change from baseline in FACIT-F score at each visit.<br>• Percentage change from baseline in FACIT-F score at each visit.<br>• Proportion of participants with at least a 4-point increase in FACIT-F score at each visit.<br>• Mean change from baseline in WPAI-HS score at each visit.<br>• Mean change from baseline in EQ-5D-5L score at each visit.
To explore blood biomarkers in participants treated with povorcitinib.	Expression of select biomarkers in peripheral blood at baseline, Week 3, and Week 12.

Table 1: Objectives and Endpoints (Continued)

Objectives	Endpoints
**Exploratory (continued)**	
To determine the systemic exposure of povorcitinib.	Population PK parameters of povorcitinib such as apparent clearance, apparent volume of distribution, apparent oral absorption rate constant, and absorption lag time, as deemed applicable, as well as model-based post hoc predictions of steady-state PK exposures such as $C_{max,ss}$, $C_{avg,ss}$, $C_{tau,ss}$, $t_{max,ss}$, and $t_{½}$.
To determine the povorcitinib PK/PD relationship.	E-R analysis of select clinical response endpoints as deemed appropriate, such as HiSCR at Week 12, HiSCR75 at Week 12, Skin Pain at Week 12, etc.

3. STUDY DESIGN

INCB 54707-301 and INCB 54707-302 are Phase 3, multicenter, randomized, parallel-group, PC, double-blind studies to evaluate the efficacy and safety of povorcitinib (45 or 75 mg QD) over a 12-week PC period followed by a 42-week EXT period. The studies will enroll participants aged 18 years or older who had a diagnosis of moderate to severe HS (Hurley Stage II or III) for at least 3 months, HS lesions in ≥ 2 distinct anatomical areas, a total AN count ≥ 5 at screening and baseline, and a history of failure of, intolerance to, or contraindication to an adequate course of conventional systemic therapy for HS.

Participants will be screened for up to 28 days before the first dose of study drug. Participants will be randomized 1:1:1 to povorcitinib (45 or 75 mg QD) or placebo QD and stratified based on ANdT count (< 11 or ≥ 11) at baseline and pervious treatment of HS with a biologic drug. Participants who complete the PC period may continue to a 42-week EXT period with povorcitinib 45 or 75 mg QD.

Participants randomized to povorcitinib at baseline will remain at their respective dose level (45 or 75 mg QD) throughout the PC and EXT periods, while participants randomized to placebo will receive povorcitinib 45 or 75 mg QD (1:1 ratio) for the EXT period after completion of the PC period. Participants who complete 54 weeks of treatment in this study may be eligible to participate in a separate EXT study to further evaluate the safety and efficacy of the investigational product. For participants who do not participate in the separate EXT study, a safety follow-up visit should be conducted approximately 30 days after the EOT visit, or last administration of study drug (in case of premature discontinuation/early termination).

The study schema is shown below in Figure 1.

Figure 1: Study Design Schema

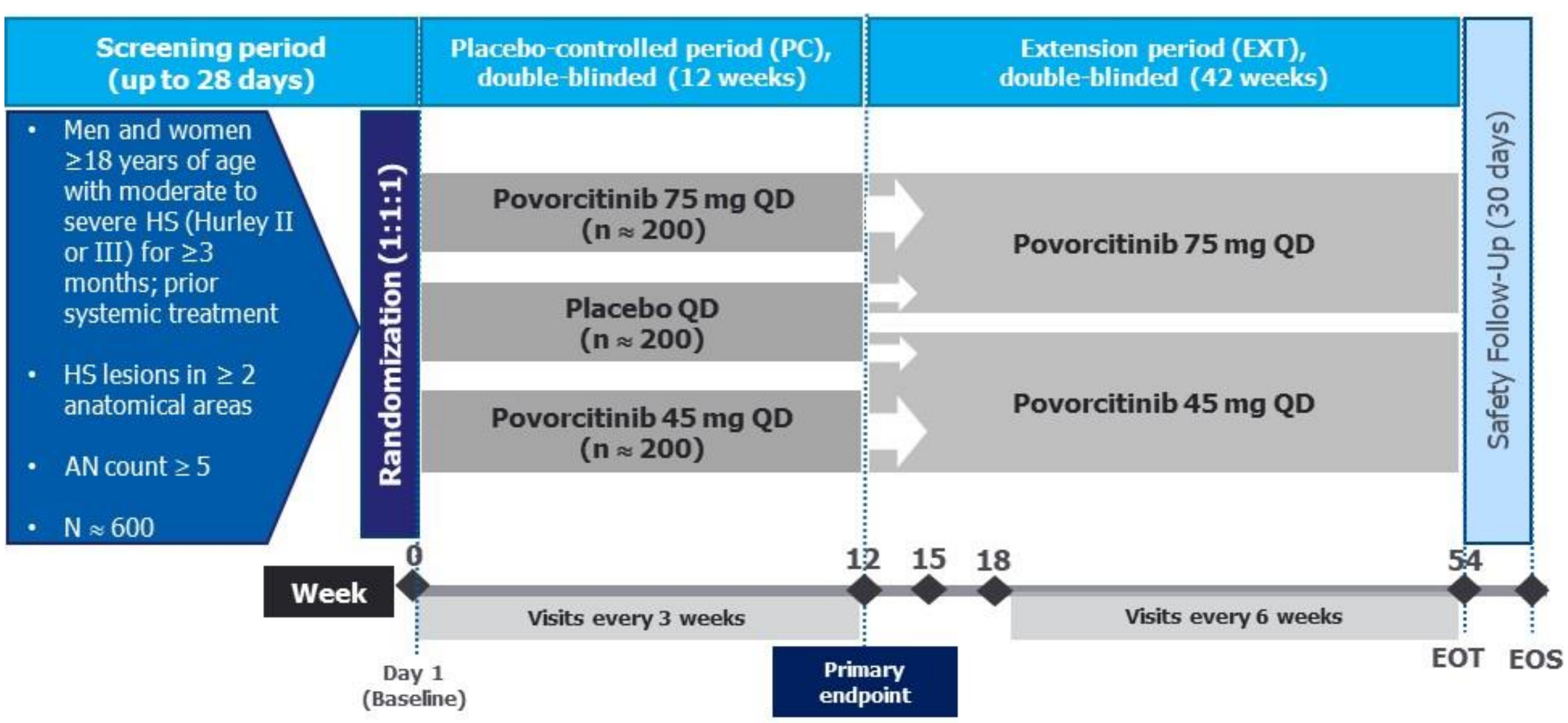

The overall study period will include both the PC and EXT periods and is 54 weeks.

Given the design, a 12-week analysis will be performed to assess efficacy and safety during the PC, double-blind treatment period. This will occur after all participants have completed the study activities up to Week 12 and all data pertaining to the PC period are cleaned.

The final analysis will occur once the last participant completes the last visit in each study.

3.1. Randomization

In each study, approximately 600 participants will be randomized 2:2:1:1 to 1 of the following 4 treatment groups at the time of randomization (baseline):

- Povorcitinib 45 mg
- Povorcitinib 75 mg
- Placebo to povorcitinib 45 mg
- Placebo to povorcitinib 75 mg

The 2:2:1:1 randomization ratio will maintain the overall randomization ratio of 1:1:1 to povorcitinib (45 or 75 mg) or placebo for the 12-week PC period. Participant randomization will be stratified based on ANdT count (< 11 or ≥ 11) and previous treatment of HS with a biologic drug.

Participants who complete the PC period will enter the EXT period. During the EXT period, participants randomized to povorcitinib at baseline will remain at their respective dose level (45 or 75 mg), while participants randomized to placebo will receive povorcitinib 45 or 75 mg according to the treatment group assignment.

3.2. Control of Type I Error

For the primary and key secondary endpoints, the overall familywise Type I error rate will be 2-sided $\alpha = 0.05$.

The following is a list of primary and key secondary null hypotheses to be tested, denoted by $H_{ij,0}$, where i indicates the povorcitinib dose group (i = 1 for 75 mg, i = 2 for 45 mg) and j is the numerical identifier of the endpoint for the dose group (j = 1 for HiSCR at Week 12, j = 2 for HiSCR75 at Week 12, j = 3 for flare by Week 12, j = 4 for Skin Pain Response at Week 12).

Primary null hypotheses:

- $H_{11,0}$: Proportion of participants achieving HiSCR at Week 12 in the povorcitinib 75 mg group is equal to the placebo group
- $H_{21,0}$: Proportion of participants achieving HiSCR at Week 12 in the povorcitinib 45 mg group is equal to the placebo group

Key secondary null hypotheses:

- $H_{12,0}$: Proportion of participants achieving HiSCR75 at Week 12 in the povorcitinib 75 mg group is equal to the placebo group
- $H_{22,0}$: Proportion of participants achieving HiSCR75 at Week 12 in the povorcitinib 45 mg group is equal to the placebo group
- $H_{13,0}$: Proportion of participants with flare by Week 12 in the povorcitinib 75 mg group is equal to the placebo group

- $H_{23,0}$: Proportion of participants with flare by Week 12 in the povorcitinib 45 mg group is equal to the placebo group
- $H_{14,0}$: Proportion of participants achieving Skin Pain Response at Week 12 in the povorcitinib 75 mg group is equal to the placebo group
- $H_{24,0}$: Proportion of participants achieving Skin Pain Response at Week 12 in the povorcitinib 45 mg group is equal to the placebo group

where Skin Pain Response is defined as a ≥ 3-point reduction from baseline in Skin Pain NRS for regulatory purposes in US/FDA/aligned countries or Skin Pain NRS30 for regulatory purposes in EU/EMA/aligned countries.

To adjust for multiplicity and provide strong control of overall familywise error rate, the graphic approach (Bretz et al 2011) will be used in the primary and the key secondary endpoints outlined in Figure 2.

In Step 1, within Family 1, the algorithm begins by testing the null hypotheses for each of the 2 povorcitinib dose groups versus placebo on the primary endpoint ($H_{11,0}$ and $H_{21,0}$) at its assigned significance level of 2-sided $\alpha = 0.025$. If any hypothesis is rejected (ie, deemed significant), then its significance level will be transferred to the subsequent test following the arrow. The numbers along the arrows denote the weights for transferring significance levels. Specifically, a weight of 1 indicates 100% transfer of the significance level. It should be noted it is possible that a hypothesis for a particular endpoint fails to be rejected the first time it is tested but subsequently receives enough significance level from successfully rejected hypotheses and is rejected the second time it is tested. This iterative process of updating the graph and redistribution of the significance level is continued until no further hypotheses can be tested within Family 1.

In Step 2, if all 4 null hypotheses within Family 1 are rejected, the 2 null hypotheses $H_{14,0}$ and $H_{13,0}$ for povorcitinib 75 mg versus placebo within Family 2 will be tested using the gatekeeping procedure at a significance level of 2-sided $\alpha = 0.05$. The null hypothesis $H_{14,0}$ will be tested first, if it is rejected, then the null hypothesis $H_{13,0}$ will be tested.

In Step 3, if both null hypotheses $H_{13,0}$ and $H_{14,0}$ within Family 2 are rejected, the 2 null hypotheses $H_{24,0}$ and $H_{23,0}$ for povorcitinib 45 mg versus placebo within Family 3 will be tested using the gatekeeping procedure at a significance level of 2-sided $\alpha = 0.05$. The null hypothesis $H_{24,0}$ will be tested first, if it is rejected, then the null hypothesis $H_{23,0}$ will be tested.

Other secondary and exploratory endpoints will be tested using 2-sided $\alpha = 0.05$ but are not considered Type I error–controlled hypotheses.

Figure 2: Graphic Approach for Multiplicity Adjustment for Testing Primary and Key Secondary Endpoints

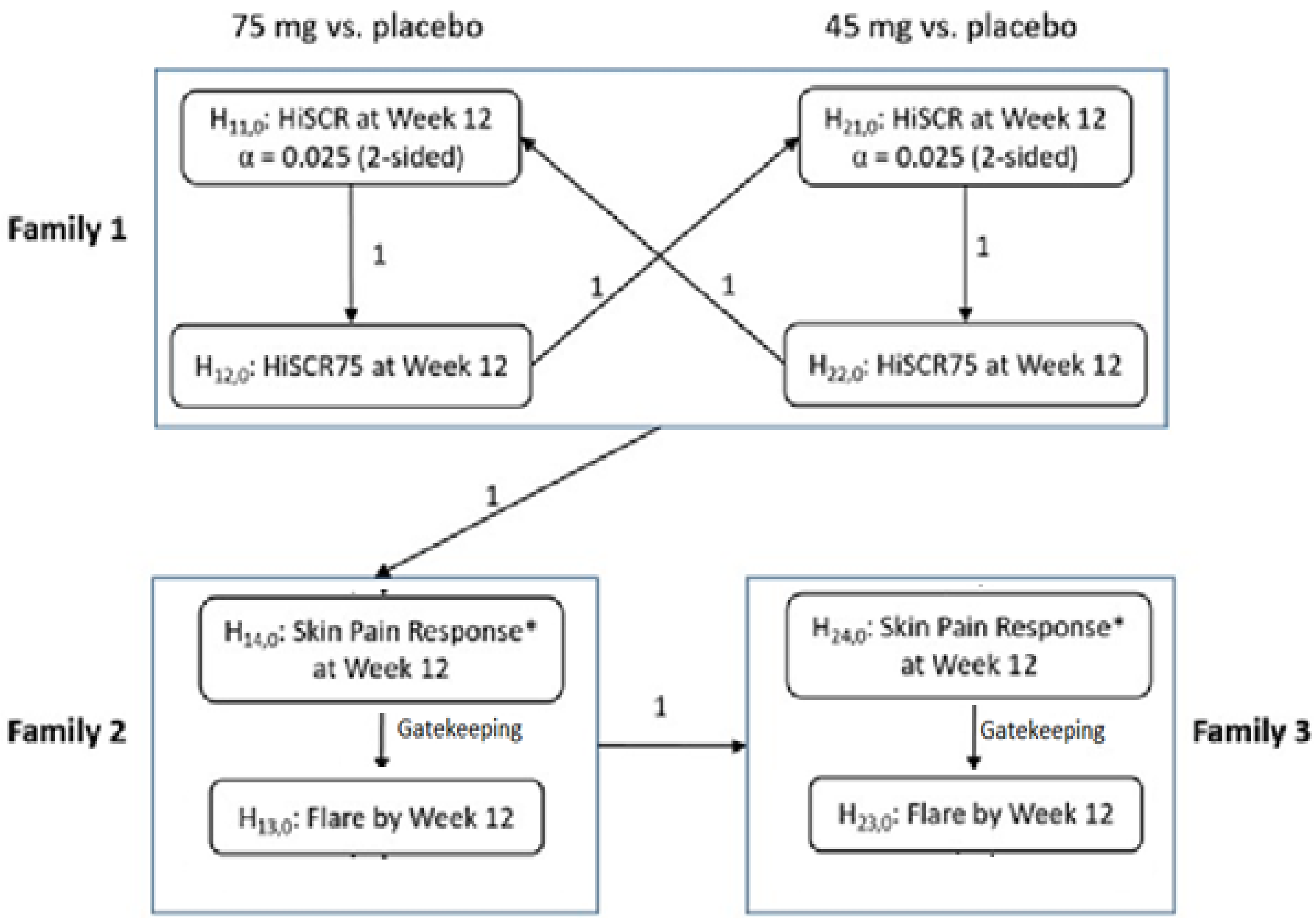

* Skin Pain Response is defined as a ≥ 3-point reduction from baseline in Skin Pain NRS for regulatory purposes in US/FDA/aligned countries or Skin Pain NRS30 for regulatory purposes in EU/EMA/aligned countries.

3.3. Sample Size Considerations

The primary endpoint of the studies is the proportion of participants who achieve HiSCR at Week 12. Participants will be randomized in a 1:1:1 ratio to 1 of 2 doses of povorcitinib (45 or 75 mg QD) or placebo and stratified by ANdT count (< 11 and ≥ 11; approximately ≤ 50% of participants may have an ANdT count < 11) and previous treatment of HS with a biologic drug. Based on the observed HiSCR rates at Weeks 8 and 12 reported in a Phase 2, randomized, dose-ranging study of povorcitinib (INCB 54707-204), it is assumed that the HiSCR rate at Week 12 would be 48% for the povorcitinib 75-mg group, 45% for the povorcitinib 45-mg group, and 30% for the placebo group. Using the chi-square test, without continuity correction, at a 2-sided $\alpha = 0.025$, a sample size of 200 participants per group (with a total of 600 participants) will achieve approximately 93% power to detect a statistically significant difference between the povorcitinib 75-mg group and the placebo group and approximately 80.7% power to detect a statistically significant difference between the povorcitinib 45-mg and the placebo group.

The powers for the primary and key secondary endpoints are provided in Table 2.

Table 2: Powers for Primary and Key Secondary Endpoints

Endpoints	Povorcitinib Dose Group	Response Rate for Povorcitinib Dose Group[a]	Response Rate for Placebo Group[a]	Power
Primary endpoint				
Proportion of participants who achieve HiSCR at Week 12	Povorcitinib 75 mg	48%	30%	93%
	Povorcitinib 45 mg	45%	30%	80.7%[b]
Key secondary endpoints				
Proportion of participants who achieve HiSCR75 at Week 12	Povorcitinib 75 mg	38%	20%	96.1%[b]
	Povorcitinib 45 mg	33%	20%	76.2%[b]
Proportion of participants with flare by Week 12	Povorcitinib 75 mg	20%	40%	98.5%[b]
	Povorcitinib 45 mg	25%	40%	83.5%[b]
Proportion of participants achieving ≥ 3-point reduction in Skin Pain NRS at Week 12[c], for regulatory purposes in US/FDA/aligned countries	Povorcitinib 75 mg	22.5%	7.5%	91.3%[d]
	Povorcitinib 45 mg	20%	7.5%	79.6%[d]
Proportion of participants achieving NRS30 at Week 12[e], for regulatory purposes in EU/EMA/aligned countries	Povorcitinib 75 mg	34%	18%	90.3%[b]
	Povorcitinib 45 mg	34%	18%	90.3%[b]

[a] Based on the results from a Phase 2, randomized, dose-ranging study (INCB 54707-204).
[b] Based on the chi-square test at a 2-sided $\alpha = 0.025$.
[c] It is expected that approximately 76% of participants in the ITT Population will have Skin Pain NRS score ≥ 3 at baseline based on the results from Study INCB 54707-204.
[d] Based on the Fisher exact test at a 2-sided $\alpha = 0.025$.
[e] It is expected that approximately 93% of participants in the ITT Population will have Skin Pain NRS score ≥ 1 at baseline based on the results from Study INCB 54707-204.

3.4. Schedule of Assessments

Refer to INCB 54707-301 Protocol Amendment 2 and INCB 54707-302 Protocol Amendment 2 dated 01 MAY 2023 for a full description of all study procedures and assessment schedules.

4. DATA HANDLING DEFINITIONS AND CONVENTIONS

4.1. Scheduled Study Evaluations and Study Periods

4.1.1. Day 1

Day 1 is the date that the first dose of povorcitinib or placebo is administered to the participants.

For randomized participants not treated with any study drug, Day 1 is defined as the date of randomization.

4.1.2. Study Day

If a visit/reporting date is on or after Day 1, then the study day at the visit/reporting date will be calculated as

Day # = (visit/reporting date – Day 1 date + 1).

If the visit/reporting date is before Day 1, then the study day at the visit/reporting date will be calculated as

Day # = (visit/reporting date – Day 1 date).

A study day of –1 indicates 1 day before Day 1.

4.1.3. Baseline Value

Baseline for the Skin Pain NRS score is defined as the average of the 7 daily Skin Pain NRS scores before randomization. If 4 or more daily scores are missing (out of the 7), the baseline Skin Pain NRS will be set to missing.

Baseline for the Itch NRS score is defined as the average of the 7 daily Itch NRS scores before randomization. If 4 or more daily scores are missing (out of the 7), the baseline Itch NRS will be set to missing.

The baseline value of all other efficacy parameters is the last nonmissing measurement obtained before randomization, unless otherwise specified.

The baseline value of safety parameters is the last nonmissing measurement obtained before the first administration of povorcitinib or placebo in the PC period, unless otherwise specified. For randomized participants not treated with any study drug, baseline is defined as the last nonmissing assessment before or on the day of randomization for all parameters.

Note that for participants who originally received placebo in the PC period and received at least 1 dose of povorcitinib during the EXT period (hereafter referred to as crossover participants), the baseline definition in the EXT period is defined as follows:

- For efficacy evaluations in the EXT period, baseline is the last nonmissing measurement obtained before randomization in the PC period (hereafter referred to as PC baseline).
- For safety evaluations in the EXT period, baseline is the last nonmissing measurement obtained before the first administration of povorcitinib 45 or 75 mg in the EXT period (hereafter referred to as EXT baseline).

Table 3 provides an outline regarding the baseline for efficacy and safety evaluations in each period.

Table 3: Definition of Baseline

Treatment Period	Treatment Groups	Baseline For Efficacy Evaluations	Baseline For Safety Evaluations
PC period<br>(up to Week 12)	Placebo<br>Povorcitinib 45 mg<br>Povorcitinib 75 mg	PC baseline	PC baseline
EXT period<br>(Week 12 through Week 54)	Placebo to povorcitinib 45 mg<br>Placebo to povorcitinib 75 mg	PC baseline	EXT baseline
Overall study period<br>(up through Week 54)	Povorcitinib 45 mg<br>Povorcitinib 75 mg	PC baseline	PC baseline

When scheduled assessments and unscheduled assessments occur on the same day and the time of the assessment or time of first dose is not available, use the following convention to determine baseline:

- If both a scheduled and an unscheduled visit are available on the day of the first dose and the time is missing, use the scheduled assessment as baseline.
- If all scheduled assessments are missing on the day of the first dose and an unscheduled assessment is available, use the unscheduled assessment as baseline.

4.1.4. Handling of Missing and Incomplete Dates

In general, values for missing dates will not be handled unless methods for handling missing dates are specified in this section or relevant sections. The original reported dates collected on the eCRF should be used in all relevant listings. The following rules will be used for handling partial dates for analyses requiring dates.

When calculating the time since diagnosis of HS, a partial HS diagnosis date will be handled as follows in the calculation:

- If only the day of the month is missing, then the first day of the month will be used.
- If both the month and day are missing, then 01 JAN of the year will be used.
- If the diagnosis date is completely missing, then the time since diagnosis will not be calculated.

When the date of the last dose in a specific period is used in deriving variables such as duration of treatment or TEAE flag for the period, a missing or partial date of the last dose in the period will be handled as follows:

- If only the day is missing, then the earlier date of the last day of the month or the date that the participant discontinued treatment during the period will be used.
- Otherwise, the date that the participant discontinued treatment during the period will be used as the date of the last dose in the period.

4.2. Variable Definitions

The following variables will only be calculated if not reported on the eCRF.

4.2.1. Body Mass Index

Body mass index will be calculated as follows:

BMI (kg/m^2) = [weight (kg)] / [height (m)]^2.

4.2.2. Prior and Concomitant Medication

Prior medication is defined as any nonstudy medication started before the first dose of povorcitinib or placebo in the PC period.

Concomitant medication in the PC period is defined as any nonstudy medication that is started accordingly:

- Before the date of first administration of povorcitinib or placebo in the PC period and is ongoing throughout the PC period or ends on/after the date of first study drug administration in the PC period

  or

- On/after the date of first administration of povorcitinib or placebo in the PC period, and before the date of first administration of povorcitinib in the EXT period for participants who entered EXT period.

Concomitant medication in the EXT period is defined as any nonstudy medication that is started accordingly:

- Before the date of first administration of povorcitinib in the EXT period and is ongoing or ends on/after the date of first study drug administration in the EXT period.

  or

- On/after the date of first administration of povorcitinib in the EXT period.

Concomitant medication in the overall study period is defined as any nonstudy medication that is started accordingly:

- Before the date of first administration of povorcitinib in the PC period and is ongoing or ends on/after the date of first study drug administration in the PC period

  or

- On/after the date of first administration of povorcitinib in the PC period.

A prior medication could also be classified as "both prior and concomitant medication" if the end date is on or after first dose of povorcitinib or placebo in the PC period. In the listing, it will be indicated whether a medication is only prior, only concomitant (by period), or both prior and concomitant.

For the purposes of analysis, all medications will be considered concomitant medications unless the medications can unequivocally be defined as not concomitant.

5. STATISTICAL METHODOLOGY

5.1. General Methodology

Unless otherwise noted, SAS® software (SAS Institute Inc, Cary, NC; v9.1 or later) will be used for the generation of all tables, graphs, and statistical analyses. Descriptive summaries for continuous variables will include but not be limited to the number of observations, mean, standard deviation, median, minimum, and maximum. Descriptive summaries for categorical variables will include the number and percentage of participants in each category.

5.2. Treatment Groups

Each study is randomized, double-blind, and PC over a 12-week PC period followed by a 42-week EXT period. Table summaries, unless otherwise indicated, will present data by treatment groups.

For the PC period, data will be summarized by the following treatment groups:

- Placebo
- Povorcitinib 45 mg
- Povorcitinib75 mg

For the EXT period, data will be summarized by the following treatment groups:

- Placebo to povorcitinib 45 mg, including participants who originally received placebo in the PC period and received at least 1 dose of povorcitinib 45 mg during the EXT period with up to 42 weeks of povorcitinib 45 mg exposure
- Placebo to povorcitinib 75 mg, including participants who originally received placebo in the PC period and received at least 1 dose of povorcitinib 75 mg during the EXT period with up to 42 weeks of povorcitinib 75 mg exposure

For the overall study period, the results will be summarized and presented by the following treatment groups:

- Povorcitinib 45 mg, including participants who received at least 1 dose of povorcitinib 45 mg during the PC period with up to 54 weeks of povorcitinib 45 mg exposure
- Povorcitinib 75 mg, including participants who received at least 1 dose of povorcitinib 75 mg during the PC period with up to 54 weeks of povorcitinib 75 mg exposure

5.3. Analysis Populations

5.3.1. All-Screened Population

The All-Screened Population will include all participants who signed the informed consent form.

5.3.2. Intent-to-Treat Population

The ITT Population will include all randomized participants. Treatment groups for this population will be defined according to the treatment assignment at the time of randomization regardless of the actual study treatment the participant might take during their participation in the PC period.

The ITT Population will be used for the summary of demographics, baseline characteristics, participant disposition, and analyses of efficacy data.

5.3.3. Per Protocol Population

Participants in the ITT Population who are considered to be sufficiently compliant with the Protocol will compose the PP Population.

In general, the following are considered important Protocol deviations that may significantly affect the primary analysis:

- Missing data for the primary endpoint
- Overall compliance less than 50% during the PC period
- Important Protocol deviations related to inclusion/exclusion criteria, discontinuation criteria, and use of prohibited concomitant medications.

These deviations will be reviewed by the study team. Decisions as to whether any of these deviations warrant exclusion from the PP Population by the clinical team will be made prior to database lock and randomization unblinding.

The PP Population will be used in the supportive sensitivity analysis for the primary efficacy endpoint.

5.3.4. Safety Population

The Safety Population will include all participants who received at least 1 dose of povorcitinib or placebo during the PC period. Treatment groups for this population will be determined according to the actual treatment the participant received on Day 1 regardless of assigned study treatment.

All safety analyses for the PC period as well as for the overall study period, will be conducted using the Safety Population.

5.3.5. Extension-Evaluable Population

The EXT-Evaluable Population will include all participants who received at least 1 dose of povorcitinib 45 or 75 mg during the EXT period.

The EXT-Evaluable Population will be used for the summary of demographics, baseline characteristics, and participant disposition in the EXT period.

All analyses for the participants who originally received placebo up to and including Week 12 and received at least 1 dose of povorcitinib during the EXT period will be conducted with the EXT-Evaluable Population.

6. BASELINE, EXPOSURE, AND DISPOSITION

Appendix A provides a list of data displays. Sample data displays are included in a separate document.

6.1. Demographics, Baseline Characteristics, and Disease History

Unless otherwise stated, demographics, baseline characteristics, and disease history will be summarized by treatment group and overall.

6.1.1. Demographics and Baseline Characteristics

The following demographics and baseline characteristics will be summarized for the ITT Population and the EXT-Evaluable Population: age, age category (18 to < 65 years, ≥ 65 years), sex, race, ethnicity, weight, height, BMI, BMI category (< 25 kg/m^2, ≥ 25 to < 30 kg/m^2, ≥ 30 to < 40 kg/m^2, ≥ 40 to < 50 kg/m^2, ≥ 50 kg/m^2), and geographic region (North America, Europe [UK included] and Australia, Japan).

The following baseline characteristics will be summarized for the ITT Population only: nicotine use (never used, current user, former user), medical cannabis use (never used, current user, former user), and alcohol use (never used, current user, former user).

6.1.2. Baseline Disease Characteristics

The following baseline disease characteristics and disease history will be summarized for all participants in the ITT Population:

- Prior oral antibiotic
- Prior biologic (any, biologic anti-TNF, biologic anti–IL-17, other biologic)
- Prior other HS therapy (topical antibiotic or antiseptic, topical retinoid, topical JAK inhibitor, topical corticosteroid, topical other, intralesional corticosteroid, intravenous antibiotic, intravenous corticosteroid, oral antihyperglycemic, oral hormonal therapy, oral JAK inhibitor, oral other)
- Prior HS surgery (any, incision and drainage, deroofing, lesion ablation [electrosurgery], local excision, laser therapy, other)
- Family cardiovascular/clotting disease (history of sudden cardiac death, familial hypercholesterolemia associated with premature coronary artery disease, myocardial infarction below age 50 years, factor V Leiden, protein C and/or protein S deficiency, prothrombin gene mutation, any unprovoked deep vein thrombosis, thromboembolic disease)
- Hurley stage (Stage I, Stage II, Stage III)
- AN count
- AN count category (< 5, 5-6, 7-8, 9-10, 11-15, 16-25, > 25)
- ANdT count
- ANdT count category (< 5, 5-6, 7-8, 9-10, 11-15, 16-25, > 25; < 11 and ≥ 11)

- Abscess count
- Abscess count category (0, 1, 2, 3-5, 6-10, 11-20, > 20)
- Inflammatory nodule count
- Inflammatory nodule count category (0, 1-4, 5, 6-8, 9-11, 12-15, 16-20, > 20)
- Draining tunnel count
- Draining tunnel count category (0, 1-2, 3-5, 6-10, 11-20, > 20)
- Number of anatomical areas with HS lesions (< 2, 2, 3, 4, 5, 6, 7, 8-10, ≥ 11)
- IHS4 score
- IHS4 score category (mild [≤ 3], moderate [4-10], severe [≥ 11])
- Skin Pain NRS score
- Skin Pain NRS score category (0, 1-2, ≥ 3; mild [0 to ≤ 4], moderate [> 4 to ≤ 7], severe [> 7])
- Itch NRS score
- Itch NRS score category (0, 1-2, ≥ 3; mild [0 to ≤ 4], moderate [> 4 to ≤ 7], severe [> 7])
- Analgesic use
- Opioid use
- DLQI score
- DLQI score category (0-1, 2-5, 6-10, 11-20, 21-30)
- FACIT-F score
- HiSQoL score

6.1.3. Disease History

For participants in the ITT Population, the following HS medical history variables will be summarized by assigned treatment group:

- HS disease duration (years)
  - Calculated as: disease duration (years) = (date of randomization − date of initial HS diagnosis + 1) / 365.25
- Comorbidity/disease (HS, Crohn disease, diabetes, hypertension, metabolic syndrome, rheumatoid arthritis, polycystic ovary syndrome, psoriasis)

6.1.4. Medical History

For participants in the ITT Population, medical history will be summarized by assigned treatment group. This summary will include the number and percentage of participants with medical history for each body system/organ class as documented on the eCRF.

6.2. Disposition of Participants

The number and percentage of participants enrolled by country and/or site will be provided by treatment group and overall.

The number and percentage of participants who were randomized, who were treated, who completed treatment in the PC period, who discontinued study treatment with a primary reason for discontinuation, and who withdrew from the study with a primary reason for withdrawal will be summarized for the ITT Population in the PC period.

The number and percentage of participants who were treated, who completed treatment, who discontinued study treatment with a primary reason for discontinuation, and who withdrew from the study with a primary reason for withdrawal will be summarized for the EXT-Evaluable Population in the EXT period.

6.3. Protocol Deviations

Protocol deviations recorded will be summarized by treatment group and overall for the ITT Population in the PC period and the EXT-Evaluable Population in the EXT period. Individual Protocol deviations will also be listed.

6.4. Exposure

For the Safety Population in the PC period, the EXT-Evaluable Population in the EXT period, and the participants who took only povorcitinib 45 or 75 mg during the overall study period, study drug exposure will be summarized by treatment group descriptively as the following:

- **Duration of treatment with study drug (days):** date of last dose of study drug – date of first dose of study drug + 1.

  Note that the date of first and last dose of study drug are period-specific and defined in Table 4.

The number and percentage of participants exposed to study drug during the overall study period will be summarized for the following cumulative duration intervals: ≥ 1 month, ≥ 3 months, ≥ 6 months, and ≥ 12 months.

The average daily dose of study drug will be summarized by treatment group descriptively as the following:

- **Average daily dose of povorcitinib (mg/day):** total actual povorcitinib dose taken (mg) / duration of treatment with study drug (days).

  Total actual dose taken will be calculated based on the information entered on the Drug Accountability eCRF.

Table 4: Definition of First and Last Dose Dates

Treatment Period	Treatment Group	First Dose Date	Last Dose Date
PC period	• Placebo<br>• Povorcitinib 45 mg<br>• Povorcitinib 75 mg	Date of first dose of study drug in the PC period	Date of last dose of study drug in the PC period
EXT period	• Placebo to povorcitinib 45 mg<br>• Placebo to povorcitinib 75 mg	Date of first dose of study drug in the EXT period	Date of last dose of study drug in the EXT period
Overall study period	• Povorcitinib 45 mg<br>• Povorcitinib 75 mg	Date of first dose of study drug in the PC period	Date of last dose of study drug during the overall study period

6.5. Study Drug Compliance

For the Safety Population during the PC period, the EXT-Evaluable Population during the EXT period, and the participants who took only povorcitinib 45 or 75 mg during the overall study period, overall compliance (%) for povorcitinib/placebo will be calculated as follows:

Compliance (%) = 100 × (total dose actually taken in the specific period / total prescribed dose).

The total prescribed dose is defined as the sum of the doses prescribed by the investigator accounting for dose modifications.

The total dose actually taken will be calculated as: total number of tablets dispensed – total number of tablets returned. The total number of tablets dispensed and returned in each period will be based on information entered on the Drug Accountability eCRF. If there is dispensed drug that has not been returned, the actual dose taken starting from the dispense date of the unreturned drug will be imputed by the dose taken as reported on the Dosing eCRF.

6.6. Prior and Concomitant Medication

Prior medications and concomitant medications will be coded using the WHO Drug Dictionary and will be summarized by treatment group and overall. The number and percentage of participants in the ITT Population for each prior medication and concomitant medication in the PC period will be summarized by WHO drug class and WHO drug PT as well as by study-specified category and type within the category (if applicable).

For the EXT-Evaluable Population, only concomitant medications in the EXT period will be summarized by WHO drug class and WHO drug PT as well as by study-specified category and type within the category (if applicable).

For participants who took only povorcitinib 45 or 75 mg, concomitant medications during the overall study period will be summarized by WHO drug class and WHO drug PT as well as by study-specified category and type within the category (if applicable).

In addition, for each study-specific prior treatment category, the main reason for discontinuation and response to treatment by type within the category (if applicable), will be summarized by treatment group and overall in the ITT Population. The reason for inadequate response to prior oral antibiotics or biologics for HS will also be summarized by treatment group and overall in the ITT Population.

7. EFFICACY

Appendix A provides a list of data displays. Sample data displays are included in a separate document.

7.1. General Considerations

The efficacy endpoints will be analyzed by treatment group based on ITT Population. The difference between each of the povorcitinib dose groups and placebo for the primary and key secondary efficacy endpoints will be tested using a graphical approach for multiplicity adjustment to ensure a strong control of the familywise Type I error rate at 2-sided $\alpha = 0.05$ (see Figure 2). Analyses of other secondary and exploratory efficacy endpoints for the PC period will be performed at 2-sided $\alpha = 0.05$ without the multiplicity adjustment.

For crossover participants, efficacy data in EXT period will be summarized separately if needed. No statistical comparisons will be performed for EXT period.

Unless otherwise stated, the strata identified in the randomization process will be used in all efficacy analyses.

7.2. Efficacy Parameters

7.2.1. Hidradenitis Suppurativa Lesion Count

The HS lesions that will be counted in this study are outlined below:

- Abscess
- Inflammatory nodule
- Noninflammatory nodule
- Draining tunnel
- Nondraining tunnel

The distinct anatomical areas that will be considered for lesion count are outlined below:

- Face/neck
- Left axilla (armpit)/arm
- Right axilla (armpit)/arm
- Left chest/breast and inframammary area
- Right chest/breast and inframammary area
- Abdomen
- Trunk/torso (back)
- Left buttock (perianal)
- Right buttock (perianal)
- Left inguinocrural fold (groin)/thigh/leg

- Right inguinocrural fold (groin)/thigh/leg
- Genital area
- Other area(s)

7.2.1.1. Hidradenitis Suppurativa Clinical Response

The HiSCR is defined by the status of 3 types of lesions: abscess, inflammatory nodules, and draining tunnels. The definition of response requires all 3 criteria below to be met, relative to baseline:

- At least 50% reduction in AN count
- No increase in abscess count
- No increase in draining tunnel count

HiSCR75, HiSCR90, and HiSCR100 are respectively defined as at least a 75%, 90%, and 100% reduction from baseline in the total AN count, with no increase in abscess or draining tunnel count.

7.2.1.2. AN and ANdT Counts

The AN count is defined as the sum of abscess and inflammatory nodule counts. The AN count results will be used to calculate change in AN count relative to baseline as well as AN50, AN75, AN90, and AN100, defined respectively as at least a 50%, 75%, 90%, and 100% decrease in AN count relative to baseline.

The ANdT count is defined as the sum of abscess, inflammatory nodule, and draining tunnel counts. The ANdT count results will be used to calculate the change in ANdT count relative to baseline as well as ANdT50, ANdT75, ANdT90, and ANdT100, defined respectively as at least a 50%, 75%, 90%, and 100% decrease in ANdT count relative to baseline.

7.2.1.3. International Hidradenitis Suppurativa Severity Score System

The IHS4 score can be calculated as follows:

IHS4 (points) = (number of inflammatory nodules × 1) + (number of abscesses × 2) + (number of draining tunnels × 4)

The IHS4 disease categories are defined as follows:

- Mild disease: ≤ 3points
- Moderate disease: 4 to 10 points
- Severe disease: ≥ 11 points

IHS4-55, IHS4-75, IHS4-90, and IHS4-100 are respectively defined as at least a 55%, 75%, 90%, and 100% reduction from baseline in IHS4 score.

7.2.1.4. Flare Incidence

The definition of flare is outlined below:

- At least a 25% increase in the total AN count, relative to baseline

  and

- An increase of at least 2 in the total AN count, relative to baseline.

7.2.1.5. Lesion Rescue

In the event of acutely painful lesion(s) that may require intervention, the investigator or designee will perform Protocol-allowed rescue. The frequency and number of lesion rescue treatments will be assessed as endpoints. Rescued lesions will be counted as permanently present (ie, nonresponder lesions), even if they subside over the course of the study.

7.2.1.6. Hurley Stage

Each anatomical area will have the specific Hurley stage captured at scheduled visits. If more than 1 stage is present in the same area, the worst stage in that area should be documented. The participants will be assigned an overall Hurley stage classification corresponding to the stage of the worst involved anatomical area.

7.2.2. Patient-Reported Outcomes

7.2.2.1. Skin Pain NRS

Participants will answer the Skin Pain NRS question daily via eDiary from screening through Week 54 based on a 24-hour recall period.

The Skin Pain NRS scale ranges from 0 (no skin pain) to 10 (skin pain as bad as you can imagine) and evaluates the worst level of skin pain due to the participant's HS.

The Skin Pain NRS score for baseline will be determined by averaging the 7 daily NRS scores before randomization. The by-visit Skin Pain NRS score for postbaseline visits will be determined by averaging the 7 daily NRS scores before the visit day.

If 4 or more daily scores are missing (out of the 7), the Skin Pain NRS score at the visit will be set to missing.

Skin Pain NRS30 is defined as at least a 30% reduction and at least a 1-unit reduction from baseline in the Skin Pain NRS.

7.2.2.2. Analgesic Use

Analgesic (nonopioid and opioid) use is allowed during the study and will be reported as part of participant's daily diary. The first question will ask if the participant has taken any pain medication (nonopioid or opioid) for HS-related pain the past 24 hours; if yes, a second question will ask if the medication was an opioid.

The frequency of analgesic (nonopioid or opioid) use and opioid use for baseline will be determined by summing the 7 daily assessments before Day 1 (Day –7 to Day –1). The

frequency of analgesic (nonopioid or opioid) use and opioid use for postbaseline visits will be determined by summing the 7 daily assessments before the visit day.

If a participant has a frequency of > 0 analgesic (nonopioid or opioid) use/opioid use at a visit, then the participant is considered as having analgesic (nonopioid or opioid) use/opioid use at the visit.

7.2.2.3. Itch NRS

Participants will answer the Itch NRS question daily via eDiary from screening through Week 54 based on a 24-hour recall period.

The Itch NRS scale ranges from 0 (no itch) to 10 (worst itch imaginable).

The Itch NRS score for baseline will be determined by averaging the 7 daily Itch NRS scores before randomization. The by-visit Itch NRS score for postbaseline visits will be determined by averaging the 7 daily Itch NRS scores before the visit day. If 4 or more daily scores are missing (out of the 7), the Itch NRS score at the visit will be set to missing.

The most appropriate threshold for defining a clinically relevant, within-person response is a ≥ 4-point reduction relative to baseline (Kimball et al 2016).

Itch NRS30 is defined as at least a 30% reduction and at least a 1-unit reduction from baseline in the Itch NRS.

7.2.2.4. Hidradenitis Suppurativa Quality of Life

The HiSQoL is a 17-item, HS-specific, health-related, validated, QoL instrument with a 7-day recall period (Kirby et al 2020), and it will be used to assess HS symptoms and the impact of HS problems on QoL through 3 domains (symptoms, activities and adaptations, and psychosocial).

The scoring for each question is indicated in Table 5.

Table 5: Hidradenitis Suppurativa Quality of Life Scoring

Response	Score
Extremely	4
Very much	3
Moderately	2
Slightly	1
Not at all	0
Question unanswered	0
Unable to do due to my HS	4
I am not sexually active (Question 15)	0
I do not work or study (Question 17)	0

The HiSQoL total score will be calculated by summing the scores for each question. The sum will range between 0 and 68, with a higher score indicating higher HS-related impairment of QoL.

The HiSQoL can be analyzed under 3 subscales as indicated in Table 6.

Table 6: Hidradenitis Suppurativa Quality of Life Subscales

Section	Questions	Minimum Score	Maximum Score
Symptoms	8, 9, 10, 11	0	16
Psychosocial	6, 12, 13, 14, 15	0	20
Activities and adaptations	1, 2, 3, 4, 5, 7, 16, 17	0	36

Interpretation of incorrectly completed questionnaires are as follows:

- Unanswered questions are scored as 0.
- If 2 or more questions are unanswered, the questionnaire is not scored.
- If more than 1 response option is written for any item, the response option with the highest score should be recorded.
- If a response is written between 2 response options, the lower of the 2 score options should be recorded.
- When using subscales, if the answer to 1 item in a subscale is missing, that subscale should not be scored.

7.2.2.5. Dermatology Life Quality Index

The DLQI is a 10-question, validated questionnaire to measure how much the skin problem has affected the participant over the previous 7 days, across symptoms and feelings, daily activities, leisure, work and school, personal relations, and treatment (Finlay and Khan 1994).

The scoring of each question will be as follows: very much = 3; a lot = 2; a little = 1; not at all = 0; not relevant = 0.

The following imputation will be applied:

- If 1 question is left unanswered, it will be scored 0 and the scores will be summed and expressed as usual (ie, out of a maximum of 30).
- If 2 or more questions are left unanswered, the questionnaire will not be scored.
- If Question 7 is answered "yes," it will be scored 3. If Question 7 is answered "no" but then either "a lot" or "a little" is ticked, it will be scored 2 or 1, respectively. If "not relevant" is ticked, the score will be 0. If Question 7 is answered "no" but the second half is left incomplete, the score will remain 0.
- If 2 or more response options are ticked, the response option with the highest score will be recorded.
- If there is a response between 2 tick boxes, the lower of the 2 score options will be recorded.

The DLQI will be calculated by summing the score of each question resulting in a maximum of 30 and a minimum of 0. The higher the score, the more QoL is impaired.

A change from baseline in DLQI score of at least a 4-point reduction is considered clinically important (Basra et al 2015).

The meaning of total DLQI scores can be categorized as follows:

- 0 to 1 = No effect
- 2 to 5 = Small effect
- 6 to 10 = Moderate effect
- 11 to 20 = Very large effect
- 21 to 30 = Extremely large effect

The questionnaire will also be analyzed under 6 subscales as follows:

- Symptoms and feelings (Questions 1 and 2)
- Daily activities (Questions 3 and 4)
- Leisure (Questions 5 and 6)
- Work and school (Question 7)
- Personal relationships (Questions 8 and 9)
- Treatment (Question 10)

7.2.2.6. Functional Assessment of Chronic Illness Therapy – Fatigue Scale

The FACIT-F scale is a 13-item questionnaire originally designed to assess fatigue, over a 7-day recall, in patients with chronic disease. Each item uses a 5-point scale ranging from "4 = not at all" to "0 = very much." The total FACIT-F score ranges from 0 (maximum fatigue) to 52 (no fatigue), the higher the score less fatigue (better the quality of life). Lower scores are indicative of a greater impact on the participants' QoL (Cella et al 2019).

The FACIT-F Scale Questionnaire v4 will use the scoring algorithm presented by Cella et al 2002.

If greater than 50% of the 13 items (ie, ≥ 7 items) have missing responses, the total FACIT-F score will be set as missing.

A clinically important difference for the FACIT-F total score is defined as a 4-point improvement from baseline (Cella et al 2019); there is no HS-specific, clinically relevant threshold to define improvement.

7.2.2.7. Work Productivity and Activity Impairment – Hidradenitis Suppurativa

The WPAI-HS questionnaire is a validated instrument to measure impairments in both paid work and unpaid work. It measures absenteeism and presenteeism as well as impairments in unpaid activity because of HS and other health problems during the past 7 days. Absenteeism, presenteeism, and overall work impairment will be assessed only for employed participants

(Reilly et al 1993). Each item applies a standardized scoring that yields a final result expressed as a percentage, with higher percentage scores indicating greater impairment.

Questions:

- 1 = Currently employed
- 2 = Missed work hours, problem
- 3 = Missed work hours, other
- 4 = Hours actually worked
- 5 = How much problem affected productivity
- 6 = How much problem affected activities

Scores:

- Percent work time missed = 100 × [Q2 / (Q2 + Q4)]
- Percent impairment while working = 100 × (Q5 / 10)
- Percent overall work impairment = 100 × {[Q2 / (Q2 + Q4)] + ({1 – [Q2 / (Q2 + Q4)]} × (Q5 / 10))}
- Percent activity impairment = 100 × (Q6 / 10)

Missing values will not be imputed.

7.2.2.8. EQ-5D-5L Questionnaire

The EQ-5D-5L questionnaire is a standardized, validated instrument for use as a measure of health outcome (Herdman et al 2011). The EQ-5D-5L questionnaire will provide data for use in economic models and analyses, including developing health utilities or quality-adjusted life years. The EQ-5D-5L questionnaire consists of the following 2 sections: the EQ-5D descriptive system and the EQ VAS. The descriptive system comprises 5 dimensions: mobility, self-care, usual activities, pain/discomfort, and anxiety/depression. Each dimension has 5 levels: Level 1 = no problems, Level 2 = slight problems, Level 3 = moderate problems, Level 4 = severe problems, and Level 5 = extreme problems. The EQ VAS records the participant's self-rated health on a vertical VAS (0-100), where the endpoints are labeled "the best health you can imagine" (100 score) and "the worst health you can imagine" (0 score).

7.3. Efficacy Hypotheses

The primary alternative hypothesis is that each of the povorcitinib dose groups (75 and 45 mg) is superior to placebo at Week 12 in the proportion of participants who achieve HiSCR in participants with moderate to severe HS. Assuming p_T is the proportion of participants who achieve HiSCR at Week 12 in the povorcitinib dose group (75 or 45 mg) and p_C is the proportion of participants who achieve HiSCR at Week 12 in the placebo group, the primary hypotheses of the study are as follows:

- H_0 (null hypothesis): $p_T = p_C$
- H_A (alternative hypothesis): $p_T > p_C$

7.4. Analysis of the Primary Efficacy Parameter

The primary efficacy endpoint is the proportion of participants who achieve HiSCR at Week 12.

7.4.1. Primary Estimand

The primary estimand with the 5 attributes and strategies for addressing the defined intercurrent events is provided in Table 7.

Table 7: Primary Estimand

**Estimand Attribute**	**Definition**
**Treatment**	Each of the povorcitinib dose groups (75 and 45 mg) compared with placebo
**Population**	Participants with moderate to severe HS (as defined by study inclusion/exclusion criteria)
**Variable**	Achievement of HiSCR at Week 12, defined as at least a 50% reduction from baseline in the total AN count with no increase from baseline in abscess or draining tunnel count at Week 12
**Intercurrent Events**	**Strategies for Addressing Intercurrent Event**
1. Treatment discontinuation due to any reason	Participants who discontinue treatment for any reason at any time before Week 12 are considered to be nonresponders (composite strategy)
2. Initiation of systemic antibiotic rescue therapy or prohibited medication with effect on HS outcomes (eg, immunomodulatory biologics)	Participants who initiate systemic antibiotic rescue therapy or prohibited medication with effect on HS outcomes at any time before Week 12 are considered to be nonresponders (composite strategy)
3. Initiation of lesion rescue using Protocol-allowed interventions (injection with intralesional corticosteroid, or incision and drainage of the lesion)	The individual lesion(s) receiving rescue treatment will be counted as permanently present throughout the study
**Population-level summary**	Proportion of participants achieving HiSCR at Week 12 in each treatment group, the difference in HiSCR rate between each of the povorcitinib dose groups (75 and 45 mg) vs placebo

The primary analysis will be based on the ITT Population. The primary alternative hypothesis (superiority of each povorcitinib dose group [75 and 45 mg] compared with the placebo group) will be tested at a 2-sided $\alpha = 0.025$ level using the CMH test stratified by randomization stratification factors (baseline ANdT count [< 11 or ≥ 11] and previous treatment of HS with a biologic drug). A stratum-adjusted HiSCR rate difference between each of the povorcitinib dose groups (75 and 45 mg) versus placebo and the associated 95% Wald CI will be computed using Mantel-Haenszel stratum weights (Mantel and Haenszel 1959) and Sato's variance (Sato 1989). The stratified CMH p-value and common odds ratio with the 95% CI will be provided. The number of participants achieving HiSCR at Week 12 and the respective rates as well as the

2-sided 95% exact CIs based on the Clopper-Pearson method will also be reported by treatment group.

Based on the composite strategy for handling Intercurrent Event 1, treatment discontinuation, and Intercurrent Event 2, initiation of systemic antibiotic rescue therapy or prohibited mediation with effect on HS outcomes, participants who have any of the intercurrent events will be considered as nonresponders for all subsequent visits on or after the occurrence date of the intercurrent event. As well, participants who miss a postbaseline value at a visit (ie, true missing values due to data not collected) will be imputed as nonresponders for that visit only.

7.4.2. Sensitivity and Supportive Analyses of Primary Endpoint

A sensitivity analysis for the primary estimand will be conducted using the treatment strategy to address the intercurrent events with all data included regardless of occurrence of any intercurrent event, followed by multiple imputation of missing values. For each imputation iteration, a fully conditional specification method (van Buuren 2007) that assumes the existence of a joint distribution of all variables will be used to impute missing postbaseline values for HiSCR components, including abscess count, inflammatory nodule count, and draining tunnel count. For each of the 3 HiSCR components, a regression model including treatment group, randomization stratification factors, and baseline and scheduled postbaseline values up to Week 12 will be specified for the fully conditional specification method. After the missing HiSCR component values are imputed, the binary variables will be derived based on the definition. The stratified CMH test will be applied to each full dataset for the timepoint of interest. The imputation will be repeated 40 times with randomization seed 2024 to generate corresponding complete datasets in order to reflect the uncertainty around the true values, and the results will be combined for the inference using Rubin's rule.

Another sensitivity analysis will be conducted using a treatment strategy for intercurrent events following a tipping point analysis to examine the potential effects of missing data. The missing HiSCR at Week 12 in each treatment group will be replaced by a range of values from the most conservative scenario to the least conservative scenario. The most conservative scenario is that all the missing cases from participants in the povorcitinib dose group (75 or 45 mg) are nonresponders and all the missing cases from participants in the placebo group are responders, while the least conservative scenario is the reverse. For each scenario, comparisons between each of the povorcitinib dose groups (75 and 45 mg) versus placebo will be performed using the Fisher exact test.

In addition, the primary estimand will be performed using the same stratified CMH test in the PP Population as a supportive analysis.

7.4.3. Subgroup Analyses for Primary Endpoint

Subgroups will be formed based on the following participant characteristics and baseline variables for those participants whose data are available:

- Baseline ANdT count (< 11, ≥ 11)
- Previous treatment of HS with a biologic drug (yes, no)
- Age categories (18 to < 65 years, ≥ 65 years)

- Sex (female, male)
- Race (White, Black or African American, Asian, Other)
- Ethnicity (Hispanic or Latino, Not Hispanic or Latino)
- BMI categories (< 30 kg/m^2, 30 to < 40 kg/m^2, ≥ 40 kg/m^2)
- Geographic region (North America, Europe [UK included] and Australia, Japan)
- Baseline Hurley Stage (Stage II, Stage III)
- Baseline draining tunnel count (0, 1-2, ≥ 3)

The primary efficacy endpoint will be summarized using descriptive statistics for each subgroup defined above. For each subgroup category, the point estimate and 95% CI for treatment difference between each of the povorcitinib dose groups and placebo will be provided as well as presented in forest plots.

7.5. Analysis of the Secondary Efficacy Parameters

7.5.1. Analysis of the Key Secondary Efficacy Parameters

For the key secondary estimands, the 5 attributes and strategies for addressing the defined intercurrent events are provided in Table 8.

The key secondary analyses will be conducted based on the following populations:

- HiSCR75 at Week 12 and flare by Week 12 = ITT Population
- ≥ 3-point reduction in Skin Pain NRS at Week 12 = ITT Population with baseline Skin Pain NRS score ≥ 3

All key secondary efficacy endpoints will be analyzed using the stratified CMH method similar to that specified in the primary analysis. Similar to the primary analysis, participants who have true missing values due to data not collected will be imputed as nonresponders for that visit only.

The key secondary efficacy parameters will also be summarized for the following 2 subgroups:

- Baseline ANdT count (< 11, ≥ 11)
- Previous treatment of HS with a biologic drug (yes, no)

Table 8: Key Secondary Estimands

Estimand Attribute	Definition
Key secondary estimand for HiSCR75 at Week 12	
**Treatment**	Same as primary estimand
**Population**	Same as primary estimand
**Variable**	Achievement of HiSCR75 at Week 12, defined as at least a 75% reduction from baseline in the total AN count with no increase from baseline in abscess or draining tunnel count
**Intercurrent Events**	**Strategies for Addressing Intercurrent Events**
1. Treatment discontinuation for any reason	Same as primary estimand
2. Initiation of systemic antibiotic rescue therapy or prohibited medication with effect on HS outcomes (eg, immunomodulatory biologic)	Same as primary estimand
3. Initiation of lesion rescue using Protocol-allowed interventions (injection with intralesional corticosteroid, or incision and drainage of the lesion)	Same as primary estimand
**Population-level summary**	Proportion of participants achieving HiSCR75 at Week 12 in each treatment group, the difference in HiSCR75 rate between each of the povorcitinib dose groups (75 and 45 mg) vs placebo
Key secondary estimand for flare by Week 12	
**Treatment**	Same as primary estimand
**Population**	Same as primary estimand
**Variable**	Having flare at Week 12, defined as at least a 25% increase in the total AN count with a minimum increase of 2 relative to baseline
**Intercurrent Events**	**Strategies for Addressing Intercurrent Events**
1. Treatment discontinuation for any reason	Participants who discontinue treatment for any reason at any time before Week 12 are considered to have a flare (composite strategy)
2. Initiation of systemic antibiotic rescue therapy or prohibited medication with effect on HS outcomes (eg, immunomodulatory biologic)	Participants who initiate systemic antibiotic rescue therapy or prohibited medication with effect on HS outcomes at any time prior to Week 12 are considered to have a flare (composite strategy)
3. Initiation of lesion rescue using Protocol-allowed interventions (injection with intralesional corticosteroid, or incision and drainage of the lesion)	Same as primary estimand

Table 8: Key Secondary Estimands (Continued)

Estimand Attribute	Definition
**Population-level summary**	Proportion of participants with flare by Week 12 in each treatment group, the difference between each of the povorcitinib dose groups (75 and 45 mg) vs placebo
Key secondary estimand for Skin Pain Response at Week 12	
**Treatment**	Same as primary estimand
**Population**	Same as primary estimand
**Variable**	Achievement of Skin Pain Response at Week 12, defined as ≥ 3-point reduction from baseline in Skin Pain NRS at Week 12
**Intercurrent Events**	**Strategies for Addressing Intercurrent Events**
1. Treatment discontinuation for any reason	Same as primary estimand
2. Initiation of systemic antibiotic rescue therapy or prohibited medication with effect on HS outcomes (eg, immunomodulatory biologic)	Same as primary estimand
3. Initiation of lesion rescue using Protocol-allowed interventions (injection with intralesional corticosteroid, or incision and drainage of the lesion)	Same as primary estimand
**Population-level summary**	Proportion of participants achieving Skin Pain Response at Week 12 in each treatment group, the difference in response rate between each of the povorcitinib dose groups (75 and 45 mg) vs placebo

7.5.2. Analysis of Other Secondary Efficacy Parameters

In the PC period, all other secondary endpoints will be analyzed by treatment group based on the ITT Population. For categorical endpoints, summary statistics will include the number of participants, frequency, and percentages. A similar stratified CMH test as specified in the primary analysis will be used at Week 12, if applicable. The same strategies will be used to handle the intercurrent events followed by the nonresponder imputation method to impute missing values. For continuous efficacy endpoints, descriptive statistics will be provided for baseline, the actual measurements will be used at each visit, and the change and percentage change from baseline will be provided at each visit, if applicable. Summary statistics will include the number of participants, mean, standard deviation, median, minimum, and maximum. An analysis of covariance model with treatment group, randomization stratification factors, and baseline measurement will be fit at Week 12, if applicable. The estimated treatment difference between each of the povorcitinib dose groups (75 and 45 mg) and the placebo group, the associated 95% CIs, and the p-value will be presented.

As a supportive analysis, the continuous efficacy endpoints for the PC period may be analyzed using the MMRM to include all available data at postbaseline visits up to Week 12. Participants

who have a baseline value and at least 1 postbaseline value in the PC period will be included in the analysis. The MMRM will include the fixed effects of treatment group, randomization stratification factors, visit, treatment by visit interaction, and covariates of baseline measurements and baseline measurements by visit interaction. The variance-covariance matrix of the within-participant errors in the MMRM will be modeled as unstructured. The Kenward-Roger method will be used to estimate the degrees of freedom. Missing values will not be otherwise imputed using the MMRM approach. The least-squares means along with 95% CIs and the p-value for the difference in mean change from baseline at each visit between each of the povorcitinib dose groups (75 and 45 mg) and the placebo group from the MMRM will be provided.

Efficacy endpoints in the EXT period and the overall study period will be summarized using descriptive statistics based on the EXT-Evaluable Population and ITT Population, respectively. For categorical measurements, summary statistics will include sample size, frequency, and percentage. For continuous measurements, summary statistics will include sample size, mean, median, standard deviation, standard error of the mean, minimum, and maximum. Summary statistics for continuous measures will be provided for baseline, the actual measurements at each visit, and the change and percentage change from baseline at each visit, if applicable.

7.6. Analysis of Exploratory Efficacy Parameters

All exploratory efficacy variables will be summarized using descriptive statistics. For the binary endpoints, summary statistics will include the number of participants, frequency, and percentages. For continuous measurements, descriptive statistics will be provided for baseline, the actual measurements will be used at each visit, and the change and percentage change from baseline will be provided at each visit, if applicable. Summary statistics will include the number of participants, mean, standard deviation, median, minimum, and maximum.

8. SAFETY AND TOLERABILITY

Appendix A provides a list of data displays. Sample data displays are included in a separate document.

8.1. General Considerations

The analyses in this section will be provided for the Safety Population in the PC period and the overall study period, and the EXT-Evaluable Population in the EXT period, unless otherwise specified. Summary tables may be replaced with listings when appropriate. For instance, an AE frequency table may be replaced with a listing if it only contains a few unique PTs reported on relatively few participants.

8.2. Adverse Events

8.2.1. Adverse Event Definitions

A TEAE in the PC period is any AE, either reported for the first time or the worsening of a pre-existing event, occurring after the first dose of study drug in the PC period and until 30 days after the last dose of study drug for participants who do not enter the EXT period. For participants who enter the EXT period, the end date is 30 days after the last dose date in the PC period or the day before the first dose in the EXT period, whichever is first.

A TEAE in the EXT period is any AE, either reported for the first time or the worsening of a pre-existing event, occurring after the first dose of study drug in the EXT period and until 30 days after the last dose of study drug for participants who do not participate a separate EXT study. For participants who participate a separate EXT study, the end date is the last dose date in this study.

A TEAE in the overall study period is any AE, either reported for the first time or the worsening of a pre-existing event, occurring after the first dose of study drug in the PC period and until 30 days after the last dose of study drug during the overall study period for participants who do not participate a separate EXT study. For participants who participate a separate EXT study, the end date is the last dose date in this study.

Analysis of AEs (as discussed below) will be limited to TEAEs, but data listings will include all AEs regardless of their timing in relation to study drug administration. For purposes of analysis, all AEs will be considered TEAEs unless the AE can unequivocally be defined as not treatment-emergent.

Adverse events will be tabulated by MedDRA PT and SOC. Severity of AEs will be graded using the NCI CTCAE v5.0. The CTCAE reporting guidelines and grading details are available on the Cancer Therapy Evaluation Program website.

The subset of AEs considered by the investigator to be related to study drug will be considered to be treatment-related AEs. If the investigator does not specify the relationship of the AE to study drug, the AE will be considered to be treatment-related. The incidence of AEs and treatment-related AEs will be tabulated. In addition, serious TEAEs will also be tabulated.

[redacted]

[redacted]	[redacted]
[redacted]	[redacted]
[redacted]	[redacted]
[redacted]	[redacted]
[redacted]	[redacted]

[redacted]

[redacted]

[redacted]	[redacted]
[redacted]	[redacted]
[redacted]	[redacted]
[redacted]	[redacted]
[redacted]	[redacted]
[redacted]	[redacted]
[redacted]	[redacted]
[redacted]	[redacted]
[redacted]	[redacted]
[redacted]	[redacted]
[redacted]	[redacted]

8.2.4. Adverse Events Per 100 Patient-Years

In order to adjust for differences in participants' exposure in the study and potential dropout rates between the treatment groups, TEAEs per 100 PY will be calculated for each treatment group in the Safety Population for the overall study period as follows:

TEAEs per 100 PY = 100 × (number of participants with at least 1 TEAE / total PY)

where total PY are calculated as: the sum of study drug exposure for all participants in the treatment group / 365.25.

8.2.5. Adverse Event Summaries

Adverse events will be summarized by treatment group and overall povorcitinib dose groups for the Safety Population in the PC period and overall study period and for the EXT-Evaluable Population in the EXT period.

An overall summary of AEs will include the following:

- Number (%) of participants who had any TEAEs
- Number (%) of participants who had any Grade 3 or higher TEAEs
- Number (%) of participants who had any serious TEAEs
- Number (%) of participants who had any TEAEs related to study drug
- Number (%) of participants who had any fatal TEAEs
- Number (%) of participants who temporarily interrupted study drug because of TEAEs

- Number (%) of participants who permanently discontinued study drug because of TEAEs
- Number (%) of participants who had any treatment-emergent AESIs

In addition, the exposure-adjusted TEAE rates per 100 PY will be provided for each AE category described above in an overall summary of AEs.

The following summaries will be produced (if 10 or fewer participants appear in a table, a listing may be appropriate):

- Summary of TEAEs by MedDRA SOC and PT
- Summary of TEAEs by MedDRA PT in decreasing order of frequency
- Summary of TEAEs by MedDRA SOC, PT, and maximum severity
- Summary of TEAEs by MedDRA SOC, PT, and CTCAE grade category
- Summary of Grade 3 or higher TEAEs by MedDRA SOC and PT
- Summary of Grade 3 or higher TEAEs by MedDRA PT in decreasing order of frequency
- Summary of serious TEAEs by MedDRA SOC and PT
- Summary of serious TEAEs by MedDRA PT in decreasing order of frequency
- Summary of treatment-related TEAEs by MedDRA SOC and PT
- Summary of treatment-related TEAEs by MedDRA PT in decreasing order of frequency
- Summary of Grade 3 or higher treatment-related TEAEs by MedDRA SOC and PT
- Summary of treatment-related serious TEAEs by MedDRA SOC and PT
- Summary of TEAEs with a fatal outcome by MedDRA SOC and PT
- Summary of TEAEs leading to dose interruption by MedDRA SOC and PT
- Summary of TEAEs leading to discontinuation of study drug by MedDRA SOC and PT
- Summary of treatment-emergent ECIs (PT component) by category and MedDRA PT
- Summary of treatment-emergent ECIs (laboratory component) by laboratory parameters
- Summary of treatment-emergent AESIs by category and MedDRA PT
- Summary of treatment-emergent AESIs leading to dose interruption by category and MedDRA PT
- Summary of treatment-emergent AESIs leading to discontinuation of study drug by category and MedDRA PT

In addition, TEAE rates per 100 PY will be provided in a summary of TEAEs by MedDRA SOC and PT and a summary of treatment-emergent AESIs by category and MedDRA PT. Adjudicated cardiovascular events and other embolic and thrombotic events will be listed. The laboratory component and PT component of each ECI category will also be listed as applicable.

8.3. Clinical Laboratory Tests

8.3.1. Laboratory Value Definitions

For numeric laboratory results, the change and percentage change from baseline will be calculated. Baseline will be determined according to Section 4.1.3. If there are multiple values that meet the criteria for baseline, the value from the central laboratory has priority over the value from the local laboratory. Thereafter, additional rules may be provided after consultation with the medical monitor to delineate which value will be defined as baseline.

8.3.2. Laboratory Value Summaries

All test results and associated normal ranges from central laboratories will be reported in SI units. All tests with numeric values will have a unique unit per test. Any laboratory test results and associated normal ranges from local laboratories will be converted to SI units.

For numeric laboratory values, baseline value, postbaseline value, change from baseline and percent change from baseline will be summarized descriptively by visit. The number and percentage of participants with low, normal, and high laboratory values will be summarized by visit. The mean change from baseline will be plotted by visit. In addition, boxplots will be provided for hemoglobin, hematocrit, platelet count, hemoglobin A1c, high-sensitivity C-reactive protein, absolute lymphocyte count, absolute neutrophil count, white blood cell count, CPK, creatinine, AST, ALT, total bilirubin, cholesterol, HDL, LDL, and HDL/LDL ratio.

For test results that will be summarized with available normal ranges, the number and percentage of participants with laboratory values being low (but never high), normal, high (but never low), and both low and high will be calculated for each test. This shift summary will be produced for each test. The denominator for the percentage calculation will use the number of participants in the baseline category (ie, low, high, normal, missing) as the denominator for the percentage in each of the categories during the study.

Severity grades will be assigned to laboratory test values based on the numerical component of CTCAE v5. Shift tables will also be presented showing change in CTCAE grade from baseline to the worst grade postbaseline. Separate summaries for abnormally high and abnormally low laboratory values will be provided when the laboratory parameter has both high and low grading criteria. The denominator for the percentage calculation will be the number of participants in the baseline category. The number of participants who have worsening of laboratory abnormalities will be summarized by maximum severity.

8.3.3. Potentially Clinically Important Laboratory Values

Criteria for potentially clinically important laboratory values are listed in Table 11. The number and percentage of participants with postbaseline laboratory values that meet each criterion will be summarized at each scheduled visit by treatment group. Participants with potentially clinically important laboratory values will also be listed.

Table 11: Criteria for Potentially Clinically Important Laboratory Values

Laboratory Parameter	Grade (Criteria)
Hemoglobin	Grade 1 (100 g/L to < LLN)<br>Grade 2 (80 to < 100 g/L)<br>Grade 3 (< 80 g/L)
Lymphocytes	Grade 1 (0.8×10^9/L to < LLN)<br>Grade 2 (0.5 to < 0.8×10^9/L)<br>Grade 3 (0.2 to < 0.5×10^9/L)<br>Grade 4 (< 0.2×10^9/L)
Neutrophils	Grade 1 (1.5×10^9/L to < LLN)<br>Grade 2 (1.0 to < 1.5×10^9/L)<br>Grade 3 (0.5 to < 1.0×10^9/L)<br>Grade 4 (< 0.5×10^9/L)
Platelets	Grade 1 (75×10^9/L to < LLN)<br>Grade 2 (50 to < 75×10^9/L)<br>Grade 3 (25 to < 50×10^9/L)<br>Grade 4 (< 25×10^9/L)
Creatinine	Grade 1 (> ULN to 1.5 × ULN)<br>Grade 2 (> 1.5 to 3 × ULN or > 1.5 to 3 × baseline)<br>Grade 3 (> 3 to 6 × ULN or > 3 × baseline)<br>Grade 4 (> 6 × ULN)
CPK	Grade 1 (> ULN to 2.5 × ULN)<br>Grade 2 (> 2.5 to 5 × ULN)<br>Grade 3 (> 5 to 10 × ULN)<br>Grade 4 (> 10 × ULN)

8.3.4. Potential Hy's Law Events

The participants with elevated ALT or AST ≥ 3 × ULN range and alkaline phosphatase ≤ 2 × ULN range accompanied by total bilirubin > 2 × ULN range at the same visit will be listed and plotted by treatment group.

8.4. Vital Signs

Mean values at each scheduled visit for vital signs, including systolic blood pressure, diastolic blood pressure, pulse, respiratory rate, body temperature, weight, and BMI will be plotted.

Criteria for clinically notable vital sign abnormalities are defined in Table 12. The abnormal values for participants exhibiting clinically notable vital sign abnormalities will be listed along with their treatment group. Alert vital signs are defined as an absolute value outside the defined range and percentage change from baseline greater than 25%. Note that the definition of alert vital signs does not apply for body temperature, weight, or BMI. The abnormal values for participants exhibiting alert vital sign abnormalities will be listed.

Table 12: Criteria for Clinically Notable Vital Sign Abnormalities

Parameter	High Threshold	Low Threshold
Systolic blood pressure	≥ 155 mmHg	≤ 85 mmHg
Diastolic blood pressure	≥ 100 mmHg	≤ 40 mmHg
Pulse	≥100 bpm	≤ 45 bpm
Body temperature	≥ 38°C	≤ 35.5°C
Respiratory rate	≥ 24 breaths/min	≤ 8 breaths/min

8.5. Electrocardiograms

Twelve-lead ECGs including PR, QT, QRS, and QTcF intervals will be obtained for each participant at screening, Week 12, and Week 54. Values at each scheduled visit, change, and percentage change from baseline will be summarized for each ECG parameter. Baseline will be determined as the average of all nonmissing values before the first administration of povorcitinib or placebo.

9. INTERIM ANALYSES

No formal interim analysis is planned in this study.

10. CHANGES AND MODIFICATIONS TO THE ANALYSIS PLAN

All versions of the SAP are listed in Table 13.

Table 13: Statistical Analysis Plan Versions

SAP Version	Date
Original	17 DEC 2024

10.1. Changes to Protocol-Defined Analyses

Analyses for HiSCR100, IHS4-75, IHS4-90, and IHS4-100, as well as subgroup analyses for the key secondary endpoints have been added.

The following has been updated based on health authority feedback:

- In Section 3.2, changed multiplicity control method from Hochberg to gatekeeping in Step 2 and Step 3 of level of significance session.
- In Section 7.4.1, removed the phrase "treatment policy strategy" regarding the strategy for addressing initiation of lesion rescue intercurrent event.
- In Section 8.2.2 and 8.2.3, ECI and AESI categories have been updated.

10.2. Changes to the Statistical Analysis Plan

Not applicable.

11. REFERENCES

Basra MK, Salek MS, Camilleri L, Sturkey R, Finlay AY. Determining the minimal clinically important difference and responsiveness of the Dermatology Life Quality Index (DLQI): further data. Dermatology 2015;230:27-33.

Bretz F, Posch M, Glimm E, Klinglmueller F, Maurer W, Rohmeyer K. Graphical approaches for multiple comparison procedures using weighted Bonferroni, Simes, or parametric tests. Biom J 2011;53:894-913.

Cella D, Lai JS, Chang CH, Peterman A, Slavin M. Fatigue in cancer patients compared with fatigue in the general United States population. Cancer 2002;94:528-538.

Cella D, Wilson H, Shalhoub H, et al. Content validity and psychometric evaluation of Functional Assessment of Chronic Illness Therapy-Fatigue in patients with psoriatic arthritis. J Patient Rep Outcomes 2019;3:30.

Finlay AY, Khan GK. Dermatology Life Quality Index (DLQI) — a simple practical measure for routine clinical use. Clin Exp Dermatol 1994;19:210-216.

Herdman M, Gudex C, Lloyd A, et al. Development and preliminary testing of the new five-level version of EQ-5D (EQ-5D-5L). Qual Life Res 2011;20:1727-1736.

Kimball AB, Naegeli AN, Edson-Heredia E, et al. Psychometric properties of the Itch Numeric Rating Scale in patients with moderate-to-severe plaque psoriasis. Br J Dermatol 2016;175:157-162.

Kirby JS, Thorlacius L, Villumsen B, et al. The Hidradenitis Suppurativa Quality of Life (HiSQOL) score: development and validation of a measure for clinical trials. Br J Dermatol 2020;183:340-348.

Mantel N, Haenszel W. Statistical aspects of analysis of data from retrospective studies of disease. J Natl Cancer Inst 1959;22:719-748.

Reilly MC, Zbrozek AS, Dukes EM. The validity and reproducibility of a work productivity and activity impairment instrument. Pharmacoeconomics 1993;4:353-365.

Sato T. On the variance estimator of the Mantel-Haenszel risk difference [letter to the editor]. Biometrics 1989;45:1323-1324.

van Buuren S. Multiple imputation of discrete and continuous data by fully conditional specification. Stat Methods Med Res 2007;16:219-242.

APPENDIX A. PLANNED TABLES, FIGURES, AND LISTINGS

This appendix provides a list of the planned tables, figures, and listings for the Clinical Study Report. Shells are provided in a separate document for tables that are not in the Standard Safety Tables v2.0.

The lists of tables, figures, and listings are to be used as guideline. Modifications of the lists that do not otherwise affect the nature of the analysis will not warrant an amendment to the SAP.

Note that separate TFLs will be provided for the INCB 54707-301 and INCB 54707-302 studies.

Tables

Table No.	Title	Population	Standard
**Baseline and Demographic Characteristics**			
1.1.1	Analysis Populations	ITT	X
1.1.1.1	Screening Failures	All Screened	X
1.1.2.1	Summary of Participant Disposition in the PC Period	ITT	X
1.1.2.2	Summary of Participant Disposition in the EXT Period	EXT-Evaluable	X
1.1.3	Summary of Number of Participants Enrolled by Country and Site	ITT	X
1.1.4.1	Summary of Protocol Deviations During the PC Period	ITT	X
1.1.4.2	Summary of Protocol Deviations During the EXT Period	EXT-Evaluable	X
1.2.1	Summary of Demographics and Baseline Characteristics in the PC Period	ITT	X
1.2.2	Summary of Demographics and Baseline Characteristics in the EXT Period	EXT-Evaluable	X
1.3	Summary of Baseline Disease Characteristics and Disease History	ITT	X
1.4.1.1	Summary of Prior Medications	ITT	X
1.4.1.2	Summary of Prior Medications by Study-Specified Category, Type, and WHO Term	ITT	
1.4.1.3.1	Summary of Prior Oral Antibiotics or Biologics for HS - Response to Treatment by Type	ITT	
1.4.1.3.2	Summary of Prior Oral Antibiotics or Biologics for HS - Main Reason for Discontinuation by Type	ITT	
1.4.1.3.3	Summary of Prior Oral Antibiotics or Biologics for HS - Reason for Inadequate Response by Type	ITT	
1.4.1.4.1	Summary of Other Prior HS Therapy (Not Antibiotic/Biologic) - Response to Treatment by Type	ITT	
1.4.1.4.2	Summary of Other Prior HS Therapy (Not Antibiotic/Biologic) - Main Reason for Discontinuation by Type	ITT	
1.4.1.5.1	Summary of Prior JAK Inhibitor Therapy (Not for HS) - Response to Treatment	ITT	
1.4.1.5.2	Summary of Prior JAK Inhibitor Therapy (Not for HS) - Main Reason for Discontinuation	ITT	
1.4.1.6.1	Summary of Prior Biologic Therapy (Not for HS) - Response to Treatment	ITT	
1.4.1.6.2	Summary of Prior Biologic Therapy (Not for HS) - Main Reason for Discontinuation	ITT	
1.4.2.1.1	Summary of Concomitant Medications During the PC Period	ITT	X

Table No.	Title	Population	Standard
1.4.2.1.2	Summary of Concomitant Medications by Study-Specified Category, Type, and WHO Term During the PC Period	ITT	
1.4.2.2.1	Summary of Concomitant Medications During the EXT Period for Crossover Participants	EXT-Evaluable	X
1.4.2.2.2	Summary of Concomitant Medications by Study-Specified Category, Type, and WHO Term During the EXT Period for Crossover Participants	EXT-Evaluable	
1.4.2.3.1	Summary of Concomitant Medications During the Overall Study Period for Participants Who Took Only Povorcitinib	ITT	X
1.4.2.3.2	Summary of Concomitant Medications by Study-Specified Category, Type, and WHO Term During the Overall Study Period for Participants Who Took Only Povorcitinib	ITT	
1.5	Summary of General Medical History	ITT	X
**Efficacy**			
2.1.1	Summary and Analysis of Proportion of Participants Achieving HiSCR by Visit During the PC Period	ITT	
2.1.2.1	Summary and Analysis of Proportion of Participants Achieving HiSCR by Visit During the PC Period - Supportive Analysis	PP	
2.1.2.2	Summary and Analysis of Proportion of Participants Achieving HiSCR by Visit During the PC Period - Multiple Imputation Sensitivity Analysis	ITT	
2.1.2.3	Summary and Analysis of Proportion of Participants Achieving HiSCR by Visit During the PC Period - Tipping Point Sensitivity Analysis	ITT	
2.1.3.x[a]	Summary and Analysis of Proportion of Participants Achieving HiSCR by Visit During the PC Period - Subgroup Analysis	ITT	
2.1.4.1	Summary of Proportion of Participants Achieving HiSCR by Visit During the Overall Study Period for Participants Who Took Only Povorcitinib	ITT	
2.1.4.2	Summary of Proportion of Participants Achieving HiSCR by Visit During the EXT Period for Crossover Participants	EXT-Evaluable	
2.2.1.1.1	Summary and Analysis of Proportion of Participants Achieving HiSCR75 by Visit During the PC Period	ITT	
2.2.1.1.2.x[b]	Summary and Analysis of Proportion of Participants Achieving HiSCR75 by Visit During the PC Period - Subgroup Analysis	ITT	
2.2.1.2	Summary of Proportion of Participants Achieving HiSCR75 by Visit During the Overall Study Period for Participants Who Took Only Povorcitinib	ITT	
2.2.1.3	Summary of Proportion of Participants Achieving HiSCR75 by Visit During the EXT Period for Crossover Participants	EXT-Evaluable	
2.2.2.1.1	Summary and Analysis of Proportion of Participants With Flare by Week 12	ITT	
2.2.2.1.2.x[b]	Summary and Analysis of Proportion of Participants With Flare by Week 12 - Subgroup Analysis	ITT	
2.2.2.2.2	Summary of Proportion of Participants With Flare by Week 24 for Participants Who Took Only Povorcitinib	ITT	
2.2.2.2.3	Summary of Proportion of Participants With Flare by Week 24 During the EXT Period for Crossover Participants	EXT-Evaluable	
2.2.2.3.2	Summary of Proportion of Participants With Flare by Week 54 for Participants Who Took Only Povorcitinib	ITT	

Table No.	Title	Population	Standard
2.2.2.3.3	Summary of Proportion of Participants With Flare by Week 54 During the EXT Period for Crossover Participants	EXT-Evaluable	
2.2.3.1.1.1	Summary and Analysis of Proportion of Participants With a >= 3-Point Decrease in Skin Pain NRS Score by Visit During the PC Period (With Baseline Skin Pain NRS Score >= 3)	ITT	
2.2.3.1.1.2.x[b]	Summary and Analysis of Proportion of Participants With a >= 3-Point Decrease in Skin Pain NRS Score by Visit During the PC Period (With Baseline Skin Pain NRS Score >= 3) - Subgroup Analysis	ITT	
2.2.3.1.2	Summary Proportion of Participants With a >= 3-Point Decrease in Skin Pain NRS Score by Visit During the Overall Study Period for Participants Who Took Only Povorcitinib (With Baseline Skin Pain NRS Score >= 3)	ITT	
2.2.3.1.3	Summary of Proportion of Participants With a >= 3-Point Decrease in Skin Pain NRS Score by Visit During the EXT Period for Crossover Participants (With Baseline Skin Pain NRS Score >= 3)	EXT-Evaluable	
2.2.3.2.1.1	Summary and Analysis of Proportion of Participants Achieving Skin Pain NRS30 by Visit During the PC Period (With Baseline Skin Pain NRS Score >= 3)	ITT	
2.2.3.2.1.2.x[b]	Summary and Analysis of Proportion of Participants Achieving Skin Pain NRS30 by Visit During the PC Period (With Baseline Skin Pain NRS Score >= 3) - Subgroup Analysis	ITT	
2.2.3.2.2	Summary of Proportion of Participants Achieving Skin Pain NRS30 by Visit During the Overall Study Period for Participants Who Took Only Povorcitinib (With Baseline Skin Pain NRS Score >= 3)	ITT	
2.2.3.2.3	Summary of Proportion of Participants Achieving Skin Pain NRS30 by Visit During the EXT Period for Crossover Participants (With Baseline Skin Pain NRS Score >= 3)	EXT-Evaluable	
2.2.4.1	Summary and Analysis of Proportion of Participants With at Least a 4-Point Increase in FACIT-F Score by Visit During the PC Period	ITT	
2.2.4.2	Summary of Proportion of Participants With at Least a 4-Point Increase in FACIT-F Score by Visit During the Overall Study Period for Participants Who Took Only Povorcitinib	ITT	
2.2.4.3	Summary of Proportion of Participants With at Least a 4-Point Increase in FACIT-F Score by Visit During the EXT Period for Crossover Participants	EXT-Evaluable	
2.2.5.1	Summary and Analysis of DLQI Score by Visit During the PC Period	ITT	
2.2.5.2	Summary of DLQI Score by Visit During the Overall Study Period for Participants Who Took Only Povorcitinib	ITT	
2.2.5.3	Summary of DLQI Score by Visit During the EXT Period for Crossover Participants	EXT-Evaluable	
2.2.6.1	Summary of Proportion of Participants With at Least a 4-Point Decrease From Baseline in DLQI Score by Visit During the PC Period	ITT	
2.2.6.2	Summary of Proportion of Participants With at Least a 4-Point Decrease From Baseline in DLQI Score by Visit During the Overall Study Period for Participants Who Took Only Povorcitinib	ITT	
2.2.6.3	Summary of Proportion of Participants With at Least a 4-Point Decrease From Baseline in DLQI Score by Visit During the EXT Period for Crossover Participants	EXT-Evaluable	

Table No.	Title	Population	Standard
2.2.7.1	Summary of Abscess Count by Visit During the PC Period	ITT	
2.2.7.2	Summary of Abscess Count by Visit During the Overall Study Period for Participants Who Took Only Povorcitinib	ITT	
2.2.7.3	Summary of Abscess Count by Visit During the EXT Period for Crossover Participants	EXT-Evaluable	
2.2.8.1	Summary of Inflammatory Nodule Count by Visit During the PC Period	ITT	
2.2.8.2	Summary of Inflammatory Nodule Count by Visit During the Overall Study Period for Participants Who Took Only Povorcitinib	ITT	
2.2.8.3	Summary of Inflammatory Nodule Count by Visit During the EXT Period for Crossover Participants	EXT-Evaluable	
2.2.9.1	Summary of Draining Tunnel Count by Visit During the PC Period	ITT	
2.2.9.2	Summary of Draining Tunnel Count by Visit During the Overall Study Period for Participants Who Took Only Povorcitinib	ITT	
2.2.9.3	Summary of Draining Tunnel Count by Visit During the EXT Period for Crossover Participants	EXT-Evaluable	
2.2.10.1	Summary of Total Abscess and Inflammatory Nodule Count by Visit During the PC Period	ITT	
2.2.10.2	Summary of Total Abscess and Inflammatory Nodule Count by Visit During the Overall Study Period for Participants Who Took Only Povorcitinib	ITT	
2.2.10.3	Summary of Total Abscess and Inflammatory Nodule Count by Visit During the EXT Period for Crossover Participants	EXT-Evaluable	
2.2.11	Summary of Proportion of Participants Achieving Maintenance of HiSCR by Visit During the EXT Period for Participants Who Took Only Povorcitinib	EXT-Evaluable	
2.2.12	Summary of Proportion of Participants Achieving Maintenance of HiSCR75 by Visit During the EXT Period for Participants Who Took Only Povorcitinib	EXT-Evaluable	
2.3.1.1	Summary of Proportion of Participants Achieving HiSCR90 by Visit During the PC Period	ITT	
2.3.1.2	Summary of Proportion of Participants Achieving HiSCR90 by Visit During the Overall Study Period for Participants Who Took Only Povorcitinib	ITT	
2.3.1.3	Summary of Proportion of Participants Achieving HiSCR90 by Visit During the EXT Period for Crossover Participants	EXT-Evaluable	
2.3.2.1	Summary of Proportion of Participants Achieving HiSCR100 by Visit During the PC Period	ITT	
2.3.2.2	Summary of Proportion of Participants Achieving HiSCR100 by Visit During the Overall Study Period for Participants Who Took Only Povorcitinib	ITT	
2.3.2.3	Summary of Proportion of Participants Achieving HiSCR100 by Visit During the EXT Period for Crossover Participants	EXT-Evaluable	
2.3.3.1	Summary of Proportion of Participants With Total Abscess and Inflammatory Nodule Count of 0, 1, or 2 by Visit During the PC Period	ITT	
2.3.3.2	Summary of Proportion of Participants With Total Abscess and Inflammatory Nodule Count of 0, 1, or 2 by Visit During the Overall Study Period for Participants Who Took Only Povorcitinib	ITT	

Table No.	Title	Population	Standard
2.3.4.1	Summary of Proportion of Participants Achieving AN50 by Visit During the PC Period	ITT	
2.3.4.2	Summary of Proportion of Participants Achieving AN50 by Visit During the Overall Study Period for Participants Who Took Only Povorcitinib	ITT	
2.3.5.1	Summary of Proportion of Participants Achieving AN75 by Visit During the PC Period	ITT	
2.3.5.2	Summary of Proportion of Participants Achieving AN75 by Visit During the Overall Study Period for Participants Who Took Only Povorcitinib	ITT	
2.3.6.1	Summary of Proportion of Participants Achieving AN90 by Visit During the PC Period	ITT	
2.3.6.2	Summary of Proportion of Participants Achieving AN90 by Visit During the Overall Study Period for Participants Who Took Only Povorcitinib	ITT	
2.3.7.1	Summary of Proportion of Participants Achieving AN100 by Visit During the PC Period	ITT	
2.3.7.2	Summary of Proportion of Participants Achieving AN100 by Visit During the Overall Study Period for Participants Who Took Only Povorcitinib	ITT	
2.3.8.1	Summary of Proportion of Participants Achieving ANdT50 by Visit During the PC Period	ITT	
2.3.8.2	Summary of Proportion of Participants Achieving ANdT50 by Visit During the Overall Study Period for Participants Who Took Only Povorcitinib	ITT	
2.3.9.1	Summary of Proportion of Participants Achieving ANdT75 by Visit During the PC Period	ITT	
2.3.9.2	Summary of Proportion of Participants Achieving ANdT75 by Visit During the Overall Study Period for Participants Who Took Only Povorcitinib	ITT	
2.3.10.1	Summary of Proportion of Participants Achieving ANdT90 by Visit During the PC Period	ITT	
2.3.10.2	Summary of Proportion of Participants Achieving ANdT90 by Visit During the Overall Study Period for Participants Who Took Only Povorcitinib	ITT	
2.3.11.1	Summary of Proportion of Participants Achieving ANdT100 by Visit During the PC Period	ITT	
2.3.11.2	Summary of Proportion of Participants Achieving ANdT100 by Visit During the Overall Study Period for Participants Who Took Only Povorcitinib	ITT	
2.3.12.1	Summary of Proportion of Participants With at Least a 1-Unit Decrease From Baseline in Draining Tunnel Count by Visit During the PC Period	ITT	
2.3.12.2	Summary of Proportion of Participants With at Least a 1-Unit Decrease From Baseline in Draining Tunnel Count by Visit During the Overall Study Period for Participants Who Took Only Povorcitinib	ITT	
2.3.13.1	Summary of ANdT Count by Visit During the PC Period	ITT	
2.3.13.2	Summary of ANdT Count by Visit During the Overall Study Period for Participants Who Took Only Povorcitinib	ITT	

Table No.	Title	Population	Standard
2.3.14.1	Summary of IHS4 Score by Visit During the PC Period	ITT	
2.3.14.2	Summary of IHS4 Score by Visit During the Overall Study Period for Participants Who Took Only Povorcitinib	ITT	
2.3.15.1	Summary of Proportion of Participants Achieving IHS4-55 by Visit During the PC Period	ITT	
2.3.15.2	Summary of Proportion of Participants Achieving IHS4-55 by Visit During the Overall Study Period for Participants Who Took Only Povorcitinib	ITT	
2.3.16.1	Summary of Proportion of Participants Achieving IHS4-75 by Visit During the PC Period	ITT	
2.3.16.2	Summary of Proportion of Participants Achieving IHS4-75 by Visit During the Overall Study Period for Participants Who Took Only Povorcitinib	ITT	
2.3.17.1	Summary of Proportion of Participants Achieving IHS4-90 by Visit During the PC Period	ITT	
2.3.17.2	Summary of Proportion of Participants Achieving IHS4-90 by Visit During the Overall Study Period for Participants Who Took Only Povorcitinib	ITT	
2.3.18.1	Summary of Proportion of Participants Achieving IHS4-100 by Visit During the PC Period	ITT	
2.3.18.2	Summary of Proportion of Participants Achieving IHS4-100 by Visit During the Overall Study Period for Participants Who Took Only Povorcitinib	ITT	
2.3.19.1	Summary of Proportion of Participants With IHS4 Disease Category by Visit During the PC Period	ITT	
2.3.19.2	Summary of Proportion of Participants With IHS4 Disease Category by Visit During the Overall Study Period for Participants Who Took Only Povorcitinib	ITT	
2.3.20.1	Summary of Proportion of Participants With HS Flares by Visit During the PC Period	ITT	
2.3.20.2	Summary of Proportion of Participants With HS Flares by Visit During the Overall Study Period for Participants Who Took Only Povorcitinib	ITT	
2.3.21	Summary of Proportion of Participants With at Least 1 HS Flare During the EXT Period for Participants Who Took Only Povorcitinib	ITT	
2.3.22.1	Summary of Flares During the PC Period	ITT	
2.3.22.2	Summary of Flares During the EXT Period for Participants Who Took Only Povorcitinib	EXT-Evaluable	
2.3.23.1	Summary of Skin Pain NRS Score by Visit During the PC Period	ITT	
2.3.23.2	Summary of Skin Pain NRS Score by Visit During the Overall Study Period for Participants Who Took Only Povorcitinib	ITT	
2.3.24.1	Summary of Proportion of Participants With a >= 4-Point Decrease in Skin Pain NRS Score by Visit During the PC Period	ITT	
2.3.24.2	Summary of Proportion of Participants With a >= 4-Point Decrease in Skin Pain NRS Score by Visit During the Overall Study Period for Participants Who Took Only Povorcitinib	ITT	
2.3.25.1	Summary of Proportion of Participants With Analgesic Use by Visit During the PC Period	ITT	

Table No.	Title	Population	Standard
2.3.25.2	Summary of Proportion of Participants With Analgesic Use by Visit During the Overall Study Period for Participants Who Took Only Povorcitinib	ITT	
2.3.26.1	Summary of Proportion of Participants With Opioid Use by Visit During the PC Period	ITT	
2.3.26.2	Summary of Proportion of Participants With Opioid Use by Visit During the Overall Study Period for Participants Who Took Only Povorcitinib	ITT	
2.3.27.1	Summary of Proportion of Participants Requiring Rescue Lesional Treatment During the PC Period	ITT	
2.3.27.2	Summary of Proportion of Participants Requiring Rescue Lesional Treatment During the Overall Study Period for Participants Who Took Only Povorcitinib	ITT	
2.3.28.1	Summary of Proportion of Participants Requiring Systemic Antibiotic Rescue Therapy During the PC Period	ITT	
2.3.28.2	Summary of Proportion of Participants Requiring Systemic Antibiotic Rescue Therapy During the Overall Study Period for Participants Who Took Only Povorcitinib	ITT	
2.3.29.1	Summary of Itch NRS Score by Visit During the PC Period	ITT	
2.3.29.2	Summary of Itch NRS Score by Visit During the Overall Study Period for Participants Who Took Only Povorcitinib	ITT	
2.3.30.1	Summary of Proportion of Participants Achieving Itch NRS30 During the PC Period (With Baseline Itch NRS Score >= 3)	ITT	
2.3.30.2	Summary of Proportion of Participants Achieving Itch NRS30 During the Overall Study Period for Participants Who Took Only Povorcitinib (With Baseline Itch NRS Score >= 3)	ITT	
2.3.31.1	Summary of Proportion of Participants With a >= 4-Point Decrease in Itch NRS Score by Visit During the PC Period	ITT	
2.3.31.2	Summary of Proportion of Participants With a >= 4-Point Decrease in Itch NRS Score by Visit During the Overall Study Period for Participants Who Took Only Povorcitinib	ITT	
2.3.32.1	Summary of HiSQoL Score by Visit During the PC Period	ITT	
2.3.32.2	Summary of HiSQoL Score by Visit During the Overall Study Period for Participants Who Took Only Povorcitinib	ITT	
2.3.33.1	Summary of FACIT-F Score by Visit During the PC Period	ITT	
2.3.33.2	Summary of FACIT-F Score by Visit During the Overall Study Period for Participants Who Took Only Povorcitinib	ITT	
2.3.34.1	Summary of WPAI-HS Score by Visit During the PC Period	ITT	
2.3.34.2	Summary of WPAI-HS Score by Visit During the Overall Study Period for Participants Who Took Only Povorcitinib	ITT	
2.3.35.1	Summary of EQ-5D-5L VAS Score by Visit During the PC Period	ITT	
2.3.35.2	Summary of EQ-5D-5L VAS Score by Visit During the Overall Study Period for Participants Who Took Only Povorcitinib	ITT	
2.3.36.1	Summary of EQ-5D-5L Questionnaire by Visit During the PC Period	ITT	
2.3.36.2	Summary of EQ-5D-5L Questionnaire by Visit During the Overall Study Period for Participants Who Took Only Povorcitinib	ITT	
2.3.37.1	Summary of Time to the First Flare During the PC Period	ITT	
2.3.37.2	Summary of Time to the First Flare During the EXT Period for Participants Who Took Only Povorcitinib	EXT-Evaluable	

Table No.	Title	Population	Standard
**Safety**			
3.1.1.1	Summary of Study Drug Exposure During the PC Period	Safety	X
3.1.1.2	Summary of Study Drug Exposure During the Overall Study Period for Participants Who Took Only Povorcitinib	Safety	X
3.1.1.3	Summary of Study Drug Exposure During the EXT Period for Crossover Participants	EXT-Evaluable	X
3.1.2.1	Summary of Study Drug Compliance During the PC Period	Safety	X
3.1.2.2	Summary of Study Drug Compliance During the Overall Study Period for Participants Who Took Only Povorcitinib	Safety	X
3.1.2.3	Summary of Study Drug Compliance During the EXT Period for Crossover Participants	EXT-Evaluable	X
3.2.1.1	Overall Summary of Treatment-Emergent Adverse Events During the PC Period	Safety	X
3.2.1.2	Overall Summary of Treatment-Emergent Adverse Events During the Overall Study Period for Participants Who Took Only Povorcitinib	Safety	X
3.2.1.3	Overall Summary of Treatment-Emergent Adverse Events During the EXT Period for Crossover Participants	EXT-Evaluable	X
3.2.2.1	Summary of Treatment-Emergent Adverse Events by MedDRA System Organ Class and Preferred Term During the PC Period	Safety	X
3.2.2.2	Summary of Treatment-Emergent Adverse Events by MedDRA System Organ Class and Preferred Term During the Overall Study Period for Participants Who Took Only Povorcitinib	Safety	X
3.2.2.3	Summary of Treatment-Emergent Adverse Events by MedDRA System Organ Class and Preferred Term During the EXT Period for Crossover Participants	EXT-Evaluable	X
3.2.3.1	Summary of Treatment-Emergent Adverse Events by MedDRA Preferred Term in Decreasing Order of Frequency During the PC Period	Safety	X
3.2.3.2	Summary of Treatment-Emergent Adverse Events by MedDRA Preferred Term in Decreasing Order of Frequency During the Overall Study Period for Participants Who Took Only Povorcitinib	Safety	X
3.2.3.3	Summary of Treatment-Emergent Adverse Events by MedDRA Preferred Term in Decreasing Order of Frequency During the EXT Period for Crossover Participants	EXT-Evaluable	X
3.2.4.1	Summary of Treatment-Emergent Adverse Events by MedDRA System Organ Class, Preferred Term, and Maximum Severity During the PC Period	Safety	X
3.2.4.2	Summary of Treatment-Emergent Adverse Events by MedDRA System Organ Class, Preferred Term, and Maximum Severity During the Overall Study Period for Participants Who Took Only Povorcitinib	Safety	X
3.2.4.3	Summary of Treatment-Emergent Adverse Events by MedDRA System Organ Class, Preferred Term, and Maximum Severity During the EXT Period for Crossover Participants	EXT-Evaluable	X
3.2.5.1	Summary of Treatment-Emergent Adverse Events by MedDRA System Organ Class, Preferred Term, and CTCAE Grade Category During the PC Period	Safety	X

Table No.	Title	Population	Standard
3.2.5.2	Summary of Treatment-Emergent Adverse Events by MedDRA System Organ Class, Preferred Term, and CTCAE Grade Category During the Overall Study Period for Participants Who Took Only Povorcitinib	Safety	X
3.2.5.3	Summary of Treatment-Emergent Adverse Events by MedDRA System Organ Class, Preferred Term, and CTCAE Grade Category During the EXT Period for Crossover Participants	EXT-Evaluable	X
3.2.6.1	Summary of Grade 3 or Higher Treatment-Emergent Adverse Events by MedDRA System Organ Class and Preferred Term During the PC Period	Safety	X
3.2.6.2	Summary of Grade 3 or Higher Treatment-Emergent Adverse Events by MedDRA System Organ Class and Preferred Term During the Overall Study Period for Participants Who Took Only Povorcitinib	Safety	X
3.2.6.3	Summary of Grade 3 or Higher Treatment-Emergent Adverse Events by MedDRA System Organ Class and Preferred Term During the EXT Period for Crossover Participants	EXT-Evaluable	X
3.2.7.1	Summary of Grade 3 or Higher Treatment-Emergent Adverse Events by MedDRA Preferred Term in Decreasing Order of Frequency During the PC Period	Safety	X
3.2.7.2	Summary of Grade 3 or Higher Treatment-Emergent Adverse Events by MedDRA Preferred Term in Decreasing Order of Frequency During the Overall Study Period for Participants Who Took Only Povorcitinib	Safety	X
3.2.7.3	Summary of Grade 3 or Higher Treatment-Emergent Adverse Events by MedDRA Preferred Term in Decreasing Order of Frequency During the EXT Period for Crossover Participants	EXT-Evaluable	X
3.2.8.1	Summary of Serious Treatment-Emergent Adverse Events by MedDRA System Organ Class and Preferred Term During the PC Period	Safety	X
3.2.8.2	Summary of Serious Treatment-Emergent Adverse Events by MedDRA System Organ Class and Preferred Term During the Overall Study Period for Participants Who Took Only Povorcitinib	Safety	X
3.2.8.3	Summary of Serious Treatment-Emergent Adverse Events by MedDRA System Organ Class and Preferred Term During the EXT Period for Crossover Participants	EXT-Evaluable	X
3.2.9.1	Summary of Serious Treatment-Emergent Adverse Events by MedDRA Preferred Term in Decreasing Order of Frequency During the PC Period	Safety	X
3.2.9.2	Summary of Serious Treatment-Emergent Adverse Events by MedDRA Preferred Term in Decreasing Order of Frequency During the Overall Study Period for Participants Who Took Only Povorcitinib	Safety	X
3.2.9.3	Summary of Serious Treatment-Emergent Adverse Events by MedDRA Preferred Term in Decreasing Order of Frequency During the EXT Period for Crossover Participants	EXT-Evaluable	X
3.2.10.1	Summary of Treatment-Related Treatment-Emergent Adverse Events by MedDRA System Organ Class and Preferred Term During the PC Period	Safety	X
3.2.10.2	Summary of Treatment-Related Treatment-Emergent Adverse Events by MedDRA System Organ Class and Preferred Term During the Overall Study Period for Participants Who Took Only Povorcitinib	Safety	X

Table No.	Title	Population	Standard
3.2.10.3	Summary of Treatment-Related Treatment-Emergent Adverse Events by MedDRA System Organ Class and Preferred Term During the EXT Period for Crossover Participants	EXT-Evaluable	X
3.2.11.1	Summary of Treatment-Related Treatment-Emergent Adverse Events by MedDRA Preferred Term in Decreasing Order of Frequency During the PC Period	Safety	X
3.2.11.2	Summary of Treatment-Related Treatment-Emergent Adverse Events by MedDRA Preferred Term in Decreasing Order of Frequency During the Overall Study Period for Participants Who Took Only Povorcitinib	Safety	X
3.2.11.3	Summary of Treatment-Related Treatment-Emergent Adverse Events by MedDRA Preferred Term in Decreasing Order of Frequency During the EXT Period for Crossover Participants	EXT-Evaluable	X
3.2.12.1	Summary of Grade 3 or Higher Treatment-Related Treatment-Emergent Adverse Events by MedDRA System Organ Class and Preferred Term During the PC Period	Safety	X
3.2.12.2	Summary of Grade 3 or Higher Treatment-Related Treatment-Emergent Adverse Events by MedDRA System Organ Class and Preferred Term During the Overall Study Period for Participants Who Took Only Povorcitinib	Safety	X
3.2.12.3	Summary of Grade 3 or Higher Treatment-Related Treatment-Emergent Adverse Events by MedDRA System Organ Class and Preferred Term During the EXT Period for Crossover Participants	EXT-Evaluable	X
3.2.13.1	Summary of Treatment-Related Serious Treatment-Emergent Adverse Events by MedDRA System Organ Class and Preferred Term During the PC Period	Safety	X
3.2.13.2	Summary of Treatment-Related Serious Treatment-Emergent Adverse Events by MedDRA System Organ Class and Preferred Term During the Overall Study Period for Participants Who Took Only Povorcitinib	Safety	X
3.2.13.3	Summary of Treatment-Related Serious Treatment-Emergent Adverse Events by MedDRA System Organ Class and Preferred During the EXT Period for Crossover Participants	EXT-Evaluable	X
3.2.14.1	Summary of Treatment-Emergent Adverse Events With a Fatal Outcome by MedDRA System Organ Class and Preferred Term During the PC Period	Safety	X
3.2.14.2	Summary of Treatment-Emergent Adverse Events With a Fatal Outcome by MedDRA System Organ Class and Preferred Term During the Overall Study Period for Participants Who Took Only Povorcitinib	Safety	X
3.2.14.3	Summary of Treatment-Emergent Adverse Events With a Fatal Outcome by MedDRA System Organ Class and Preferred Term During the EXT Period for Crossover Participants	EXT-Evaluable	X
3.2.15.1	Summary of Treatment-Emergent Adverse Events Leading to Dose Interruption by MedDRA System Organ Class and Preferred Term During the PC Period	Safety	X
3.2.15.2	Summary of Treatment-Emergent Adverse Events Leading to Dose Interruption by MedDRA System Organ Class and Preferred Term During the Overall Study Period for Participants Who Took Only Povorcitinib	Safety	X

Table No.	Title	Population	Standard
3.2.15.3	Summary of Treatment-Emergent Adverse Events Leading to Dose Interruption by MedDRA System Organ Class and Preferred Term During the EXT Period for Crossover Participants	EXT-Evaluable	X
3.2.16.1	Summary of Treatment-Emergent Adverse Events Leading to Discontinuation of Study Drug by MedDRA System Organ Class and Preferred Term During the PC Period	Safety	X
3.2.16.2	Summary of Treatment-Emergent Adverse Events Leading to Discontinuation of Study Drug by MedDRA System Organ Class and Preferred Term During the Overall Study Period for Participants Who Took Only Povorcitinib	Safety	X
3.2.16.3	Summary of Treatment-Emergent Adverse Events Leading to Discontinuation of Study Drug by MedDRA System Organ Class and Preferred Term During the EXT Period for Crossover Participants	EXT-Evaluable	X
3.2.17.1	Summary of Treatment-Emergent Adverse Events of Special Interest by Category and MedDRA Preferred Term During the PC Period	Safety	X
3.2.17.2	Summary of Treatment-Emergent Adverse Events of Special Interest by Category and MedDRA Preferred Term During the Overall Study Period for Participants Who Took Only Povorcitinib	Safety	X
3.2.17.3	Summary of Treatment-Emergent Adverse Events of Special Interest by Category and MedDRA Preferred Term During the EXT Period for Crossover Participants	EXT-Evaluable	X
3.2.18.1	Summary of Treatment-Emergent Adverse Events of Special Interest Leading to Dose Interruption by Category and MedDRA Preferred Term During the PC Period	Safety	X
3.2.18.2	Summary of Treatment-Emergent Adverse Events of Special Interest Leading to Dose Interruption by Category and MedDRA Preferred Term During the Overall Study Period for Participants Who Took Only Povorcitinib	Safety	X
3.2.18.3	Summary of Treatment-Emergent Adverse Events of Special Interest Leading to Dose Interruption by Category and MedDRA Preferred Term During the EXT Period for Crossover Participants	EXT-Evaluable	X
3.2.19.1	Summary of Treatment-Emergent Adverse Events of Special Interest Leading to Discontinuation of Study Drug by Category and MedDRA Preferred Term During the PC Period	Safety	X
3.2.19.2	Summary of Treatment-Emergent Adverse Events of Special Interest Leading to Discontinuation of Study Drug by Category and MedDRA Preferred Term During the Overall Study Period for Participants Who Took Only Povorcitinib	Safety	X
3.2.19.3	Summary of Treatment-Emergent Adverse Events of Special Interest Leading to Discontinuation of Study Drug by Category and MedDRA Preferred Term During the EXT Period for Crossover Participants	EXT-Evaluable	X
3.2.20.1	Summary of Treatment-Emergent Events of Clinical Interest (PT Component) by Category and MedDRA Preferred Term During the PC Period	Safety	X
3.2.20.2	Summary of Treatment-Emergent Events of Clinical Interest (PT Component) by Category and MedDRA Preferred Term During the Overall Study Period for Participants Who Took Only Povorcitinib	Safety	X
3.2.20.3	Summary of Treatment-Emergent Events of Clinical Interest (PT Component) by Category and MedDRA Preferred Term During the EXT Period for Crossover Participants	EXT-Evaluable	X

Table No.	Title	Population	Standard
3.2.21.1	Summary of Treatment-Emergent Events of Clinical Interest (Laboratory Component) by Laboratory Parameters During the PC Period	Safety	X
3.2.21.2	Summary of Treatment-Emergent Events of Clinical Interest (Laboratory Component) by Laboratory Parameters During the Overall Study Period for Participants Who Took Only Povorcitinib	Safety	X
3.2.21.3	Summary of Treatment-Emergent Events of Clinical Interest (Laboratory Component) by Laboratory Parameters During the EXT Period for Crossover Participants	EXT-Evaluable	X
3.2.22	Overall Summary of Exposure-Adjusted Incidence Rates of Treatment-Emergent Adverse Events During the Overall Study Period	Safety	X
3.2.23	Summary of Exposure-Adjusted Incidence Rates of Treatment-Emergent Adverse Events by MedDRA System Organ Class and Preferred Term During the Overall Study Period	Safety	X
3.2.24	Summary of Exposure-Adjusted Incidence Rates of Treatment-Emergent Adverse Events of Special Interest by Category and MedDRA Preferred Term During the Overall Study Period	Safety	X
3.3.1.1.1	Summary of Laboratory Values During the PC Period - Hematology	Safety	X
3.3.1.1.2	Summary of Laboratory Values During the Overall Study Period for Participants Who Took Only Povorcitinib - Hematology	Safety	X
3.3.1.1.3	Summary of Laboratory Values During the EXT Period for Crossover Participants - Hematology	EXT-Evaluable	X
3.3.1.2.1	Summary of Laboratory Values During the PC Period - Chemistry	Safety	X
3.3.1.2.2	Summary of Laboratory Values During the Overall Study Period for Participants Who Took Only Povorcitinib - Chemistry	Safety	X
3.3.1.2.3	Summary of Laboratory Values During the EXT Period for Crossover Participants - Chemistry	EXT-Evaluable	X
3.3.1.3.1	Summary of Laboratory Values During the PC Period - Lipid Panel	Safety	X
3.3.1.3.2	Summary of Laboratory Values During the Overall Study Period for Participants Who Took Only Povorcitinib - Lipid Panel	Safety	X
3.3.1.3.3	Summary of Laboratory Values During the EXT Period for Crossover Participants - Lipid Panel	EXT-Evaluable	X
3.3.1.4.1	Summary of Laboratory Values During the PC Period - Inflammation Markers	Safety	X
3.3.1.4.2	Summary of Laboratory Values During the Overall Study Period for Participants Who Took Only Povorcitinib - Inflammation Markers	Safety	X
3.3.1.4.3	Summary of Laboratory Values During the EXT Period for Crossover Participants - Inflammation Markers	EXT-Evaluable	X
3.3.2.1.1	Shift Summary of Hematology Laboratory Values - to the Worst Abnormal Value During the PC Period	Safety	X
3.3.2.1.2	Shift Summary of Hematology Laboratory Values - to the Worst Abnormal Value During the Overall Study Period for Participants Who Took Only Povorcitinib	Safety	X
3.3.2.1.3	Shift Summary of Hematology Laboratory Values - to the Worst Abnormal Value During the EXT Period for Crossover Participants	EXT-Evaluable	X
3.3.2.2.1	Shift Summary of Chemistry Laboratory Values - to the Worst Abnormal Value During the PC Period	Safety	X

Table No.	Title	Population	Standard
3.3.2.2.2	Shift Summary of Chemistry Laboratory Values - to the Worst Abnormal Value During the Overall Study Period for Participants Who Took Only Povorcitinib	Safety	X
3.3.2.2.3	Shift Summary of Chemistry Laboratory Values - to the Worst Abnormal Value During the EXT Period for Crossover Participants	EXT-Evaluable	X
3.3.2.3.1	Shift Summary of Lipid Panel Laboratory Values - to the Worst Abnormal Value During the PC Period	Safety	X
3.3.2.3.2	Shift Summary of Lipid Panel Laboratory Values - to the Worst Abnormal Value During the Overall Study Period for Participants Who Took Only Povorcitinib	Safety	X
3.3.2.3.3	Shift Summary of Lipid Panel Laboratory Values - to the Worst Abnormal Value During the EXT Period for Crossover Participants	EXT-Evaluable	X
3.3.3.1.1	Shift Summary of Hematology Laboratory Values in CTCAE Grade - to the Worst Grade Abnormal Value During the PC Period	Safety	X
3.3.3.1.2	Shift Summary of Hematology Laboratory Values in CTCAE Grade - to the Worst Grade Abnormal Value During the Overall Study Period for Participants Who Took Only Povorcitinib	Safety	X
3.3.3.1.3	Shift Summary of Hematology Laboratory Values in CTCAE Grade - to the Worst Grade Abnormal Value During the EXT Period for Crossover Participants	EXT-Evaluable	X
3.3.3.2.1	Shift Summary of Chemistry Laboratory Values in CTCAE Grade - to the Worst Grade Abnormal Value During the PC Period	Safety	X
3.3.3.2.2	Shift Summary of Chemistry Laboratory Values in CTCAE Grade - to the Worst Grade Abnormal Value During the Overall Study Period for Participants Who Took Only Povorcitinib	Safety	X
3.3.3.2.3	Shift Summary of Chemistry Laboratory Values in CTCAE Grade - to the Worst Grade Abnormal Value During the EXT Period for Crossover Participants	EXT-Evaluable	X
3.3.3.3.1	Shift Summary of Lipid Panel Laboratory Values in CTCAE Grade - to the Worst Grade Abnormal Value During the PC Period	Safety	X
3.3.3.3.2	Shift Summary of Lipid Panel Laboratory Values in CTCAE Grade - to the Worst Grade Abnormal Value During the Overall Study Period for Participants Who Took Only Povorcitinib	Safety	X
3.3.3.3.3	Shift Summary of Lipid Panel Laboratory Values in CTCAE Grade - to the Worst Grade Abnormal Value During the EXT Period for Crossover Participants	EXT-Evaluable	X
3.3.4.1.1	Treatment-Emergent Worsening of Laboratory Abnormalities - Hematology During the PC Period	Safety	X
3.3.4.1.2	Treatment-Emergent Worsening of Laboratory Abnormalities - Hematology During the Overall Study Period for Participants Who Took Only Povorcitinib	Safety	X
3.3.4.1.3	Treatment-Emergent Worsening of Laboratory Abnormalities - Hematology During the EXT Period for Crossover Participants	EXT-Evaluable	X
3.3.4.2.1	Treatment-Emergent Worsening of Laboratory Abnormalities - Chemistry During the PC Period	Safety	X
3.3.4.2.2	Treatment-Emergent Worsening of Laboratory Abnormalities - Chemistry During the Overall Study Period for Participants Who Took Only Povorcitinib	Safety	X
3.3.4.2.3	Treatment-Emergent Worsening of Laboratory Abnormalities - Chemistry During the EXT Period for Crossover Participants	EXT-Evaluable	X

Table No.	Title	Population	Standard
3.3.4.3.1	Treatment-Emergent Worsening of Laboratory Abnormalities - Lipid Panel During the PC Period	Safety	X
3.3.4.3.2	Treatment-Emergent Worsening of Laboratory Abnormalities - Lipid Panel During the Overall Study Period for Participants Who Took Only Povorcitinib	Safety	X
3.3.4.3.3	Treatment-Emergent Worsening of Laboratory Abnormalities - Lipid Panel During the EXT Period for Crossover Participants	EXT-Evaluable	X
3.3.5.1	Summary of Potential Clinically Important Laboratory Values by Visit During the PC Period	Safety	X
3.3.5.2	Summary of Potential Clinically Important Laboratory Values by Visit During the Overall Study Period for Participants Who Took Only Povorcitinib	Safety	X
3.3.5.3	Summary of Potential Clinically Important Laboratory Values by Visit During the EXT Period for Crossover Participants	EXT-Evaluable	X
3.5.1.1.1	Summary of PR Interval (ms) From 12-Lead ECG During the PC Period	Safety	X
3.5.1.1.2	Summary of PR Interval (ms) From 12-Lead ECG During the Overall Study Period for Participants Who Took Only Povorcitinib	Safety	X
3.5.1.1.3	Summary of PR Interval (ms) From 12-Lead ECG During the EXT Period for Crossover Participants	EXT-Evaluable	X
3.5.1.2.1	Summary of QRS Interval (ms) From 12-Lead ECG During the PC Period	Safety	X
3.5.1.2.2	Summary of QRS Interval (ms) From 12-Lead ECG During the Overall Study Period for Participants Who Took Only Povorcitinib	Safety	X
3.5.1.2.3	Summary of QRS Interval (ms) From 12-Lead ECG During the EXT Period for Crossover Participants	EXT-Evaluable	X
3.5.1.3.1	Summary of QT Interval (ms) From 12-Lead ECG During the PC Period	Safety	X
3.5.1.3.2	Summary of QT Interval (ms) From 12-Lead ECG During the Overall Study Period for Participants Who Took Only Povorcitinib	Safety	X
3.5.1.3.3	Summary of QT Interval (ms) From 12-Lead ECG During the EXT Period for Crossover Participants	EXT-Evaluable	X
3.5.1.4.1	Summary of QTcF Interval (ms) From 12-Lead ECG During the PC Period	Safety	X
3.5.1.4.2	Summary of QTcF Interval (ms) From 12-Lead ECG During the Overall Study Period for Participants Who Took Only Povorcitinib	Safety	X
3.5.1.4.3	Summary of QTcF Interval (ms) From 12-Lead ECG During the EXT Period for Crossover Participants	EXT-Evaluable	X
3.5.1.5.1	Summary of Heart Rate (beats/min) From 12-Lead ECG During the PC Period	Safety	X
3.5.1.5.2	Summary of Heart Rate (beats/min) From 12-Lead ECG During the Overall Study Period for Participants Who Took Only Povorcitinib	Safety	X
3.5.1.5.3	Summary of Heart Rate (beats/min) From 12-Lead ECG During the EXT Period for Crossover Participants	EXT-Evaluable	X
3.5.1.6.1	Summary of Clinically Significant ECG Abnormalities During the PC Period	Safety	X
3.5.1.6.2	Summary of Clinically Significant ECG Abnormalities During the Overall Study Period for Participants Who Took Only Povorcitinib	Safety	X

Table No.	Title	Population	Standard
3.5.1.6.3	Summary of Clinically Significant ECG Abnormalities During the EXT Period for Crossover Participants	EXT-Evaluable	X

[a] x = 1 to 10 for each subgroup described in Section 7.4.3.
[b] x = 1 to 2 for each subgroup described in Section 7.5.1.

Figures

Figure No.	Title
4.1.1	Proportion of Participants Achieving HiSCR by Visit and Treatment Group During the PC Period Using Nonresponder Imputation
4.1.2	Forest Plot of Participants Achieving HiSCR at Week 12 by Subgroup
4.1.3	Proportion of Participants Achieving HiSCR by Visit and Treatment Group During the Overall Study Period
4.2.1.1	Proportion of Participants Achieving HiSCR75 by Visit and Treatment Group During the PC Period Using Nonresponder Imputation
4.2.1.2	Proportion of Participants Achieving HiSCR75 by Visit and Treatment Group During the Overall Study Period
4.2.2.1	Mean (+/- SE) Percent Change From Baseline in AN Count by Visit and Treatment Group During the PC Period
4.2.2.2	Mean (+/- SE) Percent Change From Baseline in AN Count by Visit and Treatment Group During the Overall Study Period
4.2.3.1	Proportion of Participants With >= 3-Point Decrease in Skin Pain NRS Score by Visit During the PC Period (With Baseline Skin Pain NRS Score >= 3)
4.2.3.2	Proportion of Participants With >= 3-Point Decrease in Skin Pain NRS Score by Visit During the Overall Study Period (With Baseline Skin Pain NRS Score >= 3)
4.2.4.1	Summary and Analysis of Proportion of Participants Achieving Skin Pain NRS30 by Visit During the PC Period (With Baseline Skin Pain NRS Score >= 3)
4.2.4.2	Summary of Proportion of Participants Achieving Skin Pain NRS30 by Visit During the Overall Study Period (With Baseline Skin Pain NRS Score >= 3)
4.2.5.1	Mean (+/- SE) Percent Change From Baseline in Skin Pain NRS Score by Visit and Treatment Group During the PC Period
4.2.5.2	Mean (+/- SE) Percent Change From Baseline in Skin Pain NRS Score by Visit and Treatment Group During the Overall Study Period
4.2.6.1	Proportion of Participants With at Least a 4-Point Increase in FACIT-F Score by Visit During PC Period
4.2.6.2	Proportion of Participants With at Least a 4-Point Increase in FACIT-F Score by Visit During the Overall Study Period
4.2.7.1	Mean (+/- SE) Change From Baseline in FACIT-F Scale Score by Visit and Treatment Group During the PC Period
4.2.7.2	Mean (+/- SE) Change From Baseline in FACIT-F Scale Score by Visit and Treatment Group During the Overall Study Period
4.2.8.1	Mean (+/- SE) Change From Baseline in DLQI Total Score by Visit and Treatment Group During the PC Period
4.2.8.2	Mean (+/- SE) Change From Baseline in DLQI Total Score by Visit and Treatment Group During the Overall Study Period
4.2.9.1	Proportion of Participants Achieving HiSCR90 by Visit and Treatment Group During the PC Period Using Nonresponder Imputation
4.2.9.2	Proportion of Participants Achieving HiSCR90 by Visit and Treatment Group During the Overall Study Period

Figure No.	Title
4.2.10.1	Proportion of Participants Achieving HiSCR100 by Visit and Treatment Group During the PC Period Using Nonresponder Imputation
4.2.10.2	Proportion of Participants Achieving HiSCR100 by Visit and Treatment Group During the Overall Study Period
4.6.1.1.1	Mean Laboratory Data Change From Baseline by Visit and Treatment Group During the PC Period - Hematology
4.6.1.1.2	Mean Laboratory Data Change From Baseline by Visit and Treatment Group During the Overall Study Period for Participants Who Took Only Povorcitinib - Hematology
4.6.1.1.3	Mean Laboratory Data Change From Baseline by Visit and Treatment Group During the EXT Period for Crossover Participants - Hematology
4.6.1.2.1	Mean Laboratory Data Change From Baseline by Visit and Treatment Group During the PC Period - Chemistry
4.6.1.2.2	Mean Laboratory Data Change From Baseline by Visit and Treatment Group During the Overall Study Period for Participants Who Took Only Povorcitinib - Chemistry
4.6.1.2.3	Mean Laboratory Data Change From Baseline by Visit and Treatment Group During the EXT Period for Crossover Participants - Chemistry
4.6.1.3.1	Mean Laboratory Data Change From Baseline by Visit and Treatment Group During the PC Period - Lipid Panel
4.6.1.3.2	Mean Laboratory Data Change From Baseline by Visit and Treatment Group During the Overall Study Period for Participants Who Took Only Povorcitinib - Lipid Panel
4.6.1.3.3	Mean Laboratory Data Change From Baseline by Visit and Treatment Group During the EXT Period for Crossover Participants - Lipid Panel
4.6.2.1.1	Box-and-Whisker Plot of Select Laboratory Values by Visit and Treatment Group During the PC Period
4.6.2.1.2	Box-and-Whisker Plot of Select Laboratory Values by Visit and Treatment Group During the Overall Study Period for Participants Who Took Only Povorcitinib
4.6.2.1.3	Box-and-Whisker Plot of Select Laboratory Values by Visit and Treatment Group During the EXT Period for Crossover Participants
4.6.2.2.1	Box-and-Whisker Plot of Change From Baseline in Select Laboratory Values by Visit and Treatment Group During the PC Period
4.6.2.2.2	Box-and-Whisker Plot of Change From Baseline in Select Laboratory Values by Visit and Treatment Group During the Overall Study Period for Participants Who Took Only Povorcitinib
4.6.2.2.3	Box-and-Whisker Plot of Change From Baseline in Select Laboratory Values by Visit and Treatment Group During the EXT Period for Crossover Participants
4.6.2.3.1	Box-and-Whisker Plot of Percent Change From Baseline in Select Laboratory Values by Visit and Treatment Group During the PC Period
4.6.2.3.2	Box-and-Whisker Plot of Percent Change From Baseline in Select Laboratory Values by Visit and Treatment Group During the Overall Study Period for Participants Who Took Only Povorcitinib
4.6.2.3.3	Box-and-Whisker Plot of Percent Change From Baseline in Select Laboratory Values by Visit and Treatment Group During the EXT Period for Crossover Participants
4.6.2.4.1	Box-and-Whisker Plot of Lipid Panel Values by Visit and Treatment Group During the PC Period
4.6.2.4.2	Box-and-Whisker Plot of Lipid Panel Values by Visit and Treatment Group During the Overall Study Period for Participants Who Took Only Povorcitinib
4.6.2.4.3	Box-and-Whisker Plot of Lipid Panel Values by Visit and Treatment Group During the EXT Period for Crossover Participants
4.6.2.5.1	Box-and-Whisker Plot of Change From Baseline in Lipid Panel Values by Visit and Treatment Group During the PC Period
4.6.2.5.2	Box-and-Whisker Plot of Change From Baseline in Lipid Panel Values by Visit and Treatment Group During the Overall Study Period for Participants Who Took Only Povorcitinib

Figure No.	Title
4.6.2.5.3	Box-and-Whisker Plot of Change From Baseline in Lipid Panel Values by Visit and Treatment Group During the EXT Period for Crossover Participants
4.6.2.6.1	Box-and-Whisker Plot of Percent Change From Baseline in Lipid Panel Values by Visit and Treatment Group During the PC Period
4.6.2.6.2	Box-and-Whisker Plot of Percent Change From Baseline in Lipid Panel Values by Visit and Treatment Group During the Overall Study Period for Participants Who Took Only Povorcitinib
4.6.2.6.3	Box-and-Whisker Plot of Percent Change From Baseline in Lipid Panel Values by Visit and Treatment Group During the EXT Period for Crossover Participants
4.6.3	Plot of Potential Hy's Law Events
4.7.1.1	Mean and 95% Confidence Interval Vital Sign Values by Visit and Treatment Group During the PC Period - Systolic Blood Pressure
4.7.1.2	Mean and 95% Confidence Interval Vital Sign Values by Visit and Treatment Group During the Overall Study Period for Participants Who Took Only Povorcitinib - Systolic Blood Pressure
4.7.1.3	Mean and 95% Confidence Interval Vital Sign Values by Visit During the EXT Period for Crossover Participants - Systolic Blood Pressure
4.7.2.1	Mean and 95% Confidence Interval Vital Sign Values by Visit and Treatment Group During the PC Period - Diastolic Blood Pressure
4.7.2.2	Mean and 95% Confidence Interval Vital Sign Values by Visit and Treatment Group During the Overall Study Period for Participants Who Took Only Povorcitinib - Diastolic Blood Pressure
4.7.2.3	Mean and 95% Confidence Interval Vital Sign Values by Visit During the EXT Period for Crossover Participants - Diastolic Blood Pressure
4.7.3.1	Mean and 95% Confidence Interval Vital Sign Values by Visit and Treatment Group During the PC Period - Pulse
4.7.3.2	Mean and 95% Confidence Interval Vital Sign Values by Visit and Treatment Group During the Overall Study Period for Participants Who Took Only Povorcitinib - Pulse
4.7.3.3	Mean and 95% Confidence Interval Vital Sign Values by Visit During the EXT Period for Crossover Participants - Pulse
4.7.4.1	Mean and 95% Confidence Interval Vital Sign Values by Visit and Treatment Group During the PC Period - Respiratory Rate
4.7.4.2	Mean and 95% Confidence Interval Vital Sign Values by Visit and Treatment Group During the Overall Study Period for Participants Who Took Only Povorcitinib - Respiratory Rate
4.7.4.3	Mean and 95% Confidence Interval Vital Sign Values by Visit During the EXT Period for Crossover Participants - Respiratory Rate
4.7.5.1	Mean and 95% Confidence Interval Vital Sign Values by Visit and Treatment Group During the PC Period - Body Temperature
4.7.5.2	Mean and 95% Confidence Interval Vital Sign Values by Visit and Treatment Group During the Overall Study Period for Participants Who Took Only Povorcitinib - Body Temperature
4.7.5.3	Mean and 95% Confidence Interval Vital Sign Values by Visit During the EXT Period for Crossover Participants - Body Temperature
4.7.6.1	Mean and 95% Confidence Interval Vital Sign Values by Visit and Treatment Group During the PC Period - Body Weight
4.7.6.2	Mean and 95% Confidence Interval Vital Sign Values by Visit and Treatment Group During the Overall Study Period for Participants Who Took Only Povorcitinib - Body Weight
4.7.6.3	Mean and 95% Confidence Interval Vital Sign Values by Visit During the EXT Period for Crossover Participants - Body Weight
4.7.7.1	Mean and 95% Confidence Interval Vital Sign Values by Visit and Treatment Group During the PC Period - BMI
4.7.7.2	Mean and 95% Confidence Interval Vital Sign Values by Visit and Treatment Group During the Overall Study Period for Participants Who Took Only Povorcitinib - BMI

Figure No.	Title
4.7.7.3	Mean and 95% Confidence Interval Vital Sign Values by Visit During the EXT Period for Crossover Participants - BMI

Listings

Listing No.	Title
2.1.1.1	Participant Enrollment and Disposition Status for the PC Period
2.1.1.2	Participant Enrollment and Disposition Status for the EXT Period
2.2.1	Participant Inclusion and Exclusion Criteria Violations
2.2.2	Protocol Deviations
2.3	Analysis Populations
2.4.1	Demographic and Baseline Characteristics
2.4.2	Disease History
2.4.3	Prior Therapy for Hidradenitis Suppurativa
2.4.4	Medical History
2.4.5	Prior and Concomitant Medication
2.5.1	Study Drug Compliance
2.5.2	Study Drug Administration
2.6.1	Abscess Count, Inflammatory Nodule Count, Draining Tunnel Count
2.6.2	AN Count, ANdT Count, HiSCR, HiSCR75, HiSCR90, HiSCR100, and Flare
2.6.3	IHS4 Score
2.6.4	Analgesic Use
2.6.5	Rescue Lesional Treatment
2.6.6	HiSQoL Total and Subscale Scores
2.6.7	DLQI Total and Subscale Scores
2.6.8	Skin Pain NRS Score
2.6.9	Itch NRS Score
2.6.10	FACIT-F Scale Score
2.6.11	WPAI-HS Score
2.6.12	EQ-5D-5L Score and EQ VAS
2.6.13	Hurley Stage
2.7.1	Adverse Events
2.7.2	Serious Adverse Events
2.7.3	Grade 3 and Higher Adverse Events
2.7.4	Treatment-Related Adverse Events
2.7.5	Fatal Adverse Events
2.7.6	Adverse Events Leading to Dose Interruption
2.7.7	Adverse Events Leading to Discontinuation of Study Drug
2.7.8	Adverse Events of Special Interest
2.7.9.1.1	Events of Clinical Interest (Laboratory Component) - Increased Serum Creatinine and Renal Disfunction
2.7.9.1.2	Events of Clinical Interest (Preferred Term Component) - Increased Serum Creatinine and Renal Disfunction
2.7.9.2.1	Events of Clinical Interest (Laboratory Component) - Hepatic Events and Increased Hepatic Transaminases

Listing No.	Title
2.7.9.2.2	Events of Clinical Interest (Preferred Term Component) - Hepatic Events and Increased Hepatic Transaminases
2.7.9.3.1	Events of Clinical Interest (Laboratory Component) - Increased Creatine Phosphokinase
2.7.9.3.2	Events of Clinical Interest (Preferred Term Component) - Increased Creatine Phosphokinase
2.7.9.4	Events of Clinical Interest - Acne
2.7.10	Adjudicated Cardiovascular Events
2.8.1.1	Clinical Laboratory Values - Hematology
2.8.1.2	Clinical Laboratory Values - Chemistry
2.8.1.3	Clinical Laboratory Values - Lipid Panel
2.8.1.4	Clinical Laboratory Values - Urinalysis
2.8.1.5	Clinical Laboratory Values - Inflammation Markers
2.8.1.6	Clinical Laboratory Values - Nicotine Metabolite Screen
2.8.2	Abnormal Clinical Laboratory Values
2.8.3	Potentially Clinically Important Laboratory Values
2.8.4	Potential Hy's Law Events
2.9.1	Vital Signs
2.9.2	Abnormal Vital Sign Values
2.9.3	Alert Vital Sign Values
2.10.1	12-Lead ECG Values

[redacted]

[redacted]
[redacted]
[redacted]
[redacted]
[redacted]
[redacted]
[redacted]
[redacted]
[redacted]
[redacted]
[redacted]
[redacted]
[redacted]
[redacted]
[redacted]
[redacted]
[redacted]
[redacted]
[redacted]
[redacted]
[redacted]
[redacted]
[redacted]
[redacted]
[redacted]
[redacted]
[redacted]
[redacted]
[redacted]
[redacted]
[redacted]
[redacted]
[redacted]
[redacted]

[redacted]

[redacted]
[redacted]
[redacted]
[redacted]
[redacted]
[redacted]
[redacted]
[redacted]
[redacted]
[redacted]
[redacted]
[redacted]
[redacted]
[redacted]
[redacted]
[redacted]
[redacted]
[redacted]
[redacted]
[redacted]
[redacted]
[redacted]
[redacted]
[redacted]
[redacted]
[redacted]
[redacted]
[redacted]
[redacted]
[redacted]
[redacted]
[redacted]
[redacted]
[redacted]
[redacted]
[redacted]
[redacted]
[redacted]
[redacted]

[redacted]

[redacted]
[redacted]
[redacted]
[redacted]
[redacted]
[redacted]
[redacted]
[redacted]

[redacted]

[redacted]
[redacted]
[redacted]
[redacted]
[redacted]
[redacted]
[redacted]
[redacted]
[redacted]
[redacted]
[redacted]
[redacted]
[redacted]
[redacted]
[redacted]
[redacted]
[redacted]
[redacted]
[redacted]
[redacted]
[redacted]
[redacted]

[redacted]

[redacted]
[redacted]
[redacted]
[redacted]
[redacted]
[redacted]
[redacted]
[redacted]
[redacted]
[redacted]
[redacted]
[redacted]
[redacted]
[redacted]
[redacted]
[redacted]
[redacted]
[redacted]
[redacted]
[redacted]
[redacted]
[redacted]
[redacted]
[redacted]
[redacted]
[redacted]
[redacted]
[redacted]
[redacted]
[redacted]
[redacted]
[redacted]
[redacted]
[redacted]
[redacted]
[redacted]
[redacted]
[redacted]
[redacted]

[redacted]

[redacted]

[redacted]
[redacted]
[redacted]
[redacted]
[redacted]
[redacted]
[redacted]
[redacted]
[redacted]
[redacted]
[redacted]
[redacted]
[redacted]
[redacted]
[redacted]
[redacted]
[redacted]
[redacted]
[redacted]
[redacted]
[redacted]
[redacted]
[redacted]
[redacted]
[redacted]
[redacted]
[redacted]
[redacted]
[redacted]
[redacted]
[redacted]
[redacted]
[redacted]
[redacted]
[redacted]
[redacted]
[redacted]
[redacted]
[redacted]
[redacted]

[redacted]

[redacted]
[redacted]
[redacted]
[redacted]
[redacted]
[redacted]
[redacted]
[redacted]
[redacted]
[redacted]
[redacted]
[redacted]
[redacted]
[redacted]
[redacted]
[redacted]
[redacted]
[redacted]
[redacted]
[redacted]
[redacted]
[redacted]
[redacted]
[redacted]
[redacted]
[redacted]
[redacted]
[redacted]
[redacted]
[redacted]
[redacted]
[redacted]
[redacted]
[redacted]
[redacted]
[redacted]
[redacted]
[redacted]
[redacted]
[redacted]

[redacted]

[redacted]
[redacted]
[redacted]
[redacted]
[redacted]
[redacted]
[redacted]
[redacted]
[redacted]
[redacted]
[redacted]
[redacted]

[redacted]

[redacted]
[redacted]
[redacted]
[redacted]
[redacted]
[redacted]
[redacted]
[redacted]
[redacted]
[redacted]
[redacted]
[redacted]
[redacted]
[redacted]
[redacted]
[redacted]
[redacted]
[redacted]
[redacted]
[redacted]
[redacted]
[redacted]
[redacted]
[redacted]
[redacted]
[redacted]
[redacted]
[redacted]
[redacted]
[redacted]
[redacted]
[redacted]
[redacted]
[redacted]
[redacted]
[redacted]
[redacted]
[redacted]
[redacted]

[redacted]

[redacted]
[redacted]
[redacted]
[redacted]
[redacted]
[redacted]
[redacted]
[redacted]
[redacted]
[redacted]
[redacted]
[redacted]
[redacted]
[redacted]
[redacted]
