# Supplementary material for: Povorcitinib for hidradenitis suppurativa: the randomized, double-blind, placebo-controlled STOP-HS1 and STOP-HS2 phase 3 trials
Source: Nat Med. 2026 Jul 23;32(8):3071–81. doi: 10.1038/s41591-026-04534-z (PMC13473019; doi:10.1038/s41591-026-04534-z)
Supplement: Supplementary file 2 [file 41591_2026_4534_MOESM2_ESM.pdf]

nature portfolio

Corresponding author(s): Christos C. Zouboulis, MD

Last updated by author(s): May 27, 2026

Reporting Summary

Nature Portfolio wishes to improve the reproducibility of the work that we publish. This form provides structure for consistency and transparency in reporting. For further information on Nature Portfolio policies, see our Editorial Policies and the Editorial Policy Checklist.

Statistics

For all statistical analyses, confirm that the following items are present in the figure legend, table legend, main text, or Methods section.

n/a	Confirmed	
☐	☒	The exact sample size (*n*) for each experimental group/condition, given as a discrete number and unit of measurement
☒	☐	A statement on whether measurements were taken from distinct samples or whether the same sample was measured repeatedly
☐	☒	The statistical test(s) used AND whether they are one- or two-sided<br>*Only common tests should be described solely by name; describe more complex techniques in the Methods section.*
☐	☒	A description of all covariates tested
☐	☒	A description of any assumptions or corrections, such as tests of normality and adjustment for multiple comparisons
☐	☒	A full description of the statistical parameters including central tendency (e.g. means) or other basic estimates (e.g. regression coefficient) AND variation (e.g. standard deviation) or associated estimates of uncertainty (e.g. confidence intervals)
☐	☒	For null hypothesis testing, the test statistic (e.g. *F*, *t*, *r*) with confidence intervals, effect sizes, degrees of freedom and *P* value noted<br>*Give P values as exact values whenever suitable.*
☒	☐	For Bayesian analysis, information on the choice of priors and Markov chain Monte Carlo settings
☐	☒	For hierarchical and complex designs, identification of the appropriate level for tests and full reporting of outcomes
☒	☐	Estimates of effect sizes (e.g. Cohen's *d*, Pearson's *r*), indicating how they were calculated

Our web collection on statistics for biologists contains articles on many of the points above.

Software and code

Policy information about availability of computer code

Data collection	IRT data collection was done via Suvoda platform. EDC data was collected on Medidata RAVE (2022.3.0). PRO data, including daily diary data, was collected using Signant Health TrialMAX platform. Photography and associated information were collected/managed via Canfield Clinical Services portal
Data analysis	All analyses were done with SAS® software (SAS Institute Inc, Cary, NC, USA; version 9·4). No custom code was created.

For manuscripts utilizing custom algorithms or software that are central to the research but not yet described in published literature, software must be made available to editors and reviewers. We strongly encourage code deposition in a community repository (e.g. GitHub). See the Nature Portfolio guidelines for submitting code & software for further information.

Data

Policy information about availability of data

All manuscripts must include a data availability statement. This statement should provide the following information, where applicable:

- Accession codes, unique identifiers, or web links for publicly available datasets
- A description of any restrictions on data availability
- For clinical datasets or third party data, please ensure that the statement adheres to our policy

Incyte Corporation (Wilmington, DE, USA) is committed to data sharing that advances science and medicine while protecting patient privacy. Qualified external scientific researchers may request anonymised datasets owned by Incyte for the purpose of conducting legitimate scientific research. Researchers may request anonymised datasets from any interventional study (except Phase 1 studies) for which the product and indication have been approved on or after 1 January 2020 in

at least one major market (eg. US, EU, JPN). Data will be available for request after the primary publication or 2 years after the study has ended. Information on Incyte's clinical trial data sharing policy and instructions for submitting clinical trial data requests are available at: https://www.incyte.com/Portals/0/Assets/Compliance%20and%20Transparency/clinical-trial-data-sharing.pdf?ver=2020-05-21-132838-960. The redacted study protocol is available in the Appendix.

Research involving human participants, their data, or biological material

Policy information about studies with human participants or human data. See also policy information about sex, gender (identity/presentation), and sexual orientation and race, ethnicity and racism.

Reporting on sex and gender	Sex was recorded as a biological variable based on clinical records at screening; included options were female and male. Demographic information about the number of females and males is provided in Table 1 and in Supplementary Table S1. Gender was not collected
Reporting on race, ethnicity, or other socially relevant groupings	Patient-reported race/ethnicity were collected to report patient population demographics (reported in Table 1 and in Supplementary Table S1), and were not analyzed further.
Population characteristics	Patient demographic and clinical characteristics are reported in Table 1 and in Supplementary Table S1.
Recruitment	The sponsor engaged with external stakeholders and accepted feedback to create the protocol. Sponsor selected appropriate sites based on evidence-based trial feasibility analysis, and chose sites with investigators who had required experience, adequate site infrastructure, institutional resources, and target population access. Participants were recruited by 103 sites (STOP-HS1) and 99 sites (STOP-HS2) across Australia, Canada, Europe, Japan, and the United States. Institutional ethics committee-approved recruitment strategies were used (full list of IRBs and ethics committees provided in the supplement). All participants had to meet protocol's eligibility criteria to be randomized; eligibility criteria were aligned with a population with moderate-to-severe HS and followed scientific consultation from health agencies, mainly FDA and EMA. These recruitment strategies minimized self-selection bias and are unlikely to impact results.
Ethics oversight	The study protocol and all amendments were reviewed and approved by the independent ethics committee or institutional review board at each study center. Due to the extent of this information, details on the institutes/organizations are provided in the Supplementary data. Additionally, this study was conducted in compliance with the Declaration of Helsinki, study protocol, Good Clinical Practices, and all applicable laws and regulations. All participants provided written informed consent before any study procedures were undertaken.

Note that full information on the approval of the study protocol must also be provided in the manuscript.

Field-specific reporting

Please select the one below that is the best fit for your research. If you are not sure, read the appropriate sections before making your selection.

- [x] Life sciences
- [ ] Behavioural & social sciences
- [ ] Ecological, evolutionary & environmental sciences

For a reference copy of the document with all sections, see nature.com/documents/nr-reporting-summary-flat.pdf

Life sciences study design

All studies must disclose on these points even when the disclosure is negative.

Sample size	Based on HiSCR50 response rates observed in a phase 2, randomised, dose-ranging study of povorcitinib, the assumed HiSCR50 response rates at Week 12 were 45% for povorcitinib 45 mg, 48% for povorcitinib 75 mg, and 30% for placebo. Under these assumptions, a sample size of 200 participants per group (600 total) provided 93% power to demonstrate statistical superiority of povorcitinib 75 mg over placebo and 81% power to demonstrate superiority of povorcitinib 45 mg over placebo, at a two-sided significance level of 0·025 using a chi-square test without continuity correction. Both trials were independently powered to test primary and key secondary outcomes.
Data exclusions	No data were excluded
Replication	Two phase 3 trials of identical design were conducted in parallel. Analyses were conducted with data as observed and with nonresponder imputation, with consistent results
Randomization	An interactive response technology system was used to assign patient identification numbers, track visits, randomise patients per prespecified parameters, and mask treatment group assignments. Patients were stratified by the total abscess, inflammatory nodule, and draining tunnel count (<11 or ≥11) and previous biologic use for HS (yes or no) and were randomly assigned 2:2:1:1 to once-daily povorcitinib 45 mg, povorcitinib 75 mg, placebo with crossover to povorcitinib 45 mg at Week 12, or placebo with crossover to povorcitinib 75 mg at Week 12; the overall treatment period was 54 weeks.
Blinding	Study was double blinded.

Reporting for specific materials, systems and methods

We require information from authors about some types of materials, experimental systems and methods used in many studies. Here, indicate whether each material, system or method listed is relevant to your study. If you are not sure if a list item applies to your research, read the appropriate section before selecting a response.

Materials & experimental systems

n/a	Involved in the study
☒	☐ Antibodies
☒	☐ Eukaryotic cell lines
☒	☐ Palaeontology and archaeology
☒	☐ Animals and other organisms
☐	☒ Clinical data
☒	☐ Dual use research of concern
☒	☐ Plants

Methods

n/a	Involved in the study
☒	☐ ChIP-seq
☒	☐ Flow cytometry
☒	☐ MRI-based neuroimaging

Clinical data

Policy information about clinical studies

All manuscripts should comply with the ICMJE guidelines for publication of clinical research and a completed CONSORT checklist must be included with all submissions.

Clinical trial registration: ClinicalTrials.gov NCT05620823, NCT05620836

Study protocol: The full study protocol and SAP are available in the supplement

Data collection: 103 sites (STOP-HS1) and 99 sites (STOP-HS2) across Australia, Canada, Europe, Japan, and the United States. STOP-HS1 was conducted 19Dec2022-23Dec2025, and STOP-HS2 22Feb2023-21Nov2025. All study investigators and sites are listed in the supplement

Outcomes: Primary and secondary outcomes were predefined based on typical endpoints in registrational clinical trials for HS and with scientific consultation with health agencies, mainly FDA and EMA.

The primary endpoint in each trial was HiSCR50 at Week 12, defined as a reduction of at least 50% from baseline in total abscess and inflammatory nodule count, with no increase from baseline in abscess or draining tunnel count. Key secondary endpoints were HiSCR75 (reduction of ≥75% from baseline in total abscess and inflammatory nodule count, with no increase from baseline in abscess or draining tunnel count) at Week 12; skin pain response (among patients with baseline Skin Pain NRS score of at least 3) at Week 12, assessed either as the percentage of patients who achieved a reduction of at least 3 points from baseline (for US Food and Drug Administration and aligned countries) or as Skin Pain NRS30 (defined as a reduction of ≥30% and at least 1 unit from baseline; for European Medicines Agency and aligned countries); and flare occurrence (defined as a ≥25% and ≥2-unit increase in abscess and inflammatory nodule count relative to baseline) by Week 12.
Secondary endpoints included the percentage of patients with baseline FACIT-F score no more than 48 achieving at least a 4-point increase (improvement) at Week 12 and the percentage achieving HiSCR50 and HiSCR75 at Week 12 and maintaining it at each visit during the extension period. At Weeks 24 and 54, the percentages of patients achieving HiSCR50, HiSCR75, Skin Pain response, or experiencing a flare were assessed. Additionally, change from baseline in DLQI and change/percentage change from baseline in lesion counts (abscess, inflammatory nodule, draining tunnel) were evaluated at each visit through Week 54.

Plants

Seed stocks: *Report on the source of all seed stocks or other plant material used. If applicable, state the seed stock centre and catalogue number. If plant specimens were collected from the field, describe the collection location, date and sampling procedures.*

Novel plant genotypes: *Describe the methods by which all novel plant genotypes were produced. This includes those generated by transgenic approaches, gene editing, chemical/radiation-based mutagenesis and hybridization. For transgenic lines, describe the transformation method, the number of independent lines analyzed and the generation upon which experiments were performed. For gene-edited lines, describe the editor used, the endogenous sequence targeted for editing, the targeting guide RNA sequence (if applicable) and how the editor was applied.*

Authentication: *Describe any authentication procedures for each seed stock used or novel genotype generated. Describe any experiments used to assess the effect of a mutation and, where applicable, how potential secondary effects (e.g. second site T-DNA insertions, mosiacism, off-target gene editing) were examined.*
